# *Azospirillum* Genomes Reveal Transition of Bacteria from Aquatic to Terrestrial Environments

**DOI:** 10.1371/journal.pgen.1002430

**Published:** 2011-12-22

**Authors:** Florence Wisniewski-Dyé, Kirill Borziak, Gurusahai Khalsa-Moyers, Gladys Alexandre, Leonid O. Sukharnikov, Kristin Wuichet, Gregory B. Hurst, W. Hayes McDonald, Jon S. Robertson, Valérie Barbe, Alexandra Calteau, Zoé Rouy, Sophie Mangenot, Claire Prigent-Combaret, Philippe Normand, Mickaël Boyer, Patricia Siguier, Yves Dessaux, Claudine Elmerich, Guy Condemine, Ganisan Krishnen, Ivan Kennedy, Andrew H. Paterson, Victor González, Patrick Mavingui, Igor B. Zhulin

**Affiliations:** 1 CNRS, UMR 5557, Ecologie Microbienne, Université de Lyon, Villeurbanne, France; 2 BioEnergy Science Center, University of Tennessee–Oak Ridge National Laboratory, Oak Ridge, Tennessee, United States of America; 3 Genome Science and Technology Program, University of Tennessee–Oak Ridge National Laboratory, Oak Ridge, Tennessee, United States of America; 4 Department of Biochemistry, Cell and Molecular Biology, University of Tennessee, Knoxville, Tennessee, United States of America; 5 Department of Microbiology, University of Tennessee, Knoxville, Tennessee, United States of America; 6 Chemical Sciences Division, Oak Ridge National Laboratory, Oak Ridge, Tennessee, United States of America; 7 Plant Genome Mapping Laboratory, University of Georgia, Athens, Georgia, United States of America; 8 Institut de Génomique, CEA, Génoscope, Evry, France; 9 Laboratoire d'Analyse Bioinformatique en Génomique et Métabolisme CNRS UMR8030, CEA, Génoscope, Evry, France; 10 UMR5100 Laboratoire de Microbiologie et Génétique Moléculaire, CNRS-Université Paul Sabatier, Toulouse, France; 11 Institut des Sciences du Végétal, UPR 2355, CNRS, Gif-sur-Yvette, France; 12 Département de Microbiologie, BMGE, Institut Pasteur, Paris, France; 13 CNRS, UMR5240, Microbiologie Adaptation et Pathogénie, Université de Lyon, Villeurbanne, France; 14 Faculty of Agriculture, Food, and Natural Resources, The University of Sydney, Sydney, Australia; 15 Centro de Ciencias Genómicas, Universidad Nacional Autónoma de México, Cuernavaca, México; 16 Computer Science and Mathematics Division, Oak Ridge National Laboratory, Oak Ridge, Tennessee, United States of America; Progentech, United States of America

## Abstract

Fossil records indicate that life appeared in marine environments ∼3.5 billion years ago (Gyr) and transitioned to terrestrial ecosystems nearly 2.5 Gyr. Sequence analysis suggests that “hydrobacteria” and “terrabacteria” might have diverged as early as 3 Gyr. Bacteria of the genus *Azospirillum* are associated with roots of terrestrial plants; however, virtually all their close relatives are aquatic. We obtained genome sequences of two *Azospirillum* species and analyzed their gene origins. While most *Azospirillum* house-keeping genes have orthologs in its close aquatic relatives, this lineage has obtained nearly half of its genome from terrestrial organisms. The majority of genes encoding functions critical for association with plants are among horizontally transferred genes. Our results show that transition of some aquatic bacteria to terrestrial habitats occurred much later than the suggested initial divergence of hydro- and terrabacterial clades. The birth of the genus *Azospirillum* approximately coincided with the emergence of vascular plants on land.

## Author Summary

Genome sequencing and analysis of plant-associated beneficial soil bacteria *Azospirillum* spp. reveals that these organisms transitioned from aquatic to terrestrial environments significantly later than the suggested major Precambrian divergence of aquatic and terrestrial bacteria. Separation of *Azospirillum* from their close aquatic relatives coincided with the emergence of vascular plants on land. Nearly half of the *Azospirillum* genome has been acquired horizontally, from distantly related terrestrial bacteria. The majority of horizontally acquired genes encode functions that are critical for adaptation to the rhizosphere and interaction with host plants.

## Introduction

Fossil records indicate that life appeared in marine environments ∼3.5–3.8 billion years ago (Gyr) [1] and transitioned to terrestrial ecosystems ∼2.6 Gyr [2]. The lack of fossil records for bacteria makes it difficult to assess the timing of their transition to terrestrial environments; however sequence analysis suggests that a large clade of prokaryotic phyla (termed “terrabacteria”) might have evolved on land as early as 3 Gyr, with some lineages later reinvading marine habitats [3]. For example, cyanobacteria belong to the terrabacterial clade, but one of its well-studied representatives, *Prochlorococcus*, is the dominant primary producer in the oceans [4].

**Figure 1:**
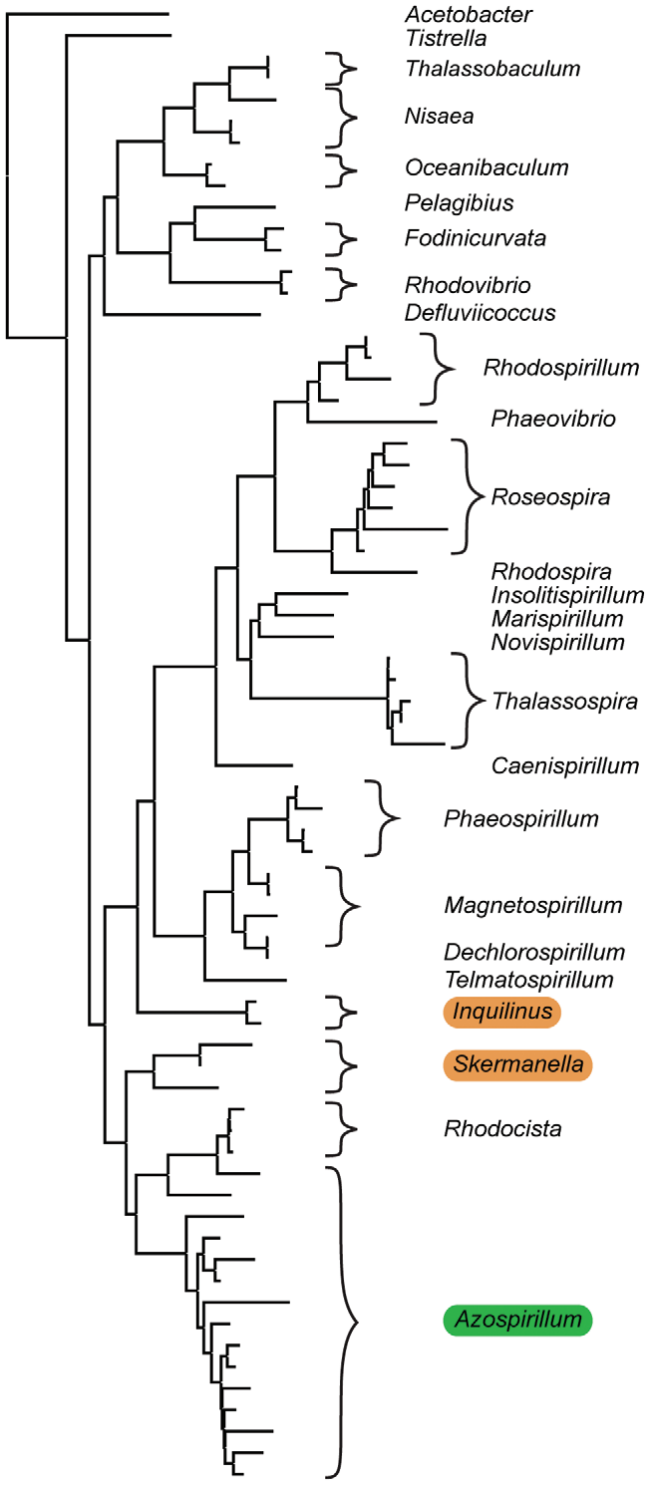
Habitats of *Azospirillum* and its closest aquabacterial relatives. A maximum-likelihood tree built from 16S rRNA sequences from members of *Rhodospirillaceae*. *Acetobacter acetii*, a member of the same order *Rhodospirillales*, but a different family, *Acetobacteriaceae*, is shown as an outgroup. Aquatic inhabitants are not highlighted; terrestrial are highlighted in brown and plant-associated *Azospirillum* is highlighted in green. See Table S1 for details.

Bacteria of the genus *Azospirillum* are found primarily in terrestrial habitats, where they colonize roots of important cereals and other grasses and promote plant growth by several mechanisms including nitrogen fixation and phytohormone secretion [5], [6]. *Azospirillum* belong to proteobacteria, one of the largest groups of “hydrobacteria”, a clade of prokaryotes that originated in marine environments [3]. Nearly all known representatives of its family *Rhodospirillaceae* are found in aquatic habitats (Figure 1 and Table S1) suggesting that *Azospirillum* represents a lineage which might have transitioned to terrestrial environments much later than the Precambrian split of “hydrobacteria” and “terrabacteria”. To obtain insight into how bacteria transitioned from marine to terrestrial environments, we sequenced two well studied species, *A. brasilense* and *A. lipoferum*, and a third genome of an undefined *Azospirillum* species became available while we were carrying out this work [7].

## Results/Discussion

In contrast to the genomes of their closest relatives (other *Rhodospirillaceae*), the three *Azospirillum* genomes are larger and are comprised of not one, but seven replicons each (Figure S1 and Table 1). Multiple replicons have been previously suggested for various *Azospirillum* strains [8]. The largest replicon in each genome has all characteristics of a bacterial chromosome, whereas the smallest is a plasmid. Five replicons in the genomes of *A. lipoferum* and *Azospirillum* Sp. 510 can be defined as “chromids” (intermediates between chromosomes and plasmids [9]), whereas in *A. brasilense* only three replicons are “chromids” (Tables S2 and S3). While multiple replicons, and chromids specifically, are not unusual in proteobacteria [9], [10], *Azospirillum lipoferum* has the largest number of chromids among all prokaryotes sequenced to date [9] indicating a potential for genome plasticity.

**Table 1:** General features of *Azospirillum* genomes.

	*Azospirillum lipoferum* 4B	*Azospirillum brasilense* Sp245
**Sequence length**	6846400 bp	7530241 bp
**GC content (%)**	67.67	68.49
**Number of Contigs**	7	67
**Total number of genes**	6354	7962
**Total number of CDS**	6233	7848
**Protein coding regions (%)**	87.02	85.62
**Number of rRNA operons**	9	9
**Number of tRNA genes**	79	81
**Genes with functional assignment**	4125	4770
**Genes with general function prediction only**	657	746
**Genes of unknown function**	1451	2332

**Figure 2:**
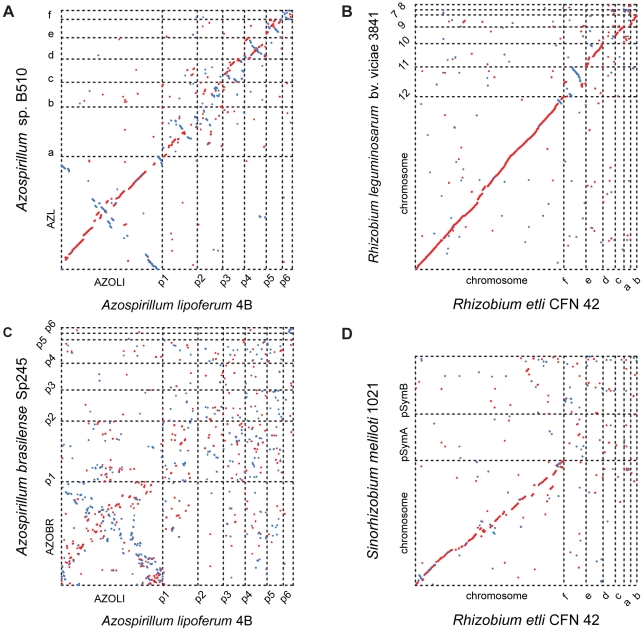
Whole-genome alignments for *Azospirillum* and related multi-replicon rhizobial species. Relative distances between genomes (calculated from a concatenated ribosomal protein tree): *A. lipoferum* 4B to *Azospirillum* sp.510 – 0.01; *Rhizobium etli to Rhizobium leguminosarum* – 0.02; *A. lipoferum* 4B to *A. brasilense* Sp245 – 0.10; *Rhizobium etli* to *S. meliloti* – 0.11.

Comparisons among the three genomes reveal further evidence of extraordinary genome plasticity in *Azospirillum*, a feature that has also been suggested by some experimental data [11]. We found very little synteny between replicons of *Azospirillum* species. The genetic relatedness among *Azospirillum* strains is comparable to that of rhizobia, other multi-replicon alpha-proteobacteria (Table S4). Surprisingly, we found substantially more genomic rearrangement within *Azospirillum* genomes than within rhizobial genomes (Figure 2) that are suggested to exemplify genome plasticity in prokaryotes [10]. This could be a consequence of many repetitive sequences and other recombination hotspots (Tables S4 and S5), although the detailed mechanisms underlying such extraordinary genome plasticity remain incompletely understood.

**Figure 3:**
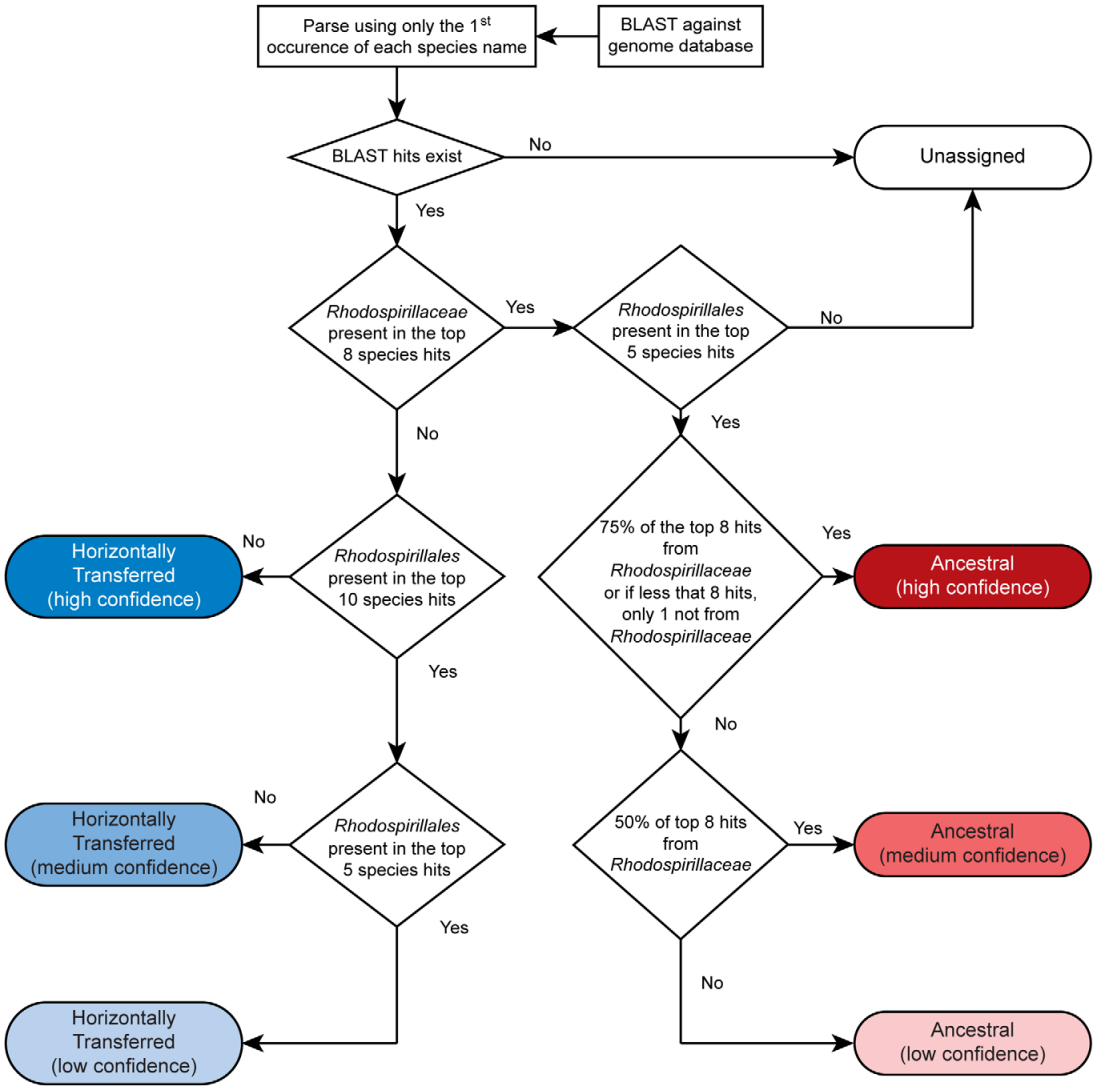
Scheme for detecting ancestral and horizontally transferred genes. See Materials and Methods for details.

**Figure 4:**
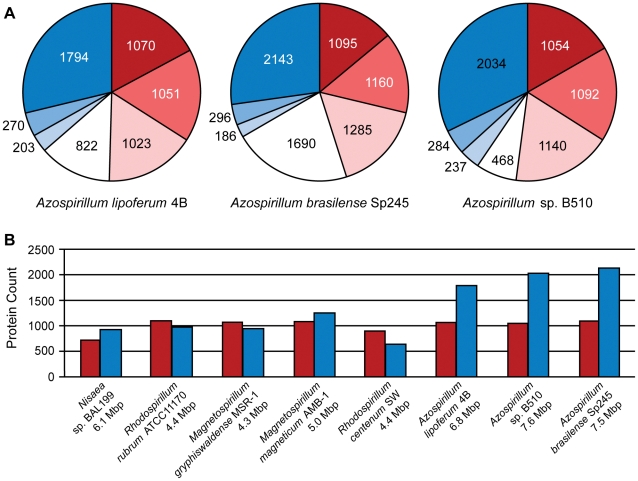
Ancestral (red) and horizontally transferred (blue) genes in *Azospirillum*. (A) Proportion of ancestral and horizontally transferred genes predicted in three *Azospirillum* genomes with varying confidence: intensity of color shows high (dark), medium (medium) and low (light) levels of confidence for predictions (see Materials and Methods). Genes that cannot be assigned using this protocol are shown in white. Majority of these genes are unique to each species and have no identifiable homologs; thus, they are likely the result of HGT. (B) Proportion of ancestral and horizontally transferred genes in genomes of *Rhodospirillaceae*. Only genes that were predicted with high confidence are shown.

**Figure 5:**
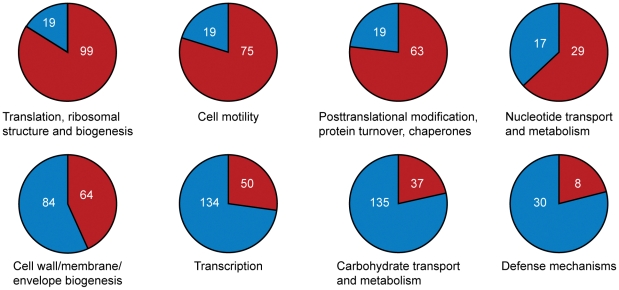
Functional categories for *A. lipoferum* 4B genes enriched in ancestral (top) and horizontally transferred (bottom) genes. Only genes that were predicted with high confidence are shown.

Which genes does *Azospirillum* share with its aquatic relatives, and what is the origin of its additional genes? To answer this question, we developed a robust scheme for detecting ancestral and horizontally transferred (HGT) genes (Figure 3) using bioinformatics tools, then classified most protein coding genes in the *Azospirillum* genomes as ancestral or horizontally transferred with quantified degrees of confidence (Figure 4A and Table S6). Remarkably, nearly half of the genes in each *Azospirillum* genome whose origins can be resolved appeared to be horizontally transferred. As a control, we subjected the genomes of other *Rhodospirillaceae* to the same analysis, finding a substantially lower HGT level in aquatic species, while the number of ancestral genes in all organisms was comparable (Figure 4B). Horizontally transferred genes are frequently expendable, whereas ancestral genes usually serve ‘house-keeping’ functions and are conserved over long evolutionary distances [12]. To further validate our classifications, we determined functional assignments of genes in each of the two categories using the COG database [13]. The ‘ancestral’ set primarily contained genes involved in ‘house-keeping’ functions such as translation, posttranslational modification, cell division, and nucleotide and coenzyme metabolism (Figure 5). The HGT set contained a large proportion of genes involved in specific dispensable functions, such as defense mechanisms, cell wall biogenesis, transport and metabolism of amino acids, carbohydrates, inorganic ions and secondary metabolites (Figure 5 and Table S6). This is consistent with the role of HGT in adaptation to the rhizosphere, an environment rich in amino acids, carbohydrates, inorganic ions and secondary metabolites excreted by plant roots [14].

**Figure 6:**
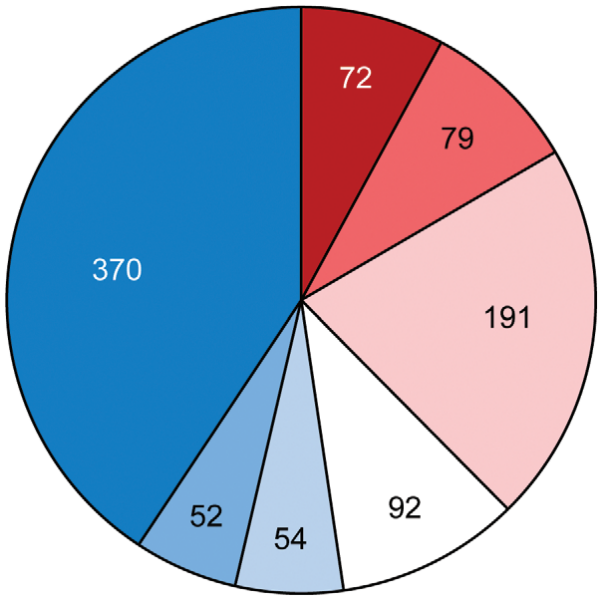
Proportion of ancestral (red) and horizontally transferred (blue) genes involved in adaptation of *Azospirillum* to the rhizosphere and its interaction with host plants (see Table S7 for details). Color intensity indicates high (dark), medium (medium) and low (light) confidence levels for prediction (see Materials and Methods for details).

Such an extraordinary high level of HGT in *Azospirillum* genomes leads us to hypothesize that it was a major driving force in the transition of these bacteria from aquatic to terrestrial environments and adaptation to plant hosts. This process was likely promoted by conjugation and transduction, as *Azospirillum* hosts phages and notably a Gene Transfer Agent [15]; this should have also resulted in loss of ancestral ‘aquatic’ genes that are not useful in the new habitat. Indeed, one of the defining features of *Rhodospirillaceae*, photosynthesis (responsible for the original taxonomic naming of these organisms – purple bacteria) is completely absent from *Azospirillum*. We have analyzed origins of genes that are proposed to be important for adaptation to the rhizosphere and interactions with the host plant [6], [16]. Consistent with our hypothesis, the majority of these genes were predicted to be horizontally transferred (Figure 6 and Table S7). It is important however to stress that plant-microbe interactions involve a complex interplay of many functions that are determined by both ancestral and horizontally acquired genes.

**Figure 7:**
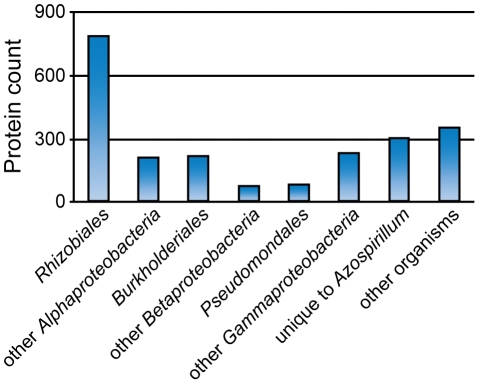
Taxonomic distribution of the best BLAST hits for predicted HGT in *Azospirillum*.

What was the source of horizontally transferred genes? A large proportion of genes that we assigned as HGT show relatedness to terrestrial proteobacteria, including representatives of *Rhizobiales* (distantly related alpha-proteobacteria) and *Burkholderiales* (beta-proteobacteria) (Figure 7) that are soil and plant-associated organisms. In the absence of fossil data, it is nearly impossible to determine the time of divergence for a specific bacterial lineage; however, a rough approximation (1–2% divergence in the 16S rRNA gene equals 50 Myr [17]) suggests that azospirilla might have diverged from their aquatic *Rhodospirillaceae* relatives 200–400 Myr (Table S8). This upper time limit coincides with the initial major radiation of vascular plants on land and evolution of plant roots, to 400 Myr [18], [19]. Grasses, the main plant host for *Azospirillum*, appeared much later, about 65–80 Myr [20], which is consistent with reports that azospirilla can also colonize plants other than grasses.

**Figure 8:**
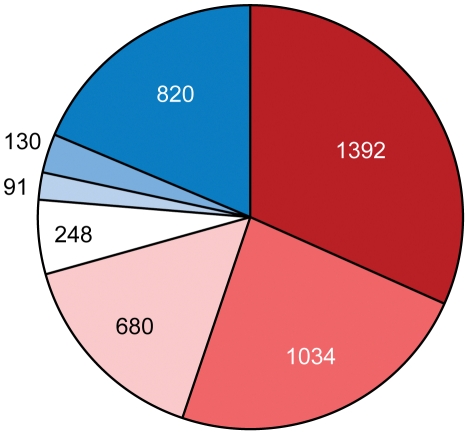
Proportion of ancestral (red) and horizontally transferred (blue) genes in the proteomics data for *A. lipoferum* 4B. Color intensity indicates high (dark), medium (medium) and low (light) confidence levels for prediction. See Table S9 for details.

Using a global proteomics approach we have found that many HGT genes including nearly 1/3 of those that are common to all three *Azospirillum* genomes were expressed under standard experimental conditions and under nitrogen limitation, a condition usually encountered in the rhizosphere of natural ecosystems (Figure 8 and Table S9).

Genes that differentiated the *Azospirillum* species from one another and from their closest relatives are implicated in specializations, such as plant colonization. *Azospirillum* and closely related *Rhodospirillum centenum* both possess multiple chemotaxis operons and are model organisms to study chemotaxis [21], [22]. Interestingly, operon 1, which controls chemotaxis in *R. centenum* [22], plays only a minor role in chemotaxis of *A. brasilense* [23]. All three *Azospirillum* species possess three chemotaxis operons that are orthologous to those in *R. centenum*; however, they also have additional chemotaxis operons that are absent from their close aquatic relative (Figure S2 and Tables S6 and S10). Additional chemotaxis operons have been acquired by azospirilla prior to each speciation event yielding 4, 5 and 6 chemotaxis systems in *A. brasilense* Sp245, *A. lipoferum* 4B and *Azospirillum* sp. 510, respectively. These stepwise acquisitions have made the latter organism an absolute “chemotaxis champion”, with 128 chemotaxis genes, more than any other prokaryote sequenced to date (data from MiST database [24]). Recent analysis showed the prevalence of chemotaxis genes in the rhizosphere [25]. We have determined that the dominant chemotaxis genes in this dataset belong to a specific chemotaxis class F7 [26] (Figure S3 and Table S11). Strikingly, it is this F7 system that is shared by all *Azospirillum* and is predicted to have been transferred horizontally into their common ancestor.

**Figure 9:**
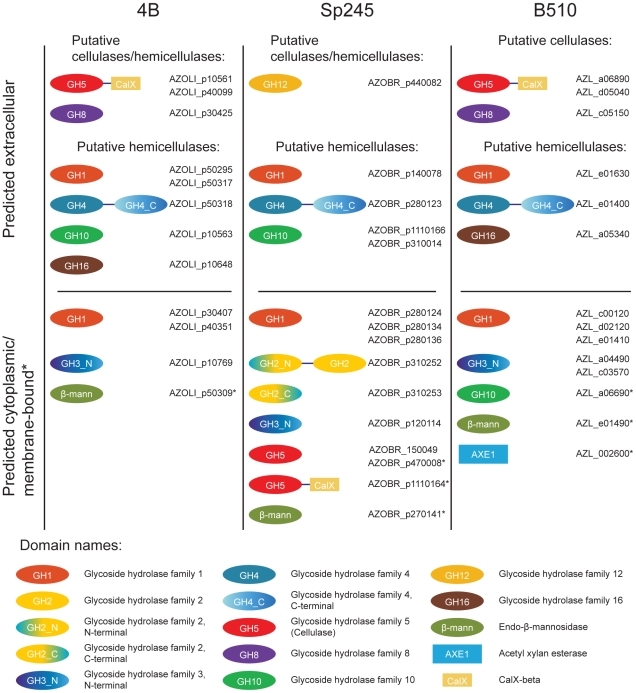
Glycoside hydrolases in *Azospirillum* with a potential to degrade the plant cell wall. The genomes of *Azospirillum* encode from 26 to 34 glycoside hydrolases that belong to various CAZy [54] families (Table S12). Total number of glycoside hydrolases in *Azospirillum* species is similar to that in a soil cellulolytic bacterium *Thermobifida fusca* [61]. All three species have orthologs of putative cellulases (AZOLI_p10561, AZOLI_p40099; AZOBR_p1110164; AZL_a06890; AZL_d05040) with unique domain architecture: GH_5 – CalX-β. The other two putative cellulases (AZOBR_150049, AZOBR_p470008) are found only in *A. brasilense*. In addition to putative cellulases, *Azospirillum* species encode putative extracellular endoglucanases that may be involved in cellulose/hemicellulose degradation. For example, glycoside hydrolases that belong to family GH8 (AZOLI_p30425, AZL_c05150), which are known for a wide range of cellulose-containing substrates [62]–[64] and family GH12 (AZOBR_p440082). All three species are predicted to secrete a number of putative hemicellulases, that belong to glycoside hydrolase families GH1 (β-glycosidases), GH4 (glucuronidase/galactosidase), GH10 (endo-xylanases) and GH16 (licheninases) (Table S12). CAZy families were assigned as described in Materials and Methods.

**Figure 10:**
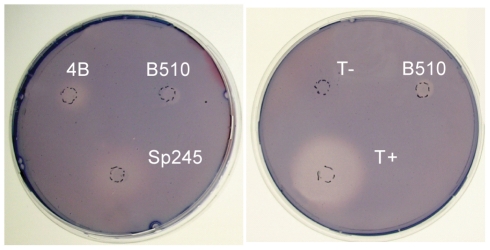
Cellulolytic activity of *A. brasilense* Sp245 cells. All three *Azospirillum* species are shown on the left panel. Known cellulose degrader (*Dickeya dadantii* 3937, T+) and non-degrader (*Agrobacterium tumefaciens* NT1, T-) are shown as positive and negative controls, respectively.

**Figure 11:**
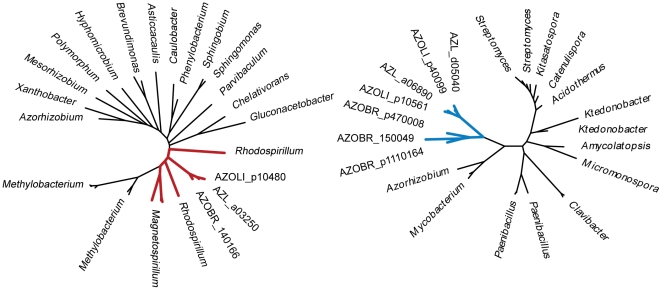
Phylogenetic trees for thiamine synthetase (left) and cellulase (right). The trees exemplify ancestral and HGT relationships, respectively, that were predicted with high confidence. Trees were built from aligned sequences of the *A. brasilense* Sp245 query and twenty most similar sequences determined by BLAST. The thiamine synthetase set contains only representatives of alpha-proteobacteria including *Rhodospirillaceae* (shown in red). The cellulase set consists of representatives of Actinobacteria, Firmicutes, and Chloroflexi with only one representative of alpha-proteobacteria other than *Azospirillum* (that are shown in blue, highlighting their HGT origin), *Azorhizobium*.

Cellulolytic activity may be crucial to the ability of some azospirilla to penetrate plant roots [27]. All *Azospirillum* genomes encode a substantial number of glycosyl hydrolases that are essential for decomposition of plant cell walls (Figure 9). The total number of putative cellulases and hemicellulases in azospirilla is comparable to that in soil cellulolytic bacteria (Table S12) and most of them are predicted to be acquired horizontally (Table S6). We tested three *Azospirillum* species and found detectable cellulolytic activity in *A. brasilense* Sp245 (Figure 10). The *A. brasilense* Sp245 genome contains three enzymes encoded by AZOBR_p470008, AZOBR_p1110164 and AZOBR_150049 (Figure 11) that are orthologous to biochemically verified cellulases. We propose that these and other horizontally transferred genes (*e.g.* glucuronate isomerase, which is involved in pectin decomposition) contributed to establishing *A. brasilense* Sp245 as a successful endophyte [27]. Interestingly, another successful endophytic bacterium, *Herbaspirillum seropedicae*, lacks the genes coding for plant cell wall degradation enzymes [28] indicating that endophytes may use very different strategies for penetrating the plant.

**Figure 12:**
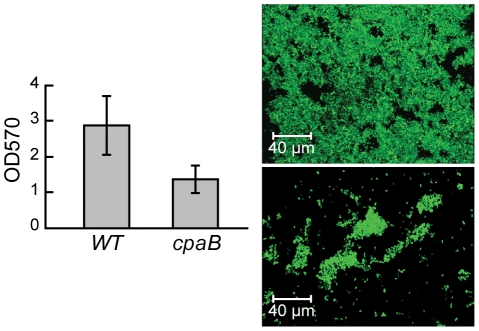
TAD pili in *A. brasilense* are required for biofilm formation. Quantification of biofilm formed by wild type (wt) and a pili mutant (*cpaB*) on glass using crystal violet staining (left panel) and 3-D-reconstruction of the biofilm formed by wild type (top) and a pili mutant (bottom) by confocal microscopy (right panel).

Attachment, another function important for plant association by *Azospirillum*, was also acquired horizontally. Type IV pili is a universal feature for initiating and maintaining contact with the plant host [29], [30]. The genome of *A. brasilense* Sp245 lacks genes coding for Type IV pili, but encodes a set of genes for TAD (tight adhesion) pili that are known to be HGT prone [31]. In our analysis, TAD pili were confidently predicted to be a result of HGT (Table S6). We show that a mutant deficient in TAD pili had a severe defect in attachment and biofilm formation (Figure 12) suggesting a role for TAD in plant-microbe association.

### Concluding remarks

Horizontal gene transfer has been long recognized as a major evolutionary force in prokaryotes [12]. Its role in the emergence of new pathogens and adaptation to environmental changes is well documented [32]. While other recent studies indicate that HGT levels in natural environments may reach as much as 20% of a bacterial genome [33], our data suggest that HGT has affected nearly 50% of the *Azospirillum* genomes, in close association with dramatic changes in lifestyle necessary for transition from aquatic to terrestrial environments and association with plants. Emergence of these globally distributed plant-associated bacteria, which appear to coincide with radiation of land plants and root development, likely has dramatically changed the soil ecosystem.

## Materials and Methods

### Genome sequencing and assembly

The genome of *Azospirillum lipoferum* 4B was sequenced by the whole random shotgun method with a mixture of ∼12X coverage of Sanger reads, obtained from three different libraries, and ∼18X coverage of 454 reads. Two plasmid libraries of 3 kb (A) and 10 kb (B), obtained by mechanical shearing with a Hydroshear device (GeneMachines, San Carlos, California, USA), were constructed at Genoscope (Evry, France) into pcDNA2.1 (Invitrogen) and into the pCNS home vector (pSU18 modified, Bartolome et al.[34]), respectively. Large inserts (40 kb) (C) were introduced into the PmlI site of pCC1FOS. Sequencing with vector-based primers was carried out using the ABI 3730 Applera Sequencer. A total of 95904 (A), 35520 (B) and 15360 (C) reads were analysed and assembled with 504591 reads obtained with Genome Sequencer FLX (Roche Applied Science). The Arachne “HybridAssemble” version (Broad institute, MA) combining 454 contigs with Sanger reads was used for assembly. To validate the assembly, the Mekano interface (Genoscope), based on visualization of clone links inside and between contigs, was used to check the clones coverage and misassemblies. In addition, the consensus was confirmed using Consed functionalities (www.phrap.org), notably the consensus quality and the high quality discrepancies. The finishing step was achieved by PCR, primer walks and transposon bomb libraries and a total of 5460 sequences (58, 602 and 4800 respectively) were needed for gap closure and quality assessment.

The genome of strain *Azospirillum brasilense* Sp245 was sequenced by the whole random shotgun method with a mixture of ∼10X coverage of Sanger reads obtained from three different libraries and ∼25X coverage of 454 reads. A plasmid library of 3 kb, obtained by mechanical shearing with a Hydroshear device (GeneMachines, San Carlos, California, USA), were constructed at Plant Genome Mapping Laboratory (University of Georgia, USA) into pcDNA2.1 vector (Invitrogen). Large inserts (40 kb) were introduced into the PmlI site of pCC1FOS. Sequencing with vector-based primers was carried out using the ABI 3730 Applera Sequencer. The Arachne “HybridAssemble” version combining 454 contigs with Sanger reads was used for assembly. Contig scaffolds were created using Sequencher (Gene Codes) and validated using clone link inside and between contigs.

### Genome annotation

AMIGene software [35] was used to predict coding sequences (CDSs) that were submitted to automatic functional annotation [36]. The resulting 6233 *A. lipoferum* 4B CDSs and 7848 *A. brasilense* Sp245 CDSs were assigned a unique identifier prefixed with “AZOLI” or “AZOBR” according to their respective genomes. Putative orthologs and synteny groups were computed between the sequenced genomes and 650 other complete genomes downloaded from the RefSeq database (NCBI) using the procedure described in Vallenet et al. [36]. Manual validation of the automatic annotation was performed using the MaGe (Magnifying Genomes) interface. IS finder (www-is.biotoul.fr) was used to annotate insertion sequences [37]. The *A. lipoferum* 4B nucleotide sequence and annotation data have been deposited to EMBL databank under accession numbers: FQ311868 (chromosome), FQ311869 (p1), FQ311870 (p2), FQ311871 (p3), FQ311872 (p4), FQ311873 (p5), FQ311874 (p6). The *A. brasilense* Sp245 nucleotide sequence and annotation data have been deposited at EMBL databank under accession numbers: HE577327 (chromosome), HE577328 (p1), HE577329 (p2), HE577330 (p3), HE577331 (p4), HE577332 (p5), HE577333 (p6). In addition, all the data (i.e., syntactic and functional annotations, and results of comparative analysis) were stored in a relational database, called AzospirilluScope [36], which is publicly available at http://www.genoscope.cns.fr/agc/mage/microscope/about/collabprojects.php?P_id=39.

### Computational genomics/bioinformatics

BLAST searches were performed using NCBI toolkit version 2.2.24+ [38]. Multiple sequence alignments were built using the L-INS-i algorithm of MAFFT [39] with default parameters. Phylogenetic tree construction was performed using PhyML [40] with default parameters unless otherwise specified. 16S rRNA sequences were retrieved from the Ribosomal Database Project [41].

A concatenated ribosomal protein tree was constructed from sequenced members of alpha-proteobacteria with a 98% 16S rRNA sequence identity cutoff to limit overrepresentation. The following ribosomal proteins were used: L3, L5, L11, L13, L14, S3, S7, S9, S11, and S17. The proteins were identified using corresponding Pfam models and HMMER [42] searches against the genomes of sequenced alpha-proteobacteria selected above. The sequences were aligned and concatenated. GBlocks [43] with default parameters was used to reduce the number of low information columns. The tree was constructed using PhyML with the following options: empirical amino acid frequencies, 4 substitution categories, estimated gamma distribution parameter, and NNI tree topology search.

### Assignment of gene ancestry

Protein sequences queries from all 3 *Azospirillum* genomes were used in BLAST searches against the non-redundant microbial genome set constructed by Wuichet and Zhulin [26] supplemented with sequenced members of *Rhodospirillales* absent in the original set (*Acetobacter pasteurianus* IFO 3283-01, *alpha proteobacterium* BAL199, *Magnetospirillum gryphiswaldense* MSR-1, and *Magnetospirillum magnetotacticum* MS-1). E-value cutoff of 10∧−4 was used.

Only the first occurrence of each species was used in ancestry assignment. Proteins were assigned as being ancestral or horizontally transferred, with varying degrees of confidence, based on the presence of members of *Rhodospirillales* and *Rhodospirillaceae* in the top eight BLAST hits. Ancestral assignment was based on the top 8 hits, based on the number of *Rhodospirillaceae* genomes in the database: 2 *Azospirillum*, 3 *Magnetospirillum*, 2 *Rhodospirillum*, and *Nisaea* sp. BAL199, excluding the organism on which ancestry assignment is being performed. High confidence ancestral proteins have at least 6 of the top 8 species belonging to *Rhodospirillales* or all but 1, if the BLAST result had less than 8 species. This rule allows for 1–2 independent events of HGT from *Rhodospirillales* to other distantly related species. Medium confidence ancestral proteins have at least 4 *Rhodospirillaceae* in the top 8. Low confidence ancestral proteins have at least 1 *Rhodospirillaceae* in the top 8, excluding hits to other *Azospirillum* genomes. High confidence horizontally transferred proteins have 0 hits to *Rhodospirillales* in the top 10, excluding hits to other *Azospirillum* genomes. Medium confidence horizontally transferred proteins have 0 hits to *Rhodospirillales* in the top 5, excluding hits to other *Azospirillum* genomes. Low confidence horizontally transferred proteins have 0 hits to *Rhodospirillaceae* in the top 8, excluding hits to other *Azospirillum* genomes. Unassigned proteins either have no BLAST hits outside *Azospirillum*, or simultaneously classify as medium confidence horizontally transferred and medium or low confidence ancestral.

### Proteomics

#### Cell growth

*Azospirillum brasilense strain Sp245:* Overnight starter cultures (5 mL) were inoculated from fresh plates. Starter cultures were grown overnight at 27°C in a shaking water bath in minimal media containing malate as carbon source and ammonium chloride as nitrogen source. Cells were pelleted from starter cultures and washed with appropriate growth media. Base media for all cultures was minimal media (MMAB) [44] with 20 mM malate as carbon source, ammonium chloride as nitrogen source where appropriate, and molybdate. Starter cultures were resuspended with appropriate media and used to inoculate 250 mL cultures for nitrogen-fixing growth, or 500 ml cultures for non-nitrogen-fixing growth. Nitrogen fixation requires a great deal of energy and continuous optimal oxygen concentration, so growth of nitrogen fixing cells is slower than those growing in nitrogen sufficient conditions. Cells grown under nitrogen fixing conditions exhibit a doubling time of 170 minutes while control (non nitrogen fixing) cells have a doubling time of 120 minutes [21]. Further, OD of cells grown under nitrogen fixing cultures never reaches high levels, tending to level off at or below an OD600 of 0.2–0.3 [21]. Therefore, each growth condition was optimized as follows. For nitrogen-fixing cultures, nitrogen gas was sparged through the head space of the media bottle through the serum port, and sufficient air was injected to give a final oxygen content in the head space of 2%; cultures were grown at 25°C without shaking to early log phase (OD_600_ = 0.1–0.2) to minimize exposure to high levels of oxygen, as *Azospirillum* species are microaerophilic diazotrophs. Non-nitrogen fixing cultures were grown under optimum growth conditions (shaking and in presence of ammonium) at 25°C on an orbital shaker to mid-log phase (OD_600_ = 0.5–0.6). Cells were harvested by centrifugation at 8000 rpm for 10 minutes, washed twice with 50 mM Tris (pH 7.9), then pelleted by centrifugation at 8000 rpm for 10 minutes, and stored at −80 C. Cell pellets from two biological replicates were pooled for subsequent proteome preparation. *Azospirillum lipoferum*: Growth conditions were as described above for *A. brasilense* Sp245, except that cells were grown in MMAB media supplemented with 1 mg/L D-biotin.

#### Proteome preparation for LC/LC-MS/MS

Frozen cell pellets (0.1 g for each sample) were resuspended at a rate of 500 µl lysis buffer/0.1 g wet cell pellet weight in lysis buffer of 6 M guanidine hydrochloride, 10 mM DTT solubilized in 50 mM Tris-HCl, 10 mM CaCl_2_ [45]. Resuspended cells were then further lysed by sonication. Lysate was centrifuged at 18,000 g for 20 minutes to clear cellular debris. Supernatant was collected for tryptic digestion. 10 mM DTT was added and lysate was incubated at 60°C for 1 hour. Lysate was then diluted 6-fold with trypsin digestion buffer (50 mM Tris-HCl, 10 mM CaCl_2_, 10 mM DTT, pH 7.9) and 20 µg sequencing-grade trypsin (Promega, Madison, WI) was added to each sample. Samples were incubated overnight at 37°C with gentle rotation. An additional 20 µg of trypsin was added the following morning and samples were subsequently incubated for an additional 5–6 hours at 37°C with gentle rotation. Digestion was halted by addition of 5 µl formic acid to the 5 ml lysate. Samples were then desalted using Sep-Pak Plus C-18 solid phase extraction (Waters, Milford, MA) following manufacturer's recommendations, and subsequently concentrated and solvent-exchanged into 100% HPLC-grade H_2_O, 0.1% formic acid using vacuum centrifugation (Savant, Thermo Scientific). Samples were aliquoted into 40 µL volumes and stored at −80°C until analysis.

#### LC/LC-MS/MS analysis

Proteome samples were analyzed via Multi-dimensional Protein Identification Technology (MudPIT) [46]–[48] with triphasic columns. Columns were individually packed using a pressure cell (New Objective, Woburn, MA). Back columns were loaded in 150 µm ID fused silica capillary tubing first with 3 cm of Luna 5 µm particle diameter strong cation exchange (SCX) resin (Phenomenex, Torrance, CA) followed by 3 cm of Aqua 5 µm C-18 reverse phase resin (Phenomenex). Proteome aliquots (40 µl) were loaded directly onto the back column via pressure cell and subsequently coupled to the front column. Front columns were pulled from 100 µm ID fused silica capillary tubing to a tip with an inside diameter of 5 µm using a P-2000 laser puller (Sutter Instruments, Novato, CA), and packed with a 17 cm long bed of Aqua 5 µm diameter C-18 reverse phase resin. This column acts as the resolving column for peptides eluted from the back column. For analysis, the combined columns were placed directly in-line with an LTQ mass spectrometer (ThermoScientific, San Jose, CA) using a Proxeon source.

Chromatographic separation was accomplished with an Ultimate HPLC system (LC Packings, a division of Dionex, San Francisco, CA) providing a flow rate of 100 µl/minute which was split prior to the resolving column such that the final flow rate through the resolving column was ∼300 nl/minute. Twelve two-dimensional (2D) chromatographic steps were done. An initial 1 hour gradient from buffer A (95% water, 5% acetonitrile, 0.1% formic acid) to buffer B (70% acetonitrile, 0.1% formic acid) bumped the peptides from the initial reverse phase column onto the strong cation exchange column. Subsequent cycles included 2 minute salt pulses with varying percentages of 500 mM ammonium acetate (10, 15, 20, 25, 30, 35, 40, 45, 50, 60%) to first elute subsets of peptides from the SCX column according to charge, followed by a 2 hour gradient from buffer A to buffer B, to further separate peptides by hydrophobicity. The final chromatographic step consisted of a 20 minute salt pulse of 100% 500 mM ammonium acetate, followed by a 2 hour A-to-B gradient.

Data collection was controlled by Xcaliber software (ThermoScientific). Data was collected in data-dependent mode with one full scan followed by 6 dependent scans, each with 2 microscans. Dynamic exclusion was employed with a repeat count of 1, repeat duration of 60 s and exclusion list size of 300 and duration of 180 s. Isolation mass width was set at 3 m/z units.

#### Data analysis

The Sp245 protein database was constructed from translated CDSs called in the draft genome sequence (http://genome.ornl.gov/microbial/abra/19sep08/). The 4B protein database was constructed from translated CDSs called in the complete genome sequence. A list of common contaminants was appended to the gene call sequences, and all coding sequences, including contaminant sequences, were reversed and appended to the forward sequences in order to serve as distractors. From the number of identifications in the reverse direction, peptide false positive (FP) rates were determined using the formula %FP = 2[No. reverse ID/(no. reverse ID+no. real ID)] [49]; FP rates ranged from 1.4%–4.3%. All MS/MS spectra were searched against the corresponding database using SEQUEST [50], specifying tryptic digestion, peptide mass tolerance of 3 m/z and a fragment ion tolerance of 0.5 m/z. Additionally, search parameters included two dynamic modifications: 1. methylation represented by a mass shift of +14 m/z on glutamate residues, and 2. deamidation followed by methylation represented by a mass shift of +15 m/z on glutamine residues. Output data files were sorted and filtered with DTASelect [51], specifying XCorr filter levels of 1.8 for peptides with a charge state of +1, 2.5 for those with charge state +2 and 3.5 for charge state +3, minimum delta CN of 0.08, semi-tryptic status and 2 peptides per protein identification. In order to determine relative abundance of a given protein in a sample, normalized spectral abundance factors (NSAF) were calculated for each individual protein k using the formula NSAF_k_ = (SpC/L)_k_/Σ (SpC/L)_n_, where SpC is the total spectral count for all peptides contributing to protein k, L is the length of protein k, and n is the total number of proteins detected in the sample [52].

### Identification of glycoside hydrolases

Bidirectional BLAST was used to identify orthologs of the putative glycoside hydrolase (GH) genes. Phyml package was used to confirm evolutionary relationships and visualize the results. Domain architectures were obtained through Pfam [53] search for each protein. Then information from CAZy [54] and recent analysis [55] was used to assign putative activities of the predicted GHs.

### Classification of chemotaxis systems in the rhizosphere

Chemotaxis proteins were identified in genomic datasets as previously described [56]. Using CheA sequences from a recent chemotaxis system classification analysis [26], alignments of the P3–P5 regions of CheA were built for each class and for the entire set of CheA sequences. Each alignment was made non-redundant so that no pair of sequences shared more than 80% sequence identity. Hidden Markov Models (HMMs) were built from each non-redundant alignment and used to create library via the HMMER3 software package (version HMMER 3.0b3) [42] and default parameters.

The rhizosphere CheA sequences from a recent study [25] were run against the CheA HMM library. Unclassified sequences (Unc) are those with top hits to the full CheA set HMM rather than a class-specific HMM. The remaining sequences were assigned to the class of the top scoring HMM.

### Cellulase assay

*Azospirillum* strains and control strains (*Dickeya dadantii* 3937 as a positive control, *A. tumefaciens* NT1 as a negative control) were cultured for 16 h in liquid AB minimal medium [57] containing 0.2% malate and 1 mg/L biotin. An aliquot of 10^7^ cells (for *Dickeya dadantii* 3937) or 2.10^7^ cells (for all other strains) was deposited on top of AB plates containing 0.1% carboxymethylcellulose instead of malate. Plates were incubated for 5 days before being stained as previously described [58].

### Pili mutant and attachment assay

A 211-bp *cpaB* (AZOBR_p460079) internal fragment was amplified by PCR with primers F6678 (GCGTGGACCTGATCCTGAC) and F6679 (GTGACCGTCTCGCTCTGAC) and subcloned into pGEM-T easy (Promega). White colonies were screened by PCR with primers F6678 and F6679 for correct insertion in pGEM-T easy, resulting in pR3.37. The insert of plasmid pR3.37 was digested with *Not*I and cloned into the *Not*I site of pKNOCK-Km [59], resulting in pR3.39 after transfer into chemically-competent cells of *E. coli* S17.1 λ*pir*. pR3.39 was introduced into *A. brasilense* Sp245 by biparental mating. Transconjugants resulting from a single recombination event of pR3.39 were selected on AB medium containing 0.2% malate, ampicillin (100 mg/mL) and kanamycin (40 mg/mL). The correct insertion of pKNOCK into *cpaB* was confirmed by PCR with primers (F6678 and F5595 TGTCCAGATAGCCCAGTAGC, located on pKNOCK) and sequencing of the PCR amplicon.

Sp245 and Sp245*cpaB* were labelled with pMP2444 [60] allowing the constitutive expression of EGFP. The strains were grown in NFB* (Nitrogen free broth containing 0.025% of LB) with appropriate antibiotics in glass tubes containing a cover-slide, under a mild lateral agitation for 6 days. After the incubation, the liquid and the cover-slide were removed from the tubes and the biofilm formed at the air/liquid interface was colored by 0.1% crystal violet. After two washings with distilled water, crystal violet was solubilized by ethanol and quantified by spectrophotometry at 570 nm. The experiment was performed twice in triplicate. In parallel, the colonization of the glass cover-slide was monitored by confocal laser scanning microscopy (510 Meta microscope; Carl Zeiss S.A.S.) equipped with an argon-krypton laser, detectors, and filter sets for green fluorescence (i.e., 488 nm for excitation and 510 to 531 nm for detection). Series of horizontal (*x-y*) optical sections with a thickness of 1 µm were taken throughout the full length of the Sp245 and Sp245*cpaB* biofilms. Three dimensional reconstructions of biofilms were performed using LSM software release 3.5 (Carl Zeiss S.A.S.).

## Supporting Information

Figure S1 Chromosomes, chromids, and plasmids in *Azospirillum* genomes. Schematic representation of chromosomes, chromids and plasmids of *A. lipoferum* 4B (A to G) and *A. brasilense* Sp245 (H to N). Radii are not to scale. The two outer rings (1 and 2) represent genes on the forward and reverse strands, respectively, colored by COG functional categories: red, Information Storage and Processing; blue, Cellular Processes and Signaling; green, Metabolism; purple, Poorly Characterized; gray, No Detected COGs. The next ring (3): tRNA (blue) and rRNA (red) genes. Ring 4 shows orthology assignment for all predicted proteins: red = present in all 3 *Azospirillum* strains (4B, Sp245, B510), orange = present in 4B and Sp245, purple = present in 4B and B510, green = present in Sp245 and B510, blue = unique to the strain. Ring 5 shows ancestry assignment for all predicted proteins: red = ancestral, blue = horizontally transferred (color intensity indicates high (dark), medium (medium) and low (light) confidence levels for prediction), gray = unassigned. Ring 6 represents the G/C skew (green = increased abundance on the direct strand; purple = increased abundance on the reverse strand) and ring 7 represents GC content.
(PDF)

Figure S2 Chemotaxis operons in *Azospirillum*. F5, F9 and ACF class chemotaxis systems were present in a common ancestor of azospirilla and other *Rhodospirillaceae* (e.g. *Rhodospirillum centenum*) [65], [66]. The F7 system was horizontally transferred to a common ancestor of *Azospirillum*. The F8 system was horizontally transferred to a common ancestor of *Azospirillum lipoferum*. The unclassified chemotaxis system (Unc) was obtained horizontally by *Azospirillum* sp. B510 only. See Tables S6 and S10 for detailed information for each system. Chemotaxis classes were assigned according to previous work by Wuichet & Zhulin [26].
(TIF)

Figure S3 Abundance of the F7 chemotaxis system in the rhizosphere. Chemotaxis systems were assigned as described in SI Materials and Methods. See Table S11 for detailed information.
(TIF)

Table S1 Typical habitats of *Rhodospirillaceae*.
(PDF)

Table S2 Identification of chromids in *Azospirillum* by house-keeping gene analysis.
(PDF)

Table S3 Identification of chromids in *Azospirillum* by GC content.
(PDF)

Table S4 ANI analysis of *Azospirillum* and rhizobial genomes.
(PDF)

Table S5 Recombination hotspots in *Azospirillum* genomes.
(PDF)

Table S6 Origin of *Azospirillum* genes.
(PDF)

Table S7 Genes that are potentially involved in adaptation of *Azospirillum* to the rhizosphere and its interaction with host plants.
(PDF)

Table S8 Divergence in the 16S rRNA gene between *Azospirillum lipoferum* 4B and other members of *Rhodospirillaceae*.
(PDF)

Table S9 Proteomic analysis of *Azospirillum*.
(PDF)

Table S10 Orthologous chemotaxis operons in *Azospirillum* and *Rhodospirillum centenum*.
(PDF)

Table S11 Classification of chemotaxis systems in rhizosphere.
(PDF)

Table S12 Putative complex carbohydrate-degrading enzymes in three *Azospirillum* species in comparison with a soil cellulolytic bacterium *Thermobifida fusca*.
(PDF)

## References

[1] Mojzsis SJ, Arrhenius G, McKeegan KD, Harrison TM, Nutman AP (1996). Evidence for life on Earth before 3,800 million years ago.. Nature.

[2] Watanabe Y, Martini JE, Ohmoto H (2000). Geochemical evidence for terrestrial ecosystems 2.6 billion years ago.. Nature.

[3] Battistuzzi FU, Hedges SB (2009). A major clade of prokaryotes with ancient adaptations to life on land.. Mol Biol Evol.

[4] Kettler GC, Martiny AC, Huang K, Zucker J, Coleman ML (2007). Patterns and implications of gene gain and loss in the evolution of *Prochlorococcus*.. PLoS Genet.

[5] Okon Y, Labandera-Gonzalez CA (1994). Agronomic applications of *Azospirillum*: An evaluation of 20 years worldwide field inoculation.. Soil Biol Biochem.

[6] Steenhoudt O, Vanderleyden J (2000). *Azospirillum*, a free-living nitrogen-fixing bacterium closely associated with grasses: genetic, biochemical and ecological aspects.. FEMS Microbiol Rev.

[7] Kaneko T, Minamisawa K, Isawa T, Nakatsukasa H, Mitsui H (2010). Complete genomic structure of the cultivated rice endophyte *Azospirillum* sp. B510.. DNA Res.

[8] Martin-Didonet CC, Chubatsu LS, Souza EM, Kleina M, Rego FG (2000). Genome structure of the genus *Azospirillum*.. J Bacteriol.

[9] Harrison PW, Lower RP, Kim NK, Young JP (2010). Introducing the bacterial ‘chromid’: not a chromosome, not a plasmid.. Trends Microbiol.

[10] Gonzalez V, Santamaria RI, Bustos P, Hernandez-Gonzalez I, Medrano-Soto A (2006). The partitioned *Rhizobium etli* genome: genetic and metabolic redundancy in seven interacting replicons.. Proc Natl Acad Sci U S A.

[11] Vial L, Lavire C, Mavingui P, Blaha D, Haurat J (2006). Phase variation and genomic architecture changes in *Azospirillum*.. J Bacteriol.

[12] Koonin EV, Makarova KS, Aravind L (2001). Horizontal gene transfer in prokaryotes: quantification and classification.. Annu Rev Microbiol.

[13] Tatusov RL, Natale DA, Garkavtsev IV, Tatusova TA, Shankavaram UT (2001). The COG database: new developments in phylogenetic classification of proteins from complete genomes.. Nucleic Acids Res.

[14] Dennis PG, Miller AJ, Hirsch PR (2010). Are root exudates more important than other sources of rhizodeposits in structuring rhizosphere bacterial communities?. FEMS Microbiol Ecol.

[15] Boyer M, Haurat J, Samain S, Segurens B, Gavory F (2008). Bacteriophage prevalence in the genus *Azospirillum* and analysis of the first genome sequence of an *Azospirillum brasilense* integrative phage.. Appl Environ Microbiol.

[16] Giraud E, Moulin L, Vallenet D, Barbe V, Cytryn E (2007). Legumes symbioses: absence of Nod genes in photosynthetic bradyrhizobia.. Science.

[17] Kuo CH, Ochman H (2009). Inferring clocks when lacking rocks: the variable rates of molecular evolution in bacteria.. Biol Direct.

[18] Kenrick P, Crane PR (1997). The origin and early evolution of plants on land.. Nature.

[19] Raven JA, Edwards D (2001). Roots: evolutionary origins and biogeochemical significance.. J Exp Bot.

[20] Prasad V, Stromberg CA, Alimohammadian H, Sahni A (2005). Dinosaur coprolites and the early evolution of grasses and grazers.. Science.

[21] Xie Z, Ulrich LE, Zhulin IB, Alexandre G (2010). PAS domain containing chemoreceptor couples dynamic changes in metabolism with chemotaxis.. Proc Natl Acad Sci U S A.

[22] Jiang ZY, Bauer CE (1997). Analysis of a chemotaxis operon from *Rhodospirillum centenum*.. J Bacteriol.

[23] Bible AN, Stephens BB, Ortega DR, Xie Z, Alexandre G (2008). Function of a chemotaxis-like signal transduction pathway in modulating motility, cell clumping, and cell length in the alphaproteobacterium *Azospirillum brasilense*.. J Bacteriol.

[24] Ulrich LE, Zhulin IB (2010). The MiST2 database: a comprehensive genomics resource on microbial signal transduction.. Nucleic Acids Res.

[25] Buchan A, Crombie B, Alexandre GM (2010). Temporal dynamics and genetic diversity of chemotactic-competent microbial populations in the rhizosphere.. Environ Microbiol.

[26] Wuichet K, Zhulin IB (2010). Origins and diversification of a complex signal transduction system in prokaryotes.. Sci Signal.

[27] Assmus B, Hutzler P, Kirchhof G, Amann R, Lawrence JR (1995). In situ localization of *Azospirillum brasilense* in the rhizosphere of wheat with fluorescently labeled, rRNA-targeted oligonucleotide probes and scanning confocal laser microscopy.. Appl Environ Microbiol.

[28] Pedrosa FO, Monteiro RA, Wassem R, Cruz LM, Ayub RA (2011). Genome of *Herbaspirillum seropedicae* strain SmR1, a specialized diazotrophic endophyte of tropical grasses.. PLoS Genet.

[29] Dorr J, Hurek T, Reinhold-Hurek B (1998). Type IV pili are involved in plant-microbe and fungus-microbe interactions.. Mol Microbiol.

[30] Ramey BE, Koutsoudis M, von Bodman SB, Fuqua C (2004). Biofilm formation in plant-microbe associations.. Curr Opin Microbiol.

[31] Tomich M, Planet PJ, Figurski DH (2007). The tad locus: postcards from the widespread colonization island.. Nat Rev Microbiol.

[32] Handelsman J, Tiedje J, Alvarez-Cohen L, Ashburner M, Cann IKO (2007). The New Science of Metagenomics: Revealing the Secrets of Our Microbial Planet..

[33] Caro-Quintero A, Deng J, Auchtung J, Brettar I, Hofle MG (2011). Unprecedented levels of horizontal gene transfer among spatially co-occurring *Shewanella* bacteria from the Baltic Sea.. ISME J.

[34] Bartolome B, Jubete Y, Martinez E, de la Cruz F (1991). Construction and properties of a family of pACYC184-derived cloning vectors compatible with pBR322 and its derivatives.. Gene.

[35] Bocs S, Cruveiller S, Vallenet D, Nuel G, Medigue C (2003). AMIGene: Annotation of MIcrobial Genes.. Nucleic Acids Res.

[36] Vallenet D, Labarre L, Rouy Z, Barbe V, Bocs S (2006). MaGe: a microbial genome annotation system supported by synteny results.. Nucleic Acids Res.

[37] Siguier P, Perochon J, Lestrade L, Mahillon J, Chandler M (2006). ISfinder: the reference centre for bacterial insertion sequences.. Nucleic Acids Res.

[38] Altschul SF, Madden TL, Schaffer AA, Zhang J, Zhang Z (1997). Gapped BLAST and PSI-BLAST: a new generation of protein database search programs.. Nucleic Acids Res.

[39] Katoh K, Kuma K, Toh H, Miyata T (2005). MAFFT version 5: improvement in accuracy of multiple sequence alignment.. Nucleic Acids Res.

[40] Guindon S, Dufayard JF, Lefort V, Anisimova M, Hordijk W (2010). New algorithms and methods to estimate maximum-likelihood phylogenies: assessing the performance of PhyML 3.0.. Syst Biol.

[41] Cole JR, Wang Q, Cardenas E, Fish J, Chai B (2009). The Ribosomal Database Project: improved alignments and new tools for rRNA analysis.. Nucleic Acids Res.

[42] Eddy SR (1998). Profile hidden Markov models.. Bioinformatics.

[43] Talavera G, Castresana J (2007). Improvement of phylogenies after removing divergent and ambiguously aligned blocks from protein sequence alignments.. Syst Biol.

[44] Hauwaerts D, Alexandre G, Das SK, Vanderleyden J, Zhulin IB (2002). A major chemotaxis gene cluster in *Azospirillum brasilense* and relationships between chemotaxis operons in alpha-proteobacteria.. FEMS Microbiol Lett.

[45] Thompson MR, Chourey K, Froelich JM, Erickson BK, VerBerkmoes NC (2008). Experimental approach for deep proteome measurements from small-scale microbial biomass samples.. Anal Chem.

[46] McDonald WH, Ohi R, Miyamoto DT, Mitchison TJ, Yates JR (2002). Comparison of three directly coupled HPLC MS/MS strategies for identification of proteins from complex mixtures: single-dimension LC-MS/MS, 2-phase MudPIT, and 3-phase MudPIT.. Int J Mass Spectrom.

[47] Washburn MP, Wolters D, Yates JR (2001). Large-scale analysis of the yeast proteome by multidimensional protein identification technology.. Nat Biotechnol.

[48] Wolters DA, Washburn MP, Yates JR (2001). An automated multidimensional protein identification technology for shotgun proteomics.. Anal Chem.

[49] Peng JM, Elias JE, Thoreen CC, Licklider LJ, Gygi SP (2003). Evaluation of multidimensional chromatography coupled with tandem mass spectrometry (LC/LC-MS/MS) for large-scale protein analysis: The yeast proteome.. J Proteome Res.

[50] Eng JK, Mccormack AL, Yates JR (1994). An approach to correlate tandem mass spectral data of peptides with amino acid sequences in a protein database.. J Am Soc Mass Spectr.

[51] Tabb DL, McDonald WH, Yates JR (2002). DTASelect and contrast: Tools for assembling and comparing protein identifications from shotgun proteomics.. J Proteome Res.

[52] Washburn MP, Florens L, Carozza MJ, Swanson SK, Fournier M (2006). Analyzing chromatin remodeling complexes using shotgun proteomics and normalized spectral abundance factors.. Methods.

[53] Finn RD, Mistry J, Tate J, Coggill P, Heger A (2010). The Pfam protein families database.. Nucleic Acids Res.

[54] Cantarel BL, Coutinho PM, Rancurel C, Bernard T, Lombard V (2009). The Carbohydrate-Active EnZymes database (CAZy): an expert resource for Glycogenomics.. Nucleic Acids Res.

[55] Sukharnikov LO, Cantwell BJ, Podar M, Zhulin IB (2011). Cellulases: ambiguous nonhomologous enzymes in a genomic perspective.. Trends Biotechnol.

[56] Wuichet K, Alexander RP, Zhulin IB (2007). Comparative genomic and protein sequence analyses of a complex system controlling bacterial chemotaxis.. Methods Enzymol.

[57] Shaw PD, Ping G, Daly SL, Cha C, Cronan JE (1997). Detecting and characterizing N-acyl-homoserine lactone signal molecules by thin-layer chromatography.. Proc Natl Acad Sci U S A.

[58] Park SR, Cho SJ, Yun HD (2000). Cloning and sequencing of pel gene responsible for CMCase activity from *Erwinia chrysanthemi* PY35.. Biosci Biotechnol Biochem.

[59] Alexeyev MF (1999). The pKNOCK series of broad-host-range mobilizable suicide vectors for gene knockout and targeted DNA insertion into the chromosome of gram-negative bacteria.. Biotechniques.

[60] Bloemberg GV, Wijfjes AH, Lamers GE, Stuurman N, Lugtenberg BJ (2000). Simultaneous imaging of *Pseudomonas fluorescens* WCS365 populations expressing three different autofluorescent proteins in the rhizosphere: new perspectives for studying microbial communities.. Mol Plant Microbe Interact.

[61] Lykidis A, Mavromatis K, Ivanova N, Anderson I, Land M (2007). Genome sequence and analysis of the soil cellulolytic actinomycete *Thermobifida fusca* YX.. J Bacteriol.

[62] Qi M, Jun HS, Forsberg CW (2007). Characterization and synergistic interactions of *Fibrobacter succinogenes* glycoside hydrolases.. Appl Environ Microbiol.

[63] Fierobe HP, Bagnara-Tardif C, Gaudin C, Guerlesquin F, Sauve P (1993). Purification and characterization of endoglucanase C from *Clostridium cellulolyticum*. Catalytic comparison with endoglucanase A.. European journal of biochemistry/FEBS.

[64] Ogura J, Toyoda A, Kurosawa T, Chong AL, Chohnan S (2006). Purification, characterization, and gene analysis of cellulase (Cel8A) from *Lysobacter* sp. IB-9374.. Biosci Biotechnol Biochem.

[65] Berleman JE, Bauer CE (2005). Involvement of a Che-like signal transduction cascade in regulating cyst cell development in *Rhodospirillum centenum*.. Mol Microbiol.

[66] Berleman JE, Bauer CE (2005). A che-like signal transduction cascade involved in controlling flagella biosynthesis in *Rhodospirillum centenum*.. Mol Microbiol.

