# Supplementary material for: Azospirillum Genomes Reveal Transition of Bacteria from Aquatic to Terrestrial Environments
Source: PLoS Genet. 2011 Dec 22;7(12):e1002430. doi: 10.1371/journal.pgen.1002430 (PMC3245306; doi:10.1371/journal.pgen.1002430)
Supplement: Figure S1 — Chromosomes, chromids, and plasmids in Azospirillum genomes. Schematic representation of chromosomes, chromids and plasmids of A. lipoferum 4B (A to G) and A. brasilense Sp245 (H to N). Radii are not to scale. The two outer rings (1 and 2) represent genes on the forward and reverse strands, respectively, colored by COG functional categories: red, Information Storage and Processing; blue, Cellular Processes and Signaling; green, Metabolism; purple, Poorly Characterized; gray, No Detected COGs. The next ring (3): tRNA (blue) and rRNA (red) genes. Ring 4 shows orthology assignment for all predicted proteins: red = present in all 3 Azospirillum strains (4B, Sp245, B510), orange = present in 4B and Sp245, purple = present in 4B and B510, green = present in Sp245 and B510, blue = unique to the strain. Ring 5 shows ancestry assignment for all predicted proteins: red = ancestral, blue = horizontally transferred (color intensity indicates high (dark), medium (medium) and low (light) confidence levels for prediction), gray = unassigned. Ring 6 represents the G/C skew (green = increased abundance on the direct strand; purple = increased abundance on the reverse strand) and ring 7 represents GC content. (PDF) [file pgen.1002430.s001.pdf]

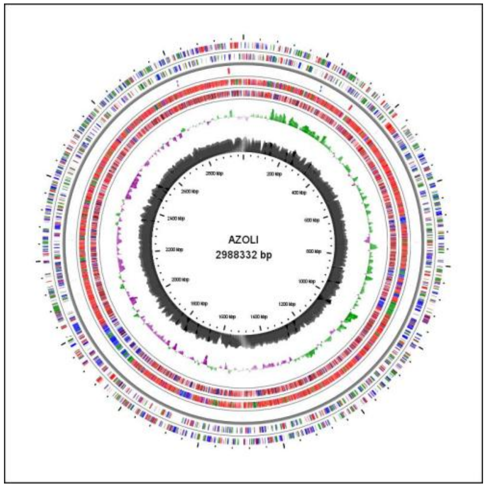

A *A. lipoferum* 4B – chromosome – AZOLI

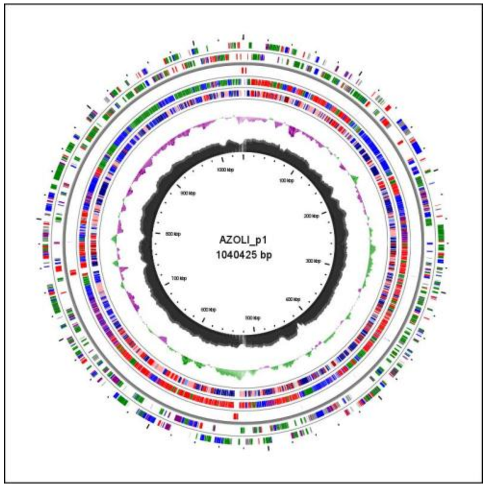

B *A. lipoferum* 4B – chromid 1 – AZOLI_p1

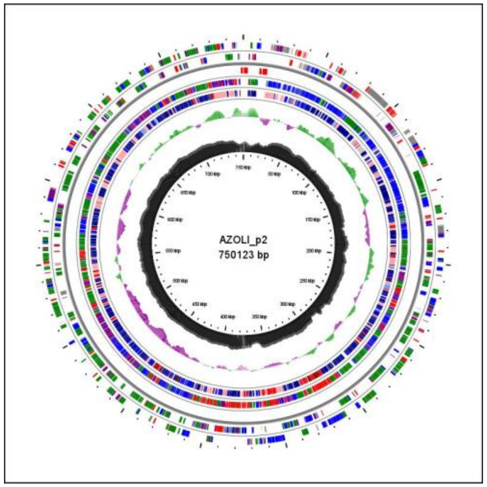

C *A. lipoferum* 4B – chromid 2 – AZOLI_p2

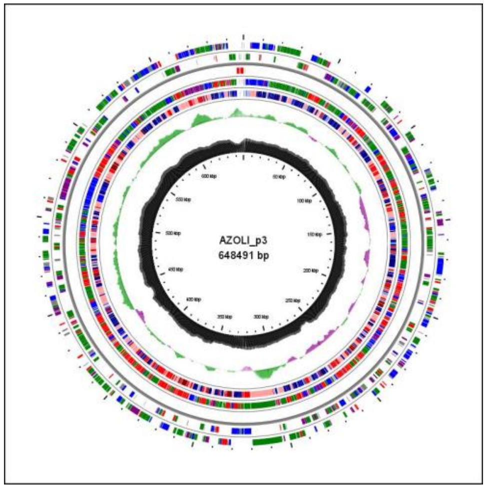

D *A. lipoferum* 4B – chromid 3 – AZOLI_p3

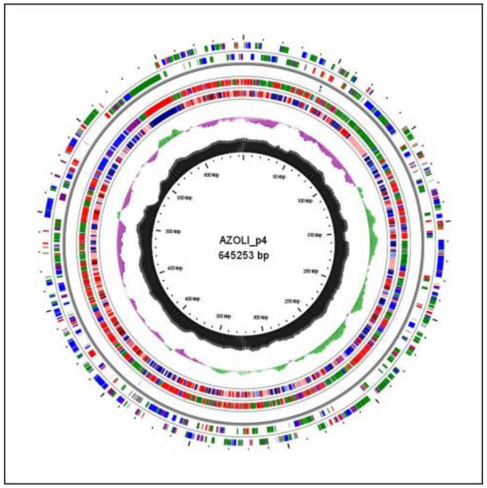

E *A. lipoferum* 4B – chromid 4 – AZOLI_p4

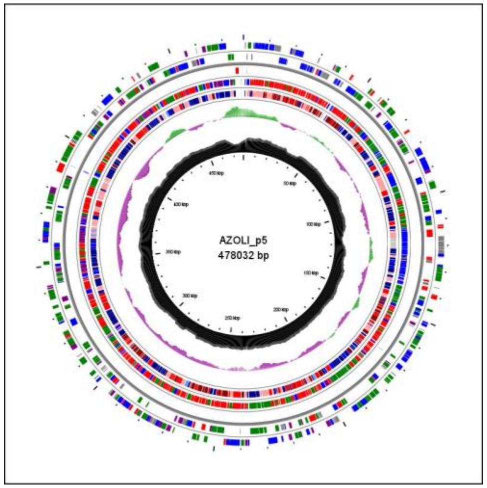

F *A. lipoferum* 4B – chromid 5 – AZOLI_p5

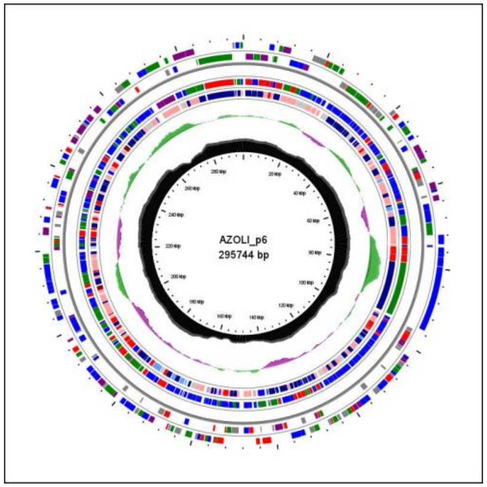

G *A. lipoferum* 4B – plasmid 1 – AZOLI_p6

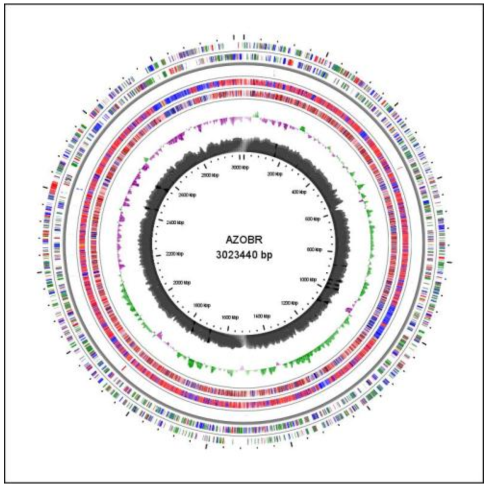

H. *A. brasilense* Sp245 – chromosome –AZOBR

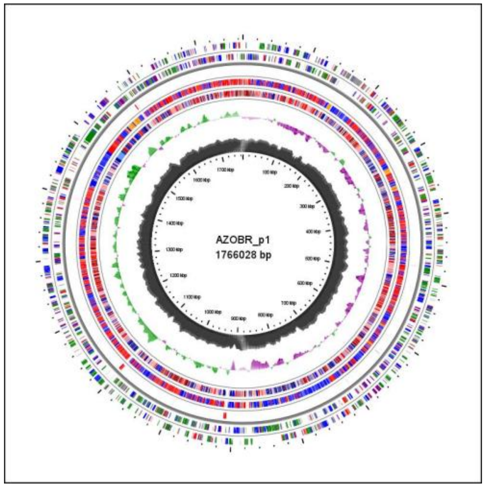

I *A. brasilense* Sp245 – chromid 1 –AZOBR_p1

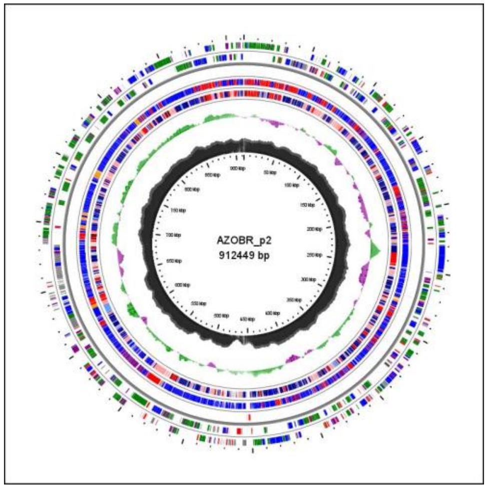

J *A. brasilense* Sp245 – chromid 2 –AZOBR_p2

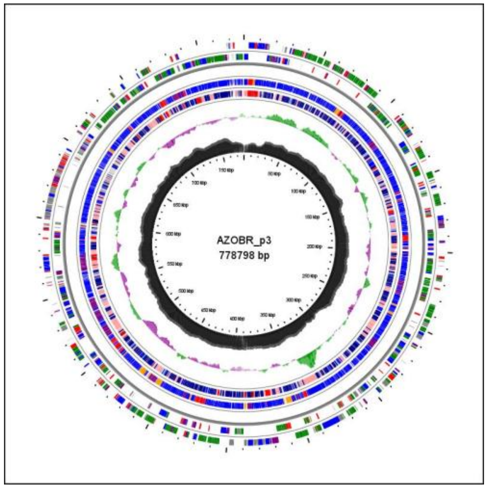

K *A. brasilense* Sp245 – plasmid 1 –AZOBR_p3

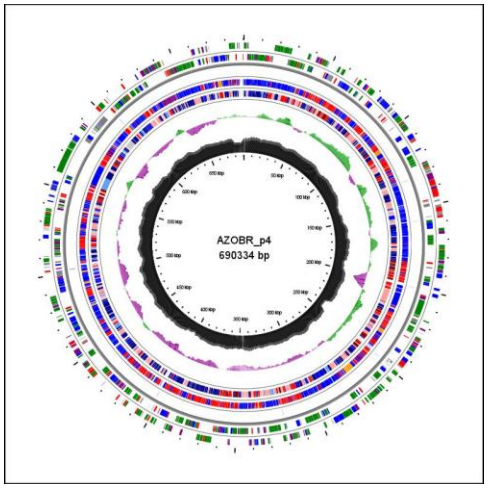

L *A. brasilense* Sp245 – chromid 3 –AZOBR_p4

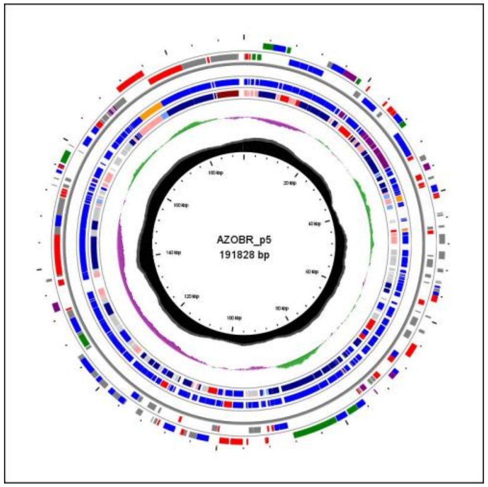

M *A. brasilense* Sp245 – plasmid 2 –AZOBR_p5

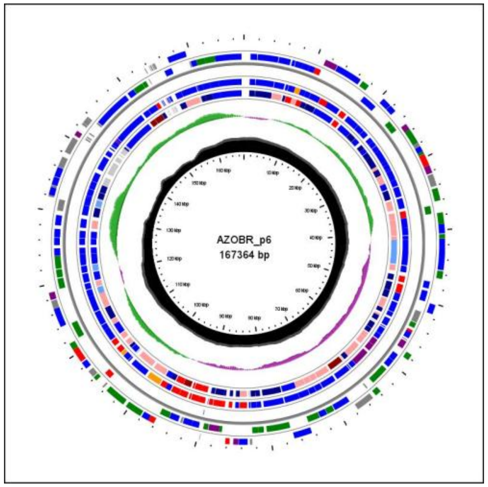

N *A. brasilense* Sp245 – plasmid 3 –AZOBR_p6
