# Supplementary figures and images for: Azospirillum Genomes Reveal Transition of Bacteria from Aquatic to Terrestrial Environments
Source: PLoS Genet. 2011 Dec 22;7(12):e1002430. doi: 10.1371/journal.pgen.1002430 (PMC3245306; doi:10.1371/journal.pgen.1002430)

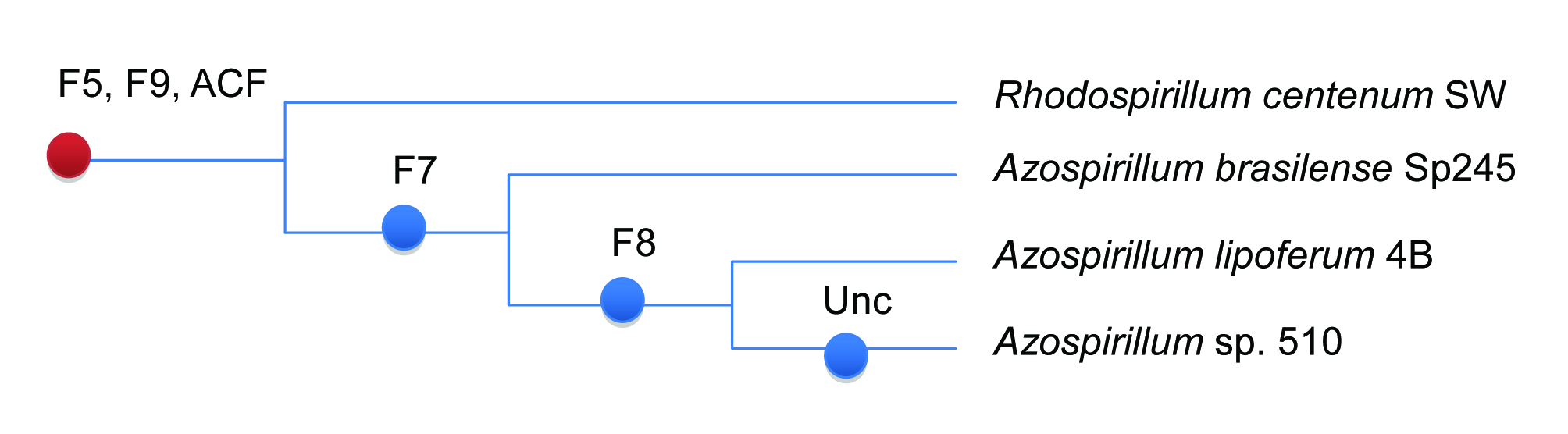

Supplement: Figure S2 — Chemotaxis operons in Azospirillum. F5, F9 and ACF class chemotaxis systems were present in a common ancestor of azospirilla and other Rhodospirillaceae (e.g. Rhodospirillum centenum) [65], [66]. The F7 system was horizontally transferred to a common ancestor of Azospirillum. The F8 system was horizontally transferred to a common ancestor of Azospirillum lipoferum. The unclassified chemotaxis system (Unc) was obtained horizontally by Azospirillum sp. B510 only. See Tables S6 and S10 for detailed information for each system. Chemotaxis classes were assigned according to previous work by Wuichet & Zhulin [26]. (TIF) [file pgen.1002430.s002.tif]

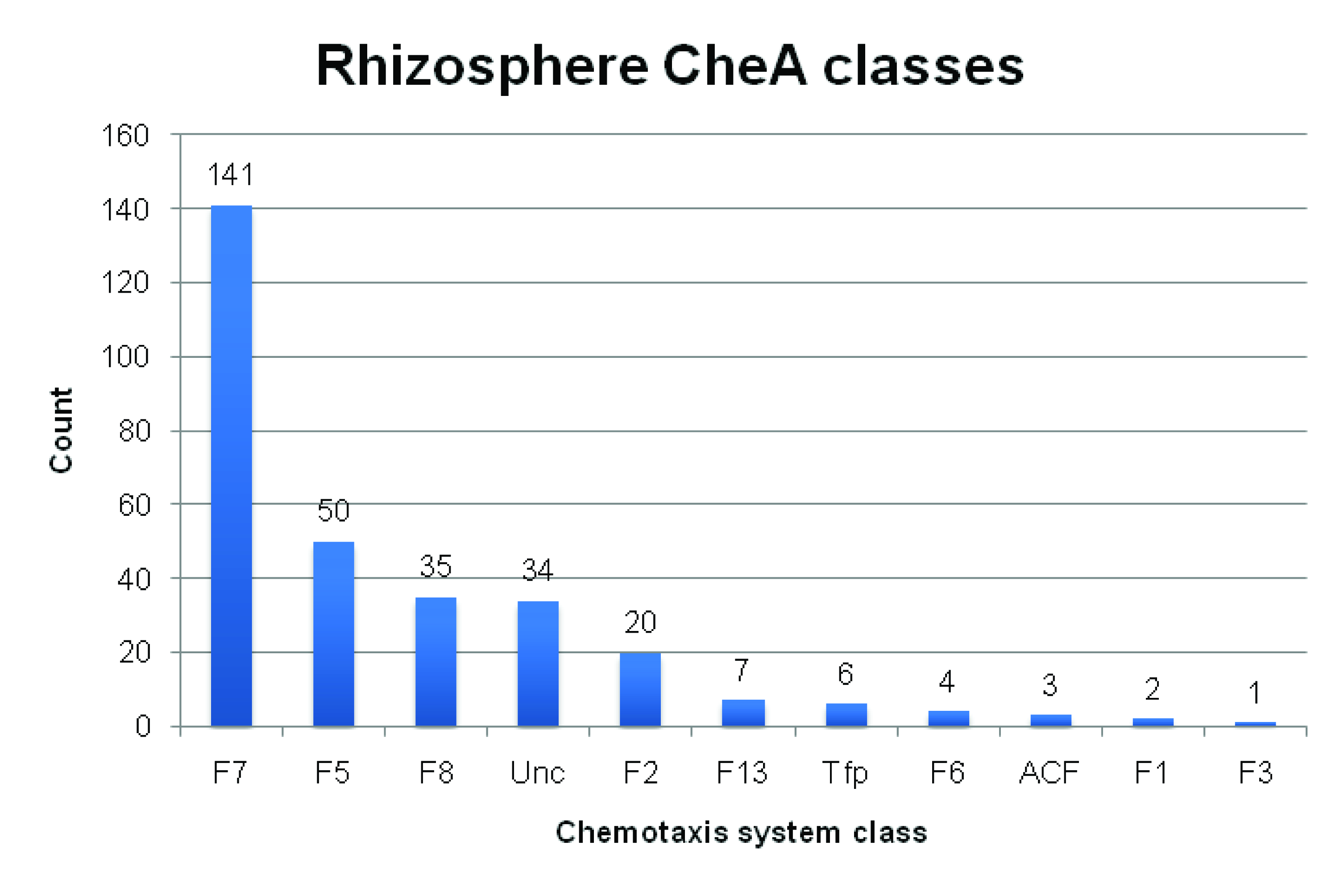

Supplement: Figure S3 — Abundance of the F7 chemotaxis system in the rhizosphere. Chemotaxis systems were assigned as described in SI Materials and Methods. See Table S11 for detailed information. (TIF) [file pgen.1002430.s003.tif]
