# Supplementary material for: Azospirillum Genomes Reveal Transition of Bacteria from Aquatic to Terrestrial Environments
Source: PLoS Genet. 2011 Dec 22;7(12):e1002430. doi: 10.1371/journal.pgen.1002430 (PMC3245306; doi:10.1371/journal.pgen.1002430)
Supplement: Table S1 — Typical habitats of Rhodospirillaceae. (PDF) [file pgen.1002430.s004.pdf]

Table S1. Habitats of *Rhodospirillaceae*

Species	Habitat	Reference
Azospirillum amazonense	roots of maize, sorghum, rice and wheat plants, as well as forage grasses grown around Brazil	[1]
Azospirillum brasilense Sp245	colonizing several plants including cereals, forage grasses, vegetables, legumes, and banana plants	[2]
Azospirillum canadense	corn rhizosphere	[3]
Azospirillum doebereinerae	root of *Miscanthus sinensis* cv. "Giganteus" and *Miscanthus sacchariflorus* and also in the rhizosphere soil of these plants grown in Freising, Germany	[4]
Azospirillum halopraeferens	root surface of Kallar grass (*Leptochloa fusca*) grown in saline-sodic soils in Punjab, Pakistan	[5]
Azospirillum irakense	rhizosphere soil and roots of rice plants grown in the region of Diwaniyah in Iraq	[6]
Azospirillum largimobile	fresh lake water in Australia	[7, 8]
Azospirillum lipoferum 4B	rice field of Camargue (South of France)	[9]
Azospirillum melinis	isolated from molasses grass (*Melinis minutiflora* Beauv.)	[10]
Azospirillum oryzae	roots of the rice plant *Oryza sativa*	[11]
Azospirillum palatum	forest soil in Zhejiang province, China	[12]
Azospirillum picis	discarded road tar	[13]
Azospirillum rugosum	oil-contaminated soil sample	[14]
Azospirillum sp. B510	endophytic bacterium isolated from stems of rice plants	[15]
Azospirillum zeae	corn rhizosphere	[16]
Caenispirillum bisanense	sludge from the wastewater treatment plant	[17]
Dechlorospirillum sp.	sewage treatment plant	[18]
Defluviicoccus vanus	wastewater	[19]
Fodinicurvata fenggangensis	salt mine in Yunnan, south-west China	[20]
Fodinicurvata sediminis	salt mine in Yunnan, south-west China	[20]
Inquilinus ginsengisoli	soil	[21]
Inquilinus limosus	human respiratory tract	[22]
Insolitispirillum peregrinum	primary oxidation pond	[23]
Magnetospirillum bellicus	bioelectrical reactor (BER) inoculated from creek water	[24]
Magnetospirillum gryphiswaldense MSR-1	freshwater sediment	[25]
Magnetospirillum magneticum AMB-1	pond water in Tokyo Japan	[26]
Magnetospirillum magnetotacticum MS-1	microaerobic zones from freshwater sediments	[27]
Marispirillum indicum	deep sea	[28]
Nisaea denitrificans	mediterranean sea	[29]
Nisaea nitritireducens	mediterranean sea	[29]
Nisaea sp. BAL199	3m depth of Baltic proper	[30]
Novispirillum itersonii	pond water	[23]
Oceanibaculum indicum	deep sea Indian Ocean	[31]
Oceanibaculum pacificum	hydrothermal sediment of the south-west Pacific ocean	[32]
Pelagibius litoralis	coastal seawater Korea	[33]
Phaeospirillum chandramohanii	freshwater habitat	[34]

Phaeospirillum cystidoformans	freshwater habitat	[35]
Phaeospirillum fulvum	stagnant and anoxic freshwater habitats that are exposed to the light	[35]
Phaeospirillum molischianum	stagnant and anoxic freshwater habitats that are exposed to the light	[35]
Phaeovibrio sulfidiphilus	brackish water	[36]
Rhodocista pekingensis	wastewater	[37]
Rhodocista xerospirillum	lake water	[38]
Rhodospira trueperi	salt marsh microbial mat	[39]
Rhodospirillum centenum SW	hot spring (hot spring mud) Wyoming, fresh water	[40]
Rhodospirillum photometricum	freshwater pond	[41]
Rhodospirillum rubrum ATCC 11170	aquatic environments such as lakes, streams, and standing water	[42]
Rhodospirillum sulfurexigens	freshwater reservoir	[43]
Rhodovibrio salinarum	halophylic, sea water	[35]
Rhodovibrio sodomensis	water/sediment of the Dead Sea	[35]
Roseospira goensis	seawater	[44]
Roseospira marina	sediments, saline springs, microbial mats	[45]
Roseospira mediosalina	sediments, saline springs, microbial mats	[45]
Roseospira navarrensis	sediments, saline springs, microbial mats	[45]
Roseospira thiosulfatophila	microbial mats in French Polynesia	[45]
Roseospira visakhapatnamensis	seawater	[44]
Skermanella aerolata	air	[46]
Skermanella parooensis	water from the Paroo Channel in southwest Queensland	[47]
Skermanella xinjiangensis	desert soil	[48]
Telmatospirillum siberiense	groundwater (mesotrophic fen)	[49]
Thalassobaculum litoreum	coastal seawater	[50]
Thalassobaculum salexigens	Mediterranean Sea	[51]
Thalassospira lucentensis	Mediterranean Sea	[52, 53]
Thalassospira profundimaris	deep sea	[54]
Thalassospira tepidiphila	petroleum-contaminated seawater during a bioremediation experiment	[55]
Thalassospira xiamenensis	surface water of a waste-oil pool	[54]
Thalassospira xianhensis	oil-degrading marine bacterium from oil-polluted soil	[56]
Tistrella mobilis	wastewater, deep sea	[53, 57]

Table of all currently described members of the family *Rhodospirillaceae* and the habitat of their initial isolation as of January 2011. Species names in bold refer to sequenced strains, both complete and draft genomes.

References:

1. Magalhaes FM, Baldani JI, Souto SM, Kuykendall JR, Doebereiner J (1983) A new acid-tolerant Azospirillum species. An. Acad. Bras. Cienc. 55: 417-430.
2. Tarrand JJ, Krieg NR, Dobereiner J (1978) A taxonomic study of the *Spirillum lipoferum* group, with descriptions of a new genus, *Azospirillum* gen. nov. and two species, *Azospirillum lipoferum* (Beijerinck) comb. nov. and *Azospirillum brasilense* sp. nov. Can J Microbiol 24: 967-980.
3. Mehnaz S, Weselowski B, Lazarovits G (2007) *Azospirillum canadense* sp. nov., a nitrogen-fixing bacterium isolated from corn rhizosphere. Int J Syst Evol Microbiol 57: 620-624.
4. Eckert B, Weber OB, Kirchhof G, Halbritter A, Stoffels M, et al. (2001) *Azospirillum doebereinerae* sp. nov., a nitrogen-fixing bacterium associated with the C4-grass *Miscanthus*. Int J Syst Evol Microbiol 51: 17-26.
5. Hurek T, Reinhold B, Fendrik I, Niemann EG (1987) Root-zone-specific oxygen tolerance of *Azospirillum* spp. and diazotrophic rods closely associated with Kallar grass. Appl Environ Microbiol 53: 163-169.
6. Khammas KM, Ageron E, Grimont PA, Kaiser P (1989) *Azospirillum irakense* sp. nov., a nitrogen-fixing bacterium associated with rice roots and rhizosphere soil. Res Microbiol 140: 679-693.
7. Dekhil SB, Cahill M, Stackebrandt E, Sly LI (1997) Transfer of Conglomeromonas largomobilis subsp largomobilis to the genus Azospirillum as Azopirillum largomobile comb nov, and elevation of Conglomeromonas largomobilis subsp parooensis to the new type species of Conglomeromonas, Conglomeromonas parooensis sp nov. Systematic and Applied Microbiology 20: 72-77.
8. Skerman VBD, Sly LI, Williamson ML (1983) Conglomeromonas-Largomobilis Gen-Nov, Sp-Nov, a Sodium-Sensitive, Mixed-Flagellated Organism from Fresh Waters. International Journal of Systematic Bacteriology 33: 300-308.
9. Ladha JK, Barraquio WL, Watanabe I (1982) Immunological techniques to identify *Azospirillum* associated with wetland rice. Can J Microbiol 28: 478-485.
10. Peng G, Wang H, Zhang G, Hou W, Liu Y, et al. (2006) *Azospirillum melinis* sp. nov., a group of diazotrophs isolated from tropical molasses grass. Int J Syst Evol Microbiol 56: 1263-1271.
11. Xie CH, Yokota A (2005) *Azospirillum oryzae* sp. nov., a nitrogen-fixing bacterium isolated from the roots of the rice plant *Oryza sativa*. Int J Syst Evol Microbiol 55: 1435-1438.
12. Zhou Y, Wei W, Wang X, Xu L, Lai R (2009) *Azospirillum palatum* sp. nov., isolated from forest soil in Zhejiang province, China. J Gen Appl Microbiol 55: 1-7.
13. Lin SY, Young CC, Hupfer H, Siering C, Arun AB, et al. (2009) *Azospirillum picis* sp. nov., isolated from discarded tar. Int J Syst Evol Microbiol 59: 761-765.
14. Young CC, Hupfer H, Siering C, Ho MJ, Arun AB, et al. (2008) *Azospirillum rugosum* sp. nov., isolated from oil-contaminated soil. Int J Syst Evol Microbiol 58: 959-963.
15. Kaneko T, Minamisawa K, Isawa T, Nakatsukasa H, Mitsui H, et al. (2010) Complete genomic structure of the cultivated rice endophyte *Azospirillum* sp. B510. DNA Res 17: 37-50.
16. Mehnaz S, Weselowski B, Lazarovits G (2007) *Azospirillum zeae* sp. nov., a diazotrophic bacterium isolated from rhizosphere soil of *Zea mays*. Int J Syst Evol Microbiol 57: 2805-2809.
17. Yoon JH, Kang SJ, Park S, Oh TK (2007) *Caenispirillum bisanense* gen. nov., sp. nov., isolated from sludge of a dye works. Int J Syst Evol Microbiol 57: 1217-1221.
18. Bardiya N, Bae JH (2008) Isolation and characterization of *Dechlorospirillum* anomalous strain JB116 from a sewage treatment plant. Microbiol Res 163: 182-191.
19. Maszenan AM, Seviour RJ, Patel BK, Janssen PH, Wanner J (2005) *Defluvicoccus vanus* gen. nov., sp. nov., a novel Gram-negative coccus/coccobacillus in the '*Alphaproteobacteria*' from activated sludge. Int J Syst Evol Microbiol 55: 2105-2111.

20. Wang YX, Liu JH, Zhang XX, Chen YG, Wang ZG, et al. (2009) *Fodinicurvata sediminis* gen. nov., sp. nov. and *Fodinicurvata fenggangensis* sp. nov., poly-beta-hydroxybutyrate-producing bacteria in the family *Rhodospirillaceae*. Int J Syst Evol Microbiol 59: 2575-2581.
21. Jung HM, Lee JS, Bae HM, Yi TH, Kim SY, et al. (2011) *Inquilinus ginsengisoli* sp. nov., isolated from soil of a ginseng field. Int J Syst Evol Microbiol 61: 201-204.
22. Wellinghausen N, Essig A, Sommerburg O (2005) *Inquilinus limosus* in patients with cystic fibrosis, Germany. Emerg Infect Dis 11: 457-459.
23. Yoon JH, Kang SJ, Park S, Lee SY, Oh TK (2007) Reclassification of *Aquaspirillum itersonii* and *Aquaspirillum peregrinum* as *Novispirillum itersonii* gen. nov., comb. nov. and *Insolitispirillum peregrinum* gen. nov., comb. nov. Int J Syst Evol Microbiol 57: 2830-2835.
24. Thrash JC, Ahmadi S, Torok T, Coates JD (2010) *Magnetospirillum bellicus* sp. nov., a novel dissimilatory perchlorate-reducing alphaproteobacterium isolated from a bioelectrical reactor. Appl Environ Microbiol 76: 4730-4737.
25. Geelhoed JS, Sorokin DY, Epping E, Tourova TP, Banciu HL, et al. (2009) Microbial sulfide oxidation in the oxic-anoxic transition zone of freshwater sediment: involvement of lithoautotrophic *Magnetospirillum* strain J10. FEMS Microbiol Ecol 70: 54-65.
26. Matsunaga T, Okamura Y, Fukuda Y, Wahyudi AT, Murase Y, et al. (2005) Complete genome sequence of the facultative anaerobic magnetotactic bacterium *Magnetospirillum* sp. strain AMB-1. DNA Res 12: 157-166.
27. Schleifer KH, Schuler D, Spring S, Weizenegger M, Amann R, et al. (1991) The Genus Magnetospirillum Gen-Nov - Description of Magnetospirillum-Gryphiswaldense Sp-Nov and Transfer of Aquaspirillum-Magnetotacticum to Magnetospirillum-Magnetotacticum Comb-Nov. Systematic and Applied Microbiology 14: 379-385.
28. Lai Q, Yuan J, Gu L, Shao Z (2009) *Marispirillum indicum* gen. nov., sp. nov., isolated from a deep-sea environment. Int J Syst Evol Microbiol 59: 1278-1281.
29. Urios L, Michotey V, Intertaglia L, Lesongeur F, Lebaron P (2008) *Nisaea denitrificans* gen. nov., sp. nov. and *Nisaea nitritireducens* sp. nov., two novel members of the class *Alphaproteobacteria* from the Mediterranean Sea. Int J Syst Evol Microbiol 58: 2336-2341.
30. Riemann L, Leitet C, Pommier T, Simu K, Holmfeldt K, et al. (2008) The native bacterioplankton community in the central Baltic Sea is influenced by freshwater bacterial species. Appl Environ Microbiol 74: 503-515.
31. Lai Q, Yuan J, Wu C, Shao Z (2009) *Oceanibaculum indicum* gen. nov., sp. nov., isolated from deep seawater of the Indian Ocean. Int J Syst Evol Microbiol 59: 1733-1737.
32. Dong C, Lai Q, Chen L, Sun F, Shao Z, et al. (2010) *Oceanibaculum pacificum* sp. nov., isolated from hydrothermal field sediment of the south-west Pacific Ocean. Int J Syst Evol Microbiol 60: 219-222.
33. Choi DH, Hwang CY, Cho BC (2009) *Pelagibius litoralis* gen. nov., sp. nov., a marine bacterium in the family *Rhodospirillaceae* isolated from coastal seawater. Int J Syst Evol Microbiol 59: 818-823.
34. Anil Kumar P, Srinivas TN, Takaichi S, Maoka T, Sasikala C, et al. (2009) *Phaeospirillum chandramohanii* sp. nov., a phototrophic alphaproteobacterium with carotenoid glycosides. Int J Syst Evol Microbiol 59: 2089-2093.
35. Imhoff JF, Petri R, Suling J (1998) Reclassification of species of the spiral-shaped phototrophic purple non-sulfur bacteria of the alpha-Proteobacteria: description of the new genera Phaeospirillum gen. nov., Rhodovibrio gen. nov., Rhodothalassium gen. nov. and Roseospira gen. nov. as well as transfer of Rhodospirillum fulvum to Phaeospirillum fulvum comb. nov., of Rhodospirillum molischianum to Phaeospirillum molischianum comb. nov., of Rhodospirillum salinarum to Rhodovibrio salexigens. Int J Syst Bacteriol 48 Pt 3: 793-798.

36. Lakshmi KV, Sasikala C, Ashok GV, Chandrasekaran R, Ramana CV (2010) *Phaeovibrio sulfidiphilus* gen. nov., sp. nov., phototrophic alphaproteobacterium isolated from brackish water. Int J Syst Evol Microbiol.
37. Zhang D, Yang H, Zhang W, Huang Z, Liu SJ (2003) *Rhodocista pekingensis* sp. nov., a cyst-forming phototrophic bacterium from a municipal wastewater treatment plant. Int J Syst Evol Microbiol 53: 1111-1114.
38. Winkelmann G, Schmidtkunz K, Rainey FA (1996) Characterization of a novel *Spirillum*-like bacterium that degrades ferrioxamine-type siderophores. Biometals 9: 78-83.
39. Pfennig N, Lunsdorf H, Suling J, Imhoff JF (1997) *Rhodospira trueperi* gen. nov., spec. nov., a new phototrophic Proteobacterium of the alpha group. Arch Microbiol 168: 39-45.
40. Favinger J, Stadtwald R, Gest H (1989) *Rhodospirillum centenum*, sp. nov., a thermotolerant cyst-forming anoxygenic photosynthetic bacterium. Antonie Van Leeuwenhoek 55: 291-296.
41. Skerman VBD, Mcgowan V, Sneath PHA (1980) Approved lists of bacterial names. Int J Syst Evol Microbiol 30: 225-420.
42. Reslewic S, Zhou S, Place M, Zhang Y, Briska A, et al. (2005) Whole-genome shotgun optical mapping of *Rhodospirillum rubrum*. Appl Environ Microbiol 71: 5511-5522.
43. Anil Kumar P, Aparna P, Srinivas TN, Sasikala C, Ramana Ch V (2008) *Rhodospirillum sulfurexigens* sp. nov., a phototrophic alphaproteobacterium requiring a reduced sulfur source for growth. Int J Syst Evol Microbiol 58: 2917-2920.
44. Kalyan Chakravarthy S, Srinivas TN, Anil Kumar P, Sasikala C, Ramana Ch V (2007) *Roseospira visakhapatnamensis* sp. nov. and *Roseospira goensis* sp. nov. Int J Syst Evol Microbiol 57: 2453-2457.
45. Guyoneaud R, Moune S, Eatock C, Bothorel V, Hirschler-Rea A, et al. (2002) Characterization of three spiral-shaped purple nonsulfur bacteria isolated from coastal lagoon sediments, saline sulfur springs, and microbial mats: emended description of the genus *Roseospira* and description of *Roseospira marina* sp. nov., *Roseospira navarrensis* sp. nov., and *Roseospira thiosulfatophila* sp. nov. Arch Microbiol 178: 315-324.
46. Weon HY, Kim BY, Hong SB, Joa JH, Nam SS, et al. (2007) *Skermanella aerolata* sp. nov., isolated from air, and emended description of the genus *Skermanella*. Int J Syst Evol Microbiol 57: 1539-1542.
47. Sly LI, Stackebrandt E (1999) Description of *Skermanella parooensis* gen. nov., sp. nov. to accommodate *Conglomeromonas largomobilis* subsp. parooensis following the transfer of *Conglomeromonas largomobilis* subsp. largomobilis to the genus *Azospirillum*. Int J Syst Evol Microbiol 49: 541-544.
48. An H, Zhang L, Tang Y, Luo X, Sun T, et al. (2009) *Skermanella xinjiangensis* sp. nov., isolated from the desert of Xinjiang, China. Int J Syst Evol Microbiol 59: 1531-1534.
49. Sizova MV, Panikov NS, Spiridonova EM, Slobodova NV, Tourova TP (2007) Novel facultative anaerobic acidotolerant *Telmatospirillum siberiense* gen. nov. sp. nov. isolated from mesotrophic fen. Syst Appl Microbiol 30: 213-220.
50. Zhang GI, Hwang CY, Cho BC (2008) *Thalassobaculum litoreum* gen. nov., sp. nov., a member of the family *Rhodospirillaceae* isolated from coastal seawater. Int J Syst Evol Microbiol 58: 479-485.
51. Urios L, Michotey V, Intertaglia L, Lesongeur F, Lebaron P (2010) *Thalassobaculum salexigens* sp. nov., a new member of the family *Rhodospirillaceae* from the NW Mediterranean Sea, and emended description of the genus *Thalassobaculum*. Int J Syst Evol Microbiol 60: 209-213.
52. Lopez-Lopez A, Pujalte MJ, Benlloch S, Mata-Roig M, Rossello-Mora R, et al. (2002) Thalassospira lucentensis gen. nov., sp. nov., a new marine member of the alpha-Proteobacteria. Int J Syst Evol Microbiol 52: 1277-1283.

53. Cui Z, Shao Z (2009) [Predominant strains of polycyclic aromatic hydrocarbon-degrading consortia from deep sea of the Middle Atlantic Ridge]. Wei Sheng Wu Xue Bao 49: 902-909.
54. Liu C, Wu Y, Li L, Ma Y, Shao Z (2007) *Thalassospira xiamenensis* sp. nov. and *Thalassospira profundimaris* sp. nov. Int J Syst Evol Microbiol 57: 316-320.
55. Kodama Y, Stiknowati LI, Ueki A, Ueki K, Watanabe K (2008) *Thalassospira tepidiphila* sp. nov., a polycyclic aromatic hydrocarbon-degrading bacterium isolated from seawater. Int J Syst Evol Microbiol 58: 711-715.
56. Zhao B, Wang H, Li R, Mao X (2010) *Thalassospira xianhensis* sp. nov., a polycyclic aromatic hydrocarbon-degrading marine bacterium. Int J Syst Evol Microbiol 60: 1125-1129.
57. Shi BH, Arunpairojana V, Palakawong S, Yokota A (2002) Tistrella mobilis gen nov, sp nov, a novel polyhydroxyalkanoate-producing bacterium belonging to alpha-Proteobacteria. J Gen Appl Microbiol 48: 335-343.
