# Supplementary material for: Azospirillum Genomes Reveal Transition of Bacteria from Aquatic to Terrestrial Environments
Source: PLoS Genet. 2011 Dec 22;7(12):e1002430. doi: 10.1371/journal.pgen.1002430 (PMC3245306; doi:10.1371/journal.pgen.1002430)
Supplement: Table S2 — Identification of chromids in Azospirillum by house-keeping gene analysis. (PDF) [file pgen.1002430.s005.pdf]

Table S2. Identification of chromids in *Azospirillum* by house-keeping gene analysis

Housekeeping Genes	4B	Sp245	B510
1-(5-phosphoribosyl)-5-[(5-phosphoribosylamino)methylideneamino] imidazole-4-carboxamide isomerase	AZOLI_3051	AZOBR_10372	AZL_028790
16S rRNA processing protein	AZOLI_0372	AZOBR_40189	AZL_005170
1-deoxy-D-xylulose 5-phosphate synthase	AZOLI_p30184	AZOBR_p1140022	AZL_c00990
1-deoxy-D-xylulose-5-phosphate synthase		AZOBR_p140100	
1-hydroxy-2-methyl-2-(E)-butenyl 4-diphosphate synthase	AZOLI_2859	AZOBR_p1110054	AZL_001390
30S ribosomal protein S4	AZOLI_2437	AZOBR_40368	AZL_023540
30S ribosomal protein S9	AZOLI_p10094	AZOBR_100379	AZL_a00320
30S ribosomal subunit protein S1	AZOLI_0174	AZOBR_40092	AZL_002930
30S ribosomal subunit protein S10	AZOLI_0460	AZOBR_150257	AZL_005800
30S ribosomal subunit protein S11	AZOLI_1845	AZOBR_100177	AZL_018770
30S ribosomal subunit protein S12	AZOLI_0455	AZOBR_160004	AZL_005760
30S ribosomal subunit protein S17	AZOLI_0470	AZOBR_150247	AZL_005900
30S ribosomal subunit protein S19	AZOLI_0465	AZOBR_150252	AZL_005850
30S ribosomal subunit protein S2	AZOLI_1070	AZOBR_100274	AZL_016260
30S ribosomal subunit protein S3	AZOLI_0467	AZOBR_150250	AZL_005870
30S ribosomal subunit protein S5	AZOLI_0478	AZOBR_150238	AZL_005980
30S ribosomal subunit protein S7	AZOLI_0456	AZOBR_160003	AZL_005770
30S ribosomal subunit protein S8	AZOLI_0475	AZOBR_150241	AZL_005950
30S ribosomal subunit protein S9		AZOBR_150242	
3-dehydroquinate synthase	AZOLI_2944	AZOBR_40120	AZL_000830
3-isopropylmalate dehydratase, large subunit	AZOLI_2479	AZOBR_40194	AZL_023920
3-isopropylmalate dehydratase, small subunit	AZOLI_0698	AZOBR_200181	AZL_008890
3-isopropylmalate dehydrogenase	AZOLI_0699	AZOBR_200179	AZL_008900
3-methyl-2-oxobutanoate hydroxymethyltransferase	AZOLI_1886	AZOBR_100084	AZL_019370
3-oxoacyl-[acyl-carrier-protein] reductase	AZOLI_1673	AZOBR_110107	AZL_016740
4-hydroxy-3-methylbut-2-enyl diphosphate reductase	AZOLI_1245	AZOBR_150037	AZL_015900
50S ribosomal protein L19	AZOLI_0374	AZOBR_40191	AZL_005190
50S ribosomal protein L31	AZOLI_2402	AZOBR_150224	AZL_023230
50S ribosomal protein L9	AZOLI_1679	AZOBR_110102	AZL_016800
50S ribosomal subunit protein L1	AZOLI_0446	AZOBR_160011	AZL_005690
50S ribosomal subunit protein L10	AZOLI_0448	AZOBR_160010	AZL_005700
50S ribosomal subunit protein L11	AZOLI_0445	AZOBR_160012	AZL_005680
50S ribosomal subunit protein L13	AZOLI_p10093	AZOBR_100378	AZL_a00310
50S ribosomal subunit protein L14	AZOLI_0471	AZOBR_150246	AZL_005910
50S ribosomal subunit protein L15	AZOLI_0480	AZOBR_150236	AZL_006000
50S ribosomal subunit protein L16	AZOLI_0468	AZOBR_150249	AZL_005880
50S ribosomal subunit protein L17	AZOLI_1843	AZOBR_100179	AZL_018750
50S ribosomal subunit protein L18	AZOLI_0477	AZOBR_150239	AZL_005970
50S ribosomal subunit protein L2	AZOLI_0464	AZOBR_150253	AZL_005840
50S ribosomal subunit protein L20	AZOLI_0107	AZOBR_10192	AZL_026000
50S ribosomal subunit protein L22	AZOLI_0466	AZOBR_150251	AZL_005860
50S ribosomal subunit protein L23	AZOLI_0463	AZOBR_150254	AZL_005830
50S ribosomal subunit protein L24	AZOLI_0472	AZOBR_150245	AZL_005920
50S ribosomal subunit protein L27	AZOLI_2409	AZOBR_40325	AZL_023270
50S ribosomal subunit protein L3	AZOLI_0461	AZOBR_150256	AZL_005810
50S ribosomal subunit protein L4	AZOLI_0462	AZOBR_150255	AZL_005820
50S ribosomal subunit protein L5	AZOLI_0473	AZOBR_150244	AZL_005930
50S ribosomal subunit protein L6	AZOLI_0476	AZOBR_150240	AZL_005960
50S ribosomal subunit protein L7/L12	AZOLI_0449	AZOBR_160009	AZL_005710
7,8-Dihydro-6-hydroxymethylpterin-pyrophosphokinase	AZOLI_p10810	AZOBR_p110121	AZL_a04360
A/G-specific adenine glycosylase	AZOLI_p30347	AZOBR_p1130120	AZL_c00560
Acetolactate synthase, large subunit	AZOLI_2237	AZOBR_100110	AZL_021740
Acetolactate synthase, small subunit	AZOLI_2236	AZOBR_100111	AZL_021730

Acetyl-coenzyme A synthetase	AZOLI_2526	AZOBR_10049	AZL_024380
Acetyl-coenzyme A synthetase	AZOLI_2526	AZOBR_10049	AZL_024380
Acetyl-coenzyme A synthetase	AZOLI_2526	AZOBR_10049	AZL_024380
Acetylglutamate kinase	AZOLI_0381	AZOBR_40136	AZL_005260
Adenylate kinase	AZOLI_0482	AZOBR_150234	AZL_006020
Adenylosuccinate synthetase	AZOLI_p30239	AZOBR_150079	AZL_c04110
Adenylosuccinate synthetase	AZOLI_p30239	AZOBR_150079	AZL_c04110
Alanyl-tRNA synthetase	AZOLI_p10762	AZOBR_150171	AZL_a04560
Amidophosphoribosyltransferase	AZOLI_1187	AZOBR_110090	AZL_017000
Anthranilate phosphoribosyltransferase	AZOLI_1096	AZOBR_100304	AZL_016010
Anthranilate synthase component I	AZOLI_1036	AZOBR_100311	AZL_012360
Argininosuccinate lyase	AZOLI_p40218	AZOBR_p140086	AZL_d01140
Argininosuccinate synthase	AZOLI_2761	AZOBR_10163	AZL_002110
Arginyl-tRNA synthetase	AZOLI_p10771	AZOBR_p120117	AZL_a04470
Aspartyl-tRNA synthetase	AZOLI_1069	AZOBR_p130143	AZL_016270
ATP-dependent chaperone	AZOLI_2850	AZOBR_p1110063	AZL_001460
ATP-dependent chaperone	AZOLI_p30461	AZOBR_p170093	AZL_c04990
ATP-dependent chaperone		AZOBR_p210122	
ATP-dependent Clp protease proteolytic subunit	AZOLI_1860	AZOBR_100236	AZL_019170
ATP-dependent DNA helicase	AZOLI_1658	AZOBR_140194	AZL_016620
Bifunctional: 2-C-methyl-D-erythritol 4-phosphate cytidylyltransferase/ 2-C-methyl-D-erythritol 2,4-cyclodiphosphate synthase	AZOLI_1339	AZOBR_p110006	AZL_015110
Bifunctional: 5,10-methylene-tetrahydrofolate dehydrogenase; 5,10-methylene-tetrahydrofolate cyclohydrolase	AZOLI_2920	AZOBR_10134	AZL_026930
Bifunctional: 5,10-methylene-tetrahydrofolate dehydrogenase; 5,10-methylene-tetrahydrofolate cyclohydrolase			AZL_a09610
Bifunctional: diaminohydroxyphosphoribosylaminopyrimidine deaminase/5-amino-6-(5-phosphoribosylamino) uracil reductase	AZOLI_0872	AZOBR_140241	AZL_010180
Bifunctional: IMP cyclohydrolase/phosphoribosylaminoimidazolecarboxamide formyltransferase	AZOLI_2745	AZOBR_10040	AZL_002390
Carbamoyl phosphate synthase, large subunit	AZOLI_0141	AZOBR_180250	AZL_002620
Carbamoyl phosphate synthetase, small subunit	AZOLI_0928	AZOBR_150132	AZL_010540
Carboxy-terminal-processing peptidase S41A	AZOLI_2415	AZOBR_40311	AZL_023320
Cell division protein FtsH; ATP-dependent metalloprotease	AZOLI_0837	AZOBR_100329	AZL_009880
Cell division protein FtsZ	AZOLI_2137	AZOBR_180108	AZL_020880
Chaperone protein, heat shock protein (Hsp40)	AZOLI_2991	AZOBR_10494	AZL_000400
Chaperone protein, heat shock protein (HSP70)	AZOLI_2990	AZOBR_10493	AZL_000410
Chorismate synthase	AZOLI_2271	AZOBR_150090	AZL_022220
Chromosomal replication initiator protein dnaA	AZOLI_0002	AZOBR_10277	AZL_026690
Chromosome partitioning protein	AZOLI_3020	AZOBR_100099	AZL_000140
Chromosome partitioning protein	AZOLI_3019	AZOBR_10404	AZL_000150
Component in transcription antitermination	AZOLI_0443	AZOBR_160013	AZL_005670
Conserved protein of unknown function	AZOLI_p10096	AZOBR_110019	AZL_a00340
Conserved protein of unknown function	AZOLI_0824	AZOBR_140009	AZL_009760
Conserved protein of unknown function	AZOLI_2419	AZOBR_40317	AZL_023360
Conserved protein of unknown function; putative ATPase domain	AZOLI_3166	AZOBR_10116	AZL_027170
Conserved protein of unknown function; putative cysteine hydrolases domain	AZOLI_2288	AZOBR_p440149	AZL_022340
Conserved protein of unknown function; putative kinase domain	AZOLI_3165	AZOBR_10117	AZL_027180
Conserved protein of unknown function; putative PLP-binding barrel domain	AZOLI_0060	AZOBR_10415	AZL_026290
CTP synthase	AZOLI_1224	AZOBR_100316	AZL_013700
Cysteinyl-tRNA synthetase	AZOLI_2357	AZOBR_40357	AZL_022910
Cytidine and deoxycytidylate deaminase	AZOLI_1948	AZOBR_40412	AZL_019820
D-alanine-D-alanine ligase	AZOLI_2134	AZOBR_180105	AZL_020850
Delta-aminolevulinic acid dehydratase	AZOLI_1110	AZOBR_110120	AZL_014030

Dephospho-CoA kinase	AZOLI_3030	AZOBR_10393	AZL_000040
Diaminopimelate decarboxylase	AZOLI_p40216	AZOBR_p140088	AZL_d01130
Dihydropteroate synthase	AZOLI_p50060	AZOBR_100215	AZL_e01240
Dihydroxyacid dehydratase (DAD)	AZOLI_p40147	AZOBR_p110151	AZL_d00200
Dimethyladenosine transferase	AZOLI_1824	AZOBR_p130171	AZL_018600
dITP/XTP pyrophosphatase	AZOLI_3016	AZOBR_10485	AZL_000180
Division-specific transpeptidase	AZOLI_2124	AZOBR_180093	AZL_020760
DNA gyrase subunit B	AZOLI_3153	AZOBR_p170041	AZL_027280
DNA gyrase, subunit A	AZOLI_1235	AZOBR_p120069	AZL_013920
DNA helicase II	AZOLI_1919	AZOBR_180127	AZL_019670
DNA ligase	AZOLI_2143	AZOBR_180112	AZL_020910
DNA mismatch repair protein MutL	AZOLI_1931	AZOBR_70002	AZL_019750
DNA mismatch repair protein MutS	AZOLI_1558	AZOBR_10469	AZL_017120
DNA polymerase I	AZOLI_p30313	AZOBR_p440034	AZL_c04610
DNA polymerase III (gamma and tau subunits)	AZOLI_2632	AZOBR_40273	AZL_025120
DNA polymerase III, alpha subunit	AZOLI_p40433	AZOBR_100271	AZL_d01720
DNA polymerase III, beta-subunit	AZOLI_3155	AZOBR_p170039	AZL_027260
DNA primase	AZOLI_0931	AZOBR_150129	AZL_010570
DNA processing single strand binding protein	AZOLI_1814	AZOBR_p130067	AZL_018520
DNA repair protein	AZOLI_1185	AZOBR_110092	AZL_017020
DNA repair protein RecN	AZOLI_1909	AZOBR_180116	AZL_019580
DNA translocase	AZOLI_0155	AZOBR_10505	AZL_002770
DNA translocase	AZOLI_p40638	AZOBR_180242	AZL_d00740
DNA-(apurinic or apyrimidinic site) lyase	AZOLI_3098	AZOBR_10347	AZL_028370
DNA-directed RNA polymerase alpha chain	AZOLI_1844	AZOBR_100178	AZL_018760
Elongation factor G (EF-G)	AZOLI_0457	AZOBR_150110	AZL_005780
Elongation factor G (EF-G)	AZOLI_1851	AZOBR_160002	AZL_018800
Elongation factor Ts (EF-Ts)	AZOLI_1071	AZOBR_100276	AZL_016250
Elongation factor Tu (EF-Tu)	AZOLI_0439	AZOBR_160001	AZL_005650
Elongation factor Tu (EF-Tu)	AZOLI_0458	AZOBR_160015	AZL_005790
Enolase (2-phosphoglycerate dehydratase)	AZOLI_p10268	AZOBR_100325	AZL_a02700
Enolase (2-phosphoglycerate dehydratase)	AZOLI_p20459	AZOBR_p420024	AZL_b03090
Excinuclease ABC, subunit A	AZOLI_1383	AZOBR_p120082	AZL_014850
Excinulease of the UvrABC repair system	AZOLI_1261	AZOBR_p130191	AZL_015800
Ferric uptake regulator	AZOLI_2595	AZOBR_10297	AZL_024860
Ferric uptake regulator	AZOLI_2652	AZOBR_200223	AZL_026170
Ferric uptake regulator		AZOBR_p150055	
FolC bifunctional protein [Tetrahydrofolate synthase; Dihydrofolate synthase]	AZOLI_2925	AZOBR_10102	AZL_027050
Fused UDP-N-acetylglucosamine pyrophosphate/glucosamine-1-phosphate acetyl transferase	AZOLI_p10414	AZOBR_140159	AZL_a07260
Gamma-glutamyl phosphate reductase	AZOLI_2421	AZOBR_40319	AZL_023380
Glucosamine-fructose-6-phosphate aminotransferase	AZOLI_p10413	AZOBR_140158	AZL_a07270
Glucose-inhibited division protein A	AZOLI_3022	AZOBR_10401	AZL_000120
Glutamate 5-kinase	AZOLI_2412	AZOBR_40323	AZL_023290
Glutamate synthase, alpha subunit	AZOLI_2890	AZOBR_p1110032	AZL_000990
Glutamate synthase, alpha subunit		AZOBR_p1110033	
Glutamine synthetase	AZOLI_1867	AZOBR_100228	AZL_019220
Glutamyl-tRNA synthetase	AZOLI_1089	AZOBR_100296	AZL_016080
Glyceraldehyde-3-phosphate dehydrogenase (GAPDH)	AZOLI_0815	AZOBR_150011	AZL_009710
Glyceraldehyde-3-phosphate dehydrogenase (GAPDH)	AZOLI_p20464		AZL_b03040
Glycerol-3-phosphate dehydrogenase (NAD+)	AZOLI_0257	AZOBR_10060	AZL_004060
GMP synthase (glutamine-hydrolyzing)	AZOLI_1516	AZOBR_p130108	AZL_011710
GTPase involved in cell partioning and DNA repair	AZOLI_2411	AZOBR_40324	AZL_023280
GTP-binding elongation factor	AZOLI_0118	AZOBR_10180	AZL_025910
GTP-binding protein TypA/BipA	AZOLI_1055	AZOBR_140217	AZL_012510
GTP-dependent nucleic acid-binding protein	AZOLI_1962	AZOBR_p440026	AZL_019980
Heat Shock Chaperone (HSP-70 cofactor)	AZOLI_3110	AZOBR_10488	AZL_028250

Histidinol dehydrogenase	AZOLI_p40530	AZOBR_p1120021	AZL_d03600
Histidyl-tRNA synthetase	AZOLI_1957	AZOBR_40406	AZL_019900
Holliday junction DNA helicase ruvA	AZOLI_0826	AZOBR_140011	AZL_009780
Holliday junction DNA helicase ruvB	AZOLI_0827	AZOBR_140012	AZL_009790
Holliday junction resolvase RuvC	AZOLI_0825	AZOBR_140010	AZL_009770
Hydroxymethylbilane synthase	AZOLI_0255	AZOBR_10056	AZL_004040
Imidazole glycerol phosphate synthase, HisF subunit	AZOLI_3052	AZOBR_10373	AZL_028780
Imidazole glycerol phosphate synthase, HisH subunit	AZOLI_3049	AZOBR_10370	AZL_028810
Imidazoleglycerol-phosphate dehydratase	AZOLI_3048	AZOBR_10369	AZL_028820
Indole-3-glycerol-phosphate synthase	AZOLI_1095	AZOBR_100303	AZL_016020
Inner membrane insertion protein	AZOLI_0378	AZOBR_40140	AZL_005230
Inosine-5'-monophosphate dehydrogenase	AZOLI_2145	AZOBR_p130122	AZL_020930
Isoleucine tRNA synthetase	AZOLI_0609	AZOBR_70014	AZL_007060
Isopentenyl-adenosine A37 tRNA methylthiolase	AZOLI_3087	AZOBR_10327	AZL_028470
Ketol-acid reductoisomerase	AZOLI_2232	AZOBR_100112	AZL_021700
Large subunit of chaperonin GroESL	AZOLI_2685	AZOBR_20016	AZL_003460
Large subunit of chaperonin GroESL	AZOLI_p10389	AZOBR_p170096	AZL_a01300
Large subunit of chaperonin GroESL	AZOLI_p10728	AZOBR_p270280	AZL_c02850
Leucine tRNA synthetase	AZOLI_0067	AZOBR_10408	AZL_026240
Lipoyl synthase	AZOLI_0731	AZOBR_50039	AZL_009030
Lipoyl synthase	AZOLI_1335	AZOBR_p110003	AZL_015150
Methionine aminopeptidase	AZOLI_0227	AZOBR_110093	AZL_017050
Methionine aminopeptidase	AZOLI_1183	AZOBR_40394	AZL_025320
Methionyl-tRNA formyltransferase	AZOLI_2491	AZOBR_40200	AZL_024040
Methionyl-tRNA synthetase	AZOLI_1724	AZOBR_130005	AZL_018180
Modification methylase HemK	AZOLI_2855	AZOBR_p1110058	AZL_001420
N-acetyl-gamma-glutamyl-phosphate reductase	AZOLI_p10095	AZOBR_100380	AZL_a00330
Nucleoside diphosphate kinase	AZOLI_0990	AZOBR_p130163	AZL_018050
Octaprenyl diphosphate synthase	AZOLI_p10352	AZOBR_180071	AZL_a03800
O-sialoglycoprotein endopeptidase	AZOLI_0256	AZOBR_10058	AZL_004050
Pantothenate synthetase	AZOLI_p10480	AZOBR_140166	AZL_a03250
Peptide chain release factor 1	AZOLI_2857	AZOBR_p1110056	AZL_001410
Peptide methionine sulfoxide reductase	AZOLI_0157	AZOBR_180235	AZL_002790
Peptide methionine sulfoxide reductase msrB	AZOLI_p50224	AZOBR_30021	AZL_e02560
Peptidyl-tRNA hydrolase	AZOLI_1961	AZOBR_p440154	AZL_019940
Phenylalanyl-tRNA synthetase alpha chain	AZOLI_0108	AZOBR_10191	AZL_025990
Phenylalanyl-tRNA synthetase beta chain	AZOLI_0109	AZOBR_10189	AZL_025980
Phosphatidate cytidylyltransferase	AZOLI_1075	AZOBR_100280	AZL_016210
Phosphoadenosine phosphosulfate reductase	AZOLI_0771	AZOBR_140266	AZL_009340
Phosphoenolpyruvate-protein phosphotransferase	AZOLI_2958	AZOBR_p430050	AZL_000710
Phosphoglycerate kinase	AZOLI_2630	AZOBR_40277	AZL_025100
Phospho-N-acetylmuramoyl-pentapeptide transferase	AZOLI_2127	AZOBR_180096	AZL_020790
Phosphopantetheine adenylyltransferase	AZOLI_1234	AZOBR_p120068	AZL_013910
Phosphopantothenoylcysteine decarboxylase (CoaC); Phosphopantothenatecysteine ligase (CoaB)	AZOLI_2934	AZOBR_10233	AZL_027120
Phosphoribosylamine-glycine ligase	AZOLI_1951	AZOBR_40409	AZL_019850
Phosphoribosylaminoimidazole carboxylase	AZOLI_1432	AZOBR_100015	AZL_017460
Phosphoribosyl-AMP cyclohydrolase	AZOLI_1585	AZOBR_120038	AZL_012880
Phosphoribosylformylglycinamidine cyclo-ligase	AZOLI_1191	AZOBR_p130159	AZL_013100
Phosphoribosylglycinamide formyltransferase	AZOLI_1190	AZOBR_p130160	AZL_013090
Polynucleotide phosphorylase	AZOLI_3068	AZOBR_10306	AZL_028630
Prephenate dehydratase	AZOLI_0294	AZOBR_40270	AZL_004330
Preprotein translocase secA subunit	AZOLI_0218	AZOBR_40040	AZL_025630
Preprotein translocase secY subunit	AZOLI_0481	AZOBR_150235	AZL_006010
Primosomal protein N' (replication factor Y)	AZOLI_2610	AZOBR_40300	AZL_024970
Protein-export membrane protein secD	AZOLI_1395	AZOBR_p120096	AZL_014530
Pseudouridine synthase	AZOLI_p40053	AZOBR_150074	AZL_d04590
Putative 4Fe-4S ferredoxin, iron-sulfur binding	AZOLI_2153	AZOBR_100045	AZL_021000

Putative ABC transporter, ATP-binding component	AZOLI_2873	AZOBR_p1110044	AZL_001080
Putative alanine racemase (alr)	AZOLI_p50284	AZOBR_110098	AZL_e01720
Putative ATPase of the MinD/MRP superfamily	AZOLI_p40466	AZOBR_10403	AZL_013580
Putative deoxyribonuclease TadD-like	AZOLI_1725	AZOBR_130006	AZL_018190
Putative Glutamate racemase	AZOLI_2249	AZOBR_110034	AZL_021850
Putative metalloprotease	AZOLI_3085	AZOBR_10325	AZL_028490
Putative N-acetylmuramoyl-L-alanine amidase	AZOLI_0387	AZOBR_170005	AZL_005320
Putative N-acetylmuramoyl-L-alanine amidase I	AZOLI_p10327	AZOBR_p1160055	AZL_a03600
Putative oxygen-independent coproporphyrinogen III oxidase (HemN-like)	AZOLI_3017	AZOBR_10484	AZL_000170
Putative peptidase	AZOLI_2985	AZOBR_10335	AZL_000460
Putative Peroxide-responsive repressor (PerR-like), fur family	AZOLI_0078	AZOBR_10218	AZL_003780
Putative phosphate starvation-inducible protein, PhoH-like	AZOLI_3086	AZOBR_10326	AZL_028480
Putative RNA methyltransferase	AZOLI_2705	AZOBR_10263	AZL_003330
Putative tetrapyrrole methyltransferase	AZOLI_3124	AZOBR_10476	AZL_028060
Putative transferase (transferase hexapeptide repeat)	AZOLI_p20453		AZL_b00660
Putative translation factor (Sua5-like)	AZOLI_2476	AZOBR_70173	AZL_023880
Pyrroline-5-carboxylate reductase		AZOBR_100037	AZL_017290
Pyrroline-5-carboxylate reductase		AZOBR_p230015	
Pyruvate kinase	AZOLI_p10527	AZOBR_100175	AZL_a09040
Pyruvate kinase		AZOBR_p420013	
Recombinase A (recA)	AZOLI_1478	AZOBR_150173	AZL_012130
Recombination protein RecR	AZOLI_2635	AZOBR_40275	AZL_025140
Replicative DNA helicase	AZOLI_p50283	AZOBR_110099	AZL_e01730
Riboflavin kinase and FAD synthetase	AZOLI_0507	AZOBR_70017	AZL_006230
Riboflavin synthase, beta subunit	AZOLI_0869	AZOBR_140244	AZL_010150
Ribonuclease HII	AZOLI_p40328	AZOBR_p1130126	AZL_d02320
Ribonuclease R	AZOLI_2273	AZOBR_p130133	AZL_022230
Ribose-phosphate pyrophosphokinase (RPPK)	AZOLI_2290	AZOBR_p440152	AZL_022350
Ribosomal RNA large subunit methyltransferase N	AZOLI_2819	AZOBR_10166	AZL_001710
Ribosome recycling factor	AZOLI_1073	AZOBR_100278	AZL_016230
Ribosome-associated GTPase	AZOLI_1166	AZOBR_140309	AZL_017090
Ribulose-phosphate 3-epimerase	AZOLI_1322	AZOBR_100154	AZL_015270
Ribulose-phosphate 3-epimerase	AZOLI_p10722		
RNA polymerase sigma factor (sigma70)	AZOLI_0932	AZOBR_150128	AZL_010580
RNA polymerase, beta prime subunit	AZOLI_0451	AZOBR_160006	AZL_005730
S-adenosyl-dependent methyl transferase	AZOLI_2122	AZOBR_180091	AZL_020740
S-adenosylmethionine synthetase	AZOLI_3081	AZOBR_10320	AZL_028530
S-adenosylmethionine:tRNA ribosyltransferase-isomerase	AZOLI_2150	AZOBR_100049	AZL_020970
Samll subunit of chaperonin GroESL	AZOLI_2686	AZOBR_20015	AZL_003450
Serine acetyltransferase	AZOLI_0542	AZOBR_70110	AZL_006530
Serine hydroxymethyltransferase	AZOLI_0862	AZOBR_140239	AZL_010090
Serine hydroxymethyltransferase	AZOLI_p10855		AZL_c01670
Serine O-acetyltransferase	AZOLI_1974	AZOBR_70184	AZL_020070
Seryl-tRNA synthetase	AZOLI_1401	AZOBR_p120104	AZL_014470
Shikimate dehydrogenase	AZOLI_3031	AZOBR_10392	AZL_000030
Shikimate kinase	AZOLI_2943	AZOBR_40121	AZL_000840
Signal peptidase I	AZOLI_1505	AZOBR_p1170091	AZL_011810
Single-stranded DNA-binding protein	AZOLI_1365	AZOBR_p120080	AZL_014860
Single-stranded-DNA-specific exonuclease recJ	AZOLI_0789	AZOBR_100398	AZL_009490
Site-specific tyrosine recombinase	AZOLI_2940	AZOBR_40126	AZL_000870
sn-glycerol-3-phosphate dehydrogenase FAD/NAD(P)-binding (aerobic)	AZOLI_p50197	AZOBR_p280087	AZL_e03080
Succinyl-CoA synthetase, alpha subunit, NAD(P)-binding	AZOLI_0205	AZOBR_200164	AZL_025770
Succinyl-CoA synthetase, beta subunit	AZOLI_0206	AZOBR_200163	AZL_025760
Succinyl-CoA synthetase, beta subunit	AZOLI_p10320		AZL_a03540
Succinyl-CoA synthetase, NAD(P)-binding, alpha subunit	AZOLI_p10321		AZL_a03550
TatABCE protein translocation system subunit	AZOLI_p10763	AZOBR_p120107	AZL_a04550

Thiamine-phosphate synthase	AZOLI_0809	AZOBR_150017	AZL_009640
Thioredoxin 1, redox factor	AZOLI_3159	AZOBR_10124	AZL_027220
Threonyl-tRNA synthetase	AZOLI_2575	AZOBR_200035	AZL_024740
Thymidylate kinase	AZOLI_1722	AZOBR_130003	AZL_018160
Transaldolase	AZOLI_2611	AZOBR_40299	AZL_024980
Transcription repair coupling factor	AZOLI_1467	AZOBR_140180	AZL_012200
Transcription termination factor	AZOLI_3077	AZOBR_10313	AZL_028570
Transferase involved in phenylacetate degradation	AZOLI_2570	AZOBR_100381	AZL_024690
Transketolase (TK)	AZOLI_0816	AZOBR_140002	AZL_009720
Transketolase (TK)	AZOLI_p10717		
Transketolase (TK)	AZOLI_p20253		
Translation initiation factor IF-1	AZOLI_p40538	AZOBR_p1120026	AZL_d03670
Translation initiation factor IF-2	AZOLI_3075		AZL_028590
Trans-translation protein (ssrA RNA-binding protein)	AZOLI_1104	AZOBR_110115	AZL_013960
Triosephosphate isomerase	AZOLI_1222	AZOBR_100314	AZL_013680
tRNA (5-methylaminomethyl-2-thiouridylate)-methyltransferase	AZOLI_1980	AZOBR_70189	AZL_020120
tRNA (Guanine-N(1)-)-methyltransferase	AZOLI_0373	AZOBR_40190	AZL_005180
tRNA (guanine-N(7)-)-methyltransferase	AZOLI_3080	AZOBR_10319	AZL_028540
tRNA delta(2)-isopentenylpyrophosphate transferase	AZOLI_2239	AZOBR_100108	AZL_021760
tRNA modification GTPase	AZOLI_3023	AZOBR_10400	AZL_000110
tRNA pseudouridine synthase	AZOLI_3071	AZOBR_10309	AZL_028610
tRNA-dihydrouridine synthase B	AZOLI_1341	AZOBR_p110008	AZL_015090
tRNA-guanine transglycosylase	AZOLI_2151	AZOBR_100048	AZL_020980
Tryptophan synthase, alpha subunit	AZOLI_2929	AZOBR_10104	AZL_027070
Tryptophan synthase, beta subunit	AZOLI_2930	AZOBR_10105	AZL_027080
Two component sensor histidine kinase; phosphate regulon	AZOLI_3010	AZOBR_10247	AZL_000230
Two-component response regulator; phosphate regulon	AZOLI_3004	AZOBR_10241	AZL_000280
Tyrosine tRNA synthetase	AZOLI_0340	AZOBR_70181	AZL_004860
Tyrosine tRNA synthetase	AZOLI_2471	AZOBR_p170010	AZL_023840
Ubiquinone/menaquinone biosynthesis methyltransferase	AZOLI_0096	AZOBR_10285	AZL_026020
UDP-glucose 4-epimerase	AZOLI_p20384	AZOBR_p210032	AZL_a07190
UDP-N-acetylglucosamine 1-carboxyvinyltransferase	AZOLI_2260	AZOBR_140031	AZL_021950
UDP-N-acetylglucosamine:N-acetylmuramyl-(pentapeptide) pyrophosphoryl-undecaprenol N-acetylglucosamine transferase	AZOLI_2130	AZOBR_180099	AZL_020820
UDP-N-acetyl-muramate:alanine ligase	AZOLI_2132	AZOBR_180103	AZL_020830
UDP-N-acetylmuramoylalanine-D-glutamate ligase	AZOLI_2128	AZOBR_180097	AZL_020800
UDP-N-acetylmuramoyl-L-alanyl-D-glutamate-2,6-diaminopimelate ligase	AZOLI_2125	AZOBR_180094	AZL_020770
UDP-N-acetylmuramoyl-tripeptide-D-alanyl-D-alanine ligase	AZOLI_2126	AZOBR_180095	AZL_020780
Undecaprenyl pyrophosphate synthetase	AZOLI_1074	AZOBR_100279	AZL_016220
Uridylate kinase	AZOLI_1072	AZOBR_100277	AZL_016240
Uroporphyrinogen-III C-methyltransferase	AZOLI_p40238	AZOBR_p140042	AZL_d03380
Valyl-tRNA synthetase	AZOLI_1559	AZOBR_120063	AZL_013080
Zinc metalloprotease	AZOLI_1077	AZOBR_100282	AZL_016190

Table of proteins present in all chromid containing genomes [1], including their genomic location (as locus tags) in the 3 strains of *Azospirillum*. Those genes that remained on the ancestral/primary chromosome are colored in white. Those genes that migrated to a different replicon in 4B or B510 are in red. Those genes that migrated to a different replicon in Sp245 are in orange. Those genes that migrated to a different replicon in all 3 strains are in blue.

References:

1. Harrison PW, Lower RP, Kim NK, Young JP (2010) Introducing the bacterial 'chromid': not a chromosome, not a plasmid. Trends Microbiol 18: 141-148.
