# Supplementary material for: Azospirillum Genomes Reveal Transition of Bacteria from Aquatic to Terrestrial Environments
Source: PLoS Genet. 2011 Dec 22;7(12):e1002430. doi: 10.1371/journal.pgen.1002430 (PMC3245306; doi:10.1371/journal.pgen.1002430)
Supplement: Table S3 — Identification of chromids in Azospirillum by GC content. (PDF) [file pgen.1002430.s006.pdf]

Table S3. Identification of chromids in *Azospirillum* by GC content

Replicon	G+C content (%)	Chromid GC content cutoff (%) ±0.92% difference with host chromosome
***A. lipoferum* 4B**		
**AZOLI**	67.61	**68.32 – 66.99**
**AZOLI_p1**	67.57	
**AZOLI_p2**	67.59	
**AZOLI_p3**	67.82	
**AZOLI_p4**	68.30	**outside cutoff**
**AZOLI_p5**	67.73	
**AZOLI_p6**	67.08	
		**Chromid GC content cutoff (%) ±0.92% difference with host chromosome**
***A. brasilense* Sp245**		
**AZOBR**	68.61	**69.24 – 67.98**
**AZOBR_p1**	68.65	
**AZOBR_p2**	68.34	
**AZOBR_p3**	68.23	
**AZOBR_p4**	68.99	
**AZOBR_p5**	66.69	**outside cutoff**
**AZOBR_p6**	66.76	**outside cutoff**
		**Chromid GC content cutoff (%) ±0.92% difference with host chromosome**
***A.* sp. B510**		
**AZL**	67.76	**68.38 – 67.14**
**AZL_a**	67.64	
**AZL_b**	67.46	
**AZL_c**	67.40	
**AZL_d**	67.97	
**AZL_e**	67.51	
**AZL_f**	65.88	**outside cutoff**

As described by Harrison *et al.* [1], GC content was calculated for all the replicons. A cutoff value was calculated as GC within 0.521 ± 0.399% (mean ± standard deviation) of the host chromosome. Those labeled in red are outside this cutoff for chromid definition.

References:

1. Harrison PW, Lower RP, Kim NK, Young JP (2010) Introducing the bacterial 'chromid': not a chromosome, not a plasmid. Trends Microbiol 18: 141-148.
