# Supplementary material for: Azospirillum Genomes Reveal Transition of Bacteria from Aquatic to Terrestrial Environments
Source: PLoS Genet. 2011 Dec 22;7(12):e1002430. doi: 10.1371/journal.pgen.1002430 (PMC3245306; doi:10.1371/journal.pgen.1002430)
Supplement: Table S4 — ANI analysis of Azospirillum and rhizobial genomes. (PDF) [file pgen.1002430.s007.pdf]

Table S4. ANI analysis of *Azospirillum* and rhizobial genomes

Pair of strains*	Number of MUMs	MUMs (bp)	ANIm (%)	Coverage (%)	Genetic Distance †
4B vs B510	1964	4 782 709	91	71	0.0114
4B vs Sp245	1637	2 012 936	89	33	0.0972
CFN42 vs Rl3841	649	2 796 109	89	43	0.0215
CFN42 vs Sm1021	590	745403	84	11	0.110

* 4B, *A. lipoferum* ; B510, *Azospirillum* sp. ; Sp245, *A. brasilense* ; CFN42, *Rhizobium etli* ; Rl3841, *Rhizobium leguminosarum* biovar *viciae* ; Sm1021, *Sinorhizobium meliloti*.

† Genetic Distance based on concatenated ribosomal protein tree.

ANIm is the Average Nucleotide Identity calculated from the maximal unique matches (MUMs) determined by the MUMmer 2.1 program in pairwise comparisons [1].

References:

1. Delcher AL, Phillippy A, Carlton J, Salzberg SL (2002) Fast algorithms for large-scale genome alignment and comparison. Nucleic Acids Res 30: 2478-2483.
