# Supplementary material for: Azospirillum Genomes Reveal Transition of Bacteria from Aquatic to Terrestrial Environments
Source: PLoS Genet. 2011 Dec 22;7(12):e1002430. doi: 10.1371/journal.pgen.1002430 (PMC3245306; doi:10.1371/journal.pgen.1002430)
Supplement: Table S5 — Recombination hotspots in Azospirillum genomes. (PDF) [file pgen.1002430.s008.pdf]

Table S5. Recombination hotspots in *Azospirillum* genomes

Strains[a]	Direct[b] repeats (>80bp)	Palindromic[c] repeats (>80bp)	IS[d] elements (potentially active)	CRISPR[e]
4B	497	412	99 (55)	126
B510	1720	1406	310 (176)	153
Sp245	283	256	ND	12

[a]4B, *A. lipoferum* ; B510, *Azospirillum* sp. ; Sp245, *A. brasilense.*

[bc]Direct and palindromic repeats were calculated using the repfind application of REPUTER with the default parameters [1].

[d]IS, Insertion sequences. ND, not determined.

[e]CRISPR, Clustered Regularly Interspaced Short Palindromic Repeats.

References:

1. Kurtz S, Choudhuri JV, Ohlebusch E, Schleiermacher C, Stoye J, et al. (2001) REPuter: the manifold applications of repeat analysis on a genomic scale. Nucleic Acids Res 29: 4633-4642.
