# Supplementary material for: Azospirillum Genomes Reveal Transition of Bacteria from Aquatic to Terrestrial Environments
Source: PLoS Genet. 2011 Dec 22;7(12):e1002430. doi: 10.1371/journal.pgen.1002430 (PMC3245306; doi:10.1371/journal.pgen.1002430)
Supplement: Table S6 — Origin of Azospirillum genes. (PDF) [file pgen.1002430.s009.pdf]

Table S6. Origin of *Azospirillum* genes

Locus Tag (4B)	Ancestry Assignment	Locus Tag (Sp245)	Ancestry Assignment
AZOLI_0001	Horizontally Transferred (high confidence)	AZOBR_10001	Unassigned
AZOLI_0002	Ancestral (high confidence)	AZOBR_10002	Horizontally Transferred (high confidence)
AZOLI_0003	Horizontally Transferred (high confidence)	AZOBR_10003	Ancestral (low confidence)
AZOLI_0004	Ancestral (medium confidence)	AZOBR_10004	Horizontally Transferred (high confidence)
AZOLI_0005	Ancestral (medium confidence)	AZOBR_10005	Ancestral (low confidence)
AZOLI_0006	Ancestral (medium confidence)	AZOBR_10006	Horizontally Transferred (high confidence)
AZOLI_0007	Ancestral (high confidence)	AZOBR_10007	Unassigned
AZOLI_0008	Ancestral (medium confidence)	AZOBR_10008	Ancestral (low confidence)
AZOLI_0009	Horizontally Transferred (high confidence)	AZOBR_10009	Ancestral (high confidence)
AZOLI_0010	Ancestral (low confidence)	AZOBR_10010	Ancestral (high confidence)
AZOLI_0011	Ancestral (low confidence)	AZOBR_10011	Ancestral (low confidence)
AZOLI_0012	Ancestral (high confidence)	AZOBR_10012	Horizontally Transferred (high confidence)
AZOLI_0013	Unassigned	AZOBR_10013	Ancestral (low confidence)
AZOLI_0014	Horizontally Transferred (low confidence)	AZOBR_10014	Ancestral (low confidence)
AZOLI_0015	Horizontally Transferred (high confidence)	AZOBR_10015	Horizontally Transferred (medium confidence)
AZOLI_0016	Horizontally Transferred (high confidence)	AZOBR_10016	Horizontally Transferred (high confidence)
AZOLI_0018	Unassigned	AZOBR_10017	Horizontally Transferred (high confidence)
AZOLI_0019	Unassigned	AZOBR_10018	Ancestral (high confidence)
AZOLI_0020	Unassigned	AZOBR_10019	Ancestral (medium confidence)
AZOLI_0021	Unassigned	AZOBR_10020	Ancestral (medium confidence)
AZOLI_0022	Unassigned	AZOBR_10021	Unassigned
AZOLI_0023	Unassigned	AZOBR_10022	Ancestral (medium confidence)
AZOLI_0024	Unassigned	AZOBR_10023	Ancestral (medium confidence)
AZOLI_0025	Ancestral (medium confidence)	AZOBR_10024	Horizontally Transferred (high confidence)
AZOLI_0026	Horizontally Transferred (high confidence)	AZOBR_10025	Ancestral (low confidence)
AZOLI_0027	Ancestral (medium confidence)	AZOBR_10026	Horizontally Transferred (high confidence)
AZOLI_0028	Ancestral (medium confidence)	AZOBR_10027	Ancestral (high confidence)
AZOLI_0029	Horizontally Transferred (low confidence)	AZOBR_10028	Horizontally Transferred (high confidence)
AZOLI_0030	Unassigned	AZOBR_10029	Unassigned
AZOLI_0031	Ancestral (medium confidence)	AZOBR_10030	Unassigned
AZOLI_0032	Ancestral (medium confidence)	AZOBR_10031	Horizontally Transferred (high confidence)
AZOLI_0033	Horizontally Transferred (high confidence)	AZOBR_10032	Ancestral (high confidence)
AZOLI_0034	Horizontally Transferred (high confidence)	AZOBR_10033	Horizontally Transferred (high confidence)
AZOLI_0035	Ancestral (medium confidence)	AZOBR_10034	Unassigned
AZOLI_0036	Ancestral (high confidence)	AZOBR_10035	Horizontally Transferred (high confidence)
AZOLI_0037	Ancestral (high confidence)	AZOBR_10036	Unassigned
AZOLI_0038	Ancestral (high confidence)	AZOBR_10037	Horizontally Transferred (high confidence)
AZOLI_0039	Unassigned	AZOBR_10038	Ancestral (medium confidence)
AZOLI_0041	Unassigned	AZOBR_10039	Horizontally Transferred (high confidence)
AZOLI_0044	Horizontally Transferred (high confidence)	AZOBR_10040	Ancestral (medium confidence)
AZOLI_0046	Horizontally Transferred (high confidence)	AZOBR_10041	Ancestral (medium confidence)
AZOLI_0047	Horizontally Transferred (high confidence)	AZOBR_10042	Unassigned
AZOLI_0048	Ancestral (medium confidence)	AZOBR_10043	Ancestral (high confidence)
AZOLI_0049	Horizontally Transferred (medium confidence)	AZOBR_10044	Horizontally Transferred (high confidence)
AZOLI_0050	Ancestral (high confidence)	AZOBR_10045	Ancestral (high confidence)
AZOLI_0051	Ancestral (medium confidence)	AZOBR_10046	Ancestral (low confidence)
AZOLI_0052	Ancestral (low confidence)	AZOBR_10047	Ancestral (medium confidence)
AZOLI_0053	Ancestral (medium confidence)	AZOBR_10048	Ancestral (low confidence)
AZOLI_0054	Horizontally Transferred (high confidence)	AZOBR_10049	Ancestral (high confidence)
AZOLI_0055	Ancestral (low confidence)	AZOBR_10050	Unassigned
AZOLI_0056	Ancestral (medium confidence)	AZOBR_10051	Ancestral (low confidence)
AZOLI_0057	Ancestral (high confidence)	AZOBR_10052	Ancestral (high confidence)
AZOLI_0058	Ancestral (high confidence)	AZOBR_10053	Ancestral (high confidence)
AZOLI_0059	Ancestral (medium confidence)	AZOBR_10054	Ancestral (medium confidence)
AZOLI_0060	Ancestral (medium confidence)	AZOBR_10055	Ancestral (high confidence)
AZOLI_0061	Horizontally Transferred (high confidence)	AZOBR_10056	Ancestral (medium confidence)
AZOLI_0062	Ancestral (high confidence)	AZOBR_10057	Ancestral (low confidence)
AZOLI_0063	Ancestral (high confidence)	AZOBR_10058	Ancestral (high confidence)
AZOLI_0064	Ancestral (high confidence)	AZOBR_10059	Ancestral (low confidence)
AZOLI_0066	Ancestral (high confidence)	AZOBR_10060	Ancestral (medium confidence)
AZOLI_0067	Ancestral (high confidence)	AZOBR_10061	Ancestral (medium confidence)
AZOLI_0068	Ancestral (medium confidence)	AZOBR_10062	Ancestral (high confidence)
AZOLI_0069	Ancestral (high confidence)	AZOBR_10063	Ancestral (low confidence)
AZOLI_0070	Horizontally Transferred (high confidence)	AZOBR_10064	Horizontally Transferred (high confidence)
AZOLI_0071	Horizontally Transferred (high confidence)	AZOBR_10065	Ancestral (high confidence)
AZOLI_0072	Ancestral (low confidence)	AZOBR_10066	Unassigned
AZOLI_0073	Horizontally Transferred (low confidence)	AZOBR_10067	Unassigned
AZOLI_0074	Ancestral (low confidence)	AZOBR_10068	Unassigned

AZOLI_0075	Ancestral (high confidence)	AZOBR_10069	Unassigned
AZOLI_0077	Ancestral (high confidence)	AZOBR_10070	Horizontally Transferred (high confidence)
AZOLI_0078	Ancestral (high confidence)	AZOBR_10071	Horizontally Transferred (high confidence)
AZOLI_0079	Ancestral (medium confidence)	AZOBR_10072	Ancestral (low confidence)
AZOLI_0080	Horizontally Transferred (high confidence)	AZOBR_10073	Horizontally Transferred (high confidence)
AZOLI_0081	Ancestral (high confidence)	AZOBR_10074	Ancestral (medium confidence)
AZOLI_0082	Ancestral (medium confidence)	AZOBR_10075	Ancestral (low confidence)
AZOLI_0083	Ancestral (medium confidence)	AZOBR_10076	Ancestral (medium confidence)
AZOLI_0084	Ancestral (medium confidence)	AZOBR_10077	Horizontally Transferred (high confidence)
AZOLI_0085	Ancestral (medium confidence)	AZOBR_10078	Unassigned
AZOLI_0086	Ancestral (medium confidence)	AZOBR_10079	Ancestral (medium confidence)
AZOLI_0087	Ancestral (medium confidence)	AZOBR_10080	Horizontally Transferred (high confidence)
AZOLI_0088	Ancestral (high confidence)	AZOBR_10081	Horizontally Transferred (high confidence)
AZOLI_0089	Ancestral (medium confidence)	AZOBR_10082	Horizontally Transferred (high confidence)
AZOLI_0090	Ancestral (medium confidence)	AZOBR_10083	Horizontally Transferred (high confidence)
AZOLI_0091	Ancestral (high confidence)	AZOBR_10084	Horizontally Transferred (high confidence)
AZOLI_0092	Ancestral (medium confidence)	AZOBR_10085	Unassigned
AZOLI_0093	Horizontally Transferred (low confidence)	AZOBR_10086	Unassigned
AZOLI_0094	Ancestral (low confidence)	AZOBR_10087	Unassigned
AZOLI_0095	Ancestral (high confidence)	AZOBR_10088	Ancestral (medium confidence)
AZOLI_0096	Ancestral (high confidence)	AZOBR_10089	Ancestral (high confidence)
AZOLI_0097	Horizontally Transferred (high confidence)	AZOBR_10090	Ancestral (high confidence)
AZOLI_0098	Unassigned	AZOBR_10091	Horizontally Transferred (medium confidence)
AZOLI_0101	Unassigned	AZOBR_10092	Unassigned
AZOLI_0104	Unassigned	AZOBR_10093	Horizontally Transferred (high confidence)
AZOLI_0105	Unassigned	AZOBR_10094	Ancestral (medium confidence)
AZOLI_0106	Unassigned	AZOBR_10095	Horizontally Transferred (high confidence)
AZOLI_0107	Ancestral (high confidence)	AZOBR_10096	Ancestral (medium confidence)
AZOLI_0108	Ancestral (high confidence)	AZOBR_10097	Ancestral (low confidence)
AZOLI_0109	Ancestral (high confidence)	AZOBR_10098	Unassigned
AZOLI_0110	Unassigned	AZOBR_10099	Horizontally Transferred (high confidence)
AZOLI_0111	Ancestral (low confidence)	AZOBR_10100	Horizontally Transferred (high confidence)
AZOLI_0112	Horizontally Transferred (high confidence)	AZOBR_10101	Horizontally Transferred (high confidence)
AZOLI_0113	Horizontally Transferred (high confidence)	AZOBR_10102	Ancestral (medium confidence)
AZOLI_0114	Horizontally Transferred (high confidence)	AZOBR_10103	Ancestral (high confidence)
AZOLI_0115	Horizontally Transferred (high confidence)	AZOBR_10104	Ancestral (medium confidence)
AZOLI_0116	Horizontally Transferred (high confidence)	AZOBR_10105	Ancestral (medium confidence)
AZOLI_0117	Horizontally Transferred (high confidence)	AZOBR_10106	Unassigned
AZOLI_0118	Ancestral (high confidence)	AZOBR_10107	Ancestral (medium confidence)
AZOLI_0119	Horizontally Transferred (high confidence)	AZOBR_10108	Ancestral (high confidence)
AZOLI_0120	Horizontally Transferred (high confidence)	AZOBR_10109	Ancestral (high confidence)
AZOLI_0121	Ancestral (low confidence)	AZOBR_10110	Horizontally Transferred (high confidence)
AZOLI_0122	Ancestral (medium confidence)	AZOBR_10111	Ancestral (medium confidence)
AZOLI_0123	Unassigned	AZOBR_10112	Ancestral (medium confidence)
AZOLI_0124	Ancestral (medium confidence)	AZOBR_10113	Ancestral (medium confidence)
AZOLI_0125	Horizontally Transferred (high confidence)	AZOBR_10114	Ancestral (high confidence)
AZOLI_0126	Horizontally Transferred (high confidence)	AZOBR_10115	Ancestral (medium confidence)
AZOLI_0129	Unassigned	AZOBR_10116	Ancestral (high confidence)
AZOLI_0130	Horizontally Transferred (high confidence)	AZOBR_10117	Ancestral (high confidence)
AZOLI_0131	Unassigned	AZOBR_10118	Ancestral (medium confidence)
AZOLI_0132	Ancestral (medium confidence)	AZOBR_10119	Ancestral (high confidence)
AZOLI_0133	Ancestral (medium confidence)	AZOBR_10120	Ancestral (high confidence)
AZOLI_0134	Ancestral (low confidence)	AZOBR_10121	Ancestral (high confidence)
AZOLI_0135	Ancestral (medium confidence)	AZOBR_10122	Ancestral (high confidence)
AZOLI_0136	Ancestral (medium confidence)	AZOBR_10123	Ancestral (high confidence)
AZOLI_0137	Ancestral (high confidence)	AZOBR_10124	Ancestral (high confidence)
AZOLI_0138	Ancestral (low confidence)	AZOBR_10125	Ancestral (medium confidence)
AZOLI_0139	Horizontally Transferred (high confidence)	AZOBR_10126	Unassigned
AZOLI_0140	Ancestral (high confidence)	AZOBR_10127	Ancestral (medium confidence)
AZOLI_0141	Ancestral (high confidence)	AZOBR_10128	Ancestral (high confidence)
AZOLI_0142	Ancestral (medium confidence)	AZOBR_10129	Ancestral (low confidence)
AZOLI_0143	Horizontally Transferred (high confidence)	AZOBR_10130	Ancestral (high confidence)
AZOLI_0144	Ancestral (low confidence)	AZOBR_10131	Unassigned
AZOLI_0145	Ancestral (low confidence)	AZOBR_10132	Horizontally Transferred (high confidence)
AZOLI_0146	Unassigned	AZOBR_10133	Ancestral (medium confidence)
AZOLI_0147	Ancestral (low confidence)	AZOBR_10134	Horizontally Transferred (high confidence)
AZOLI_0148	Ancestral (medium confidence)	AZOBR_10135	Ancestral (high confidence)
AZOLI_0149	Ancestral (medium confidence)	AZOBR_10136	Ancestral (medium confidence)
AZOLI_0150	Ancestral (low confidence)	AZOBR_10137	Unassigned
AZOLI_0151	Unassigned	AZOBR_10138	Ancestral (low confidence)
AZOLI_0152	Ancestral (low confidence)	AZOBR_10139	Ancestral (medium confidence)
AZOLI_0153	Ancestral (low confidence)	AZOBR_10140	Ancestral (medium confidence)
AZOLI_0154	Unassigned	AZOBR_10141	Ancestral (medium confidence)
AZOLI_0155	Ancestral (high confidence)	AZOBR_10142	Ancestral (medium confidence)
AZOLI_0156	Ancestral (medium confidence)	AZOBR_10143	Horizontally Transferred (high confidence)

AZOLI_0157	Horizontally Transferred (low confidence)	AZOBR_10144	Horizontally Transferred (high confidence)
AZOLI_0158	Horizontally Transferred (high confidence)	AZOBR_10145	Unassigned
AZOLI_0159	Ancestral (medium confidence)	AZOBR_10146	Ancestral (high confidence)
AZOLI_0160	Ancestral (medium confidence)	AZOBR_10147	Ancestral (medium confidence)
AZOLI_0161	Ancestral (low confidence)	AZOBR_10148	Horizontally Transferred (high confidence)
AZOLI_0162	Ancestral (low confidence)	AZOBR_10149	Unassigned
AZOLI_0163	Horizontally Transferred (high confidence)	AZOBR_10150	Unassigned
AZOLI_0164	Ancestral (high confidence)	AZOBR_10151	Ancestral (high confidence)
AZOLI_0165	Ancestral (medium confidence)	AZOBR_10152	Ancestral (high confidence)
AZOLI_0166	Ancestral (high confidence)	AZOBR_10153	Ancestral (high confidence)
AZOLI_0167	Horizontally Transferred (high confidence)	AZOBR_10154	Ancestral (high confidence)
AZOLI_0168	Horizontally Transferred (high confidence)	AZOBR_10155	Ancestral (medium confidence)
AZOLI_0170	Ancestral (high confidence)	AZOBR_10156	Ancestral (medium confidence)
AZOLI_0172	Ancestral (high confidence)	AZOBR_10157	Ancestral (medium confidence)
AZOLI_0173	Ancestral (high confidence)	AZOBR_10158	Ancestral (low confidence)
AZOLI_0174	Ancestral (high confidence)	AZOBR_10159	Ancestral (low confidence)
AZOLI_0176	Ancestral (low confidence)	AZOBR_10160	Ancestral (low confidence)
AZOLI_0177	Unassigned	AZOBR_10161	Horizontally Transferred (high confidence)
AZOLI_0178	Ancestral (high confidence)	AZOBR_10162	Ancestral (high confidence)
AZOLI_0179	Horizontally Transferred (high confidence)	AZOBR_10163	Ancestral (high confidence)
AZOLI_0181	Ancestral (high confidence)	AZOBR_10164	Horizontally Transferred (high confidence)
AZOLI_0182	Ancestral (low confidence)	AZOBR_10165	Horizontally Transferred (high confidence)
AZOLI_0183	Unassigned	AZOBR_10166	Ancestral (high confidence)
AZOLI_0184	Unassigned	AZOBR_10167	Ancestral (medium confidence)
AZOLI_0186	Unassigned	AZOBR_10168	Ancestral (high confidence)
AZOLI_0188	Unassigned	AZOBR_10169	Ancestral (high confidence)
AZOLI_0190	Ancestral (medium confidence)	AZOBR_10170	Unassigned
AZOLI_0191	Ancestral (low confidence)	AZOBR_10171	Ancestral (low confidence)
AZOLI_0192	Ancestral (medium confidence)	AZOBR_10172	Ancestral (medium confidence)
AZOLI_0193	Ancestral (medium confidence)	AZOBR_10173	Ancestral (low confidence)
AZOLI_0194	Ancestral (medium confidence)	AZOBR_10174	Horizontally Transferred (high confidence)
AZOLI_0195	Ancestral (medium confidence)	AZOBR_10175	Ancestral (low confidence)
AZOLI_0196	Ancestral (medium confidence)	AZOBR_10176	Ancestral (medium confidence)
AZOLI_0197	Horizontally Transferred (high confidence)	AZOBR_10177	Unassigned
AZOLI_0198	Unassigned	AZOBR_10178	Ancestral (medium confidence)
AZOLI_0199	Ancestral (medium confidence)	AZOBR_10179	Ancestral (medium confidence)
AZOLI_0200	Horizontally Transferred (high confidence)	AZOBR_10180	Ancestral (medium confidence)
AZOLI_0201	Unassigned	AZOBR_10181	Horizontally Transferred (high confidence)
AZOLI_0202	Horizontally Transferred (high confidence)	AZOBR_10182	Unassigned
AZOLI_0203	Ancestral (low confidence)	AZOBR_10183	Horizontally Transferred (high confidence)
AZOLI_0204	Ancestral (high confidence)	AZOBR_10184	Horizontally Transferred (high confidence)
AZOLI_0205	Ancestral (high confidence)	AZOBR_10185	Horizontally Transferred (high confidence)
AZOLI_0206	Ancestral (high confidence)	AZOBR_10186	Ancestral (medium confidence)
AZOLI_0207	Ancestral (low confidence)	AZOBR_10187	Unassigned
AZOLI_0208	Horizontally Transferred (high confidence)	AZOBR_10188	Unassigned
AZOLI_0209	Ancestral (high confidence)	AZOBR_10189	Ancestral (high confidence)
AZOLI_0210	Horizontally Transferred (high confidence)	AZOBR_10190	Ancestral (high confidence)
AZOLI_0211	Unassigned	AZOBR_10191	Ancestral (high confidence)
AZOLI_0212	Ancestral (high confidence)	AZOBR_10192	Ancestral (high confidence)
AZOLI_0213	Ancestral (low confidence)	AZOBR_10193	Unassigned
AZOLI_0214	Horizontally Transferred (high confidence)	AZOBR_10194	Ancestral (low confidence)
AZOLI_0215	Horizontally Transferred (high confidence)	AZOBR_10195	Ancestral (medium confidence)
AZOLI_0216	Ancestral (low confidence)	AZOBR_10196	Ancestral (medium confidence)
AZOLI_0217	Ancestral (low confidence)	AZOBR_10197	Ancestral (medium confidence)
AZOLI_0218	Ancestral (high confidence)	AZOBR_10198	Ancestral (low confidence)
AZOLI_0219	Ancestral (high confidence)	AZOBR_10199	Unassigned
AZOLI_0220	Ancestral (high confidence)	AZOBR_10200	Unassigned
AZOLI_0221	Ancestral (medium confidence)	AZOBR_10201	Ancestral (high confidence)
AZOLI_0222	Ancestral (low confidence)	AZOBR_10202	Ancestral (high confidence)
AZOLI_0223	Unassigned	AZOBR_10203	Unassigned
AZOLI_0224	Unassigned	AZOBR_10204	Ancestral (medium confidence)
AZOLI_0225	Unassigned	AZOBR_10205	Unassigned
AZOLI_0226	Ancestral (medium confidence)	AZOBR_10206	Horizontally Transferred (high confidence)
AZOLI_0227	Ancestral (high confidence)	AZOBR_10207	Horizontally Transferred (high confidence)
AZOLI_0229	Ancestral (high confidence)	AZOBR_10208	Ancestral (low confidence)
AZOLI_0230	Ancestral (medium confidence)	AZOBR_10209	Ancestral (medium confidence)
AZOLI_0231	Ancestral (high confidence)	AZOBR_10210	Horizontally Transferred (high confidence)
AZOLI_0232	Horizontally Transferred (high confidence)	AZOBR_10211	Ancestral (medium confidence)
AZOLI_0233	Ancestral (high confidence)	AZOBR_10212	Ancestral (high confidence)
AZOLI_0234	Ancestral (medium confidence)	AZOBR_10213	Horizontally Transferred (high confidence)
AZOLI_0235	Ancestral (medium confidence)	AZOBR_10214	Ancestral (medium confidence)
AZOLI_0236	Ancestral (high confidence)	AZOBR_10215	Ancestral (high confidence)
AZOLI_0237	Ancestral (low confidence)	AZOBR_10216	Ancestral (high confidence)
AZOLI_0238	Ancestral (low confidence)	AZOBR_10217	Ancestral (low confidence)
AZOLI_0239	Unassigned	AZOBR_10218	Ancestral (high confidence)

AZOLI_0240	Horizontally Transferred (high confidence)	AZOBR_10219	Unassigned
AZOLI_0241	Ancestral (low confidence)	AZOBR_10220	Horizontally Transferred (high confidence)
AZOLI_0242	Ancestral (low confidence)	AZOBR_10221	Ancestral (low confidence)
AZOLI_0243	Ancestral (low confidence)	AZOBR_10222	Horizontally Transferred (high confidence)
AZOLI_0244	Ancestral (low confidence)	AZOBR_10223	Ancestral (high confidence)
AZOLI_0245	Unassigned	AZOBR_10224	Ancestral (low confidence)
AZOLI_0246	Horizontally Transferred (high confidence)	AZOBR_10225	Ancestral (low confidence)
AZOLI_0247	Horizontally Transferred (high confidence)	AZOBR_10226	Ancestral (low confidence)
AZOLI_0248	Horizontally Transferred (high confidence)	AZOBR_10227	Ancestral (low confidence)
AZOLI_0249	Unassigned	AZOBR_10228	Ancestral (medium confidence)
AZOLI_0251	Horizontally Transferred (high confidence)	AZOBR_10229	Ancestral (medium confidence)
AZOLI_0252	Ancestral (medium confidence)	AZOBR_10230	Unassigned
AZOLI_0253	Ancestral (high confidence)	AZOBR_10231	Ancestral (low confidence)
AZOLI_0254	Ancestral (high confidence)	AZOBR_10232	Unassigned
AZOLI_0255	Ancestral (medium confidence)	AZOBR_10233	Ancestral (high confidence)
AZOLI_0256	Ancestral (high confidence)	AZOBR_10234	Ancestral (low confidence)
AZOLI_0257	Ancestral (medium confidence)	AZOBR_10235	Ancestral (high confidence)
AZOLI_0258	Horizontally Transferred (high confidence)	AZOBR_10236	Ancestral (high confidence)
AZOLI_0259	Ancestral (low confidence)	AZOBR_10237	Horizontally Transferred (high confidence)
AZOLI_0260	Ancestral (low confidence)	AZOBR_10238	Ancestral (medium confidence)
AZOLI_0261	Ancestral (low confidence)	AZOBR_10239	Ancestral (high confidence)
AZOLI_0262	Ancestral (low confidence)	AZOBR_10240	Horizontally Transferred (high confidence)
AZOLI_0263	Ancestral (medium confidence)	AZOBR_10241	Ancestral (high confidence)
AZOLI_0264	Ancestral (medium confidence)	AZOBR_10242	Ancestral (high confidence)
AZOLI_0265	Ancestral (medium confidence)	AZOBR_10243	Ancestral (medium confidence)
AZOLI_0266	Ancestral (low confidence)	AZOBR_10244	Ancestral (medium confidence)
AZOLI_0268	Horizontally Transferred (high confidence)	AZOBR_10245	Ancestral (high confidence)
AZOLI_0269	Ancestral (high confidence)	AZOBR_10246	Ancestral (high confidence)
AZOLI_0270	Ancestral (medium confidence)	AZOBR_10247	Ancestral (high confidence)
AZOLI_0271	Ancestral (medium confidence)	AZOBR_10248	Horizontally Transferred (high confidence)
AZOLI_0272	Ancestral (high confidence)	AZOBR_10249	Ancestral (high confidence)
AZOLI_0274	Ancestral (high confidence)	AZOBR_10250	Unassigned
AZOLI_0275	Ancestral (medium confidence)	AZOBR_10251	Ancestral (high confidence)
AZOLI_0276	Ancestral (high confidence)	AZOBR_10252	Ancestral (medium confidence)
AZOLI_0277	Ancestral (high confidence)	AZOBR_10253	Ancestral (high confidence)
AZOLI_0278	Unassigned	AZOBR_10254	Ancestral (high confidence)
AZOLI_0279	Ancestral (high confidence)	AZOBR_10255	Ancestral (low confidence)
AZOLI_0280	Horizontally Transferred (high confidence)	AZOBR_10256	Ancestral (medium confidence)
AZOLI_0281	Ancestral (high confidence)	AZOBR_10257	Horizontally Transferred (high confidence)
AZOLI_0282	Ancestral (medium confidence)	AZOBR_10258	Ancestral (high confidence)
AZOLI_0283	Horizontally Transferred (medium confidence)	AZOBR_10259	Ancestral (high confidence)
AZOLI_0284	Unassigned	AZOBR_10260	Unassigned
AZOLI_0285	Ancestral (medium confidence)	AZOBR_10261	Ancestral (high confidence)
AZOLI_0286	Unassigned	AZOBR_10262	Ancestral (high confidence)
AZOLI_0287	Horizontally Transferred (high confidence)	AZOBR_10263	Ancestral (medium confidence)
AZOLI_0288	Ancestral (high confidence)	AZOBR_10264	Ancestral (low confidence)
AZOLI_0290	Ancestral (medium confidence)	AZOBR_10265	Unassigned
AZOLI_0292	Horizontally Transferred (medium confidence)	AZOBR_10266	Ancestral (high confidence)
AZOLI_0293	Horizontally Transferred (high confidence)	AZOBR_10267	Ancestral (medium confidence)
AZOLI_0294	Ancestral (high confidence)	AZOBR_10268	Unassigned
AZOLI_0295	Ancestral (high confidence)	AZOBR_10269	Ancestral (low confidence)
AZOLI_0296	Ancestral (medium confidence)	AZOBR_10270	Ancestral (low confidence)
AZOLI_0297	Ancestral (high confidence)	AZOBR_10271	Unassigned
AZOLI_0298	Ancestral (high confidence)	AZOBR_10272	Unassigned
AZOLI_0299	Ancestral (medium confidence)	AZOBR_10273	Ancestral (low confidence)
AZOLI_0301	Ancestral (high confidence)	AZOBR_10274	Horizontally Transferred (high confidence)
AZOLI_0302	Unassigned	AZOBR_10275	Ancestral (low confidence)
AZOLI_0303	Ancestral (medium confidence)	AZOBR_10276	Ancestral (low confidence)
AZOLI_0304	Unassigned	AZOBR_10277	Ancestral (high confidence)
AZOLI_0305	Horizontally Transferred (high confidence)	AZOBR_10278	Unassigned
AZOLI_0306	Horizontally Transferred (high confidence)	AZOBR_10279	Ancestral (medium confidence)
AZOLI_0307	Unassigned	AZOBR_10280	Ancestral (high confidence)
AZOLI_0308	Ancestral (medium confidence)	AZOBR_10281	Ancestral (high confidence)
AZOLI_0309	Ancestral (high confidence)	AZOBR_10282	Ancestral (high confidence)
AZOLI_0311	Horizontally Transferred (high confidence)	AZOBR_10283	Ancestral (low confidence)
AZOLI_0312	Horizontally Transferred (high confidence)	AZOBR_10284	Ancestral (medium confidence)
AZOLI_0313	Ancestral (medium confidence)	AZOBR_10285	Ancestral (high confidence)
AZOLI_0314	Ancestral (high confidence)	AZOBR_10286	Ancestral (high confidence)
AZOLI_0315	Ancestral (high confidence)	AZOBR_10287	Ancestral (low confidence)
AZOLI_0316	Horizontally Transferred (high confidence)	AZOBR_10288	Ancestral (medium confidence)
AZOLI_0317	Ancestral (medium confidence)	AZOBR_10289	Ancestral (high confidence)
AZOLI_0318	Ancestral (medium confidence)	AZOBR_10290	Ancestral (low confidence)
AZOLI_0319	Ancestral (low confidence)	AZOBR_10291	Ancestral (medium confidence)
AZOLI_0320	Ancestral (low confidence)	AZOBR_10292	Ancestral (low confidence)
AZOLI_0321	Ancestral (low confidence)	AZOBR_10293	Ancestral (high confidence)

AZOLI_0322	Ancestral (low confidence)	AZOBR_10294	Ancestral (high confidence)
AZOLI_0323	Unassigned	AZOBR_10295	Ancestral (low confidence)
AZOLI_0324	Horizontally Transferred (high confidence)	AZOBR_10296	Ancestral (low confidence)
AZOLI_0325	Unassigned	AZOBR_10297	Ancestral (high confidence)
AZOLI_0326	Horizontally Transferred (high confidence)	AZOBR_10298	Ancestral (high confidence)
AZOLI_0327	Ancestral (high confidence)	AZOBR_10299	Unassigned
AZOLI_0328	Ancestral (medium confidence)	AZOBR_10300	Ancestral (high confidence)
AZOLI_0329	Ancestral (medium confidence)	AZOBR_10301	Ancestral (low confidence)
AZOLI_0339	Horizontally Transferred (high confidence)	AZOBR_10302	Horizontally Transferred (high confidence)
AZOLI_0340	Ancestral (high confidence)	AZOBR_10303	Horizontally Transferred (high confidence)
AZOLI_0341	Horizontally Transferred (high confidence)	AZOBR_10304	Ancestral (medium confidence)
AZOLI_0342	Ancestral (low confidence)	AZOBR_10305	Horizontally Transferred (high confidence)
AZOLI_0344	Ancestral (low confidence)	AZOBR_10306	Ancestral (medium confidence)
AZOLI_0345	Horizontally Transferred (high confidence)	AZOBR_10307	Unassigned
AZOLI_0346	Horizontally Transferred (high confidence)	AZOBR_10308	Ancestral (high confidence)
AZOLI_0347	Horizontally Transferred (high confidence)	AZOBR_10309	Ancestral (high confidence)
AZOLI_0348	Ancestral (medium confidence)	AZOBR_10310	Ancestral (high confidence)
AZOLI_0349	Ancestral (medium confidence)	AZOBR_10311	Ancestral (high confidence)
AZOLI_0350	Ancestral (medium confidence)	AZOBR_10312	Ancestral (high confidence)
AZOLI_0351	Ancestral (medium confidence)	AZOBR_10313	Ancestral (high confidence)
AZOLI_0352	Ancestral (low confidence)	AZOBR_10314	Ancestral (high confidence)
AZOLI_0353	Horizontally Transferred (low confidence)	AZOBR_10315	Horizontally Transferred (high confidence)
AZOLI_0354	Ancestral (medium confidence)	AZOBR_10316	Unassigned
AZOLI_0355	Horizontally Transferred (medium confidence)	AZOBR_10317	Unassigned
AZOLI_0356	Horizontally Transferred (medium confidence)	AZOBR_10318	Unassigned
AZOLI_0357	Ancestral (medium confidence)	AZOBR_10319	Ancestral (high confidence)
AZOLI_0358	Ancestral (high confidence)	AZOBR_10320	Ancestral (high confidence)
AZOLI_0359	Ancestral (high confidence)	AZOBR_10321	Unassigned
AZOLI_0360	Ancestral (high confidence)	AZOBR_10322	Ancestral (high confidence)
AZOLI_0361	Ancestral (high confidence)	AZOBR_10323	Ancestral (high confidence)
AZOLI_0362	Ancestral (high confidence)	AZOBR_10324	Ancestral (high confidence)
AZOLI_0363	Ancestral (high confidence)	AZOBR_10325	Ancestral (high confidence)
AZOLI_0365	Ancestral (high confidence)	AZOBR_10326	Ancestral (medium confidence)
AZOLI_0366	Ancestral (high confidence)	AZOBR_10327	Ancestral (high confidence)
AZOLI_0367	Horizontally Transferred (high confidence)	AZOBR_10328	Unassigned
AZOLI_0368	Ancestral (medium confidence)	AZOBR_10329	Ancestral (high confidence)
AZOLI_0369	Ancestral (medium confidence)	AZOBR_10330	Ancestral (high confidence)
AZOLI_0370	Ancestral (high confidence)	AZOBR_10331	Ancestral (high confidence)
AZOLI_0371	Ancestral (medium confidence)	AZOBR_10332	Unassigned
AZOLI_0372	Ancestral (medium confidence)	AZOBR_10333	Ancestral (high confidence)
AZOLI_0373	Ancestral (high confidence)	AZOBR_10334	Unassigned
AZOLI_0374	Ancestral (high confidence)	AZOBR_10335	Ancestral (medium confidence)
AZOLI_0375	Ancestral (low confidence)	AZOBR_10336	Ancestral (high confidence)
AZOLI_0376	Ancestral (medium confidence)	AZOBR_10337	Ancestral (high confidence)
AZOLI_0377	Horizontally Transferred (high confidence)	AZOBR_10338	Ancestral (medium confidence)
AZOLI_0378	Ancestral (high confidence)	AZOBR_10339	Ancestral (low confidence)
AZOLI_0379	Ancestral (medium confidence)	AZOBR_10340	Horizontally Transferred (high confidence)
AZOLI_0380	Ancestral (low confidence)	AZOBR_10341	Ancestral (high confidence)
AZOLI_0381	Ancestral (high confidence)	AZOBR_10342	Ancestral (high confidence)
AZOLI_0382	Ancestral (medium confidence)	AZOBR_10343	Ancestral (medium confidence)
AZOLI_0383	Horizontally Transferred (high confidence)	AZOBR_10344	Ancestral (high confidence)
AZOLI_0384	Ancestral (medium confidence)	AZOBR_10345	Ancestral (medium confidence)
AZOLI_0385	Ancestral (medium confidence)	AZOBR_10346	Ancestral (medium confidence)
AZOLI_0386	Ancestral (medium confidence)	AZOBR_10347	Ancestral (medium confidence)
AZOLI_0387	Ancestral (high confidence)	AZOBR_10348	Ancestral (medium confidence)
AZOLI_0388	Ancestral (high confidence)	AZOBR_10349	Horizontally Transferred (medium confidence)
AZOLI_0389	Horizontally Transferred (high confidence)	AZOBR_10350	Ancestral (high confidence)
AZOLI_0390	Horizontally Transferred (high confidence)	AZOBR_10351	Unassigned
AZOLI_0391	Unassigned	AZOBR_10352	Ancestral (low confidence)
AZOLI_0392	Ancestral (high confidence)	AZOBR_10353	Horizontally Transferred (high confidence)
AZOLI_0393	Unassigned	AZOBR_10354	Horizontally Transferred (high confidence)
AZOLI_0394	Horizontally Transferred (high confidence)	AZOBR_10355	Ancestral (low confidence)
AZOLI_0395	Ancestral (low confidence)	AZOBR_10356	Ancestral (medium confidence)
AZOLI_0397	Horizontally Transferred (high confidence)	AZOBR_10357	Ancestral (low confidence)
AZOLI_0398	Unassigned	AZOBR_10358	Ancestral (low confidence)
AZOLI_0399	Ancestral (low confidence)	AZOBR_10359	Ancestral (low confidence)
AZOLI_0400	Horizontally Transferred (high confidence)	AZOBR_10360	Unassigned
AZOLI_0402	Ancestral (medium confidence)	AZOBR_10361	Ancestral (high confidence)
AZOLI_0403	Horizontally Transferred (high confidence)	AZOBR_10362	Ancestral (high confidence)
AZOLI_0405	Ancestral (low confidence)	AZOBR_10363	Ancestral (medium confidence)
AZOLI_0406	Ancestral (low confidence)	AZOBR_10364	Unassigned
AZOLI_0407	Ancestral (low confidence)	AZOBR_10365	Unassigned
AZOLI_0408	Horizontally Transferred (high confidence)	AZOBR_10366	Ancestral (high confidence)
AZOLI_0410	Ancestral (low confidence)	AZOBR_10367	Ancestral (medium confidence)
AZOLI_0411	Ancestral (low confidence)	AZOBR_10368	Ancestral (medium confidence)

AZOLI_0412	Ancestral (low confidence)	AZOBR_10369	Ancestral (high confidence)
AZOLI_0413	Ancestral (low confidence)	AZOBR_10370	Horizontally Transferred (high confidence)
AZOLI_0414	Ancestral (low confidence)	AZOBR_10371	Ancestral (medium confidence)
AZOLI_0415	Ancestral (low confidence)	AZOBR_10372	Ancestral (medium confidence)
AZOLI_0416	Ancestral (low confidence)	AZOBR_10373	Ancestral (high confidence)
AZOLI_0417	Ancestral (medium confidence)	AZOBR_10374	Ancestral (high confidence)
AZOLI_0419	Unassigned	AZOBR_10375	Ancestral (high confidence)
AZOLI_0421	Ancestral (medium confidence)	AZOBR_10376	Ancestral (low confidence)
AZOLI_0422	Ancestral (medium confidence)	AZOBR_10377	Ancestral (high confidence)
AZOLI_0423	Horizontally Transferred (high confidence)	AZOBR_10378	Ancestral (high confidence)
AZOLI_0424	Unassigned	AZOBR_10379	Unassigned
AZOLI_0425	Unassigned	AZOBR_10380	Ancestral (low confidence)
AZOLI_0427	Unassigned	AZOBR_10381	Ancestral (medium confidence)
AZOLI_0430	Ancestral (low confidence)	AZOBR_10382	Ancestral (high confidence)
AZOLI_0432	Ancestral (medium confidence)	AZOBR_10383	Horizontally Transferred (high confidence)
AZOLI_0433	Ancestral (low confidence)	AZOBR_10384	Horizontally Transferred (high confidence)
AZOLI_0434	Horizontally Transferred (low confidence)	AZOBR_10385	Horizontally Transferred (high confidence)
AZOLI_0435	Unassigned	AZOBR_10386	Horizontally Transferred (high confidence)
AZOLI_0436	Ancestral (high confidence)	AZOBR_10387	Ancestral (medium confidence)
AZOLI_0439	Ancestral (medium confidence)	AZOBR_10388	Ancestral (high confidence)
AZOLI_0442	Ancestral (high confidence)	AZOBR_10389	Unassigned
AZOLI_0443	Ancestral (high confidence)	AZOBR_10390	Ancestral (medium confidence)
AZOLI_0445	Ancestral (high confidence)	AZOBR_10391	Ancestral (high confidence)
AZOLI_0446	Ancestral (medium confidence)	AZOBR_10392	Ancestral (high confidence)
AZOLI_0447	Unassigned	AZOBR_10393	Ancestral (high confidence)
AZOLI_0448	Ancestral (high confidence)	AZOBR_10394	Ancestral (high confidence)
AZOLI_0449	Ancestral (low confidence)	AZOBR_10395	Ancestral (medium confidence)
AZOLI_0450	Ancestral (high confidence)	AZOBR_10396	Ancestral (medium confidence)
AZOLI_0451	Ancestral (medium confidence)	AZOBR_10397	Ancestral (high confidence)
AZOLI_0454	Unassigned	AZOBR_10398	Ancestral (high confidence)
AZOLI_0455	Ancestral (high confidence)	AZOBR_10399	Ancestral (high confidence)
AZOLI_0456	Ancestral (high confidence)	AZOBR_10400	Ancestral (high confidence)
AZOLI_0457	Ancestral (high confidence)	AZOBR_10401	Ancestral (high confidence)
AZOLI_0458	Ancestral (medium confidence)	AZOBR_10402	Ancestral (high confidence)
AZOLI_0459	Unassigned	AZOBR_10403	Ancestral (high confidence)
AZOLI_0460	Ancestral (high confidence)	AZOBR_10404	Ancestral (high confidence)
AZOLI_0461	Ancestral (high confidence)	AZOBR_10405	Ancestral (low confidence)
AZOLI_0462	Ancestral (high confidence)	AZOBR_10406	Ancestral (high confidence)
AZOLI_0463	Ancestral (high confidence)	AZOBR_10407	Ancestral (high confidence)
AZOLI_0464	Ancestral (high confidence)	AZOBR_10408	Ancestral (high confidence)
AZOLI_0465	Ancestral (high confidence)	AZOBR_10409	Ancestral (high confidence)
AZOLI_0466	Ancestral (medium confidence)	AZOBR_10410	Ancestral (high confidence)
AZOLI_0467	Ancestral (high confidence)	AZOBR_10411	Unassigned
AZOLI_0468	Ancestral (medium confidence)	AZOBR_10412	Unassigned
AZOLI_0469	Ancestral (high confidence)	AZOBR_10413	Ancestral (high confidence)
AZOLI_0470	Ancestral (medium confidence)	AZOBR_10414	Horizontally Transferred (high confidence)
AZOLI_0471	Ancestral (high confidence)	AZOBR_10415	Ancestral (high confidence)
AZOLI_0472	Ancestral (medium confidence)	AZOBR_10416	Ancestral (medium confidence)
AZOLI_0473	Ancestral (high confidence)	AZOBR_10417	Ancestral (high confidence)
AZOLI_0474	Ancestral (low confidence)	AZOBR_10418	Ancestral (high confidence)
AZOLI_0475	Ancestral (medium confidence)	AZOBR_10419	Ancestral (medium confidence)
AZOLI_0476	Ancestral (high confidence)	AZOBR_10420	Ancestral (low confidence)
AZOLI_0477	Ancestral (high confidence)	AZOBR_10421	Horizontally Transferred (high confidence)
AZOLI_0478	Ancestral (high confidence)	AZOBR_10422	Horizontally Transferred (high confidence)
AZOLI_0479	Ancestral (high confidence)	AZOBR_10423	Horizontally Transferred (high confidence)
AZOLI_0480	Horizontally Transferred (high confidence)	AZOBR_10424	Unassigned
AZOLI_0481	Ancestral (medium confidence)	AZOBR_10425	Ancestral (medium confidence)
AZOLI_0482	Ancestral (low confidence)	AZOBR_10426	Ancestral (low confidence)
AZOLI_0483	Ancestral (medium confidence)	AZOBR_10427	Ancestral (low confidence)
AZOLI_0484	Ancestral (low confidence)	AZOBR_10428	Ancestral (low confidence)
AZOLI_0485	Ancestral (high confidence)	AZOBR_10429	Ancestral (low confidence)
AZOLI_0486	Ancestral (high confidence)	AZOBR_10430	Ancestral (low confidence)
AZOLI_0487	Ancestral (low confidence)	AZOBR_10431	Ancestral (medium confidence)
AZOLI_0488	Ancestral (low confidence)	AZOBR_10432	Horizontally Transferred (high confidence)
AZOLI_0489	Horizontally Transferred (high confidence)	AZOBR_10433	Ancestral (low confidence)
AZOLI_0490	Unassigned	AZOBR_10434	Unassigned
AZOLI_0491	Ancestral (medium confidence)	AZOBR_10435	Ancestral (low confidence)
AZOLI_0492	Horizontally Transferred (high confidence)	AZOBR_10436	Ancestral (low confidence)
AZOLI_0493	Unassigned	AZOBR_10437	Ancestral (low confidence)
AZOLI_0494	Horizontally Transferred (high confidence)	AZOBR_10438	Ancestral (high confidence)
AZOLI_0495	Horizontally Transferred (medium confidence)	AZOBR_10439	Ancestral (low confidence)
AZOLI_0496	Unassigned	AZOBR_10440	Ancestral (low confidence)
AZOLI_0497	Horizontally Transferred (high confidence)	AZOBR_10441	Ancestral (high confidence)
AZOLI_0498	Unassigned	AZOBR_10442	Horizontally Transferred (high confidence)
AZOLI_0499	Unassigned	AZOBR_10443	Unassigned

AZOLI_0501	Ancestral (high confidence)	AZOBR_10444	Ancestral (medium confidence)
AZOLI_0502	Ancestral (low confidence)	AZOBR_10445	Ancestral (low confidence)
AZOLI_0503	Horizontally Transferred (high confidence)	AZOBR_10446	Ancestral (low confidence)
AZOLI_0504	Ancestral (medium confidence)	AZOBR_10447	Ancestral (low confidence)
AZOLI_0505	Ancestral (high confidence)	AZOBR_10448	Horizontally Transferred (high confidence)
AZOLI_0506	Horizontally Transferred (high confidence)	AZOBR_10449	Ancestral (low confidence)
AZOLI_0507	Ancestral (high confidence)	AZOBR_10450	Horizontally Transferred (high confidence)
AZOLI_0508	Ancestral (medium confidence)	AZOBR_10451	Horizontally Transferred (high confidence)
AZOLI_0509	Ancestral (low confidence)	AZOBR_10452	Unassigned
AZOLI_0510	Ancestral (low confidence)	AZOBR_10453	Unassigned
AZOLI_0511	Unassigned	AZOBR_10454	Ancestral (low confidence)
AZOLI_0512	Ancestral (medium confidence)	AZOBR_10455	Ancestral (high confidence)
AZOLI_0513	Ancestral (low confidence)	AZOBR_10456	Ancestral (high confidence)
AZOLI_0514	Ancestral (medium confidence)	AZOBR_10457	Ancestral (low confidence)
AZOLI_0515	Ancestral (medium confidence)	AZOBR_10458	Ancestral (medium confidence)
AZOLI_0516	Ancestral (low confidence)	AZOBR_10459	Ancestral (high confidence)
AZOLI_0517	Ancestral (high confidence)	AZOBR_10460	Ancestral (medium confidence)
AZOLI_0518	Unassigned	AZOBR_10461	Unassigned
AZOLI_0519	Ancestral (medium confidence)	AZOBR_10462	Horizontally Transferred (high confidence)
AZOLI_0520	Ancestral (high confidence)	AZOBR_10463	Ancestral (low confidence)
AZOLI_0521	Horizontally Transferred (high confidence)	AZOBR_10464	Unassigned
AZOLI_0522	Ancestral (high confidence)	AZOBR_10465	Horizontally Transferred (high confidence)
AZOLI_0523	Ancestral (high confidence)	AZOBR_10466	Ancestral (high confidence)
AZOLI_0524	Ancestral (high confidence)	AZOBR_10467	Horizontally Transferred (high confidence)
AZOLI_0525	Ancestral (medium confidence)	AZOBR_10468	Ancestral (medium confidence)
AZOLI_0526	Horizontally Transferred (high confidence)	AZOBR_10469	Ancestral (high confidence)
AZOLI_0527	Horizontally Transferred (low confidence)	AZOBR_10470	Unassigned
AZOLI_0528	Ancestral (low confidence)	AZOBR_10471	Horizontally Transferred (high confidence)
AZOLI_0529	Ancestral (medium confidence)	AZOBR_10472	Horizontally Transferred (high confidence)
AZOLI_0530	Ancestral (high confidence)	AZOBR_10473	Ancestral (high confidence)
AZOLI_0531	Ancestral (high confidence)	AZOBR_10474	Ancestral (high confidence)
AZOLI_0532	Horizontally Transferred (high confidence)	AZOBR_10475	Ancestral (high confidence)
AZOLI_0533	Ancestral (low confidence)	AZOBR_10476	Ancestral (medium confidence)
AZOLI_0534	Unassigned	AZOBR_10477	Ancestral (medium confidence)
AZOLI_0535	Ancestral (medium confidence)	AZOBR_10478	Ancestral (medium confidence)
AZOLI_0536	Ancestral (medium confidence)	AZOBR_10479	Ancestral (high confidence)
AZOLI_0537	Ancestral (medium confidence)	AZOBR_10480	Horizontally Transferred (high confidence)
AZOLI_0538	Unassigned	AZOBR_10481	Unassigned
AZOLI_0539	Horizontally Transferred (medium confidence)	AZOBR_10482	Horizontally Transferred (high confidence)
AZOLI_0540	Horizontally Transferred (high confidence)	AZOBR_10483	Ancestral (medium confidence)
AZOLI_0541	Horizontally Transferred (medium confidence)	AZOBR_10484	Ancestral (medium confidence)
AZOLI_0542	Horizontally Transferred (high confidence)	AZOBR_10485	Ancestral (high confidence)
AZOLI_0543	Ancestral (high confidence)	AZOBR_10486	Ancestral (high confidence)
AZOLI_0544	Horizontally Transferred (low confidence)	AZOBR_10487	Ancestral (high confidence)
AZOLI_0545	Horizontally Transferred (medium confidence)	AZOBR_10488	Ancestral (high confidence)
AZOLI_0546	Ancestral (low confidence)	AZOBR_10489	Ancestral (low confidence)
AZOLI_0548	Horizontally Transferred (high confidence)	AZOBR_10490	Ancestral (high confidence)
AZOLI_0549	Ancestral (low confidence)	AZOBR_10491	Ancestral (medium confidence)
AZOLI_0550	Horizontally Transferred (high confidence)	AZOBR_10492	Horizontally Transferred (high confidence)
AZOLI_0551	Horizontally Transferred (high confidence)	AZOBR_10493	Ancestral (high confidence)
AZOLI_0552	Ancestral (high confidence)	AZOBR_10494	Ancestral (high confidence)
AZOLI_0553	Ancestral (high confidence)	AZOBR_10495	Ancestral (medium confidence)
AZOLI_0554	Ancestral (medium confidence)	AZOBR_10496	Ancestral (medium confidence)
AZOLI_0555	Ancestral (medium confidence)	AZOBR_10497	Ancestral (medium confidence)
AZOLI_0556	Ancestral (high confidence)	AZOBR_10498	Ancestral (high confidence)
AZOLI_0557	Ancestral (medium confidence)	AZOBR_10499	Ancestral (high confidence)
AZOLI_0558	Horizontally Transferred (high confidence)	AZOBR_10500	Ancestral (medium confidence)
AZOLI_0560	Ancestral (low confidence)	AZOBR_10501	Ancestral (medium confidence)
AZOLI_0561	Horizontally Transferred (high confidence)	AZOBR_10502	Ancestral (high confidence)
AZOLI_0562	Ancestral (low confidence)	AZOBR_10503	Ancestral (high confidence)
AZOLI_0563	Ancestral (medium confidence)	AZOBR_10504	Ancestral (high confidence)
AZOLI_0564	Ancestral (low confidence)	AZOBR_10505	Ancestral (high confidence)
AZOLI_0565	Ancestral (high confidence)	AZOBR_10506	Ancestral (high confidence)
AZOLI_0566	Horizontally Transferred (high confidence)	AZOBR_10507	Ancestral (low confidence)
AZOLI_0567	Ancestral (high confidence)	AZOBR_10508	Ancestral (medium confidence)
AZOLI_0568	Horizontally Transferred (high confidence)	AZOBR_10509	Horizontally Transferred (high confidence)
AZOLI_0569	Horizontally Transferred (high confidence)	AZOBR_10510	Horizontally Transferred (high confidence)
AZOLI_0570	Ancestral (low confidence)	AZOBR_10511	Horizontally Transferred (high confidence)
AZOLI_0571	Horizontally Transferred (high confidence)	AZOBR_10512	Horizontally Transferred (high confidence)
AZOLI_0572	Unassigned	AZOBR_10513	Horizontally Transferred (high confidence)
AZOLI_0573	Ancestral (medium confidence)	AZOBR_10514	Ancestral (medium confidence)
AZOLI_0574	Ancestral (medium confidence)	AZOBR_10515	Unassigned
AZOLI_0575	Ancestral (medium confidence)	AZOBR_10516	Unassigned
AZOLI_0576	Ancestral (medium confidence)	AZOBR_10517	Unassigned
AZOLI_0577	Ancestral (high confidence)	AZOBR_10518	Unassigned

AZOLI_0578	Ancestral (high confidence)	AZOBR_10519	Ancestral (medium confidence)
AZOLI_0579	Ancestral (high confidence)	AZOBR_10520	Ancestral (low confidence)
AZOLI_0580	Unassigned	AZOBR_10521	Ancestral (low confidence)
AZOLI_0581	Unassigned	AZOBR_10522	Unassigned
AZOLI_0583	Unassigned	AZOBR_10523	Ancestral (medium confidence)
AZOLI_0584	Horizontally Transferred (low confidence)	AZOBR_10524	Ancestral (low confidence)
AZOLI_0585	Ancestral (low confidence)	AZOBR_10525	Ancestral (low confidence)
AZOLI_0586	Ancestral (high confidence)	AZOBR_10526	Ancestral (high confidence)
AZOLI_0587	Horizontally Transferred (high confidence)	AZOBR_10527	Ancestral (medium confidence)
AZOLI_0588	Ancestral (high confidence)	AZOBR_10528	Ancestral (medium confidence)
AZOLI_0589	Ancestral (low confidence)	AZOBR_10529	Unassigned
AZOLI_0590	Ancestral (medium confidence)	AZOBR_10530	Unassigned
AZOLI_0591	Ancestral (high confidence)	AZOBR_10531	Ancestral (medium confidence)
AZOLI_0592	Ancestral (low confidence)	AZOBR_10532	Ancestral (medium confidence)
AZOLI_0593	Ancestral (low confidence)	AZOBR_10533	Unassigned
AZOLI_0595	Ancestral (low confidence)	AZOBR_10534	Horizontally Transferred (medium confidence)
AZOLI_0596	Ancestral (low confidence)	AZOBR_10535	Ancestral (medium confidence)
AZOLI_0597	Ancestral (high confidence)	AZOBR_10536	Ancestral (low confidence)
AZOLI_0598	Ancestral (high confidence)	AZOBR_10537	Ancestral (medium confidence)
AZOLI_0599	Ancestral (high confidence)	AZOBR_10538	Horizontally Transferred (high confidence)
AZOLI_0600	Horizontally Transferred (high confidence)	AZOBR_10539	Ancestral (medium confidence)
AZOLI_0601	Horizontally Transferred (high confidence)	AZOBR_10540	Ancestral (medium confidence)
AZOLI_0602	Ancestral (low confidence)	AZOBR_20001	Horizontally Transferred (high confidence)
AZOLI_0604	Horizontally Transferred (high confidence)	AZOBR_20002	Ancestral (low confidence)
AZOLI_0605	Ancestral (high confidence)	AZOBR_20003	Ancestral (low confidence)
AZOLI_0606	Ancestral (high confidence)	AZOBR_20004	Horizontally Transferred (medium confidence)
AZOLI_0607	Ancestral (high confidence)	AZOBR_20005	Ancestral (medium confidence)
AZOLI_0608	Ancestral (high confidence)	AZOBR_20006	Unassigned
AZOLI_0609	Ancestral (high confidence)	AZOBR_20007	Ancestral (high confidence)
AZOLI_0610	Horizontally Transferred (high confidence)	AZOBR_20008	Ancestral (medium confidence)
AZOLI_0611	Horizontally Transferred (high confidence)	AZOBR_20009	Ancestral (medium confidence)
AZOLI_0612	Unassigned	AZOBR_20010	Ancestral (medium confidence)
AZOLI_0613	Horizontally Transferred (high confidence)	AZOBR_20011	Ancestral (medium confidence)
AZOLI_0614	Horizontally Transferred (high confidence)	AZOBR_20012	Ancestral (low confidence)
AZOLI_0615	Unassigned	AZOBR_20013	Ancestral (low confidence)
AZOLI_0616	Horizontally Transferred (high confidence)	AZOBR_20014	Unassigned
AZOLI_0617	Ancestral (medium confidence)	AZOBR_20015	Unassigned
AZOLI_0618	Horizontally Transferred (low confidence)	AZOBR_20016	Ancestral (high confidence)
AZOLI_0619	Unassigned	AZOBR_20017	Horizontally Transferred (medium confidence)
AZOLI_0620	Ancestral (medium confidence)	AZOBR_20018	Unassigned
AZOLI_0621	Ancestral (medium confidence)	AZOBR_20019	Ancestral (low confidence)
AZOLI_0622	Ancestral (high confidence)	AZOBR_20020	Ancestral (low confidence)
AZOLI_0623	Ancestral (high confidence)	AZOBR_20021	Ancestral (medium confidence)
AZOLI_0624	Ancestral (low confidence)	AZOBR_20022	Ancestral (medium confidence)
AZOLI_0625	Horizontally Transferred (high confidence)	AZOBR_20023	Ancestral (high confidence)
AZOLI_0626	Horizontally Transferred (medium confidence)	AZOBR_20024	Horizontally Transferred (high confidence)
AZOLI_0627	Ancestral (medium confidence)	AZOBR_20025	Ancestral (low confidence)
AZOLI_0628	Ancestral (medium confidence)	AZOBR_20026	Horizontally Transferred (high confidence)
AZOLI_0629	Ancestral (medium confidence)	AZOBR_20027	Ancestral (low confidence)
AZOLI_0630	Unassigned	AZOBR_20028	Ancestral (low confidence)
AZOLI_0631	Ancestral (low confidence)	AZOBR_20029	Ancestral (low confidence)
AZOLI_0632	Ancestral (medium confidence)	AZOBR_20030	Unassigned
AZOLI_0633	Unassigned	AZOBR_20031	Ancestral (medium confidence)
AZOLI_0634	Ancestral (medium confidence)	AZOBR_20032	Horizontally Transferred (high confidence)
AZOLI_0635	Ancestral (medium confidence)	AZOBR_20033	Horizontally Transferred (high confidence)
AZOLI_0636	Horizontally Transferred (high confidence)	AZOBR_20034	Unassigned
AZOLI_0637	Ancestral (medium confidence)	AZOBR_30001	Ancestral (high confidence)
AZOLI_0638	Ancestral (low confidence)	AZOBR_30002	Ancestral (medium confidence)
AZOLI_0639	Ancestral (low confidence)	AZOBR_30003	Unassigned
AZOLI_0640	Unassigned	AZOBR_30004	Horizontally Transferred (medium confidence)
AZOLI_0641	Ancestral (high confidence)	AZOBR_30005	Ancestral (medium confidence)
AZOLI_0642	Ancestral (high confidence)	AZOBR_30006	Unassigned
AZOLI_0643	Ancestral (medium confidence)	AZOBR_30007	Horizontally Transferred (high confidence)
AZOLI_0644	Ancestral (medium confidence)	AZOBR_30008	Ancestral (low confidence)
AZOLI_0645	Ancestral (high confidence)	AZOBR_30009	Ancestral (low confidence)
AZOLI_0647	Ancestral (high confidence)	AZOBR_30010	Ancestral (low confidence)
AZOLI_0648	Ancestral (medium confidence)	AZOBR_30011	Ancestral (medium confidence)
AZOLI_0649	Ancestral (high confidence)	AZOBR_30012	Ancestral (medium confidence)
AZOLI_0651	Unassigned	AZOBR_30013	Horizontally Transferred (high confidence)
AZOLI_0652	Ancestral (low confidence)	AZOBR_30014	Horizontally Transferred (high confidence)
AZOLI_0653	Ancestral (low confidence)	AZOBR_30015	Horizontally Transferred (high confidence)
AZOLI_0654	Ancestral (low confidence)	AZOBR_30016	Horizontally Transferred (high confidence)
AZOLI_0655	Ancestral (low confidence)	AZOBR_30017	Unassigned
AZOLI_0656	Ancestral (high confidence)	AZOBR_30018	Ancestral (medium confidence)
AZOLI_0657	Ancestral (high confidence)	AZOBR_30019	Ancestral (low confidence)

AZOLI_0658	Ancestral (high confidence)	AZOBR_30020	Ancestral (low confidence)
AZOLI_0659	Ancestral (medium confidence)	AZOBR_30021	Unassigned
AZOLI_0660	Horizontally Transferred (high confidence)	AZOBR_30022	Horizontally Transferred (high confidence)
AZOLI_0661	Horizontally Transferred (medium confidence)	AZOBR_30023	Unassigned
AZOLI_0662	Ancestral (medium confidence)	AZOBR_30024	Ancestral (medium confidence)
AZOLI_0663	Ancestral (high confidence)	AZOBR_30025	Horizontally Transferred (medium confidence)
AZOLI_0664	Ancestral (high confidence)	AZOBR_30026	Horizontally Transferred (high confidence)
AZOLI_0665	Ancestral (high confidence)	AZOBR_30027	Horizontally Transferred (high confidence)
AZOLI_0666	Ancestral (high confidence)	AZOBR_30028	Horizontally Transferred (medium confidence)
AZOLI_0667	Ancestral (high confidence)	AZOBR_30029	Ancestral (low confidence)
AZOLI_0669	Horizontally Transferred (high confidence)	AZOBR_30030	Ancestral (low confidence)
AZOLI_0670	Unassigned	AZOBR_30031	Horizontally Transferred (high confidence)
AZOLI_0671	Ancestral (low confidence)	AZOBR_30032	Horizontally Transferred (high confidence)
AZOLI_0672	Horizontally Transferred (high confidence)	AZOBR_30033	Unassigned
AZOLI_0673	Horizontally Transferred (high confidence)	AZOBR_30034	Horizontally Transferred (low confidence)
AZOLI_0674	Horizontally Transferred (high confidence)	AZOBR_30035	Horizontally Transferred (high confidence)
AZOLI_0676	Unassigned	AZOBR_30036	Unassigned
AZOLI_0677	Ancestral (high confidence)	AZOBR_30037	Horizontally Transferred (medium confidence)
AZOLI_0678	Ancestral (low confidence)	AZOBR_30038	Horizontally Transferred (high confidence)
AZOLI_0679	Ancestral (low confidence)	AZOBR_30039	Unassigned
AZOLI_0680	Horizontally Transferred (low confidence)	AZOBR_30040	Horizontally Transferred (high confidence)
AZOLI_0681	Unassigned	AZOBR_30041	Horizontally Transferred (medium confidence)
AZOLI_0683	Horizontally Transferred (high confidence)	AZOBR_30042	Unassigned
AZOLI_0684	Ancestral (high confidence)	AZOBR_30043	Ancestral (medium confidence)
AZOLI_0685	Ancestral (high confidence)	AZOBR_30044	Ancestral (low confidence)
AZOLI_0686	Unassigned	AZOBR_40001	Ancestral (high confidence)
AZOLI_0687	Horizontally Transferred (high confidence)	AZOBR_40002	Ancestral (low confidence)
AZOLI_0688	Ancestral (low confidence)	AZOBR_40003	Ancestral (high confidence)
AZOLI_0689	Ancestral (low confidence)	AZOBR_40004	Unassigned
AZOLI_0690	Ancestral (high confidence)	AZOBR_40005	Unassigned
AZOLI_0691	Ancestral (high confidence)	AZOBR_40006	Unassigned
AZOLI_0692	Ancestral (low confidence)	AZOBR_40007	Unassigned
AZOLI_0693	Ancestral (low confidence)	AZOBR_40008	Unassigned
AZOLI_0694	Ancestral (high confidence)	AZOBR_40009	Horizontally Transferred (high confidence)
AZOLI_0695	Ancestral (low confidence)	AZOBR_40010	Unassigned
AZOLI_0696	Ancestral (low confidence)	AZOBR_40011	Unassigned
AZOLI_0697	Ancestral (low confidence)	AZOBR_40012	Unassigned
AZOLI_0698	Ancestral (medium confidence)	AZOBR_40013	Unassigned
AZOLI_0699	Ancestral (high confidence)	AZOBR_40014	Unassigned
AZOLI_0700	Ancestral (low confidence)	AZOBR_40015	Unassigned
AZOLI_0702	Ancestral (low confidence)	AZOBR_40016	Unassigned
AZOLI_0703	Ancestral (high confidence)	AZOBR_40017	Unassigned
AZOLI_0704	Ancestral (high confidence)	AZOBR_40018	Ancestral (medium confidence)
AZOLI_0705	Ancestral (low confidence)	AZOBR_40019	Ancestral (low confidence)
AZOLI_0706	Ancestral (high confidence)	AZOBR_40020	Unassigned
AZOLI_0707	Ancestral (high confidence)	AZOBR_40021	Horizontally Transferred (high confidence)
AZOLI_0709	Horizontally Transferred (medium confidence)	AZOBR_40022	Unassigned
AZOLI_0711	Unassigned	AZOBR_40023	Unassigned
AZOLI_0712	Ancestral (low confidence)	AZOBR_40024	Unassigned
AZOLI_0713	Ancestral (low confidence)	AZOBR_40025	Unassigned
AZOLI_0714	Horizontally Transferred (low confidence)	AZOBR_40026	Unassigned
AZOLI_0715	Ancestral (low confidence)	AZOBR_40027	Unassigned
AZOLI_0716	Ancestral (low confidence)	AZOBR_40028	Horizontally Transferred (low confidence)
AZOLI_0717	Unassigned	AZOBR_40029	Unassigned
AZOLI_0718	Horizontally Transferred (high confidence)	AZOBR_40030	Unassigned
AZOLI_0719	Horizontally Transferred (high confidence)	AZOBR_40031	Unassigned
AZOLI_0720	Unassigned	AZOBR_40032	Horizontally Transferred (high confidence)
AZOLI_0721	Horizontally Transferred (high confidence)	AZOBR_40033	Unassigned
AZOLI_0722	Unassigned	AZOBR_40034	Ancestral (low confidence)
AZOLI_0723	Unassigned	AZOBR_40035	Unassigned
AZOLI_0724	Horizontally Transferred (high confidence)	AZOBR_40036	Ancestral (medium confidence)
AZOLI_0725	Horizontally Transferred (high confidence)	AZOBR_40037	Ancestral (medium confidence)
AZOLI_0726	Horizontally Transferred (low confidence)	AZOBR_40038	Ancestral (high confidence)
AZOLI_0727	Horizontally Transferred (low confidence)	AZOBR_40039	Ancestral (high confidence)
AZOLI_0728	Ancestral (low confidence)	AZOBR_40040	Ancestral (high confidence)
AZOLI_0729	Unassigned	AZOBR_40041	Ancestral (low confidence)
AZOLI_0730	Ancestral (medium confidence)	AZOBR_40042	Horizontally Transferred (high confidence)
AZOLI_0731	Ancestral (low confidence)	AZOBR_40043	Unassigned
AZOLI_0732	Horizontally Transferred (high confidence)	AZOBR_40044	Ancestral (high confidence)
AZOLI_0733	Horizontally Transferred (medium confidence)	AZOBR_40045	Horizontally Transferred (high confidence)
AZOLI_0734	Horizontally Transferred (high confidence)	AZOBR_40046	Horizontally Transferred (high confidence)
AZOLI_0735	Horizontally Transferred (high confidence)	AZOBR_40047	Ancestral (low confidence)
AZOLI_0736	Horizontally Transferred (high confidence)	AZOBR_40048	Ancestral (high confidence)
AZOLI_0737	Horizontally Transferred (high confidence)	AZOBR_40049	Ancestral (high confidence)
AZOLI_0738	Unassigned	AZOBR_40050	Horizontally Transferred (low confidence)

AZOLI_0739	Ancestral (high confidence)	AZOBR_40051	Ancestral (high confidence)
AZOLI_0740	Ancestral (medium confidence)	AZOBR_40052	Ancestral (medium confidence)
AZOLI_0742	Unassigned	AZOBR_40053	Ancestral (high confidence)
AZOLI_0743	Unassigned	AZOBR_40054	Ancestral (low confidence)
AZOLI_0744	Unassigned	AZOBR_40055	Unassigned
AZOLI_0746	Ancestral (high confidence)	AZOBR_40056	Ancestral (high confidence)
AZOLI_0747	Horizontally Transferred (high confidence)	AZOBR_40057	Ancestral (high confidence)
AZOLI_0748	Horizontally Transferred (high confidence)	AZOBR_40058	Ancestral (medium confidence)
AZOLI_0750	Ancestral (medium confidence)	AZOBR_40059	Ancestral (low confidence)
AZOLI_0752	Ancestral (high confidence)	AZOBR_40060	Ancestral (low confidence)
AZOLI_0753	Ancestral (high confidence)	AZOBR_40061	Ancestral (medium confidence)
AZOLI_0754	Ancestral (high confidence)	AZOBR_40062	Ancestral (medium confidence)
AZOLI_0755	Ancestral (high confidence)	AZOBR_40063	Ancestral (low confidence)
AZOLI_0756	Ancestral (low confidence)	AZOBR_40064	Ancestral (medium confidence)
AZOLI_0758	Ancestral (low confidence)	AZOBR_40065	Ancestral (medium confidence)
AZOLI_0759	Ancestral (low confidence)	AZOBR_40066	Ancestral (low confidence)
AZOLI_0760	Ancestral (low confidence)	AZOBR_40067	Ancestral (medium confidence)
AZOLI_0761	Ancestral (low confidence)	AZOBR_40068	Horizontally Transferred (high confidence)
AZOLI_0762	Ancestral (high confidence)	AZOBR_40069	Unassigned
AZOLI_0763	Ancestral (high confidence)	AZOBR_40070	Horizontally Transferred (high confidence)
AZOLI_0764	Ancestral (low confidence)	AZOBR_40071	Ancestral (low confidence)
AZOLI_0766	Horizontally Transferred (high confidence)	AZOBR_40072	Ancestral (high confidence)
AZOLI_0767	Horizontally Transferred (high confidence)	AZOBR_40073	Unassigned
AZOLI_0768	Horizontally Transferred (high confidence)	AZOBR_40074	Ancestral (medium confidence)
AZOLI_0769	Ancestral (high confidence)	AZOBR_40075	Ancestral (high confidence)
AZOLI_0770	Horizontally Transferred (high confidence)	AZOBR_40076	Horizontally Transferred (high confidence)
AZOLI_0771	Ancestral (low confidence)	AZOBR_40077	Horizontally Transferred (high confidence)
AZOLI_0773	Horizontally Transferred (low confidence)	AZOBR_40078	Unassigned
AZOLI_0774	Unassigned	AZOBR_40079	Unassigned
AZOLI_0775	Ancestral (low confidence)	AZOBR_40080	Unassigned
AZOLI_0776	Ancestral (medium confidence)	AZOBR_40081	Ancestral (medium confidence)
AZOLI_0777	Horizontally Transferred (high confidence)	AZOBR_40082	Ancestral (low confidence)
AZOLI_0779	Ancestral (high confidence)	AZOBR_40083	Unassigned
AZOLI_0781	Ancestral (medium confidence)	AZOBR_40084	Horizontally Transferred (high confidence)
AZOLI_0782	Ancestral (high confidence)	AZOBR_40085	Horizontally Transferred (high confidence)
AZOLI_0783	Ancestral (medium confidence)	AZOBR_40086	Ancestral (low confidence)
AZOLI_0784	Ancestral (medium confidence)	AZOBR_40087	Unassigned
AZOLI_0785	Ancestral (high confidence)	AZOBR_40088	Ancestral (high confidence)
AZOLI_0786	Horizontally Transferred (medium confidence)	AZOBR_40089	Horizontally Transferred (high confidence)
AZOLI_0787	Ancestral (medium confidence)	AZOBR_40090	Ancestral (high confidence)
AZOLI_0788	Ancestral (medium confidence)	AZOBR_40091	Ancestral (high confidence)
AZOLI_0789	Ancestral (high confidence)	AZOBR_40092	Ancestral (high confidence)
AZOLI_0791	Horizontally Transferred (high confidence)	AZOBR_40093	Ancestral (medium confidence)
AZOLI_0792	Horizontally Transferred (high confidence)	AZOBR_40094	Ancestral (high confidence)
AZOLI_0793	Horizontally Transferred (high confidence)	AZOBR_40095	Ancestral (high confidence)
AZOLI_0794	Ancestral (medium confidence)	AZOBR_40096	Ancestral (low confidence)
AZOLI_0795	Unassigned	AZOBR_40097	Horizontally Transferred (low confidence)
AZOLI_0796	Ancestral (high confidence)	AZOBR_40098	Unassigned
AZOLI_0797	Ancestral (high confidence)	AZOBR_40099	Horizontally Transferred (high confidence)
AZOLI_0798	Ancestral (medium confidence)	AZOBR_40100	Ancestral (high confidence)
AZOLI_0799	Ancestral (high confidence)	AZOBR_40101	Ancestral (medium confidence)
AZOLI_0800	Ancestral (high confidence)	AZOBR_40102	Ancestral (high confidence)
AZOLI_0801	Ancestral (high confidence)	AZOBR_40103	Ancestral (medium confidence)
AZOLI_0802	Ancestral (high confidence)	AZOBR_40104	Unassigned
AZOLI_0803	Ancestral (high confidence)	AZOBR_40105	Unassigned
AZOLI_0805	Ancestral (high confidence)	AZOBR_40106	Horizontally Transferred (high confidence)
AZOLI_0807	Ancestral (medium confidence)	AZOBR_40107	Horizontally Transferred (high confidence)
AZOLI_0808	Horizontally Transferred (high confidence)	AZOBR_40108	Horizontally Transferred (high confidence)
AZOLI_0809	Ancestral (high confidence)	AZOBR_40109	Ancestral (high confidence)
AZOLI_0810	Ancestral (medium confidence)	AZOBR_40110	Ancestral (medium confidence)
AZOLI_0811	Horizontally Transferred (high confidence)	AZOBR_40111	Horizontally Transferred (high confidence)
AZOLI_0812	Ancestral (medium confidence)	AZOBR_40112	Horizontally Transferred (high confidence)
AZOLI_0813	Unassigned	AZOBR_40113	Ancestral (medium confidence)
AZOLI_0814	Ancestral (medium confidence)	AZOBR_40114	Ancestral (high confidence)
AZOLI_0815	Ancestral (low confidence)	AZOBR_40115	Unassigned
AZOLI_0816	Ancestral (low confidence)	AZOBR_40116	Ancestral (high confidence)
AZOLI_0819	Unassigned	AZOBR_40117	Ancestral (medium confidence)
AZOLI_0820	Horizontally Transferred (high confidence)	AZOBR_40118	Ancestral (medium confidence)
AZOLI_0821	Ancestral (high confidence)	AZOBR_40119	Ancestral (high confidence)
AZOLI_0822	Ancestral (low confidence)	AZOBR_40120	Ancestral (high confidence)
AZOLI_0823	Ancestral (high confidence)	AZOBR_40121	Ancestral (high confidence)
AZOLI_0824	Ancestral (high confidence)	AZOBR_40122	Unassigned
AZOLI_0825	Ancestral (medium confidence)	AZOBR_40123	Ancestral (medium confidence)
AZOLI_0826	Ancestral (high confidence)	AZOBR_40124	Ancestral (high confidence)
AZOLI_0827	Ancestral (high confidence)	AZOBR_40125	Ancestral (medium confidence)

AZOLI_0828	Ancestral (high confidence)	AZOBR_40126	Ancestral (medium confidence)
AZOLI_0829	Ancestral (high confidence)	AZOBR_40127	Ancestral (low confidence)
AZOLI_0830	Ancestral (medium confidence)	AZOBR_40128	Horizontally Transferred (medium confidence)
AZOLI_0831	Horizontally Transferred (medium confidence)	AZOBR_40129	Ancestral (low confidence)
AZOLI_0832	Ancestral (high confidence)	AZOBR_40130	Ancestral (high confidence)
AZOLI_0833	Ancestral (high confidence)	AZOBR_40131	Horizontally Transferred (high confidence)
AZOLI_0834	Unassigned	AZOBR_40132	Unassigned
AZOLI_0835	Ancestral (high confidence)	AZOBR_40133	Horizontally Transferred (high confidence)
AZOLI_0836	Ancestral (high confidence)	AZOBR_40134	Horizontally Transferred (high confidence)
AZOLI_0837	Ancestral (high confidence)	AZOBR_40135	Ancestral (medium confidence)
AZOLI_0838	Ancestral (medium confidence)	AZOBR_40136	Ancestral (high confidence)
AZOLI_0839	Ancestral (high confidence)	AZOBR_40137	Unassigned
AZOLI_0840	Ancestral (medium confidence)	AZOBR_40138	Ancestral (low confidence)
AZOLI_0841	Ancestral (low confidence)	AZOBR_40139	Ancestral (medium confidence)
AZOLI_0842	Ancestral (medium confidence)	AZOBR_40140	Ancestral (high confidence)
AZOLI_0843	Ancestral (medium confidence)	AZOBR_40141	Unassigned
AZOLI_0845	Ancestral (low confidence)	AZOBR_40142	Ancestral (medium confidence)
AZOLI_0846	Ancestral (high confidence)	AZOBR_40143	Ancestral (low confidence)
AZOLI_0847	Ancestral (high confidence)	AZOBR_40144	Horizontally Transferred (high confidence)
AZOLI_0848	Ancestral (medium confidence)	AZOBR_40145	Horizontally Transferred (high confidence)
AZOLI_0849	Ancestral (high confidence)	AZOBR_40146	Horizontally Transferred (high confidence)
AZOLI_0850	Horizontally Transferred (high confidence)	AZOBR_40147	Unassigned
AZOLI_0851	Ancestral (medium confidence)	AZOBR_40148	Horizontally Transferred (high confidence)
AZOLI_0852	Ancestral (high confidence)	AZOBR_40149	Unassigned
AZOLI_0853	Ancestral (low confidence)	AZOBR_40150	Horizontally Transferred (high confidence)
AZOLI_0854	Ancestral (medium confidence)	AZOBR_40151	Ancestral (low confidence)
AZOLI_0855	Ancestral (medium confidence)	AZOBR_40152	Horizontally Transferred (high confidence)
AZOLI_0856	Ancestral (low confidence)	AZOBR_40153	Horizontally Transferred (high confidence)
AZOLI_0857	Ancestral (high confidence)	AZOBR_40154	Horizontally Transferred (high confidence)
AZOLI_0858	Horizontally Transferred (high confidence)	AZOBR_40155	Horizontally Transferred (medium confidence)
AZOLI_0859	Ancestral (high confidence)	AZOBR_40156	Horizontally Transferred (high confidence)
AZOLI_0860	Unassigned	AZOBR_40157	Horizontally Transferred (high confidence)
AZOLI_0861	Ancestral (high confidence)	AZOBR_40158	Unassigned
AZOLI_0862	Ancestral (high confidence)	AZOBR_40159	Horizontally Transferred (high confidence)
AZOLI_0863	Ancestral (medium confidence)	AZOBR_40160	Ancestral (high confidence)
AZOLI_0864	Ancestral (high confidence)	AZOBR_40161	Ancestral (medium confidence)
AZOLI_0865	Unassigned	AZOBR_40162	Horizontally Transferred (high confidence)
AZOLI_0866	Ancestral (medium confidence)	AZOBR_40163	Horizontally Transferred (high confidence)
AZOLI_0867	Ancestral (medium confidence)	AZOBR_40164	Horizontally Transferred (low confidence)
AZOLI_0868	Ancestral (low confidence)	AZOBR_40165	Unassigned
AZOLI_0869	Ancestral (medium confidence)	AZOBR_40166	Ancestral (medium confidence)
AZOLI_0870	Ancestral (high confidence)	AZOBR_40167	Ancestral (medium confidence)
AZOLI_0871	Ancestral (high confidence)	AZOBR_40168	Ancestral (medium confidence)
AZOLI_0872	Ancestral (high confidence)	AZOBR_40169	Ancestral (high confidence)
AZOLI_0873	Ancestral (high confidence)	AZOBR_40170	Ancestral (low confidence)
AZOLI_0874	Ancestral (medium confidence)	AZOBR_40171	Unassigned
AZOLI_0875	Ancestral (high confidence)	AZOBR_40172	Ancestral (low confidence)
AZOLI_0876	Ancestral (high confidence)	AZOBR_40173	Ancestral (high confidence)
AZOLI_0877	Ancestral (high confidence)	AZOBR_40174	Ancestral (medium confidence)
AZOLI_0878	Ancestral (high confidence)	AZOBR_40175	Ancestral (medium confidence)
AZOLI_0879	Unassigned	AZOBR_40176	Unassigned
AZOLI_0880	Ancestral (high confidence)	AZOBR_40177	Ancestral (high confidence)
AZOLI_0881	Ancestral (high confidence)	AZOBR_40178	Ancestral (high confidence)
AZOLI_0883	Unassigned	AZOBR_40179	Ancestral (high confidence)
AZOLI_0884	Unassigned	AZOBR_40180	Ancestral (high confidence)
AZOLI_0885	Horizontally Transferred (high confidence)	AZOBR_40181	Ancestral (high confidence)
AZOLI_0886	Ancestral (low confidence)	AZOBR_40182	Ancestral (high confidence)
AZOLI_0887	Ancestral (low confidence)	AZOBR_40183	Horizontally Transferred (high confidence)
AZOLI_0888	Horizontally Transferred (high confidence)	AZOBR_40184	Ancestral (medium confidence)
AZOLI_0889	Unassigned	AZOBR_40185	Ancestral (medium confidence)
AZOLI_0890	Horizontally Transferred (medium confidence)	AZOBR_40186	Unassigned
AZOLI_0891	Ancestral (low confidence)	AZOBR_40187	Ancestral (high confidence)
AZOLI_0893	Ancestral (medium confidence)	AZOBR_40188	Ancestral (medium confidence)
AZOLI_0894	Unassigned	AZOBR_40189	Ancestral (medium confidence)
AZOLI_0895	Horizontally Transferred (low confidence)	AZOBR_40190	Ancestral (high confidence)
AZOLI_0896	Ancestral (low confidence)	AZOBR_40191	Ancestral (high confidence)
AZOLI_0897	Horizontally Transferred (high confidence)	AZOBR_40192	Ancestral (low confidence)
AZOLI_0898	Horizontally Transferred (medium confidence)	AZOBR_40193	Horizontally Transferred (high confidence)
AZOLI_0899	Horizontally Transferred (high confidence)	AZOBR_40194	Ancestral (high confidence)
AZOLI_0900	Horizontally Transferred (high confidence)	AZOBR_40195	Horizontally Transferred (high confidence)
AZOLI_0901	Horizontally Transferred (high confidence)	AZOBR_40196	Horizontally Transferred (high confidence)
AZOLI_0902	Horizontally Transferred (high confidence)	AZOBR_40197	Horizontally Transferred (high confidence)
AZOLI_0903	Horizontally Transferred (high confidence)	AZOBR_40198	Ancestral (high confidence)
AZOLI_0904	Unassigned	AZOBR_40199	Ancestral (medium confidence)
AZOLI_0905	Horizontally Transferred (high confidence)	AZOBR_40200	Ancestral (high confidence)

AZOLI_0906	Horizontally Transferred (medium confidence)	AZOBR_40201	Ancestral (high confidence)
AZOLI_0907	Horizontally Transferred (high confidence)	AZOBR_40202	Ancestral (low confidence)
AZOLI_0909	Horizontally Transferred (low confidence)	AZOBR_40203	Ancestral (medium confidence)
AZOLI_0910	Horizontally Transferred (low confidence)	AZOBR_40204	Unassigned
AZOLI_0911	Horizontally Transferred (low confidence)	AZOBR_40205	Ancestral (low confidence)
AZOLI_0912	Horizontally Transferred (low confidence)	AZOBR_40206	Ancestral (high confidence)
AZOLI_0913	Horizontally Transferred (low confidence)	AZOBR_40207	Horizontally Transferred (high confidence)
AZOLI_0914	Horizontally Transferred (low confidence)	AZOBR_40208	Ancestral (high confidence)
AZOLI_0915	Horizontally Transferred (high confidence)	AZOBR_40209	Ancestral (high confidence)
AZOLI_0916	Unassigned	AZOBR_40210	Ancestral (medium confidence)
AZOLI_0917	Horizontally Transferred (high confidence)	AZOBR_40211	Ancestral (low confidence)
AZOLI_0918	Horizontally Transferred (medium confidence)	AZOBR_40212	Unassigned
AZOLI_0919	Horizontally Transferred (high confidence)	AZOBR_40213	Horizontally Transferred (high confidence)
AZOLI_0920	Ancestral (medium confidence)	AZOBR_40214	Ancestral (high confidence)
AZOLI_0921	Unassigned	AZOBR_40215	Horizontally Transferred (high confidence)
AZOLI_0922	Ancestral (low confidence)	AZOBR_40216	Ancestral (medium confidence)
AZOLI_0923	Ancestral (medium confidence)	AZOBR_40217	Unassigned
AZOLI_0924	Horizontally Transferred (high confidence)	AZOBR_40218	Ancestral (high confidence)
AZOLI_0925	Ancestral (medium confidence)	AZOBR_40219	Unassigned
AZOLI_0926	Ancestral (low confidence)	AZOBR_40220	Horizontally Transferred (high confidence)
AZOLI_0927	Horizontally Transferred (high confidence)	AZOBR_40221	Horizontally Transferred (high confidence)
AZOLI_0928	Ancestral (high confidence)	AZOBR_40222	Unassigned
AZOLI_0929	Ancestral (high confidence)	AZOBR_40223	Ancestral (high confidence)
AZOLI_0930	Ancestral (low confidence)	AZOBR_40224	Unassigned
AZOLI_0931	Ancestral (high confidence)	AZOBR_40225	Ancestral (low confidence)
AZOLI_0932	Ancestral (high confidence)	AZOBR_40226	Horizontally Transferred (high confidence)
AZOLI_0933	Horizontally Transferred (high confidence)	AZOBR_40227	Ancestral (medium confidence)
AZOLI_0934	Unassigned	AZOBR_40228	Ancestral (medium confidence)
AZOLI_0935	Unassigned	AZOBR_40229	Ancestral (medium confidence)
AZOLI_0936	Ancestral (medium confidence)	AZOBR_40230	Ancestral (medium confidence)
AZOLI_0937	Ancestral (low confidence)	AZOBR_40231	Ancestral (low confidence)
AZOLI_0938	Horizontally Transferred (high confidence)	AZOBR_40232	Ancestral (medium confidence)
AZOLI_0939	Unassigned	AZOBR_40233	Ancestral (medium confidence)
AZOLI_0942	Horizontally Transferred (medium confidence)	AZOBR_40234	Ancestral (high confidence)
AZOLI_0943	Ancestral (low confidence)	AZOBR_40235	Ancestral (low confidence)
AZOLI_0944	Horizontally Transferred (high confidence)	AZOBR_40236	Unassigned
AZOLI_0946	Ancestral (medium confidence)	AZOBR_40237	Horizontally Transferred (high confidence)
AZOLI_0947	Ancestral (medium confidence)	AZOBR_40238	Horizontally Transferred (low confidence)
AZOLI_0948	Ancestral (low confidence)	AZOBR_40239	Horizontally Transferred (high confidence)
AZOLI_0950	Ancestral (low confidence)	AZOBR_40240	Horizontally Transferred (high confidence)
AZOLI_0951	Horizontally Transferred (high confidence)	AZOBR_40241	Ancestral (low confidence)
AZOLI_0952	Horizontally Transferred (high confidence)	AZOBR_40242	Unassigned
AZOLI_0953	Ancestral (low confidence)	AZOBR_40243	Ancestral (medium confidence)
AZOLI_0954	Unassigned	AZOBR_40244	Ancestral (low confidence)
AZOLI_0956	Unassigned	AZOBR_40245	Horizontally Transferred (low confidence)
AZOLI_0957	Unassigned	AZOBR_40246	Ancestral (medium confidence)
AZOLI_0958	Horizontally Transferred (high confidence)	AZOBR_40247	Horizontally Transferred (high confidence)
AZOLI_0959	Ancestral (medium confidence)	AZOBR_40248	Ancestral (low confidence)
AZOLI_0960	Ancestral (medium confidence)	AZOBR_40249	Unassigned
AZOLI_0961	Ancestral (low confidence)	AZOBR_40250	Ancestral (medium confidence)
AZOLI_0963	Horizontally Transferred (high confidence)	AZOBR_40251	Ancestral (medium confidence)
AZOLI_0964	Ancestral (high confidence)	AZOBR_40252	Ancestral (high confidence)
AZOLI_0965	Horizontally Transferred (high confidence)	AZOBR_40253	Ancestral (high confidence)
AZOLI_0966	Unassigned	AZOBR_40254	Ancestral (high confidence)
AZOLI_0967	Horizontally Transferred (high confidence)	AZOBR_40255	Ancestral (medium confidence)
AZOLI_0968	Ancestral (medium confidence)	AZOBR_40256	Ancestral (high confidence)
AZOLI_0969	Ancestral (medium confidence)	AZOBR_40257	Ancestral (medium confidence)
AZOLI_0970	Ancestral (high confidence)	AZOBR_40258	Horizontally Transferred (high confidence)
AZOLI_0971	Unassigned	AZOBR_40259	Horizontally Transferred (high confidence)
AZOLI_0972	Ancestral (high confidence)	AZOBR_40260	Horizontally Transferred (high confidence)
AZOLI_0973	Ancestral (low confidence)	AZOBR_40261	Horizontally Transferred (medium confidence)
AZOLI_0974	Ancestral (low confidence)	AZOBR_40262	Ancestral (medium confidence)
AZOLI_0975	Ancestral (low confidence)	AZOBR_40263	Unassigned
AZOLI_0976	Ancestral (medium confidence)	AZOBR_40264	Horizontally Transferred (high confidence)
AZOLI_0977	Ancestral (medium confidence)	AZOBR_40265	Unassigned
AZOLI_0978	Ancestral (high confidence)	AZOBR_40266	Unassigned
AZOLI_0979	Horizontally Transferred (high confidence)	AZOBR_40267	Horizontally Transferred (high confidence)
AZOLI_0980	Ancestral (high confidence)	AZOBR_40268	Ancestral (medium confidence)
AZOLI_0981	Horizontally Transferred (medium confidence)	AZOBR_40269	Ancestral (medium confidence)
AZOLI_0982	Horizontally Transferred (low confidence)	AZOBR_40270	Ancestral (high confidence)
AZOLI_0983	Ancestral (medium confidence)	AZOBR_40271	Ancestral (medium confidence)
AZOLI_0984	Ancestral (high confidence)	AZOBR_40272	Ancestral (medium confidence)
AZOLI_0985	Unassigned	AZOBR_40273	Ancestral (high confidence)
AZOLI_0986	Ancestral (medium confidence)	AZOBR_40274	Ancestral (high confidence)
AZOLI_0987	Ancestral (low confidence)	AZOBR_40275	Ancestral (medium confidence)

AZOLI_0988	Horizontally Transferred (high confidence)	AZOBR_40276	Unassigned
AZOLI_0989	Unassigned	AZOBR_40277	Ancestral (high confidence)
AZOLI_0990	Ancestral (medium confidence)	AZOBR_40278	Ancestral (low confidence)
AZOLI_0991	Horizontally Transferred (high confidence)	AZOBR_40279	Ancestral (medium confidence)
AZOLI_0992	Ancestral (low confidence)	AZOBR_40280	Ancestral (high confidence)
AZOLI_0993	Ancestral (medium confidence)	AZOBR_40281	Ancestral (low confidence)
AZOLI_0994	Unassigned	AZOBR_40282	Ancestral (medium confidence)
AZOLI_0995	Horizontally Transferred (high confidence)	AZOBR_40283	Ancestral (medium confidence)
AZOLI_0997	Ancestral (medium confidence)	AZOBR_40284	Horizontally Transferred (high confidence)
AZOLI_0998	Horizontally Transferred (low confidence)	AZOBR_40285	Ancestral (low confidence)
AZOLI_0999	Horizontally Transferred (high confidence)	AZOBR_40286	Ancestral (medium confidence)
AZOLI_1000	Horizontally Transferred (medium confidence)	AZOBR_40287	Horizontally Transferred (high confidence)
AZOLI_1001	Ancestral (low confidence)	AZOBR_40288	Ancestral (high confidence)
AZOLI_1002	Unassigned	AZOBR_40289	Horizontally Transferred (high confidence)
AZOLI_1003	Unassigned	AZOBR_40290	Horizontally Transferred (high confidence)
AZOLI_1004	Horizontally Transferred (medium confidence)	AZOBR_40291	Unassigned
AZOLI_1005	Horizontally Transferred (low confidence)	AZOBR_40292	Ancestral (medium confidence)
AZOLI_1006	Horizontally Transferred (low confidence)	AZOBR_40293	Unassigned
AZOLI_1007	Horizontally Transferred (low confidence)	AZOBR_40294	Unassigned
AZOLI_1008	Horizontally Transferred (medium confidence)	AZOBR_40295	Unassigned
AZOLI_1009	Horizontally Transferred (low confidence)	AZOBR_40296	Unassigned
AZOLI_1010	Horizontally Transferred (low confidence)	AZOBR_40297	Ancestral (high confidence)
AZOLI_1011	Horizontally Transferred (medium confidence)	AZOBR_40298	Ancestral (high confidence)
AZOLI_1012	Horizontally Transferred (high confidence)	AZOBR_40299	Ancestral (high confidence)
AZOLI_1013	Horizontally Transferred (high confidence)	AZOBR_40300	Ancestral (high confidence)
AZOLI_1014	Horizontally Transferred (high confidence)	AZOBR_40301	Ancestral (medium confidence)
AZOLI_1015	Horizontally Transferred (low confidence)	AZOBR_40302	Ancestral (medium confidence)
AZOLI_1016	Horizontally Transferred (medium confidence)	AZOBR_40303	Ancestral (high confidence)
AZOLI_1018	Horizontally Transferred (high confidence)	AZOBR_40304	Ancestral (high confidence)
AZOLI_1019	Horizontally Transferred (low confidence)	AZOBR_40305	Ancestral (high confidence)
AZOLI_1020	Horizontally Transferred (low confidence)	AZOBR_40306	Ancestral (high confidence)
AZOLI_1021	Horizontally Transferred (low confidence)	AZOBR_40307	Ancestral (high confidence)
AZOLI_1022	Ancestral (medium confidence)	AZOBR_40308	Ancestral (medium confidence)
AZOLI_1023	Ancestral (medium confidence)	AZOBR_40309	Ancestral (high confidence)
AZOLI_1024	Ancestral (medium confidence)	AZOBR_40310	Ancestral (low confidence)
AZOLI_1025	Horizontally Transferred (high confidence)	AZOBR_40311	Ancestral (high confidence)
AZOLI_1026	Ancestral (medium confidence)	AZOBR_40312	Ancestral (high confidence)
AZOLI_1027	Ancestral (medium confidence)	AZOBR_40313	Ancestral (medium confidence)
AZOLI_1028	Ancestral (medium confidence)	AZOBR_40314	Ancestral (high confidence)
AZOLI_1029	Ancestral (medium confidence)	AZOBR_40315	Unassigned
AZOLI_1030	Ancestral (low confidence)	AZOBR_40316	Ancestral (high confidence)
AZOLI_1031	Ancestral (low confidence)	AZOBR_40317	Ancestral (low confidence)
AZOLI_1032	Horizontally Transferred (medium confidence)	AZOBR_40318	Ancestral (high confidence)
AZOLI_1033	Ancestral (low confidence)	AZOBR_40319	Ancestral (high confidence)
AZOLI_1034	Ancestral (low confidence)	AZOBR_40320	Ancestral (low confidence)
AZOLI_1036	Ancestral (high confidence)	AZOBR_40321	Ancestral (low confidence)
AZOLI_1037	Ancestral (high confidence)	AZOBR_40322	Ancestral (low confidence)
AZOLI_1039	Horizontally Transferred (low confidence)	AZOBR_40323	Ancestral (high confidence)
AZOLI_1040	Horizontally Transferred (high confidence)	AZOBR_40324	Ancestral (high confidence)
AZOLI_1041	Unassigned	AZOBR_40325	Ancestral (high confidence)
AZOLI_1042	Ancestral (medium confidence)	AZOBR_40326	Ancestral (medium confidence)
AZOLI_1044	Unassigned	AZOBR_40327	Horizontally Transferred (medium confidence)
AZOLI_1046	Ancestral (medium confidence)	AZOBR_40328	Ancestral (low confidence)
AZOLI_1047	Horizontally Transferred (high confidence)	AZOBR_40329	Ancestral (low confidence)
AZOLI_1048	Ancestral (medium confidence)	AZOBR_40330	Ancestral (low confidence)
AZOLI_1049	Ancestral (medium confidence)	AZOBR_40331	Unassigned
AZOLI_1050	Horizontally Transferred (high confidence)	AZOBR_40332	Ancestral (high confidence)
AZOLI_1052	Ancestral (medium confidence)	AZOBR_40333	Unassigned
AZOLI_1053	Ancestral (medium confidence)	AZOBR_40334	Ancestral (medium confidence)
AZOLI_1054	Horizontally Transferred (high confidence)	AZOBR_40335	Ancestral (medium confidence)
AZOLI_1055	Ancestral (medium confidence)	AZOBR_40336	Ancestral (low confidence)
AZOLI_1056	Ancestral (medium confidence)	AZOBR_40337	Horizontally Transferred (low confidence)
AZOLI_1057	Ancestral (low confidence)	AZOBR_40338	Ancestral (low confidence)
AZOLI_1058	Unassigned	AZOBR_40339	Ancestral (low confidence)
AZOLI_1059	Horizontally Transferred (high confidence)	AZOBR_40340	Ancestral (medium confidence)
AZOLI_1060	Horizontally Transferred (high confidence)	AZOBR_40341	Ancestral (high confidence)
AZOLI_1061	Ancestral (high confidence)	AZOBR_40342	Horizontally Transferred (high confidence)
AZOLI_1062	Ancestral (low confidence)	AZOBR_40343	Ancestral (medium confidence)
AZOLI_1063	Ancestral (high confidence)	AZOBR_40344	Horizontally Transferred (high confidence)
AZOLI_1064	Ancestral (high confidence)	AZOBR_40345	Horizontally Transferred (high confidence)
AZOLI_1065	Ancestral (high confidence)	AZOBR_40346	Ancestral (medium confidence)
AZOLI_1066	Ancestral (high confidence)	AZOBR_40347	Horizontally Transferred (high confidence)
AZOLI_1068	Ancestral (high confidence)	AZOBR_40348	Horizontally Transferred (high confidence)
AZOLI_1069	Ancestral (high confidence)	AZOBR_40349	Ancestral (medium confidence)
AZOLI_1070	Ancestral (high confidence)	AZOBR_40350	Horizontally Transferred (high confidence)

AZOLI_1071	Ancestral (high confidence)	AZOBR_40351	Unassigned
AZOLI_1072	Ancestral (medium confidence)	AZOBR_40352	Ancestral (medium confidence)
AZOLI_1073	Ancestral (medium confidence)	AZOBR_40353	Ancestral (high confidence)
AZOLI_1074	Ancestral (high confidence)	AZOBR_40354	Ancestral (low confidence)
AZOLI_1075	Ancestral (high confidence)	AZOBR_40355	Unassigned
AZOLI_1076	Ancestral (high confidence)	AZOBR_40356	Ancestral (high confidence)
AZOLI_1077	Ancestral (high confidence)	AZOBR_40357	Ancestral (high confidence)
AZOLI_1078	Ancestral (high confidence)	AZOBR_40358	Horizontally Transferred (high confidence)
AZOLI_1079	Ancestral (high confidence)	AZOBR_40359	Horizontally Transferred (high confidence)
AZOLI_1080	Ancestral (high confidence)	AZOBR_40360	Horizontally Transferred (medium confidence)
AZOLI_1081	Ancestral (high confidence)	AZOBR_40361	Ancestral (high confidence)
AZOLI_1082	Ancestral (high confidence)	AZOBR_40362	Ancestral (high confidence)
AZOLI_1083	Ancestral (high confidence)	AZOBR_40363	Ancestral (high confidence)
AZOLI_1084	Ancestral (medium confidence)	AZOBR_40364	Ancestral (high confidence)
AZOLI_1085	Horizontally Transferred (high confidence)	AZOBR_40365	Unassigned
AZOLI_1086	Unassigned	AZOBR_40366	Unassigned
AZOLI_1088	Ancestral (medium confidence)	AZOBR_40367	Ancestral (high confidence)
AZOLI_1089	Ancestral (high confidence)	AZOBR_40368	Ancestral (medium confidence)
AZOLI_1090	Ancestral (high confidence)	AZOBR_40369	Horizontally Transferred (high confidence)
AZOLI_1091	Ancestral (high confidence)	AZOBR_40370	Ancestral (low confidence)
AZOLI_1092	Ancestral (high confidence)	AZOBR_40371	Ancestral (low confidence)
AZOLI_1093	Ancestral (high confidence)	AZOBR_40372	Horizontally Transferred (high confidence)
AZOLI_1094	Unassigned	AZOBR_40373	Horizontally Transferred (high confidence)
AZOLI_1095	Ancestral (medium confidence)	AZOBR_40374	Unassigned
AZOLI_1096	Ancestral (high confidence)	AZOBR_40375	Ancestral (high confidence)
AZOLI_1097	Ancestral (high confidence)	AZOBR_40376	Ancestral (medium confidence)
AZOLI_1098	Horizontally Transferred (high confidence)	AZOBR_40377	Unassigned
AZOLI_1099	Ancestral (high confidence)	AZOBR_40378	Ancestral (high confidence)
AZOLI_1100	Ancestral (high confidence)	AZOBR_40379	Ancestral (high confidence)
AZOLI_1101	Ancestral (medium confidence)	AZOBR_40380	Horizontally Transferred (high confidence)
AZOLI_1102	Ancestral (high confidence)	AZOBR_40381	Horizontally Transferred (high confidence)
AZOLI_1103	Ancestral (high confidence)	AZOBR_40382	Horizontally Transferred (high confidence)
AZOLI_1104	Ancestral (medium confidence)	AZOBR_40383	Horizontally Transferred (high confidence)
AZOLI_1105	Horizontally Transferred (high confidence)	AZOBR_40384	Horizontally Transferred (high confidence)
AZOLI_1106	Horizontally Transferred (high confidence)	AZOBR_40385	Ancestral (high confidence)
AZOLI_1107	Ancestral (low confidence)	AZOBR_40386	Ancestral (high confidence)
AZOLI_1108	Horizontally Transferred (high confidence)	AZOBR_40387	Ancestral (medium confidence)
AZOLI_1109	Horizontally Transferred (high confidence)	AZOBR_40388	Ancestral (high confidence)
AZOLI_1110	Ancestral (high confidence)	AZOBR_40389	Ancestral (medium confidence)
AZOLI_1111	Ancestral (medium confidence)	AZOBR_40390	Ancestral (high confidence)
AZOLI_1112	Ancestral (high confidence)	AZOBR_40391	Unassigned
AZOLI_1113	Unassigned	AZOBR_40392	Horizontally Transferred (high confidence)
AZOLI_1115	Ancestral (high confidence)	AZOBR_40393	Ancestral (medium confidence)
AZOLI_1116	Ancestral (medium confidence)	AZOBR_40394	Ancestral (high confidence)
AZOLI_1117	Unassigned	AZOBR_40395	Ancestral (high confidence)
AZOLI_1118	Ancestral (low confidence)	AZOBR_40396	Ancestral (medium confidence)
AZOLI_1119	Horizontally Transferred (high confidence)	AZOBR_40397	Ancestral (high confidence)
AZOLI_1120	Ancestral (medium confidence)	AZOBR_40398	Horizontally Transferred (high confidence)
AZOLI_1121	Horizontally Transferred (high confidence)	AZOBR_40399	Unassigned
AZOLI_1122	Ancestral (medium confidence)	AZOBR_40400	Unassigned
AZOLI_1123	Ancestral (medium confidence)	AZOBR_40401	Ancestral (low confidence)
AZOLI_1124	Ancestral (low confidence)	AZOBR_40402	Unassigned
AZOLI_1125	Unassigned	AZOBR_40403	Unassigned
AZOLI_1126	Ancestral (high confidence)	AZOBR_40404	Horizontally Transferred (high confidence)
AZOLI_1127	Ancestral (low confidence)	AZOBR_40405	Ancestral (medium confidence)
AZOLI_1128	Ancestral (low confidence)	AZOBR_40406	Ancestral (low confidence)
AZOLI_1129	Ancestral (medium confidence)	AZOBR_40407	Ancestral (low confidence)
AZOLI_1130	Horizontally Transferred (high confidence)	AZOBR_40408	Ancestral (high confidence)
AZOLI_1131	Ancestral (medium confidence)	AZOBR_40409	Ancestral (high confidence)
AZOLI_1132	Ancestral (low confidence)	AZOBR_40410	Horizontally Transferred (high confidence)
AZOLI_1134	Ancestral (high confidence)	AZOBR_40411	Ancestral (low confidence)
AZOLI_1135	Horizontally Transferred (high confidence)	AZOBR_40412	Ancestral (low confidence)
AZOLI_1136	Horizontally Transferred (high confidence)	AZOBR_40413	Horizontally Transferred (high confidence)
AZOLI_1137	Ancestral (high confidence)	AZOBR_40414	Ancestral (high confidence)
AZOLI_1138	Ancestral (medium confidence)	AZOBR_40415	Ancestral (medium confidence)
AZOLI_1139	Ancestral (medium confidence)	AZOBR_40416	Ancestral (low confidence)
AZOLI_1140	Ancestral (high confidence)	AZOBR_40417	Horizontally Transferred (high confidence)
AZOLI_1141	Ancestral (high confidence)	AZOBR_40418	Unassigned
AZOLI_1142	Horizontally Transferred (high confidence)	AZOBR_40419	Ancestral (low confidence)
AZOLI_1143	Ancestral (medium confidence)	AZOBR_40420	Horizontally Transferred (high confidence)
AZOLI_1144	Ancestral (medium confidence)	AZOBR_40421	Horizontally Transferred (high confidence)
AZOLI_1145	Horizontally Transferred (high confidence)	AZOBR_40422	Horizontally Transferred (high confidence)
AZOLI_1146	Ancestral (low confidence)	AZOBR_40423	Unassigned
AZOLI_1147	Ancestral (low confidence)	AZOBR_40424	Horizontally Transferred (high confidence)
AZOLI_1148	Horizontally Transferred (medium confidence)	AZOBR_40425	Unassigned

AZOLI_1149	Unassigned	AZOBR_40426	Horizontally Transferred (low confidence)
AZOLI_1150	Ancestral (medium confidence)	AZOBR_40427	Horizontally Transferred (medium confidence)
AZOLI_1151	Ancestral (high confidence)	AZOBR_40428	Horizontally Transferred (high confidence)
AZOLI_1152	Ancestral (medium confidence)	AZOBR_40429	Horizontally Transferred (high confidence)
AZOLI_1153	Ancestral (medium confidence)	AZOBR_40430	Unassigned
AZOLI_1154	Ancestral (medium confidence)	AZOBR_40431	Horizontally Transferred (high confidence)
AZOLI_1155	Horizontally Transferred (medium confidence)	AZOBR_40432	Horizontally Transferred (high confidence)
AZOLI_1156	Ancestral (medium confidence)	AZOBR_40433	Horizontally Transferred (high confidence)
AZOLI_1157	Ancestral (high confidence)	AZOBR_40434	Horizontally Transferred (high confidence)
AZOLI_1158	Ancestral (medium confidence)	AZOBR_40435	Horizontally Transferred (high confidence)
AZOLI_1160	Ancestral (medium confidence)	AZOBR_40436	Ancestral (medium confidence)
AZOLI_1161	Horizontally Transferred (high confidence)	AZOBR_40437	Unassigned
AZOLI_1162	Horizontally Transferred (high confidence)	AZOBR_40438	Horizontally Transferred (high confidence)
AZOLI_1163	Unassigned	AZOBR_40439	Horizontally Transferred (high confidence)
AZOLI_1164	Ancestral (high confidence)	AZOBR_40440	Horizontally Transferred (high confidence)
AZOLI_1165	Ancestral (high confidence)	AZOBR_40441	Ancestral (low confidence)
AZOLI_1166	Ancestral (high confidence)	AZOBR_40442	Horizontally Transferred (high confidence)
AZOLI_1167	Unassigned	AZOBR_40443	Horizontally Transferred (high confidence)
AZOLI_1168	Ancestral (low confidence)	AZOBR_40444	Unassigned
AZOLI_1169	Ancestral (low confidence)	AZOBR_40445	Horizontally Transferred (medium confidence)
AZOLI_1170	Ancestral (low confidence)	AZOBR_50001	Unassigned
AZOLI_1171	Ancestral (low confidence)	AZOBR_50002	Unassigned
AZOLI_1172	Ancestral (low confidence)	AZOBR_50003	Horizontally Transferred (high confidence)
AZOLI_1173	Ancestral (low confidence)	AZOBR_50004	Horizontally Transferred (high confidence)
AZOLI_1174	Ancestral (low confidence)	AZOBR_50005	Unassigned
AZOLI_1175	Ancestral (low confidence)	AZOBR_50006	Horizontally Transferred (high confidence)
AZOLI_1176	Horizontally Transferred (high confidence)	AZOBR_50007	Unassigned
AZOLI_1177	Horizontally Transferred (high confidence)	AZOBR_50008	Horizontally Transferred (high confidence)
AZOLI_1178	Ancestral (low confidence)	AZOBR_50009	Ancestral (low confidence)
AZOLI_1179	Horizontally Transferred (high confidence)	AZOBR_50010	Unassigned
AZOLI_1180	Ancestral (low confidence)	AZOBR_50011	Horizontally Transferred (high confidence)
AZOLI_1181	Ancestral (medium confidence)	AZOBR_50012	Ancestral (low confidence)
AZOLI_1182	Horizontally Transferred (high confidence)	AZOBR_50013	Ancestral (low confidence)
AZOLI_1183	Ancestral (medium confidence)	AZOBR_50014	Ancestral (low confidence)
AZOLI_1185	Ancestral (high confidence)	AZOBR_50015	Ancestral (low confidence)
AZOLI_1186	Ancestral (high confidence)	AZOBR_50016	Horizontally Transferred (high confidence)
AZOLI_1187	Ancestral (high confidence)	AZOBR_50017	Ancestral (low confidence)
AZOLI_1188	Ancestral (high confidence)	AZOBR_50018	Ancestral (low confidence)
AZOLI_1189	Horizontally Transferred (high confidence)	AZOBR_50019	Ancestral (low confidence)
AZOLI_1190	Ancestral (high confidence)	AZOBR_50020	Ancestral (low confidence)
AZOLI_1191	Ancestral (high confidence)	AZOBR_50021	Ancestral (low confidence)
AZOLI_1192	Ancestral (high confidence)	AZOBR_50022	Ancestral (low confidence)
AZOLI_1193	Ancestral (low confidence)	AZOBR_50023	Ancestral (low confidence)
AZOLI_1194	Ancestral (high confidence)	AZOBR_50024	Ancestral (low confidence)
AZOLI_1195	Ancestral (medium confidence)	AZOBR_50025	Horizontally Transferred (high confidence)
AZOLI_1196	Ancestral (medium confidence)	AZOBR_50026	Ancestral (medium confidence)
AZOLI_1197	Ancestral (high confidence)	AZOBR_50027	Horizontally Transferred (high confidence)
AZOLI_1199	Ancestral (low confidence)	AZOBR_50028	Unassigned
AZOLI_1200	Horizontally Transferred (high confidence)	AZOBR_50029	Horizontally Transferred (high confidence)
AZOLI_1202	Unassigned	AZOBR_50030	Horizontally Transferred (high confidence)
AZOLI_1203	Ancestral (medium confidence)	AZOBR_50031	Unassigned
AZOLI_1204	Ancestral (low confidence)	AZOBR_50032	Horizontally Transferred (medium confidence)
AZOLI_1205	Ancestral (high confidence)	AZOBR_50033	Horizontally Transferred (high confidence)
AZOLI_1206	Ancestral (low confidence)	AZOBR_50034	Horizontally Transferred (high confidence)
AZOLI_1207	Ancestral (high confidence)	AZOBR_50035	Horizontally Transferred (high confidence)
AZOLI_1208	Ancestral (low confidence)	AZOBR_50036	Horizontally Transferred (high confidence)
AZOLI_1209	Ancestral (low confidence)	AZOBR_50037	Horizontally Transferred (medium confidence)
AZOLI_1210	Horizontally Transferred (high confidence)	AZOBR_50038	Unassigned
AZOLI_1211	Horizontally Transferred (high confidence)	AZOBR_50039	Horizontally Transferred (medium confidence)
AZOLI_1212	Ancestral (low confidence)	AZOBR_50040	Horizontally Transferred (high confidence)
AZOLI_1213	Unassigned	AZOBR_50041	Unassigned
AZOLI_1214	Ancestral (low confidence)	AZOBR_50042	Horizontally Transferred (high confidence)
AZOLI_1215	Ancestral (medium confidence)	AZOBR_50043	Unassigned
AZOLI_1216	Ancestral (high confidence)	AZOBR_50044	Horizontally Transferred (high confidence)
AZOLI_1217	Horizontally Transferred (high confidence)	AZOBR_50045	Horizontally Transferred (high confidence)
AZOLI_1218	Ancestral (high confidence)	AZOBR_50046	Horizontally Transferred (high confidence)
AZOLI_1219	Ancestral (medium confidence)	AZOBR_50047	Horizontally Transferred (high confidence)
AZOLI_1220	Horizontally Transferred (high confidence)	AZOBR_50048	Horizontally Transferred (high confidence)
AZOLI_1221	Ancestral (high confidence)	AZOBR_50049	Unassigned
AZOLI_1222	Ancestral (high confidence)	AZOBR_50050	Horizontally Transferred (high confidence)
AZOLI_1223	Ancestral (low confidence)	AZOBR_50051	Horizontally Transferred (high confidence)
AZOLI_1224	Ancestral (high confidence)	AZOBR_50052	Unassigned
AZOLI_1225	Ancestral (medium confidence)	AZOBR_50053	Horizontally Transferred (high confidence)
AZOLI_1226	Ancestral (low confidence)	AZOBR_50054	Horizontally Transferred (low confidence)
AZOLI_1227	Ancestral (medium confidence)	AZOBR_50055	Horizontally Transferred (high confidence)

AZOLI_1228	Ancestral (low confidence)	AZOBR_50056	Horizontally Transferred (high confidence)
AZOLI_1229	Ancestral (low confidence)	AZOBR_50057	Horizontally Transferred (high confidence)
AZOLI_1230	Unassigned	AZOBR_50058	Horizontally Transferred (high confidence)
AZOLI_1231	Horizontally Transferred (high confidence)	AZOBR_50059	Ancestral (low confidence)
AZOLI_1232	Ancestral (medium confidence)	AZOBR_50060	Horizontally Transferred (high confidence)
AZOLI_1234	Ancestral (high confidence)	AZOBR_50061	Ancestral (low confidence)
AZOLI_1235	Ancestral (high confidence)	AZOBR_50062	Unassigned
AZOLI_1236	Ancestral (low confidence)	AZOBR_50063	Horizontally Transferred (high confidence)
AZOLI_1237	Horizontally Transferred (high confidence)	AZOBR_60001	Ancestral (low confidence)
AZOLI_1238	Horizontally Transferred (high confidence)	AZOBR_60002	Unassigned
AZOLI_1239	Ancestral (medium confidence)	AZOBR_60003	Horizontally Transferred (low confidence)
AZOLI_1241	Unassigned	AZOBR_60004	Horizontally Transferred (high confidence)
AZOLI_1242	Horizontally Transferred (medium confidence)	AZOBR_60005	Ancestral (low confidence)
AZOLI_1243	Horizontally Transferred (medium confidence)	AZOBR_60006	Ancestral (medium confidence)
AZOLI_1244	Ancestral (medium confidence)	AZOBR_60007	Ancestral (medium confidence)
AZOLI_1245	Ancestral (medium confidence)	AZOBR_60008	Horizontally Transferred (medium confidence)
AZOLI_1246	Ancestral (high confidence)	AZOBR_60009	Ancestral (low confidence)
AZOLI_1248	Unassigned	AZOBR_60010	Unassigned
AZOLI_1251	Unassigned	AZOBR_60011	Ancestral (low confidence)
AZOLI_1252	Ancestral (high confidence)	AZOBR_60012	Horizontally Transferred (high confidence)
AZOLI_1253	Ancestral (medium confidence)	AZOBR_60013	Horizontally Transferred (high confidence)
AZOLI_1254	Ancestral (low confidence)	AZOBR_60014	Unassigned
AZOLI_1255	Ancestral (high confidence)	AZOBR_60015	Horizontally Transferred (high confidence)
AZOLI_1256	Unassigned	AZOBR_60016	Horizontally Transferred (high confidence)
AZOLI_1257	Horizontally Transferred (high confidence)	AZOBR_60017	Unassigned
AZOLI_1258	Ancestral (low confidence)	AZOBR_60018	Horizontally Transferred (medium confidence)
AZOLI_1259	Ancestral (medium confidence)	AZOBR_60019	Ancestral (low confidence)
AZOLI_1260	Unassigned	AZOBR_60020	Unassigned
AZOLI_1261	Ancestral (high confidence)	AZOBR_60021	Horizontally Transferred (high confidence)
AZOLI_1262	Ancestral (low confidence)	AZOBR_60022	Horizontally Transferred (medium confidence)
AZOLI_1263	Ancestral (medium confidence)	AZOBR_60023	Horizontally Transferred (high confidence)
AZOLI_1264	Ancestral (medium confidence)	AZOBR_60024	Horizontally Transferred (medium confidence)
AZOLI_1265	Horizontally Transferred (high confidence)	AZOBR_60025	Horizontally Transferred (high confidence)
AZOLI_1266	Ancestral (low confidence)	AZOBR_70001	Horizontally Transferred (high confidence)
AZOLI_1269	Horizontally Transferred (high confidence)	AZOBR_70002	Ancestral (high confidence)
AZOLI_1270	Ancestral (high confidence)	AZOBR_70003	Horizontally Transferred (high confidence)
AZOLI_1271	Ancestral (medium confidence)	AZOBR_70004	Horizontally Transferred (high confidence)
AZOLI_1274	Ancestral (high confidence)	AZOBR_70005	Unassigned
AZOLI_1275	Ancestral (high confidence)	AZOBR_70006	Unassigned
AZOLI_1276	Ancestral (medium confidence)	AZOBR_70007	Unassigned
AZOLI_1277	Ancestral (low confidence)	AZOBR_70008	Ancestral (low confidence)
AZOLI_1278	Unassigned	AZOBR_70009	Horizontally Transferred (high confidence)
AZOLI_1279	Ancestral (high confidence)	AZOBR_70010	Ancestral (high confidence)
AZOLI_1280	Ancestral (high confidence)	AZOBR_70011	Ancestral (high confidence)
AZOLI_1281	Ancestral (high confidence)	AZOBR_70012	Ancestral (high confidence)
AZOLI_1282	Ancestral (low confidence)	AZOBR_70013	Ancestral (high confidence)
AZOLI_1283	Ancestral (medium confidence)	AZOBR_70014	Ancestral (high confidence)
AZOLI_1284	Horizontally Transferred (high confidence)	AZOBR_70015	Unassigned
AZOLI_1285	Horizontally Transferred (high confidence)	AZOBR_70016	Horizontally Transferred (high confidence)
AZOLI_1286	Ancestral (low confidence)	AZOBR_70017	Ancestral (high confidence)
AZOLI_1287	Ancestral (low confidence)	AZOBR_70018	Unassigned
AZOLI_1288	Horizontally Transferred (low confidence)	AZOBR_70019	Ancestral (medium confidence)
AZOLI_1289	Horizontally Transferred (high confidence)	AZOBR_70020	Horizontally Transferred (high confidence)
AZOLI_1290	Horizontally Transferred (high confidence)	AZOBR_70021	Ancestral (low confidence)
AZOLI_1291	Horizontally Transferred (high confidence)	AZOBR_70022	Unassigned
AZOLI_1292	Horizontally Transferred (high confidence)	AZOBR_70023	Ancestral (medium confidence)
AZOLI_1293	Unassigned	AZOBR_70024	Unassigned
AZOLI_1294	Unassigned	AZOBR_70025	Unassigned
AZOLI_1296	Ancestral (low confidence)	AZOBR_70026	Ancestral (medium confidence)
AZOLI_1297	Ancestral (high confidence)	AZOBR_70027	Unassigned
AZOLI_1298	Ancestral (high confidence)	AZOBR_70028	Ancestral (high confidence)
AZOLI_1299	Ancestral (high confidence)	AZOBR_70029	Ancestral (low confidence)
AZOLI_1300	Ancestral (medium confidence)	AZOBR_70030	Unassigned
AZOLI_1301	Horizontally Transferred (medium confidence)	AZOBR_70031	Horizontally Transferred (low confidence)
AZOLI_1302	Ancestral (medium confidence)	AZOBR_70032	Unassigned
AZOLI_1303	Ancestral (high confidence)	AZOBR_70033	Ancestral (high confidence)
AZOLI_1304	Ancestral (high confidence)	AZOBR_70034	Ancestral (medium confidence)
AZOLI_1305	Ancestral (high confidence)	AZOBR_70035	Ancestral (low confidence)
AZOLI_1306	Ancestral (high confidence)	AZOBR_70036	Ancestral (medium confidence)
AZOLI_1307	Ancestral (high confidence)	AZOBR_70037	Ancestral (high confidence)
AZOLI_1308	Ancestral (medium confidence)	AZOBR_70038	Ancestral (low confidence)
AZOLI_1309	Unassigned	AZOBR_70039	Ancestral (high confidence)
AZOLI_1310	Ancestral (low confidence)	AZOBR_70040	Ancestral (medium confidence)
AZOLI_1311	Ancestral (high confidence)	AZOBR_70041	Ancestral (high confidence)
AZOLI_1312	Ancestral (high confidence)	AZOBR_70042	Ancestral (high confidence)

AZOLI_1313	Ancestral (high confidence)	AZOBR_70043	Ancestral (low confidence)
AZOLI_1314	Ancestral (low confidence)	AZOBR_70044	Ancestral (medium confidence)
AZOLI_1315	Horizontally Transferred (high confidence)	AZOBR_70045	Ancestral (high confidence)
AZOLI_1316	Ancestral (medium confidence)	AZOBR_70046	Ancestral (low confidence)
AZOLI_1317	Ancestral (medium confidence)	AZOBR_70047	Ancestral (low confidence)
AZOLI_1318	Ancestral (medium confidence)	AZOBR_70048	Ancestral (medium confidence)
AZOLI_1319	Unassigned	AZOBR_70049	Ancestral (medium confidence)
AZOLI_1320	Ancestral (low confidence)	AZOBR_70050	Unassigned
AZOLI_1321	Ancestral (low confidence)	AZOBR_70051	Ancestral (low confidence)
AZOLI_1322	Unassigned	AZOBR_70052	Ancestral (medium confidence)
AZOLI_1323	Horizontally Transferred (high confidence)	AZOBR_70053	Horizontally Transferred (high confidence)
AZOLI_1324	Ancestral (low confidence)	AZOBR_70054	Ancestral (medium confidence)
AZOLI_1325	Ancestral (medium confidence)	AZOBR_70055	Ancestral (medium confidence)
AZOLI_1326	Unassigned	AZOBR_70056	Ancestral (medium confidence)
AZOLI_1328	Ancestral (high confidence)	AZOBR_70057	Ancestral (medium confidence)
AZOLI_1329	Ancestral (high confidence)	AZOBR_70058	Unassigned
AZOLI_1330	Ancestral (high confidence)	AZOBR_70059	Unassigned
AZOLI_1331	Ancestral (high confidence)	AZOBR_70060	Horizontally Transferred (high confidence)
AZOLI_1332	Horizontally Transferred (high confidence)	AZOBR_70061	Unassigned
AZOLI_1333	Ancestral (high confidence)	AZOBR_70062	Ancestral (low confidence)
AZOLI_1334	Horizontally Transferred (medium confidence)	AZOBR_70063	Ancestral (low confidence)
AZOLI_1335	Ancestral (medium confidence)	AZOBR_70064	Unassigned
AZOLI_1336	Ancestral (high confidence)	AZOBR_70065	Ancestral (high confidence)
AZOLI_1337	Horizontally Transferred (high confidence)	AZOBR_70066	Ancestral (high confidence)
AZOLI_1338	Ancestral (high confidence)	AZOBR_70067	Ancestral (medium confidence)
AZOLI_1339	Ancestral (high confidence)	AZOBR_70068	Ancestral (high confidence)
AZOLI_1340	Ancestral (high confidence)	AZOBR_70069	Ancestral (high confidence)
AZOLI_1341	Ancestral (high confidence)	AZOBR_70070	Unassigned
AZOLI_1342	Ancestral (high confidence)	AZOBR_70071	Ancestral (high confidence)
AZOLI_1343	Ancestral (high confidence)	AZOBR_70072	Unassigned
AZOLI_1344	Ancestral (high confidence)	AZOBR_70073	Ancestral (high confidence)
AZOLI_1345	Ancestral (high confidence)	AZOBR_70074	Ancestral (high confidence)
AZOLI_1346	Horizontally Transferred (high confidence)	AZOBR_70075	Unassigned
AZOLI_1347	Horizontally Transferred (high confidence)	AZOBR_70076	Unassigned
AZOLI_1348	Ancestral (high confidence)	AZOBR_70077	Ancestral (low confidence)
AZOLI_1349	Ancestral (high confidence)	AZOBR_70078	Ancestral (low confidence)
AZOLI_1350	Ancestral (high confidence)	AZOBR_70079	Ancestral (low confidence)
AZOLI_1351	Horizontally Transferred (high confidence)	AZOBR_70080	Unassigned
AZOLI_1352	Horizontally Transferred (high confidence)	AZOBR_70081	Unassigned
AZOLI_1353	Ancestral (medium confidence)	AZOBR_70082	Ancestral (high confidence)
AZOLI_1355	Ancestral (medium confidence)	AZOBR_70083	Ancestral (medium confidence)
AZOLI_1356	Ancestral (low confidence)	AZOBR_70084	Unassigned
AZOLI_1357	Ancestral (low confidence)	AZOBR_70085	Ancestral (high confidence)
AZOLI_1358	Ancestral (low confidence)	AZOBR_70086	Ancestral (low confidence)
AZOLI_1359	Ancestral (low confidence)	AZOBR_70087	Unassigned
AZOLI_1360	Horizontally Transferred (high confidence)	AZOBR_70088	Ancestral (low confidence)
AZOLI_1361	Ancestral (medium confidence)	AZOBR_70089	Ancestral (low confidence)
AZOLI_1362	Horizontally Transferred (high confidence)	AZOBR_70090	Horizontally Transferred (high confidence)
AZOLI_1363	Horizontally Transferred (high confidence)	AZOBR_70091	Unassigned
AZOLI_1364	Ancestral (low confidence)	AZOBR_70092	Ancestral (high confidence)
AZOLI_1365	Horizontally Transferred (high confidence)	AZOBR_70093	Ancestral (medium confidence)
AZOLI_1366	Horizontally Transferred (low confidence)	AZOBR_70094	Ancestral (medium confidence)
AZOLI_1367	Unassigned	AZOBR_70095	Ancestral (high confidence)
AZOLI_1368	Horizontally Transferred (high confidence)	AZOBR_70096	Ancestral (low confidence)
AZOLI_1370	Ancestral (medium confidence)	AZOBR_70097	Ancestral (low confidence)
AZOLI_1371	Ancestral (low confidence)	AZOBR_70098	Ancestral (high confidence)
AZOLI_1372	Ancestral (high confidence)	AZOBR_70099	Unassigned
AZOLI_1373	Ancestral (high confidence)	AZOBR_70100	Unassigned
AZOLI_1375	Ancestral (medium confidence)	AZOBR_70101	Horizontally Transferred (high confidence)
AZOLI_1376	Ancestral (high confidence)	AZOBR_70102	Ancestral (high confidence)
AZOLI_1377	Ancestral (high confidence)	AZOBR_70103	Unassigned
AZOLI_1378	Horizontally Transferred (medium confidence)	AZOBR_70104	Horizontally Transferred (high confidence)
AZOLI_1379	Ancestral (high confidence)	AZOBR_70105	Unassigned
AZOLI_1380	Ancestral (high confidence)	AZOBR_70106	Ancestral (medium confidence)
AZOLI_1381	Ancestral (high confidence)	AZOBR_70107	Ancestral (medium confidence)
AZOLI_1382	Ancestral (high confidence)	AZOBR_70108	Ancestral (medium confidence)
AZOLI_1383	Ancestral (high confidence)	AZOBR_70109	Horizontally Transferred (medium confidence)
AZOLI_1384	Unassigned	AZOBR_70110	Horizontally Transferred (high confidence)
AZOLI_1385	Ancestral (medium confidence)	AZOBR_70111	Ancestral (high confidence)
AZOLI_1386	Ancestral (medium confidence)	AZOBR_70112	Horizontally Transferred (low confidence)
AZOLI_1387	Ancestral (medium confidence)	AZOBR_70113	Horizontally Transferred (medium confidence)
AZOLI_1388	Ancestral (medium confidence)	AZOBR_70114	Ancestral (low confidence)
AZOLI_1389	Ancestral (low confidence)	AZOBR_70115	Horizontally Transferred (high confidence)
AZOLI_1390	Ancestral (medium confidence)	AZOBR_70116	Ancestral (low confidence)
AZOLI_1391	Unassigned	AZOBR_70117	Ancestral (low confidence)

AZOLI_1392	Ancestral (low confidence)	AZOBR_70118	Unassigned
AZOLI_1393	Ancestral (high confidence)	AZOBR_70119	Ancestral (low confidence)
AZOLI_1394	Ancestral (medium confidence)	AZOBR_70120	Horizontally Transferred (high confidence)
AZOLI_1395	Ancestral (medium confidence)	AZOBR_70121	Horizontally Transferred (high confidence)
AZOLI_1396	Ancestral (high confidence)	AZOBR_70122	Horizontally Transferred (high confidence)
AZOLI_1397	Ancestral (high confidence)	AZOBR_70123	Ancestral (low confidence)
AZOLI_1398	Ancestral (high confidence)	AZOBR_70124	Horizontally Transferred (high confidence)
AZOLI_1399	Ancestral (high confidence)	AZOBR_70125	Horizontally Transferred (low confidence)
AZOLI_1400	Ancestral (high confidence)	AZOBR_70126	Unassigned
AZOLI_1401	Ancestral (high confidence)	AZOBR_70127	Horizontally Transferred (medium confidence)
AZOLI_1402	Ancestral (low confidence)	AZOBR_70128	Unassigned
AZOLI_1403	Ancestral (high confidence)	AZOBR_70129	Unassigned
AZOLI_1404	Unassigned	AZOBR_70130	Horizontally Transferred (high confidence)
AZOLI_1406	Ancestral (low confidence)	AZOBR_70131	Unassigned
AZOLI_1407	Ancestral (high confidence)	AZOBR_70132	Ancestral (high confidence)
AZOLI_1408	Horizontally Transferred (low confidence)	AZOBR_70133	Ancestral (high confidence)
AZOLI_1409	Ancestral (high confidence)	AZOBR_70134	Ancestral (low confidence)
AZOLI_1411	Ancestral (medium confidence)	AZOBR_70135	Horizontally Transferred (high confidence)
AZOLI_1412	Ancestral (low confidence)	AZOBR_70136	Ancestral (low confidence)
AZOLI_1413	Ancestral (high confidence)	AZOBR_70137	Horizontally Transferred (medium confidence)
AZOLI_1414	Ancestral (high confidence)	AZOBR_70138	Horizontally Transferred (high confidence)
AZOLI_1415	Ancestral (high confidence)	AZOBR_70139	Horizontally Transferred (high confidence)
AZOLI_1416	Ancestral (medium confidence)	AZOBR_70140	Horizontally Transferred (high confidence)
AZOLI_1417	Ancestral (medium confidence)	AZOBR_70141	Horizontally Transferred (high confidence)
AZOLI_1418	Ancestral (high confidence)	AZOBR_70142	Horizontally Transferred (high confidence)
AZOLI_1419	Ancestral (high confidence)	AZOBR_70143	Ancestral (high confidence)
AZOLI_1420	Ancestral (medium confidence)	AZOBR_70144	Unassigned
AZOLI_1421	Ancestral (high confidence)	AZOBR_70145	Unassigned
AZOLI_1422	Ancestral (medium confidence)	AZOBR_70146	Ancestral (low confidence)
AZOLI_1423	Ancestral (low confidence)	AZOBR_70147	Horizontally Transferred (high confidence)
AZOLI_1424	Ancestral (medium confidence)	AZOBR_70148	Horizontally Transferred (high confidence)
AZOLI_1425	Unassigned	AZOBR_70149	Unassigned
AZOLI_1426	Horizontally Transferred (high confidence)	AZOBR_70150	Ancestral (medium confidence)
AZOLI_1427	Ancestral (low confidence)	AZOBR_70151	Ancestral (low confidence)
AZOLI_1428	Ancestral (medium confidence)	AZOBR_70152	Unassigned
AZOLI_1430	Ancestral (medium confidence)	AZOBR_70153	Unassigned
AZOLI_1431	Horizontally Transferred (high confidence)	AZOBR_70154	Ancestral (low confidence)
AZOLI_1432	Ancestral (high confidence)	AZOBR_70155	Ancestral (high confidence)
AZOLI_1433	Ancestral (high confidence)	AZOBR_70156	Ancestral (medium confidence)
AZOLI_1434	Ancestral (high confidence)	AZOBR_70157	Ancestral (medium confidence)
AZOLI_1435	Ancestral (low confidence)	AZOBR_70158	Unassigned
AZOLI_1437	Ancestral (low confidence)	AZOBR_70159	Ancestral (medium confidence)
AZOLI_1438	Ancestral (high confidence)	AZOBR_70160	Unassigned
AZOLI_1439	Ancestral (low confidence)	AZOBR_70161	Ancestral (medium confidence)
AZOLI_1440	Horizontally Transferred (high confidence)	AZOBR_70162	Ancestral (low confidence)
AZOLI_1441	Horizontally Transferred (high confidence)	AZOBR_70163	Ancestral (low confidence)
AZOLI_1442	Horizontally Transferred (high confidence)	AZOBR_70164	Ancestral (medium confidence)
AZOLI_1443	Unassigned	AZOBR_70165	Ancestral (medium confidence)
AZOLI_1444	Horizontally Transferred (high confidence)	AZOBR_70166	Unassigned
AZOLI_1445	Ancestral (low confidence)	AZOBR_70167	Unassigned
AZOLI_1446	Ancestral (medium confidence)	AZOBR_70168	Horizontally Transferred (high confidence)
AZOLI_1447	Ancestral (medium confidence)	AZOBR_70169	Ancestral (medium confidence)
AZOLI_1448	Horizontally Transferred (high confidence)	AZOBR_70170	Ancestral (high confidence)
AZOLI_1449	Unassigned	AZOBR_70171	Unassigned
AZOLI_1450	Horizontally Transferred (high confidence)	AZOBR_70172	Ancestral (high confidence)
AZOLI_1451	Unassigned	AZOBR_70173	Ancestral (high confidence)
AZOLI_1452	Ancestral (medium confidence)	AZOBR_70174	Ancestral (high confidence)
AZOLI_1453	Ancestral (medium confidence)	AZOBR_70175	Ancestral (low confidence)
AZOLI_1454	Ancestral (low confidence)	AZOBR_70176	Ancestral (high confidence)
AZOLI_1455	Ancestral (medium confidence)	AZOBR_70177	Ancestral (high confidence)
AZOLI_1456	Horizontally Transferred (high confidence)	AZOBR_70178	Ancestral (high confidence)
AZOLI_1457	Ancestral (medium confidence)	AZOBR_70179	Ancestral (high confidence)
AZOLI_1458	Ancestral (high confidence)	AZOBR_70180	Horizontally Transferred (high confidence)
AZOLI_1459	Ancestral (high confidence)	AZOBR_70181	Ancestral (high confidence)
AZOLI_1460	Ancestral (medium confidence)	AZOBR_70182	Ancestral (high confidence)
AZOLI_1461	Ancestral (high confidence)	AZOBR_70183	Ancestral (high confidence)
AZOLI_1462	Horizontally Transferred (high confidence)	AZOBR_70184	Ancestral (high confidence)
AZOLI_1463	Horizontally Transferred (high confidence)	AZOBR_70185	Ancestral (high confidence)
AZOLI_1464	Ancestral (high confidence)	AZOBR_70186	Ancestral (high confidence)
AZOLI_1465	Horizontally Transferred (medium confidence)	AZOBR_70187	Ancestral (high confidence)
AZOLI_1466	Horizontally Transferred (medium confidence)	AZOBR_70188	Ancestral (medium confidence)
AZOLI_1467	Ancestral (high confidence)	AZOBR_70189	Ancestral (medium confidence)
AZOLI_1468	Horizontally Transferred (high confidence)	AZOBR_70190	Ancestral (low confidence)
AZOLI_1470	Unassigned	AZOBR_70191	Unassigned
AZOLI_1471	Horizontally Transferred (high confidence)	AZOBR_70192	Ancestral (low confidence)

AZOLI_1472	Horizontally Transferred (high confidence)	AZOBR_70193	Horizontally Transferred (low confidence)
AZOLI_1473	Horizontally Transferred (high confidence)	AZOBR_70194	Unassigned
AZOLI_1474	Ancestral (medium confidence)	AZOBR_70195	Ancestral (low confidence)
AZOLI_1475	Ancestral (medium confidence)	AZOBR_70196	Horizontally Transferred (high confidence)
AZOLI_1476	Horizontally Transferred (high confidence)	AZOBR_70197	Horizontally Transferred (high confidence)
AZOLI_1477	Horizontally Transferred (high confidence)	AZOBR_70198	Ancestral (low confidence)
AZOLI_1478	Ancestral (high confidence)	AZOBR_70199	Horizontally Transferred (high confidence)
AZOLI_1479	Ancestral (high confidence)	AZOBR_70200	Unassigned
AZOLI_1480	Ancestral (medium confidence)	AZOBR_70201	Horizontally Transferred (high confidence)
AZOLI_1481	Ancestral (high confidence)	AZOBR_70202	Horizontally Transferred (high confidence)
AZOLI_1482	Ancestral (high confidence)	AZOBR_70203	Unassigned
AZOLI_1483	Ancestral (high confidence)	AZOBR_70204	Horizontally Transferred (low confidence)
AZOLI_1484	Ancestral (high confidence)	AZOBR_70205	Unassigned
AZOLI_1485	Ancestral (high confidence)	AZOBR_70206	Unassigned
AZOLI_1486	Ancestral (high confidence)	AZOBR_70207	Horizontally Transferred (high confidence)
AZOLI_1487	Unassigned	AZOBR_70208	Horizontally Transferred (high confidence)
AZOLI_1488	Ancestral (high confidence)	AZOBR_70209	Ancestral (low confidence)
AZOLI_1489	Ancestral (medium confidence)	AZOBR_70210	Horizontally Transferred (high confidence)
AZOLI_1490	Ancestral (medium confidence)	AZOBR_70211	Unassigned
AZOLI_1491	Horizontally Transferred (high confidence)	AZOBR_70212	Unassigned
AZOLI_1492	Ancestral (high confidence)	AZOBR_70213	Unassigned
AZOLI_1493	Ancestral (medium confidence)	AZOBR_70214	Horizontally Transferred (medium confidence)
AZOLI_1494	Ancestral (medium confidence)	AZOBR_80001	Ancestral (low confidence)
AZOLI_1495	Ancestral (high confidence)	AZOBR_80002	Unassigned
AZOLI_1496	Horizontally Transferred (high confidence)	AZOBR_80003	Unassigned
AZOLI_1497	Ancestral (low confidence)	AZOBR_80004	Ancestral (low confidence)
AZOLI_1498	Ancestral (medium confidence)	AZOBR_80005	Ancestral (medium confidence)
AZOLI_1499	Ancestral (high confidence)	AZOBR_80006	Horizontally Transferred (medium confidence)
AZOLI_1501	Ancestral (medium confidence)	AZOBR_80007	Unassigned
AZOLI_1502	Horizontally Transferred (high confidence)	AZOBR_80008	Unassigned
AZOLI_1503	Ancestral (high confidence)	AZOBR_80009	Unassigned
AZOLI_1504	Ancestral (high confidence)	AZOBR_80010	Horizontally Transferred (high confidence)
AZOLI_1505	Ancestral (medium confidence)	AZOBR_80011	Horizontally Transferred (high confidence)
AZOLI_1506	Horizontally Transferred (high confidence)	AZOBR_80012	Horizontally Transferred (high confidence)
AZOLI_1507	Ancestral (high confidence)	AZOBR_80013	Horizontally Transferred (high confidence)
AZOLI_1508	Ancestral (high confidence)	AZOBR_80014	Ancestral (low confidence)
AZOLI_1509	Ancestral (medium confidence)	AZOBR_80015	Horizontally Transferred (high confidence)
AZOLI_1510	Ancestral (high confidence)	AZOBR_90001	Horizontally Transferred (high confidence)
AZOLI_1511	Ancestral (high confidence)	AZOBR_90002	Unassigned
AZOLI_1512	Ancestral (high confidence)	AZOBR_90003	Ancestral (low confidence)
AZOLI_1513	Ancestral (high confidence)	AZOBR_90004	Unassigned
AZOLI_1515	Ancestral (low confidence)	AZOBR_90005	Unassigned
AZOLI_1516	Ancestral (medium confidence)	AZOBR_90006	Ancestral (high confidence)
AZOLI_1517	Horizontally Transferred (high confidence)	AZOBR_90007	Horizontally Transferred (high confidence)
AZOLI_1518	Horizontally Transferred (high confidence)	AZOBR_90008	Ancestral (high confidence)
AZOLI_1519	Unassigned	AZOBR_90009	Ancestral (low confidence)
AZOLI_1520	Horizontally Transferred (low confidence)	AZOBR_90010	Horizontally Transferred (low confidence)
AZOLI_1521	Horizontally Transferred (high confidence)	AZOBR_90011	Horizontally Transferred (high confidence)
AZOLI_1522	Horizontally Transferred (high confidence)	AZOBR_90012	Horizontally Transferred (high confidence)
AZOLI_1523	Horizontally Transferred (high confidence)	AZOBR_90013	Unassigned
AZOLI_1524	Horizontally Transferred (high confidence)	AZOBR_90014	Unassigned
AZOLI_1525	Ancestral (medium confidence)	AZOBR_90015	Unassigned
AZOLI_1526	Horizontally Transferred (high confidence)	AZOBR_90016	Horizontally Transferred (medium confidence)
AZOLI_1527	Ancestral (high confidence)	AZOBR_90017	Ancestral (low confidence)
AZOLI_1528	Horizontally Transferred (low confidence)	AZOBR_90018	Horizontally Transferred (medium confidence)
AZOLI_1529	Unassigned	AZOBR_90019	Unassigned
AZOLI_1530	Horizontally Transferred (low confidence)	AZOBR_90020	Ancestral (low confidence)
AZOLI_1531	Ancestral (low confidence)	AZOBR_90021	Unassigned
AZOLI_1532	Horizontally Transferred (high confidence)	AZOBR_100001	Ancestral (low confidence)
AZOLI_1533	Ancestral (low confidence)	AZOBR_100002	Ancestral (low confidence)
AZOLI_1534	Ancestral (low confidence)	AZOBR_100003	Unassigned
AZOLI_1535	Unassigned	AZOBR_100004	Ancestral (low confidence)
AZOLI_1536	Unassigned	AZOBR_100005	Horizontally Transferred (high confidence)
AZOLI_1537	Horizontally Transferred (medium confidence)	AZOBR_100006	Horizontally Transferred (high confidence)
AZOLI_1538	Ancestral (high confidence)	AZOBR_100007	Horizontally Transferred (high confidence)
AZOLI_1539	Ancestral (medium confidence)	AZOBR_100008	Horizontally Transferred (high confidence)
AZOLI_1540	Ancestral (medium confidence)	AZOBR_100009	Ancestral (low confidence)
AZOLI_1541	Ancestral (medium confidence)	AZOBR_100010	Ancestral (high confidence)
AZOLI_1543	Unassigned	AZOBR_100011	Ancestral (medium confidence)
AZOLI_1545	Ancestral (medium confidence)	AZOBR_100012	Unassigned
AZOLI_1546	Ancestral (medium confidence)	AZOBR_100013	Ancestral (high confidence)
AZOLI_1547	Ancestral (high confidence)	AZOBR_100014	Ancestral (high confidence)
AZOLI_1548	Ancestral (high confidence)	AZOBR_100015	Ancestral (high confidence)
AZOLI_1549	Horizontally Transferred (high confidence)	AZOBR_100016	Ancestral (medium confidence)
AZOLI_1550	Unassigned	AZOBR_100017	Ancestral (medium confidence)

AZOLI_1551	Ancestral (medium confidence)	AZOBR_100018	Ancestral (low confidence)
AZOLI_1552	Ancestral (medium confidence)	AZOBR_100019	Ancestral (low confidence)
AZOLI_1553	Ancestral (high confidence)	AZOBR_100020	Horizontally Transferred (high confidence)
AZOLI_1554	Ancestral (high confidence)	AZOBR_100021	Unassigned
AZOLI_1555	Horizontally Transferred (high confidence)	AZOBR_100022	Ancestral (low confidence)
AZOLI_1556	Ancestral (low confidence)	AZOBR_100023	Ancestral (low confidence)
AZOLI_1557	Ancestral (medium confidence)	AZOBR_100024	Ancestral (medium confidence)
AZOLI_1558	Ancestral (high confidence)	AZOBR_100025	Ancestral (high confidence)
AZOLI_1559	Ancestral (high confidence)	AZOBR_100026	Unassigned
AZOLI_1560	Ancestral (medium confidence)	AZOBR_100027	Ancestral (high confidence)
AZOLI_1561	Ancestral (high confidence)	AZOBR_100028	Ancestral (high confidence)
AZOLI_1562	Ancestral (low confidence)	AZOBR_100029	Ancestral (high confidence)
AZOLI_1563	Ancestral (high confidence)	AZOBR_100030	Ancestral (medium confidence)
AZOLI_1564	Unassigned	AZOBR_100031	Ancestral (high confidence)
AZOLI_1567	Horizontally Transferred (high confidence)	AZOBR_100032	Ancestral (high confidence)
AZOLI_1568	Ancestral (medium confidence)	AZOBR_100033	Ancestral (high confidence)
AZOLI_1569	Ancestral (medium confidence)	AZOBR_100034	Unassigned
AZOLI_1570	Ancestral (low confidence)	AZOBR_100035	Ancestral (high confidence)
AZOLI_1571	Ancestral (high confidence)	AZOBR_100036	Ancestral (high confidence)
AZOLI_1572	Ancestral (medium confidence)	AZOBR_100037	Ancestral (medium confidence)
AZOLI_1573	Ancestral (medium confidence)	AZOBR_100038	Ancestral (low confidence)
AZOLI_1574	Ancestral (high confidence)	AZOBR_100039	Ancestral (medium confidence)
AZOLI_1575	Unassigned	AZOBR_100040	Horizontally Transferred (high confidence)
AZOLI_1576	Ancestral (medium confidence)	AZOBR_100041	Ancestral (medium confidence)
AZOLI_1577	Ancestral (high confidence)	AZOBR_100042	Ancestral (high confidence)
AZOLI_1578	Unassigned	AZOBR_100043	Ancestral (high confidence)
AZOLI_1579	Unassigned	AZOBR_100044	Ancestral (medium confidence)
AZOLI_1580	Horizontally Transferred (high confidence)	AZOBR_100045	Ancestral (medium confidence)
AZOLI_1581	Unassigned	AZOBR_100046	Horizontally Transferred (medium confidence)
AZOLI_1582	Unassigned	AZOBR_100047	Horizontally Transferred (low confidence)
AZOLI_1583	Unassigned	AZOBR_100048	Ancestral (high confidence)
AZOLI_1584	Ancestral (high confidence)	AZOBR_100049	Ancestral (high confidence)
AZOLI_1585	Ancestral (high confidence)	AZOBR_100050	Ancestral (medium confidence)
AZOLI_1586	Unassigned	AZOBR_100051	Ancestral (medium confidence)
AZOLI_1588	Unassigned	AZOBR_100052	Horizontally Transferred (high confidence)
AZOLI_1589	Ancestral (low confidence)	AZOBR_100053	Horizontally Transferred (high confidence)
AZOLI_1590	Horizontally Transferred (high confidence)	AZOBR_100054	Unassigned
AZOLI_1591	Ancestral (medium confidence)	AZOBR_100055	Ancestral (high confidence)
AZOLI_1592	Ancestral (medium confidence)	AZOBR_100056	Horizontally Transferred (high confidence)
AZOLI_1593	Ancestral (high confidence)	AZOBR_100057	Horizontally Transferred (high confidence)
AZOLI_1595	Ancestral (high confidence)	AZOBR_100058	Horizontally Transferred (high confidence)
AZOLI_1596	Ancestral (medium confidence)	AZOBR_100059	Ancestral (high confidence)
AZOLI_1597	Ancestral (high confidence)	AZOBR_100060	Unassigned
AZOLI_1598	Ancestral (medium confidence)	AZOBR_100061	Horizontally Transferred (high confidence)
AZOLI_1599	Horizontally Transferred (high confidence)	AZOBR_100062	Horizontally Transferred (high confidence)
AZOLI_1600	Ancestral (medium confidence)	AZOBR_100063	Unassigned
AZOLI_1601	Ancestral (medium confidence)	AZOBR_100064	Ancestral (low confidence)
AZOLI_1602	Ancestral (medium confidence)	AZOBR_100065	Unassigned
AZOLI_1603	Ancestral (low confidence)	AZOBR_100066	Ancestral (medium confidence)
AZOLI_1604	Ancestral (medium confidence)	AZOBR_100067	Horizontally Transferred (high confidence)
AZOLI_1605	Ancestral (high confidence)	AZOBR_100068	Ancestral (low confidence)
AZOLI_1606	Ancestral (high confidence)	AZOBR_100069	Ancestral (medium confidence)
AZOLI_1608	Horizontally Transferred (medium confidence)	AZOBR_100070	Ancestral (high confidence)
AZOLI_1609	Ancestral (medium confidence)	AZOBR_100071	Ancestral (low confidence)
AZOLI_1610	Ancestral (medium confidence)	AZOBR_100072	Ancestral (medium confidence)
AZOLI_1611	Ancestral (high confidence)	AZOBR_100073	Ancestral (medium confidence)
AZOLI_1612	Horizontally Transferred (high confidence)	AZOBR_100074	Unassigned
AZOLI_1613	Ancestral (high confidence)	AZOBR_100075	Ancestral (medium confidence)
AZOLI_1614	Ancestral (medium confidence)	AZOBR_100076	Ancestral (low confidence)
AZOLI_1615	Horizontally Transferred (high confidence)	AZOBR_100077	Ancestral (low confidence)
AZOLI_1616	Horizontally Transferred (high confidence)	AZOBR_100078	Horizontally Transferred (high confidence)
AZOLI_1617	Horizontally Transferred (high confidence)	AZOBR_100079	Ancestral (medium confidence)
AZOLI_1618	Ancestral (medium confidence)	AZOBR_100080	Ancestral (low confidence)
AZOLI_1619	Ancestral (low confidence)	AZOBR_100081	Ancestral (high confidence)
AZOLI_1621	Ancestral (medium confidence)	AZOBR_100082	Ancestral (medium confidence)
AZOLI_1622	Ancestral (low confidence)	AZOBR_100083	Horizontally Transferred (high confidence)
AZOLI_1623	Ancestral (medium confidence)	AZOBR_100084	Ancestral (medium confidence)
AZOLI_1624	Ancestral (medium confidence)	AZOBR_100085	Ancestral (high confidence)
AZOLI_1625	Ancestral (low confidence)	AZOBR_100086	Unassigned
AZOLI_1626	Ancestral (high confidence)	AZOBR_100087	Ancestral (low confidence)
AZOLI_1627	Ancestral (high confidence)	AZOBR_100088	Ancestral (high confidence)
AZOLI_1628	Ancestral (medium confidence)	AZOBR_100089	Ancestral (medium confidence)
AZOLI_1629	Ancestral (high confidence)	AZOBR_100090	Ancestral (medium confidence)
AZOLI_1630	Ancestral (medium confidence)	AZOBR_100091	Horizontally Transferred (low confidence)
AZOLI_1631	Ancestral (high confidence)	AZOBR_100092	Ancestral (medium confidence)

AZOLI_1632	Ancestral (medium confidence)	AZOBR_100093	Horizontally Transferred (high confidence)
AZOLI_1633	Horizontally Transferred (high confidence)	AZOBR_100094	Unassigned
AZOLI_1634	Horizontally Transferred (high confidence)	AZOBR_100095	Horizontally Transferred (high confidence)
AZOLI_1635	Horizontally Transferred (high confidence)	AZOBR_100096	Horizontally Transferred (high confidence)
AZOLI_1636	Horizontally Transferred (high confidence)	AZOBR_100097	Horizontally Transferred (high confidence)
AZOLI_1638	Ancestral (low confidence)	AZOBR_100098	Horizontally Transferred (low confidence)
AZOLI_1639	Ancestral (medium confidence)	AZOBR_100099	Ancestral (high confidence)
AZOLI_1640	Unassigned	AZOBR_100100	Unassigned
AZOLI_1641	Ancestral (medium confidence)	AZOBR_100101	Ancestral (medium confidence)
AZOLI_1642	Ancestral (high confidence)	AZOBR_100102	Ancestral (high confidence)
AZOLI_1643	Ancestral (medium confidence)	AZOBR_100103	Ancestral (high confidence)
AZOLI_1644	Ancestral (medium confidence)	AZOBR_100104	Ancestral (medium confidence)
AZOLI_1645	Horizontally Transferred (high confidence)	AZOBR_100105	Ancestral (high confidence)
AZOLI_1646	Ancestral (medium confidence)	AZOBR_100106	Ancestral (high confidence)
AZOLI_1647	Ancestral (low confidence)	AZOBR_100107	Ancestral (low confidence)
AZOLI_1648	Horizontally Transferred (low confidence)	AZOBR_100108	Ancestral (high confidence)
AZOLI_1649	Horizontally Transferred (low confidence)	AZOBR_100109	Horizontally Transferred (high confidence)
AZOLI_1651	Horizontally Transferred (high confidence)	AZOBR_100110	Ancestral (high confidence)
AZOLI_1653	Horizontally Transferred (high confidence)	AZOBR_100111	Ancestral (medium confidence)
AZOLI_1654	Ancestral (medium confidence)	AZOBR_100112	Ancestral (high confidence)
AZOLI_1656	Horizontally Transferred (high confidence)	AZOBR_100113	Ancestral (high confidence)
AZOLI_1657	Ancestral (high confidence)	AZOBR_100114	Horizontally Transferred (high confidence)
AZOLI_1658	Ancestral (high confidence)	AZOBR_100115	Unassigned
AZOLI_1659	Unassigned	AZOBR_100116	Ancestral (high confidence)
AZOLI_1660	Horizontally Transferred (high confidence)	AZOBR_100117	Ancestral (high confidence)
AZOLI_1661	Horizontally Transferred (high confidence)	AZOBR_100118	Ancestral (high confidence)
AZOLI_1662	Horizontally Transferred (high confidence)	AZOBR_100119	Ancestral (high confidence)
AZOLI_1663	Horizontally Transferred (high confidence)	AZOBR_100120	Ancestral (medium confidence)
AZOLI_1664	Horizontally Transferred (high confidence)	AZOBR_100121	Unassigned
AZOLI_1665	Horizontally Transferred (high confidence)	AZOBR_100122	Horizontally Transferred (high confidence)
AZOLI_1666	Ancestral (medium confidence)	AZOBR_100123	Ancestral (medium confidence)
AZOLI_1667	Ancestral (medium confidence)	AZOBR_100124	Ancestral (medium confidence)
AZOLI_1668	Ancestral (medium confidence)	AZOBR_100125	Unassigned
AZOLI_1669	Ancestral (high confidence)	AZOBR_100126	Ancestral (medium confidence)
AZOLI_1670	Ancestral (high confidence)	AZOBR_100127	Ancestral (low confidence)
AZOLI_1671	Ancestral (high confidence)	AZOBR_100128	Ancestral (low confidence)
AZOLI_1672	Unassigned	AZOBR_100129	Horizontally Transferred (high confidence)
AZOLI_1673	Ancestral (high confidence)	AZOBR_100130	Horizontally Transferred (high confidence)
AZOLI_1674	Ancestral (high confidence)	AZOBR_100131	Ancestral (medium confidence)
AZOLI_1675	Horizontally Transferred (high confidence)	AZOBR_100132	Unassigned
AZOLI_1676	Ancestral (medium confidence)	AZOBR_100133	Horizontally Transferred (high confidence)
AZOLI_1677	Ancestral (low confidence)	AZOBR_100134	Ancestral (high confidence)
AZOLI_1678	Ancestral (high confidence)	AZOBR_100135	Unassigned
AZOLI_1679	Ancestral (high confidence)	AZOBR_100136	Ancestral (low confidence)
AZOLI_1680	Ancestral (medium confidence)	AZOBR_100137	Ancestral (low confidence)
AZOLI_1681	Ancestral (high confidence)	AZOBR_100138	Ancestral (low confidence)
AZOLI_1682	Horizontally Transferred (high confidence)	AZOBR_100139	Ancestral (low confidence)
AZOLI_1683	Unassigned	AZOBR_100140	Unassigned
AZOLI_1684	Ancestral (low confidence)	AZOBR_100141	Ancestral (medium confidence)
AZOLI_1686	Horizontally Transferred (medium confidence)	AZOBR_100142	Horizontally Transferred (medium confidence)
AZOLI_1687	Ancestral (medium confidence)	AZOBR_100143	Horizontally Transferred (high confidence)
AZOLI_1688	Ancestral (medium confidence)	AZOBR_100144	Horizontally Transferred (high confidence)
AZOLI_1689	Unassigned	AZOBR_100145	Ancestral (medium confidence)
AZOLI_1690	Ancestral (low confidence)	AZOBR_100146	Ancestral (low confidence)
AZOLI_1691	Horizontally Transferred (high confidence)	AZOBR_100147	Horizontally Transferred (high confidence)
AZOLI_1692	Horizontally Transferred (high confidence)	AZOBR_100148	Horizontally Transferred (high confidence)
AZOLI_1693	Unassigned	AZOBR_100149	Ancestral (medium confidence)
AZOLI_1694	Horizontally Transferred (high confidence)	AZOBR_100150	Ancestral (medium confidence)
AZOLI_1701	Unassigned	AZOBR_100151	Ancestral (medium confidence)
AZOLI_1703	Ancestral (medium confidence)	AZOBR_100152	Ancestral (low confidence)
AZOLI_1704	Horizontally Transferred (high confidence)	AZOBR_100153	Ancestral (low confidence)
AZOLI_1705	Ancestral (high confidence)	AZOBR_100154	Unassigned
AZOLI_1707	Unassigned	AZOBR_100155	Unassigned
AZOLI_1709	Ancestral (high confidence)	AZOBR_100156	Horizontally Transferred (high confidence)
AZOLI_1711	Unassigned	AZOBR_100157	Horizontally Transferred (low confidence)
AZOLI_1713	Ancestral (high confidence)	AZOBR_100158	Horizontally Transferred (low confidence)
AZOLI_1715	Horizontally Transferred (high confidence)	AZOBR_100159	Ancestral (low confidence)
AZOLI_1716	Unassigned	AZOBR_100160	Ancestral (low confidence)
AZOLI_1717	Horizontally Transferred (high confidence)	AZOBR_100161	Ancestral (medium confidence)
AZOLI_1718	Ancestral (low confidence)	AZOBR_100162	Ancestral (low confidence)
AZOLI_1719	Ancestral (high confidence)	AZOBR_100163	Horizontally Transferred (high confidence)
AZOLI_1721	Ancestral (high confidence)	AZOBR_100164	Ancestral (low confidence)
AZOLI_1722	Ancestral (high confidence)	AZOBR_100165	Ancestral (low confidence)
AZOLI_1723	Ancestral (high confidence)	AZOBR_100166	Unassigned
AZOLI_1724	Ancestral (high confidence)	AZOBR_100167	Horizontally Transferred (high confidence)

AZOLI_1725	Ancestral (high confidence)	AZOBR_100168	Ancestral (high confidence)
AZOLI_1726	Ancestral (high confidence)	AZOBR_100169	Horizontally Transferred (high confidence)
AZOLI_1727	Ancestral (medium confidence)	AZOBR_100170	Ancestral (medium confidence)
AZOLI_1728	Ancestral (low confidence)	AZOBR_100171	Ancestral (high confidence)
AZOLI_1729	Ancestral (medium confidence)	AZOBR_100172	Ancestral (low confidence)
AZOLI_1731	Ancestral (high confidence)	AZOBR_100173	Horizontally Transferred (high confidence)
AZOLI_1732	Ancestral (high confidence)	AZOBR_100174	Ancestral (medium confidence)
AZOLI_1733	Ancestral (high confidence)	AZOBR_100175	Ancestral (high confidence)
AZOLI_1734	Ancestral (high confidence)	AZOBR_100176	Ancestral (medium confidence)
AZOLI_1735	Ancestral (high confidence)	AZOBR_100177	Ancestral (medium confidence)
AZOLI_1736	Ancestral (high confidence)	AZOBR_100178	Ancestral (high confidence)
AZOLI_1737	Unassigned	AZOBR_100179	Ancestral (high confidence)
AZOLI_1738	Ancestral (high confidence)	AZOBR_100180	Ancestral (medium confidence)
AZOLI_1739	Ancestral (high confidence)	AZOBR_100181	Horizontally Transferred (high confidence)
AZOLI_1740	Ancestral (high confidence)	AZOBR_100182	Unassigned
AZOLI_1741	Ancestral (medium confidence)	AZOBR_100183	Ancestral (high confidence)
AZOLI_1743	Unassigned	AZOBR_100184	Horizontally Transferred (high confidence)
AZOLI_1744	Unassigned	AZOBR_100185	Ancestral (medium confidence)
AZOLI_1748	Ancestral (low confidence)	AZOBR_100186	Ancestral (medium confidence)
AZOLI_1751	Ancestral (low confidence)	AZOBR_100187	Ancestral (high confidence)
AZOLI_1752	Ancestral (medium confidence)	AZOBR_100188	Ancestral (low confidence)
AZOLI_1753	Unassigned	AZOBR_100189	Ancestral (low confidence)
AZOLI_1755	Horizontally Transferred (high confidence)	AZOBR_100190	Ancestral (high confidence)
AZOLI_1756	Horizontally Transferred (high confidence)	AZOBR_100191	Ancestral (high confidence)
AZOLI_1757	Ancestral (medium confidence)	AZOBR_100192	Ancestral (low confidence)
AZOLI_1758	Unassigned	AZOBR_100193	Ancestral (low confidence)
AZOLI_1759	Unassigned	AZOBR_100194	Unassigned
AZOLI_1760	Ancestral (low confidence)	AZOBR_100195	Ancestral (high confidence)
AZOLI_1761	Ancestral (medium confidence)	AZOBR_100196	Unassigned
AZOLI_1762	Ancestral (low confidence)	AZOBR_100197	Horizontally Transferred (high confidence)
AZOLI_1763	Horizontally Transferred (high confidence)	AZOBR_100198	Ancestral (low confidence)
AZOLI_1764	Unassigned	AZOBR_100199	Ancestral (low confidence)
AZOLI_1765	Unassigned	AZOBR_100200	Ancestral (low confidence)
AZOLI_1766	Horizontally Transferred (high confidence)	AZOBR_100201	Ancestral (low confidence)
AZOLI_1767	Unassigned	AZOBR_100202	Horizontally Transferred (high confidence)
AZOLI_1768	Unassigned	AZOBR_100203	Horizontally Transferred (low confidence)
AZOLI_1769	Ancestral (high confidence)	AZOBR_100204	Unassigned
AZOLI_1770	Ancestral (low confidence)	AZOBR_100205	Unassigned
AZOLI_1771	Ancestral (low confidence)	AZOBR_100206	Horizontally Transferred (high confidence)
AZOLI_1772	Ancestral (low confidence)	AZOBR_100207	Horizontally Transferred (high confidence)
AZOLI_1773	Unassigned	AZOBR_100208	Ancestral (low confidence)
AZOLI_1774	Ancestral (medium confidence)	AZOBR_100209	Ancestral (medium confidence)
AZOLI_1775	Ancestral (low confidence)	AZOBR_100210	Horizontally Transferred (high confidence)
AZOLI_1776	Unassigned	AZOBR_100211	Horizontally Transferred (high confidence)
AZOLI_1777	Ancestral (medium confidence)	AZOBR_100212	Horizontally Transferred (high confidence)
AZOLI_1778	Horizontally Transferred (high confidence)	AZOBR_100213	Unassigned
AZOLI_1779	Unassigned	AZOBR_100214	Horizontally Transferred (high confidence)
AZOLI_1780	Unassigned	AZOBR_100215	Ancestral (high confidence)
AZOLI_1781	Horizontally Transferred (low confidence)	AZOBR_100216	Ancestral (high confidence)
AZOLI_1782	Ancestral (high confidence)	AZOBR_100217	Ancestral (high confidence)
AZOLI_1783	Unassigned	AZOBR_100218	Ancestral (low confidence)
AZOLI_1784	Unassigned	AZOBR_100219	Horizontally Transferred (high confidence)
AZOLI_1785	Ancestral (low confidence)	AZOBR_100220	Ancestral (low confidence)
AZOLI_1786	Unassigned	AZOBR_100221	Ancestral (low confidence)
AZOLI_1787	Unassigned	AZOBR_100222	Unassigned
AZOLI_1789	Unassigned	AZOBR_100223	Ancestral (medium confidence)
AZOLI_1790	Unassigned	AZOBR_100224	Ancestral (medium confidence)
AZOLI_1791	Horizontally Transferred (medium confidence)	AZOBR_100225	Ancestral (low confidence)
AZOLI_1792	Horizontally Transferred (medium confidence)	AZOBR_100226	Ancestral (low confidence)
AZOLI_1793	Ancestral (low confidence)	AZOBR_100227	Ancestral (low confidence)
AZOLI_1794	Ancestral (low confidence)	AZOBR_100228	Ancestral (high confidence)
AZOLI_1795	Unassigned	AZOBR_100229	Unassigned
AZOLI_1796	Unassigned	AZOBR_100230	Ancestral (high confidence)
AZOLI_1797	Ancestral (low confidence)	AZOBR_100231	Unassigned
AZOLI_1798	Ancestral (high confidence)	AZOBR_100232	Unassigned
AZOLI_1799	Ancestral (high confidence)	AZOBR_100233	Ancestral (low confidence)
AZOLI_1800	Ancestral (high confidence)	AZOBR_100234	Ancestral (high confidence)
AZOLI_1801	Ancestral (high confidence)	AZOBR_100235	Ancestral (high confidence)
AZOLI_1802	Ancestral (medium confidence)	AZOBR_100236	Ancestral (high confidence)
AZOLI_1803	Ancestral (high confidence)	AZOBR_100237	Ancestral (high confidence)
AZOLI_1804	Ancestral (high confidence)	AZOBR_100238	Unassigned
AZOLI_1805	Ancestral (low confidence)	AZOBR_100239	Ancestral (high confidence)
AZOLI_1806	Ancestral (low confidence)	AZOBR_100240	Unassigned
AZOLI_1807	Unassigned	AZOBR_100241	Ancestral (high confidence)
AZOLI_1808	Ancestral (medium confidence)	AZOBR_100242	Unassigned

AZOLI_1810	Horizontally Transferred (high confidence)	AZOBR_100243	Ancestral (high confidence)
AZOLI_1811	Ancestral (low confidence)	AZOBR_100244	Ancestral (high confidence)
AZOLI_1812	Ancestral (low confidence)	AZOBR_100245	Ancestral (high confidence)
AZOLI_1814	Ancestral (high confidence)	AZOBR_100246	Ancestral (high confidence)
AZOLI_1815	Ancestral (high confidence)	AZOBR_100247	Ancestral (high confidence)
AZOLI_1816	Unassigned	AZOBR_100248	Ancestral (medium confidence)
AZOLI_1817	Ancestral (high confidence)	AZOBR_100249	Ancestral (high confidence)
AZOLI_1818	Ancestral (high confidence)	AZOBR_100250	Ancestral (high confidence)
AZOLI_1819	Ancestral (high confidence)	AZOBR_100251	Ancestral (high confidence)
AZOLI_1820	Horizontally Transferred (low confidence)	AZOBR_100252	Ancestral (medium confidence)
AZOLI_1821	Horizontally Transferred (low confidence)	AZOBR_100253	Ancestral (medium confidence)
AZOLI_1822	Ancestral (high confidence)	AZOBR_100254	Ancestral (high confidence)
AZOLI_1823	Ancestral (medium confidence)	AZOBR_100255	Ancestral (high confidence)
AZOLI_1824	Ancestral (high confidence)	AZOBR_100256	Ancestral (high confidence)
AZOLI_1825	Horizontally Transferred (high confidence)	AZOBR_100257	Ancestral (medium confidence)
AZOLI_1826	Horizontally Transferred (high confidence)	AZOBR_100258	Ancestral (high confidence)
AZOLI_1827	Horizontally Transferred (high confidence)	AZOBR_100259	Ancestral (high confidence)
AZOLI_1829	Horizontally Transferred (high confidence)	AZOBR_100260	Ancestral (high confidence)
AZOLI_1830	Ancestral (low confidence)	AZOBR_100261	Ancestral (medium confidence)
AZOLI_1831	Horizontally Transferred (high confidence)	AZOBR_100262	Ancestral (medium confidence)
AZOLI_1832	Horizontally Transferred (high confidence)	AZOBR_100263	Ancestral (medium confidence)
AZOLI_1833	Ancestral (low confidence)	AZOBR_100264	Ancestral (medium confidence)
AZOLI_1834	Ancestral (medium confidence)	AZOBR_100265	Ancestral (medium confidence)
AZOLI_1835	Ancestral (medium confidence)	AZOBR_100266	Ancestral (high confidence)
AZOLI_1836	Ancestral (medium confidence)	AZOBR_100267	Ancestral (high confidence)
AZOLI_1837	Ancestral (high confidence)	AZOBR_100268	Horizontally Transferred (medium confidence)
AZOLI_1838	Horizontally Transferred (high confidence)	AZOBR_100269	Ancestral (low confidence)
AZOLI_1839	Ancestral (high confidence)	AZOBR_100270	Horizontally Transferred (high confidence)
AZOLI_1840	Unassigned	AZOBR_100271	Ancestral (high confidence)
AZOLI_1841	Horizontally Transferred (high confidence)	AZOBR_100272	Unassigned
AZOLI_1842	Ancestral (low confidence)	AZOBR_100273	Ancestral (low confidence)
AZOLI_1843	Ancestral (medium confidence)	AZOBR_100274	Ancestral (high confidence)
AZOLI_1844	Ancestral (high confidence)	AZOBR_100275	Ancestral (low confidence)
AZOLI_1845	Ancestral (medium confidence)	AZOBR_100276	Ancestral (high confidence)
AZOLI_1846	Ancestral (medium confidence)	AZOBR_100277	Ancestral (high confidence)
AZOLI_1847	Horizontally Transferred (high confidence)	AZOBR_100278	Ancestral (medium confidence)
AZOLI_1849	Ancestral (medium confidence)	AZOBR_100279	Ancestral (high confidence)
AZOLI_1851	Ancestral (high confidence)	AZOBR_100280	Ancestral (high confidence)
AZOLI_1852	Ancestral (low confidence)	AZOBR_100281	Ancestral (high confidence)
AZOLI_1853	Ancestral (high confidence)	AZOBR_100282	Ancestral (high confidence)
AZOLI_1854	Ancestral (low confidence)	AZOBR_100283	Ancestral (high confidence)
AZOLI_1855	Horizontally Transferred (low confidence)	AZOBR_100284	Ancestral (high confidence)
AZOLI_1856	Ancestral (high confidence)	AZOBR_100285	Ancestral (high confidence)
AZOLI_1857	Ancestral (high confidence)	AZOBR_100286	Ancestral (high confidence)
AZOLI_1858	Ancestral (high confidence)	AZOBR_100287	Ancestral (high confidence)
AZOLI_1859	Unassigned	AZOBR_100288	Ancestral (high confidence)
AZOLI_1860	Ancestral (high confidence)	AZOBR_100289	Ancestral (low confidence)
AZOLI_1861	Ancestral (high confidence)	AZOBR_100290	Horizontally Transferred (high confidence)
AZOLI_1862	Unassigned	AZOBR_100291	Horizontally Transferred (high confidence)
AZOLI_1863	Ancestral (high confidence)	AZOBR_100292	Ancestral (low confidence)
AZOLI_1864	Ancestral (low confidence)	AZOBR_100293	Unassigned
AZOLI_1865	Ancestral (high confidence)	AZOBR_100294	Ancestral (medium confidence)
AZOLI_1867	Ancestral (high confidence)	AZOBR_100295	Ancestral (medium confidence)
AZOLI_1868	Horizontally Transferred (high confidence)	AZOBR_100296	Ancestral (high confidence)
AZOLI_1869	Horizontally Transferred (high confidence)	AZOBR_100297	Ancestral (high confidence)
AZOLI_1870	Horizontally Transferred (high confidence)	AZOBR_100298	Ancestral (high confidence)
AZOLI_1872	Ancestral (medium confidence)	AZOBR_100299	Ancestral (high confidence)
AZOLI_1873	Ancestral (high confidence)	AZOBR_100300	Ancestral (high confidence)
AZOLI_1874	Horizontally Transferred (high confidence)	AZOBR_100301	Ancestral (high confidence)
AZOLI_1875	Horizontally Transferred (high confidence)	AZOBR_100302	Unassigned
AZOLI_1876	Ancestral (high confidence)	AZOBR_100303	Ancestral (medium confidence)
AZOLI_1877	Unassigned	AZOBR_100304	Ancestral (medium confidence)
AZOLI_1878	Horizontally Transferred (low confidence)	AZOBR_100305	Ancestral (high confidence)
AZOLI_1881	Ancestral (high confidence)	AZOBR_100306	Horizontally Transferred (high confidence)
AZOLI_1882	Ancestral (medium confidence)	AZOBR_100307	Ancestral (high confidence)
AZOLI_1883	Ancestral (high confidence)	AZOBR_100308	Ancestral (high confidence)
AZOLI_1884	Ancestral (medium confidence)	AZOBR_100309	Unassigned
AZOLI_1885	Ancestral (high confidence)	AZOBR_100310	Ancestral (low confidence)
AZOLI_1886	Ancestral (medium confidence)	AZOBR_100311	Ancestral (high confidence)
AZOLI_1888	Unassigned	AZOBR_100312	Ancestral (high confidence)
AZOLI_1889	Ancestral (medium confidence)	AZOBR_100313	Unassigned
AZOLI_1890	Ancestral (medium confidence)	AZOBR_100314	Ancestral (low confidence)
AZOLI_1891	Ancestral (medium confidence)	AZOBR_100315	Ancestral (medium confidence)
AZOLI_1892	Ancestral (medium confidence)	AZOBR_100316	Ancestral (high confidence)
AZOLI_1893	Ancestral (low confidence)	AZOBR_100317	Ancestral (medium confidence)

AZOLI_1894	Ancestral (high confidence)	AZOBR_100318	Ancestral (medium confidence)
AZOLI_1895	Horizontally Transferred (high confidence)	AZOBR_100319	Horizontally Transferred (medium confidence)
AZOLI_1896	Ancestral (high confidence)	AZOBR_100320	Horizontally Transferred (low confidence)
AZOLI_1897	Ancestral (medium confidence)	AZOBR_100321	Horizontally Transferred (low confidence)
AZOLI_1898	Ancestral (high confidence)	AZOBR_100322	Ancestral (low confidence)
AZOLI_1899	Ancestral (low confidence)	AZOBR_100323	Ancestral (low confidence)
AZOLI_1900	Ancestral (low confidence)	AZOBR_100324	Horizontally Transferred (high confidence)
AZOLI_1901	Ancestral (high confidence)	AZOBR_100325	Ancestral (high confidence)
AZOLI_1902	Ancestral (high confidence)	AZOBR_100326	Ancestral (high confidence)
AZOLI_1903	Unassigned	AZOBR_100327	Ancestral (high confidence)
AZOLI_1904	Ancestral (medium confidence)	AZOBR_100328	Ancestral (high confidence)
AZOLI_1905	Ancestral (medium confidence)	AZOBR_100329	Ancestral (high confidence)
AZOLI_1906	Horizontally Transferred (high confidence)	AZOBR_100330	Ancestral (low confidence)
AZOLI_1907	Ancestral (low confidence)	AZOBR_100331	Horizontally Transferred (high confidence)
AZOLI_1908	Unassigned	AZOBR_100332	Unassigned
AZOLI_1909	Ancestral (high confidence)	AZOBR_100333	Horizontally Transferred (high confidence)
AZOLI_1910	Ancestral (high confidence)	AZOBR_100334	Ancestral (low confidence)
AZOLI_1911	Unassigned	AZOBR_100335	Horizontally Transferred (medium confidence)
AZOLI_1912	Ancestral (high confidence)	AZOBR_100336	Ancestral (medium confidence)
AZOLI_1913	Ancestral (medium confidence)	AZOBR_100337	Ancestral (low confidence)
AZOLI_1914	Ancestral (medium confidence)	AZOBR_100338	Horizontally Transferred (high confidence)
AZOLI_1915	Ancestral (high confidence)	AZOBR_100339	Unassigned
AZOLI_1916	Ancestral (medium confidence)	AZOBR_100340	Ancestral (medium confidence)
AZOLI_1917	Ancestral (high confidence)	AZOBR_100341	Ancestral (medium confidence)
AZOLI_1918	Ancestral (high confidence)	AZOBR_100342	Ancestral (medium confidence)
AZOLI_1919	Ancestral (medium confidence)	AZOBR_100343	Ancestral (medium confidence)
AZOLI_1920	Unassigned	AZOBR_100344	Horizontally Transferred (medium confidence)
AZOLI_1921	Ancestral (low confidence)	AZOBR_100345	Ancestral (high confidence)
AZOLI_1922	Ancestral (high confidence)	AZOBR_100346	Ancestral (medium confidence)
AZOLI_1924	Unassigned	AZOBR_100347	Horizontally Transferred (medium confidence)
AZOLI_1925	Horizontally Transferred (high confidence)	AZOBR_100348	Ancestral (low confidence)
AZOLI_1926	Horizontally Transferred (high confidence)	AZOBR_100349	Ancestral (medium confidence)
AZOLI_1927	Horizontally Transferred (high confidence)	AZOBR_100350	Horizontally Transferred (high confidence)
AZOLI_1928	Ancestral (low confidence)	AZOBR_100351	Horizontally Transferred (high confidence)
AZOLI_1930	Horizontally Transferred (high confidence)	AZOBR_100352	Horizontally Transferred (high confidence)
AZOLI_1931	Ancestral (high confidence)	AZOBR_100353	Horizontally Transferred (high confidence)
AZOLI_1932	Unassigned	AZOBR_100354	Horizontally Transferred (high confidence)
AZOLI_1936	Unassigned	AZOBR_100355	Ancestral (low confidence)
AZOLI_1938	Unassigned	AZOBR_100356	Ancestral (high confidence)
AZOLI_1939	Horizontally Transferred (low confidence)	AZOBR_100357	Ancestral (medium confidence)
AZOLI_1940	Unassigned	AZOBR_100358	Ancestral (low confidence)
AZOLI_1941	Unassigned	AZOBR_100359	Ancestral (low confidence)
AZOLI_1942	Horizontally Transferred (high confidence)	AZOBR_100360	Ancestral (medium confidence)
AZOLI_1943	Unassigned	AZOBR_100361	Ancestral (high confidence)
AZOLI_1944	Horizontally Transferred (high confidence)	AZOBR_100362	Unassigned
AZOLI_1945	Unassigned	AZOBR_100363	Horizontally Transferred (high confidence)
AZOLI_1946	Ancestral (high confidence)	AZOBR_100364	Ancestral (low confidence)
AZOLI_1947	Ancestral (high confidence)	AZOBR_100365	Unassigned
AZOLI_1948	Unassigned	AZOBR_100366	Unassigned
AZOLI_1949	Ancestral (low confidence)	AZOBR_100367	Unassigned
AZOLI_1950	Horizontally Transferred (high confidence)	AZOBR_100368	Ancestral (medium confidence)
AZOLI_1951	Ancestral (high confidence)	AZOBR_100369	Ancestral (medium confidence)
AZOLI_1952	Ancestral (high confidence)	AZOBR_100370	Ancestral (medium confidence)
AZOLI_1953	Horizontally Transferred (low confidence)	AZOBR_100371	Ancestral (high confidence)
AZOLI_1954	Ancestral (low confidence)	AZOBR_100372	Ancestral (medium confidence)
AZOLI_1955	Ancestral (low confidence)	AZOBR_100373	Ancestral (high confidence)
AZOLI_1956	Horizontally Transferred (high confidence)	AZOBR_100374	Ancestral (medium confidence)
AZOLI_1957	Ancestral (low confidence)	AZOBR_100375	Ancestral (high confidence)
AZOLI_1958	Horizontally Transferred (high confidence)	AZOBR_100376	Unassigned
AZOLI_1959	Unassigned	AZOBR_100377	Ancestral (medium confidence)
AZOLI_1960	Ancestral (medium confidence)	AZOBR_100378	Ancestral (high confidence)
AZOLI_1961	Ancestral (low confidence)	AZOBR_100379	Ancestral (low confidence)
AZOLI_1962	Ancestral (high confidence)	AZOBR_100380	Ancestral (high confidence)
AZOLI_1963	Ancestral (low confidence)	AZOBR_100381	Ancestral (medium confidence)
AZOLI_1964	Horizontally Transferred (high confidence)	AZOBR_100382	Ancestral (medium confidence)
AZOLI_1965	Ancestral (low confidence)	AZOBR_100383	Ancestral (high confidence)
AZOLI_1966	Horizontally Transferred (high confidence)	AZOBR_100384	Horizontally Transferred (high confidence)
AZOLI_1967	Horizontally Transferred (high confidence)	AZOBR_100385	Horizontally Transferred (high confidence)
AZOLI_1968	Horizontally Transferred (high confidence)	AZOBR_100386	Unassigned
AZOLI_1969	Ancestral (high confidence)	AZOBR_100387	Unassigned
AZOLI_1970	Ancestral (high confidence)	AZOBR_100388	Ancestral (high confidence)
AZOLI_1971	Unassigned	AZOBR_100389	Ancestral (medium confidence)
AZOLI_1972	Ancestral (high confidence)	AZOBR_100390	Ancestral (medium confidence)
AZOLI_1973	Ancestral (high confidence)	AZOBR_100391	Horizontally Transferred (high confidence)
AZOLI_1974	Ancestral (high confidence)	AZOBR_100392	Horizontally Transferred (medium confidence)

AZOLI_1975	Ancestral (high confidence)	AZOBR_100393	Horizontally Transferred (high confidence)
AZOLI_1976	Ancestral (high confidence)	AZOBR_100394	Unassigned
AZOLI_1977	Unassigned	AZOBR_100395	Unassigned
AZOLI_1978	Ancestral (high confidence)	AZOBR_100396	Ancestral (low confidence)
AZOLI_1979	Ancestral (high confidence)	AZOBR_100397	Ancestral (high confidence)
AZOLI_1980	Ancestral (low confidence)	AZOBR_100398	Ancestral (high confidence)
AZOLI_1981	Horizontally Transferred (medium confidence)	AZOBR_100399	Horizontally Transferred (high confidence)
AZOLI_1982	Horizontally Transferred (low confidence)	AZOBR_100400	Horizontally Transferred (high confidence)
AZOLI_1984	Horizontally Transferred (high confidence)	AZOBR_100401	Unassigned
AZOLI_1985	Horizontally Transferred (high confidence)	AZOBR_100402	Horizontally Transferred (high confidence)
AZOLI_1986	Horizontally Transferred (high confidence)	AZOBR_100403	Unassigned
AZOLI_1987	Unassigned	AZOBR_100404	Ancestral (low confidence)
AZOLI_1988	Horizontally Transferred (medium confidence)	AZOBR_100405	Horizontally Transferred (high confidence)
AZOLI_1989	Unassigned	AZOBR_100406	Horizontally Transferred (high confidence)
AZOLI_1994	Horizontally Transferred (high confidence)	AZOBR_100407	Unassigned
AZOLI_1995	Unassigned	AZOBR_100408	Ancestral (low confidence)
AZOLI_1996	Horizontally Transferred (high confidence)	AZOBR_100409	Ancestral (high confidence)
AZOLI_1997	Ancestral (high confidence)	AZOBR_100410	Ancestral (medium confidence)
AZOLI_1998	Ancestral (low confidence)	AZOBR_100411	Horizontally Transferred (high confidence)
AZOLI_1999	Horizontally Transferred (high confidence)	AZOBR_100412	Horizontally Transferred (high confidence)
AZOLI_2000	Horizontally Transferred (high confidence)	AZOBR_110001	Horizontally Transferred (high confidence)
AZOLI_2001	Horizontally Transferred (low confidence)	AZOBR_110002	Unassigned
AZOLI_2002	Unassigned	AZOBR_110003	Ancestral (medium confidence)
AZOLI_2004	Horizontally Transferred (high confidence)	AZOBR_110004	Horizontally Transferred (high confidence)
AZOLI_2005	Unassigned	AZOBR_110005	Horizontally Transferred (medium confidence)
AZOLI_2006	Ancestral (low confidence)	AZOBR_110006	Horizontally Transferred (high confidence)
AZOLI_2007	Ancestral (low confidence)	AZOBR_110007	Horizontally Transferred (high confidence)
AZOLI_2008	Ancestral (low confidence)	AZOBR_110008	Horizontally Transferred (high confidence)
AZOLI_2010	Ancestral (low confidence)	AZOBR_110009	Unassigned
AZOLI_2011	Horizontally Transferred (high confidence)	AZOBR_110010	Unassigned
AZOLI_2012	Unassigned	AZOBR_110011	Horizontally Transferred (high confidence)
AZOLI_2013	Horizontally Transferred (high confidence)	AZOBR_110012	Ancestral (high confidence)
AZOLI_2015	Unassigned	AZOBR_110013	Ancestral (medium confidence)
AZOLI_2016	Horizontally Transferred (high confidence)	AZOBR_110014	Horizontally Transferred (high confidence)
AZOLI_2017	Horizontally Transferred (high confidence)	AZOBR_110015	Ancestral (low confidence)
AZOLI_2018	Horizontally Transferred (high confidence)	AZOBR_110016	Horizontally Transferred (medium confidence)
AZOLI_2019	Horizontally Transferred (high confidence)	AZOBR_110017	Unassigned
AZOLI_2020	Ancestral (low confidence)	AZOBR_110018	Ancestral (low confidence)
AZOLI_2021	Ancestral (low confidence)	AZOBR_110019	Ancestral (low confidence)
AZOLI_2022	Ancestral (medium confidence)	AZOBR_110020	Ancestral (medium confidence)
AZOLI_2023	Ancestral (low confidence)	AZOBR_110021	Horizontally Transferred (high confidence)
AZOLI_2024	Unassigned	AZOBR_110022	Horizontally Transferred (high confidence)
AZOLI_2025	Ancestral (low confidence)	AZOBR_110023	Horizontally Transferred (medium confidence)
AZOLI_2026	Horizontally Transferred (high confidence)	AZOBR_110024	Horizontally Transferred (high confidence)
AZOLI_2027	Horizontally Transferred (high confidence)	AZOBR_110025	Ancestral (low confidence)
AZOLI_2028	Unassigned	AZOBR_110026	Ancestral (low confidence)
AZOLI_2029	Horizontally Transferred (medium confidence)	AZOBR_110027	Ancestral (medium confidence)
AZOLI_2030	Unassigned	AZOBR_110028	Horizontally Transferred (high confidence)
AZOLI_2031	Horizontally Transferred (medium confidence)	AZOBR_110029	Horizontally Transferred (high confidence)
AZOLI_2032	Unassigned	AZOBR_110030	Ancestral (medium confidence)
AZOLI_2033	Unassigned	AZOBR_110031	Horizontally Transferred (low confidence)
AZOLI_2034	Horizontally Transferred (high confidence)	AZOBR_110032	Unassigned
AZOLI_2035	Horizontally Transferred (high confidence)	AZOBR_110033	Ancestral (low confidence)
AZOLI_2036	Unassigned	AZOBR_110034	Ancestral (low confidence)
AZOLI_2037	Horizontally Transferred (medium confidence)	AZOBR_110035	Unassigned
AZOLI_2038	Horizontally Transferred (high confidence)	AZOBR_110036	Ancestral (low confidence)
AZOLI_2039	Horizontally Transferred (high confidence)	AZOBR_110037	Horizontally Transferred (low confidence)
AZOLI_2040	Horizontally Transferred (low confidence)	AZOBR_110038	Horizontally Transferred (high confidence)
AZOLI_2041	Unassigned	AZOBR_110039	Horizontally Transferred (high confidence)
AZOLI_2043	Horizontally Transferred (high confidence)	AZOBR_110040	Unassigned
AZOLI_2045	Unassigned	AZOBR_110041	Horizontally Transferred (high confidence)
AZOLI_2046	Unassigned	AZOBR_110042	Unassigned
AZOLI_2047	Unassigned	AZOBR_110043	Ancestral (medium confidence)
AZOLI_2048	Unassigned	AZOBR_110044	Horizontally Transferred (high confidence)
AZOLI_2049	Horizontally Transferred (high confidence)	AZOBR_110045	Ancestral (low confidence)
AZOLI_2050	Horizontally Transferred (high confidence)	AZOBR_110046	Unassigned
AZOLI_2051	Horizontally Transferred (high confidence)	AZOBR_110047	Unassigned
AZOLI_2052	Ancestral (low confidence)	AZOBR_110048	Ancestral (medium confidence)
AZOLI_2053	Ancestral (low confidence)	AZOBR_110049	Ancestral (low confidence)
AZOLI_2054	Unassigned	AZOBR_110050	Ancestral (medium confidence)
AZOLI_2055	Horizontally Transferred (high confidence)	AZOBR_110051	Ancestral (low confidence)
AZOLI_2056	Ancestral (medium confidence)	AZOBR_110052	Ancestral (high confidence)
AZOLI_2057	Horizontally Transferred (high confidence)	AZOBR_110053	Ancestral (low confidence)
AZOLI_2058	Horizontally Transferred (high confidence)	AZOBR_110054	Ancestral (low confidence)
AZOLI_2059	Horizontally Transferred (high confidence)	AZOBR_110055	Unassigned

AZOLI_2060	Ancestral (medium confidence)	AZOBR_110056	Horizontally Transferred (high confidence)
AZOLI_2061	Horizontally Transferred (low confidence)	AZOBR_110057	Ancestral (low confidence)
AZOLI_2062	Horizontally Transferred (high confidence)	AZOBR_110058	Unassigned
AZOLI_2063	Horizontally Transferred (high confidence)	AZOBR_110059	Ancestral (low confidence)
AZOLI_2064	Horizontally Transferred (high confidence)	AZOBR_110060	Ancestral (medium confidence)
AZOLI_2065	Horizontally Transferred (high confidence)	AZOBR_110061	Ancestral (high confidence)
AZOLI_2066	Horizontally Transferred (high confidence)	AZOBR_110062	Horizontally Transferred (high confidence)
AZOLI_2067	Unassigned	AZOBR_110063	Horizontally Transferred (high confidence)
AZOLI_2068	Horizontally Transferred (high confidence)	AZOBR_110064	Horizontally Transferred (high confidence)
AZOLI_2070	Horizontally Transferred (high confidence)	AZOBR_110065	Ancestral (high confidence)
AZOLI_2071	Horizontally Transferred (medium confidence)	AZOBR_110066	Ancestral (low confidence)
AZOLI_2072	Horizontally Transferred (medium confidence)	AZOBR_110067	Horizontally Transferred (medium confidence)
AZOLI_2073	Unassigned	AZOBR_110068	Ancestral (low confidence)
AZOLI_2076	Unassigned	AZOBR_110069	Horizontally Transferred (high confidence)
AZOLI_2077	Ancestral (low confidence)	AZOBR_110070	Ancestral (medium confidence)
AZOLI_2078	Ancestral (low confidence)	AZOBR_110071	Ancestral (medium confidence)
AZOLI_2079	Horizontally Transferred (high confidence)	AZOBR_110072	Unassigned
AZOLI_2080	Unassigned	AZOBR_110073	Ancestral (medium confidence)
AZOLI_2081	Unassigned	AZOBR_110074	Ancestral (high confidence)
AZOLI_2082	Unassigned	AZOBR_110075	Ancestral (high confidence)
AZOLI_2083	Unassigned	AZOBR_110076	Ancestral (high confidence)
AZOLI_2084	Unassigned	AZOBR_110077	Ancestral (medium confidence)
AZOLI_2085	Horizontally Transferred (high confidence)	AZOBR_110078	Ancestral (high confidence)
AZOLI_2086	Horizontally Transferred (high confidence)	AZOBR_110079	Ancestral (high confidence)
AZOLI_2088	Ancestral (low confidence)	AZOBR_110080	Ancestral (medium confidence)
AZOLI_2089	Horizontally Transferred (high confidence)	AZOBR_110081	Unassigned
AZOLI_2090	Ancestral (low confidence)	AZOBR_110082	Ancestral (medium confidence)
AZOLI_2091	Unassigned	AZOBR_110083	Ancestral (medium confidence)
AZOLI_2095	Horizontally Transferred (high confidence)	AZOBR_110084	Ancestral (high confidence)
AZOLI_2096	Unassigned	AZOBR_110085	Ancestral (low confidence)
AZOLI_2097	Horizontally Transferred (high confidence)	AZOBR_110086	Ancestral (low confidence)
AZOLI_2098	Horizontally Transferred (low confidence)	AZOBR_110087	Ancestral (low confidence)
AZOLI_2099	Horizontally Transferred (high confidence)	AZOBR_110088	Ancestral (medium confidence)
AZOLI_2100	Horizontally Transferred (high confidence)	AZOBR_110089	Ancestral (high confidence)
AZOLI_2101	Horizontally Transferred (high confidence)	AZOBR_110090	Ancestral (high confidence)
AZOLI_2102	Unassigned	AZOBR_110091	Ancestral (high confidence)
AZOLI_2103	Horizontally Transferred (high confidence)	AZOBR_110092	Ancestral (high confidence)
AZOLI_2104	Ancestral (low confidence)	AZOBR_110093	Ancestral (medium confidence)
AZOLI_2105	Ancestral (low confidence)	AZOBR_110094	Horizontally Transferred (high confidence)
AZOLI_2106	Ancestral (low confidence)	AZOBR_110095	Unassigned
AZOLI_2107	Unassigned	AZOBR_110096	Ancestral (high confidence)
AZOLI_2108	Horizontally Transferred (high confidence)	AZOBR_110097	Ancestral (high confidence)
AZOLI_2109	Horizontally Transferred (medium confidence)	AZOBR_110098	Ancestral (high confidence)
AZOLI_2110	Ancestral (low confidence)	AZOBR_110099	Ancestral (high confidence)
AZOLI_2111	Ancestral (medium confidence)	AZOBR_110100	Unassigned
AZOLI_2112	Unassigned	AZOBR_110101	Ancestral (low confidence)
AZOLI_2113	Ancestral (medium confidence)	AZOBR_110102	Ancestral (high confidence)
AZOLI_2114	Horizontally Transferred (high confidence)	AZOBR_110103	Ancestral (high confidence)
AZOLI_2115	Ancestral (high confidence)	AZOBR_110104	Ancestral (low confidence)
AZOLI_2116	Ancestral (high confidence)	AZOBR_110105	Ancestral (medium confidence)
AZOLI_2117	Ancestral (high confidence)	AZOBR_110106	Ancestral (high confidence)
AZOLI_2118	Ancestral (low confidence)	AZOBR_110107	Ancestral (medium confidence)
AZOLI_2120	Unassigned	AZOBR_110108	Ancestral (high confidence)
AZOLI_2121	Ancestral (medium confidence)	AZOBR_110109	Ancestral (high confidence)
AZOLI_2122	Ancestral (high confidence)	AZOBR_110110	Ancestral (high confidence)
AZOLI_2123	Ancestral (high confidence)	AZOBR_110111	Ancestral (medium confidence)
AZOLI_2124	Ancestral (high confidence)	AZOBR_110112	Unassigned
AZOLI_2125	Ancestral (high confidence)	AZOBR_110113	Ancestral (high confidence)
AZOLI_2126	Ancestral (medium confidence)	AZOBR_110114	Ancestral (high confidence)
AZOLI_2127	Ancestral (high confidence)	AZOBR_110115	Ancestral (high confidence)
AZOLI_2128	Ancestral (high confidence)	AZOBR_110116	Horizontally Transferred (high confidence)
AZOLI_2129	Ancestral (high confidence)	AZOBR_110117	Horizontally Transferred (high confidence)
AZOLI_2130	Ancestral (high confidence)	AZOBR_110118	Horizontally Transferred (high confidence)
AZOLI_2131	Horizontally Transferred (high confidence)	AZOBR_110119	Horizontally Transferred (high confidence)
AZOLI_2132	Ancestral (high confidence)	AZOBR_110120	Ancestral (high confidence)
AZOLI_2133	Ancestral (medium confidence)	AZOBR_110121	Ancestral (medium confidence)
AZOLI_2134	Ancestral (medium confidence)	AZOBR_110122	Ancestral (medium confidence)
AZOLI_2135	Ancestral (medium confidence)	AZOBR_110123	Ancestral (high confidence)
AZOLI_2136	Ancestral (high confidence)	AZOBR_110124	Unassigned
AZOLI_2137	Ancestral (medium confidence)	AZOBR_110125	Ancestral (medium confidence)
AZOLI_2140	Ancestral (high confidence)	AZOBR_110126	Horizontally Transferred (high confidence)
AZOLI_2141	Unassigned	AZOBR_110127	Ancestral (medium confidence)
AZOLI_2142	Ancestral (medium confidence)	AZOBR_110128	Ancestral (medium confidence)
AZOLI_2143	Ancestral (medium confidence)	AZOBR_110129	Unassigned
AZOLI_2144	Horizontally Transferred (high confidence)	AZOBR_110130	Unassigned

AZOLI_2145	Ancestral (high confidence)	AZOBR_120001	Ancestral (medium confidence)
AZOLI_2146	Ancestral (high confidence)	AZOBR_120002	Ancestral (medium confidence)
AZOLI_2147	Ancestral (medium confidence)	AZOBR_120003	Horizontally Transferred (high confidence)
AZOLI_2149	Ancestral (medium confidence)	AZOBR_120004	Horizontally Transferred (high confidence)
AZOLI_2150	Ancestral (high confidence)	AZOBR_120005	Horizontally Transferred (high confidence)
AZOLI_2151	Ancestral (high confidence)	AZOBR_120006	Unassigned
AZOLI_2152	Horizontally Transferred (low confidence)	AZOBR_120007	Ancestral (low confidence)
AZOLI_2153	Ancestral (medium confidence)	AZOBR_120008	Unassigned
AZOLI_2154	Horizontally Transferred (high confidence)	AZOBR_120009	Ancestral (medium confidence)
AZOLI_2155	Horizontally Transferred (high confidence)	AZOBR_120010	Ancestral (high confidence)
AZOLI_2156	Ancestral (high confidence)	AZOBR_120011	Ancestral (high confidence)
AZOLI_2157	Horizontally Transferred (high confidence)	AZOBR_120012	Horizontally Transferred (high confidence)
AZOLI_2158	Horizontally Transferred (high confidence)	AZOBR_120013	Ancestral (medium confidence)
AZOLI_2159	Ancestral (low confidence)	AZOBR_120014	Ancestral (low confidence)
AZOLI_2160	Ancestral (medium confidence)	AZOBR_120015	Ancestral (medium confidence)
AZOLI_2161	Ancestral (high confidence)	AZOBR_120016	Ancestral (medium confidence)
AZOLI_2162	Ancestral (high confidence)	AZOBR_120017	Ancestral (medium confidence)
AZOLI_2163	Ancestral (medium confidence)	AZOBR_120018	Ancestral (low confidence)
AZOLI_2164	Ancestral (low confidence)	AZOBR_120019	Ancestral (medium confidence)
AZOLI_2165	Ancestral (low confidence)	AZOBR_120020	Ancestral (medium confidence)
AZOLI_2166	Ancestral (low confidence)	AZOBR_120021	Ancestral (medium confidence)
AZOLI_2167	Ancestral (low confidence)	AZOBR_120022	Ancestral (medium confidence)
AZOLI_2168	Ancestral (low confidence)	AZOBR_120023	Ancestral (medium confidence)
AZOLI_2169	Ancestral (high confidence)	AZOBR_120024	Ancestral (medium confidence)
AZOLI_2170	Ancestral (low confidence)	AZOBR_120025	Horizontally Transferred (high confidence)
AZOLI_2171	Ancestral (high confidence)	AZOBR_120026	Ancestral (medium confidence)
AZOLI_2172	Ancestral (high confidence)	AZOBR_120027	Horizontally Transferred (medium confidence)
AZOLI_2173	Ancestral (high confidence)	AZOBR_120028	Unassigned
AZOLI_2174	Ancestral (medium confidence)	AZOBR_120029	Ancestral (medium confidence)
AZOLI_2175	Horizontally Transferred (high confidence)	AZOBR_120030	Ancestral (low confidence)
AZOLI_2176	Horizontally Transferred (high confidence)	AZOBR_120031	Ancestral (low confidence)
AZOLI_2177	Ancestral (medium confidence)	AZOBR_120032	Ancestral (medium confidence)
AZOLI_2178	Ancestral (high confidence)	AZOBR_120033	Ancestral (medium confidence)
AZOLI_2179	Unassigned	AZOBR_120034	Ancestral (medium confidence)
AZOLI_2180	Ancestral (high confidence)	AZOBR_120035	Horizontally Transferred (high confidence)
AZOLI_2182	Ancestral (medium confidence)	AZOBR_120036	Ancestral (high confidence)
AZOLI_2183	Horizontally Transferred (high confidence)	AZOBR_120037	Ancestral (medium confidence)
AZOLI_2184	Horizontally Transferred (high confidence)	AZOBR_120038	Ancestral (high confidence)
AZOLI_2186	Ancestral (medium confidence)	AZOBR_120039	Ancestral (high confidence)
AZOLI_2187	Ancestral (medium confidence)	AZOBR_120040	Unassigned
AZOLI_2188	Ancestral (high confidence)	AZOBR_120041	Unassigned
AZOLI_2190	Ancestral (high confidence)	AZOBR_120042	Unassigned
AZOLI_2191	Ancestral (high confidence)	AZOBR_120043	Horizontally Transferred (high confidence)
AZOLI_2192	Ancestral (high confidence)	AZOBR_120044	Ancestral (medium confidence)
AZOLI_2193	Ancestral (high confidence)	AZOBR_120045	Horizontally Transferred (medium confidence)
AZOLI_2194	Ancestral (high confidence)	AZOBR_120046	Ancestral (medium confidence)
AZOLI_2195	Ancestral (high confidence)	AZOBR_120047	Ancestral (low confidence)
AZOLI_2196	Ancestral (medium confidence)	AZOBR_120048	Ancestral (high confidence)
AZOLI_2197	Ancestral (medium confidence)	AZOBR_120049	Ancestral (medium confidence)
AZOLI_2198	Ancestral (high confidence)	AZOBR_120050	Ancestral (medium confidence)
AZOLI_2199	Ancestral (high confidence)	AZOBR_120051	Ancestral (high confidence)
AZOLI_2200	Ancestral (high confidence)	AZOBR_120052	Ancestral (low confidence)
AZOLI_2201	Ancestral (medium confidence)	AZOBR_120053	Ancestral (medium confidence)
AZOLI_2202	Ancestral (high confidence)	AZOBR_120054	Ancestral (medium confidence)
AZOLI_2203	Ancestral (high confidence)	AZOBR_120055	Unassigned
AZOLI_2204	Ancestral (high confidence)	AZOBR_120056	Unassigned
AZOLI_2205	Ancestral (high confidence)	AZOBR_120057	Ancestral (high confidence)
AZOLI_2206	Ancestral (high confidence)	AZOBR_120058	Ancestral (low confidence)
AZOLI_2207	Unassigned	AZOBR_120059	Ancestral (high confidence)
AZOLI_2208	Ancestral (medium confidence)	AZOBR_120060	Ancestral (high confidence)
AZOLI_2209	Unassigned	AZOBR_120061	Ancestral (low confidence)
AZOLI_2210	Ancestral (medium confidence)	AZOBR_120062	Unassigned
AZOLI_2211	Ancestral (high confidence)	AZOBR_120063	Ancestral (high confidence)
AZOLI_2212	Ancestral (medium confidence)	AZOBR_120064	Ancestral (medium confidence)
AZOLI_2213	Ancestral (high confidence)	AZOBR_120065	Ancestral (medium confidence)
AZOLI_2214	Ancestral (high confidence)	AZOBR_120066	Ancestral (high confidence)
AZOLI_2215	Horizontally Transferred (low confidence)	AZOBR_130001	Ancestral (high confidence)
AZOLI_2216	Horizontally Transferred (high confidence)	AZOBR_130002	Ancestral (high confidence)
AZOLI_2217	Ancestral (low confidence)	AZOBR_130003	Ancestral (high confidence)
AZOLI_2218	Horizontally Transferred (medium confidence)	AZOBR_130004	Ancestral (high confidence)
AZOLI_2219	Ancestral (high confidence)	AZOBR_130005	Ancestral (high confidence)
AZOLI_2220	Ancestral (medium confidence)	AZOBR_130006	Ancestral (high confidence)
AZOLI_2221	Ancestral (medium confidence)	AZOBR_130007	Ancestral (high confidence)
AZOLI_2222	Unassigned	AZOBR_130008	Horizontally Transferred (high confidence)
AZOLI_2224	Ancestral (high confidence)	AZOBR_130009	Horizontally Transferred (high confidence)

AZOLI_2226	Horizontally Transferred (high confidence)	AZOBR_130010	Horizontally Transferred (high confidence)
AZOLI_2228	Ancestral (high confidence)	AZOBR_130011	Horizontally Transferred (high confidence)
AZOLI_2229	Ancestral (high confidence)	AZOBR_130012	Ancestral (low confidence)
AZOLI_2230	Ancestral (high confidence)	AZOBR_130013	Unassigned
AZOLI_2231	Ancestral (high confidence)	AZOBR_130014	Ancestral (high confidence)
AZOLI_2232	Ancestral (high confidence)	AZOBR_130015	Ancestral (medium confidence)
AZOLI_2234	Horizontally Transferred (high confidence)	AZOBR_130016	Ancestral (medium confidence)
AZOLI_2235	Horizontally Transferred (high confidence)	AZOBR_130017	Ancestral (medium confidence)
AZOLI_2236	Ancestral (high confidence)	AZOBR_130018	Ancestral (medium confidence)
AZOLI_2237	Ancestral (medium confidence)	AZOBR_130019	Ancestral (low confidence)
AZOLI_2238	Horizontally Transferred (high confidence)	AZOBR_130020	Ancestral (medium confidence)
AZOLI_2239	Ancestral (high confidence)	AZOBR_130021	Ancestral (low confidence)
AZOLI_2240	Ancestral (high confidence)	AZOBR_130022	Horizontally Transferred (high confidence)
AZOLI_2241	Ancestral (high confidence)	AZOBR_130023	Horizontally Transferred (high confidence)
AZOLI_2242	Horizontally Transferred (medium confidence)	AZOBR_130024	Ancestral (low confidence)
AZOLI_2244	Horizontally Transferred (high confidence)	AZOBR_130025	Ancestral (low confidence)
AZOLI_2245	Horizontally Transferred (high confidence)	AZOBR_130026	Ancestral (high confidence)
AZOLI_2246	Ancestral (low confidence)	AZOBR_130027	Ancestral (high confidence)
AZOLI_2247	Ancestral (low confidence)	AZOBR_130028	Ancestral (low confidence)
AZOLI_2248	Ancestral (low confidence)	AZOBR_130029	Ancestral (medium confidence)
AZOLI_2249	Ancestral (low confidence)	AZOBR_130030	Ancestral (high confidence)
AZOLI_2250	Ancestral (low confidence)	AZOBR_130031	Horizontally Transferred (high confidence)
AZOLI_2251	Horizontally Transferred (medium confidence)	AZOBR_130032	Ancestral (low confidence)
AZOLI_2252	Horizontally Transferred (low confidence)	AZOBR_130033	Horizontally Transferred (high confidence)
AZOLI_2253	Ancestral (medium confidence)	AZOBR_130034	Ancestral (medium confidence)
AZOLI_2254	Ancestral (high confidence)	AZOBR_130035	Horizontally Transferred (high confidence)
AZOLI_2255	Unassigned	AZOBR_130036	Horizontally Transferred (high confidence)
AZOLI_2256	Ancestral (low confidence)	AZOBR_130037	Ancestral (high confidence)
AZOLI_2257	Ancestral (low confidence)	AZOBR_130038	Ancestral (high confidence)
AZOLI_2258	Horizontally Transferred (high confidence)	AZOBR_130039	Ancestral (medium confidence)
AZOLI_2259	Ancestral (medium confidence)	AZOBR_130040	Ancestral (low confidence)
AZOLI_2260	Ancestral (high confidence)	AZOBR_130041	Ancestral (high confidence)
AZOLI_2261	Ancestral (medium confidence)	AZOBR_130042	Ancestral (high confidence)
AZOLI_2262	Unassigned	AZOBR_130043	Unassigned
AZOLI_2263	Unassigned	AZOBR_130044	Ancestral (high confidence)
AZOLI_2264	Ancestral (medium confidence)	AZOBR_130045	Unassigned
AZOLI_2265	Ancestral (high confidence)	AZOBR_130046	Ancestral (low confidence)
AZOLI_2266	Ancestral (high confidence)	AZOBR_130050	Unassigned
AZOLI_2267	Horizontally Transferred (high confidence)	AZOBR_130051	Horizontally Transferred (high confidence)
AZOLI_2268	Ancestral (low confidence)	AZOBR_140001	Ancestral (low confidence)
AZOLI_2269	Unassigned	AZOBR_140002	Ancestral (medium confidence)
AZOLI_2270	Ancestral (high confidence)	AZOBR_140003	Unassigned
AZOLI_2271	Ancestral (high confidence)	AZOBR_140004	Horizontally Transferred (high confidence)
AZOLI_2272	Horizontally Transferred (high confidence)	AZOBR_140005	Ancestral (medium confidence)
AZOLI_2273	Ancestral (high confidence)	AZOBR_140006	Ancestral (low confidence)
AZOLI_2274	Ancestral (high confidence)	AZOBR_140007	Ancestral (high confidence)
AZOLI_2275	Ancestral (high confidence)	AZOBR_140008	Unassigned
AZOLI_2276	Ancestral (low confidence)	AZOBR_140009	Ancestral (high confidence)
AZOLI_2277	Ancestral (medium confidence)	AZOBR_140010	Ancestral (medium confidence)
AZOLI_2278	Unassigned	AZOBR_140011	Ancestral (high confidence)
AZOLI_2280	Ancestral (medium confidence)	AZOBR_140012	Ancestral (high confidence)
AZOLI_2281	Ancestral (medium confidence)	AZOBR_140013	Ancestral (high confidence)
AZOLI_2282	Ancestral (medium confidence)	AZOBR_140014	Ancestral (high confidence)
AZOLI_2283	Unassigned	AZOBR_140015	Ancestral (medium confidence)
AZOLI_2284	Ancestral (medium confidence)	AZOBR_140016	Ancestral (low confidence)
AZOLI_2285	Ancestral (high confidence)	AZOBR_140017	Ancestral (high confidence)
AZOLI_2286	Ancestral (high confidence)	AZOBR_140018	Ancestral (high confidence)
AZOLI_2287	Ancestral (high confidence)	AZOBR_140019	Unassigned
AZOLI_2288	Ancestral (high confidence)	AZOBR_140020	Ancestral (high confidence)
AZOLI_2289	Unassigned	AZOBR_140021	Unassigned
AZOLI_2290	Ancestral (high confidence)	AZOBR_140022	Ancestral (high confidence)
AZOLI_2291	Ancestral (high confidence)	AZOBR_140023	Ancestral (low confidence)
AZOLI_2292	Ancestral (low confidence)	AZOBR_140024	Ancestral (high confidence)
AZOLI_2293	Unassigned	AZOBR_140025	Ancestral (medium confidence)
AZOLI_2294	Unassigned	AZOBR_140026	Unassigned
AZOLI_2295	Unassigned	AZOBR_140027	Horizontally Transferred (high confidence)
AZOLI_2296	Horizontally Transferred (high confidence)	AZOBR_140028	Unassigned
AZOLI_2297	Horizontally Transferred (high confidence)	AZOBR_140029	Horizontally Transferred (high confidence)
AZOLI_2298	Horizontally Transferred (high confidence)	AZOBR_140030	Ancestral (low confidence)
AZOLI_2300	Horizontally Transferred (high confidence)	AZOBR_140031	Ancestral (high confidence)
AZOLI_2302	Ancestral (high confidence)	AZOBR_140032	Ancestral (medium confidence)
AZOLI_2303	Ancestral (medium confidence)	AZOBR_140033	Horizontally Transferred (high confidence)
AZOLI_2304	Ancestral (high confidence)	AZOBR_140034	Horizontally Transferred (high confidence)
AZOLI_2305	Ancestral (low confidence)	AZOBR_140035	Horizontally Transferred (high confidence)
AZOLI_2308	Ancestral (medium confidence)	AZOBR_140036	Unassigned

AZOLI_2309	Horizontally Transferred (high confidence)	AZOBR_140037	Ancestral (medium confidence)
AZOLI_2311	Horizontally Transferred (high confidence)	AZOBR_140038	Ancestral (medium confidence)
AZOLI_2312	Horizontally Transferred (medium confidence)	AZOBR_140039	Horizontally Transferred (high confidence)
AZOLI_2313	Unassigned	AZOBR_140040	Horizontally Transferred (high confidence)
AZOLI_2314	Unassigned	AZOBR_140041	Unassigned
AZOLI_2315	Ancestral (medium confidence)	AZOBR_140042	Ancestral (medium confidence)
AZOLI_2316	Horizontally Transferred (high confidence)	AZOBR_140043	Ancestral (medium confidence)
AZOLI_2317	Horizontally Transferred (high confidence)	AZOBR_140044	Ancestral (high confidence)
AZOLI_2318	Ancestral (low confidence)	AZOBR_140045	Ancestral (medium confidence)
AZOLI_2319	Horizontally Transferred (high confidence)	AZOBR_140046	Unassigned
AZOLI_2320	Horizontally Transferred (high confidence)	AZOBR_140047	Ancestral (medium confidence)
AZOLI_2321	Unassigned	AZOBR_140048	Ancestral (low confidence)
AZOLI_2322	Ancestral (low confidence)	AZOBR_140049	Ancestral (low confidence)
AZOLI_2323	Ancestral (medium confidence)	AZOBR_140050	Ancestral (medium confidence)
AZOLI_2324	Horizontally Transferred (high confidence)	AZOBR_140051	Ancestral (medium confidence)
AZOLI_2325	Horizontally Transferred (high confidence)	AZOBR_140052	Ancestral (medium confidence)
AZOLI_2326	Ancestral (medium confidence)	AZOBR_140053	Ancestral (high confidence)
AZOLI_2327	Ancestral (medium confidence)	AZOBR_140054	Ancestral (high confidence)
AZOLI_2328	Horizontally Transferred (high confidence)	AZOBR_140055	Ancestral (high confidence)
AZOLI_2329	Ancestral (low confidence)	AZOBR_140056	Unassigned
AZOLI_2331	Ancestral (medium confidence)	AZOBR_140057	Ancestral (high confidence)
AZOLI_2332	Horizontally Transferred (high confidence)	AZOBR_140058	Ancestral (high confidence)
AZOLI_2333	Horizontally Transferred (high confidence)	AZOBR_140059	Horizontally Transferred (high confidence)
AZOLI_2334	Horizontally Transferred (high confidence)	AZOBR_140060	Horizontally Transferred (high confidence)
AZOLI_2335	Horizontally Transferred (high confidence)	AZOBR_140061	Ancestral (high confidence)
AZOLI_2336	Ancestral (low confidence)	AZOBR_140062	Ancestral (low confidence)
AZOLI_2337	Ancestral (low confidence)	AZOBR_140063	Ancestral (low confidence)
AZOLI_2338	Horizontally Transferred (high confidence)	AZOBR_140064	Unassigned
AZOLI_2339	Unassigned	AZOBR_140065	Ancestral (medium confidence)
AZOLI_2340	Horizontally Transferred (high confidence)	AZOBR_140066	Horizontally Transferred (high confidence)
AZOLI_2341	Horizontally Transferred (high confidence)	AZOBR_140067	Ancestral (medium confidence)
AZOLI_2342	Ancestral (medium confidence)	AZOBR_140068	Ancestral (medium confidence)
AZOLI_2343	Ancestral (high confidence)	AZOBR_140069	Unassigned
AZOLI_2344	Ancestral (high confidence)	AZOBR_140070	Unassigned
AZOLI_2345	Ancestral (high confidence)	AZOBR_140071	Unassigned
AZOLI_2347	Ancestral (medium confidence)	AZOBR_140072	Ancestral (low confidence)
AZOLI_2348	Ancestral (high confidence)	AZOBR_140073	Ancestral (medium confidence)
AZOLI_2349	Ancestral (high confidence)	AZOBR_140074	Ancestral (medium confidence)
AZOLI_2350	Horizontally Transferred (medium confidence)	AZOBR_140075	Horizontally Transferred (high confidence)
AZOLI_2351	Horizontally Transferred (high confidence)	AZOBR_140076	Unassigned
AZOLI_2352	Horizontally Transferred (medium confidence)	AZOBR_140077	Unassigned
AZOLI_2353	Ancestral (medium confidence)	AZOBR_140078	Ancestral (medium confidence)
AZOLI_2354	Ancestral (medium confidence)	AZOBR_140079	Ancestral (high confidence)
AZOLI_2355	Ancestral (low confidence)	AZOBR_140080	Ancestral (high confidence)
AZOLI_2356	Unassigned	AZOBR_140081	Ancestral (low confidence)
AZOLI_2357	Ancestral (high confidence)	AZOBR_140082	Unassigned
AZOLI_2358	Ancestral (high confidence)	AZOBR_140083	Horizontally Transferred (high confidence)
AZOLI_2359	Unassigned	AZOBR_140084	Horizontally Transferred (high confidence)
AZOLI_2360	Horizontally Transferred (high confidence)	AZOBR_140085	Ancestral (low confidence)
AZOLI_2361	Ancestral (high confidence)	AZOBR_140086	Unassigned
AZOLI_2362	Ancestral (medium confidence)	AZOBR_140087	Horizontally Transferred (high confidence)
AZOLI_2363	Horizontally Transferred (high confidence)	AZOBR_140088	Unassigned
AZOLI_2364	Ancestral (high confidence)	AZOBR_140089	Horizontally Transferred (high confidence)
AZOLI_2366	Horizontally Transferred (high confidence)	AZOBR_140090	Unassigned
AZOLI_2367	Ancestral (medium confidence)	AZOBR_140091	Ancestral (low confidence)
AZOLI_2368	Horizontally Transferred (high confidence)	AZOBR_140092	Ancestral (low confidence)
AZOLI_2369	Horizontally Transferred (high confidence)	AZOBR_140093	Ancestral (low confidence)
AZOLI_2370	Ancestral (medium confidence)	AZOBR_140094	Ancestral (low confidence)
AZOLI_2371	Ancestral (medium confidence)	AZOBR_140095	Ancestral (medium confidence)
AZOLI_2372	Ancestral (medium confidence)	AZOBR_140096	Ancestral (low confidence)
AZOLI_2373	Ancestral (medium confidence)	AZOBR_140097	Horizontally Transferred (high confidence)
AZOLI_2375	Ancestral (low confidence)	AZOBR_140098	Ancestral (low confidence)
AZOLI_2376	Horizontally Transferred (high confidence)	AZOBR_140099	Unassigned
AZOLI_2377	Unassigned	AZOBR_140100	Ancestral (medium confidence)
AZOLI_2380	Ancestral (medium confidence)	AZOBR_140101	Ancestral (medium confidence)
AZOLI_2381	Ancestral (medium confidence)	AZOBR_140102	Ancestral (high confidence)
AZOLI_2382	Ancestral (medium confidence)	AZOBR_140103	Ancestral (high confidence)
AZOLI_2383	Ancestral (medium confidence)	AZOBR_140104	Ancestral (low confidence)
AZOLI_2384	Ancestral (high confidence)	AZOBR_140105	Ancestral (low confidence)
AZOLI_2385	Ancestral (high confidence)	AZOBR_140106	Ancestral (medium confidence)
AZOLI_2386	Unassigned	AZOBR_140107	Ancestral (medium confidence)
AZOLI_2387	Ancestral (high confidence)	AZOBR_140108	Ancestral (low confidence)
AZOLI_2388	Ancestral (medium confidence)	AZOBR_140109	Ancestral (low confidence)
AZOLI_2389	Ancestral (high confidence)	AZOBR_140110	Ancestral (low confidence)
AZOLI_2391	Ancestral (high confidence)	AZOBR_140111	Ancestral (high confidence)

AZOLI_2392	Horizontally Transferred (high confidence)	AZOBR_140112	Ancestral (medium confidence)
AZOLI_2393	Unassigned	AZOBR_140113	Ancestral (low confidence)
AZOLI_2394	Horizontally Transferred (high confidence)	AZOBR_140114	Horizontally Transferred (high confidence)
AZOLI_2395	Ancestral (medium confidence)	AZOBR_140115	Unassigned
AZOLI_2396	Horizontally Transferred (high confidence)	AZOBR_140116	Ancestral (medium confidence)
AZOLI_2397	Unassigned	AZOBR_140117	Unassigned
AZOLI_2398	Ancestral (medium confidence)	AZOBR_140118	Horizontally Transferred (high confidence)
AZOLI_2399	Ancestral (low confidence)	AZOBR_140119	Ancestral (low confidence)
AZOLI_2400	Horizontally Transferred (high confidence)	AZOBR_140120	Unassigned
AZOLI_2401	Horizontally Transferred (high confidence)	AZOBR_140121	Ancestral (high confidence)
AZOLI_2402	Ancestral (medium confidence)	AZOBR_140122	Ancestral (medium confidence)
AZOLI_2403	Ancestral (high confidence)	AZOBR_140123	Ancestral (high confidence)
AZOLI_2404	Ancestral (medium confidence)	AZOBR_140124	Ancestral (high confidence)
AZOLI_2405	Unassigned	AZOBR_140125	Ancestral (high confidence)
AZOLI_2406	Horizontally Transferred (high confidence)	AZOBR_140126	Ancestral (high confidence)
AZOLI_2407	Unassigned	AZOBR_140127	Ancestral (low confidence)
AZOLI_2408	Ancestral (medium confidence)	AZOBR_140128	Ancestral (medium confidence)
AZOLI_2409	Ancestral (medium confidence)	AZOBR_140129	Ancestral (low confidence)
AZOLI_2410	Unassigned	AZOBR_140130	Ancestral (low confidence)
AZOLI_2411	Ancestral (high confidence)	AZOBR_140131	Ancestral (high confidence)
AZOLI_2412	Ancestral (high confidence)	AZOBR_140132	Ancestral (high confidence)
AZOLI_2413	Ancestral (low confidence)	AZOBR_140133	Ancestral (low confidence)
AZOLI_2414	Ancestral (low confidence)	AZOBR_140134	Ancestral (medium confidence)
AZOLI_2415	Ancestral (high confidence)	AZOBR_140135	Ancestral (low confidence)
AZOLI_2416	Ancestral (high confidence)	AZOBR_140136	Ancestral (high confidence)
AZOLI_2417	Ancestral (medium confidence)	AZOBR_140137	Ancestral (high confidence)
AZOLI_2418	Ancestral (high confidence)	AZOBR_140138	Ancestral (medium confidence)
AZOLI_2419	Ancestral (medium confidence)	AZOBR_140139	Ancestral (medium confidence)
AZOLI_2420	Ancestral (high confidence)	AZOBR_140140	Ancestral (high confidence)
AZOLI_2421	Ancestral (high confidence)	AZOBR_140141	Unassigned
AZOLI_2422	Ancestral (low confidence)	AZOBR_140142	Ancestral (medium confidence)
AZOLI_2423	Ancestral (high confidence)	AZOBR_140143	Ancestral (medium confidence)
AZOLI_2424	Horizontally Transferred (high confidence)	AZOBR_140144	Ancestral (medium confidence)
AZOLI_2425	Ancestral (low confidence)	AZOBR_140145	Ancestral (high confidence)
AZOLI_2426	Horizontally Transferred (high confidence)	AZOBR_140146	Horizontally Transferred (high confidence)
AZOLI_2427	Ancestral (low confidence)	AZOBR_140147	Unassigned
AZOLI_2428	Horizontally Transferred (high confidence)	AZOBR_140148	Ancestral (medium confidence)
AZOLI_2429	Horizontally Transferred (medium confidence)	AZOBR_140149	Horizontally Transferred (medium confidence)
AZOLI_2430	Horizontally Transferred (high confidence)	AZOBR_140150	Ancestral (high confidence)
AZOLI_2431	Ancestral (low confidence)	AZOBR_140151	Ancestral (low confidence)
AZOLI_2432	Ancestral (medium confidence)	AZOBR_140152	Horizontally Transferred (high confidence)
AZOLI_2433	Ancestral (medium confidence)	AZOBR_140153	Ancestral (medium confidence)
AZOLI_2434	Ancestral (medium confidence)	AZOBR_140154	Ancestral (low confidence)
AZOLI_2435	Ancestral (medium confidence)	AZOBR_140155	Horizontally Transferred (high confidence)
AZOLI_2436	Ancestral (medium confidence)	AZOBR_140156	Ancestral (medium confidence)
AZOLI_2437	Ancestral (medium confidence)	AZOBR_140157	Ancestral (medium confidence)
AZOLI_2438	Ancestral (low confidence)	AZOBR_140158	Ancestral (high confidence)
AZOLI_2439	Ancestral (high confidence)	AZOBR_140159	Ancestral (high confidence)
AZOLI_2440	Ancestral (high confidence)	AZOBR_140160	Ancestral (medium confidence)
AZOLI_2441	Ancestral (high confidence)	AZOBR_140161	Unassigned
AZOLI_2442	Horizontally Transferred (high confidence)	AZOBR_140162	Ancestral (low confidence)
AZOLI_2443	Ancestral (medium confidence)	AZOBR_140163	Unassigned
AZOLI_2444	Horizontally Transferred (high confidence)	AZOBR_140164	Unassigned
AZOLI_2445	Ancestral (low confidence)	AZOBR_140165	Unassigned
AZOLI_2446	Ancestral (high confidence)	AZOBR_140166	Ancestral (high confidence)
AZOLI_2447	Horizontally Transferred (high confidence)	AZOBR_140167	Ancestral (low confidence)
AZOLI_2448	Unassigned	AZOBR_140168	Horizontally Transferred (high confidence)
AZOLI_2449	Ancestral (high confidence)	AZOBR_140169	Ancestral (high confidence)
AZOLI_2450	Ancestral (high confidence)	AZOBR_140170	Ancestral (medium confidence)
AZOLI_2451	Ancestral (high confidence)	AZOBR_140171	Horizontally Transferred (high confidence)
AZOLI_2452	Ancestral (high confidence)	AZOBR_140172	Ancestral (high confidence)
AZOLI_2453	Ancestral (low confidence)	AZOBR_140173	Horizontally Transferred (high confidence)
AZOLI_2454	Ancestral (medium confidence)	AZOBR_140174	Ancestral (medium confidence)
AZOLI_2455	Ancestral (low confidence)	AZOBR_140175	Unassigned
AZOLI_2456	Ancestral (low confidence)	AZOBR_140176	Ancestral (low confidence)
AZOLI_2457	Unassigned	AZOBR_140177	Horizontally Transferred (high confidence)
AZOLI_2458	Ancestral (medium confidence)	AZOBR_140178	Horizontally Transferred (high confidence)
AZOLI_2459	Ancestral (high confidence)	AZOBR_140179	Horizontally Transferred (high confidence)
AZOLI_2460	Horizontally Transferred (high confidence)	AZOBR_140180	Ancestral (high confidence)
AZOLI_2461	Horizontally Transferred (high confidence)	AZOBR_140181	Horizontally Transferred (high confidence)
AZOLI_2462	Horizontally Transferred (high confidence)	AZOBR_140182	Horizontally Transferred (high confidence)
AZOLI_2463	Ancestral (low confidence)	AZOBR_140183	Ancestral (medium confidence)
AZOLI_2464	Ancestral (medium confidence)	AZOBR_140184	Ancestral (medium confidence)
AZOLI_2465	Ancestral (high confidence)	AZOBR_140185	Ancestral (high confidence)
AZOLI_2466	Horizontally Transferred (high confidence)	AZOBR_140186	Ancestral (medium confidence)

AZOLI_2467	Unassigned	AZOBR_140187	Ancestral (high confidence)
AZOLI_2468	Ancestral (high confidence)	AZOBR_140188	Ancestral (medium confidence)
AZOLI_2469	Ancestral (high confidence)	AZOBR_140189	Ancestral (low confidence)
AZOLI_2470	Ancestral (high confidence)	AZOBR_140190	Horizontally Transferred (medium confidence)
AZOLI_2471	Ancestral (high confidence)	AZOBR_140191	Horizontally Transferred (medium confidence)
AZOLI_2473	Ancestral (low confidence)	AZOBR_140192	Ancestral (medium confidence)
AZOLI_2474	Ancestral (medium confidence)	AZOBR_140193	Unassigned
AZOLI_2475	Ancestral (high confidence)	AZOBR_140194	Ancestral (high confidence)
AZOLI_2476	Ancestral (high confidence)	AZOBR_140195	Unassigned
AZOLI_2477	Unassigned	AZOBR_140196	Unassigned
AZOLI_2478	Ancestral (medium confidence)	AZOBR_140197	Unassigned
AZOLI_2479	Ancestral (high confidence)	AZOBR_140198	Unassigned
AZOLI_2480	Horizontally Transferred (high confidence)	AZOBR_140199	Unassigned
AZOLI_2482	Ancestral (high confidence)	AZOBR_140200	Unassigned
AZOLI_2483	Unassigned	AZOBR_140201	Ancestral (high confidence)
AZOLI_2484	Ancestral (high confidence)	AZOBR_140202	Ancestral (low confidence)
AZOLI_2485	Horizontally Transferred (high confidence)	AZOBR_140203	Ancestral (low confidence)
AZOLI_2486	Unassigned	AZOBR_140204	Horizontally Transferred (high confidence)
AZOLI_2487	Horizontally Transferred (medium confidence)	AZOBR_140205	Ancestral (high confidence)
AZOLI_2488	Ancestral (medium confidence)	AZOBR_140206	Ancestral (medium confidence)
AZOLI_2490	Ancestral (high confidence)	AZOBR_140207	Unassigned
AZOLI_2491	Ancestral (high confidence)	AZOBR_140208	Ancestral (low confidence)
AZOLI_2492	Ancestral (high confidence)	AZOBR_140209	Unassigned
AZOLI_2493	Ancestral (medium confidence)	AZOBR_140210	Horizontally Transferred (high confidence)
AZOLI_2494	Ancestral (high confidence)	AZOBR_140211	Unassigned
AZOLI_2495	Ancestral (low confidence)	AZOBR_140212	Ancestral (medium confidence)
AZOLI_2496	Horizontally Transferred (high confidence)	AZOBR_140213	Ancestral (high confidence)
AZOLI_2497	Horizontally Transferred (high confidence)	AZOBR_140214	Horizontally Transferred (medium confidence)
AZOLI_2498	Horizontally Transferred (high confidence)	AZOBR_140215	Horizontally Transferred (high confidence)
AZOLI_2499	Ancestral (low confidence)	AZOBR_140216	Horizontally Transferred (high confidence)
AZOLI_2500	Ancestral (low confidence)	AZOBR_140217	Ancestral (medium confidence)
AZOLI_2501	Ancestral (high confidence)	AZOBR_140218	Unassigned
AZOLI_2502	Ancestral (medium confidence)	AZOBR_140219	Ancestral (medium confidence)
AZOLI_2503	Ancestral (low confidence)	AZOBR_140220	Ancestral (medium confidence)
AZOLI_2504	Ancestral (high confidence)	AZOBR_140221	Unassigned
AZOLI_2505	Ancestral (medium confidence)	AZOBR_140222	Ancestral (medium confidence)
AZOLI_2506	Unassigned	AZOBR_140223	Horizontally Transferred (high confidence)
AZOLI_2507	Horizontally Transferred (high confidence)	AZOBR_140224	Ancestral (low confidence)
AZOLI_2508	Ancestral (high confidence)	AZOBR_140225	Ancestral (medium confidence)
AZOLI_2509	Ancestral (medium confidence)	AZOBR_140226	Ancestral (medium confidence)
AZOLI_2510	Ancestral (high confidence)	AZOBR_140227	Unassigned
AZOLI_2511	Ancestral (high confidence)	AZOBR_140228	Ancestral (low confidence)
AZOLI_2512	Horizontally Transferred (high confidence)	AZOBR_140229	Ancestral (low confidence)
AZOLI_2513	Horizontally Transferred (high confidence)	AZOBR_140230	Ancestral (medium confidence)
AZOLI_2514	Unassigned	AZOBR_140231	Ancestral (medium confidence)
AZOLI_2515	Ancestral (medium confidence)	AZOBR_140232	Ancestral (low confidence)
AZOLI_2516	Ancestral (low confidence)	AZOBR_140233	Ancestral (high confidence)
AZOLI_2517	Ancestral (high confidence)	AZOBR_140234	Horizontally Transferred (high confidence)
AZOLI_2518	Ancestral (medium confidence)	AZOBR_140235	Ancestral (high confidence)
AZOLI_2519	Ancestral (high confidence)	AZOBR_140236	Unassigned
AZOLI_2520	Ancestral (low confidence)	AZOBR_140237	Unassigned
AZOLI_2521	Ancestral (low confidence)	AZOBR_140238	Ancestral (high confidence)
AZOLI_2522	Horizontally Transferred (low confidence)	AZOBR_140239	Ancestral (medium confidence)
AZOLI_2523	Ancestral (low confidence)	AZOBR_140240	Ancestral (high confidence)
AZOLI_2524	Ancestral (medium confidence)	AZOBR_140241	Ancestral (high confidence)
AZOLI_2525	Horizontally Transferred (high confidence)	AZOBR_140242	Ancestral (high confidence)
AZOLI_2526	Ancestral (high confidence)	AZOBR_140243	Ancestral (high confidence)
AZOLI_2527	Unassigned	AZOBR_140244	Ancestral (medium confidence)
AZOLI_2528	Ancestral (medium confidence)	AZOBR_140245	Ancestral (low confidence)
AZOLI_2529	Ancestral (medium confidence)	AZOBR_140246	Ancestral (medium confidence)
AZOLI_2530	Ancestral (low confidence)	AZOBR_140247	Ancestral (medium confidence)
AZOLI_2531	Ancestral (medium confidence)	AZOBR_140248	Ancestral (high confidence)
AZOLI_2532	Ancestral (medium confidence)	AZOBR_140249	Ancestral (high confidence)
AZOLI_2533	Ancestral (medium confidence)	AZOBR_140250	Ancestral (high confidence)
AZOLI_2534	Unassigned	AZOBR_140251	Ancestral (medium confidence)
AZOLI_2535	Unassigned	AZOBR_140252	Unassigned
AZOLI_2536	Ancestral (high confidence)	AZOBR_140253	Ancestral (high confidence)
AZOLI_2537	Ancestral (medium confidence)	AZOBR_140254	Ancestral (high confidence)
AZOLI_2538	Ancestral (medium confidence)	AZOBR_140255	Ancestral (high confidence)
AZOLI_2539	Horizontally Transferred (high confidence)	AZOBR_140256	Ancestral (high confidence)
AZOLI_2540	Ancestral (low confidence)	AZOBR_140257	Ancestral (high confidence)
AZOLI_2541	Horizontally Transferred (high confidence)	AZOBR_140258	Horizontally Transferred (high confidence)
AZOLI_2542	Unassigned	AZOBR_140259	Unassigned
AZOLI_2543	Horizontally Transferred (medium confidence)	AZOBR_140260	Horizontally Transferred (high confidence)
AZOLI_2544	Ancestral (low confidence)	AZOBR_140261	Ancestral (medium confidence)

AZOLI_2545	Horizontally Transferred (high confidence)	AZOBR_140262	Ancestral (low confidence)
AZOLI_2546	Horizontally Transferred (high confidence)	AZOBR_140263	Unassigned
AZOLI_2547	Ancestral (low confidence)	AZOBR_140264	Unassigned
AZOLI_2548	Unassigned	AZOBR_140265	Horizontally Transferred (low confidence)
AZOLI_2549	Horizontally Transferred (high confidence)	AZOBR_140266	Ancestral (low confidence)
AZOLI_2550	Horizontally Transferred (high confidence)	AZOBR_140267	Horizontally Transferred (high confidence)
AZOLI_2551	Ancestral (low confidence)	AZOBR_140268	Ancestral (high confidence)
AZOLI_2553	Ancestral (low confidence)	AZOBR_140269	Ancestral (medium confidence)
AZOLI_2554	Ancestral (medium confidence)	AZOBR_140270	Ancestral (low confidence)
AZOLI_2555	Ancestral (low confidence)	AZOBR_140271	Ancestral (medium confidence)
AZOLI_2556	Ancestral (medium confidence)	AZOBR_140272	Horizontally Transferred (high confidence)
AZOLI_2557	Ancestral (medium confidence)	AZOBR_140273	Ancestral (high confidence)
AZOLI_2558	Ancestral (medium confidence)	AZOBR_140274	Ancestral (medium confidence)
AZOLI_2560	Ancestral (medium confidence)	AZOBR_140275	Ancestral (medium confidence)
AZOLI_2561	Horizontally Transferred (high confidence)	AZOBR_140276	Ancestral (low confidence)
AZOLI_2562	Ancestral (low confidence)	AZOBR_140277	Ancestral (medium confidence)
AZOLI_2563	Ancestral (low confidence)	AZOBR_140278	Ancestral (medium confidence)
AZOLI_2564	Ancestral (medium confidence)	AZOBR_140279	Horizontally Transferred (high confidence)
AZOLI_2565	Horizontally Transferred (high confidence)	AZOBR_140280	Horizontally Transferred (high confidence)
AZOLI_2566	Horizontally Transferred (high confidence)	AZOBR_140281	Horizontally Transferred (high confidence)
AZOLI_2567	Ancestral (medium confidence)	AZOBR_140282	Ancestral (low confidence)
AZOLI_2569	Ancestral (low confidence)	AZOBR_140283	Unassigned
AZOLI_2570	Ancestral (medium confidence)	AZOBR_140284	Ancestral (medium confidence)
AZOLI_2571	Horizontally Transferred (medium confidence)	AZOBR_140285	Ancestral (medium confidence)
AZOLI_2572	Ancestral (low confidence)	AZOBR_140286	Horizontally Transferred (medium confidence)
AZOLI_2573	Ancestral (low confidence)	AZOBR_140287	Ancestral (low confidence)
AZOLI_2574	Ancestral (medium confidence)	AZOBR_140288	Ancestral (low confidence)
AZOLI_2575	Ancestral (medium confidence)	AZOBR_140289	Unassigned
AZOLI_2577	Unassigned	AZOBR_140290	Ancestral (high confidence)
AZOLI_2578	Ancestral (medium confidence)	AZOBR_140291	Ancestral (high confidence)
AZOLI_2579	Ancestral (high confidence)	AZOBR_140292	Ancestral (medium confidence)
AZOLI_2580	Ancestral (medium confidence)	AZOBR_140293	Ancestral (medium confidence)
AZOLI_2583	Ancestral (medium confidence)	AZOBR_140294	Ancestral (medium confidence)
AZOLI_2584	Ancestral (medium confidence)	AZOBR_140295	Unassigned
AZOLI_2585	Ancestral (medium confidence)	AZOBR_140296	Unassigned
AZOLI_2586	Ancestral (medium confidence)	AZOBR_140297	Ancestral (low confidence)
AZOLI_2587	Ancestral (medium confidence)	AZOBR_140298	Horizontally Transferred (high confidence)
AZOLI_2588	Ancestral (medium confidence)	AZOBR_140299	Unassigned
AZOLI_2589	Ancestral (medium confidence)	AZOBR_140300	Unassigned
AZOLI_2590	Ancestral (high confidence)	AZOBR_140301	Unassigned
AZOLI_2591	Horizontally Transferred (low confidence)	AZOBR_140302	Ancestral (low confidence)
AZOLI_2592	Ancestral (medium confidence)	AZOBR_140303	Ancestral (low confidence)
AZOLI_2594	Ancestral (medium confidence)	AZOBR_140304	Unassigned
AZOLI_2595	Ancestral (high confidence)	AZOBR_140305	Ancestral (high confidence)
AZOLI_2596	Horizontally Transferred (high confidence)	AZOBR_140306	Ancestral (low confidence)
AZOLI_2597	Unassigned	AZOBR_140307	Ancestral (medium confidence)
AZOLI_2598	Ancestral (medium confidence)	AZOBR_140308	Ancestral (high confidence)
AZOLI_2599	Horizontally Transferred (high confidence)	AZOBR_140309	Ancestral (high confidence)
AZOLI_2600	Ancestral (medium confidence)	AZOBR_140310	Horizontally Transferred (high confidence)
AZOLI_2601	Ancestral (high confidence)	AZOBR_140311	Ancestral (medium confidence)
AZOLI_2602	Ancestral (high confidence)	AZOBR_140312	Unassigned
AZOLI_2603	Ancestral (high confidence)	AZOBR_150001	Unassigned
AZOLI_2604	Ancestral (high confidence)	AZOBR_150002	Horizontally Transferred (high confidence)
AZOLI_2605	Ancestral (high confidence)	AZOBR_150003	Ancestral (low confidence)
AZOLI_2607	Ancestral (medium confidence)	AZOBR_150004	Ancestral (low confidence)
AZOLI_2608	Ancestral (medium confidence)	AZOBR_150005	Horizontally Transferred (high confidence)
AZOLI_2610	Ancestral (high confidence)	AZOBR_150006	Horizontally Transferred (high confidence)
AZOLI_2611	Ancestral (high confidence)	AZOBR_150007	Horizontally Transferred (medium confidence)
AZOLI_2612	Ancestral (high confidence)	AZOBR_150008	Horizontally Transferred (high confidence)
AZOLI_2613	Ancestral (high confidence)	AZOBR_150009	Horizontally Transferred (high confidence)
AZOLI_2614	Ancestral (low confidence)	AZOBR_150010	Ancestral (medium confidence)
AZOLI_2615	Unassigned	AZOBR_150011	Ancestral (low confidence)
AZOLI_2616	Horizontally Transferred (high confidence)	AZOBR_150012	Horizontally Transferred (medium confidence)
AZOLI_2618	Unassigned	AZOBR_150013	Ancestral (medium confidence)
AZOLI_2619	Ancestral (medium confidence)	AZOBR_150014	Unassigned
AZOLI_2620	Horizontally Transferred (high confidence)	AZOBR_150015	Horizontally Transferred (high confidence)
AZOLI_2621	Horizontally Transferred (high confidence)	AZOBR_150016	Ancestral (medium confidence)
AZOLI_2622	Ancestral (medium confidence)	AZOBR_150017	Ancestral (high confidence)
AZOLI_2623	Ancestral (high confidence)	AZOBR_150018	Horizontally Transferred (high confidence)
AZOLI_2624	Horizontally Transferred (high confidence)	AZOBR_150019	Ancestral (medium confidence)
AZOLI_2625	Ancestral (low confidence)	AZOBR_150020	Ancestral (low confidence)
AZOLI_2626	Horizontally Transferred (high confidence)	AZOBR_150021	Ancestral (high confidence)
AZOLI_2627	Ancestral (medium confidence)	AZOBR_150022	Ancestral (high confidence)
AZOLI_2628	Ancestral (high confidence)	AZOBR_150023	Ancestral (high confidence)
AZOLI_2629	Ancestral (low confidence)	AZOBR_150024	Unassigned

AZOLI_2630	Ancestral (high confidence)	AZOBR_150025	Ancestral (high confidence)
AZOLI_2631	Unassigned	AZOBR_150026	Ancestral (high confidence)
AZOLI_2632	Ancestral (high confidence)	AZOBR_150027	Ancestral (medium confidence)
AZOLI_2633	Ancestral (high confidence)	AZOBR_150028	Ancestral (high confidence)
AZOLI_2634	Unassigned	AZOBR_150029	Ancestral (low confidence)
AZOLI_2635	Ancestral (low confidence)	AZOBR_150030	Ancestral (medium confidence)
AZOLI_2636	Horizontally Transferred (high confidence)	AZOBR_150031	Ancestral (high confidence)
AZOLI_2637	Ancestral (high confidence)	AZOBR_150032	Unassigned
AZOLI_2638	Horizontally Transferred (high confidence)	AZOBR_150033	Ancestral (low confidence)
AZOLI_2639	Ancestral (medium confidence)	AZOBR_150034	Ancestral (high confidence)
AZOLI_2640	Horizontally Transferred (high confidence)	AZOBR_150035	Ancestral (high confidence)
AZOLI_2641	Ancestral (high confidence)	AZOBR_150036	Unassigned
AZOLI_2642	Ancestral (low confidence)	AZOBR_150037	Ancestral (medium confidence)
AZOLI_2644	Horizontally Transferred (high confidence)	AZOBR_150038	Ancestral (low confidence)
AZOLI_2645	Unassigned	AZOBR_150039	Ancestral (high confidence)
AZOLI_2646	Ancestral (medium confidence)	AZOBR_150040	Ancestral (medium confidence)
AZOLI_2647	Horizontally Transferred (high confidence)	AZOBR_150041	Horizontally Transferred (high confidence)
AZOLI_2648	Ancestral (medium confidence)	AZOBR_150042	Ancestral (medium confidence)
AZOLI_2649	Ancestral (medium confidence)	AZOBR_150043	Ancestral (high confidence)
AZOLI_2650	Ancestral (high confidence)	AZOBR_150044	Ancestral (high confidence)
AZOLI_2651	Ancestral (high confidence)	AZOBR_150045	Unassigned
AZOLI_2652	Ancestral (high confidence)	AZOBR_150046	Ancestral (medium confidence)
AZOLI_2653	Horizontally Transferred (high confidence)	AZOBR_150047	Ancestral (high confidence)
AZOLI_2654	Ancestral (low confidence)	AZOBR_150048	Ancestral (high confidence)
AZOLI_2655	Horizontally Transferred (high confidence)	AZOBR_150049	Horizontally Transferred (high confidence)
AZOLI_2656	Unassigned	AZOBR_150050	Unassigned
AZOLI_2657	Ancestral (low confidence)	AZOBR_150051	Unassigned
AZOLI_2658	Horizontally Transferred (high confidence)	AZOBR_150052	Ancestral (low confidence)
AZOLI_2659	Ancestral (high confidence)	AZOBR_150053	Ancestral (medium confidence)
AZOLI_2660	Ancestral (high confidence)	AZOBR_150054	Ancestral (medium confidence)
AZOLI_2661	Horizontally Transferred (high confidence)	AZOBR_150055	Ancestral (low confidence)
AZOLI_2663	Ancestral (medium confidence)	AZOBR_150056	Ancestral (high confidence)
AZOLI_2664	Ancestral (high confidence)	AZOBR_150057	Unassigned
AZOLI_2665	Ancestral (low confidence)	AZOBR_150058	Ancestral (medium confidence)
AZOLI_2666	Ancestral (low confidence)	AZOBR_150059	Ancestral (high confidence)
AZOLI_2667	Ancestral (low confidence)	AZOBR_150060	Ancestral (medium confidence)
AZOLI_2668	Ancestral (medium confidence)	AZOBR_150061	Ancestral (medium confidence)
AZOLI_2669	Ancestral (low confidence)	AZOBR_150062	Ancestral (high confidence)
AZOLI_2670	Ancestral (low confidence)	AZOBR_150063	Ancestral (high confidence)
AZOLI_2671	Ancestral (low confidence)	AZOBR_150064	Horizontally Transferred (high confidence)
AZOLI_2672	Ancestral (medium confidence)	AZOBR_150065	Ancestral (medium confidence)
AZOLI_2673	Horizontally Transferred (medium confidence)	AZOBR_150066	Unassigned
AZOLI_2674	Horizontally Transferred (high confidence)	AZOBR_150067	Ancestral (medium confidence)
AZOLI_2675	Ancestral (high confidence)	AZOBR_150068	Unassigned
AZOLI_2676	Ancestral (medium confidence)	AZOBR_150069	Ancestral (low confidence)
AZOLI_2677	Ancestral (low confidence)	AZOBR_150070	Unassigned
AZOLI_2678	Ancestral (low confidence)	AZOBR_150071	Ancestral (low confidence)
AZOLI_2679	Horizontally Transferred (medium confidence)	AZOBR_150072	Ancestral (medium confidence)
AZOLI_2680	Horizontally Transferred (high confidence)	AZOBR_150073	Ancestral (medium confidence)
AZOLI_2681	Ancestral (low confidence)	AZOBR_150074	Ancestral (high confidence)
AZOLI_2682	Horizontally Transferred (high confidence)	AZOBR_150075	Ancestral (medium confidence)
AZOLI_2683	Horizontally Transferred (low confidence)	AZOBR_150076	Ancestral (high confidence)
AZOLI_2684	Unassigned	AZOBR_150077	Ancestral (high confidence)
AZOLI_2685	Ancestral (high confidence)	AZOBR_150078	Unassigned
AZOLI_2686	Ancestral (low confidence)	AZOBR_150079	Ancestral (high confidence)
AZOLI_2688	Ancestral (medium confidence)	AZOBR_150080	Ancestral (high confidence)
AZOLI_2689	Unassigned	AZOBR_150081	Ancestral (high confidence)
AZOLI_2690	Ancestral (medium confidence)	AZOBR_150082	Unassigned
AZOLI_2691	Ancestral (medium confidence)	AZOBR_150083	Ancestral (low confidence)
AZOLI_2692	Ancestral (high confidence)	AZOBR_150084	Ancestral (high confidence)
AZOLI_2693	Ancestral (medium confidence)	AZOBR_150085	Horizontally Transferred (high confidence)
AZOLI_2694	Ancestral (high confidence)	AZOBR_150086	Ancestral (low confidence)
AZOLI_2695	Ancestral (high confidence)	AZOBR_150087	Ancestral (medium confidence)
AZOLI_2697	Ancestral (medium confidence)	AZOBR_150088	Ancestral (high confidence)
AZOLI_2698	Horizontally Transferred (high confidence)	AZOBR_150089	Ancestral (medium confidence)
AZOLI_2699	Ancestral (medium confidence)	AZOBR_150090	Ancestral (medium confidence)
AZOLI_2700	Ancestral (high confidence)	AZOBR_150091	Horizontally Transferred (high confidence)
AZOLI_2702	Ancestral (high confidence)	AZOBR_150092	Ancestral (medium confidence)
AZOLI_2703	Ancestral (high confidence)	AZOBR_150093	Ancestral (medium confidence)
AZOLI_2705	Ancestral (medium confidence)	AZOBR_150094	Ancestral (high confidence)
AZOLI_2706	Ancestral (high confidence)	AZOBR_150095	Unassigned
AZOLI_2707	Ancestral (medium confidence)	AZOBR_150096	Ancestral (low confidence)
AZOLI_2708	Ancestral (low confidence)	AZOBR_150097	Ancestral (medium confidence)
AZOLI_2709	Ancestral (low confidence)	AZOBR_150098	Unassigned
AZOLI_2710	Ancestral (high confidence)	AZOBR_150099	Horizontally Transferred (low confidence)

AZOLI_2711	Ancestral (medium confidence)	AZOBR_150100	Horizontally Transferred (low confidence)
AZOLI_2712	Ancestral (low confidence)	AZOBR_150101	Horizontally Transferred (high confidence)
AZOLI_2713	Unassigned	AZOBR_150102	Ancestral (low confidence)
AZOLI_2714	Ancestral (low confidence)	AZOBR_150103	Unassigned
AZOLI_2715	Ancestral (medium confidence)	AZOBR_150104	Ancestral (medium confidence)
AZOLI_2716	Ancestral (high confidence)	AZOBR_150105	Ancestral (high confidence)
AZOLI_2717	Ancestral (medium confidence)	AZOBR_150106	Unassigned
AZOLI_2718	Ancestral (high confidence)	AZOBR_150107	Ancestral (medium confidence)
AZOLI_2719	Ancestral (high confidence)	AZOBR_150108	Ancestral (high confidence)
AZOLI_2720	Ancestral (high confidence)	AZOBR_150109	Ancestral (low confidence)
AZOLI_2721	Ancestral (low confidence)	AZOBR_150110	Ancestral (medium confidence)
AZOLI_2722	Horizontally Transferred (high confidence)	AZOBR_150111	Unassigned
AZOLI_2723	Ancestral (low confidence)	AZOBR_150112	Ancestral (low confidence)
AZOLI_2724	Ancestral (medium confidence)	AZOBR_150113	Ancestral (medium confidence)
AZOLI_2725	Unassigned	AZOBR_150114	Ancestral (low confidence)
AZOLI_2726	Ancestral (low confidence)	AZOBR_150115	Unassigned
AZOLI_2727	Unassigned	AZOBR_150116	Unassigned
AZOLI_2728	Horizontally Transferred (high confidence)	AZOBR_150117	Unassigned
AZOLI_2729	Horizontally Transferred (high confidence)	AZOBR_150118	Horizontally Transferred (high confidence)
AZOLI_2730	Horizontally Transferred (high confidence)	AZOBR_150119	Unassigned
AZOLI_2731	Horizontally Transferred (high confidence)	AZOBR_150120	Ancestral (low confidence)
AZOLI_2732	Ancestral (high confidence)	AZOBR_150121	Ancestral (low confidence)
AZOLI_2733	Ancestral (low confidence)	AZOBR_150122	Unassigned
AZOLI_2734	Ancestral (low confidence)	AZOBR_150123	Horizontally Transferred (high confidence)
AZOLI_2735	Horizontally Transferred (high confidence)	AZOBR_150124	Horizontally Transferred (high confidence)
AZOLI_2736	Horizontally Transferred (high confidence)	AZOBR_150125	Horizontally Transferred (high confidence)
AZOLI_2737	Ancestral (medium confidence)	AZOBR_150126	Horizontally Transferred (high confidence)
AZOLI_2738	Horizontally Transferred (high confidence)	AZOBR_150127	Horizontally Transferred (high confidence)
AZOLI_2739	Ancestral (low confidence)	AZOBR_150128	Ancestral (high confidence)
AZOLI_2740	Ancestral (low confidence)	AZOBR_150129	Ancestral (high confidence)
AZOLI_2741	Unassigned	AZOBR_150130	Ancestral (high confidence)
AZOLI_2742	Ancestral (high confidence)	AZOBR_150131	Unassigned
AZOLI_2743	Ancestral (medium confidence)	AZOBR_150132	Ancestral (high confidence)
AZOLI_2744	Ancestral (low confidence)	AZOBR_150133	Horizontally Transferred (high confidence)
AZOLI_2745	Ancestral (medium confidence)	AZOBR_150134	Ancestral (low confidence)
AZOLI_2746	Ancestral (medium confidence)	AZOBR_150135	Ancestral (medium confidence)
AZOLI_2747	Ancestral (high confidence)	AZOBR_150136	Unassigned
AZOLI_2748	Ancestral (medium confidence)	AZOBR_150137	Horizontally Transferred (medium confidence)
AZOLI_2749	Horizontally Transferred (high confidence)	AZOBR_150138	Unassigned
AZOLI_2751	Ancestral (high confidence)	AZOBR_150139	Ancestral (medium confidence)
AZOLI_2752	Unassigned	AZOBR_150140	Ancestral (low confidence)
AZOLI_2753	Ancestral (low confidence)	AZOBR_150141	Ancestral (medium confidence)
AZOLI_2754	Ancestral (low confidence)	AZOBR_150142	Unassigned
AZOLI_2755	Horizontally Transferred (high confidence)	AZOBR_150143	Ancestral (medium confidence)
AZOLI_2756	Ancestral (high confidence)	AZOBR_150144	Ancestral (high confidence)
AZOLI_2757	Ancestral (high confidence)	AZOBR_150145	Ancestral (high confidence)
AZOLI_2758	Ancestral (high confidence)	AZOBR_150146	Ancestral (medium confidence)
AZOLI_2759	Horizontally Transferred (low confidence)	AZOBR_150147	Ancestral (high confidence)
AZOLI_2760	Horizontally Transferred (low confidence)	AZOBR_150148	Unassigned
AZOLI_2761	Ancestral (high confidence)	AZOBR_150149	Ancestral (medium confidence)
AZOLI_2762	Ancestral (low confidence)	AZOBR_150150	Ancestral (high confidence)
AZOLI_2763	Horizontally Transferred (medium confidence)	AZOBR_150151	Horizontally Transferred (high confidence)
AZOLI_2764	Unassigned	AZOBR_150152	Horizontally Transferred (high confidence)
AZOLI_2765	Horizontally Transferred (low confidence)	AZOBR_150153	Ancestral (medium confidence)
AZOLI_2766	Horizontally Transferred (low confidence)	AZOBR_150154	Ancestral (medium confidence)
AZOLI_2767	Horizontally Transferred (high confidence)	AZOBR_150155	Unassigned
AZOLI_2768	Ancestral (medium confidence)	AZOBR_150156	Ancestral (low confidence)
AZOLI_2769	Unassigned	AZOBR_150157	Horizontally Transferred (high confidence)
AZOLI_2770	Ancestral (low confidence)	AZOBR_150158	Horizontally Transferred (low confidence)
AZOLI_2771	Ancestral (low confidence)	AZOBR_150159	Unassigned
AZOLI_2772	Ancestral (low confidence)	AZOBR_150160	Ancestral (medium confidence)
AZOLI_2773	Ancestral (high confidence)	AZOBR_150161	Unassigned
AZOLI_2774	Ancestral (high confidence)	AZOBR_150162	Unassigned
AZOLI_2775	Ancestral (medium confidence)	AZOBR_150163	Ancestral (medium confidence)
AZOLI_2776	Ancestral (high confidence)	AZOBR_150164	Horizontally Transferred (high confidence)
AZOLI_2777	Ancestral (low confidence)	AZOBR_150165	Ancestral (medium confidence)
AZOLI_2778	Ancestral (medium confidence)	AZOBR_150166	Ancestral (low confidence)
AZOLI_2779	Unassigned	AZOBR_150167	Horizontally Transferred (high confidence)
AZOLI_2781	Horizontally Transferred (high confidence)	AZOBR_150168	Ancestral (low confidence)
AZOLI_2782	Horizontally Transferred (high confidence)	AZOBR_150169	Ancestral (high confidence)
AZOLI_2783	Ancestral (high confidence)	AZOBR_150170	Ancestral (low confidence)
AZOLI_2784	Ancestral (medium confidence)	AZOBR_150171	Ancestral (high confidence)
AZOLI_2785	Ancestral (low confidence)	AZOBR_150172	Unassigned
AZOLI_2786	Ancestral (high confidence)	AZOBR_150173	Ancestral (high confidence)
AZOLI_2787	Ancestral (medium confidence)	AZOBR_150174	Unassigned

AZOLI_2788	Ancestral (high confidence)	AZOBR_150175	Ancestral (low confidence)
AZOLI_2789	Unassigned	AZOBR_150176	Ancestral (high confidence)
AZOLI_2790	Horizontally Transferred (medium confidence)	AZOBR_150177	Ancestral (medium confidence)
AZOLI_2791	Ancestral (low confidence)	AZOBR_150178	Ancestral (high confidence)
AZOLI_2792	Horizontally Transferred (low confidence)	AZOBR_150179	Ancestral (high confidence)
AZOLI_2793	Unassigned	AZOBR_150180	Ancestral (high confidence)
AZOLI_2794	Ancestral (medium confidence)	AZOBR_150181	Ancestral (high confidence)
AZOLI_2795	Ancestral (low confidence)	AZOBR_150182	Ancestral (medium confidence)
AZOLI_2796	Ancestral (medium confidence)	AZOBR_150183	Ancestral (high confidence)
AZOLI_2797	Horizontally Transferred (high confidence)	AZOBR_150184	Unassigned
AZOLI_2798	Unassigned	AZOBR_150185	Ancestral (high confidence)
AZOLI_2799	Ancestral (medium confidence)	AZOBR_150186	Unassigned
AZOLI_2800	Unassigned	AZOBR_150187	Ancestral (high confidence)
AZOLI_2801	Ancestral (medium confidence)	AZOBR_150188	Ancestral (high confidence)
AZOLI_2802	Ancestral (medium confidence)	AZOBR_150189	Ancestral (medium confidence)
AZOLI_2803	Ancestral (high confidence)	AZOBR_150190	Unassigned
AZOLI_2804	Ancestral (medium confidence)	AZOBR_150191	Ancestral (high confidence)
AZOLI_2805	Ancestral (medium confidence)	AZOBR_150192	Ancestral (medium confidence)
AZOLI_2806	Unassigned	AZOBR_150193	Ancestral (medium confidence)
AZOLI_2807	Ancestral (high confidence)	AZOBR_150194	Ancestral (high confidence)
AZOLI_2808	Horizontally Transferred (high confidence)	AZOBR_150195	Ancestral (low confidence)
AZOLI_2809	Ancestral (medium confidence)	AZOBR_150196	Ancestral (high confidence)
AZOLI_2810	Horizontally Transferred (high confidence)	AZOBR_150197	Ancestral (high confidence)
AZOLI_2811	Ancestral (medium confidence)	AZOBR_150198	Ancestral (high confidence)
AZOLI_2812	Ancestral (low confidence)	AZOBR_150199	Ancestral (high confidence)
AZOLI_2813	Ancestral (low confidence)	AZOBR_150200	Unassigned
AZOLI_2814	Ancestral (high confidence)	AZOBR_150201	Ancestral (high confidence)
AZOLI_2817	Ancestral (medium confidence)	AZOBR_150202	Ancestral (high confidence)
AZOLI_2818	Horizontally Transferred (high confidence)	AZOBR_150203	Ancestral (high confidence)
AZOLI_2819	Ancestral (high confidence)	AZOBR_150204	Ancestral (medium confidence)
AZOLI_2820	Ancestral (medium confidence)	AZOBR_150205	Horizontally Transferred (high confidence)
AZOLI_2821	Horizontally Transferred (high confidence)	AZOBR_150206	Ancestral (low confidence)
AZOLI_2822	Horizontally Transferred (high confidence)	AZOBR_150207	Ancestral (high confidence)
AZOLI_2823	Horizontally Transferred (high confidence)	AZOBR_150208	Ancestral (high confidence)
AZOLI_2824	Ancestral (medium confidence)	AZOBR_150209	Ancestral (high confidence)
AZOLI_2825	Ancestral (low confidence)	AZOBR_150210	Ancestral (high confidence)
AZOLI_2826	Ancestral (low confidence)	AZOBR_150211	Ancestral (medium confidence)
AZOLI_2827	Ancestral (medium confidence)	AZOBR_150212	Ancestral (high confidence)
AZOLI_2828	Unassigned	AZOBR_150213	Ancestral (high confidence)
AZOLI_2829	Ancestral (high confidence)	AZOBR_150214	Ancestral (low confidence)
AZOLI_2830	Unassigned	AZOBR_150215	Unassigned
AZOLI_2831	Ancestral (low confidence)	AZOBR_150216	Unassigned
AZOLI_2832	Ancestral (high confidence)	AZOBR_150217	Ancestral (medium confidence)
AZOLI_2833	Ancestral (high confidence)	AZOBR_150218	Ancestral (low confidence)
AZOLI_2834	Horizontally Transferred (low confidence)	AZOBR_150219	Ancestral (low confidence)
AZOLI_2835	Ancestral (high confidence)	AZOBR_150220	Unassigned
AZOLI_2836	Ancestral (medium confidence)	AZOBR_150221	Ancestral (low confidence)
AZOLI_2837	Ancestral (high confidence)	AZOBR_150222	Ancestral (low confidence)
AZOLI_2838	Horizontally Transferred (high confidence)	AZOBR_150223	Unassigned
AZOLI_2839	Ancestral (low confidence)	AZOBR_150224	Ancestral (high confidence)
AZOLI_2840	Unassigned	AZOBR_150225	Ancestral (high confidence)
AZOLI_2841	Ancestral (medium confidence)	AZOBR_150226	Ancestral (medium confidence)
AZOLI_2842	Ancestral (high confidence)	AZOBR_150227	Ancestral (medium confidence)
AZOLI_2843	Ancestral (high confidence)	AZOBR_150228	Horizontally Transferred (medium confidence)
AZOLI_2844	Ancestral (low confidence)	AZOBR_150229	Ancestral (low confidence)
AZOLI_2845	Ancestral (medium confidence)	AZOBR_150230	Ancestral (low confidence)
AZOLI_2846	Ancestral (low confidence)	AZOBR_150231	Ancestral (high confidence)
AZOLI_2847	Horizontally Transferred (high confidence)	AZOBR_150232	Ancestral (low confidence)
AZOLI_2848	Ancestral (low confidence)	AZOBR_150233	Ancestral (medium confidence)
AZOLI_2849	Ancestral (high confidence)	AZOBR_150234	Ancestral (medium confidence)
AZOLI_2850	Ancestral (high confidence)	AZOBR_150235	Ancestral (high confidence)
AZOLI_2852	Horizontally Transferred (high confidence)	AZOBR_150236	Horizontally Transferred (high confidence)
AZOLI_2853	Ancestral (medium confidence)	AZOBR_150237	Ancestral (high confidence)
AZOLI_2854	Ancestral (medium confidence)	AZOBR_150238	Ancestral (medium confidence)
AZOLI_2855	Ancestral (high confidence)	AZOBR_150239	Ancestral (high confidence)
AZOLI_2856	Horizontally Transferred (high confidence)	AZOBR_150240	Ancestral (high confidence)
AZOLI_2857	Ancestral (high confidence)	AZOBR_150241	Ancestral (high confidence)
AZOLI_2858	Ancestral (high confidence)	AZOBR_150242	Ancestral (high confidence)
AZOLI_2859	Ancestral (high confidence)	AZOBR_150243	Ancestral (medium confidence)
AZOLI_2860	Ancestral (high confidence)	AZOBR_150244	Ancestral (high confidence)
AZOLI_2861	Ancestral (high confidence)	AZOBR_150245	Ancestral (high confidence)
AZOLI_2862	Ancestral (high confidence)	AZOBR_150246	Ancestral (high confidence)
AZOLI_2863	Ancestral (high confidence)	AZOBR_150247	Ancestral (medium confidence)
AZOLI_2864	Ancestral (low confidence)	AZOBR_150248	Ancestral (high confidence)
AZOLI_2865	Horizontally Transferred (high confidence)	AZOBR_150249	Ancestral (medium confidence)

AZOLI_2866	Horizontally Transferred (high confidence)	AZOBR_150250	Ancestral (high confidence)
AZOLI_2868	Unassigned	AZOBR_150251	Ancestral (medium confidence)
AZOLI_2869	Ancestral (low confidence)	AZOBR_150252	Ancestral (high confidence)
AZOLI_2870	Ancestral (high confidence)	AZOBR_150253	Ancestral (high confidence)
AZOLI_2871	Ancestral (medium confidence)	AZOBR_150254	Ancestral (high confidence)
AZOLI_2872	Ancestral (high confidence)	AZOBR_150255	Ancestral (high confidence)
AZOLI_2873	Ancestral (high confidence)	AZOBR_150256	Ancestral (high confidence)
AZOLI_2874	Ancestral (high confidence)	AZOBR_150257	Ancestral (high confidence)
AZOLI_2875	Ancestral (high confidence)	AZOBR_150258	Unassigned
AZOLI_2876	Ancestral (medium confidence)	AZOBR_160001	Ancestral (medium confidence)
AZOLI_2877	Ancestral (medium confidence)	AZOBR_160002	Ancestral (high confidence)
AZOLI_2879	Unassigned	AZOBR_160003	Ancestral (high confidence)
AZOLI_2881	Ancestral (medium confidence)	AZOBR_160004	Ancestral (high confidence)
AZOLI_2882	Ancestral (low confidence)	AZOBR_160005	Ancestral (low confidence)
AZOLI_2883	Ancestral (low confidence)	AZOBR_160006	Ancestral (medium confidence)
AZOLI_2884	Unassigned	AZOBR_160007	Ancestral (high confidence)
AZOLI_2886	Ancestral (medium confidence)	AZOBR_160008	Unassigned
AZOLI_2887	Unassigned	AZOBR_160009	Ancestral (medium confidence)
AZOLI_2889	Ancestral (medium confidence)	AZOBR_160010	Ancestral (medium confidence)
AZOLI_2890	Ancestral (high confidence)	AZOBR_160011	Ancestral (medium confidence)
AZOLI_2891	Horizontally Transferred (high confidence)	AZOBR_160012	Ancestral (medium confidence)
AZOLI_2893	Ancestral (high confidence)	AZOBR_160013	Ancestral (high confidence)
AZOLI_2894	Ancestral (high confidence)	AZOBR_160014	Ancestral (medium confidence)
AZOLI_2896	Ancestral (high confidence)	AZOBR_160015	Ancestral (medium confidence)
AZOLI_2897	Unassigned	AZOBR_160016	Unassigned
AZOLI_2898	Ancestral (medium confidence)	AZOBR_160017	Ancestral (high confidence)
AZOLI_2899	Ancestral (high confidence)	AZOBR_160018	Unassigned
AZOLI_2900	Ancestral (medium confidence)	AZOBR_160019	Unassigned
AZOLI_2901	Ancestral (high confidence)	AZOBR_160020	Ancestral (medium confidence)
AZOLI_2902	Unassigned	AZOBR_160021	Ancestral (low confidence)
AZOLI_2903	Ancestral (high confidence)	AZOBR_160022	Unassigned
AZOLI_2904	Ancestral (medium confidence)	AZOBR_160023	Unassigned
AZOLI_2905	Ancestral (medium confidence)	AZOBR_160024	Unassigned
AZOLI_2906	Horizontally Transferred (high confidence)	AZOBR_160025	Unassigned
AZOLI_2907	Horizontally Transferred (high confidence)	AZOBR_160026	Ancestral (medium confidence)
AZOLI_2908	Ancestral (low confidence)	AZOBR_160027	Unassigned
AZOLI_2909	Ancestral (medium confidence)	AZOBR_160028	Unassigned
AZOLI_2910	Horizontally Transferred (low confidence)	AZOBR_160029	Horizontally Transferred (high confidence)
AZOLI_2911	Ancestral (low confidence)	AZOBR_160030	Unassigned
AZOLI_2913	Horizontally Transferred (high confidence)	AZOBR_160031	Ancestral (medium confidence)
AZOLI_2915	Unassigned	AZOBR_160032	Horizontally Transferred (high confidence)
AZOLI_2916	Horizontally Transferred (high confidence)	AZOBR_160033	Ancestral (medium confidence)
AZOLI_2917	Horizontally Transferred (high confidence)	AZOBR_160034	Unassigned
AZOLI_2918	Horizontally Transferred (high confidence)	AZOBR_160035	Unassigned
AZOLI_2919	Ancestral (medium confidence)	AZOBR_160036	Ancestral (medium confidence)
AZOLI_2920	Horizontally Transferred (high confidence)	AZOBR_160037	Unassigned
AZOLI_2921	Ancestral (high confidence)	AZOBR_160038	Ancestral (medium confidence)
AZOLI_2922	Ancestral (medium confidence)	AZOBR_160039	Ancestral (low confidence)
AZOLI_2923	Unassigned	AZOBR_160040	Unassigned
AZOLI_2924	Unassigned	AZOBR_160041	Ancestral (low confidence)
AZOLI_2925	Ancestral (medium confidence)	AZOBR_160042	Ancestral (low confidence)
AZOLI_2926	Ancestral (high confidence)	AZOBR_160043	Ancestral (low confidence)
AZOLI_2927	Ancestral (medium confidence)	AZOBR_160044	Horizontally Transferred (high confidence)
AZOLI_2928	Ancestral (medium confidence)	AZOBR_160045	Ancestral (low confidence)
AZOLI_2929	Ancestral (medium confidence)	AZOBR_160046	Ancestral (low confidence)
AZOLI_2930	Ancestral (high confidence)	AZOBR_160047	Unassigned
AZOLI_2931	Ancestral (medium confidence)	AZOBR_160048	Horizontally Transferred (high confidence)
AZOLI_2932	Horizontally Transferred (low confidence)	AZOBR_160049	Ancestral (low confidence)
AZOLI_2933	Ancestral (medium confidence)	AZOBR_160050	Ancestral (low confidence)
AZOLI_2934	Ancestral (medium confidence)	AZOBR_160051	Ancestral (high confidence)
AZOLI_2935	Unassigned	AZOBR_160052	Ancestral (low confidence)
AZOLI_2936	Ancestral (medium confidence)	AZOBR_160053	Horizontally Transferred (high confidence)
AZOLI_2937	Ancestral (high confidence)	AZOBR_160054	Unassigned
AZOLI_2938	Ancestral (high confidence)	AZOBR_160055	Ancestral (low confidence)
AZOLI_2939	Horizontally Transferred (high confidence)	AZOBR_160056	Ancestral (low confidence)
AZOLI_2940	Ancestral (medium confidence)	AZOBR_160057	Ancestral (low confidence)
AZOLI_2941	Ancestral (high confidence)	AZOBR_160058	Horizontally Transferred (high confidence)
AZOLI_2942	Unassigned	AZOBR_160059	Horizontally Transferred (high confidence)
AZOLI_2943	Ancestral (medium confidence)	AZOBR_160060	Horizontally Transferred (high confidence)
AZOLI_2944	Ancestral (high confidence)	AZOBR_160061	Horizontally Transferred (high confidence)
AZOLI_2945	Ancestral (low confidence)	AZOBR_160062	Horizontally Transferred (high confidence)
AZOLI_2946	Ancestral (high confidence)	AZOBR_160063	Horizontally Transferred (high confidence)
AZOLI_2947	Ancestral (medium confidence)	AZOBR_160064	Horizontally Transferred (high confidence)
AZOLI_2948	Ancestral (medium confidence)	AZOBR_160065	Horizontally Transferred (high confidence)
AZOLI_2949	Ancestral (medium confidence)	AZOBR_160066	Horizontally Transferred (high confidence)

AZOLI_2950	Ancestral (high confidence)	AZOBR_160067	Ancestral (low confidence)
AZOLI_2951	Ancestral (high confidence)	AZOBR_160068	Ancestral (low confidence)
AZOLI_2952	Ancestral (low confidence)	AZOBR_160069	Unassigned
AZOLI_2953	Ancestral (high confidence)	AZOBR_160070	Ancestral (medium confidence)
AZOLI_2954	Ancestral (medium confidence)	AZOBR_160071	Horizontally Transferred (high confidence)
AZOLI_2955	Ancestral (medium confidence)	AZOBR_160072	Unassigned
AZOLI_2956	Ancestral (medium confidence)	AZOBR_160073	Ancestral (low confidence)
AZOLI_2957	Ancestral (low confidence)	AZOBR_160074	Horizontally Transferred (high confidence)
AZOLI_2958	Ancestral (high confidence)	AZOBR_160075	Horizontally Transferred (high confidence)
AZOLI_2959	Ancestral (high confidence)	AZOBR_160076	Ancestral (low confidence)
AZOLI_2960	Ancestral (high confidence)	AZOBR_160077	Ancestral (low confidence)
AZOLI_2961	Ancestral (high confidence)	AZOBR_160078	Ancestral (low confidence)
AZOLI_2962	Ancestral (medium confidence)	AZOBR_160079	Horizontally Transferred (high confidence)
AZOLI_2963	Horizontally Transferred (high confidence)	AZOBR_170001	Ancestral (high confidence)
AZOLI_2965	Ancestral (high confidence)	AZOBR_170002	Horizontally Transferred (high confidence)
AZOLI_2966	Ancestral (high confidence)	AZOBR_170003	Horizontally Transferred (high confidence)
AZOLI_2968	Ancestral (high confidence)	AZOBR_170004	Ancestral (high confidence)
AZOLI_2969	Ancestral (medium confidence)	AZOBR_170005	Ancestral (high confidence)
AZOLI_2970	Horizontally Transferred (high confidence)	AZOBR_170006	Ancestral (medium confidence)
AZOLI_2971	Horizontally Transferred (high confidence)	AZOBR_170007	Ancestral (high confidence)
AZOLI_2972	Horizontally Transferred (high confidence)	AZOBR_170008	Ancestral (low confidence)
AZOLI_2973	Horizontally Transferred (high confidence)	AZOBR_170009	Ancestral (medium confidence)
AZOLI_2974	Ancestral (low confidence)	AZOBR_170010	Ancestral (high confidence)
AZOLI_2975	Unassigned	AZOBR_170011	Ancestral (high confidence)
AZOLI_2976	Unassigned	AZOBR_180001	Unassigned
AZOLI_2977	Unassigned	AZOBR_180002	Ancestral (high confidence)
AZOLI_2979	Ancestral (high confidence)	AZOBR_180003	Ancestral (high confidence)
AZOLI_2980	Ancestral (high confidence)	AZOBR_180004	Ancestral (high confidence)
AZOLI_2981	Unassigned	AZOBR_180005	Ancestral (high confidence)
AZOLI_2982	Ancestral (high confidence)	AZOBR_180006	Ancestral (medium confidence)
AZOLI_2983	Ancestral (high confidence)	AZOBR_180007	Ancestral (medium confidence)
AZOLI_2984	Ancestral (medium confidence)	AZOBR_180008	Ancestral (low confidence)
AZOLI_2985	Ancestral (medium confidence)	AZOBR_180009	Ancestral (high confidence)
AZOLI_2986	Ancestral (high confidence)	AZOBR_180010	Ancestral (low confidence)
AZOLI_2987	Ancestral (high confidence)	AZOBR_180011	Ancestral (low confidence)
AZOLI_2988	Ancestral (medium confidence)	AZOBR_180012	Ancestral (high confidence)
AZOLI_2989	Horizontally Transferred (high confidence)	AZOBR_180013	Horizontally Transferred (high confidence)
AZOLI_2990	Ancestral (high confidence)	AZOBR_180014	Unassigned
AZOLI_2991	Ancestral (high confidence)	AZOBR_180015	Horizontally Transferred (high confidence)
AZOLI_2992	Ancestral (medium confidence)	AZOBR_180016	Ancestral (high confidence)
AZOLI_2993	Horizontally Transferred (medium confidence)	AZOBR_180017	Ancestral (high confidence)
AZOLI_2994	Horizontally Transferred (medium confidence)	AZOBR_180018	Ancestral (high confidence)
AZOLI_2995	Ancestral (medium confidence)	AZOBR_180019	Ancestral (high confidence)
AZOLI_2997	Ancestral (medium confidence)	AZOBR_180020	Ancestral (high confidence)
AZOLI_2998	Ancestral (high confidence)	AZOBR_180021	Ancestral (high confidence)
AZOLI_2999	Ancestral (high confidence)	AZOBR_180022	Ancestral (high confidence)
AZOLI_3000	Ancestral (medium confidence)	AZOBR_180023	Horizontally Transferred (high confidence)
AZOLI_3001	Ancestral (medium confidence)	AZOBR_180024	Ancestral (high confidence)
AZOLI_3002	Ancestral (low confidence)	AZOBR_180025	Ancestral (high confidence)
AZOLI_3003	Ancestral (high confidence)	AZOBR_180026	Unassigned
AZOLI_3004	Ancestral (high confidence)	AZOBR_180027	Ancestral (high confidence)
AZOLI_3005	Ancestral (medium confidence)	AZOBR_180028	Ancestral (medium confidence)
AZOLI_3006	Ancestral (medium confidence)	AZOBR_180029	Ancestral (low confidence)
AZOLI_3007	Ancestral (high confidence)	AZOBR_180030	Ancestral (high confidence)
AZOLI_3008	Ancestral (high confidence)	AZOBR_180031	Ancestral (medium confidence)
AZOLI_3010	Ancestral (high confidence)	AZOBR_180032	Ancestral (medium confidence)
AZOLI_3011	Horizontally Transferred (high confidence)	AZOBR_180033	Horizontally Transferred (high confidence)
AZOLI_3012	Horizontally Transferred (high confidence)	AZOBR_180034	Unassigned
AZOLI_3013	Unassigned	AZOBR_180035	Horizontally Transferred (high confidence)
AZOLI_3014	Ancestral (high confidence)	AZOBR_180036	Unassigned
AZOLI_3015	Ancestral (high confidence)	AZOBR_180037	Ancestral (low confidence)
AZOLI_3016	Ancestral (high confidence)	AZOBR_180038	Unassigned
AZOLI_3017	Ancestral (medium confidence)	AZOBR_180039	Ancestral (high confidence)
AZOLI_3018	Horizontally Transferred (high confidence)	AZOBR_180040	Horizontally Transferred (high confidence)
AZOLI_3019	Ancestral (high confidence)	AZOBR_180041	Horizontally Transferred (high confidence)
AZOLI_3020	Ancestral (high confidence)	AZOBR_180042	Unassigned
AZOLI_3021	Ancestral (high confidence)	AZOBR_180043	Horizontally Transferred (high confidence)
AZOLI_3022	Ancestral (high confidence)	AZOBR_180044	Horizontally Transferred (medium confidence)
AZOLI_3023	Ancestral (medium confidence)	AZOBR_180045	Ancestral (high confidence)
AZOLI_3024	Ancestral (medium confidence)	AZOBR_180046	Horizontally Transferred (medium confidence)
AZOLI_3025	Ancestral (high confidence)	AZOBR_180047	Horizontally Transferred (high confidence)
AZOLI_3026	Ancestral (medium confidence)	AZOBR_180048	Horizontally Transferred (high confidence)
AZOLI_3027	Ancestral (high confidence)	AZOBR_180049	Unassigned
AZOLI_3028	Ancestral (medium confidence)	AZOBR_180050	Horizontally Transferred (high confidence)
AZOLI_3029	Ancestral (high confidence)	AZOBR_180051	Unassigned

AZOLI_3030	Ancestral (high confidence)	AZOBR_180052	Ancestral (low confidence)
AZOLI_3031	Ancestral (high confidence)	AZOBR_180053	Unassigned
AZOLI_3032	Ancestral (high confidence)	AZOBR_180054	Horizontally Transferred (high confidence)
AZOLI_3033	Ancestral (high confidence)	AZOBR_180055	Horizontally Transferred (high confidence)
AZOLI_3035	Ancestral (high confidence)	AZOBR_180056	Ancestral (low confidence)
AZOLI_3036	Ancestral (high confidence)	AZOBR_180057	Unassigned
AZOLI_3037	Ancestral (medium confidence)	AZOBR_180058	Unassigned
AZOLI_3038	Ancestral (high confidence)	AZOBR_180059	Ancestral (medium confidence)
AZOLI_3039	Ancestral (medium confidence)	AZOBR_180060	Ancestral (medium confidence)
AZOLI_3040	Unassigned	AZOBR_180061	Ancestral (medium confidence)
AZOLI_3041	Unassigned	AZOBR_180062	Unassigned
AZOLI_3042	Ancestral (high confidence)	AZOBR_180063	Ancestral (high confidence)
AZOLI_3043	Ancestral (high confidence)	AZOBR_180064	Ancestral (high confidence)
AZOLI_3044	Ancestral (high confidence)	AZOBR_180065	Ancestral (high confidence)
AZOLI_3045	Ancestral (medium confidence)	AZOBR_180066	Ancestral (medium confidence)
AZOLI_3046	Ancestral (high confidence)	AZOBR_180067	Horizontally Transferred (high confidence)
AZOLI_3048	Ancestral (high confidence)	AZOBR_180068	Ancestral (low confidence)
AZOLI_3049	Unassigned	AZOBR_180069	Ancestral (high confidence)
AZOLI_3050	Ancestral (medium confidence)	AZOBR_180070	Ancestral (low confidence)
AZOLI_3051	Ancestral (medium confidence)	AZOBR_180071	Ancestral (high confidence)
AZOLI_3052	Ancestral (high confidence)	AZOBR_180072	Unassigned
AZOLI_3053	Ancestral (high confidence)	AZOBR_180073	Horizontally Transferred (high confidence)
AZOLI_3054	Ancestral (high confidence)	AZOBR_180074	Ancestral (low confidence)
AZOLI_3055	Ancestral (high confidence)	AZOBR_180075	Unassigned
AZOLI_3056	Ancestral (high confidence)	AZOBR_180076	Horizontally Transferred (medium confidence)
AZOLI_3057	Ancestral (medium confidence)	AZOBR_180077	Ancestral (low confidence)
AZOLI_3058	Ancestral (high confidence)	AZOBR_180078	Horizontally Transferred (high confidence)
AZOLI_3059	Ancestral (low confidence)	AZOBR_180079	Horizontally Transferred (high confidence)
AZOLI_3060	Ancestral (high confidence)	AZOBR_180080	Horizontally Transferred (high confidence)
AZOLI_3061	Ancestral (high confidence)	AZOBR_180081	Ancestral (high confidence)
AZOLI_3062	Ancestral (high confidence)	AZOBR_180082	Unassigned
AZOLI_3063	Ancestral (high confidence)	AZOBR_180083	Ancestral (low confidence)
AZOLI_3064	Horizontally Transferred (high confidence)	AZOBR_180084	Ancestral (low confidence)
AZOLI_3065	Horizontally Transferred (high confidence)	AZOBR_180085	Ancestral (medium confidence)
AZOLI_3066	Horizontally Transferred (high confidence)	AZOBR_180086	Ancestral (high confidence)
AZOLI_3067	Horizontally Transferred (high confidence)	AZOBR_180087	Ancestral (high confidence)
AZOLI_3068	Ancestral (medium confidence)	AZOBR_180088	Ancestral (high confidence)
AZOLI_3069	Unassigned	AZOBR_180089	Unassigned
AZOLI_3070	Ancestral (medium confidence)	AZOBR_180090	Ancestral (high confidence)
AZOLI_3071	Ancestral (high confidence)	AZOBR_180091	Ancestral (high confidence)
AZOLI_3072	Ancestral (high confidence)	AZOBR_180092	Ancestral (high confidence)
AZOLI_3073	Horizontally Transferred (medium confidence)	AZOBR_180093	Ancestral (high confidence)
AZOLI_3074	Ancestral (low confidence)	AZOBR_180094	Ancestral (high confidence)
AZOLI_3075	Ancestral (high confidence)	AZOBR_180095	Ancestral (medium confidence)
AZOLI_3076	Ancestral (high confidence)	AZOBR_180096	Ancestral (high confidence)
AZOLI_3077	Ancestral (high confidence)	AZOBR_180097	Ancestral (high confidence)
AZOLI_3078	Ancestral (high confidence)	AZOBR_180098	Ancestral (high confidence)
AZOLI_3079	Horizontally Transferred (high confidence)	AZOBR_180099	Ancestral (high confidence)
AZOLI_3080	Ancestral (high confidence)	AZOBR_180100	Horizontally Transferred (high confidence)
AZOLI_3081	Ancestral (high confidence)	AZOBR_180101	Ancestral (medium confidence)
AZOLI_3082	Ancestral (high confidence)	AZOBR_180102	Horizontally Transferred (high confidence)
AZOLI_3083	Ancestral (high confidence)	AZOBR_180103	Ancestral (high confidence)
AZOLI_3084	Ancestral (high confidence)	AZOBR_180104	Ancestral (medium confidence)
AZOLI_3085	Ancestral (high confidence)	AZOBR_180105	Ancestral (medium confidence)
AZOLI_3086	Ancestral (medium confidence)	AZOBR_180106	Ancestral (medium confidence)
AZOLI_3087	Ancestral (high confidence)	AZOBR_180107	Ancestral (high confidence)
AZOLI_3088	Ancestral (low confidence)	AZOBR_180108	Ancestral (medium confidence)
AZOLI_3089	Ancestral (high confidence)	AZOBR_180109	Unassigned
AZOLI_3090	Unassigned	AZOBR_180110	Ancestral (high confidence)
AZOLI_3091	Ancestral (high confidence)	AZOBR_180111	Ancestral (medium confidence)
AZOLI_3092	Horizontally Transferred (high confidence)	AZOBR_180112	Ancestral (low confidence)
AZOLI_3093	Ancestral (high confidence)	AZOBR_180113	Horizontally Transferred (high confidence)
AZOLI_3094	Ancestral (medium confidence)	AZOBR_180114	Ancestral (high confidence)
AZOLI_3095	Ancestral (medium confidence)	AZOBR_180115	Unassigned
AZOLI_3096	Horizontally Transferred (high confidence)	AZOBR_180116	Ancestral (high confidence)
AZOLI_3097	Unassigned	AZOBR_180117	Ancestral (high confidence)
AZOLI_3098	Ancestral (medium confidence)	AZOBR_180118	Ancestral (high confidence)
AZOLI_3099	Ancestral (medium confidence)	AZOBR_180119	Ancestral (high confidence)
AZOLI_3100	Ancestral (high confidence)	AZOBR_180120	Ancestral (high confidence)
AZOLI_3101	Ancestral (high confidence)	AZOBR_180121	Ancestral (high confidence)
AZOLI_3102	Horizontally Transferred (high confidence)	AZOBR_180122	Ancestral (medium confidence)
AZOLI_3103	Horizontally Transferred (high confidence)	AZOBR_180123	Ancestral (high confidence)
AZOLI_3104	Ancestral (medium confidence)	AZOBR_180124	Unassigned
AZOLI_3105	Ancestral (high confidence)	AZOBR_180125	Horizontally Transferred (high confidence)
AZOLI_3106	Ancestral (high confidence)	AZOBR_180126	Ancestral (high confidence)

AZOLI_3107	Ancestral (medium confidence)	AZOBR_180127	Ancestral (medium confidence)
AZOLI_3108	Ancestral (high confidence)	AZOBR_180128	Ancestral (low confidence)
AZOLI_3109	Ancestral (low confidence)	AZOBR_180129	Ancestral (high confidence)
AZOLI_3110	Ancestral (high confidence)	AZOBR_180130	Horizontally Transferred (high confidence)
AZOLI_3111	Ancestral (high confidence)	AZOBR_180131	Horizontally Transferred (high confidence)
AZOLI_3112	Horizontally Transferred (high confidence)	AZOBR_180132	Unassigned
AZOLI_3113	Ancestral (medium confidence)	AZOBR_180133	Horizontally Transferred (high confidence)
AZOLI_3114	Horizontally Transferred (high confidence)	AZOBR_180134	Ancestral (low confidence)
AZOLI_3115	Unassigned	AZOBR_180135	Ancestral (low confidence)
AZOLI_3117	Unassigned	AZOBR_180136	Unassigned
AZOLI_3118	Ancestral (low confidence)	AZOBR_180137	Ancestral (medium confidence)
AZOLI_3119	Ancestral (low confidence)	AZOBR_180138	Unassigned
AZOLI_3120	Ancestral (high confidence)	AZOBR_180139	Unassigned
AZOLI_3121	Ancestral (high confidence)	AZOBR_180140	Horizontally Transferred (high confidence)
AZOLI_3122	Ancestral (high confidence)	AZOBR_180141	Ancestral (high confidence)
AZOLI_3123	Ancestral (medium confidence)	AZOBR_180142	Ancestral (medium confidence)
AZOLI_3124	Ancestral (medium confidence)	AZOBR_180143	Unassigned
AZOLI_3125	Ancestral (medium confidence)	AZOBR_180144	Unassigned
AZOLI_3126	Ancestral (medium confidence)	AZOBR_180145	Ancestral (high confidence)
AZOLI_3127	Ancestral (medium confidence)	AZOBR_180146	Ancestral (low confidence)
AZOLI_3128	Ancestral (medium confidence)	AZOBR_180147	Unassigned
AZOLI_3129	Ancestral (medium confidence)	AZOBR_180148	Ancestral (low confidence)
AZOLI_3130	Unassigned	AZOBR_180149	Unassigned
AZOLI_3131	Horizontally Transferred (high confidence)	AZOBR_180150	Ancestral (low confidence)
AZOLI_3132	Unassigned	AZOBR_180151	Ancestral (medium confidence)
AZOLI_3133	Ancestral (high confidence)	AZOBR_180152	Horizontally Transferred (low confidence)
AZOLI_3134	Ancestral (medium confidence)	AZOBR_180153	Ancestral (low confidence)
AZOLI_3135	Ancestral (high confidence)	AZOBR_180154	Ancestral (low confidence)
AZOLI_3136	Unassigned	AZOBR_180155	Ancestral (high confidence)
AZOLI_3137	Ancestral (high confidence)	AZOBR_180156	Unassigned
AZOLI_3139	Ancestral (high confidence)	AZOBR_180157	Ancestral (high confidence)
AZOLI_3149	Horizontally Transferred (high confidence)	AZOBR_180158	Ancestral (medium confidence)
AZOLI_3150	Horizontally Transferred (high confidence)	AZOBR_180159	Unassigned
AZOLI_3151	Ancestral (medium confidence)	AZOBR_180160	Ancestral (medium confidence)
AZOLI_3152	Unassigned	AZOBR_180161	Ancestral (medium confidence)
AZOLI_3153	Ancestral (high confidence)	AZOBR_180162	Ancestral (low confidence)
AZOLI_3154	Ancestral (high confidence)	AZOBR_180163	Ancestral (medium confidence)
AZOLI_3155	Ancestral (high confidence)	AZOBR_180164	Ancestral (low confidence)
AZOLI_3156	Horizontally Transferred (high confidence)	AZOBR_180165	Ancestral (low confidence)
AZOLI_3157	Ancestral (medium confidence)	AZOBR_180166	Ancestral (medium confidence)
AZOLI_3158	Ancestral (high confidence)	AZOBR_180167	Unassigned
AZOLI_3159	Ancestral (high confidence)	AZOBR_180168	Unassigned
AZOLI_3160	Ancestral (high confidence)	AZOBR_180169	Horizontally Transferred (high confidence)
AZOLI_3161	Unassigned	AZOBR_180170	Ancestral (medium confidence)
AZOLI_3162	Unassigned	AZOBR_180171	Ancestral (medium confidence)
AZOLI_3163	Ancestral (high confidence)	AZOBR_180172	Ancestral (medium confidence)
AZOLI_3164	Ancestral (medium confidence)	AZOBR_180173	Ancestral (medium confidence)
AZOLI_3165	Ancestral (high confidence)	AZOBR_180174	Unassigned
AZOLI_3166	Ancestral (high confidence)	AZOBR_180175	Horizontally Transferred (high confidence)
AZOLI_3167	Ancestral (medium confidence)	AZOBR_180176	Horizontally Transferred (low confidence)
AZOLI_3168	Ancestral (high confidence)	AZOBR_180177	Horizontally Transferred (high confidence)
AZOLI_3169	Horizontally Transferred (high confidence)	AZOBR_180178	Ancestral (low confidence)
AZOLI_3170	Horizontally Transferred (high confidence)	AZOBR_180179	Ancestral (medium confidence)
AZOLI_3171	Horizontally Transferred (high confidence)	AZOBR_180180	Horizontally Transferred (high confidence)
AZOLI_3172	Horizontally Transferred (high confidence)	AZOBR_180181	Horizontally Transferred (medium confidence)
AZOLI_3173	Unassigned	AZOBR_180182	Ancestral (medium confidence)
AZOLI_3174	Ancestral (medium confidence)	AZOBR_180183	Ancestral (high confidence)
AZOLI_3175	Ancestral (low confidence)	AZOBR_180184	Ancestral (medium confidence)
AZOLI_3176	Ancestral (high confidence)	AZOBR_180185	Unassigned
AZOLI_3177	Ancestral (high confidence)	AZOBR_180186	Unassigned
AZOLI_3178	Ancestral (high confidence)	AZOBR_180187	Unassigned
AZOLI_p10003	Unassigned	AZOBR_180188	Unassigned
AZOLI_p10005	Horizontally Transferred (high confidence)	AZOBR_180189	Unassigned
AZOLI_p10010	Horizontally Transferred (high confidence)	AZOBR_180190	Unassigned
AZOLI_p10011	Horizontally Transferred (high confidence)	AZOBR_180191	Ancestral (high confidence)
AZOLI_p10012	Horizontally Transferred (high confidence)	AZOBR_180192	Ancestral (high confidence)
AZOLI_p10013	Ancestral (medium confidence)	AZOBR_180193	Ancestral (medium confidence)
AZOLI_p10015	Horizontally Transferred (high confidence)	AZOBR_180194	Unassigned
AZOLI_p10016	Horizontally Transferred (low confidence)	AZOBR_180195	Ancestral (high confidence)
AZOLI_p10017	Horizontally Transferred (medium confidence)	AZOBR_180196	Horizontally Transferred (high confidence)
AZOLI_p10018	Ancestral (medium confidence)	AZOBR_180197	Ancestral (medium confidence)
AZOLI_p10019	Ancestral (medium confidence)	AZOBR_180198	Unassigned
AZOLI_p10020	Horizontally Transferred (high confidence)	AZOBR_180199	Ancestral (high confidence)
AZOLI_p10021	Horizontally Transferred (medium confidence)	AZOBR_180200	Ancestral (medium confidence)
AZOLI_p10022	Ancestral (low confidence)	AZOBR_180201	Unassigned

AZOLI_p10023	Ancestral (low confidence)	AZOBR_180202	Horizontally Transferred (high confidence)
AZOLI_p10024	Ancestral (medium confidence)	AZOBR_180203	Ancestral (low confidence)
AZOLI_p10025	Ancestral (medium confidence)	AZOBR_180204	Ancestral (low confidence)
AZOLI_p10026	Ancestral (low confidence)	AZOBR_180205	Unassigned
AZOLI_p10027	Ancestral (high confidence)	AZOBR_180206	Unassigned
AZOLI_p10028	Ancestral (medium confidence)	AZOBR_180207	Horizontally Transferred (low confidence)
AZOLI_p10029	Ancestral (medium confidence)	AZOBR_180208	Horizontally Transferred (high confidence)
AZOLI_p10030	Ancestral (medium confidence)	AZOBR_180209	Unassigned
AZOLI_p10031	Horizontally Transferred (low confidence)	AZOBR_180210	Horizontally Transferred (high confidence)
AZOLI_p10032	Ancestral (medium confidence)	AZOBR_180211	Ancestral (low confidence)
AZOLI_p10033	Ancestral (medium confidence)	AZOBR_180212	Unassigned
AZOLI_p10035	Horizontally Transferred (high confidence)	AZOBR_180213	Horizontally Transferred (high confidence)
AZOLI_p10036	Ancestral (medium confidence)	AZOBR_180214	Ancestral (low confidence)
AZOLI_p10037	Horizontally Transferred (medium confidence)	AZOBR_180215	Ancestral (low confidence)
AZOLI_p10038	Unassigned	AZOBR_180216	Horizontally Transferred (high confidence)
AZOLI_p10039	Ancestral (low confidence)	AZOBR_180217	Horizontally Transferred (medium confidence)
AZOLI_p10040	Ancestral (high confidence)	AZOBR_180218	Unassigned
AZOLI_p10041	Unassigned	AZOBR_180219	Horizontally Transferred (medium confidence)
AZOLI_p10042	Horizontally Transferred (medium confidence)	AZOBR_180220	Horizontally Transferred (medium confidence)
AZOLI_p10043	Horizontally Transferred (medium confidence)	AZOBR_180221	Horizontally Transferred (high confidence)
AZOLI_p10044	Unassigned	AZOBR_180222	Ancestral (low confidence)
AZOLI_p10045	Ancestral (high confidence)	AZOBR_180223	Ancestral (low confidence)
AZOLI_p10046	Ancestral (low confidence)	AZOBR_180224	Ancestral (low confidence)
AZOLI_p10047	Ancestral (low confidence)	AZOBR_180225	Horizontally Transferred (high confidence)
AZOLI_p10049	Horizontally Transferred (medium confidence)	AZOBR_180226	Ancestral (low confidence)
AZOLI_p10050	Horizontally Transferred (high confidence)	AZOBR_180227	Horizontally Transferred (high confidence)
AZOLI_p10051	Unassigned	AZOBR_180228	Ancestral (low confidence)
AZOLI_p10052	Ancestral (low confidence)	AZOBR_180229	Unassigned
AZOLI_p10053	Horizontally Transferred (low confidence)	AZOBR_180230	Ancestral (high confidence)
AZOLI_p10054	Horizontally Transferred (medium confidence)	AZOBR_180231	Horizontally Transferred (high confidence)
AZOLI_p10055	Horizontally Transferred (high confidence)	AZOBR_180232	Ancestral (medium confidence)
AZOLI_p10056	Horizontally Transferred (high confidence)	AZOBR_180233	Horizontally Transferred (high confidence)
AZOLI_p10057	Ancestral (medium confidence)	AZOBR_180234	Unassigned
AZOLI_p10058	Ancestral (low confidence)	AZOBR_180235	Unassigned
AZOLI_p10061	Horizontally Transferred (high confidence)	AZOBR_180236	Horizontally Transferred (high confidence)
AZOLI_p10064	Unassigned	AZOBR_180237	Horizontally Transferred (high confidence)
AZOLI_p10065	Horizontally Transferred (high confidence)	AZOBR_180238	Unassigned
AZOLI_p10066	Horizontally Transferred (high confidence)	AZOBR_180239	Unassigned
AZOLI_p10067	Ancestral (low confidence)	AZOBR_180240	Ancestral (medium confidence)
AZOLI_p10068	Horizontally Transferred (low confidence)	AZOBR_180241	Ancestral (medium confidence)
AZOLI_p10069	Ancestral (low confidence)	AZOBR_180242	Ancestral (high confidence)
AZOLI_p10070	Unassigned	AZOBR_180243	Horizontally Transferred (high confidence)
AZOLI_p10071	Ancestral (low confidence)	AZOBR_180244	Ancestral (medium confidence)
AZOLI_p10072	Horizontally Transferred (medium confidence)	AZOBR_180245	Unassigned
AZOLI_p10073	Unassigned	AZOBR_180246	Horizontally Transferred (high confidence)
AZOLI_p10074	Horizontally Transferred (high confidence)	AZOBR_180247	Ancestral (medium confidence)
AZOLI_p10075	Ancestral (low confidence)	AZOBR_180248	Unassigned
AZOLI_p10076	Ancestral (low confidence)	AZOBR_180249	Unassigned
AZOLI_p10077	Ancestral (low confidence)	AZOBR_180250	Ancestral (high confidence)
AZOLI_p10078	Unassigned	AZOBR_180251	Ancestral (high confidence)
AZOLI_p10079	Horizontally Transferred (high confidence)	AZOBR_180252	Horizontally Transferred (high confidence)
AZOLI_p10080	Unassigned	AZOBR_180253	Horizontally Transferred (high confidence)
AZOLI_p10081	Horizontally Transferred (high confidence)	AZOBR_180254	Ancestral (high confidence)
AZOLI_p10082	Ancestral (low confidence)	AZOBR_180255	Ancestral (medium confidence)
AZOLI_p10083	Horizontally Transferred (high confidence)	AZOBR_180256	Ancestral (medium confidence)
AZOLI_p10084	Ancestral (low confidence)	AZOBR_180257	Ancestral (low confidence)
AZOLI_p10085	Ancestral (low confidence)	AZOBR_180258	Ancestral (medium confidence)
AZOLI_p10086	Horizontally Transferred (high confidence)	AZOBR_180259	Horizontally Transferred (high confidence)
AZOLI_p10087	Ancestral (low confidence)	AZOBR_180260	Ancestral (low confidence)
AZOLI_p10088	Ancestral (low confidence)	AZOBR_180261	Ancestral (medium confidence)
AZOLI_p10089	Unassigned	AZOBR_180262	Ancestral (low confidence)
AZOLI_p10091	Horizontally Transferred (high confidence)	AZOBR_180263	Ancestral (high confidence)
AZOLI_p10092	Ancestral (medium confidence)	AZOBR_180264	Ancestral (low confidence)
AZOLI_p10093	Ancestral (high confidence)	AZOBR_180265	Ancestral (medium confidence)
AZOLI_p10094	Ancestral (low confidence)	AZOBR_180266	Ancestral (low confidence)
AZOLI_p10095	Ancestral (high confidence)	AZOBR_180267	Ancestral (medium confidence)
AZOLI_p10096	Ancestral (high confidence)	AZOBR_180268	Unassigned
AZOLI_p10097	Ancestral (medium confidence)	AZOBR_180269	Horizontally Transferred (high confidence)
AZOLI_p10098	Unassigned	AZOBR_180270	Unassigned
AZOLI_p10099	Horizontally Transferred (high confidence)	AZOBR_180271	Ancestral (low confidence)
AZOLI_p10100	Ancestral (medium confidence)	AZOBR_180272	Ancestral (low confidence)
AZOLI_p10101	Ancestral (medium confidence)	AZOBR_180273	Ancestral (low confidence)
AZOLI_p10102	Ancestral (medium confidence)	AZOBR_180274	Ancestral (low confidence)
AZOLI_p10103	Horizontally Transferred (high confidence)	AZOBR_180275	Ancestral (low confidence)
AZOLI_p10105	Ancestral (low confidence)	AZOBR_180276	Ancestral (low confidence)

AZOLI_p10106	Ancestral (high confidence)	AZOBR_180277	Ancestral (low confidence)
AZOLI_p10107	Ancestral (medium confidence)	AZOBR_180278	Ancestral (low confidence)
AZOLI_p10108	Ancestral (low confidence)	AZOBR_180279	Ancestral (low confidence)
AZOLI_p10109	Horizontally Transferred (low confidence)	AZOBR_180280	Ancestral (low confidence)
AZOLI_p10111	Ancestral (medium confidence)	AZOBR_180281	Ancestral (low confidence)
AZOLI_p10112	Unassigned	AZOBR_180282	Ancestral (medium confidence)
AZOLI_p10113	Unassigned	AZOBR_180283	Unassigned
AZOLI_p10114	Horizontally Transferred (high confidence)	AZOBR_180284	Horizontally Transferred (high confidence)
AZOLI_p10115	Unassigned	AZOBR_180285	Horizontally Transferred (high confidence)
AZOLI_p10116	Unassigned	AZOBR_180286	Horizontally Transferred (high confidence)
AZOLI_p10118	Unassigned	AZOBR_180287	Horizontally Transferred (high confidence)
AZOLI_p10122	Unassigned	AZOBR_180288	Horizontally Transferred (high confidence)
AZOLI_p10123	Horizontally Transferred (high confidence)	AZOBR_180289	Horizontally Transferred (high confidence)
AZOLI_p10124	Horizontally Transferred (high confidence)	AZOBR_180290	Horizontally Transferred (high confidence)
AZOLI_p10125	Ancestral (medium confidence)	AZOBR_180291	Horizontally Transferred (high confidence)
AZOLI_p10126	Unassigned	AZOBR_180292	Horizontally Transferred (high confidence)
AZOLI_p10128	Unassigned	AZOBR_180293	Horizontally Transferred (high confidence)
AZOLI_p10129	Horizontally Transferred (high confidence)	AZOBR_180294	Horizontally Transferred (low confidence)
AZOLI_p10130	Horizontally Transferred (high confidence)	AZOBR_180295	Horizontally Transferred (high confidence)
AZOLI_p10131	Ancestral (medium confidence)	AZOBR_180296	Horizontally Transferred (high confidence)
AZOLI_p10132	Horizontally Transferred (high confidence)	AZOBR_180297	Horizontally Transferred (high confidence)
AZOLI_p10133	Ancestral (low confidence)	AZOBR_180298	Horizontally Transferred (low confidence)
AZOLI_p10134	Unassigned	AZOBR_180299	Horizontally Transferred (medium confidence)
AZOLI_p10139	Horizontally Transferred (medium confidence)	AZOBR_180300	Horizontally Transferred (high confidence)
AZOLI_p10140	Unassigned	AZOBR_180301	Horizontally Transferred (high confidence)
AZOLI_p10142	Unassigned	AZOBR_180302	Horizontally Transferred (medium confidence)
AZOLI_p10144	Ancestral (high confidence)	AZOBR_180303	Horizontally Transferred (high confidence)
AZOLI_p10145	Unassigned	AZOBR_180304	Horizontally Transferred (high confidence)
AZOLI_p10147	Horizontally Transferred (high confidence)	AZOBR_180305	Horizontally Transferred (high confidence)
AZOLI_p10148	Horizontally Transferred (medium confidence)	AZOBR_180306	Horizontally Transferred (medium confidence)
AZOLI_p10149	Horizontally Transferred (high confidence)	AZOBR_180307	Horizontally Transferred (high confidence)
AZOLI_p10150	Unassigned	AZOBR_180308	Horizontally Transferred (medium confidence)
AZOLI_p10151	Unassigned	AZOBR_180309	Horizontally Transferred (high confidence)
AZOLI_p10152	Horizontally Transferred (high confidence)	AZOBR_180310	Horizontally Transferred (high confidence)
AZOLI_p10153	Horizontally Transferred (high confidence)	AZOBR_180311	Horizontally Transferred (high confidence)
AZOLI_p10154	Ancestral (low confidence)	AZOBR_180312	Ancestral (low confidence)
AZOLI_p10155	Horizontally Transferred (high confidence)	AZOBR_180313	Unassigned
AZOLI_p10156	Horizontally Transferred (high confidence)	AZOBR_180314	Horizontally Transferred (high confidence)
AZOLI_p10157	Horizontally Transferred (high confidence)	AZOBR_180315	Horizontally Transferred (high confidence)
AZOLI_p10158	Horizontally Transferred (high confidence)	AZOBR_180316	Horizontally Transferred (high confidence)
AZOLI_p10161	Ancestral (medium confidence)	AZOBR_180317	Horizontally Transferred (high confidence)
AZOLI_p10162	Unassigned	AZOBR_180318	Horizontally Transferred (high confidence)
AZOLI_p10164	Ancestral (low confidence)	AZOBR_180319	Horizontally Transferred (high confidence)
AZOLI_p10165	Ancestral (low confidence)	AZOBR_180320	Horizontally Transferred (high confidence)
AZOLI_p10166	Horizontally Transferred (medium confidence)	AZOBR_180321	Unassigned
AZOLI_p10167	Ancestral (low confidence)	AZOBR_180322	Unassigned
AZOLI_p10168	Ancestral (low confidence)	AZOBR_180323	Unassigned
AZOLI_p10169	Horizontally Transferred (medium confidence)	AZOBR_180324	Unassigned
AZOLI_p10170	Unassigned	AZOBR_180325	Unassigned
AZOLI_p10171	Horizontally Transferred (high confidence)	AZOBR_190001	Ancestral (low confidence)
AZOLI_p10172	Horizontally Transferred (high confidence)	AZOBR_190002	Unassigned
AZOLI_p10173	Ancestral (low confidence)	AZOBR_190003	Unassigned
AZOLI_p10174	Ancestral (low confidence)	AZOBR_190004	Horizontally Transferred (high confidence)
AZOLI_p10177	Ancestral (medium confidence)	AZOBR_190005	Ancestral (high confidence)
AZOLI_p10178	Ancestral (low confidence)	AZOBR_190006	Horizontally Transferred (high confidence)
AZOLI_p10180	Horizontally Transferred (high confidence)	AZOBR_190007	Horizontally Transferred (high confidence)
AZOLI_p10184	Unassigned	AZOBR_190008	Horizontally Transferred (high confidence)
AZOLI_p10185	Ancestral (medium confidence)	AZOBR_190009	Horizontally Transferred (high confidence)
AZOLI_p10186	Ancestral (medium confidence)	AZOBR_190010	Horizontally Transferred (high confidence)
AZOLI_p10187	Ancestral (medium confidence)	AZOBR_190011	Unassigned
AZOLI_p10188	Ancestral (low confidence)	AZOBR_190012	Unassigned
AZOLI_p10189	Ancestral (medium confidence)	AZOBR_190013	Unassigned
AZOLI_p10190	Unassigned	AZOBR_190014	Horizontally Transferred (high confidence)
AZOLI_p10191	Horizontally Transferred (high confidence)	AZOBR_190015	Ancestral (low confidence)
AZOLI_p10193	Unassigned	AZOBR_190016	Horizontally Transferred (high confidence)
AZOLI_p10194	Horizontally Transferred (medium confidence)	AZOBR_190017	Unassigned
AZOLI_p10195	Ancestral (medium confidence)	AZOBR_190018	Horizontally Transferred (medium confidence)
AZOLI_p10196	Horizontally Transferred (high confidence)	AZOBR_190019	Horizontally Transferred (high confidence)
AZOLI_p10197	Horizontally Transferred (medium confidence)	AZOBR_190020	Horizontally Transferred (high confidence)
AZOLI_p10198	Horizontally Transferred (medium confidence)	AZOBR_190021	Horizontally Transferred (high confidence)
AZOLI_p10199	Ancestral (high confidence)	AZOBR_190022	Horizontally Transferred (medium confidence)
AZOLI_p10200	Horizontally Transferred (high confidence)	AZOBR_190023	Horizontally Transferred (medium confidence)
AZOLI_p10202	Ancestral (low confidence)	AZOBR_190024	Unassigned
AZOLI_p10203	Ancestral (medium confidence)	AZOBR_190025	Ancestral (low confidence)
AZOLI_p10204	Horizontally Transferred (high confidence)	AZOBR_190026	Horizontally Transferred (high confidence)

AZOLI_p10205	Horizontally Transferred (high confidence)	AZOBR_190027	Ancestral (low confidence)
AZOLI_p10206	Horizontally Transferred (low confidence)	AZOBR_190028	Ancestral (low confidence)
AZOLI_p10207	Horizontally Transferred (high confidence)	AZOBR_190029	Horizontally Transferred (medium confidence)
AZOLI_p10208	Horizontally Transferred (high confidence)	AZOBR_190030	Horizontally Transferred (low confidence)
AZOLI_p10209	Horizontally Transferred (high confidence)	AZOBR_190031	Ancestral (medium confidence)
AZOLI_p10210	Horizontally Transferred (high confidence)	AZOBR_190032	Ancestral (low confidence)
AZOLI_p10211	Horizontally Transferred (high confidence)	AZOBR_190033	Ancestral (low confidence)
AZOLI_p10212	Horizontally Transferred (high confidence)	AZOBR_190034	Unassigned
AZOLI_p10213	Ancestral (medium confidence)	AZOBR_190035	Horizontally Transferred (high confidence)
AZOLI_p10214	Ancestral (medium confidence)	AZOBR_190036	Unassigned
AZOLI_p10215	Horizontally Transferred (low confidence)	AZOBR_190037	Unassigned
AZOLI_p10216	Ancestral (low confidence)	AZOBR_190038	Unassigned
AZOLI_p10217	Horizontally Transferred (high confidence)	AZOBR_190039	Ancestral (low confidence)
AZOLI_p10219	Ancestral (low confidence)	AZOBR_190040	Ancestral (low confidence)
AZOLI_p10220	Ancestral (low confidence)	AZOBR_200001	Unassigned
AZOLI_p10221	Horizontally Transferred (high confidence)	AZOBR_200002	Horizontally Transferred (high confidence)
AZOLI_p10222	Ancestral (low confidence)	AZOBR_200003	Unassigned
AZOLI_p10223	Ancestral (medium confidence)	AZOBR_200004	Horizontally Transferred (high confidence)
AZOLI_p10224	Ancestral (low confidence)	AZOBR_200005	Horizontally Transferred (medium confidence)
AZOLI_p10225	Ancestral (low confidence)	AZOBR_200006	Horizontally Transferred (high confidence)
AZOLI_p10226	Horizontally Transferred (medium confidence)	AZOBR_200007	Horizontally Transferred (medium confidence)
AZOLI_p10227	Ancestral (low confidence)	AZOBR_200008	Unassigned
AZOLI_p10228	Ancestral (low confidence)	AZOBR_200009	Horizontally Transferred (medium confidence)
AZOLI_p10229	Horizontally Transferred (medium confidence)	AZOBR_200010	Unassigned
AZOLI_p10230	Horizontally Transferred (high confidence)	AZOBR_200011	Unassigned
AZOLI_p10231	Horizontally Transferred (medium confidence)	AZOBR_200012	Unassigned
AZOLI_p10232	Ancestral (low confidence)	AZOBR_200013	Horizontally Transferred (high confidence)
AZOLI_p10233	Horizontally Transferred (high confidence)	AZOBR_200014	Horizontally Transferred (high confidence)
AZOLI_p10234	Horizontally Transferred (high confidence)	AZOBR_200015	Horizontally Transferred (medium confidence)
AZOLI_p10235	Unassigned	AZOBR_200016	Horizontally Transferred (high confidence)
AZOLI_p10236	Ancestral (medium confidence)	AZOBR_200017	Ancestral (low confidence)
AZOLI_p10237	Ancestral (low confidence)	AZOBR_200018	Unassigned
AZOLI_p10238	Unassigned	AZOBR_200019	Unassigned
AZOLI_p10239	Unassigned	AZOBR_200020	Ancestral (low confidence)
AZOLI_p10240	Ancestral (low confidence)	AZOBR_200021	Unassigned
AZOLI_p10241	Ancestral (low confidence)	AZOBR_200022	Ancestral (low confidence)
AZOLI_p10242	Horizontally Transferred (medium confidence)	AZOBR_200023	Ancestral (low confidence)
AZOLI_p10243	Horizontally Transferred (high confidence)	AZOBR_200024	Ancestral (low confidence)
AZOLI_p10244	Horizontally Transferred (high confidence)	AZOBR_200025	Unassigned
AZOLI_p10245	Horizontally Transferred (high confidence)	AZOBR_200026	Ancestral (high confidence)
AZOLI_p10246	Horizontally Transferred (medium confidence)	AZOBR_200027	Ancestral (high confidence)
AZOLI_p10247	Horizontally Transferred (high confidence)	AZOBR_200028	Ancestral (high confidence)
AZOLI_p10248	Horizontally Transferred (high confidence)	AZOBR_200029	Ancestral (medium confidence)
AZOLI_p10250	Ancestral (high confidence)	AZOBR_200030	Horizontally Transferred (high confidence)
AZOLI_p10251	Ancestral (high confidence)	AZOBR_200031	Horizontally Transferred (high confidence)
AZOLI_p10252	Horizontally Transferred (high confidence)	AZOBR_200032	Horizontally Transferred (medium confidence)
AZOLI_p10253	Horizontally Transferred (high confidence)	AZOBR_200033	Unassigned
AZOLI_p10255	Unassigned	AZOBR_200034	Ancestral (medium confidence)
AZOLI_p10256	Horizontally Transferred (high confidence)	AZOBR_200035	Ancestral (medium confidence)
AZOLI_p10257	Horizontally Transferred (high confidence)	AZOBR_200036	Unassigned
AZOLI_p10258	Ancestral (high confidence)	AZOBR_200037	Ancestral (medium confidence)
AZOLI_p10259	Ancestral (high confidence)	AZOBR_200038	Ancestral (high confidence)
AZOLI_p10260	Ancestral (high confidence)	AZOBR_200039	Ancestral (medium confidence)
AZOLI_p10261	Horizontally Transferred (high confidence)	AZOBR_200040	Unassigned
AZOLI_p10262	Ancestral (medium confidence)	AZOBR_200041	Ancestral (medium confidence)
AZOLI_p10263	Unassigned	AZOBR_200042	Ancestral (medium confidence)
AZOLI_p10265	Horizontally Transferred (high confidence)	AZOBR_200043	Ancestral (medium confidence)
AZOLI_p10266	Unassigned	AZOBR_200044	Ancestral (medium confidence)
AZOLI_p10267	Unassigned	AZOBR_200045	Ancestral (medium confidence)
AZOLI_p10268	Ancestral (high confidence)	AZOBR_200046	Ancestral (medium confidence)
AZOLI_p10269	Ancestral (medium confidence)	AZOBR_200047	Ancestral (medium confidence)
AZOLI_p10270	Ancestral (high confidence)	AZOBR_200048	Ancestral (medium confidence)
AZOLI_p10271	Horizontally Transferred (medium confidence)	AZOBR_200049	Ancestral (high confidence)
AZOLI_p10272	Ancestral (high confidence)	AZOBR_200050	Ancestral (medium confidence)
AZOLI_p10273	Horizontally Transferred (medium confidence)	AZOBR_200051	Unassigned
AZOLI_p10274	Ancestral (high confidence)	AZOBR_200052	Ancestral (low confidence)
AZOLI_p10276	Ancestral (high confidence)	AZOBR_200053	Unassigned
AZOLI_p10277	Ancestral (low confidence)	AZOBR_200054	Horizontally Transferred (high confidence)
AZOLI_p10278	Unassigned	AZOBR_200055	Horizontally Transferred (high confidence)
AZOLI_p10279	Ancestral (low confidence)	AZOBR_200056	Unassigned
AZOLI_p10280	Horizontally Transferred (medium confidence)	AZOBR_200057	Unassigned
AZOLI_p10281	Horizontally Transferred (high confidence)	AZOBR_200058	Unassigned
AZOLI_p10282	Horizontally Transferred (high confidence)	AZOBR_200059	Horizontally Transferred (high confidence)
AZOLI_p10283	Horizontally Transferred (high confidence)	AZOBR_200060	Ancestral (low confidence)
AZOLI_p10285	Horizontally Transferred (high confidence)	AZOBR_200061	Unassigned

AZOLI_p10286	Horizontally Transferred (high confidence)	AZOBR_200062	Horizontally Transferred (high confidence)
AZOLI_p10287	Horizontally Transferred (high confidence)	AZOBR_200063	Horizontally Transferred (high confidence)
AZOLI_p10289	Unassigned	AZOBR_200064	Unassigned
AZOLI_p10290	Horizontally Transferred (high confidence)	AZOBR_200065	Horizontally Transferred (high confidence)
AZOLI_p10291	Ancestral (high confidence)	AZOBR_200066	Horizontally Transferred (high confidence)
AZOLI_p10292	Horizontally Transferred (low confidence)	AZOBR_200067	Horizontally Transferred (high confidence)
AZOLI_p10293	Ancestral (low confidence)	AZOBR_200068	Unassigned
AZOLI_p10294	Ancestral (medium confidence)	AZOBR_200069	Horizontally Transferred (high confidence)
AZOLI_p10295	Unassigned	AZOBR_200070	Horizontally Transferred (high confidence)
AZOLI_p10297	Horizontally Transferred (high confidence)	AZOBR_200071	Unassigned
AZOLI_p10298	Horizontally Transferred (high confidence)	AZOBR_200072	Unassigned
AZOLI_p10299	Horizontally Transferred (high confidence)	AZOBR_200073	Unassigned
AZOLI_p10300	Ancestral (low confidence)	AZOBR_200074	Horizontally Transferred (high confidence)
AZOLI_p10301	Horizontally Transferred (high confidence)	AZOBR_200075	Horizontally Transferred (high confidence)
AZOLI_p10302	Unassigned	AZOBR_200076	Unassigned
AZOLI_p10303	Ancestral (low confidence)	AZOBR_200077	Unassigned
AZOLI_p10304	Ancestral (high confidence)	AZOBR_200078	Horizontally Transferred (high confidence)
AZOLI_p10305	Ancestral (medium confidence)	AZOBR_200079	Unassigned
AZOLI_p10306	Ancestral (low confidence)	AZOBR_200080	Horizontally Transferred (high confidence)
AZOLI_p10307	Horizontally Transferred (medium confidence)	AZOBR_200081	Horizontally Transferred (high confidence)
AZOLI_p10309	Horizontally Transferred (high confidence)	AZOBR_200082	Unassigned
AZOLI_p10310	Horizontally Transferred (high confidence)	AZOBR_200083	Ancestral (medium confidence)
AZOLI_p10311	Ancestral (medium confidence)	AZOBR_200084	Unassigned
AZOLI_p10313	Horizontally Transferred (high confidence)	AZOBR_200085	Unassigned
AZOLI_p10316	Horizontally Transferred (high confidence)	AZOBR_200086	Horizontally Transferred (high confidence)
AZOLI_p10317	Horizontally Transferred (high confidence)	AZOBR_200087	Unassigned
AZOLI_p10318	Horizontally Transferred (high confidence)	AZOBR_200088	Unassigned
AZOLI_p10319	Horizontally Transferred (high confidence)	AZOBR_200089	Unassigned
AZOLI_p10320	Ancestral (high confidence)	AZOBR_200090	Unassigned
AZOLI_p10321	Ancestral (high confidence)	AZOBR_200091	Unassigned
AZOLI_p10322	Horizontally Transferred (high confidence)	AZOBR_200092	Unassigned
AZOLI_p10324	Ancestral (medium confidence)	AZOBR_200093	Unassigned
AZOLI_p10325	Ancestral (medium confidence)	AZOBR_200094	Horizontally Transferred (high confidence)
AZOLI_p10326	Ancestral (high confidence)	AZOBR_200095	Unassigned
AZOLI_p10327	Ancestral (high confidence)	AZOBR_200096	Unassigned
AZOLI_p10331	Ancestral (medium confidence)	AZOBR_200097	Unassigned
AZOLI_p10332	Ancestral (medium confidence)	AZOBR_200098	Unassigned
AZOLI_p10333	Ancestral (low confidence)	AZOBR_200099	Unassigned
AZOLI_p10335	Ancestral (high confidence)	AZOBR_200100	Unassigned
AZOLI_p10337	Horizontally Transferred (medium confidence)	AZOBR_200101	Unassigned
AZOLI_p10338	Ancestral (high confidence)	AZOBR_200102	Unassigned
AZOLI_p10339	Ancestral (low confidence)	AZOBR_200103	Unassigned
AZOLI_p10340	Horizontally Transferred (high confidence)	AZOBR_200104	Unassigned
AZOLI_p10342	Horizontally Transferred (medium confidence)	AZOBR_200105	Horizontally Transferred (high confidence)
AZOLI_p10343	Horizontally Transferred (medium confidence)	AZOBR_200106	Unassigned
AZOLI_p10344	Ancestral (low confidence)	AZOBR_200107	Unassigned
AZOLI_p10345	Horizontally Transferred (high confidence)	AZOBR_200108	Unassigned
AZOLI_p10346	Horizontally Transferred (high confidence)	AZOBR_200109	Unassigned
AZOLI_p10347	Ancestral (high confidence)	AZOBR_200110	Ancestral (low confidence)
AZOLI_p10348	Horizontally Transferred (high confidence)	AZOBR_200111	Horizontally Transferred (high confidence)
AZOLI_p10349	Ancestral (medium confidence)	AZOBR_200112	Horizontally Transferred (high confidence)
AZOLI_p10350	Ancestral (high confidence)	AZOBR_200113	Unassigned
AZOLI_p10351	Ancestral (medium confidence)	AZOBR_200114	Unassigned
AZOLI_p10352	Ancestral (high confidence)	AZOBR_200115	Unassigned
AZOLI_p10353	Horizontally Transferred (high confidence)	AZOBR_200116	Unassigned
AZOLI_p10354	Ancestral (medium confidence)	AZOBR_200117	Unassigned
AZOLI_p10355	Ancestral (medium confidence)	AZOBR_200118	Ancestral (low confidence)
AZOLI_p10356	Horizontally Transferred (high confidence)	AZOBR_200119	Ancestral (medium confidence)
AZOLI_p10357	Ancestral (medium confidence)	AZOBR_200120	Unassigned
AZOLI_p10358	Horizontally Transferred (high confidence)	AZOBR_200121	Ancestral (low confidence)
AZOLI_p10359	Horizontally Transferred (high confidence)	AZOBR_200122	Ancestral (high confidence)
AZOLI_p10360	Horizontally Transferred (high confidence)	AZOBR_200123	Ancestral (medium confidence)
AZOLI_p10361	Horizontally Transferred (high confidence)	AZOBR_200124	Ancestral (high confidence)
AZOLI_p10363	Horizontally Transferred (high confidence)	AZOBR_200125	Ancestral (medium confidence)
AZOLI_p10364	Horizontally Transferred (high confidence)	AZOBR_200126	Ancestral (medium confidence)
AZOLI_p10365	Ancestral (low confidence)	AZOBR_200127	Ancestral (medium confidence)
AZOLI_p10366	Ancestral (high confidence)	AZOBR_200128	Ancestral (high confidence)
AZOLI_p10367	Ancestral (high confidence)	AZOBR_200129	Horizontally Transferred (high confidence)
AZOLI_p10368	Ancestral (high confidence)	AZOBR_200130	Ancestral (medium confidence)
AZOLI_p10369	Ancestral (high confidence)	AZOBR_200131	Ancestral (medium confidence)
AZOLI_p10370	Horizontally Transferred (low confidence)	AZOBR_200132	Horizontally Transferred (high confidence)
AZOLI_p10371	Horizontally Transferred (high confidence)	AZOBR_200133	Ancestral (medium confidence)
AZOLI_p10372	Horizontally Transferred (high confidence)	AZOBR_200134	Ancestral (medium confidence)
AZOLI_p10373	Ancestral (low confidence)	AZOBR_200135	Ancestral (low confidence)
AZOLI_p10374	Ancestral (medium confidence)	AZOBR_200136	Ancestral (low confidence)

AZOLI_p10375	Ancestral (medium confidence)	AZOBR_200137	Ancestral (high confidence)
AZOLI_p10376	Unassigned	AZOBR_200138	Ancestral (medium confidence)
AZOLI_p10379	Horizontally Transferred (high confidence)	AZOBR_200139	Ancestral (high confidence)
AZOLI_p10380	Horizontally Transferred (high confidence)	AZOBR_200140	Ancestral (low confidence)
AZOLI_p10381	Horizontally Transferred (high confidence)	AZOBR_200141	Ancestral (low confidence)
AZOLI_p10382	Horizontally Transferred (high confidence)	AZOBR_200142	Horizontally Transferred (medium confidence)
AZOLI_p10383	Horizontally Transferred (high confidence)	AZOBR_200143	Ancestral (high confidence)
AZOLI_p10384	Horizontally Transferred (high confidence)	AZOBR_200144	Ancestral (medium confidence)
AZOLI_p10385	Horizontally Transferred (high confidence)	AZOBR_200145	Ancestral (medium confidence)
AZOLI_p10386	Horizontally Transferred (high confidence)	AZOBR_200146	Horizontally Transferred (high confidence)
AZOLI_p10387	Horizontally Transferred (high confidence)	AZOBR_200147	Unassigned
AZOLI_p10388	Horizontally Transferred (high confidence)	AZOBR_200148	Ancestral (low confidence)
AZOLI_p10389	Unassigned	AZOBR_200149	Ancestral (low confidence)
AZOLI_p10390	Ancestral (low confidence)	AZOBR_200150	Unassigned
AZOLI_p10391	Horizontally Transferred (high confidence)	AZOBR_200151	Ancestral (high confidence)
AZOLI_p10392	Horizontally Transferred (high confidence)	AZOBR_200152	Ancestral (high confidence)
AZOLI_p10393	Horizontally Transferred (high confidence)	AZOBR_200153	Ancestral (high confidence)
AZOLI_p10394	Unassigned	AZOBR_200154	Ancestral (medium confidence)
AZOLI_p10395	Horizontally Transferred (high confidence)	AZOBR_200155	Ancestral (medium confidence)
AZOLI_p10397	Horizontally Transferred (high confidence)	AZOBR_200156	Horizontally Transferred (high confidence)
AZOLI_p10398	Horizontally Transferred (high confidence)	AZOBR_200157	Ancestral (low confidence)
AZOLI_p10399	Horizontally Transferred (high confidence)	AZOBR_200158	Ancestral (high confidence)
AZOLI_p10400	Horizontally Transferred (high confidence)	AZOBR_200159	Unassigned
AZOLI_p10401	Horizontally Transferred (high confidence)	AZOBR_200160	Ancestral (high confidence)
AZOLI_p10403	Horizontally Transferred (high confidence)	AZOBR_200161	Horizontally Transferred (high confidence)
AZOLI_p10404	Horizontally Transferred (high confidence)	AZOBR_200162	Ancestral (medium confidence)
AZOLI_p10405	Horizontally Transferred (medium confidence)	AZOBR_200163	Ancestral (high confidence)
AZOLI_p10406	Unassigned	AZOBR_200164	Ancestral (high confidence)
AZOLI_p10407	Unassigned	AZOBR_200165	Unassigned
AZOLI_p10410	Unassigned	AZOBR_200166	Ancestral (high confidence)
AZOLI_p10413	Ancestral (high confidence)	AZOBR_200167	Ancestral (low confidence)
AZOLI_p10414	Ancestral (high confidence)	AZOBR_200168	Ancestral (high confidence)
AZOLI_p10415	Ancestral (low confidence)	AZOBR_200169	Unassigned
AZOLI_p10417	Unassigned	AZOBR_200170	Horizontally Transferred (medium confidence)
AZOLI_p10419	Unassigned	AZOBR_200171	Unassigned
AZOLI_p10421	Unassigned	AZOBR_200172	Ancestral (high confidence)
AZOLI_p10422	Unassigned	AZOBR_200173	Horizontally Transferred (high confidence)
AZOLI_p10426	Horizontally Transferred (high confidence)	AZOBR_200174	Horizontally Transferred (high confidence)
AZOLI_p10427	Unassigned	AZOBR_200175	Ancestral (low confidence)
AZOLI_p10429	Horizontally Transferred (high confidence)	AZOBR_200176	Ancestral (high confidence)
AZOLI_p10430	Horizontally Transferred (high confidence)	AZOBR_200177	Horizontally Transferred (high confidence)
AZOLI_p10431	Unassigned	AZOBR_200178	Ancestral (low confidence)
AZOLI_p10433	Unassigned	AZOBR_200179	Ancestral (high confidence)
AZOLI_p10434	Ancestral (high confidence)	AZOBR_200180	Unassigned
AZOLI_p10435	Ancestral (medium confidence)	AZOBR_200181	Ancestral (medium confidence)
AZOLI_p10436	Ancestral (high confidence)	AZOBR_200182	Ancestral (low confidence)
AZOLI_p10437	Horizontally Transferred (medium confidence)	AZOBR_200183	Ancestral (low confidence)
AZOLI_p10438	Horizontally Transferred (high confidence)	AZOBR_200184	Ancestral (low confidence)
AZOLI_p10440	Horizontally Transferred (high confidence)	AZOBR_200185	Unassigned
AZOLI_p10441	Horizontally Transferred (high confidence)	AZOBR_200186	Ancestral (high confidence)
AZOLI_p10442	Ancestral (high confidence)	AZOBR_200187	Ancestral (medium confidence)
AZOLI_p10443	Horizontally Transferred (high confidence)	AZOBR_200188	Ancestral (high confidence)
AZOLI_p10444	Unassigned	AZOBR_200189	Ancestral (low confidence)
AZOLI_p10445	Unassigned	AZOBR_200190	Ancestral (low confidence)
AZOLI_p10446	Unassigned	AZOBR_200191	Unassigned
AZOLI_p10447	Unassigned	AZOBR_200192	Ancestral (medium confidence)
AZOLI_p10448	Horizontally Transferred (high confidence)	AZOBR_200193	Horizontally Transferred (low confidence)
AZOLI_p10449	Ancestral (low confidence)	AZOBR_200194	Horizontally Transferred (high confidence)
AZOLI_p10450	Horizontally Transferred (high confidence)	AZOBR_200195	Ancestral (medium confidence)
AZOLI_p10451	Unassigned	AZOBR_200196	Ancestral (medium confidence)
AZOLI_p10452	Horizontally Transferred (low confidence)	AZOBR_200197	Ancestral (low confidence)
AZOLI_p10453	Horizontally Transferred (high confidence)	AZOBR_200198	Ancestral (low confidence)
AZOLI_p10454	Ancestral (medium confidence)	AZOBR_200199	Horizontally Transferred (high confidence)
AZOLI_p10455	Unassigned	AZOBR_200200	Ancestral (low confidence)
AZOLI_p10456	Horizontally Transferred (low confidence)	AZOBR_200201	Ancestral (low confidence)
AZOLI_p10457	Ancestral (low confidence)	AZOBR_200202	Unassigned
AZOLI_p10458	Unassigned	AZOBR_200203	Ancestral (low confidence)
AZOLI_p10459	Horizontally Transferred (low confidence)	AZOBR_200204	Horizontally Transferred (high confidence)
AZOLI_p10460	Horizontally Transferred (low confidence)	AZOBR_200205	Horizontally Transferred (medium confidence)
AZOLI_p10461	Unassigned	AZOBR_200206	Ancestral (low confidence)
AZOLI_p10462	Unassigned	AZOBR_200207	Ancestral (low confidence)
AZOLI_p10463	Ancestral (high confidence)	AZOBR_200208	Ancestral (medium confidence)
AZOLI_p10464	Horizontally Transferred (high confidence)	AZOBR_200209	Ancestral (medium confidence)
AZOLI_p10465	Horizontally Transferred (high confidence)	AZOBR_200210	Ancestral (medium confidence)
AZOLI_p10466	Unassigned	AZOBR_200211	Ancestral (medium confidence)

AZOLI_p10467	Horizontally Transferred (high confidence)	AZOBR_200212	Ancestral (medium confidence)
AZOLI_p10468	Horizontally Transferred (high confidence)	AZOBR_200213	Horizontally Transferred (high confidence)
AZOLI_p10469	Horizontally Transferred (high confidence)	AZOBR_200214	Horizontally Transferred (high confidence)
AZOLI_p10470	Ancestral (medium confidence)	AZOBR_200215	Ancestral (medium confidence)
AZOLI_p10471	Horizontally Transferred (high confidence)	AZOBR_200216	Ancestral (medium confidence)
AZOLI_p10472	Ancestral (low confidence)	AZOBR_200217	Ancestral (medium confidence)
AZOLI_p10473	Unassigned	AZOBR_200218	Ancestral (medium confidence)
AZOLI_p10475	Unassigned	AZOBR_200219	Ancestral (medium confidence)
AZOLI_p10476	Horizontally Transferred (high confidence)	AZOBR_200220	Ancestral (medium confidence)
AZOLI_p10477	Ancestral (low confidence)	AZOBR_200221	Ancestral (low confidence)
AZOLI_p10479	Unassigned	AZOBR_200222	Ancestral (medium confidence)
AZOLI_p10480	Ancestral (high confidence)	AZOBR_200223	Ancestral (high confidence)
AZOLI_p10481	Horizontally Transferred (high confidence)	AZOBR_200224	Unassigned
AZOLI_p10482	Ancestral (high confidence)	AZOBR_200225	Ancestral (low confidence)
AZOLI_p10483	Ancestral (medium confidence)	AZOBR_200226	Horizontally Transferred (high confidence)
AZOLI_p10484	Horizontally Transferred (high confidence)	AZOBR_200227	Unassigned
AZOLI_p10486	Horizontally Transferred (high confidence)	AZOBR_200228	Unassigned
AZOLI_p10487	Ancestral (high confidence)	AZOBR_200229	Horizontally Transferred (high confidence)
AZOLI_p10488	Unassigned	AZOBR_p110001	Ancestral (high confidence)
AZOLI_p10489	Ancestral (medium confidence)	AZOBR_p110002	Ancestral (high confidence)
AZOLI_p10490	Ancestral (low confidence)	AZOBR_p110003	Ancestral (medium confidence)
AZOLI_p10491	Horizontally Transferred (high confidence)	AZOBR_p110004	Ancestral (high confidence)
AZOLI_p10492	Horizontally Transferred (high confidence)	AZOBR_p110005	Ancestral (medium confidence)
AZOLI_p10493	Ancestral (low confidence)	AZOBR_p110006	Ancestral (high confidence)
AZOLI_p10494	Ancestral (low confidence)	AZOBR_p110007	Ancestral (high confidence)
AZOLI_p10495	Ancestral (medium confidence)	AZOBR_p110008	Ancestral (high confidence)
AZOLI_p10496	Unassigned	AZOBR_p110009	Ancestral (high confidence)
AZOLI_p10497	Ancestral (medium confidence)	AZOBR_p110010	Ancestral (high confidence)
AZOLI_p10498	Ancestral (low confidence)	AZOBR_p110011	Ancestral (high confidence)
AZOLI_p10499	Ancestral (low confidence)	AZOBR_p110012	Ancestral (high confidence)
AZOLI_p10500	Ancestral (low confidence)	AZOBR_p110013	Ancestral (high confidence)
AZOLI_p10501	Horizontally Transferred (low confidence)	AZOBR_p110014	Unassigned
AZOLI_p10502	Ancestral (medium confidence)	AZOBR_p110015	Unassigned
AZOLI_p10503	Horizontally Transferred (high confidence)	AZOBR_p110016	Ancestral (high confidence)
AZOLI_p10504	Horizontally Transferred (high confidence)	AZOBR_p110017	Ancestral (low confidence)
AZOLI_p10505	Horizontally Transferred (high confidence)	AZOBR_p110018	Ancestral (high confidence)
AZOLI_p10507	Horizontally Transferred (high confidence)	AZOBR_p110019	Ancestral (high confidence)
AZOLI_p10508	Horizontally Transferred (high confidence)	AZOBR_p110020	Ancestral (high confidence)
AZOLI_p10509	Horizontally Transferred (high confidence)	AZOBR_p110021	Unassigned
AZOLI_p10510	Horizontally Transferred (high confidence)	AZOBR_p110022	Horizontally Transferred (high confidence)
AZOLI_p10511	Horizontally Transferred (medium confidence)	AZOBR_p110023	Horizontally Transferred (high confidence)
AZOLI_p10512	Horizontally Transferred (high confidence)	AZOBR_p110024	Ancestral (medium confidence)
AZOLI_p10513	Ancestral (medium confidence)	AZOBR_p110025	Unassigned
AZOLI_p10514	Ancestral (low confidence)	AZOBR_p110026	Horizontally Transferred (high confidence)
AZOLI_p10515	Horizontally Transferred (high confidence)	AZOBR_p110027	Ancestral (medium confidence)
AZOLI_p10516	Horizontally Transferred (high confidence)	AZOBR_p110028	Ancestral (low confidence)
AZOLI_p10517	Ancestral (low confidence)	AZOBR_p110029	Ancestral (low confidence)
AZOLI_p10518	Horizontally Transferred (medium confidence)	AZOBR_p110030	Ancestral (medium confidence)
AZOLI_p10520	Horizontally Transferred (high confidence)	AZOBR_p110031	Ancestral (low confidence)
AZOLI_p10521	Unassigned	AZOBR_p110032	Ancestral (low confidence)
AZOLI_p10522	Unassigned	AZOBR_p110033	Ancestral (low confidence)
AZOLI_p10523	Ancestral (low confidence)	AZOBR_p110034	Ancestral (medium confidence)
AZOLI_p10524	Horizontally Transferred (high confidence)	AZOBR_p110035	Unassigned
AZOLI_p10525	Horizontally Transferred (high confidence)	AZOBR_p110036	Ancestral (low confidence)
AZOLI_p10526	Unassigned	AZOBR_p110037	Ancestral (low confidence)
AZOLI_p10527	Ancestral (high confidence)	AZOBR_p110038	Ancestral (low confidence)
AZOLI_p10528	Horizontally Transferred (high confidence)	AZOBR_p110039	Horizontally Transferred (high confidence)
AZOLI_p10529	Horizontally Transferred (high confidence)	AZOBR_p110040	Horizontally Transferred (high confidence)
AZOLI_p10531	Horizontally Transferred (high confidence)	AZOBR_p110041	Ancestral (low confidence)
AZOLI_p10532	Ancestral (low confidence)	AZOBR_p110042	Horizontally Transferred (high confidence)
AZOLI_p10533	Unassigned	AZOBR_p110043	Horizontally Transferred (high confidence)
AZOLI_p10534	Unassigned	AZOBR_p110044	Horizontally Transferred (high confidence)
AZOLI_p10535	Unassigned	AZOBR_p110045	Horizontally Transferred (high confidence)
AZOLI_p10536	Ancestral (low confidence)	AZOBR_p110046	Ancestral (medium confidence)
AZOLI_p10548	Horizontally Transferred (high confidence)	AZOBR_p110047	Ancestral (medium confidence)
AZOLI_p10549	Horizontally Transferred (high confidence)	AZOBR_p110048	Ancestral (medium confidence)
AZOLI_p10550	Horizontally Transferred (high confidence)	AZOBR_p110049	Horizontally Transferred (medium confidence)
AZOLI_p10551	Horizontally Transferred (high confidence)	AZOBR_p110050	Ancestral (medium confidence)
AZOLI_p10552	Horizontally Transferred (high confidence)	AZOBR_p110051	Ancestral (low confidence)
AZOLI_p10553	Horizontally Transferred (high confidence)	AZOBR_p110052	Ancestral (low confidence)
AZOLI_p10554	Horizontally Transferred (high confidence)	AZOBR_p110053	Ancestral (high confidence)
AZOLI_p10555	Horizontally Transferred (high confidence)	AZOBR_p110054	Horizontally Transferred (high confidence)
AZOLI_p10556	Horizontally Transferred (high confidence)	AZOBR_p110055	Horizontally Transferred (high confidence)
AZOLI_p10557	Horizontally Transferred (high confidence)	AZOBR_p110056	Unassigned
AZOLI_p10558	Horizontally Transferred (high confidence)	AZOBR_p110057	Unassigned

AZOLI_p10559	Horizontally Transferred (high confidence)	AZOBR_p110058	Ancestral (medium confidence)
AZOLI_p10560	Horizontally Transferred (high confidence)	AZOBR_p110059	Horizontally Transferred (low confidence)
AZOLI_p10561	Horizontally Transferred (high confidence)	AZOBR_p110060	Horizontally Transferred (high confidence)
AZOLI_p10562	Horizontally Transferred (high confidence)	AZOBR_p110061	Horizontally Transferred (high confidence)
AZOLI_p10563	Horizontally Transferred (high confidence)	AZOBR_p110062	Horizontally Transferred (high confidence)
AZOLI_p10564	Ancestral (medium confidence)	AZOBR_p110063	Horizontally Transferred (high confidence)
AZOLI_p10565	Horizontally Transferred (high confidence)	AZOBR_p110064	Unassigned
AZOLI_p10566	Ancestral (low confidence)	AZOBR_p110065	Ancestral (low confidence)
AZOLI_p10567	Ancestral (low confidence)	AZOBR_p110066	Unassigned
AZOLI_p10569	Horizontally Transferred (high confidence)	AZOBR_p110067	Ancestral (low confidence)
AZOLI_p10570	Ancestral (medium confidence)	AZOBR_p110068	Horizontally Transferred (high confidence)
AZOLI_p10572	Ancestral (medium confidence)	AZOBR_p110069	Horizontally Transferred (high confidence)
AZOLI_p10573	Ancestral (medium confidence)	AZOBR_p110070	Ancestral (low confidence)
AZOLI_p10574	Horizontally Transferred (high confidence)	AZOBR_p110071	Ancestral (low confidence)
AZOLI_p10575	Horizontally Transferred (high confidence)	AZOBR_p110072	Ancestral (low confidence)
AZOLI_p10576	Ancestral (low confidence)	AZOBR_p110073	Ancestral (medium confidence)
AZOLI_p10577	Ancestral (medium confidence)	AZOBR_p110074	Ancestral (medium confidence)
AZOLI_p10578	Horizontally Transferred (high confidence)	AZOBR_p110075	Ancestral (high confidence)
AZOLI_p10579	Horizontally Transferred (medium confidence)	AZOBR_p110076	Ancestral (medium confidence)
AZOLI_p10580	Horizontally Transferred (high confidence)	AZOBR_p110077	Ancestral (high confidence)
AZOLI_p10581	Horizontally Transferred (high confidence)	AZOBR_p110078	Unassigned
AZOLI_p10582	Horizontally Transferred (high confidence)	AZOBR_p110079	Horizontally Transferred (high confidence)
AZOLI_p10583	Horizontally Transferred (high confidence)	AZOBR_p110080	Ancestral (high confidence)
AZOLI_p10584	Unassigned	AZOBR_p110081	Unassigned
AZOLI_p10585	Ancestral (medium confidence)	AZOBR_p110082	Ancestral (low confidence)
AZOLI_p10586	Unassigned	AZOBR_p110083	Ancestral (low confidence)
AZOLI_p10587	Horizontally Transferred (high confidence)	AZOBR_p110084	Unassigned
AZOLI_p10589	Ancestral (low confidence)	AZOBR_p110085	Unassigned
AZOLI_p10590	Horizontally Transferred (medium confidence)	AZOBR_p110086	Ancestral (medium confidence)
AZOLI_p10591	Unassigned	AZOBR_p110087	Unassigned
AZOLI_p10592	Unassigned	AZOBR_p110088	Ancestral (medium confidence)
AZOLI_p10595	Ancestral (low confidence)	AZOBR_p110089	Ancestral (low confidence)
AZOLI_p10596	Horizontally Transferred (high confidence)	AZOBR_p110090	Ancestral (medium confidence)
AZOLI_p10597	Horizontally Transferred (high confidence)	AZOBR_p110091	Ancestral (medium confidence)
AZOLI_p10598	Ancestral (low confidence)	AZOBR_p110092	Ancestral (medium confidence)
AZOLI_p10599	Ancestral (medium confidence)	AZOBR_p110093	Ancestral (medium confidence)
AZOLI_p10600	Ancestral (medium confidence)	AZOBR_p110094	Ancestral (medium confidence)
AZOLI_p10601	Unassigned	AZOBR_p110095	Ancestral (high confidence)
AZOLI_p10602	Ancestral (medium confidence)	AZOBR_p110096	Ancestral (medium confidence)
AZOLI_p10604	Unassigned	AZOBR_p110097	Unassigned
AZOLI_p10605	Ancestral (medium confidence)	AZOBR_p110098	Unassigned
AZOLI_p10606	Ancestral (medium confidence)	AZOBR_p110099	Horizontally Transferred (high confidence)
AZOLI_p10607	Ancestral (medium confidence)	AZOBR_p110100	Horizontally Transferred (high confidence)
AZOLI_p10608	Horizontally Transferred (high confidence)	AZOBR_p110101	Ancestral (low confidence)
AZOLI_p10609	Horizontally Transferred (high confidence)	AZOBR_p110102	Ancestral (low confidence)
AZOLI_p10610	Horizontally Transferred (low confidence)	AZOBR_p110103	Ancestral (low confidence)
AZOLI_p10611	Unassigned	AZOBR_p110104	Ancestral (medium confidence)
AZOLI_p10612	Ancestral (low confidence)	AZOBR_p110105	Ancestral (medium confidence)
AZOLI_p10613	Ancestral (low confidence)	AZOBR_p110106	Ancestral (medium confidence)
AZOLI_p10614	Ancestral (low confidence)	AZOBR_p110107	Ancestral (medium confidence)
AZOLI_p10615	Ancestral (low confidence)	AZOBR_p110108	Ancestral (low confidence)
AZOLI_p10616	Ancestral (low confidence)	AZOBR_p110109	Ancestral (low confidence)
AZOLI_p10617	Ancestral (low confidence)	AZOBR_p110110	Horizontally Transferred (medium confidence)
AZOLI_p10618	Ancestral (low confidence)	AZOBR_p110111	Ancestral (high confidence)
AZOLI_p10619	Ancestral (high confidence)	AZOBR_p110112	Horizontally Transferred (high confidence)
AZOLI_p10620	Ancestral (low confidence)	AZOBR_p110113	Unassigned
AZOLI_p10621	Horizontally Transferred (high confidence)	AZOBR_p110114	Unassigned
AZOLI_p10622	Horizontally Transferred (medium confidence)	AZOBR_p110115	Unassigned
AZOLI_p10623	Unassigned	AZOBR_p110116	Unassigned
AZOLI_p10624	Unassigned	AZOBR_p110117	Ancestral (low confidence)
AZOLI_p10625	Ancestral (medium confidence)	AZOBR_p110118	Horizontally Transferred (medium confidence)
AZOLI_p10626	Horizontally Transferred (high confidence)	AZOBR_p110119	Ancestral (low confidence)
AZOLI_p10627	Unassigned	AZOBR_p110120	Ancestral (medium confidence)
AZOLI_p10628	Horizontally Transferred (high confidence)	AZOBR_p110121	Ancestral (medium confidence)
AZOLI_p10629	Horizontally Transferred (high confidence)	AZOBR_p110122	Ancestral (high confidence)
AZOLI_p10630	Horizontally Transferred (high confidence)	AZOBR_p110123	Unassigned
AZOLI_p10633	Ancestral (medium confidence)	AZOBR_p110124	Horizontally Transferred (high confidence)
AZOLI_p10634	Ancestral (medium confidence)	AZOBR_p110125	Ancestral (low confidence)
AZOLI_p10635	Unassigned	AZOBR_p110126	Unassigned
AZOLI_p10636	Ancestral (medium confidence)	AZOBR_p110127	Ancestral (high confidence)
AZOLI_p10637	Horizontally Transferred (high confidence)	AZOBR_p110128	Ancestral (medium confidence)
AZOLI_p10638	Ancestral (low confidence)	AZOBR_p110129	Ancestral (medium confidence)
AZOLI_p10640	Ancestral (low confidence)	AZOBR_p110130	Ancestral (medium confidence)
AZOLI_p10641	Unassigned	AZOBR_p110131	Ancestral (high confidence)
AZOLI_p10642	Ancestral (medium confidence)	AZOBR_p110132	Ancestral (medium confidence)

AZOLI_p10643	Ancestral (low confidence)	AZOBR_p110133	Ancestral (medium confidence)
AZOLI_p10644	Horizontally Transferred (high confidence)	AZOBR_p110134	Ancestral (low confidence)
AZOLI_p10645	Ancestral (medium confidence)	AZOBR_p110135	Unassigned
AZOLI_p10646	Ancestral (medium confidence)	AZOBR_p110136	Ancestral (low confidence)
AZOLI_p10647	Ancestral (medium confidence)	AZOBR_p110137	Ancestral (low confidence)
AZOLI_p10648	Horizontally Transferred (high confidence)	AZOBR_p110138	Ancestral (low confidence)
AZOLI_p10649	Ancestral (high confidence)	AZOBR_p110139	Ancestral (low confidence)
AZOLI_p10650	Ancestral (medium confidence)	AZOBR_p110140	Ancestral (low confidence)
AZOLI_p10651	Horizontally Transferred (high confidence)	AZOBR_p110141	Horizontally Transferred (low confidence)
AZOLI_p10652	Ancestral (medium confidence)	AZOBR_p110142	Ancestral (medium confidence)
AZOLI_p10653	Ancestral (medium confidence)	AZOBR_p110143	Unassigned
AZOLI_p10654	Ancestral (medium confidence)	AZOBR_p110144	Ancestral (medium confidence)
AZOLI_p10655	Horizontally Transferred (high confidence)	AZOBR_p110145	Unassigned
AZOLI_p10656	Horizontally Transferred (high confidence)	AZOBR_p110146	Ancestral (high confidence)
AZOLI_p10657	Horizontally Transferred (high confidence)	AZOBR_p110147	Ancestral (medium confidence)
AZOLI_p10658	Horizontally Transferred (high confidence)	AZOBR_p110148	Ancestral (medium confidence)
AZOLI_p10659	Ancestral (low confidence)	AZOBR_p110149	Ancestral (high confidence)
AZOLI_p10660	Horizontally Transferred (high confidence)	AZOBR_p110150	Ancestral (medium confidence)
AZOLI_p10661	Horizontally Transferred (high confidence)	AZOBR_p110151	Unassigned
AZOLI_p10662	Ancestral (low confidence)	AZOBR_p110152	Horizontally Transferred (high confidence)
AZOLI_p10663	Ancestral (medium confidence)	AZOBR_p110153	Horizontally Transferred (medium confidence)
AZOLI_p10665	Horizontally Transferred (high confidence)	AZOBR_p110154	Horizontally Transferred (high confidence)
AZOLI_p10666	Ancestral (medium confidence)	AZOBR_p110155	Ancestral (low confidence)
AZOLI_p10667	Ancestral (medium confidence)	AZOBR_p110156	Ancestral (low confidence)
AZOLI_p10668	Ancestral (medium confidence)	AZOBR_p110157	Unassigned
AZOLI_p10669	Horizontally Transferred (high confidence)	AZOBR_p110158	Ancestral (low confidence)
AZOLI_p10670	Ancestral (low confidence)	AZOBR_p120001	Ancestral (low confidence)
AZOLI_p10671	Ancestral (low confidence)	AZOBR_p120002	Unassigned
AZOLI_p10672	Horizontally Transferred (high confidence)	AZOBR_p120003	Ancestral (low confidence)
AZOLI_p10673	Horizontally Transferred (high confidence)	AZOBR_p120004	Unassigned
AZOLI_p10674	Horizontally Transferred (medium confidence)	AZOBR_p120005	Ancestral (medium confidence)
AZOLI_p10675	Horizontally Transferred (high confidence)	AZOBR_p120006	Unassigned
AZOLI_p10676	Horizontally Transferred (high confidence)	AZOBR_p120007	Unassigned
AZOLI_p10677	Horizontally Transferred (high confidence)	AZOBR_p120008	Horizontally Transferred (high confidence)
AZOLI_p10679	Ancestral (low confidence)	AZOBR_p120009	Horizontally Transferred (high confidence)
AZOLI_p10680	Horizontally Transferred (medium confidence)	AZOBR_p120010	Unassigned
AZOLI_p10681	Horizontally Transferred (high confidence)	AZOBR_p120011	Unassigned
AZOLI_p10682	Ancestral (low confidence)	AZOBR_p120012	Unassigned
AZOLI_p10683	Horizontally Transferred (low confidence)	AZOBR_p120013	Unassigned
AZOLI_p10684	Unassigned	AZOBR_p120014	Horizontally Transferred (medium confidence)
AZOLI_p10685	Horizontally Transferred (medium confidence)	AZOBR_p120015	Unassigned
AZOLI_p10686	Horizontally Transferred (medium confidence)	AZOBR_p120016	Horizontally Transferred (low confidence)
AZOLI_p10687	Horizontally Transferred (low confidence)	AZOBR_p120017	Ancestral (medium confidence)
AZOLI_p10688	Horizontally Transferred (low confidence)	AZOBR_p120018	Ancestral (medium confidence)
AZOLI_p10689	Ancestral (low confidence)	AZOBR_p120019	Unassigned
AZOLI_p10690	Horizontally Transferred (medium confidence)	AZOBR_p120020	Unassigned
AZOLI_p10691	Horizontally Transferred (medium confidence)	AZOBR_p120021	Horizontally Transferred (high confidence)
AZOLI_p10692	Horizontally Transferred (high confidence)	AZOBR_p120022	Unassigned
AZOLI_p10693	Horizontally Transferred (medium confidence)	AZOBR_p120023	Unassigned
AZOLI_p10694	Horizontally Transferred (low confidence)	AZOBR_p120024	Horizontally Transferred (high confidence)
AZOLI_p10695	Ancestral (low confidence)	AZOBR_p120025	Horizontally Transferred (high confidence)
AZOLI_p10696	Horizontally Transferred (medium confidence)	AZOBR_p120026	Unassigned
AZOLI_p10697	Unassigned	AZOBR_p120027	Ancestral (medium confidence)
AZOLI_p10698	Horizontally Transferred (high confidence)	AZOBR_p120028	Ancestral (high confidence)
AZOLI_p10699	Horizontally Transferred (low confidence)	AZOBR_p120029	Unassigned
AZOLI_p10700	Horizontally Transferred (low confidence)	AZOBR_p120030	Unassigned
AZOLI_p10701	Horizontally Transferred (low confidence)	AZOBR_p120031	Horizontally Transferred (low confidence)
AZOLI_p10702	Horizontally Transferred (low confidence)	AZOBR_p120032	Unassigned
AZOLI_p10703	Horizontally Transferred (medium confidence)	AZOBR_p120033	Horizontally Transferred (high confidence)
AZOLI_p10704	Horizontally Transferred (medium confidence)	AZOBR_p120034	Unassigned
AZOLI_p10705	Ancestral (low confidence)	AZOBR_p120035	Horizontally Transferred (high confidence)
AZOLI_p10706	Ancestral (low confidence)	AZOBR_p120036	Unassigned
AZOLI_p10707	Horizontally Transferred (high confidence)	AZOBR_p120037	Unassigned
AZOLI_p10708	Ancestral (high confidence)	AZOBR_p120038	Ancestral (medium confidence)
AZOLI_p10710	Horizontally Transferred (medium confidence)	AZOBR_p120039	Horizontally Transferred (high confidence)
AZOLI_p10711	Horizontally Transferred (low confidence)	AZOBR_p120040	Unassigned
AZOLI_p10712	Horizontally Transferred (low confidence)	AZOBR_p120041	Unassigned
AZOLI_p10713	Unassigned	AZOBR_p120042	Unassigned
AZOLI_p10714	Horizontally Transferred (medium confidence)	AZOBR_p120043	Unassigned
AZOLI_p10715	Ancestral (medium confidence)	AZOBR_p120044	Unassigned
AZOLI_p10716	Ancestral (low confidence)	AZOBR_p120045	Unassigned
AZOLI_p10717	Ancestral (low confidence)	AZOBR_p120046	Unassigned
AZOLI_p10719	Ancestral (low confidence)	AZOBR_p120047	Unassigned
AZOLI_p10720	Ancestral (low confidence)	AZOBR_p120048	Unassigned
AZOLI_p10721	Ancestral (low confidence)	AZOBR_p120049	Horizontally Transferred (low confidence)

AZOLI_p10722	Ancestral (low confidence)	AZOBR_p120050	Unassigned
AZOLI_p10723	Ancestral (low confidence)	AZOBR_p120051	Unassigned
AZOLI_p10724	Ancestral (low confidence)	AZOBR_p120052	Ancestral (high confidence)
AZOLI_p10725	Ancestral (low confidence)	AZOBR_p120053	Ancestral (low confidence)
AZOLI_p10726	Horizontally Transferred (high confidence)	AZOBR_p120054	Horizontally Transferred (high confidence)
AZOLI_p10727	Horizontally Transferred (medium confidence)	AZOBR_p120055	Unassigned
AZOLI_p10728	Horizontally Transferred (low confidence)	AZOBR_p120056	Unassigned
AZOLI_p10729	Horizontally Transferred (medium confidence)	AZOBR_p120057	Unassigned
AZOLI_p10730	Horizontally Transferred (medium confidence)	AZOBR_p120058	Unassigned
AZOLI_p10731	Horizontally Transferred (medium confidence)	AZOBR_p120059	Horizontally Transferred (high confidence)
AZOLI_p10732	Horizontally Transferred (medium confidence)	AZOBR_p120060	Unassigned
AZOLI_p10735	Horizontally Transferred (medium confidence)	AZOBR_p120061	Horizontally Transferred (high confidence)
AZOLI_p10736	Horizontally Transferred (low confidence)	AZOBR_p120062	Horizontally Transferred (high confidence)
AZOLI_p10737	Horizontally Transferred (high confidence)	AZOBR_p120063	Unassigned
AZOLI_p10738	Horizontally Transferred (high confidence)	AZOBR_p120064	Horizontally Transferred (high confidence)
AZOLI_p10751	Horizontally Transferred (high confidence)	AZOBR_p120065	Ancestral (low confidence)
AZOLI_p10752	Horizontally Transferred (high confidence)	AZOBR_p120066	Unassigned
AZOLI_p10753	Ancestral (medium confidence)	AZOBR_p120067	Unassigned
AZOLI_p10754	Unassigned	AZOBR_p120068	Ancestral (high confidence)
AZOLI_p10755	Horizontally Transferred (low confidence)	AZOBR_p120069	Ancestral (high confidence)
AZOLI_p10756	Horizontally Transferred (high confidence)	AZOBR_p120070	Unassigned
AZOLI_p10758	Horizontally Transferred (high confidence)	AZOBR_p120071	Ancestral (low confidence)
AZOLI_p10759	Horizontally Transferred (high confidence)	AZOBR_p120072	Horizontally Transferred (high confidence)
AZOLI_p10760	Horizontally Transferred (high confidence)	AZOBR_p120073	Horizontally Transferred (high confidence)
AZOLI_p10761	Ancestral (low confidence)	AZOBR_p120074	Horizontally Transferred (high confidence)
AZOLI_p10762	Ancestral (high confidence)	AZOBR_p120075	Horizontally Transferred (high confidence)
AZOLI_p10763	Ancestral (high confidence)	AZOBR_p120076	Unassigned
AZOLI_p10764	Ancestral (high confidence)	AZOBR_p120077	Unassigned
AZOLI_p10765	Horizontally Transferred (medium confidence)	AZOBR_p120078	Unassigned
AZOLI_p10766	Ancestral (medium confidence)	AZOBR_p120079	Unassigned
AZOLI_p10767	Ancestral (high confidence)	AZOBR_p120080	Horizontally Transferred (high confidence)
AZOLI_p10768	Ancestral (medium confidence)	AZOBR_p120081	Unassigned
AZOLI_p10769	Ancestral (low confidence)	AZOBR_p120082	Ancestral (high confidence)
AZOLI_p10770	Ancestral (medium confidence)	AZOBR_p120083	Unassigned
AZOLI_p10771	Ancestral (high confidence)	AZOBR_p120084	Unassigned
AZOLI_p10772	Ancestral (medium confidence)	AZOBR_p120085	Ancestral (low confidence)
AZOLI_p10773	Ancestral (high confidence)	AZOBR_p120086	Ancestral (medium confidence)
AZOLI_p10774	Ancestral (high confidence)	AZOBR_p120087	Unassigned
AZOLI_p10776	Ancestral (medium confidence)	AZOBR_p120088	Ancestral (low confidence)
AZOLI_p10777	Horizontally Transferred (high confidence)	AZOBR_p120089	Ancestral (medium confidence)
AZOLI_p10778	Ancestral (medium confidence)	AZOBR_p120090	Ancestral (medium confidence)
AZOLI_p10779	Ancestral (high confidence)	AZOBR_p120091	Ancestral (medium confidence)
AZOLI_p10780	Ancestral (low confidence)	AZOBR_p120092	Ancestral (medium confidence)
AZOLI_p10781	Unassigned	AZOBR_p120093	Ancestral (medium confidence)
AZOLI_p10782	Ancestral (medium confidence)	AZOBR_p120094	Ancestral (high confidence)
AZOLI_p10783	Ancestral (medium confidence)	AZOBR_p120095	Ancestral (medium confidence)
AZOLI_p10784	Ancestral (medium confidence)	AZOBR_p120096	Ancestral (medium confidence)
AZOLI_p10785	Unassigned	AZOBR_p120097	Ancestral (high confidence)
AZOLI_p10786	Horizontally Transferred (high confidence)	AZOBR_p120098	Ancestral (low confidence)
AZOLI_p10787	Horizontally Transferred (high confidence)	AZOBR_p120099	Ancestral (high confidence)
AZOLI_p10788	Unassigned	AZOBR_p120100	Ancestral (high confidence)
AZOLI_p10789	Unassigned	AZOBR_p120101	Ancestral (high confidence)
AZOLI_p10790	Unassigned	AZOBR_p120102	Ancestral (high confidence)
AZOLI_p10791	Unassigned	AZOBR_p120103	Ancestral (high confidence)
AZOLI_p10793	Unassigned	AZOBR_p120104	Ancestral (high confidence)
AZOLI_p10794	Horizontally Transferred (low confidence)	AZOBR_p120105	Ancestral (low confidence)
AZOLI_p10797	Horizontally Transferred (high confidence)	AZOBR_p120106	Horizontally Transferred (low confidence)
AZOLI_p10798	Horizontally Transferred (high confidence)	AZOBR_p120107	Ancestral (high confidence)
AZOLI_p10801	Unassigned	AZOBR_p120108	Ancestral (high confidence)
AZOLI_p10802	Unassigned	AZOBR_p120109	Ancestral (low confidence)
AZOLI_p10804	Horizontally Transferred (high confidence)	AZOBR_p120110	Ancestral (low confidence)
AZOLI_p10806	Ancestral (low confidence)	AZOBR_p120111	Ancestral (high confidence)
AZOLI_p10807	Unassigned	AZOBR_p120112	Ancestral (high confidence)
AZOLI_p10808	Ancestral (low confidence)	AZOBR_p120113	Ancestral (medium confidence)
AZOLI_p10809	Ancestral (high confidence)	AZOBR_p120114	Ancestral (low confidence)
AZOLI_p10810	Ancestral (medium confidence)	AZOBR_p120115	Ancestral (medium confidence)
AZOLI_p10811	Ancestral (medium confidence)	AZOBR_p120116	Ancestral (high confidence)
AZOLI_p10812	Horizontally Transferred (high confidence)	AZOBR_p120117	Ancestral (high confidence)
AZOLI_p10813	Ancestral (low confidence)	AZOBR_p120118	Ancestral (medium confidence)
AZOLI_p10814	Ancestral (low confidence)	AZOBR_p120119	Ancestral (medium confidence)
AZOLI_p10815	Ancestral (low confidence)	AZOBR_p120120	Ancestral (medium confidence)
AZOLI_p10817	Horizontally Transferred (high confidence)	AZOBR_p120121	Ancestral (high confidence)
AZOLI_p10818	Horizontally Transferred (medium confidence)	AZOBR_p120122	Ancestral (medium confidence)
AZOLI_p10820	Ancestral (low confidence)	AZOBR_p120123	Unassigned
AZOLI_p10821	Ancestral (medium confidence)	AZOBR_p120124	Unassigned

AZOLI_p10822	Horizontally Transferred (high confidence)	AZOBR_p120125	Ancestral (medium confidence)
AZOLI_p10823	Ancestral (low confidence)	AZOBR_p120126	Ancestral (low confidence)
AZOLI_p10825	Ancestral (high confidence)	AZOBR_p120127	Unassigned
AZOLI_p10826	Ancestral (low confidence)	AZOBR_p120128	Unassigned
AZOLI_p10827	Ancestral (low confidence)	AZOBR_p120129	Ancestral (low confidence)
AZOLI_p10829	Horizontally Transferred (high confidence)	AZOBR_p120130	Ancestral (low confidence)
AZOLI_p10830	Horizontally Transferred (high confidence)	AZOBR_p130001	Horizontally Transferred (medium confidence)
AZOLI_p10832	Horizontally Transferred (high confidence)	AZOBR_p130002	Ancestral (low confidence)
AZOLI_p10833	Horizontally Transferred (high confidence)	AZOBR_p130003	Horizontally Transferred (high confidence)
AZOLI_p10834	Horizontally Transferred (high confidence)	AZOBR_p130004	Horizontally Transferred (high confidence)
AZOLI_p10835	Horizontally Transferred (high confidence)	AZOBR_p130005	Horizontally Transferred (high confidence)
AZOLI_p10836	Ancestral (low confidence)	AZOBR_p130006	Horizontally Transferred (high confidence)
AZOLI_p10837	Unassigned	AZOBR_p130007	Horizontally Transferred (medium confidence)
AZOLI_p10838	Horizontally Transferred (high confidence)	AZOBR_p130008	Horizontally Transferred (high confidence)
AZOLI_p10839	Ancestral (low confidence)	AZOBR_p130009	Horizontally Transferred (high confidence)
AZOLI_p10840	Horizontally Transferred (high confidence)	AZOBR_p130010	Ancestral (low confidence)
AZOLI_p10841	Horizontally Transferred (high confidence)	AZOBR_p130011	Horizontally Transferred (high confidence)
AZOLI_p10842	Horizontally Transferred (high confidence)	AZOBR_p130012	Ancestral (low confidence)
AZOLI_p10843	Ancestral (low confidence)	AZOBR_p130013	Horizontally Transferred (high confidence)
AZOLI_p10844	Ancestral (low confidence)	AZOBR_p130014	Ancestral (low confidence)
AZOLI_p10845	Ancestral (low confidence)	AZOBR_p130015	Ancestral (medium confidence)
AZOLI_p10846	Horizontally Transferred (high confidence)	AZOBR_p130016	Ancestral (medium confidence)
AZOLI_p10847	Horizontally Transferred (medium confidence)	AZOBR_p130017	Ancestral (medium confidence)
AZOLI_p10848	Horizontally Transferred (high confidence)	AZOBR_p130018	Unassigned
AZOLI_p10849	Ancestral (low confidence)	AZOBR_p130019	Ancestral (high confidence)
AZOLI_p10850	Ancestral (low confidence)	AZOBR_p130020	Horizontally Transferred (high confidence)
AZOLI_p10852	Ancestral (low confidence)	AZOBR_p130021	Horizontally Transferred (high confidence)
AZOLI_p10853	Horizontally Transferred (high confidence)	AZOBR_p130022	Horizontally Transferred (high confidence)
AZOLI_p10855	Horizontally Transferred (medium confidence)	AZOBR_p130023	Ancestral (low confidence)
AZOLI_p10856	Horizontally Transferred (medium confidence)	AZOBR_p130024	Horizontally Transferred (low confidence)
AZOLI_p10857	Horizontally Transferred (medium confidence)	AZOBR_p130025	Horizontally Transferred (high confidence)
AZOLI_p10858	Horizontally Transferred (high confidence)	AZOBR_p130026	Horizontally Transferred (high confidence)
AZOLI_p10859	Horizontally Transferred (medium confidence)	AZOBR_p130027	Ancestral (low confidence)
AZOLI_p10860	Horizontally Transferred (medium confidence)	AZOBR_p130028	Horizontally Transferred (high confidence)
AZOLI_p10861	Ancestral (high confidence)	AZOBR_p130029	Horizontally Transferred (high confidence)
AZOLI_p10862	Unassigned	AZOBR_p130030	Horizontally Transferred (high confidence)
AZOLI_p10863	Unassigned	AZOBR_p130031	Horizontally Transferred (high confidence)
AZOLI_p10864	Ancestral (low confidence)	AZOBR_p130032	Horizontally Transferred (high confidence)
AZOLI_p10865	Unassigned	AZOBR_p130033	Ancestral (low confidence)
AZOLI_p10866	Unassigned	AZOBR_p130034	Ancestral (high confidence)
AZOLI_p10867	Horizontally Transferred (high confidence)	AZOBR_p130035	Horizontally Transferred (medium confidence)
AZOLI_p10869	Horizontally Transferred (low confidence)	AZOBR_p130036	Horizontally Transferred (high confidence)
AZOLI_p10870	Horizontally Transferred (medium confidence)	AZOBR_p130037	Unassigned
AZOLI_p10871	Horizontally Transferred (low confidence)	AZOBR_p130038	Horizontally Transferred (high confidence)
AZOLI_p10873	Horizontally Transferred (low confidence)	AZOBR_p130039	Horizontally Transferred (high confidence)
AZOLI_p10874	Horizontally Transferred (low confidence)	AZOBR_p130040	Horizontally Transferred (high confidence)
AZOLI_p10875	Horizontally Transferred (low confidence)	AZOBR_p130041	Horizontally Transferred (medium confidence)
AZOLI_p10876	Horizontally Transferred (high confidence)	AZOBR_p130042	Ancestral (low confidence)
AZOLI_p10877	Horizontally Transferred (high confidence)	AZOBR_p130043	Horizontally Transferred (high confidence)
AZOLI_p10878	Horizontally Transferred (high confidence)	AZOBR_p130044	Horizontally Transferred (high confidence)
AZOLI_p10879	Horizontally Transferred (medium confidence)	AZOBR_p130045	Horizontally Transferred (high confidence)
AZOLI_p10880	Ancestral (medium confidence)	AZOBR_p130046	Unassigned
AZOLI_p10882	Horizontally Transferred (high confidence)	AZOBR_p130047	Horizontally Transferred (high confidence)
AZOLI_p10883	Horizontally Transferred (low confidence)	AZOBR_p130048	Horizontally Transferred (high confidence)
AZOLI_p10884	Horizontally Transferred (high confidence)	AZOBR_p130049	Horizontally Transferred (high confidence)
AZOLI_p10885	Horizontally Transferred (low confidence)	AZOBR_p130050	Unassigned
AZOLI_p10886	Horizontally Transferred (medium confidence)	AZOBR_p130051	Horizontally Transferred (high confidence)
AZOLI_p10887	Horizontally Transferred (medium confidence)	AZOBR_p130052	Horizontally Transferred (medium confidence)
AZOLI_p10888	Horizontally Transferred (low confidence)	AZOBR_p130053	Horizontally Transferred (high confidence)
AZOLI_p10889	Horizontally Transferred (medium confidence)	AZOBR_p130054	Horizontally Transferred (high confidence)
AZOLI_p10890	Horizontally Transferred (medium confidence)	AZOBR_p130055	Unassigned
AZOLI_p10891	Ancestral (low confidence)	AZOBR_p130056	Ancestral (medium confidence)
AZOLI_p10892	Ancestral (low confidence)	AZOBR_p130057	Ancestral (low confidence)
AZOLI_p10893	Horizontally Transferred (medium confidence)	AZOBR_p130058	Unassigned
AZOLI_p10894	Horizontally Transferred (medium confidence)	AZOBR_p130059	Unassigned
AZOLI_p10895	Horizontally Transferred (medium confidence)	AZOBR_p130060	Unassigned
AZOLI_p10896	Horizontally Transferred (medium confidence)	AZOBR_p130061	Horizontally Transferred (high confidence)
AZOLI_p10898	Ancestral (low confidence)	AZOBR_p130062	Ancestral (medium confidence)
AZOLI_p10900	Ancestral (high confidence)	AZOBR_p130063	Ancestral (medium confidence)
AZOLI_p10901	Horizontally Transferred (medium confidence)	AZOBR_p130064	Ancestral (medium confidence)
AZOLI_p10902	Horizontally Transferred (low confidence)	AZOBR_p130065	Horizontally Transferred (high confidence)
AZOLI_p10903	Horizontally Transferred (medium confidence)	AZOBR_p130066	Horizontally Transferred (high confidence)
AZOLI_p10904	Horizontally Transferred (low confidence)	AZOBR_p130067	Ancestral (high confidence)
AZOLI_p10905	Unassigned	AZOBR_p130068	Ancestral (high confidence)
AZOLI_p10906	Unassigned	AZOBR_p130069	Horizontally Transferred (high confidence)

AZOLI_p10907	Unassigned	AZOBR_p130070	Horizontally Transferred (high confidence)
AZOLI_p10908	Unassigned	AZOBR_p130071	Horizontally Transferred (high confidence)
AZOLI_p10909	Horizontally Transferred (high confidence)	AZOBR_p130072	Horizontally Transferred (medium confidence)
AZOLI_p10910	Horizontally Transferred (high confidence)	AZOBR_p130073	Horizontally Transferred (high confidence)
AZOLI_p10911	Horizontally Transferred (high confidence)	AZOBR_p130074	Horizontally Transferred (high confidence)
AZOLI_p10912	Horizontally Transferred (high confidence)	AZOBR_p130075	Horizontally Transferred (high confidence)
AZOLI_p10914	Horizontally Transferred (high confidence)	AZOBR_p130076	Horizontally Transferred (high confidence)
AZOLI_p10915	Horizontally Transferred (high confidence)	AZOBR_p130077	Unassigned
AZOLI_p10916	Horizontally Transferred (high confidence)	AZOBR_p130078	Horizontally Transferred (high confidence)
AZOLI_p10917	Horizontally Transferred (high confidence)	AZOBR_p130079	Ancestral (low confidence)
AZOLI_p10918	Ancestral (low confidence)	AZOBR_p130080	Ancestral (high confidence)
AZOLI_p10919	Ancestral (low confidence)	AZOBR_p130081	Horizontally Transferred (high confidence)
AZOLI_p10920	Horizontally Transferred (high confidence)	AZOBR_p130082	Horizontally Transferred (high confidence)
AZOLI_p10921	Horizontally Transferred (high confidence)	AZOBR_p130083	Ancestral (low confidence)
AZOLI_p10922	Horizontally Transferred (high confidence)	AZOBR_p130084	Unassigned
AZOLI_p10923	Horizontally Transferred (low confidence)	AZOBR_p130085	Unassigned
AZOLI_p10924	Horizontally Transferred (high confidence)	AZOBR_p130086	Unassigned
AZOLI_p10925	Horizontally Transferred (high confidence)	AZOBR_p130087	Unassigned
AZOLI_p10926	Horizontally Transferred (medium confidence)	AZOBR_p130088	Unassigned
AZOLI_p10927	Horizontally Transferred (medium confidence)	AZOBR_p130089	Unassigned
AZOLI_p10928	Horizontally Transferred (medium confidence)	AZOBR_p130090	Unassigned
AZOLI_p10929	Horizontally Transferred (medium confidence)	AZOBR_p130091	Unassigned
AZOLI_p10930	Horizontally Transferred (medium confidence)	AZOBR_p130092	Horizontally Transferred (high confidence)
AZOLI_p10931	Horizontally Transferred (high confidence)	AZOBR_p130093	Unassigned
AZOLI_p10932	Horizontally Transferred (high confidence)	AZOBR_p130094	Unassigned
AZOLI_p10933	Horizontally Transferred (high confidence)	AZOBR_p130095	Ancestral (medium confidence)
AZOLI_p10934	Horizontally Transferred (high confidence)	AZOBR_p130096	Unassigned
AZOLI_p10935	Horizontally Transferred (high confidence)	AZOBR_p130097	Horizontally Transferred (medium confidence)
AZOLI_p10936	Horizontally Transferred (high confidence)	AZOBR_p130098	Horizontally Transferred (high confidence)
AZOLI_p10937	Horizontally Transferred (high confidence)	AZOBR_p130099	Ancestral (low confidence)
AZOLI_p10938	Horizontally Transferred (high confidence)	AZOBR_p130100	Horizontally Transferred (high confidence)
AZOLI_p10939	Horizontally Transferred (medium confidence)	AZOBR_p130101	Horizontally Transferred (high confidence)
AZOLI_p10940	Horizontally Transferred (high confidence)	AZOBR_p130102	Horizontally Transferred (high confidence)
AZOLI_p10941	Horizontally Transferred (high confidence)	AZOBR_p130103	Horizontally Transferred (high confidence)
AZOLI_p10942	Horizontally Transferred (high confidence)	AZOBR_p130104	Horizontally Transferred (high confidence)
AZOLI_p10943	Ancestral (low confidence)	AZOBR_p130105	Unassigned
AZOLI_p10944	Ancestral (low confidence)	AZOBR_p130106	Horizontally Transferred (high confidence)
AZOLI_p10945	Unassigned	AZOBR_p130107	Unassigned
AZOLI_p10946	Ancestral (low confidence)	AZOBR_p130108	Ancestral (medium confidence)
AZOLI_p10948	Horizontally Transferred (low confidence)	AZOBR_p130109	Ancestral (low confidence)
AZOLI_p10949	Ancestral (low confidence)	AZOBR_p130110	Horizontally Transferred (medium confidence)
AZOLI_p10950	Horizontally Transferred (low confidence)	AZOBR_p130111	Unassigned
AZOLI_p10951	Horizontally Transferred (medium confidence)	AZOBR_p130112	Unassigned
AZOLI_p10952	Horizontally Transferred (low confidence)	AZOBR_p130113	Horizontally Transferred (high confidence)
AZOLI_p10953	Ancestral (low confidence)	AZOBR_p130114	Ancestral (high confidence)
AZOLI_p10954	Horizontally Transferred (low confidence)	AZOBR_p130115	Ancestral (medium confidence)
AZOLI_p10955	Ancestral (low confidence)	AZOBR_p130116	Unassigned
AZOLI_p10956	Ancestral (low confidence)	AZOBR_p130117	Ancestral (medium confidence)
AZOLI_p10957	Ancestral (low confidence)	AZOBR_p130118	Horizontally Transferred (high confidence)
AZOLI_p10958	Horizontally Transferred (low confidence)	AZOBR_p130119	Horizontally Transferred (high confidence)
AZOLI_p10961	Horizontally Transferred (high confidence)	AZOBR_p130120	Unassigned
AZOLI_p10962	Unassigned	AZOBR_p130121	Ancestral (low confidence)
AZOLI_p10963	Horizontally Transferred (high confidence)	AZOBR_p130122	Ancestral (medium confidence)
AZOLI_p10965	Horizontally Transferred (high confidence)	AZOBR_p130123	Ancestral (high confidence)
AZOLI_p10966	Ancestral (low confidence)	AZOBR_p130124	Ancestral (high confidence)
AZOLI_p10967	Horizontally Transferred (high confidence)	AZOBR_p130125	Unassigned
AZOLI_p10968	Horizontally Transferred (high confidence)	AZOBR_p130126	Horizontally Transferred (high confidence)
AZOLI_p10971	Ancestral (low confidence)	AZOBR_p130127	Horizontally Transferred (high confidence)
AZOLI_p10972	Horizontally Transferred (high confidence)	AZOBR_p130128	Unassigned
AZOLI_p10973	Unassigned	AZOBR_p130129	Horizontally Transferred (high confidence)
AZOLI_p10974	Ancestral (medium confidence)	AZOBR_p130130	Horizontally Transferred (high confidence)
AZOLI_p10975	Horizontally Transferred (high confidence)	AZOBR_p130131	Horizontally Transferred (high confidence)
AZOLI_p10976	Ancestral (low confidence)	AZOBR_p130132	Horizontally Transferred (high confidence)
AZOLI_p10977	Horizontally Transferred (high confidence)	AZOBR_p130133	Ancestral (high confidence)
AZOLI_p10978	Horizontally Transferred (high confidence)	AZOBR_p130134	Ancestral (high confidence)
AZOLI_p10980	Unassigned	AZOBR_p130135	Ancestral (high confidence)
AZOLI_p10981	Horizontally Transferred (low confidence)	AZOBR_p130136	Ancestral (low confidence)
AZOLI_p10983	Horizontally Transferred (high confidence)	AZOBR_p130137	Ancestral (medium confidence)
AZOLI_p10984	Horizontally Transferred (low confidence)	AZOBR_p130138	Ancestral (high confidence)
AZOLI_p10985	Horizontally Transferred (low confidence)	AZOBR_p130139	Ancestral (medium confidence)
AZOLI_p10986	Horizontally Transferred (low confidence)	AZOBR_p130140	Ancestral (medium confidence)
AZOLI_p10987	Horizontally Transferred (low confidence)	AZOBR_p130141	Ancestral (high confidence)
AZOLI_p10988	Horizontally Transferred (medium confidence)	AZOBR_p130142	Horizontally Transferred (high confidence)
AZOLI_p10989	Horizontally Transferred (high confidence)	AZOBR_p130143	Ancestral (medium confidence)
AZOLI_p10990	Horizontally Transferred (medium confidence)	AZOBR_p130144	Ancestral (high confidence)

AZOLI_p10991	Ancestral (low confidence)	AZOBR_p130145	Ancestral (high confidence)
AZOLI_p10995	Ancestral (low confidence)	AZOBR_p130146	Ancestral (high confidence)
AZOLI_p10996	Ancestral (medium confidence)	AZOBR_p130147	Ancestral (high confidence)
AZOLI_p10997	Ancestral (low confidence)	AZOBR_p130148	Ancestral (high confidence)
AZOLI_p10998	Ancestral (medium confidence)	AZOBR_p130149	Ancestral (high confidence)
AZOLI_p10999	Ancestral (medium confidence)	AZOBR_p130150	Ancestral (medium confidence)
AZOLI_p11000	Ancestral (medium confidence)	AZOBR_p130151	Horizontally Transferred (high confidence)
AZOLI_p11001	Horizontally Transferred (high confidence)	AZOBR_p130152	Horizontally Transferred (high confidence)
AZOLI_p11002	Horizontally Transferred (high confidence)	AZOBR_p130153	Horizontally Transferred (low confidence)
AZOLI_p11003	Horizontally Transferred (high confidence)	AZOBR_p130154	Ancestral (medium confidence)
AZOLI_p11004	Horizontally Transferred (high confidence)	AZOBR_p130155	Ancestral (medium confidence)
AZOLI_p11007	Horizontally Transferred (high confidence)	AZOBR_p130156	Ancestral (high confidence)
AZOLI_p11009	Horizontally Transferred (high confidence)	AZOBR_p130157	Ancestral (low confidence)
AZOLI_p11010	Horizontally Transferred (high confidence)	AZOBR_p130158	Ancestral (high confidence)
AZOLI_p11011	Unassigned	AZOBR_p130159	Ancestral (high confidence)
AZOLI_p11013	Horizontally Transferred (high confidence)	AZOBR_p130160	Ancestral (medium confidence)
AZOLI_p11014	Unassigned	AZOBR_p130161	Horizontally Transferred (high confidence)
AZOLI_p11017	Unassigned	AZOBR_p130162	Horizontally Transferred (high confidence)
AZOLI_p11018	Unassigned	AZOBR_p130163	Ancestral (medium confidence)
AZOLI_p11021	Ancestral (low confidence)	AZOBR_p130164	Ancestral (high confidence)
AZOLI_p11022	Ancestral (low confidence)	AZOBR_p130165	Unassigned
AZOLI_p11023	Unassigned	AZOBR_p130166	Unassigned
AZOLI_p11024	Horizontally Transferred (high confidence)	AZOBR_p130167	Ancestral (low confidence)
AZOLI_p11025	Horizontally Transferred (high confidence)	AZOBR_p130168	Ancestral (high confidence)
AZOLI_p11026	Horizontally Transferred (high confidence)	AZOBR_p130169	Ancestral (high confidence)
AZOLI_p11027	Horizontally Transferred (high confidence)	AZOBR_p130170	Ancestral (high confidence)
AZOLI_p11028	Unassigned	AZOBR_p130171	Ancestral (high confidence)
AZOLI_p11029	Ancestral (low confidence)	AZOBR_p130172	Unassigned
AZOLI_p11030	Ancestral (low confidence)	AZOBR_p130173	Ancestral (medium confidence)
AZOLI_p11031	Ancestral (low confidence)	AZOBR_p130174	Ancestral (low confidence)
AZOLI_p11032	Ancestral (low confidence)	AZOBR_p130175	Unassigned
AZOLI_p11033	Horizontally Transferred (high confidence)	AZOBR_p130176	Horizontally Transferred (high confidence)
AZOLI_p11034	Horizontally Transferred (medium confidence)	AZOBR_p130177	Ancestral (medium confidence)
AZOLI_p11035	Horizontally Transferred (low confidence)	AZOBR_p130178	Unassigned
AZOLI_p11036	Horizontally Transferred (high confidence)	AZOBR_p130179	Ancestral (high confidence)
AZOLI_p11037	Horizontally Transferred (high confidence)	AZOBR_p130180	Ancestral (high confidence)
AZOLI_p11040	Horizontally Transferred (high confidence)	AZOBR_p130181	Ancestral (high confidence)
AZOLI_p11042	Horizontally Transferred (high confidence)	AZOBR_p130182	Ancestral (high confidence)
AZOLI_p11043	Horizontally Transferred (high confidence)	AZOBR_p130183	Ancestral (high confidence)
AZOLI_p11044	Horizontally Transferred (high confidence)	AZOBR_p130184	Ancestral (medium confidence)
AZOLI_p11045	Unassigned	AZOBR_p130185	Horizontally Transferred (high confidence)
AZOLI_p11046	Ancestral (low confidence)	AZOBR_p130186	Ancestral (high confidence)
AZOLI_p11047	Ancestral (low confidence)	AZOBR_p130187	Unassigned
AZOLI_p11048	Ancestral (low confidence)	AZOBR_p130188	Unassigned
AZOLI_p20005	Horizontally Transferred (high confidence)	AZOBR_p130189	Unassigned
AZOLI_p20006	Horizontally Transferred (high confidence)	AZOBR_p130190	Horizontally Transferred (high confidence)
AZOLI_p20007	Horizontally Transferred (high confidence)	AZOBR_p130191	Ancestral (high confidence)
AZOLI_p20008	Horizontally Transferred (high confidence)	AZOBR_p130192	Ancestral (high confidence)
AZOLI_p20009	Horizontally Transferred (high confidence)	AZOBR_p130193	Unassigned
AZOLI_p20010	Ancestral (low confidence)	AZOBR_p130194	Ancestral (high confidence)
AZOLI_p20023	Ancestral (low confidence)	AZOBR_p130195	Ancestral (medium confidence)
AZOLI_p20024	Ancestral (medium confidence)	AZOBR_p130196	Ancestral (medium confidence)
AZOLI_p20026	Ancestral (low confidence)	AZOBR_p130197	Ancestral (medium confidence)
AZOLI_p20027	Ancestral (low confidence)	AZOBR_p130198	Ancestral (high confidence)
AZOLI_p20028	Ancestral (low confidence)	AZOBR_p130199	Horizontally Transferred (high confidence)
AZOLI_p20029	Unassigned	AZOBR_p130200	Ancestral (low confidence)
AZOLI_p20030	Horizontally Transferred (low confidence)	AZOBR_p130201	Horizontally Transferred (high confidence)
AZOLI_p20031	Unassigned	AZOBR_p130202	Horizontally Transferred (high confidence)
AZOLI_p20032	Ancestral (low confidence)	AZOBR_p130203	Horizontally Transferred (high confidence)
AZOLI_p20033	Unassigned	AZOBR_p130204	Horizontally Transferred (high confidence)
AZOLI_p20036	Unassigned	AZOBR_p130205	Unassigned
AZOLI_p20038	Ancestral (low confidence)	AZOBR_p130206	Ancestral (low confidence)
AZOLI_p20039	Ancestral (low confidence)	AZOBR_p130207	Ancestral (low confidence)
AZOLI_p20040	Unassigned	AZOBR_p130208	Ancestral (low confidence)
AZOLI_p20041	Ancestral (low confidence)	AZOBR_p130209	Ancestral (low confidence)
AZOLI_p20042	Horizontally Transferred (low confidence)	AZOBR_p130210	Horizontally Transferred (high confidence)
AZOLI_p20043	Horizontally Transferred (medium confidence)	AZOBR_p130211	Unassigned
AZOLI_p20044	Unassigned	AZOBR_p130212	Horizontally Transferred (high confidence)
AZOLI_p20045	Horizontally Transferred (high confidence)	AZOBR_p130213	Ancestral (low confidence)
AZOLI_p20046	Ancestral (high confidence)	AZOBR_p130214	Horizontally Transferred (high confidence)
AZOLI_p20047	Horizontally Transferred (medium confidence)	AZOBR_p130215	Horizontally Transferred (high confidence)
AZOLI_p20048	Horizontally Transferred (high confidence)	AZOBR_p130216	Horizontally Transferred (high confidence)
AZOLI_p20049	Horizontally Transferred (low confidence)	AZOBR_p130217	Ancestral (low confidence)
AZOLI_p20050	Horizontally Transferred (low confidence)	AZOBR_p130218	Horizontally Transferred (high confidence)
AZOLI_p20052	Unassigned	AZOBR_p130219	Horizontally Transferred (medium confidence)

AZOLI_p20053	Horizontally Transferred (high confidence)	AZOBR_p130220	Ancestral (low confidence)
AZOLI_p20054	Unassigned	AZOBR_p130221	Horizontally Transferred (high confidence)
AZOLI_p20055	Ancestral (low confidence)	AZOBR_p130222	Unassigned
AZOLI_p20056	Unassigned	AZOBR_p140001	Unassigned
AZOLI_p20057	Horizontally Transferred (high confidence)	AZOBR_p140002	Horizontally Transferred (high confidence)
AZOLI_p20058	Horizontally Transferred (high confidence)	AZOBR_p140003	Unassigned
AZOLI_p20059	Horizontally Transferred (medium confidence)	AZOBR_p140004	Unassigned
AZOLI_p20060	Horizontally Transferred (high confidence)	AZOBR_p140005	Horizontally Transferred (high confidence)
AZOLI_p20061	Horizontally Transferred (low confidence)	AZOBR_p140006	Ancestral (medium confidence)
AZOLI_p20062	Horizontally Transferred (medium confidence)	AZOBR_p140007	Unassigned
AZOLI_p20063	Unassigned	AZOBR_p140008	Horizontally Transferred (high confidence)
AZOLI_p20064	Unassigned	AZOBR_p140009	Ancestral (low confidence)
AZOLI_p20065	Horizontally Transferred (medium confidence)	AZOBR_p140010	Unassigned
AZOLI_p20066	Horizontally Transferred (high confidence)	AZOBR_p140011	Ancestral (medium confidence)
AZOLI_p20067	Unassigned	AZOBR_p140012	Unassigned
AZOLI_p20068	Horizontally Transferred (low confidence)	AZOBR_p140013	Ancestral (medium confidence)
AZOLI_p20069	Horizontally Transferred (high confidence)	AZOBR_p140014	Horizontally Transferred (high confidence)
AZOLI_p20070	Horizontally Transferred (high confidence)	AZOBR_p140015	Ancestral (low confidence)
AZOLI_p20071	Horizontally Transferred (high confidence)	AZOBR_p140016	Unassigned
AZOLI_p20072	Horizontally Transferred (medium confidence)	AZOBR_p140017	Ancestral (low confidence)
AZOLI_p20073	Unassigned	AZOBR_p140018	Unassigned
AZOLI_p20074	Horizontally Transferred (high confidence)	AZOBR_p140019	Ancestral (low confidence)
AZOLI_p20075	Horizontally Transferred (high confidence)	AZOBR_p140020	Horizontally Transferred (high confidence)
AZOLI_p20076	Horizontally Transferred (high confidence)	AZOBR_p140021	Unassigned
AZOLI_p20077	Horizontally Transferred (high confidence)	AZOBR_p140022	Horizontally Transferred (medium confidence)
AZOLI_p20078	Unassigned	AZOBR_p140023	Ancestral (medium confidence)
AZOLI_p20079	Horizontally Transferred (high confidence)	AZOBR_p140024	Unassigned
AZOLI_p20085	Horizontally Transferred (high confidence)	AZOBR_p140025	Unassigned
AZOLI_p20086	Unassigned	AZOBR_p140026	Ancestral (medium confidence)
AZOLI_p20087	Horizontally Transferred (high confidence)	AZOBR_p140027	Ancestral (low confidence)
AZOLI_p20088	Horizontally Transferred (medium confidence)	AZOBR_p140028	Ancestral (medium confidence)
AZOLI_p20089	Horizontally Transferred (high confidence)	AZOBR_p140029	Horizontally Transferred (medium confidence)
AZOLI_p20090	Horizontally Transferred (high confidence)	AZOBR_p140030	Ancestral (low confidence)
AZOLI_p20091	Horizontally Transferred (high confidence)	AZOBR_p140031	Horizontally Transferred (low confidence)
AZOLI_p20092	Unassigned	AZOBR_p140032	Horizontally Transferred (high confidence)
AZOLI_p20093	Horizontally Transferred (medium confidence)	AZOBR_p140033	Horizontally Transferred (high confidence)
AZOLI_p20094	Ancestral (low confidence)	AZOBR_p140034	Horizontally Transferred (high confidence)
AZOLI_p20095	Horizontally Transferred (low confidence)	AZOBR_p140035	Horizontally Transferred (high confidence)
AZOLI_p20099	Ancestral (medium confidence)	AZOBR_p140036	Horizontally Transferred (medium confidence)
AZOLI_p20100	Ancestral (low confidence)	AZOBR_p140037	Horizontally Transferred (medium confidence)
AZOLI_p20101	Ancestral (low confidence)	AZOBR_p140038	Ancestral (medium confidence)
AZOLI_p20102	Ancestral (low confidence)	AZOBR_p140039	Horizontally Transferred (medium confidence)
AZOLI_p20103	Horizontally Transferred (high confidence)	AZOBR_p140040	Horizontally Transferred (medium confidence)
AZOLI_p20104	Unassigned	AZOBR_p140041	Ancestral (medium confidence)
AZOLI_p20105	Horizontally Transferred (high confidence)	AZOBR_p140042	Horizontally Transferred (medium confidence)
AZOLI_p20106	Unassigned	AZOBR_p140043	Horizontally Transferred (high confidence)
AZOLI_p20109	Unassigned	AZOBR_p140044	Unassigned
AZOLI_p20110	Ancestral (low confidence)	AZOBR_p140045	Horizontally Transferred (medium confidence)
AZOLI_p20111	Unassigned	AZOBR_p140046	Horizontally Transferred (high confidence)
AZOLI_p20112	Ancestral (medium confidence)	AZOBR_p140047	Horizontally Transferred (medium confidence)
AZOLI_p20113	Unassigned	AZOBR_p140048	Horizontally Transferred (high confidence)
AZOLI_p20114	Ancestral (low confidence)	AZOBR_p140049	Unassigned
AZOLI_p20115	Ancestral (low confidence)	AZOBR_p140050	Ancestral (medium confidence)
AZOLI_p20116	Horizontally Transferred (low confidence)	AZOBR_p140051	Ancestral (medium confidence)
AZOLI_p20117	Ancestral (low confidence)	AZOBR_p140052	Ancestral (low confidence)
AZOLI_p20119	Ancestral (low confidence)	AZOBR_p140053	Unassigned
AZOLI_p20120	Ancestral (medium confidence)	AZOBR_p140054	Unassigned
AZOLI_p20121	Ancestral (medium confidence)	AZOBR_p140055	Ancestral (medium confidence)
AZOLI_p20122	Horizontally Transferred (high confidence)	AZOBR_p140056	Horizontally Transferred (medium confidence)
AZOLI_p20123	Ancestral (high confidence)	AZOBR_p140057	Ancestral (medium confidence)
AZOLI_p20126	Unassigned	AZOBR_p140058	Ancestral (high confidence)
AZOLI_p20127	Horizontally Transferred (high confidence)	AZOBR_p140059	Ancestral (high confidence)
AZOLI_p20129	Horizontally Transferred (low confidence)	AZOBR_p140060	Ancestral (medium confidence)
AZOLI_p20132	Unassigned	AZOBR_p140061	Ancestral (medium confidence)
AZOLI_p20134	Unassigned	AZOBR_p140062	Ancestral (medium confidence)
AZOLI_p20135	Horizontally Transferred (high confidence)	AZOBR_p140063	Ancestral (medium confidence)
AZOLI_p20136	Horizontally Transferred (high confidence)	AZOBR_p140064	Horizontally Transferred (high confidence)
AZOLI_p20137	Horizontally Transferred (high confidence)	AZOBR_p140065	Unassigned
AZOLI_p20138	Ancestral (low confidence)	AZOBR_p140066	Ancestral (low confidence)
AZOLI_p20139	Horizontally Transferred (low confidence)	AZOBR_p140067	Ancestral (medium confidence)
AZOLI_p20140	Horizontally Transferred (high confidence)	AZOBR_p140068	Ancestral (low confidence)
AZOLI_p20143	Ancestral (low confidence)	AZOBR_p140069	Unassigned
AZOLI_p20144	Ancestral (low confidence)	AZOBR_p140070	Horizontally Transferred (high confidence)
AZOLI_p20145	Horizontally Transferred (high confidence)	AZOBR_p140071	Ancestral (medium confidence)
AZOLI_p20146	Horizontally Transferred (high confidence)	AZOBR_p140072	Ancestral (medium confidence)

AZOLI_p20147	Horizontally Transferred (high confidence)	AZOBR_p140073	Unassigned
AZOLI_p20150	Horizontally Transferred (high confidence)	AZOBR_p140074	Ancestral (medium confidence)
AZOLI_p20151	Horizontally Transferred (high confidence)	AZOBR_p140075	Unassigned
AZOLI_p20152	Unassigned	AZOBR_p140076	Horizontally Transferred (high confidence)
AZOLI_p20153	Ancestral (low confidence)	AZOBR_p140077	Ancestral (medium confidence)
AZOLI_p20154	Horizontally Transferred (medium confidence)	AZOBR_p140078	Ancestral (medium confidence)
AZOLI_p20155	Horizontally Transferred (low confidence)	AZOBR_p140079	Ancestral (medium confidence)
AZOLI_p20156	Ancestral (low confidence)	AZOBR_p140080	Ancestral (high confidence)
AZOLI_p20157	Horizontally Transferred (high confidence)	AZOBR_p140081	Ancestral (high confidence)
AZOLI_p20158	Ancestral (low confidence)	AZOBR_p140082	Ancestral (medium confidence)
AZOLI_p20159	Horizontally Transferred (high confidence)	AZOBR_p140083	Ancestral (high confidence)
AZOLI_p20160	Horizontally Transferred (high confidence)	AZOBR_p140084	Ancestral (low confidence)
AZOLI_p20161	Horizontally Transferred (high confidence)	AZOBR_p140085	Ancestral (medium confidence)
AZOLI_p20162	Horizontally Transferred (high confidence)	AZOBR_p140086	Ancestral (medium confidence)
AZOLI_p20163	Horizontally Transferred (high confidence)	AZOBR_p140087	Horizontally Transferred (high confidence)
AZOLI_p20164	Horizontally Transferred (high confidence)	AZOBR_p140088	Ancestral (medium confidence)
AZOLI_p20165	Horizontally Transferred (high confidence)	AZOBR_p140089	Ancestral (medium confidence)
AZOLI_p20166	Horizontally Transferred (high confidence)	AZOBR_p140090	Unassigned
AZOLI_p20168	Ancestral (low confidence)	AZOBR_p140091	Ancestral (medium confidence)
AZOLI_p20169	Ancestral (low confidence)	AZOBR_p140092	Ancestral (high confidence)
AZOLI_p20171	Horizontally Transferred (high confidence)	AZOBR_p140093	Ancestral (high confidence)
AZOLI_p20172	Horizontally Transferred (high confidence)	AZOBR_p140094	Ancestral (medium confidence)
AZOLI_p20173	Ancestral (low confidence)	AZOBR_p140095	Ancestral (low confidence)
AZOLI_p20175	Horizontally Transferred (low confidence)	AZOBR_p140096	Ancestral (low confidence)
AZOLI_p20176	Horizontally Transferred (high confidence)	AZOBR_p140097	Ancestral (low confidence)
AZOLI_p20177	Horizontally Transferred (high confidence)	AZOBR_p140098	Unassigned
AZOLI_p20178	Horizontally Transferred (high confidence)	AZOBR_p140099	Ancestral (medium confidence)
AZOLI_p20179	Horizontally Transferred (low confidence)	AZOBR_p140100	Ancestral (medium confidence)
AZOLI_p20180	Horizontally Transferred (high confidence)	AZOBR_p140101	Ancestral (low confidence)
AZOLI_p20181	Horizontally Transferred (high confidence)	AZOBR_p140102	Unassigned
AZOLI_p20183	Ancestral (low confidence)	AZOBR_p150001	Ancestral (low confidence)
AZOLI_p20184	Horizontally Transferred (low confidence)	AZOBR_p150002	Unassigned
AZOLI_p20185	Ancestral (low confidence)	AZOBR_p150003	Ancestral (low confidence)
AZOLI_p20186	Ancestral (medium confidence)	AZOBR_p150004	Ancestral (low confidence)
AZOLI_p20187	Ancestral (high confidence)	AZOBR_p150005	Horizontally Transferred (high confidence)
AZOLI_p20188	Ancestral (low confidence)	AZOBR_p150006	Horizontally Transferred (high confidence)
AZOLI_p20189	Unassigned	AZOBR_p150007	Ancestral (low confidence)
AZOLI_p20191	Horizontally Transferred (high confidence)	AZOBR_p150008	Horizontally Transferred (high confidence)
AZOLI_p20192	Horizontally Transferred (high confidence)	AZOBR_p150009	Ancestral (medium confidence)
AZOLI_p20193	Horizontally Transferred (high confidence)	AZOBR_p150010	Ancestral (high confidence)
AZOLI_p20194	Unassigned	AZOBR_p150011	Horizontally Transferred (low confidence)
AZOLI_p20196	Horizontally Transferred (high confidence)	AZOBR_p150012	Unassigned
AZOLI_p20197	Horizontally Transferred (high confidence)	AZOBR_p150013	Horizontally Transferred (high confidence)
AZOLI_p20198	Horizontally Transferred (high confidence)	AZOBR_p150014	Unassigned
AZOLI_p20199	Unassigned	AZOBR_p150015	Horizontally Transferred (high confidence)
AZOLI_p20200	Horizontally Transferred (high confidence)	AZOBR_p150016	Unassigned
AZOLI_p20201	Ancestral (low confidence)	AZOBR_p150017	Ancestral (medium confidence)
AZOLI_p20202	Unassigned	AZOBR_p150018	Unassigned
AZOLI_p20203	Ancestral (low confidence)	AZOBR_p150019	Horizontally Transferred (high confidence)
AZOLI_p20204	Ancestral (low confidence)	AZOBR_p150020	Unassigned
AZOLI_p20205	Horizontally Transferred (high confidence)	AZOBR_p150021	Horizontally Transferred (high confidence)
AZOLI_p20206	Ancestral (medium confidence)	AZOBR_p150022	Horizontally Transferred (low confidence)
AZOLI_p20207	Horizontally Transferred (high confidence)	AZOBR_p150023	Ancestral (low confidence)
AZOLI_p20209	Ancestral (low confidence)	AZOBR_p150024	Horizontally Transferred (high confidence)
AZOLI_p20210	Unassigned	AZOBR_p150025	Horizontally Transferred (high confidence)
AZOLI_p20211	Unassigned	AZOBR_p150026	Ancestral (low confidence)
AZOLI_p20212	Horizontally Transferred (high confidence)	AZOBR_p150027	Horizontally Transferred (high confidence)
AZOLI_p20215	Ancestral (low confidence)	AZOBR_p150028	Ancestral (low confidence)
AZOLI_p20216	Horizontally Transferred (high confidence)	AZOBR_p150029	Ancestral (low confidence)
AZOLI_p20218	Horizontally Transferred (high confidence)	AZOBR_p150030	Horizontally Transferred (high confidence)
AZOLI_p20219	Horizontally Transferred (high confidence)	AZOBR_p150031	Unassigned
AZOLI_p20220	Horizontally Transferred (high confidence)	AZOBR_p150032	Ancestral (low confidence)
AZOLI_p20221	Horizontally Transferred (medium confidence)	AZOBR_p150033	Ancestral (low confidence)
AZOLI_p20222	Horizontally Transferred (high confidence)	AZOBR_p150034	Unassigned
AZOLI_p20223	Horizontally Transferred (high confidence)	AZOBR_p150035	Horizontally Transferred (high confidence)
AZOLI_p20224	Ancestral (medium confidence)	AZOBR_p150036	Ancestral (low confidence)
AZOLI_p20225	Horizontally Transferred (high confidence)	AZOBR_p150037	Ancestral (low confidence)
AZOLI_p20226	Horizontally Transferred (high confidence)	AZOBR_p150038	Ancestral (medium confidence)
AZOLI_p20228	Ancestral (low confidence)	AZOBR_p150039	Ancestral (medium confidence)
AZOLI_p20229	Unassigned	AZOBR_p150040	Horizontally Transferred (high confidence)
AZOLI_p20230	Horizontally Transferred (medium confidence)	AZOBR_p150041	Horizontally Transferred (high confidence)
AZOLI_p20231	Horizontally Transferred (medium confidence)	AZOBR_p150042	Ancestral (medium confidence)
AZOLI_p20232	Ancestral (medium confidence)	AZOBR_p150043	Unassigned
AZOLI_p20233	Ancestral (low confidence)	AZOBR_p150044	Ancestral (medium confidence)
AZOLI_p20234	Horizontally Transferred (high confidence)	AZOBR_p150045	Ancestral (low confidence)

AZOLI_p20235	Horizontally Transferred (high confidence)	AZOBR_p150046	Ancestral (low confidence)
AZOLI_p20236	Horizontally Transferred (high confidence)	AZOBR_p150047	Horizontally Transferred (high confidence)
AZOLI_p20237	Horizontally Transferred (high confidence)	AZOBR_p150048	Unassigned
AZOLI_p20238	Unassigned	AZOBR_p150049	Unassigned
AZOLI_p20239	Ancestral (low confidence)	AZOBR_p150050	Ancestral (low confidence)
AZOLI_p20240	Horizontally Transferred (low confidence)	AZOBR_p150051	Ancestral (low confidence)
AZOLI_p20241	Horizontally Transferred (high confidence)	AZOBR_p150052	Horizontally Transferred (high confidence)
AZOLI_p20242	Horizontally Transferred (high confidence)	AZOBR_p150053	Ancestral (high confidence)
AZOLI_p20243	Horizontally Transferred (high confidence)	AZOBR_p150054	Ancestral (medium confidence)
AZOLI_p20244	Ancestral (low confidence)	AZOBR_p150055	Ancestral (medium confidence)
AZOLI_p20245	Ancestral (low confidence)	AZOBR_p150056	Unassigned
AZOLI_p20247	Horizontally Transferred (high confidence)	AZOBR_p150057	Horizontally Transferred (medium confidence)
AZOLI_p20248	Horizontally Transferred (high confidence)	AZOBR_p150058	Horizontally Transferred (high confidence)
AZOLI_p20249	Horizontally Transferred (high confidence)	AZOBR_p150059	Horizontally Transferred (high confidence)
AZOLI_p20250	Horizontally Transferred (low confidence)	AZOBR_p150060	Unassigned
AZOLI_p20251	Horizontally Transferred (low confidence)	AZOBR_p150061	Ancestral (high confidence)
AZOLI_p20252	Horizontally Transferred (low confidence)	AZOBR_p150062	Ancestral (low confidence)
AZOLI_p20253	Horizontally Transferred (high confidence)	AZOBR_p150063	Horizontally Transferred (high confidence)
AZOLI_p20254	Horizontally Transferred (low confidence)	AZOBR_p150064	Ancestral (medium confidence)
AZOLI_p20255	Ancestral (low confidence)	AZOBR_p150065	Ancestral (medium confidence)
AZOLI_p20256	Horizontally Transferred (high confidence)	AZOBR_p150066	Unassigned
AZOLI_p20257	Horizontally Transferred (high confidence)	AZOBR_p150067	Horizontally Transferred (high confidence)
AZOLI_p20258	Horizontally Transferred (high confidence)	AZOBR_p150068	Unassigned
AZOLI_p20259	Horizontally Transferred (high confidence)	AZOBR_p150069	Unassigned
AZOLI_p20260	Horizontally Transferred (high confidence)	AZOBR_p150070	Unassigned
AZOLI_p20261	Horizontally Transferred (high confidence)	AZOBR_p150071	Unassigned
AZOLI_p20262	Unassigned	AZOBR_p150072	Ancestral (medium confidence)
AZOLI_p20264	Horizontally Transferred (high confidence)	AZOBR_p150073	Ancestral (medium confidence)
AZOLI_p20265	Unassigned	AZOBR_p150074	Unassigned
AZOLI_p20266	Horizontally Transferred (high confidence)	AZOBR_p150075	Unassigned
AZOLI_p20268	Horizontally Transferred (high confidence)	AZOBR_p150076	Ancestral (medium confidence)
AZOLI_p20269	Horizontally Transferred (high confidence)	AZOBR_p150077	Ancestral (high confidence)
AZOLI_p20270	Horizontally Transferred (high confidence)	AZOBR_p150078	Ancestral (high confidence)
AZOLI_p20271	Unassigned	AZOBR_p150079	Ancestral (high confidence)
AZOLI_p20272	Horizontally Transferred (high confidence)	AZOBR_p150080	Ancestral (medium confidence)
AZOLI_p20273	Horizontally Transferred (high confidence)	AZOBR_p150081	Ancestral (medium confidence)
AZOLI_p20274	Horizontally Transferred (high confidence)	AZOBR_p150082	Ancestral (high confidence)
AZOLI_p20275	Horizontally Transferred (high confidence)	AZOBR_p150083	Ancestral (medium confidence)
AZOLI_p20276	Horizontally Transferred (high confidence)	AZOBR_p150084	Ancestral (high confidence)
AZOLI_p20277	Ancestral (medium confidence)	AZOBR_p150085	Horizontally Transferred (high confidence)
AZOLI_p20278	Ancestral (medium confidence)	AZOBR_p150086	Unassigned
AZOLI_p20280	Unassigned	AZOBR_p150087	Ancestral (medium confidence)
AZOLI_p20283	Ancestral (high confidence)	AZOBR_p150088	Horizontally Transferred (high confidence)
AZOLI_p20285	Horizontally Transferred (high confidence)	AZOBR_p150089	Horizontally Transferred (high confidence)
AZOLI_p20286	Horizontally Transferred (high confidence)	AZOBR_p150090	Ancestral (low confidence)
AZOLI_p20287	Horizontally Transferred (high confidence)	AZOBR_p150091	Horizontally Transferred (high confidence)
AZOLI_p20288	Horizontally Transferred (high confidence)	AZOBR_p150092	Horizontally Transferred (high confidence)
AZOLI_p20289	Horizontally Transferred (high confidence)	AZOBR_p150093	Horizontally Transferred (high confidence)
AZOLI_p20290	Horizontally Transferred (high confidence)	AZOBR_p150094	Ancestral (medium confidence)
AZOLI_p20291	Horizontally Transferred (high confidence)	AZOBR_p150095	Horizontally Transferred (medium confidence)
AZOLI_p20292	Horizontally Transferred (high confidence)	AZOBR_p150096	Ancestral (low confidence)
AZOLI_p20293	Horizontally Transferred (low confidence)	AZOBR_p150097	Ancestral (low confidence)
AZOLI_p20294	Horizontally Transferred (high confidence)	AZOBR_p150098	Horizontally Transferred (high confidence)
AZOLI_p20295	Horizontally Transferred (high confidence)	AZOBR_p150099	Horizontally Transferred (high confidence)
AZOLI_p20296	Horizontally Transferred (high confidence)	AZOBR_p160001	Ancestral (medium confidence)
AZOLI_p20304	Horizontally Transferred (high confidence)	AZOBR_p160002	Ancestral (low confidence)
AZOLI_p20305	Horizontally Transferred (high confidence)	AZOBR_p160003	Unassigned
AZOLI_p20306	Horizontally Transferred (high confidence)	AZOBR_p160004	Ancestral (medium confidence)
AZOLI_p20307	Horizontally Transferred (high confidence)	AZOBR_p160005	Unassigned
AZOLI_p20308	Horizontally Transferred (high confidence)	AZOBR_p160006	Horizontally Transferred (medium confidence)
AZOLI_p20309	Horizontally Transferred (high confidence)	AZOBR_p160007	Ancestral (low confidence)
AZOLI_p20310	Horizontally Transferred (high confidence)	AZOBR_p160008	Horizontally Transferred (medium confidence)
AZOLI_p20311	Horizontally Transferred (high confidence)	AZOBR_p160009	Unassigned
AZOLI_p20312	Horizontally Transferred (high confidence)	AZOBR_p170001	Ancestral (medium confidence)
AZOLI_p20316	Horizontally Transferred (high confidence)	AZOBR_p170002	Unassigned
AZOLI_p20317	Horizontally Transferred (high confidence)	AZOBR_p170003	Ancestral (low confidence)
AZOLI_p20318	Horizontally Transferred (high confidence)	AZOBR_p170004	Ancestral (low confidence)
AZOLI_p20319	Horizontally Transferred (high confidence)	AZOBR_p170005	Unassigned
AZOLI_p20320	Unassigned	AZOBR_p170006	Ancestral (medium confidence)
AZOLI_p20321	Horizontally Transferred (high confidence)	AZOBR_p170007	Horizontally Transferred (high confidence)
AZOLI_p20322	Horizontally Transferred (high confidence)	AZOBR_p170008	Ancestral (medium confidence)
AZOLI_p20323	Horizontally Transferred (high confidence)	AZOBR_p170009	Unassigned
AZOLI_p20324	Horizontally Transferred (high confidence)	AZOBR_p170010	Ancestral (high confidence)
AZOLI_p20325	Horizontally Transferred (high confidence)	AZOBR_p170011	Ancestral (medium confidence)
AZOLI_p20326	Horizontally Transferred (high confidence)	AZOBR_p170012	Ancestral (medium confidence)

AZOLI_p20327	Ancestral (medium confidence)	AZOBR_p170013	Horizontally Transferred (high confidence)
AZOLI_p20328	Ancestral (medium confidence)	AZOBR_p170014	Horizontally Transferred (high confidence)
AZOLI_p20329	Ancestral (high confidence)	AZOBR_p170015	Horizontally Transferred (high confidence)
AZOLI_p20330	Ancestral (high confidence)	AZOBR_p170016	Ancestral (low confidence)
AZOLI_p20331	Ancestral (high confidence)	AZOBR_p170017	Unassigned
AZOLI_p20332	Ancestral (medium confidence)	AZOBR_p170018	Unassigned
AZOLI_p20333	Ancestral (medium confidence)	AZOBR_p170019	Ancestral (low confidence)
AZOLI_p20334	Ancestral (high confidence)	AZOBR_p170020	Unassigned
AZOLI_p20335	Ancestral (high confidence)	AZOBR_p170021	Horizontally Transferred (high confidence)
AZOLI_p20336	Ancestral (high confidence)	AZOBR_p170022	Horizontally Transferred (high confidence)
AZOLI_p20337	Ancestral (high confidence)	AZOBR_p170023	Unassigned
AZOLI_p20338	Ancestral (medium confidence)	AZOBR_p170024	Horizontally Transferred (high confidence)
AZOLI_p20339	Ancestral (medium confidence)	AZOBR_p170025	Horizontally Transferred (high confidence)
AZOLI_p20340	Horizontally Transferred (high confidence)	AZOBR_p170026	Horizontally Transferred (high confidence)
AZOLI_p20341	Ancestral (medium confidence)	AZOBR_p170027	Horizontally Transferred (high confidence)
AZOLI_p20342	Ancestral (medium confidence)	AZOBR_p170028	Horizontally Transferred (high confidence)
AZOLI_p20343	Unassigned	AZOBR_p170029	Horizontally Transferred (high confidence)
AZOLI_p20344	Horizontally Transferred (high confidence)	AZOBR_p170030	Horizontally Transferred (high confidence)
AZOLI_p20345	Horizontally Transferred (high confidence)	AZOBR_p170031	Horizontally Transferred (high confidence)
AZOLI_p20346	Horizontally Transferred (high confidence)	AZOBR_p170032	Horizontally Transferred (high confidence)
AZOLI_p20348	Ancestral (low confidence)	AZOBR_p170033	Horizontally Transferred (high confidence)
AZOLI_p20349	Horizontally Transferred (high confidence)	AZOBR_p170034	Ancestral (low confidence)
AZOLI_p20350	Horizontally Transferred (high confidence)	AZOBR_p170035	Unassigned
AZOLI_p20351	Horizontally Transferred (high confidence)	AZOBR_p170036	Ancestral (low confidence)
AZOLI_p20352	Horizontally Transferred (high confidence)	AZOBR_p170037	Ancestral (high confidence)
AZOLI_p20353	Horizontally Transferred (high confidence)	AZOBR_p170038	Ancestral (medium confidence)
AZOLI_p20354	Horizontally Transferred (medium confidence)	AZOBR_p170039	Ancestral (high confidence)
AZOLI_p20355	Horizontally Transferred (high confidence)	AZOBR_p170040	Ancestral (high confidence)
AZOLI_p20356	Horizontally Transferred (high confidence)	AZOBR_p170041	Ancestral (high confidence)
AZOLI_p20357	Ancestral (low confidence)	AZOBR_p170042	Unassigned
AZOLI_p20358	Ancestral (low confidence)	AZOBR_p170043	Horizontally Transferred (medium confidence)
AZOLI_p20359	Ancestral (low confidence)	AZOBR_p170044	Ancestral (low confidence)
AZOLI_p20360	Ancestral (medium confidence)	AZOBR_p170045	Horizontally Transferred (medium confidence)
AZOLI_p20361	Ancestral (medium confidence)	AZOBR_p170046	Unassigned
AZOLI_p20362	Ancestral (medium confidence)	AZOBR_p170047	Unassigned
AZOLI_p20363	Unassigned	AZOBR_p170048	Unassigned
AZOLI_p20364	Ancestral (medium confidence)	AZOBR_p170049	Unassigned
AZOLI_p20365	Horizontally Transferred (high confidence)	AZOBR_p170050	Unassigned
AZOLI_p20366	Unassigned	AZOBR_p170051	Unassigned
AZOLI_p20367	Ancestral (medium confidence)	AZOBR_p170052	Unassigned
AZOLI_p20368	Ancestral (medium confidence)	AZOBR_p170053	Unassigned
AZOLI_p20369	Ancestral (medium confidence)	AZOBR_p170054	Horizontally Transferred (high confidence)
AZOLI_p20370	Ancestral (low confidence)	AZOBR_p170055	Unassigned
AZOLI_p20371	Unassigned	AZOBR_p170056	Unassigned
AZOLI_p20372	Horizontally Transferred (high confidence)	AZOBR_p170057	Ancestral (low confidence)
AZOLI_p20373	Horizontally Transferred (high confidence)	AZOBR_p170058	Ancestral (low confidence)
AZOLI_p20374	Unassigned	AZOBR_p170059	Ancestral (low confidence)
AZOLI_p20376	Horizontally Transferred (low confidence)	AZOBR_p170060	Unassigned
AZOLI_p20377	Unassigned	AZOBR_p170061	Horizontally Transferred (medium confidence)
AZOLI_p20379	Horizontally Transferred (high confidence)	AZOBR_p170062	Horizontally Transferred (medium confidence)
AZOLI_p20381	Horizontally Transferred (high confidence)	AZOBR_p170063	Horizontally Transferred (high confidence)
AZOLI_p20382	Ancestral (high confidence)	AZOBR_p170064	Horizontally Transferred (high confidence)
AZOLI_p20383	Ancestral (medium confidence)	AZOBR_p170065	Horizontally Transferred (high confidence)
AZOLI_p20384	Horizontally Transferred (low confidence)	AZOBR_p170066	Horizontally Transferred (high confidence)
AZOLI_p20385	Unassigned	AZOBR_p170067	Unassigned
AZOLI_p20386	Unassigned	AZOBR_p170068	Unassigned
AZOLI_p20390	Unassigned	AZOBR_p170069	Horizontally Transferred (high confidence)
AZOLI_p20391	Horizontally Transferred (high confidence)	AZOBR_p170070	Ancestral (low confidence)
AZOLI_p20392	Horizontally Transferred (high confidence)	AZOBR_p170071	Unassigned
AZOLI_p20393	Horizontally Transferred (high confidence)	AZOBR_p170072	Horizontally Transferred (medium confidence)
AZOLI_p20394	Horizontally Transferred (high confidence)	AZOBR_p170073	Unassigned
AZOLI_p20395	Horizontally Transferred (high confidence)	AZOBR_p170074	Horizontally Transferred (medium confidence)
AZOLI_p20397	Horizontally Transferred (high confidence)	AZOBR_p170075	Unassigned
AZOLI_p20398	Horizontally Transferred (medium confidence)	AZOBR_p170076	Ancestral (low confidence)
AZOLI_p20399	Ancestral (low confidence)	AZOBR_p170077	Horizontally Transferred (low confidence)
AZOLI_p20400	Horizontally Transferred (high confidence)	AZOBR_p170078	Unassigned
AZOLI_p20401	Horizontally Transferred (high confidence)	AZOBR_p170079	Horizontally Transferred (high confidence)
AZOLI_p20402	Horizontally Transferred (low confidence)	AZOBR_p170080	Unassigned
AZOLI_p20403	Horizontally Transferred (medium confidence)	AZOBR_p170081	Ancestral (low confidence)
AZOLI_p20404	Unassigned	AZOBR_p170082	Horizontally Transferred (high confidence)
AZOLI_p20405	Horizontally Transferred (medium confidence)	AZOBR_p170083	Horizontally Transferred (high confidence)
AZOLI_p20406	Ancestral (low confidence)	AZOBR_p170084	Ancestral (low confidence)
AZOLI_p20407	Horizontally Transferred (high confidence)	AZOBR_p170085	Ancestral (medium confidence)
AZOLI_p20408	Ancestral (low confidence)	AZOBR_p170086	Ancestral (high confidence)
AZOLI_p20409	Ancestral (low confidence)	AZOBR_p170087	Horizontally Transferred (high confidence)

AZOLI_p20412	Ancestral (low confidence)	AZOBR_p170088	Horizontally Transferred (high confidence)
AZOLI_p20413	Ancestral (low confidence)	AZOBR_p170089	Ancestral (low confidence)
AZOLI_p20414	Unassigned	AZOBR_p170090	Horizontally Transferred (high confidence)
AZOLI_p20415	Horizontally Transferred (low confidence)	AZOBR_p170091	Ancestral (low confidence)
AZOLI_p20416	Horizontally Transferred (high confidence)	AZOBR_p170092	Unassigned
AZOLI_p20417	Horizontally Transferred (high confidence)	AZOBR_p170093	Ancestral (medium confidence)
AZOLI_p20418	Ancestral (low confidence)	AZOBR_p170094	Ancestral (low confidence)
AZOLI_p20420	Horizontally Transferred (medium confidence)	AZOBR_p170095	Ancestral (high confidence)
AZOLI_p20421	Horizontally Transferred (high confidence)	AZOBR_p170096	Ancestral (high confidence)
AZOLI_p20422	Unassigned	AZOBR_p170097	Ancestral (low confidence)
AZOLI_p20423	Horizontally Transferred (high confidence)	AZOBR_p170098	Ancestral (medium confidence)
AZOLI_p20424	Horizontally Transferred (high confidence)	AZOBR_p170099	Ancestral (low confidence)
AZOLI_p20425	Horizontally Transferred (high confidence)	AZOBR_p170100	Horizontally Transferred (high confidence)
AZOLI_p20426	Unassigned	AZOBR_p170101	Unassigned
AZOLI_p20427	Unassigned	AZOBR_p170102	Unassigned
AZOLI_p20429	Horizontally Transferred (high confidence)	AZOBR_p170103	Unassigned
AZOLI_p20430	Horizontally Transferred (medium confidence)	AZOBR_p170104	Unassigned
AZOLI_p20431	Horizontally Transferred (high confidence)	AZOBR_p170105	Ancestral (medium confidence)
AZOLI_p20432	Horizontally Transferred (medium confidence)	AZOBR_p170106	Horizontally Transferred (high confidence)
AZOLI_p20433	Horizontally Transferred (medium confidence)	AZOBR_p170107	Unassigned
AZOLI_p20435	Horizontally Transferred (medium confidence)	AZOBR_p170108	Unassigned
AZOLI_p20436	Horizontally Transferred (medium confidence)	AZOBR_p170109	Unassigned
AZOLI_p20437	Horizontally Transferred (medium confidence)	AZOBR_p170110	Horizontally Transferred (low confidence)
AZOLI_p20438	Horizontally Transferred (high confidence)	AZOBR_p170111	Unassigned
AZOLI_p20440	Horizontally Transferred (high confidence)	AZOBR_p170112	Unassigned
AZOLI_p20441	Ancestral (high confidence)	AZOBR_p170113	Unassigned
AZOLI_p20442	Horizontally Transferred (high confidence)	AZOBR_p170114	Unassigned
AZOLI_p20443	Horizontally Transferred (high confidence)	AZOBR_p170115	Horizontally Transferred (high confidence)
AZOLI_p20445	Ancestral (low confidence)	AZOBR_p170116	Horizontally Transferred (high confidence)
AZOLI_p20446	Horizontally Transferred (high confidence)	AZOBR_p170117	Horizontally Transferred (high confidence)
AZOLI_p20447	Horizontally Transferred (high confidence)	AZOBR_p170118	Unassigned
AZOLI_p20448	Horizontally Transferred (high confidence)	AZOBR_p170119	Unassigned
AZOLI_p20449	Ancestral (low confidence)	AZOBR_p170120	Unassigned
AZOLI_p20450	Horizontally Transferred (high confidence)	AZOBR_p170121	Ancestral (low confidence)
AZOLI_p20451	Horizontally Transferred (medium confidence)	AZOBR_p170122	Unassigned
AZOLI_p20452	Horizontally Transferred (high confidence)	AZOBR_p170123	Unassigned
AZOLI_p20453	Ancestral (high confidence)	AZOBR_p170124	Unassigned
AZOLI_p20454	Horizontally Transferred (high confidence)	AZOBR_p170125	Horizontally Transferred (high confidence)
AZOLI_p20455	Horizontally Transferred (high confidence)	AZOBR_p170126	Horizontally Transferred (high confidence)
AZOLI_p20457	Horizontally Transferred (high confidence)	AZOBR_p170127	Horizontally Transferred (high confidence)
AZOLI_p20459	Ancestral (high confidence)	AZOBR_p170128	Horizontally Transferred (high confidence)
AZOLI_p20460	Ancestral (medium confidence)	AZOBR_p170129	Horizontally Transferred (high confidence)
AZOLI_p20461	Ancestral (high confidence)	AZOBR_p170130	Horizontally Transferred (high confidence)
AZOLI_p20462	Ancestral (high confidence)	AZOBR_p170131	Horizontally Transferred (high confidence)
AZOLI_p20463	Ancestral (high confidence)	AZOBR_p170132	Unassigned
AZOLI_p20464	Ancestral (low confidence)	AZOBR_p170133	Horizontally Transferred (high confidence)
AZOLI_p20465	Horizontally Transferred (high confidence)	AZOBR_p170134	Unassigned
AZOLI_p20466	Horizontally Transferred (high confidence)	AZOBR_p170135	Ancestral (low confidence)
AZOLI_p20467	Horizontally Transferred (medium confidence)	AZOBR_p170136	Horizontally Transferred (high confidence)
AZOLI_p20468	Horizontally Transferred (medium confidence)	AZOBR_p170137	Horizontally Transferred (high confidence)
AZOLI_p20469	Unassigned	AZOBR_p170138	Horizontally Transferred (high confidence)
AZOLI_p20471	Horizontally Transferred (high confidence)	AZOBR_p170139	Horizontally Transferred (high confidence)
AZOLI_p20473	Horizontally Transferred (high confidence)	AZOBR_p170140	Horizontally Transferred (high confidence)
AZOLI_p20474	Horizontally Transferred (high confidence)	AZOBR_p170141	Horizontally Transferred (high confidence)
AZOLI_p20475	Horizontally Transferred (high confidence)	AZOBR_p170142	Unassigned
AZOLI_p20476	Horizontally Transferred (high confidence)	AZOBR_p170143	Horizontally Transferred (high confidence)
AZOLI_p20477	Horizontally Transferred (high confidence)	AZOBR_p170144	Horizontally Transferred (medium confidence)
AZOLI_p20478	Horizontally Transferred (high confidence)	AZOBR_p170145	Horizontally Transferred (high confidence)
AZOLI_p20480	Horizontally Transferred (medium confidence)	AZOBR_p170146	Ancestral (high confidence)
AZOLI_p20481	Horizontally Transferred (high confidence)	AZOBR_p170147	Horizontally Transferred (high confidence)
AZOLI_p20482	Horizontally Transferred (low confidence)	AZOBR_p170148	Horizontally Transferred (high confidence)
AZOLI_p20483	Horizontally Transferred (low confidence)	AZOBR_p170149	Horizontally Transferred (high confidence)
AZOLI_p20484	Horizontally Transferred (low confidence)	AZOBR_p170150	Ancestral (low confidence)
AZOLI_p20485	Horizontally Transferred (high confidence)	AZOBR_p170151	Unassigned
AZOLI_p20486	Horizontally Transferred (high confidence)	AZOBR_p170152	Horizontally Transferred (high confidence)
AZOLI_p20488	Horizontally Transferred (high confidence)	AZOBR_p170153	Horizontally Transferred (high confidence)
AZOLI_p20489	Horizontally Transferred (low confidence)	AZOBR_p170154	Horizontally Transferred (high confidence)
AZOLI_p20491	Horizontally Transferred (medium confidence)	AZOBR_p170155	Horizontally Transferred (high confidence)
AZOLI_p20492	Horizontally Transferred (high confidence)	AZOBR_p170156	Horizontally Transferred (high confidence)
AZOLI_p20493	Horizontally Transferred (high confidence)	AZOBR_p170157	Horizontally Transferred (high confidence)
AZOLI_p20494	Horizontally Transferred (high confidence)	AZOBR_p170158	Unassigned
AZOLI_p20495	Horizontally Transferred (high confidence)	AZOBR_p170159	Horizontally Transferred (high confidence)
AZOLI_p20496	Horizontally Transferred (high confidence)	AZOBR_p170160	Horizontally Transferred (high confidence)
AZOLI_p20497	Horizontally Transferred (high confidence)	AZOBR_p170161	Unassigned
AZOLI_p20498	Horizontally Transferred (high confidence)	AZOBR_p180001	Horizontally Transferred (high confidence)

AZOLI_p20499	Horizontally Transferred (high confidence)	AZOBR_p180002	Horizontally Transferred (high confidence)
AZOLI_p20500	Horizontally Transferred (high confidence)	AZOBR_p180003	Horizontally Transferred (high confidence)
AZOLI_p20501	Horizontally Transferred (medium confidence)	AZOBR_p180004	Horizontally Transferred (high confidence)
AZOLI_p20502	Horizontally Transferred (high confidence)	AZOBR_p180005	Horizontally Transferred (medium confidence)
AZOLI_p20503	Horizontally Transferred (high confidence)	AZOBR_p180006	Horizontally Transferred (high confidence)
AZOLI_p20504	Horizontally Transferred (high confidence)	AZOBR_p180007	Horizontally Transferred (high confidence)
AZOLI_p20505	Horizontally Transferred (high confidence)	AZOBR_p180008	Unassigned
AZOLI_p20506	Horizontally Transferred (high confidence)	AZOBR_p180009	Ancestral (low confidence)
AZOLI_p20507	Horizontally Transferred (high confidence)	AZOBR_p180010	Ancestral (low confidence)
AZOLI_p20508	Horizontally Transferred (high confidence)	AZOBR_p180011	Horizontally Transferred (medium confidence)
AZOLI_p20509	Unassigned	AZOBR_p180012	Horizontally Transferred (high confidence)
AZOLI_p20510	Unassigned	AZOBR_p180013	Horizontally Transferred (high confidence)
AZOLI_p20511	Ancestral (medium confidence)	AZOBR_p180014	Ancestral (low confidence)
AZOLI_p20512	Unassigned	AZOBR_p180015	Horizontally Transferred (high confidence)
AZOLI_p20513	Unassigned	AZOBR_p180016	Horizontally Transferred (high confidence)
AZOLI_p20514	Horizontally Transferred (high confidence)	AZOBR_p180017	Horizontally Transferred (high confidence)
AZOLI_p20515	Horizontally Transferred (high confidence)	AZOBR_p180018	Horizontally Transferred (high confidence)
AZOLI_p20516	Horizontally Transferred (high confidence)	AZOBR_p180019	Horizontally Transferred (high confidence)
AZOLI_p20517	Horizontally Transferred (high confidence)	AZOBR_p180020	Unassigned
AZOLI_p20518	Horizontally Transferred (medium confidence)	AZOBR_p180021	Ancestral (low confidence)
AZOLI_p20520	Horizontally Transferred (high confidence)	AZOBR_p190001	Ancestral (low confidence)
AZOLI_p20521	Horizontally Transferred (high confidence)	AZOBR_p190002	Ancestral (medium confidence)
AZOLI_p20522	Horizontally Transferred (medium confidence)	AZOBR_p190003	Ancestral (low confidence)
AZOLI_p20523	Ancestral (medium confidence)	AZOBR_p190004	Ancestral (low confidence)
AZOLI_p20524	Unassigned	AZOBR_p190005	Ancestral (medium confidence)
AZOLI_p20525	Unassigned	AZOBR_p190006	Unassigned
AZOLI_p20526	Ancestral (high confidence)	AZOBR_p190007	Unassigned
AZOLI_p20527	Horizontally Transferred (medium confidence)	AZOBR_p190008	Unassigned
AZOLI_p20528	Unassigned	AZOBR_p1100001	Horizontally Transferred (high confidence)
AZOLI_p20529	Horizontally Transferred (high confidence)	AZOBR_p1100002	Ancestral (low confidence)
AZOLI_p20530	Ancestral (low confidence)	AZOBR_p1100003	Unassigned
AZOLI_p20531	Ancestral (low confidence)	AZOBR_p1100004	Horizontally Transferred (high confidence)
AZOLI_p20532	Horizontally Transferred (medium confidence)	AZOBR_p1100005	Ancestral (low confidence)
AZOLI_p20533	Unassigned	AZOBR_p1100006	Ancestral (low confidence)
AZOLI_p20534	Horizontally Transferred (high confidence)	AZOBR_p1100007	Ancestral (low confidence)
AZOLI_p20535	Horizontally Transferred (high confidence)	AZOBR_p1100008	Ancestral (low confidence)
AZOLI_p20536	Horizontally Transferred (high confidence)	AZOBR_p1100009	Ancestral (low confidence)
AZOLI_p20537	Unassigned	AZOBR_p1100010	Horizontally Transferred (high confidence)
AZOLI_p20539	Horizontally Transferred (high confidence)	AZOBR_p1100011	Ancestral (high confidence)
AZOLI_p20540	Ancestral (medium confidence)	AZOBR_p1100012	Horizontally Transferred (low confidence)
AZOLI_p20541	Horizontally Transferred (high confidence)	AZOBR_p1100013	Horizontally Transferred (high confidence)
AZOLI_p20542	Unassigned	AZOBR_p1100014	Horizontally Transferred (high confidence)
AZOLI_p20543	Horizontally Transferred (high confidence)	AZOBR_p1100015	Horizontally Transferred (high confidence)
AZOLI_p20544	Horizontally Transferred (high confidence)	AZOBR_p1100016	Horizontally Transferred (high confidence)
AZOLI_p20545	Horizontally Transferred (high confidence)	AZOBR_p1100017	Horizontally Transferred (high confidence)
AZOLI_p20546	Unassigned	AZOBR_p1100018	Unassigned
AZOLI_p20547	Ancestral (medium confidence)	AZOBR_p1100019	Horizontally Transferred (high confidence)
AZOLI_p20548	Horizontally Transferred (medium confidence)	AZOBR_p1100020	Unassigned
AZOLI_p20549	Ancestral (low confidence)	AZOBR_p1100021	Horizontally Transferred (high confidence)
AZOLI_p20550	Ancestral (medium confidence)	AZOBR_p1100022	Ancestral (high confidence)
AZOLI_p20553	Ancestral (low confidence)	AZOBR_p1100023	Ancestral (low confidence)
AZOLI_p20554	Ancestral (low confidence)	AZOBR_p1100024	Horizontally Transferred (high confidence)
AZOLI_p20555	Unassigned	AZOBR_p1100025	Unassigned
AZOLI_p20556	Ancestral (low confidence)	AZOBR_p1100026	Ancestral (medium confidence)
AZOLI_p20557	Unassigned	AZOBR_p1100027	Ancestral (medium confidence)
AZOLI_p20558	Horizontally Transferred (medium confidence)	AZOBR_p1100028	Unassigned
AZOLI_p20559	Horizontally Transferred (high confidence)	AZOBR_p1100029	Ancestral (medium confidence)
AZOLI_p20560	Horizontally Transferred (high confidence)	AZOBR_p1100030	Ancestral (medium confidence)
AZOLI_p20561	Horizontally Transferred (high confidence)	AZOBR_p1100031	Ancestral (medium confidence)
AZOLI_p20562	Horizontally Transferred (high confidence)	AZOBR_p1100032	Horizontally Transferred (high confidence)
AZOLI_p20563	Unassigned	AZOBR_p1100033	Horizontally Transferred (low confidence)
AZOLI_p20564	Horizontally Transferred (high confidence)	AZOBR_p1100034	Unassigned
AZOLI_p20565	Horizontally Transferred (high confidence)	AZOBR_p1100035	Ancestral (medium confidence)
AZOLI_p20566	Horizontally Transferred (high confidence)	AZOBR_p1100036	Unassigned
AZOLI_p20567	Horizontally Transferred (high confidence)	AZOBR_p1100037	Unassigned
AZOLI_p20568	Horizontally Transferred (high confidence)	AZOBR_p1100038	Ancestral (high confidence)
AZOLI_p20569	Horizontally Transferred (high confidence)	AZOBR_p1100039	Ancestral (medium confidence)
AZOLI_p20570	Horizontally Transferred (high confidence)	AZOBR_p1100040	Ancestral (medium confidence)
AZOLI_p20571	Ancestral (low confidence)	AZOBR_p1100041	Horizontally Transferred (high confidence)
AZOLI_p20572	Horizontally Transferred (high confidence)	AZOBR_p1100042	Horizontally Transferred (high confidence)
AZOLI_p20573	Horizontally Transferred (high confidence)	AZOBR_p1100043	Horizontally Transferred (high confidence)
AZOLI_p20574	Horizontally Transferred (high confidence)	AZOBR_p1100044	Horizontally Transferred (medium confidence)
AZOLI_p20575	Horizontally Transferred (high confidence)	AZOBR_p1100045	Horizontally Transferred (high confidence)
AZOLI_p20577	Horizontally Transferred (high confidence)	AZOBR_p1100046	Horizontally Transferred (high confidence)
AZOLI_p20578	Horizontally Transferred (high confidence)	AZOBR_p1100047	Horizontally Transferred (high confidence)

AZOLI_p20579	Unassigned	AZOBR_p1100048	Unassigned
AZOLI_p20580	Horizontally Transferred (high confidence)	AZOBR_p1100049	Horizontally Transferred (high confidence)
AZOLI_p20581	Horizontally Transferred (medium confidence)	AZOBR_p1100050	Unassigned
AZOLI_p20583	Unassigned	AZOBR_p1100051	Unassigned
AZOLI_p20584	Horizontally Transferred (high confidence)	AZOBR_p1100056	Unassigned
AZOLI_p20585	Horizontally Transferred (high confidence)	AZOBR_p1100059	Horizontally Transferred (high confidence)
AZOLI_p20586	Horizontally Transferred (high confidence)	AZOBR_p1100060	Horizontally Transferred (high confidence)
AZOLI_p20587	Horizontally Transferred (high confidence)	AZOBR_p1100061	Ancestral (low confidence)
AZOLI_p20588	Horizontally Transferred (high confidence)	AZOBR_p1100062	Ancestral (low confidence)
AZOLI_p20589	Horizontally Transferred (high confidence)	AZOBR_p1100063	Unassigned
AZOLI_p20590	Horizontally Transferred (high confidence)	AZOBR_p1100064	Unassigned
AZOLI_p20591	Horizontally Transferred (high confidence)	AZOBR_p1100065	Horizontally Transferred (low confidence)
AZOLI_p20592	Horizontally Transferred (high confidence)	AZOBR_p1100066	Horizontally Transferred (high confidence)
AZOLI_p20593	Horizontally Transferred (high confidence)	AZOBR_p1100067	Horizontally Transferred (high confidence)
AZOLI_p20594	Horizontally Transferred (high confidence)	AZOBR_p1100068	Ancestral (low confidence)
AZOLI_p20595	Horizontally Transferred (high confidence)	AZOBR_p1100069	Horizontally Transferred (high confidence)
AZOLI_p20596	Horizontally Transferred (high confidence)	AZOBR_p1100070	Horizontally Transferred (high confidence)
AZOLI_p20597	Unassigned	AZOBR_p1100071	Unassigned
AZOLI_p20598	Horizontally Transferred (high confidence)	AZOBR_p1100072	Ancestral (low confidence)
AZOLI_p20599	Ancestral (low confidence)	AZOBR_p1100073	Ancestral (low confidence)
AZOLI_p20601	Ancestral (low confidence)	AZOBR_p1100074	Ancestral (low confidence)
AZOLI_p20602	Unassigned	AZOBR_p1100075	Unassigned
AZOLI_p20603	Horizontally Transferred (high confidence)	AZOBR_p1100076	Ancestral (low confidence)
AZOLI_p20604	Horizontally Transferred (high confidence)	AZOBR_p1100077	Ancestral (low confidence)
AZOLI_p20605	Horizontally Transferred (high confidence)	AZOBR_p1100078	Ancestral (low confidence)
AZOLI_p20606	Horizontally Transferred (high confidence)	AZOBR_p1100079	Ancestral (medium confidence)
AZOLI_p20607	Horizontally Transferred (high confidence)	AZOBR_p1100080	Unassigned
AZOLI_p20608	Horizontally Transferred (high confidence)	AZOBR_p1100081	Unassigned
AZOLI_p20609	Unassigned	AZOBR_p1100082	Ancestral (medium confidence)
AZOLI_p20610	Unassigned	AZOBR_p1100083	Ancestral (low confidence)
AZOLI_p20611	Ancestral (low confidence)	AZOBR_p1100084	Unassigned
AZOLI_p20614	Ancestral (medium confidence)	AZOBR_p1100085	Unassigned
AZOLI_p20615	Ancestral (medium confidence)	AZOBR_p1100086	Ancestral (medium confidence)
AZOLI_p20616	Horizontally Transferred (high confidence)	AZOBR_p1100087	Horizontally Transferred (high confidence)
AZOLI_p20617	Unassigned	AZOBR_p1100088	Unassigned
AZOLI_p20619	Horizontally Transferred (high confidence)	AZOBR_p1100089	Ancestral (medium confidence)
AZOLI_p20620	Horizontally Transferred (high confidence)	AZOBR_p1100090	Horizontally Transferred (high confidence)
AZOLI_p20621	Horizontally Transferred (high confidence)	AZOBR_p1100091	Unassigned
AZOLI_p20622	Horizontally Transferred (high confidence)	AZOBR_p1100092	Horizontally Transferred (high confidence)
AZOLI_p20623	Horizontally Transferred (high confidence)	AZOBR_p1100093	Ancestral (high confidence)
AZOLI_p20624	Horizontally Transferred (high confidence)	AZOBR_p1100094	Horizontally Transferred (medium confidence)
AZOLI_p20625	Horizontally Transferred (high confidence)	AZOBR_p1100095	Horizontally Transferred (high confidence)
AZOLI_p20626	Horizontally Transferred (high confidence)	AZOBR_p1100096	Horizontally Transferred (high confidence)
AZOLI_p20627	Horizontally Transferred (medium confidence)	AZOBR_p1100097	Ancestral (low confidence)
AZOLI_p20628	Horizontally Transferred (medium confidence)	AZOBR_p1100098	Horizontally Transferred (high confidence)
AZOLI_p20629	Horizontally Transferred (high confidence)	AZOBR_p1100099	Horizontally Transferred (high confidence)
AZOLI_p20630	Horizontally Transferred (high confidence)	AZOBR_p1100100	Horizontally Transferred (medium confidence)
AZOLI_p20631	Horizontally Transferred (high confidence)	AZOBR_p1100101	Horizontally Transferred (low confidence)
AZOLI_p20632	Ancestral (low confidence)	AZOBR_p1100102	Ancestral (low confidence)
AZOLI_p20633	Unassigned	AZOBR_p1100103	Horizontally Transferred (high confidence)
AZOLI_p20634	Horizontally Transferred (high confidence)	AZOBR_p1100104	Horizontally Transferred (high confidence)
AZOLI_p20635	Ancestral (low confidence)	AZOBR_p1100105	Horizontally Transferred (high confidence)
AZOLI_p20637	Ancestral (low confidence)	AZOBR_p1100106	Unassigned
AZOLI_p20638	Ancestral (low confidence)	AZOBR_p1100107	Horizontally Transferred (medium confidence)
AZOLI_p20639	Ancestral (low confidence)	AZOBR_p1100108	Ancestral (medium confidence)
AZOLI_p20640	Ancestral (low confidence)	AZOBR_p1110001	Unassigned
AZOLI_p20641	Ancestral (low confidence)	AZOBR_p1110002	Horizontally Transferred (high confidence)
AZOLI_p20642	Ancestral (low confidence)	AZOBR_p1110003	Ancestral (low confidence)
AZOLI_p20643	Ancestral (low confidence)	AZOBR_p1110004	Horizontally Transferred (high confidence)
AZOLI_p20644	Horizontally Transferred (low confidence)	AZOBR_p1110005	Ancestral (low confidence)
AZOLI_p20645	Horizontally Transferred (high confidence)	AZOBR_p1110006	Unassigned
AZOLI_p20646	Horizontally Transferred (high confidence)	AZOBR_p1110007	Unassigned
AZOLI_p20647	Horizontally Transferred (high confidence)	AZOBR_p1110008	Ancestral (low confidence)
AZOLI_p20648	Horizontally Transferred (high confidence)	AZOBR_p1110009	Horizontally Transferred (high confidence)
AZOLI_p20649	Horizontally Transferred (high confidence)	AZOBR_p1110010	Unassigned
AZOLI_p20650	Horizontally Transferred (high confidence)	AZOBR_p1110011	Unassigned
AZOLI_p20651	Horizontally Transferred (high confidence)	AZOBR_p1110012	Horizontally Transferred (medium confidence)
AZOLI_p20652	Ancestral (low confidence)	AZOBR_p1110013	Horizontally Transferred (high confidence)
AZOLI_p20653	Horizontally Transferred (high confidence)	AZOBR_p1110014	Ancestral (low confidence)
AZOLI_p20654	Ancestral (low confidence)	AZOBR_p1110015	Unassigned
AZOLI_p20655	Unassigned	AZOBR_p1110016	Horizontally Transferred (high confidence)
AZOLI_p20656	Ancestral (low confidence)	AZOBR_p1110017	Horizontally Transferred (high confidence)
AZOLI_p20658	Horizontally Transferred (high confidence)	AZOBR_p1110018	Horizontally Transferred (high confidence)
AZOLI_p20659	Horizontally Transferred (high confidence)	AZOBR_p1110019	Horizontally Transferred (high confidence)
AZOLI_p20660	Horizontally Transferred (high confidence)	AZOBR_p1110020	Unassigned

AZOLI_p20661	Horizontally Transferred (high confidence)	AZOBR_p1110021	Horizontally Transferred (high confidence)
AZOLI_p20662	Horizontally Transferred (high confidence)	AZOBR_p1110022	Horizontally Transferred (high confidence)
AZOLI_p20663	Horizontally Transferred (high confidence)	AZOBR_p1110023	Horizontally Transferred (high confidence)
AZOLI_p20665	Horizontally Transferred (medium confidence)	AZOBR_p1110024	Ancestral (medium confidence)
AZOLI_p20666	Horizontally Transferred (medium confidence)	AZOBR_p1110025	Ancestral (low confidence)
AZOLI_p20667	Horizontally Transferred (medium confidence)	AZOBR_p1110026	Ancestral (low confidence)
AZOLI_p20668	Unassigned	AZOBR_p1110027	Horizontally Transferred (high confidence)
AZOLI_p20669	Ancestral (low confidence)	AZOBR_p1110028	Horizontally Transferred (high confidence)
AZOLI_p20670	Ancestral (low confidence)	AZOBR_p1110029	Unassigned
AZOLI_p20671	Ancestral (low confidence)	AZOBR_p1110030	Ancestral (medium confidence)
AZOLI_p20672	Horizontally Transferred (high confidence)	AZOBR_p1110031	Horizontally Transferred (high confidence)
AZOLI_p20674	Horizontally Transferred (high confidence)	AZOBR_p1110032	Ancestral (high confidence)
AZOLI_p20675	Ancestral (low confidence)	AZOBR_p1110033	Ancestral (high confidence)
AZOLI_p20676	Horizontally Transferred (medium confidence)	AZOBR_p1110034	Ancestral (medium confidence)
AZOLI_p20677	Horizontally Transferred (high confidence)	AZOBR_p1110035	Ancestral (medium confidence)
AZOLI_p20678	Horizontally Transferred (high confidence)	AZOBR_p1110036	Unassigned
AZOLI_p20679	Horizontally Transferred (medium confidence)	AZOBR_p1110037	Ancestral (medium confidence)
AZOLI_p20680	Horizontally Transferred (medium confidence)	AZOBR_p1110038	Ancestral (low confidence)
AZOLI_p20681	Horizontally Transferred (high confidence)	AZOBR_p1110039	Ancestral (medium confidence)
AZOLI_p20682	Horizontally Transferred (high confidence)	AZOBR_p1110040	Ancestral (medium confidence)
AZOLI_p20683	Horizontally Transferred (high confidence)	AZOBR_p1110041	Ancestral (medium confidence)
AZOLI_p20684	Horizontally Transferred (high confidence)	AZOBR_p1110042	Ancestral (high confidence)
AZOLI_p20685	Horizontally Transferred (high confidence)	AZOBR_p1110043	Ancestral (high confidence)
AZOLI_p20686	Horizontally Transferred (high confidence)	AZOBR_p1110044	Ancestral (high confidence)
AZOLI_p20687	Horizontally Transferred (high confidence)	AZOBR_p1110045	Ancestral (high confidence)
AZOLI_p20688	Horizontally Transferred (high confidence)	AZOBR_p1110046	Ancestral (medium confidence)
AZOLI_p20689	Ancestral (high confidence)	AZOBR_p1110047	Ancestral (high confidence)
AZOLI_p20690	Horizontally Transferred (high confidence)	AZOBR_p1110048	Ancestral (low confidence)
AZOLI_p20691	Horizontally Transferred (high confidence)	AZOBR_p1110049	Ancestral (high confidence)
AZOLI_p20692	Horizontally Transferred (high confidence)	AZOBR_p1110050	Ancestral (medium confidence)
AZOLI_p20693	Ancestral (low confidence)	AZOBR_p1110051	Ancestral (high confidence)
AZOLI_p20694	Horizontally Transferred (high confidence)	AZOBR_p1110052	Ancestral (high confidence)
AZOLI_p20695	Horizontally Transferred (high confidence)	AZOBR_p1110053	Ancestral (high confidence)
AZOLI_p20696	Horizontally Transferred (high confidence)	AZOBR_p1110054	Ancestral (high confidence)
AZOLI_p20697	Horizontally Transferred (high confidence)	AZOBR_p1110055	Ancestral (high confidence)
AZOLI_p20698	Horizontally Transferred (high confidence)	AZOBR_p1110056	Ancestral (high confidence)
AZOLI_p20699	Horizontally Transferred (high confidence)	AZOBR_p1110057	Horizontally Transferred (high confidence)
AZOLI_p20700	Horizontally Transferred (high confidence)	AZOBR_p1110058	Ancestral (medium confidence)
AZOLI_p20701	Horizontally Transferred (high confidence)	AZOBR_p1110059	Ancestral (high confidence)
AZOLI_p20702	Horizontally Transferred (high confidence)	AZOBR_p1110060	Ancestral (medium confidence)
AZOLI_p20703	Horizontally Transferred (high confidence)	AZOBR_p1110061	Horizontally Transferred (high confidence)
AZOLI_p20704	Horizontally Transferred (high confidence)	AZOBR_p1110062	Unassigned
AZOLI_p20705	Horizontally Transferred (high confidence)	AZOBR_p1110063	Ancestral (high confidence)
AZOLI_p20706	Horizontally Transferred (high confidence)	AZOBR_p1110064	Ancestral (low confidence)
AZOLI_p20707	Ancestral (medium confidence)	AZOBR_p1110065	Horizontally Transferred (high confidence)
AZOLI_p20708	Ancestral (medium confidence)	AZOBR_p1110066	Horizontally Transferred (high confidence)
AZOLI_p20709	Ancestral (low confidence)	AZOBR_p1110067	Horizontally Transferred (high confidence)
AZOLI_p20710	Horizontally Transferred (high confidence)	AZOBR_p1110068	Horizontally Transferred (high confidence)
AZOLI_p20711	Horizontally Transferred (medium confidence)	AZOBR_p1110069	Ancestral (low confidence)
AZOLI_p20712	Horizontally Transferred (high confidence)	AZOBR_p1110070	Unassigned
AZOLI_p20713	Horizontally Transferred (high confidence)	AZOBR_p1110071	Ancestral (high confidence)
AZOLI_p20714	Horizontally Transferred (high confidence)	AZOBR_p1110072	Ancestral (medium confidence)
AZOLI_p20715	Horizontally Transferred (high confidence)	AZOBR_p1110073	Unassigned
AZOLI_p20716	Horizontally Transferred (high confidence)	AZOBR_p1110074	Ancestral (high confidence)
AZOLI_p20717	Horizontally Transferred (high confidence)	AZOBR_p1110075	Ancestral (medium confidence)
AZOLI_p20718	Horizontally Transferred (high confidence)	AZOBR_p1110076	Ancestral (high confidence)
AZOLI_p20719	Horizontally Transferred (high confidence)	AZOBR_p1110077	Horizontally Transferred (high confidence)
AZOLI_p20720	Horizontally Transferred (high confidence)	AZOBR_p1110078	Ancestral (high confidence)
AZOLI_p20721	Horizontally Transferred (high confidence)	AZOBR_p1110079	Ancestral (high confidence)
AZOLI_p20722	Ancestral (high confidence)	AZOBR_p1110080	Ancestral (low confidence)
AZOLI_p20723	Ancestral (high confidence)	AZOBR_p1110081	Ancestral (medium confidence)
AZOLI_p20724	Ancestral (medium confidence)	AZOBR_p1110082	Ancestral (high confidence)
AZOLI_p20725	Horizontally Transferred (high confidence)	AZOBR_p1110083	Ancestral (high confidence)
AZOLI_p20726	Ancestral (low confidence)	AZOBR_p1110084	Ancestral (medium confidence)
AZOLI_p20727	Unassigned	AZOBR_p1110085	Ancestral (medium confidence)
AZOLI_p20728	Ancestral (low confidence)	AZOBR_p1110086	Horizontally Transferred (high confidence)
AZOLI_p20729	Ancestral (low confidence)	AZOBR_p1110087	Horizontally Transferred (high confidence)
AZOLI_p20730	Ancestral (low confidence)	AZOBR_p1110088	Unassigned
AZOLI_p20731	Ancestral (low confidence)	AZOBR_p1110089	Horizontally Transferred (high confidence)
AZOLI_p20733	Ancestral (low confidence)	AZOBR_p1110090	Horizontally Transferred (low confidence)
AZOLI_p20734	Unassigned	AZOBR_p1110091	Horizontally Transferred (low confidence)
AZOLI_p20735	Horizontally Transferred (high confidence)	AZOBR_p1110092	Horizontally Transferred (low confidence)
AZOLI_p20736	Ancestral (low confidence)	AZOBR_p1110093	Horizontally Transferred (high confidence)
AZOLI_p20737	Ancestral (low confidence)	AZOBR_p1110094	Horizontally Transferred (high confidence)
AZOLI_p20738	Ancestral (low confidence)	AZOBR_p1110095	Horizontally Transferred (high confidence)

AZOLI_p20739	Ancestral (low confidence)	AZOBR_p1110096	Horizontally Transferred (high confidence)
AZOLI_p20740	Ancestral (low confidence)	AZOBR_p1110097	Horizontally Transferred (high confidence)
AZOLI_p20741	Ancestral (low confidence)	AZOBR_p1110098	Ancestral (low confidence)
AZOLI_p20742	Horizontally Transferred (high confidence)	AZOBR_p1110099	Horizontally Transferred (high confidence)
AZOLI_p20743	Horizontally Transferred (low confidence)	AZOBR_p1110100	Ancestral (medium confidence)
AZOLI_p20744	Unassigned	AZOBR_p1110101	Ancestral (low confidence)
AZOLI_p20745	Ancestral (medium confidence)	AZOBR_p1110102	Ancestral (low confidence)
AZOLI_p20747	Ancestral (low confidence)	AZOBR_p1110103	Ancestral (low confidence)
AZOLI_p20748	Ancestral (high confidence)	AZOBR_p1110104	Ancestral (low confidence)
AZOLI_p30001	Horizontally Transferred (high confidence)	AZOBR_p1110105	Ancestral (low confidence)
AZOLI_p30002	Horizontally Transferred (high confidence)	AZOBR_p1110106	Ancestral (low confidence)
AZOLI_p30004	Horizontally Transferred (medium confidence)	AZOBR_p1110107	Ancestral (low confidence)
AZOLI_p30005	Ancestral (low confidence)	AZOBR_p1110108	Ancestral (low confidence)
AZOLI_p30006	Horizontally Transferred (medium confidence)	AZOBR_p1110109	Ancestral (low confidence)
AZOLI_p30007	Ancestral (low confidence)	AZOBR_p1110110	Horizontally Transferred (high confidence)
AZOLI_p30008	Ancestral (low confidence)	AZOBR_p1110111	Horizontally Transferred (high confidence)
AZOLI_p30009	Ancestral (medium confidence)	AZOBR_p1110112	Horizontally Transferred (high confidence)
AZOLI_p30010	Horizontally Transferred (high confidence)	AZOBR_p1110113	Unassigned
AZOLI_p30011	Horizontally Transferred (high confidence)	AZOBR_p1110114	Unassigned
AZOLI_p30012	Horizontally Transferred (high confidence)	AZOBR_p1110115	Ancestral (low confidence)
AZOLI_p30013	Horizontally Transferred (high confidence)	AZOBR_p1110116	Unassigned
AZOLI_p30014	Horizontally Transferred (high confidence)	AZOBR_p1110117	Unassigned
AZOLI_p30016	Horizontally Transferred (high confidence)	AZOBR_p1110118	Ancestral (low confidence)
AZOLI_p30017	Unassigned	AZOBR_p1110119	Unassigned
AZOLI_p30018	Horizontally Transferred (high confidence)	AZOBR_p1110120	Horizontally Transferred (high confidence)
AZOLI_p30019	Horizontally Transferred (high confidence)	AZOBR_p1110121	Ancestral (medium confidence)
AZOLI_p30020	Horizontally Transferred (high confidence)	AZOBR_p1110122	Horizontally Transferred (high confidence)
AZOLI_p30021	Horizontally Transferred (high confidence)	AZOBR_p1110123	Ancestral (low confidence)
AZOLI_p30022	Horizontally Transferred (high confidence)	AZOBR_p1110124	Ancestral (low confidence)
AZOLI_p30023	Horizontally Transferred (high confidence)	AZOBR_p1110125	Unassigned
AZOLI_p30024	Horizontally Transferred (high confidence)	AZOBR_p1110126	Ancestral (medium confidence)
AZOLI_p30025	Horizontally Transferred (high confidence)	AZOBR_p1110127	Ancestral (low confidence)
AZOLI_p30026	Horizontally Transferred (high confidence)	AZOBR_p1110128	Horizontally Transferred (high confidence)
AZOLI_p30027	Unassigned	AZOBR_p1110129	Ancestral (high confidence)
AZOLI_p30028	Horizontally Transferred (high confidence)	AZOBR_p1110130	Horizontally Transferred (high confidence)
AZOLI_p30029	Horizontally Transferred (high confidence)	AZOBR_p1110131	Unassigned
AZOLI_p30030	Horizontally Transferred (high confidence)	AZOBR_p1110132	Unassigned
AZOLI_p30031	Unassigned	AZOBR_p1110133	Horizontally Transferred (high confidence)
AZOLI_p30032	Ancestral (low confidence)	AZOBR_p1110134	Horizontally Transferred (high confidence)
AZOLI_p30033	Ancestral (low confidence)	AZOBR_p1110135	Unassigned
AZOLI_p30035	Horizontally Transferred (high confidence)	AZOBR_p1110136	Ancestral (low confidence)
AZOLI_p30036	Horizontally Transferred (medium confidence)	AZOBR_p1110137	Unassigned
AZOLI_p30037	Horizontally Transferred (high confidence)	AZOBR_p1110138	Horizontally Transferred (high confidence)
AZOLI_p30039	Horizontally Transferred (high confidence)	AZOBR_p1110139	Ancestral (low confidence)
AZOLI_p30040	Horizontally Transferred (high confidence)	AZOBR_p1110140	Unassigned
AZOLI_p30041	Horizontally Transferred (high confidence)	AZOBR_p1110141	Ancestral (medium confidence)
AZOLI_p30042	Horizontally Transferred (high confidence)	AZOBR_p1110142	Unassigned
AZOLI_p30043	Horizontally Transferred (high confidence)	AZOBR_p1110145	Unassigned
AZOLI_p30044	Horizontally Transferred (high confidence)	AZOBR_p1110149	Horizontally Transferred (high confidence)
AZOLI_p30045	Ancestral (low confidence)	AZOBR_p1110150	Horizontally Transferred (high confidence)
AZOLI_p30046	Horizontally Transferred (medium confidence)	AZOBR_p1110151	Horizontally Transferred (high confidence)
AZOLI_p30047	Horizontally Transferred (high confidence)	AZOBR_p1110152	Horizontally Transferred (high confidence)
AZOLI_p30049	Horizontally Transferred (high confidence)	AZOBR_p1110153	Horizontally Transferred (high confidence)
AZOLI_p30050	Horizontally Transferred (high confidence)	AZOBR_p1110154	Unassigned
AZOLI_p30051	Horizontally Transferred (high confidence)	AZOBR_p1110155	Horizontally Transferred (high confidence)
AZOLI_p30052	Horizontally Transferred (high confidence)	AZOBR_p1110156	Horizontally Transferred (high confidence)
AZOLI_p30053	Unassigned	AZOBR_p1110157	Horizontally Transferred (high confidence)
AZOLI_p30054	Horizontally Transferred (high confidence)	AZOBR_p1110158	Horizontally Transferred (high confidence)
AZOLI_p30055	Unassigned	AZOBR_p1110159	Horizontally Transferred (high confidence)
AZOLI_p30058	Unassigned	AZOBR_p1110160	Horizontally Transferred (high confidence)
AZOLI_p30059	Ancestral (medium confidence)	AZOBR_p1110161	Unassigned
AZOLI_p30060	Horizontally Transferred (high confidence)	AZOBR_p1110162	Unassigned
AZOLI_p30061	Unassigned	AZOBR_p1110163	Horizontally Transferred (high confidence)
AZOLI_p30062	Horizontally Transferred (high confidence)	AZOBR_p1110164	Horizontally Transferred (high confidence)
AZOLI_p30063	Horizontally Transferred (high confidence)	AZOBR_p1110165	Horizontally Transferred (high confidence)
AZOLI_p30064	Horizontally Transferred (high confidence)	AZOBR_p1110166	Horizontally Transferred (high confidence)
AZOLI_p30065	Horizontally Transferred (high confidence)	AZOBR_p1110167	Ancestral (low confidence)
AZOLI_p30066	Horizontally Transferred (medium confidence)	AZOBR_p1110168	Horizontally Transferred (high confidence)
AZOLI_p30067	Ancestral (low confidence)	AZOBR_p1110169	Unassigned
AZOLI_p30068	Horizontally Transferred (high confidence)	AZOBR_p1110170	Ancestral (low confidence)
AZOLI_p30069	Horizontally Transferred (high confidence)	AZOBR_p1110171	Unassigned
AZOLI_p30070	Horizontally Transferred (high confidence)	AZOBR_p1120001	Horizontally Transferred (high confidence)
AZOLI_p30071	Horizontally Transferred (high confidence)	AZOBR_p1120002	Horizontally Transferred (high confidence)
AZOLI_p30072	Horizontally Transferred (high confidence)	AZOBR_p1120003	Ancestral (low confidence)
AZOLI_p30073	Ancestral (low confidence)	AZOBR_p1120004	Horizontally Transferred (high confidence)

AZOLI_p30074	Ancestral (low confidence)	AZOBR_p1120005	Ancestral (medium confidence)
AZOLI_p30075	Ancestral (low confidence)	AZOBR_p1120006	Unassigned
AZOLI_p30076	Ancestral (low confidence)	AZOBR_p1120007	Ancestral (low confidence)
AZOLI_p30077	Horizontally Transferred (high confidence)	AZOBR_p1120008	Ancestral (medium confidence)
AZOLI_p30078	Ancestral (low confidence)	AZOBR_p1120009	Ancestral (medium confidence)
AZOLI_p30079	Unassigned	AZOBR_p1120010	Ancestral (medium confidence)
AZOLI_p30080	Ancestral (low confidence)	AZOBR_p1120011	Horizontally Transferred (high confidence)
AZOLI_p30081	Horizontally Transferred (high confidence)	AZOBR_p1120012	Ancestral (low confidence)
AZOLI_p30082	Horizontally Transferred (high confidence)	AZOBR_p1120013	Ancestral (low confidence)
AZOLI_p30083	Ancestral (medium confidence)	AZOBR_p1120014	Ancestral (low confidence)
AZOLI_p30084	Ancestral (medium confidence)	AZOBR_p1120015	Unassigned
AZOLI_p30085	Ancestral (low confidence)	AZOBR_p1120016	Ancestral (low confidence)
AZOLI_p30086	Ancestral (medium confidence)	AZOBR_p1120017	Horizontally Transferred (high confidence)
AZOLI_p30087	Horizontally Transferred (high confidence)	AZOBR_p1120018	Unassigned
AZOLI_p30089	Ancestral (medium confidence)	AZOBR_p1120019	Unassigned
AZOLI_p30090	Unassigned	AZOBR_p1120020	Ancestral (high confidence)
AZOLI_p30091	Unassigned	AZOBR_p1120021	Ancestral (high confidence)
AZOLI_p30092	Horizontally Transferred (high confidence)	AZOBR_p1120022	Ancestral (high confidence)
AZOLI_p30093	Horizontally Transferred (high confidence)	AZOBR_p1120023	Ancestral (high confidence)
AZOLI_p30094	Ancestral (high confidence)	AZOBR_p1120024	Unassigned
AZOLI_p30095	Ancestral (low confidence)	AZOBR_p1120025	Ancestral (high confidence)
AZOLI_p30096	Horizontally Transferred (low confidence)	AZOBR_p1120026	Ancestral (high confidence)
AZOLI_p30098	Ancestral (low confidence)	AZOBR_p1120027	Ancestral (high confidence)
AZOLI_p30099	Horizontally Transferred (high confidence)	AZOBR_p1120028	Ancestral (low confidence)
AZOLI_p30100	Horizontally Transferred (high confidence)	AZOBR_p1120029	Ancestral (medium confidence)
AZOLI_p30101	Horizontally Transferred (high confidence)	AZOBR_p1120030	Unassigned
AZOLI_p30102	Horizontally Transferred (high confidence)	AZOBR_p1120031	Horizontally Transferred (high confidence)
AZOLI_p30103	Horizontally Transferred (high confidence)	AZOBR_p1120032	Horizontally Transferred (high confidence)
AZOLI_p30104	Unassigned	AZOBR_p1120033	Unassigned
AZOLI_p30105	Ancestral (low confidence)	AZOBR_p1120034	Horizontally Transferred (high confidence)
AZOLI_p30106	Ancestral (medium confidence)	AZOBR_p1120035	Ancestral (medium confidence)
AZOLI_p30107	Ancestral (medium confidence)	AZOBR_p1120036	Ancestral (low confidence)
AZOLI_p30108	Ancestral (medium confidence)	AZOBR_p1120037	Ancestral (low confidence)
AZOLI_p30109	Ancestral (medium confidence)	AZOBR_p1120038	Ancestral (low confidence)
AZOLI_p30110	Ancestral (low confidence)	AZOBR_p1120039	Horizontally Transferred (high confidence)
AZOLI_p30111	Unassigned	AZOBR_p1120040	Unassigned
AZOLI_p30112	Ancestral (medium confidence)	AZOBR_p1120041	Ancestral (low confidence)
AZOLI_p30113	Ancestral (medium confidence)	AZOBR_p1120042	Unassigned
AZOLI_p30114	Ancestral (medium confidence)	AZOBR_p1120043	Horizontally Transferred (high confidence)
AZOLI_p30115	Ancestral (low confidence)	AZOBR_p1120044	Horizontally Transferred (high confidence)
AZOLI_p30116	Unassigned	AZOBR_p1120045	Horizontally Transferred (high confidence)
AZOLI_p30117	Horizontally Transferred (high confidence)	AZOBR_p1120046	Unassigned
AZOLI_p30118	Ancestral (high confidence)	AZOBR_p1120047	Horizontally Transferred (medium confidence)
AZOLI_p30119	Unassigned	AZOBR_p1120048	Unassigned
AZOLI_p30120	Horizontally Transferred (medium confidence)	AZOBR_p1120049	Ancestral (low confidence)
AZOLI_p30121	Horizontally Transferred (high confidence)	AZOBR_p1120050	Ancestral (low confidence)
AZOLI_p30122	Horizontally Transferred (high confidence)	AZOBR_p1120051	Ancestral (low confidence)
AZOLI_p30123	Ancestral (medium confidence)	AZOBR_p1120052	Ancestral (low confidence)
AZOLI_p30124	Horizontally Transferred (high confidence)	AZOBR_p1120053	Unassigned
AZOLI_p30125	Horizontally Transferred (high confidence)	AZOBR_p1120054	Horizontally Transferred (high confidence)
AZOLI_p30126	Ancestral (medium confidence)	AZOBR_p1120055	Unassigned
AZOLI_p30127	Horizontally Transferred (high confidence)	AZOBR_p1120056	Ancestral (low confidence)
AZOLI_p30128	Ancestral (medium confidence)	AZOBR_p1120057	Unassigned
AZOLI_p30129	Ancestral (low confidence)	AZOBR_p1120058	Horizontally Transferred (high confidence)
AZOLI_p30130	Horizontally Transferred (high confidence)	AZOBR_p1120059	Horizontally Transferred (high confidence)
AZOLI_p30131	Horizontally Transferred (high confidence)	AZOBR_p1120060	Ancestral (medium confidence)
AZOLI_p30132	Horizontally Transferred (high confidence)	AZOBR_p1120061	Ancestral (medium confidence)
AZOLI_p30133	Unassigned	AZOBR_p1120062	Horizontally Transferred (high confidence)
AZOLI_p30135	Ancestral (high confidence)	AZOBR_p1120063	Unassigned
AZOLI_p30136	Unassigned	AZOBR_p1120064	Unassigned
AZOLI_p30137	Ancestral (medium confidence)	AZOBR_p1120065	Horizontally Transferred (high confidence)
AZOLI_p30138	Ancestral (medium confidence)	AZOBR_p1120066	Horizontally Transferred (high confidence)
AZOLI_p30139	Ancestral (low confidence)	AZOBR_p1120067	Horizontally Transferred (low confidence)
AZOLI_p30140	Ancestral (low confidence)	AZOBR_p1120068	Horizontally Transferred (high confidence)
AZOLI_p30141	Ancestral (low confidence)	AZOBR_p1120069	Horizontally Transferred (high confidence)
AZOLI_p30142	Horizontally Transferred (medium confidence)	AZOBR_p1120070	Horizontally Transferred (high confidence)
AZOLI_p30143	Horizontally Transferred (high confidence)	AZOBR_p1120071	Horizontally Transferred (high confidence)
AZOLI_p30144	Ancestral (low confidence)	AZOBR_p1120072	Unassigned
AZOLI_p30145	Horizontally Transferred (high confidence)	AZOBR_p1120073	Ancestral (medium confidence)
AZOLI_p30146	Horizontally Transferred (high confidence)	AZOBR_p1120074	Horizontally Transferred (high confidence)
AZOLI_p30147	Horizontally Transferred (medium confidence)	AZOBR_p1120075	Horizontally Transferred (high confidence)
AZOLI_p30148	Horizontally Transferred (medium confidence)	AZOBR_p1130001	Ancestral (medium confidence)
AZOLI_p30149	Horizontally Transferred (low confidence)	AZOBR_p1130002	Unassigned
AZOLI_p30150	Horizontally Transferred (high confidence)	AZOBR_p1130003	Horizontally Transferred (high confidence)
AZOLI_p30151	Ancestral (low confidence)	AZOBR_p1130004	Horizontally Transferred (medium confidence)

AZOLI_p30152	Ancestral (low confidence)	AZOBR_p1130005	Ancestral (low confidence)
AZOLI_p30153	Ancestral (low confidence)	AZOBR_p1130006	Ancestral (low confidence)
AZOLI_p30154	Ancestral (low confidence)	AZOBR_p1130007	Unassigned
AZOLI_p30155	Ancestral (low confidence)	AZOBR_p1130008	Ancestral (medium confidence)
AZOLI_p30156	Ancestral (low confidence)	AZOBR_p1130009	Ancestral (low confidence)
AZOLI_p30157	Horizontally Transferred (medium confidence)	AZOBR_p1130010	Ancestral (low confidence)
AZOLI_p30158	Unassigned	AZOBR_p1130011	Horizontally Transferred (high confidence)
AZOLI_p30159	Horizontally Transferred (high confidence)	AZOBR_p1130012	Horizontally Transferred (high confidence)
AZOLI_p30160	Horizontally Transferred (high confidence)	AZOBR_p1130013	Horizontally Transferred (high confidence)
AZOLI_p30161	Horizontally Transferred (medium confidence)	AZOBR_p1130014	Ancestral (medium confidence)
AZOLI_p30162	Horizontally Transferred (high confidence)	AZOBR_p1130015	Horizontally Transferred (high confidence)
AZOLI_p30163	Ancestral (medium confidence)	AZOBR_p1130016	Ancestral (low confidence)
AZOLI_p30164	Horizontally Transferred (high confidence)	AZOBR_p1130017	Horizontally Transferred (high confidence)
AZOLI_p30165	Ancestral (medium confidence)	AZOBR_p1130018	Horizontally Transferred (high confidence)
AZOLI_p30166	Ancestral (medium confidence)	AZOBR_p1130019	Horizontally Transferred (high confidence)
AZOLI_p30167	Horizontally Transferred (high confidence)	AZOBR_p1130020	Horizontally Transferred (high confidence)
AZOLI_p30168	Horizontally Transferred (high confidence)	AZOBR_p1130021	Horizontally Transferred (high confidence)
AZOLI_p30169	Horizontally Transferred (high confidence)	AZOBR_p1130022	Unassigned
AZOLI_p30170	Horizontally Transferred (high confidence)	AZOBR_p1130023	Ancestral (medium confidence)
AZOLI_p30171	Horizontally Transferred (high confidence)	AZOBR_p1130024	Ancestral (medium confidence)
AZOLI_p30172	Unassigned	AZOBR_p1130025	Horizontally Transferred (high confidence)
AZOLI_p30173	Ancestral (low confidence)	AZOBR_p1130026	Ancestral (high confidence)
AZOLI_p30174	Ancestral (high confidence)	AZOBR_p1130027	Horizontally Transferred (high confidence)
AZOLI_p30175	Ancestral (low confidence)	AZOBR_p1130028	Horizontally Transferred (high confidence)
AZOLI_p30176	Horizontally Transferred (high confidence)	AZOBR_p1130029	Ancestral (low confidence)
AZOLI_p30177	Horizontally Transferred (high confidence)	AZOBR_p1130030	Ancestral (low confidence)
AZOLI_p30178	Horizontally Transferred (high confidence)	AZOBR_p1130031	Ancestral (low confidence)
AZOLI_p30179	Unassigned	AZOBR_p1130032	Ancestral (low confidence)
AZOLI_p30180	Ancestral (low confidence)	AZOBR_p1130033	Ancestral (low confidence)
AZOLI_p30181	Unassigned	AZOBR_p1130034	Ancestral (low confidence)
AZOLI_p30182	Ancestral (high confidence)	AZOBR_p1130035	Unassigned
AZOLI_p30183	Horizontally Transferred (high confidence)	AZOBR_p1130036	Horizontally Transferred (medium confidence)
AZOLI_p30184	Ancestral (medium confidence)	AZOBR_p1130037	Unassigned
AZOLI_p30185	Ancestral (high confidence)	AZOBR_p1130038	Horizontally Transferred (high confidence)
AZOLI_p30186	Ancestral (high confidence)	AZOBR_p1130039	Ancestral (medium confidence)
AZOLI_p30187	Ancestral (medium confidence)	AZOBR_p1130040	Horizontally Transferred (high confidence)
AZOLI_p30188	Ancestral (medium confidence)	AZOBR_p1130041	Ancestral (medium confidence)
AZOLI_p30189	Unassigned	AZOBR_p1130042	Ancestral (high confidence)
AZOLI_p30190	Ancestral (high confidence)	AZOBR_p1130043	Ancestral (high confidence)
AZOLI_p30191	Ancestral (low confidence)	AZOBR_p1130044	Ancestral (high confidence)
AZOLI_p30192	Horizontally Transferred (high confidence)	AZOBR_p1130045	Ancestral (medium confidence)
AZOLI_p30193	Ancestral (medium confidence)	AZOBR_p1130046	Ancestral (medium confidence)
AZOLI_p30194	Horizontally Transferred (medium confidence)	AZOBR_p1130047	Ancestral (medium confidence)
AZOLI_p30195	Ancestral (low confidence)	AZOBR_p1130048	Ancestral (medium confidence)
AZOLI_p30196	Ancestral (low confidence)	AZOBR_p1130049	Ancestral (high confidence)
AZOLI_p30197	Ancestral (medium confidence)	AZOBR_p1130050	Ancestral (medium confidence)
AZOLI_p30198	Horizontally Transferred (high confidence)	AZOBR_p1130051	Ancestral (low confidence)
AZOLI_p30199	Unassigned	AZOBR_p1130052	Ancestral (medium confidence)
AZOLI_p30200	Ancestral (high confidence)	AZOBR_p1130053	Unassigned
AZOLI_p30201	Ancestral (high confidence)	AZOBR_p1130054	Ancestral (high confidence)
AZOLI_p30202	Horizontally Transferred (high confidence)	AZOBR_p1130055	Ancestral (medium confidence)
AZOLI_p30203	Horizontally Transferred (high confidence)	AZOBR_p1130056	Ancestral (high confidence)
AZOLI_p30205	Ancestral (high confidence)	AZOBR_p1130057	Ancestral (medium confidence)
AZOLI_p30206	Ancestral (medium confidence)	AZOBR_p1130058	Ancestral (medium confidence)
AZOLI_p30207	Ancestral (high confidence)	AZOBR_p1130059	Ancestral (medium confidence)
AZOLI_p30209	Ancestral (low confidence)	AZOBR_p1130060	Ancestral (high confidence)
AZOLI_p30210	Ancestral (medium confidence)	AZOBR_p1130061	Ancestral (high confidence)
AZOLI_p30211	Horizontally Transferred (high confidence)	AZOBR_p1130062	Ancestral (high confidence)
AZOLI_p30212	Unassigned	AZOBR_p1130063	Ancestral (high confidence)
AZOLI_p30214	Horizontally Transferred (high confidence)	AZOBR_p1130064	Ancestral (medium confidence)
AZOLI_p30215	Ancestral (high confidence)	AZOBR_p1130065	Unassigned
AZOLI_p30216	Unassigned	AZOBR_p1130066	Horizontally Transferred (high confidence)
AZOLI_p30218	Ancestral (low confidence)	AZOBR_p1130067	Ancestral (medium confidence)
AZOLI_p30219	Ancestral (medium confidence)	AZOBR_p1130068	Ancestral (high confidence)
AZOLI_p30220	Unassigned	AZOBR_p1130069	Unassigned
AZOLI_p30221	Horizontally Transferred (high confidence)	AZOBR_p1130070	Ancestral (medium confidence)
AZOLI_p30222	Ancestral (low confidence)	AZOBR_p1130071	Ancestral (low confidence)
AZOLI_p30223	Horizontally Transferred (medium confidence)	AZOBR_p1130072	Unassigned
AZOLI_p30224	Ancestral (low confidence)	AZOBR_p1130073	Ancestral (medium confidence)
AZOLI_p30225	Horizontally Transferred (high confidence)	AZOBR_p1130074	Ancestral (high confidence)
AZOLI_p30227	Horizontally Transferred (high confidence)	AZOBR_p1130075	Ancestral (high confidence)
AZOLI_p30229	Ancestral (low confidence)	AZOBR_p1130076	Ancestral (high confidence)
AZOLI_p30230	Unassigned	AZOBR_p1130077	Ancestral (high confidence)
AZOLI_p30231	Unassigned	AZOBR_p1130078	Ancestral (low confidence)
AZOLI_p30232	Horizontally Transferred (high confidence)	AZOBR_p1130079	Ancestral (high confidence)

AZOLI_p30233	Ancestral (medium confidence)	AZOBR_p1130080	Ancestral (high confidence)
AZOLI_p30234	Unassigned	AZOBR_p1130081	Unassigned
AZOLI_p30235	Horizontally Transferred (high confidence)	AZOBR_p1130082	Ancestral (high confidence)
AZOLI_p30236	Ancestral (high confidence)	AZOBR_p1130083	Ancestral (high confidence)
AZOLI_p30237	Unassigned	AZOBR_p1130084	Ancestral (low confidence)
AZOLI_p30238	Ancestral (high confidence)	AZOBR_p1130085	Unassigned
AZOLI_p30239	Ancestral (high confidence)	AZOBR_p1130086	Ancestral (high confidence)
AZOLI_p30241	Ancestral (medium confidence)	AZOBR_p1130087	Ancestral (medium confidence)
AZOLI_p30242	Horizontally Transferred (high confidence)	AZOBR_p1130088	Ancestral (high confidence)
AZOLI_p30243	Horizontally Transferred (high confidence)	AZOBR_p1130089	Ancestral (high confidence)
AZOLI_p30245	Horizontally Transferred (high confidence)	AZOBR_p1130090	Ancestral (high confidence)
AZOLI_p30246	Ancestral (medium confidence)	AZOBR_p1130091	Ancestral (medium confidence)
AZOLI_p30247	Ancestral (low confidence)	AZOBR_p1130092	Ancestral (medium confidence)
AZOLI_p30248	Horizontally Transferred (high confidence)	AZOBR_p1130093	Ancestral (high confidence)
AZOLI_p30249	Ancestral (low confidence)	AZOBR_p1130094	Ancestral (high confidence)
AZOLI_p30250	Ancestral (low confidence)	AZOBR_p1130095	Ancestral (high confidence)
AZOLI_p30251	Ancestral (low confidence)	AZOBR_p1130096	Ancestral (medium confidence)
AZOLI_p30252	Ancestral (medium confidence)	AZOBR_p1130097	Ancestral (high confidence)
AZOLI_p30253	Ancestral (medium confidence)	AZOBR_p1130098	Ancestral (medium confidence)
AZOLI_p30254	Horizontally Transferred (high confidence)	AZOBR_p1130099	Ancestral (medium confidence)
AZOLI_p30255	Ancestral (medium confidence)	AZOBR_p1130100	Ancestral (medium confidence)
AZOLI_p30256	Ancestral (medium confidence)	AZOBR_p1130101	Ancestral (medium confidence)
AZOLI_p30257	Ancestral (medium confidence)	AZOBR_p1130102	Horizontally Transferred (high confidence)
AZOLI_p30258	Ancestral (low confidence)	AZOBR_p1130103	Unassigned
AZOLI_p30259	Ancestral (low confidence)	AZOBR_p1130104	Ancestral (medium confidence)
AZOLI_p30260	Ancestral (high confidence)	AZOBR_p1130105	Ancestral (low confidence)
AZOLI_p30262	Horizontally Transferred (low confidence)	AZOBR_p1130106	Ancestral (medium confidence)
AZOLI_p30263	Ancestral (low confidence)	AZOBR_p1130107	Ancestral (medium confidence)
AZOLI_p30266	Unassigned	AZOBR_p1130108	Unassigned
AZOLI_p30267	Horizontally Transferred (high confidence)	AZOBR_p1130109	Ancestral (high confidence)
AZOLI_p30268	Horizontally Transferred (low confidence)	AZOBR_p1130110	Ancestral (medium confidence)
AZOLI_p30269	Horizontally Transferred (high confidence)	AZOBR_p1130111	Unassigned
AZOLI_p30270	Ancestral (high confidence)	AZOBR_p1130112	Ancestral (low confidence)
AZOLI_p30271	Horizontally Transferred (medium confidence)	AZOBR_p1130113	Unassigned
AZOLI_p30272	Unassigned	AZOBR_p1130114	Ancestral (medium confidence)
AZOLI_p30273	Horizontally Transferred (medium confidence)	AZOBR_p1130115	Ancestral (medium confidence)
AZOLI_p30274	Horizontally Transferred (high confidence)	AZOBR_p1130116	Unassigned
AZOLI_p30275	Horizontally Transferred (high confidence)	AZOBR_p1130117	Ancestral (high confidence)
AZOLI_p30276	Horizontally Transferred (high confidence)	AZOBR_p1130118	Ancestral (medium confidence)
AZOLI_p30277	Horizontally Transferred (medium confidence)	AZOBR_p1130119	Ancestral (high confidence)
AZOLI_p30278	Unassigned	AZOBR_p1130120	Ancestral (high confidence)
AZOLI_p30279	Horizontally Transferred (high confidence)	AZOBR_p1130121	Ancestral (medium confidence)
AZOLI_p30280	Ancestral (low confidence)	AZOBR_p1130122	Unassigned
AZOLI_p30281	Ancestral (low confidence)	AZOBR_p1130123	Ancestral (medium confidence)
AZOLI_p30282	Ancestral (medium confidence)	AZOBR_p1130124	Ancestral (high confidence)
AZOLI_p30283	Horizontally Transferred (high confidence)	AZOBR_p1130125	Ancestral (high confidence)
AZOLI_p30284	Horizontally Transferred (high confidence)	AZOBR_p1130126	Ancestral (high confidence)
AZOLI_p30285	Horizontally Transferred (high confidence)	AZOBR_p1130127	Ancestral (medium confidence)
AZOLI_p30286	Horizontally Transferred (medium confidence)	AZOBR_p1130128	Ancestral (high confidence)
AZOLI_p30287	Horizontally Transferred (high confidence)	AZOBR_p1130129	Ancestral (high confidence)
AZOLI_p30288	Horizontally Transferred (high confidence)	AZOBR_p1130130	Ancestral (medium confidence)
AZOLI_p30290	Horizontally Transferred (high confidence)	AZOBR_p1130131	Ancestral (low confidence)
AZOLI_p30291	Horizontally Transferred (high confidence)	AZOBR_p1130132	Ancestral (medium confidence)
AZOLI_p30292	Unassigned	AZOBR_p1130133	Ancestral (medium confidence)
AZOLI_p30293	Horizontally Transferred (high confidence)	AZOBR_p1130134	Horizontally Transferred (high confidence)
AZOLI_p30294	Horizontally Transferred (high confidence)	AZOBR_p1130135	Ancestral (medium confidence)
AZOLI_p30295	Horizontally Transferred (high confidence)	AZOBR_p1130136	Unassigned
AZOLI_p30296	Horizontally Transferred (high confidence)	AZOBR_p1130137	Ancestral (medium confidence)
AZOLI_p30297	Ancestral (low confidence)	AZOBR_p1130138	Horizontally Transferred (high confidence)
AZOLI_p30298	Unassigned	AZOBR_p1130139	Horizontally Transferred (medium confidence)
AZOLI_p30299	Unassigned	AZOBR_p1130140	Unassigned
AZOLI_p30300	Horizontally Transferred (high confidence)	AZOBR_p1130141	Horizontally Transferred (high confidence)
AZOLI_p30301	Horizontally Transferred (low confidence)	AZOBR_p1130142	Unassigned
AZOLI_p30302	Ancestral (medium confidence)	AZOBR_p1130143	Unassigned
AZOLI_p30303	Ancestral (low confidence)	AZOBR_p1130144	Unassigned
AZOLI_p30305	Ancestral (medium confidence)	AZOBR_p1130145	Ancestral (low confidence)
AZOLI_p30306	Ancestral (medium confidence)	AZOBR_p1130146	Ancestral (low confidence)
AZOLI_p30308	Horizontally Transferred (high confidence)	AZOBR_p1130147	Ancestral (low confidence)
AZOLI_p30309	Horizontally Transferred (high confidence)	AZOBR_p1130148	Ancestral (low confidence)
AZOLI_p30310	Horizontally Transferred (high confidence)	AZOBR_p1130149	Ancestral (high confidence)
AZOLI_p30311	Ancestral (low confidence)	AZOBR_p1130150	Unassigned
AZOLI_p30312	Horizontally Transferred (high confidence)	AZOBR_p1130151	Ancestral (low confidence)
AZOLI_p30313	Ancestral (high confidence)	AZOBR_p1130152	Horizontally Transferred (high confidence)
AZOLI_p30314	Ancestral (medium confidence)	AZOBR_p1130153	Horizontally Transferred (medium confidence)
AZOLI_p30315	Ancestral (medium confidence)	AZOBR_p1130154	Ancestral (medium confidence)

AZOLI_p30317	Horizontally Transferred (high confidence)	AZOBR_p1130155	Ancestral (high confidence)
AZOLI_p30318	Horizontally Transferred (medium confidence)	AZOBR_p1130156	Ancestral (high confidence)
AZOLI_p30319	Horizontally Transferred (high confidence)	AZOBR_p1130157	Ancestral (high confidence)
AZOLI_p30320	Ancestral (medium confidence)	AZOBR_p1130158	Horizontally Transferred (high confidence)
AZOLI_p30321	Horizontally Transferred (high confidence)	AZOBR_p1130159	Ancestral (medium confidence)
AZOLI_p30322	Horizontally Transferred (high confidence)	AZOBR_p1130160	Ancestral (high confidence)
AZOLI_p30323	Ancestral (low confidence)	AZOBR_p1130161	Ancestral (high confidence)
AZOLI_p30325	Unassigned	AZOBR_p1130162	Ancestral (high confidence)
AZOLI_p30326	Unassigned	AZOBR_p1130163	Ancestral (high confidence)
AZOLI_p30327	Horizontally Transferred (high confidence)	AZOBR_p1130164	Ancestral (high confidence)
AZOLI_p30328	Ancestral (high confidence)	AZOBR_p1130165	Unassigned
AZOLI_p30329	Ancestral (medium confidence)	AZOBR_p1130166	Horizontally Transferred (high confidence)
AZOLI_p30330	Ancestral (low confidence)	AZOBR_p1130167	Unassigned
AZOLI_p30331	Ancestral (medium confidence)	AZOBR_p1130168	Ancestral (medium confidence)
AZOLI_p30332	Horizontally Transferred (high confidence)	AZOBR_p1130169	Ancestral (medium confidence)
AZOLI_p30333	Unassigned	AZOBR_p1130170	Ancestral (medium confidence)
AZOLI_p30334	Horizontally Transferred (high confidence)	AZOBR_p1130171	Horizontally Transferred (high confidence)
AZOLI_p30335	Horizontally Transferred (high confidence)	AZOBR_p1130172	Horizontally Transferred (high confidence)
AZOLI_p30336	Unassigned	AZOBR_p1130173	Horizontally Transferred (high confidence)
AZOLI_p30338	Horizontally Transferred (low confidence)	AZOBR_p1130174	Horizontally Transferred (high confidence)
AZOLI_p30339	Horizontally Transferred (high confidence)	AZOBR_p1130175	Horizontally Transferred (high confidence)
AZOLI_p30340	Ancestral (medium confidence)	AZOBR_p1130176	Ancestral (low confidence)
AZOLI_p30341	Ancestral (low confidence)	AZOBR_p1130177	Unassigned
AZOLI_p30342	Ancestral (medium confidence)	AZOBR_p1130178	Horizontally Transferred (high confidence)
AZOLI_p30343	Ancestral (high confidence)	AZOBR_p1130179	Horizontally Transferred (high confidence)
AZOLI_p30344	Ancestral (medium confidence)	AZOBR_p1130180	Ancestral (high confidence)
AZOLI_p30345	Ancestral (high confidence)	AZOBR_p1130181	Horizontally Transferred (high confidence)
AZOLI_p30347	Ancestral (high confidence)	AZOBR_p1130182	Horizontally Transferred (high confidence)
AZOLI_p30349	Ancestral (low confidence)	AZOBR_p1130183	Ancestral (high confidence)
AZOLI_p30350	Ancestral (low confidence)	AZOBR_p1130184	Ancestral (high confidence)
AZOLI_p30351	Ancestral (high confidence)	AZOBR_p1130185	Horizontally Transferred (high confidence)
AZOLI_p30352	Ancestral (low confidence)	AZOBR_p1130186	Horizontally Transferred (high confidence)
AZOLI_p30353	Ancestral (medium confidence)	AZOBR_p1130187	Ancestral (low confidence)
AZOLI_p30354	Ancestral (high confidence)	AZOBR_p1130188	Ancestral (medium confidence)
AZOLI_p30355	Ancestral (medium confidence)	AZOBR_p1130189	Horizontally Transferred (high confidence)
AZOLI_p30356	Ancestral (medium confidence)	AZOBR_p1130190	Unassigned
AZOLI_p30357	Ancestral (low confidence)	AZOBR_p1130191	Unassigned
AZOLI_p30358	Ancestral (medium confidence)	AZOBR_p1130192	Unassigned
AZOLI_p30359	Horizontally Transferred (high confidence)	AZOBR_p1130193	Ancestral (medium confidence)
AZOLI_p30360	Ancestral (medium confidence)	AZOBR_p1130194	Ancestral (high confidence)
AZOLI_p30361	Ancestral (high confidence)	AZOBR_p1130195	Horizontally Transferred (high confidence)
AZOLI_p30362	Unassigned	AZOBR_p1130196	Ancestral (high confidence)
AZOLI_p30363	Horizontally Transferred (high confidence)	AZOBR_p1130197	Ancestral (medium confidence)
AZOLI_p30364	Horizontally Transferred (high confidence)	AZOBR_p1130198	Ancestral (medium confidence)
AZOLI_p30365	Ancestral (medium confidence)	AZOBR_p1130199	Ancestral (high confidence)
AZOLI_p30366	Ancestral (low confidence)	AZOBR_p1130200	Ancestral (low confidence)
AZOLI_p30367	Horizontally Transferred (high confidence)	AZOBR_p1130201	Unassigned
AZOLI_p30368	Horizontally Transferred (high confidence)	AZOBR_p1130202	Horizontally Transferred (high confidence)
AZOLI_p30369	Ancestral (medium confidence)	AZOBR_p1130203	Unassigned
AZOLI_p30370	Ancestral (medium confidence)	AZOBR_p1130204	Horizontally Transferred (high confidence)
AZOLI_p30371	Horizontally Transferred (high confidence)	AZOBR_p1130205	Unassigned
AZOLI_p30372	Horizontally Transferred (high confidence)	AZOBR_p1130206	Unassigned
AZOLI_p30373	Horizontally Transferred (high confidence)	AZOBR_p1130207	Ancestral (medium confidence)
AZOLI_p30374	Horizontally Transferred (high confidence)	AZOBR_p1130208	Ancestral (low confidence)
AZOLI_p30375	Unassigned	AZOBR_p1140001	Ancestral (low confidence)
AZOLI_p30376	Horizontally Transferred (medium confidence)	AZOBR_p1140002	Ancestral (high confidence)
AZOLI_p30377	Ancestral (medium confidence)	AZOBR_p1140003	Ancestral (high confidence)
AZOLI_p30378	Ancestral (low confidence)	AZOBR_p1140004	Ancestral (high confidence)
AZOLI_p30379	Ancestral (low confidence)	AZOBR_p1140005	Ancestral (medium confidence)
AZOLI_p30380	Ancestral (low confidence)	AZOBR_p1140006	Unassigned
AZOLI_p30381	Ancestral (low confidence)	AZOBR_p1140007	Ancestral (high confidence)
AZOLI_p30382	Ancestral (low confidence)	AZOBR_p1140008	Ancestral (medium confidence)
AZOLI_p30383	Ancestral (medium confidence)	AZOBR_p1140009	Ancestral (medium confidence)
AZOLI_p30384	Horizontally Transferred (low confidence)	AZOBR_p1140010	Ancestral (high confidence)
AZOLI_p30385	Ancestral (low confidence)	AZOBR_p1140011	Ancestral (high confidence)
AZOLI_p30386	Ancestral (low confidence)	AZOBR_p1140012	Ancestral (medium confidence)
AZOLI_p30387	Horizontally Transferred (medium confidence)	AZOBR_p1140013	Ancestral (low confidence)
AZOLI_p30388	Unassigned	AZOBR_p1140014	Unassigned
AZOLI_p30390	Horizontally Transferred (high confidence)	AZOBR_p1140015	Unassigned
AZOLI_p30392	Horizontally Transferred (low confidence)	AZOBR_p1140016	Ancestral (high confidence)
AZOLI_p30393	Horizontally Transferred (high confidence)	AZOBR_p1140017	Unassigned
AZOLI_p30394	Horizontally Transferred (high confidence)	AZOBR_p1140018	Unassigned
AZOLI_p30395	Horizontally Transferred (high confidence)	AZOBR_p1140019	Ancestral (medium confidence)
AZOLI_p30396	Ancestral (low confidence)	AZOBR_p1140020	Ancestral (high confidence)
AZOLI_p30397	Ancestral (low confidence)	AZOBR_p1140021	Ancestral (medium confidence)

AZOLI_p30398	Horizontally Transferred (high confidence)	AZOBR_p1140022	Ancestral (medium confidence)
AZOLI_p30399	Horizontally Transferred (high confidence)	AZOBR_p1140023	Horizontally Transferred (high confidence)
AZOLI_p30400	Horizontally Transferred (high confidence)	AZOBR_p1140024	Ancestral (high confidence)
AZOLI_p30401	Horizontally Transferred (high confidence)	AZOBR_p1140025	Ancestral (low confidence)
AZOLI_p30402	Horizontally Transferred (high confidence)	AZOBR_p1140026	Horizontally Transferred (low confidence)
AZOLI_p30403	Horizontally Transferred (high confidence)	AZOBR_p1140027	Horizontally Transferred (high confidence)
AZOLI_p30404	Horizontally Transferred (high confidence)	AZOBR_p1140028	Unassigned
AZOLI_p30405	Ancestral (low confidence)	AZOBR_p1140029	Horizontally Transferred (medium confidence)
AZOLI_p30406	Horizontally Transferred (high confidence)	AZOBR_p1140030	Unassigned
AZOLI_p30407	Ancestral (medium confidence)	AZOBR_p1140031	Ancestral (low confidence)
AZOLI_p30408	Horizontally Transferred (high confidence)	AZOBR_p1140032	Unassigned
AZOLI_p30410	Horizontally Transferred (high confidence)	AZOBR_p1140033	Unassigned
AZOLI_p30413	Horizontally Transferred (high confidence)	AZOBR_p1140034	Unassigned
AZOLI_p30415	Ancestral (low confidence)	AZOBR_p1140035	Unassigned
AZOLI_p30417	Ancestral (low confidence)	AZOBR_p1140036	Horizontally Transferred (high confidence)
AZOLI_p30418	Horizontally Transferred (low confidence)	AZOBR_p1140037	Horizontally Transferred (high confidence)
AZOLI_p30419	Horizontally Transferred (low confidence)	AZOBR_p1140038	Horizontally Transferred (high confidence)
AZOLI_p30420	Ancestral (low confidence)	AZOBR_p1140039	Unassigned
AZOLI_p30421	Unassigned	AZOBR_p1140040	Unassigned
AZOLI_p30422	Ancestral (low confidence)	AZOBR_p1140041	Unassigned
AZOLI_p30423	Horizontally Transferred (high confidence)	AZOBR_p1140042	Horizontally Transferred (low confidence)
AZOLI_p30424	Unassigned	AZOBR_p1140043	Horizontally Transferred (high confidence)
AZOLI_p30425	Unassigned	AZOBR_p1140044	Horizontally Transferred (high confidence)
AZOLI_p30426	Unassigned	AZOBR_p1140045	Horizontally Transferred (low confidence)
AZOLI_p30427	Unassigned	AZOBR_p1140046	Horizontally Transferred (high confidence)
AZOLI_p30428	Horizontally Transferred (high confidence)	AZOBR_p1140047	Unassigned
AZOLI_p30429	Ancestral (medium confidence)	AZOBR_p1140048	Horizontally Transferred (high confidence)
AZOLI_p30430	Horizontally Transferred (medium confidence)	AZOBR_p1140049	Horizontally Transferred (high confidence)
AZOLI_p30431	Unassigned	AZOBR_p1140050	Unassigned
AZOLI_p30432	Horizontally Transferred (high confidence)	AZOBR_p1140051	Unassigned
AZOLI_p30433	Ancestral (high confidence)	AZOBR_p1140052	Unassigned
AZOLI_p30434	Ancestral (high confidence)	AZOBR_p1140053	Unassigned
AZOLI_p30435	Ancestral (high confidence)	AZOBR_p1140054	Unassigned
AZOLI_p30436	Ancestral (medium confidence)	AZOBR_p1140055	Horizontally Transferred (high confidence)
AZOLI_p30437	Horizontally Transferred (high confidence)	AZOBR_p1140056	Horizontally Transferred (high confidence)
AZOLI_p30438	Ancestral (low confidence)	AZOBR_p1140057	Unassigned
AZOLI_p30439	Horizontally Transferred (high confidence)	AZOBR_p1140058	Horizontally Transferred (high confidence)
AZOLI_p30441	Horizontally Transferred (medium confidence)	AZOBR_p1140059	Horizontally Transferred (high confidence)
AZOLI_p30442	Ancestral (high confidence)	AZOBR_p1140060	Ancestral (low confidence)
AZOLI_p30443	Ancestral (low confidence)	AZOBR_p1140061	Unassigned
AZOLI_p30444	Horizontally Transferred (high confidence)	AZOBR_p1140062	Unassigned
AZOLI_p30445	Horizontally Transferred (high confidence)	AZOBR_p1140063	Unassigned
AZOLI_p30446	Unassigned	AZOBR_p1140064	Horizontally Transferred (medium confidence)
AZOLI_p30447	Horizontally Transferred (high confidence)	AZOBR_p1140065	Horizontally Transferred (medium confidence)
AZOLI_p30448	Horizontally Transferred (high confidence)	AZOBR_p1140066	Unassigned
AZOLI_p30449	Ancestral (low confidence)	AZOBR_p1140067	Horizontally Transferred (high confidence)
AZOLI_p30450	Ancestral (low confidence)	AZOBR_p1140068	Ancestral (low confidence)
AZOLI_p30451	Ancestral (medium confidence)	AZOBR_p1140069	Unassigned
AZOLI_p30452	Ancestral (low confidence)	AZOBR_p1140070	Horizontally Transferred (high confidence)
AZOLI_p30453	Ancestral (low confidence)	AZOBR_p1140071	Ancestral (low confidence)
AZOLI_p30455	Ancestral (low confidence)	AZOBR_p1140072	Unassigned
AZOLI_p30456	Horizontally Transferred (high confidence)	AZOBR_p1140073	Unassigned
AZOLI_p30457	Ancestral (medium confidence)	AZOBR_p1140074	Horizontally Transferred (medium confidence)
AZOLI_p30458	Ancestral (medium confidence)	AZOBR_p1140075	Unassigned
AZOLI_p30459	Horizontally Transferred (high confidence)	AZOBR_p1140076	Ancestral (low confidence)
AZOLI_p30460	Unassigned	AZOBR_p1140077	Ancestral (low confidence)
AZOLI_p30461	Ancestral (low confidence)	AZOBR_p1140078	Ancestral (medium confidence)
AZOLI_p30462	Ancestral (low confidence)	AZOBR_p1140079	Horizontally Transferred (high confidence)
AZOLI_p30464	Horizontally Transferred (high confidence)	AZOBR_p1140080	Horizontally Transferred (high confidence)
AZOLI_p30465	Ancestral (medium confidence)	AZOBR_p1140081	Ancestral (medium confidence)
AZOLI_p30466	Horizontally Transferred (low confidence)	AZOBR_p1140082	Horizontally Transferred (high confidence)
AZOLI_p30468	Unassigned	AZOBR_p1140083	Unassigned
AZOLI_p30469	Horizontally Transferred (high confidence)	AZOBR_p1140084	Horizontally Transferred (medium confidence)
AZOLI_p30470	Horizontally Transferred (high confidence)	AZOBR_p1140085	Unassigned
AZOLI_p30471	Ancestral (medium confidence)	AZOBR_p1140086	Horizontally Transferred (high confidence)
AZOLI_p30472	Ancestral (low confidence)	AZOBR_p1140087	Horizontally Transferred (medium confidence)
AZOLI_p30473	Horizontally Transferred (high confidence)	AZOBR_p1140088	Ancestral (low confidence)
AZOLI_p30474	Horizontally Transferred (high confidence)	AZOBR_p1140089	Ancestral (low confidence)
AZOLI_p30475	Unassigned	AZOBR_p1140090	Ancestral (low confidence)
AZOLI_p30476	Horizontally Transferred (high confidence)	AZOBR_p1140091	Horizontally Transferred (high confidence)
AZOLI_p30477	Horizontally Transferred (high confidence)	AZOBR_p1140092	Horizontally Transferred (high confidence)
AZOLI_p30478	Unassigned	AZOBR_p1140093	Horizontally Transferred (medium confidence)
AZOLI_p30479	Ancestral (medium confidence)	AZOBR_p1140094	Unassigned
AZOLI_p30480	Ancestral (low confidence)	AZOBR_p1140095	Horizontally Transferred (high confidence)
AZOLI_p30482	Horizontally Transferred (low confidence)	AZOBR_p1140096	Horizontally Transferred (high confidence)

AZOLI_p30483	Horizontally Transferred (medium confidence)	AZOBR_p1140097	Horizontally Transferred (high confidence)
AZOLI_p30484	Unassigned	AZOBR_p1140098	Horizontally Transferred (medium confidence)
AZOLI_p30485	Horizontally Transferred (high confidence)	AZOBR_p1140099	Horizontally Transferred (high confidence)
AZOLI_p30486	Horizontally Transferred (high confidence)	AZOBR_p1140100	Horizontally Transferred (high confidence)
AZOLI_p30487	Horizontally Transferred (high confidence)	AZOBR_p1140101	Horizontally Transferred (high confidence)
AZOLI_p30488	Horizontally Transferred (low confidence)	AZOBR_p1140102	Ancestral (high confidence)
AZOLI_p30489	Horizontally Transferred (high confidence)	AZOBR_p1140103	Ancestral (high confidence)
AZOLI_p30490	Horizontally Transferred (high confidence)	AZOBR_p1140104	Ancestral (high confidence)
AZOLI_p30491	Ancestral (medium confidence)	AZOBR_p1140105	Horizontally Transferred (high confidence)
AZOLI_p30492	Ancestral (high confidence)	AZOBR_p1140106	Ancestral (medium confidence)
AZOLI_p30493	Unassigned	AZOBR_p1140107	Ancestral (low confidence)
AZOLI_p30494	Ancestral (low confidence)	AZOBR_p1140108	Horizontally Transferred (high confidence)
AZOLI_p30495	Ancestral (medium confidence)	AZOBR_p1140109	Horizontally Transferred (high confidence)
AZOLI_p30496	Unassigned	AZOBR_p1140110	Horizontally Transferred (high confidence)
AZOLI_p30497	Ancestral (low confidence)	AZOBR_p1140111	Horizontally Transferred (high confidence)
AZOLI_p30498	Ancestral (low confidence)	AZOBR_p1140112	Ancestral (medium confidence)
AZOLI_p30499	Ancestral (low confidence)	AZOBR_p1140113	Ancestral (low confidence)
AZOLI_p30500	Ancestral (medium confidence)	AZOBR_p1140114	Horizontally Transferred (high confidence)
AZOLI_p30501	Ancestral (high confidence)	AZOBR_p1140115	Horizontally Transferred (medium confidence)
AZOLI_p30502	Ancestral (high confidence)	AZOBR_p1140116	Horizontally Transferred (low confidence)
AZOLI_p30503	Ancestral (high confidence)	AZOBR_p1140117	Horizontally Transferred (medium confidence)
AZOLI_p30504	Ancestral (low confidence)	AZOBR_p1140118	Horizontally Transferred (medium confidence)
AZOLI_p30505	Unassigned	AZOBR_p1140119	Horizontally Transferred (high confidence)
AZOLI_p30506	Ancestral (low confidence)	AZOBR_p1140120	Horizontally Transferred (high confidence)
AZOLI_p30507	Ancestral (medium confidence)	AZOBR_p1140121	Ancestral (high confidence)
AZOLI_p30508	Ancestral (high confidence)	AZOBR_p1140122	Ancestral (high confidence)
AZOLI_p30509	Ancestral (high confidence)	AZOBR_p1140123	Ancestral (low confidence)
AZOLI_p30510	Ancestral (medium confidence)	AZOBR_p1140124	Ancestral (low confidence)
AZOLI_p30511	Ancestral (medium confidence)	AZOBR_p1140125	Horizontally Transferred (medium confidence)
AZOLI_p30512	Ancestral (high confidence)	AZOBR_p1140126	Horizontally Transferred (high confidence)
AZOLI_p30513	Horizontally Transferred (high confidence)	AZOBR_p1140127	Horizontally Transferred (medium confidence)
AZOLI_p30514	Horizontally Transferred (high confidence)	AZOBR_p1140128	Horizontally Transferred (high confidence)
AZOLI_p30515	Ancestral (low confidence)	AZOBR_p1140129	Horizontally Transferred (high confidence)
AZOLI_p30516	Ancestral (low confidence)	AZOBR_p1140130	Horizontally Transferred (high confidence)
AZOLI_p30517	Horizontally Transferred (high confidence)	AZOBR_p1140131	Horizontally Transferred (high confidence)
AZOLI_p30518	Horizontally Transferred (medium confidence)	AZOBR_p1140132	Horizontally Transferred (high confidence)
AZOLI_p30519	Horizontally Transferred (high confidence)	AZOBR_p1140133	Horizontally Transferred (high confidence)
AZOLI_p30520	Horizontally Transferred (low confidence)	AZOBR_p1140134	Ancestral (medium confidence)
AZOLI_p30521	Ancestral (low confidence)	AZOBR_p1140135	Unassigned
AZOLI_p30522	Ancestral (low confidence)	AZOBR_p1140136	Unassigned
AZOLI_p30523	Ancestral (low confidence)	AZOBR_p1140137	Ancestral (medium confidence)
AZOLI_p30524	Ancestral (low confidence)	AZOBR_p1150001	Unassigned
AZOLI_p30525	Ancestral (low confidence)	AZOBR_p1150002	Ancestral (medium confidence)
AZOLI_p30526	Horizontally Transferred (high confidence)	AZOBR_p1150003	Ancestral (medium confidence)
AZOLI_p30527	Ancestral (low confidence)	AZOBR_p1150004	Horizontally Transferred (high confidence)
AZOLI_p30528	Ancestral (low confidence)	AZOBR_p1150005	Horizontally Transferred (high confidence)
AZOLI_p30529	Ancestral (low confidence)	AZOBR_p1150006	Horizontally Transferred (high confidence)
AZOLI_p30530	Unassigned	AZOBR_p1150007	Horizontally Transferred (high confidence)
AZOLI_p30531	Ancestral (low confidence)	AZOBR_p1150008	Horizontally Transferred (high confidence)
AZOLI_p30532	Ancestral (high confidence)	AZOBR_p1150009	Horizontally Transferred (high confidence)
AZOLI_p30533	Horizontally Transferred (high confidence)	AZOBR_p1150010	Horizontally Transferred (high confidence)
AZOLI_p30534	Horizontally Transferred (high confidence)	AZOBR_p1150011	Horizontally Transferred (high confidence)
AZOLI_p30535	Horizontally Transferred (high confidence)	AZOBR_p1150012	Horizontally Transferred (high confidence)
AZOLI_p30536	Horizontally Transferred (high confidence)	AZOBR_p1150013	Horizontally Transferred (medium confidence)
AZOLI_p30537	Horizontally Transferred (high confidence)	AZOBR_p1150014	Ancestral (medium confidence)
AZOLI_p30538	Ancestral (low confidence)	AZOBR_p1150015	Ancestral (low confidence)
AZOLI_p30540	Unassigned	AZOBR_p1150016	Ancestral (low confidence)
AZOLI_p30541	Horizontally Transferred (high confidence)	AZOBR_p1150017	Horizontally Transferred (high confidence)
AZOLI_p30542	Horizontally Transferred (high confidence)	AZOBR_p1150018	Horizontally Transferred (medium confidence)
AZOLI_p30543	Horizontally Transferred (high confidence)	AZOBR_p1150019	Horizontally Transferred (high confidence)
AZOLI_p30544	Unassigned	AZOBR_p1150020	Horizontally Transferred (high confidence)
AZOLI_p30545	Horizontally Transferred (high confidence)	AZOBR_p1150021	Ancestral (high confidence)
AZOLI_p30546	Horizontally Transferred (high confidence)	AZOBR_p1150022	Ancestral (medium confidence)
AZOLI_p30547	Unassigned	AZOBR_p1150023	Unassigned
AZOLI_p30548	Unassigned	AZOBR_p1150026	Horizontally Transferred (high confidence)
AZOLI_p30549	Ancestral (medium confidence)	AZOBR_p1150027	Horizontally Transferred (high confidence)
AZOLI_p30550	Unassigned	AZOBR_p1160001	Horizontally Transferred (high confidence)
AZOLI_p30551	Horizontally Transferred (high confidence)	AZOBR_p1160002	Unassigned
AZOLI_p30552	Horizontally Transferred (high confidence)	AZOBR_p1160003	Unassigned
AZOLI_p30555	Unassigned	AZOBR_p1160004	Ancestral (low confidence)
AZOLI_p30556	Ancestral (low confidence)	AZOBR_p1160005	Ancestral (low confidence)
AZOLI_p30557	Ancestral (low confidence)	AZOBR_p1160006	Ancestral (medium confidence)
AZOLI_p30558	Ancestral (low confidence)	AZOBR_p1160007	Ancestral (medium confidence)
AZOLI_p30559	Ancestral (low confidence)	AZOBR_p1160008	Ancestral (low confidence)
AZOLI_p30560	Unassigned	AZOBR_p1160009	Unassigned

AZOLI_p30561	Ancestral (medium confidence)	AZOBR_p1160010	Horizontally Transferred (high confidence)
AZOLI_p30562	Horizontally Transferred (high confidence)	AZOBR_p1160011	Horizontally Transferred (high confidence)
AZOLI_p30563	Ancestral (low confidence)	AZOBR_p1160012	Ancestral (low confidence)
AZOLI_p30564	Ancestral (medium confidence)	AZOBR_p1160013	Ancestral (medium confidence)
AZOLI_p30565	Ancestral (low confidence)	AZOBR_p1160014	Horizontally Transferred (high confidence)
AZOLI_p30566	Ancestral (low confidence)	AZOBR_p1160015	Horizontally Transferred (high confidence)
AZOLI_p30567	Ancestral (medium confidence)	AZOBR_p1160016	Unassigned
AZOLI_p30568	Ancestral (medium confidence)	AZOBR_p1160017	Ancestral (medium confidence)
AZOLI_p30569	Horizontally Transferred (high confidence)	AZOBR_p1160018	Ancestral (low confidence)
AZOLI_p30570	Unassigned	AZOBR_p1160019	Ancestral (medium confidence)
AZOLI_p30571	Ancestral (medium confidence)	AZOBR_p1160020	Ancestral (medium confidence)
AZOLI_p30572	Ancestral (low confidence)	AZOBR_p1160021	Ancestral (medium confidence)
AZOLI_p30573	Unassigned	AZOBR_p1160022	Ancestral (medium confidence)
AZOLI_p30574	Horizontally Transferred (high confidence)	AZOBR_p1160023	Ancestral (medium confidence)
AZOLI_p30575	Ancestral (low confidence)	AZOBR_p1160024	Ancestral (medium confidence)
AZOLI_p30576	Ancestral (low confidence)	AZOBR_p1160025	Ancestral (low confidence)
AZOLI_p30577	Horizontally Transferred (high confidence)	AZOBR_p1160026	Horizontally Transferred (high confidence)
AZOLI_p30578	Horizontally Transferred (high confidence)	AZOBR_p1160027	Horizontally Transferred (high confidence)
AZOLI_p30579	Horizontally Transferred (high confidence)	AZOBR_p1160028	Horizontally Transferred (high confidence)
AZOLI_p30580	Horizontally Transferred (high confidence)	AZOBR_p1160029	Horizontally Transferred (high confidence)
AZOLI_p30581	Horizontally Transferred (high confidence)	AZOBR_p1160030	Horizontally Transferred (high confidence)
AZOLI_p30582	Horizontally Transferred (medium confidence)	AZOBR_p1160031	Horizontally Transferred (high confidence)
AZOLI_p30583	Horizontally Transferred (medium confidence)	AZOBR_p1160032	Horizontally Transferred (high confidence)
AZOLI_p30584	Horizontally Transferred (high confidence)	AZOBR_p1160033	Ancestral (medium confidence)
AZOLI_p30585	Horizontally Transferred (high confidence)	AZOBR_p1160034	Horizontally Transferred (medium confidence)
AZOLI_p30586	Horizontally Transferred (low confidence)	AZOBR_p1160035	Ancestral (medium confidence)
AZOLI_p30587	Horizontally Transferred (medium confidence)	AZOBR_p1160036	Horizontally Transferred (high confidence)
AZOLI_p30588	Ancestral (low confidence)	AZOBR_p1160037	Unassigned
AZOLI_p30589	Ancestral (low confidence)	AZOBR_p1160038	Ancestral (medium confidence)
AZOLI_p30590	Horizontally Transferred (high confidence)	AZOBR_p1160039	Horizontally Transferred (high confidence)
AZOLI_p30591	Ancestral (low confidence)	AZOBR_p1160040	Horizontally Transferred (low confidence)
AZOLI_p30592	Horizontally Transferred (high confidence)	AZOBR_p1160041	Horizontally Transferred (high confidence)
AZOLI_p30593	Ancestral (low confidence)	AZOBR_p1160042	Horizontally Transferred (high confidence)
AZOLI_p30594	Horizontally Transferred (high confidence)	AZOBR_p1160043	Horizontally Transferred (high confidence)
AZOLI_p30595	Horizontally Transferred (high confidence)	AZOBR_p1160044	Horizontally Transferred (high confidence)
AZOLI_p30596	Horizontally Transferred (low confidence)	AZOBR_p1160045	Unassigned
AZOLI_p30600	Unassigned	AZOBR_p1160046	Ancestral (medium confidence)
AZOLI_p30601	Horizontally Transferred (high confidence)	AZOBR_p1160047	Horizontally Transferred (high confidence)
AZOLI_p30602	Horizontally Transferred (high confidence)	AZOBR_p1160048	Unassigned
AZOLI_p40001	Horizontally Transferred (high confidence)	AZOBR_p1160049	Ancestral (medium confidence)
AZOLI_p40002	Ancestral (medium confidence)	AZOBR_p1160050	Unassigned
AZOLI_p40003	Horizontally Transferred (high confidence)	AZOBR_p1160051	Unassigned
AZOLI_p40004	Ancestral (low confidence)	AZOBR_p1160052	Unassigned
AZOLI_p40005	Ancestral (high confidence)	AZOBR_p1160053	Ancestral (medium confidence)
AZOLI_p40006	Horizontally Transferred (high confidence)	AZOBR_p1160054	Ancestral (high confidence)
AZOLI_p40007	Unassigned	AZOBR_p1160055	Ancestral (high confidence)
AZOLI_p40009	Unassigned	AZOBR_p1160056	Unassigned
AZOLI_p40010	Horizontally Transferred (medium confidence)	AZOBR_p1160057	Unassigned
AZOLI_p40011	Unassigned	AZOBR_p1170001	Horizontally Transferred (high confidence)
AZOLI_p40012	Ancestral (medium confidence)	AZOBR_p1170002	Horizontally Transferred (high confidence)
AZOLI_p40013	Ancestral (medium confidence)	AZOBR_p1170003	Unassigned
AZOLI_p40014	Ancestral (medium confidence)	AZOBR_p1170004	Ancestral (high confidence)
AZOLI_p40015	Ancestral (low confidence)	AZOBR_p1170005	Ancestral (medium confidence)
AZOLI_p40016	Ancestral (low confidence)	AZOBR_p1170006	Ancestral (low confidence)
AZOLI_p40017	Ancestral (medium confidence)	AZOBR_p1170007	Unassigned
AZOLI_p40018	Ancestral (low confidence)	AZOBR_p1170008	Ancestral (high confidence)
AZOLI_p40019	Horizontally Transferred (high confidence)	AZOBR_p1170009	Unassigned
AZOLI_p40020	Horizontally Transferred (high confidence)	AZOBR_p1170010	Unassigned
AZOLI_p40021	Horizontally Transferred (high confidence)	AZOBR_p1170011	Ancestral (low confidence)
AZOLI_p40022	Horizontally Transferred (high confidence)	AZOBR_p1170012	Ancestral (low confidence)
AZOLI_p40023	Horizontally Transferred (high confidence)	AZOBR_p1170013	Ancestral (high confidence)
AZOLI_p40024	Horizontally Transferred (high confidence)	AZOBR_p1170014	Horizontally Transferred (high confidence)
AZOLI_p40025	Horizontally Transferred (high confidence)	AZOBR_p1170015	Unassigned
AZOLI_p40026	Horizontally Transferred (high confidence)	AZOBR_p1170016	Horizontally Transferred (high confidence)
AZOLI_p40027	Ancestral (low confidence)	AZOBR_p1170017	Ancestral (low confidence)
AZOLI_p40028	Unassigned	AZOBR_p1170018	Ancestral (low confidence)
AZOLI_p40029	Horizontally Transferred (high confidence)	AZOBR_p1170019	Unassigned
AZOLI_p40030	Ancestral (low confidence)	AZOBR_p1170020	Horizontally Transferred (high confidence)
AZOLI_p40031	Ancestral (low confidence)	AZOBR_p1170021	Horizontally Transferred (low confidence)
AZOLI_p40032	Ancestral (medium confidence)	AZOBR_p1170022	Horizontally Transferred (high confidence)
AZOLI_p40033	Unassigned	AZOBR_p1170023	Horizontally Transferred (medium confidence)
AZOLI_p40034	Unassigned	AZOBR_p1170024	Horizontally Transferred (medium confidence)
AZOLI_p40036	Horizontally Transferred (high confidence)	AZOBR_p1170025	Unassigned
AZOLI_p40037	Horizontally Transferred (high confidence)	AZOBR_p1170026	Ancestral (low confidence)
AZOLI_p40038	Horizontally Transferred (high confidence)	AZOBR_p1170027	Horizontally Transferred (high confidence)

AZOLI_p40039	Horizontally Transferred (high confidence)	AZOBR_p1170028	Ancestral (low confidence)
AZOLI_p40040	Horizontally Transferred (high confidence)	AZOBR_p1170029	Horizontally Transferred (high confidence)
AZOLI_p40043	Horizontally Transferred (high confidence)	AZOBR_p1170030	Unassigned
AZOLI_p40044	Ancestral (medium confidence)	AZOBR_p1170031	Unassigned
AZOLI_p40045	Horizontally Transferred (medium confidence)	AZOBR_p1170032	Horizontally Transferred (high confidence)
AZOLI_p40046	Horizontally Transferred (high confidence)	AZOBR_p1170033	Unassigned
AZOLI_p40047	Unassigned	AZOBR_p1170034	Ancestral (high confidence)
AZOLI_p40048	Unassigned	AZOBR_p1170035	Horizontally Transferred (high confidence)
AZOLI_p40050	Unassigned	AZOBR_p1170036	Ancestral (medium confidence)
AZOLI_p40051	Ancestral (medium confidence)	AZOBR_p1170037	Horizontally Transferred (high confidence)
AZOLI_p40052	Ancestral (high confidence)	AZOBR_p1170038	Ancestral (low confidence)
AZOLI_p40053	Ancestral (high confidence)	AZOBR_p1170039	Ancestral (medium confidence)
AZOLI_p40055	Ancestral (high confidence)	AZOBR_p1170040	Ancestral (medium confidence)
AZOLI_p40056	Horizontally Transferred (high confidence)	AZOBR_p1170041	Horizontally Transferred (high confidence)
AZOLI_p40057	Horizontally Transferred (low confidence)	AZOBR_p1170042	Horizontally Transferred (high confidence)
AZOLI_p40058	Horizontally Transferred (high confidence)	AZOBR_p1170043	Ancestral (low confidence)
AZOLI_p40059	Ancestral (medium confidence)	AZOBR_p1170044	Ancestral (low confidence)
AZOLI_p40060	Horizontally Transferred (high confidence)	AZOBR_p1170045	Horizontally Transferred (high confidence)
AZOLI_p40061	Ancestral (medium confidence)	AZOBR_p1170046	Ancestral (medium confidence)
AZOLI_p40062	Ancestral (medium confidence)	AZOBR_p1170047	Ancestral (medium confidence)
AZOLI_p40063	Unassigned	AZOBR_p1170048	Unassigned
AZOLI_p40064	Ancestral (low confidence)	AZOBR_p1170049	Ancestral (medium confidence)
AZOLI_p40065	Horizontally Transferred (high confidence)	AZOBR_p1170050	Horizontally Transferred (high confidence)
AZOLI_p40066	Ancestral (low confidence)	AZOBR_p1170051	Horizontally Transferred (high confidence)
AZOLI_p40067	Ancestral (medium confidence)	AZOBR_p1170052	Ancestral (medium confidence)
AZOLI_p40068	Ancestral (low confidence)	AZOBR_p1170053	Ancestral (low confidence)
AZOLI_p40069	Ancestral (high confidence)	AZOBR_p1170054	Unassigned
AZOLI_p40070	Ancestral (low confidence)	AZOBR_p1170055	Ancestral (high confidence)
AZOLI_p40071	Ancestral (medium confidence)	AZOBR_p1170056	Ancestral (high confidence)
AZOLI_p40072	Horizontally Transferred (high confidence)	AZOBR_p1170057	Unassigned
AZOLI_p40073	Horizontally Transferred (high confidence)	AZOBR_p1170058	Horizontally Transferred (high confidence)
AZOLI_p40074	Horizontally Transferred (high confidence)	AZOBR_p1170059	Ancestral (medium confidence)
AZOLI_p40075	Horizontally Transferred (high confidence)	AZOBR_p1170060	Ancestral (medium confidence)
AZOLI_p40076	Horizontally Transferred (high confidence)	AZOBR_p1170061	Unassigned
AZOLI_p40077	Horizontally Transferred (high confidence)	AZOBR_p1170062	Ancestral (low confidence)
AZOLI_p40078	Ancestral (high confidence)	AZOBR_p1170063	Unassigned
AZOLI_p40079	Ancestral (high confidence)	AZOBR_p1170064	Unassigned
AZOLI_p40080	Ancestral (medium confidence)	AZOBR_p1170065	Ancestral (low confidence)
AZOLI_p40081	Horizontally Transferred (high confidence)	AZOBR_p1170066	Unassigned
AZOLI_p40082	Ancestral (low confidence)	AZOBR_p1170067	Unassigned
AZOLI_p40083	Unassigned	AZOBR_p1170068	Horizontally Transferred (high confidence)
AZOLI_p40084	Unassigned	AZOBR_p1170069	Horizontally Transferred (high confidence)
AZOLI_p40085	Ancestral (low confidence)	AZOBR_p1170070	Ancestral (low confidence)
AZOLI_p40086	Unassigned	AZOBR_p1170071	Ancestral (low confidence)
AZOLI_p40087	Unassigned	AZOBR_p1170072	Ancestral (low confidence)
AZOLI_p40088	Unassigned	AZOBR_p1170073	Ancestral (high confidence)
AZOLI_p40090	Ancestral (low confidence)	AZOBR_p1170074	Unassigned
AZOLI_p40091	Unassigned	AZOBR_p1170075	Ancestral (low confidence)
AZOLI_p40092	Unassigned	AZOBR_p1170076	Horizontally Transferred (high confidence)
AZOLI_p40093	Ancestral (low confidence)	AZOBR_p1170077	Horizontally Transferred (high confidence)
AZOLI_p40094	Unassigned	AZOBR_p1170078	Horizontally Transferred (medium confidence)
AZOLI_p40095	Horizontally Transferred (high confidence)	AZOBR_p1170079	Ancestral (medium confidence)
AZOLI_p40096	Ancestral (low confidence)	AZOBR_p1170080	Ancestral (medium confidence)
AZOLI_p40098	Ancestral (low confidence)	AZOBR_p1170081	Ancestral (low confidence)
AZOLI_p40099	Horizontally Transferred (high confidence)	AZOBR_p1170082	Unassigned
AZOLI_p40100	Horizontally Transferred (high confidence)	AZOBR_p1170083	Ancestral (medium confidence)
AZOLI_p40101	Horizontally Transferred (high confidence)	AZOBR_p1170084	Ancestral (high confidence)
AZOLI_p40102	Horizontally Transferred (high confidence)	AZOBR_p1170085	Ancestral (medium confidence)
AZOLI_p40104	Horizontally Transferred (high confidence)	AZOBR_p1170086	Ancestral (high confidence)
AZOLI_p40105	Horizontally Transferred (high confidence)	AZOBR_p1170087	Ancestral (low confidence)
AZOLI_p40106	Horizontally Transferred (medium confidence)	AZOBR_p1170088	Ancestral (high confidence)
AZOLI_p40107	Horizontally Transferred (high confidence)	AZOBR_p1170089	Unassigned
AZOLI_p40108	Horizontally Transferred (high confidence)	AZOBR_p1170090	Unassigned
AZOLI_p40109	Horizontally Transferred (high confidence)	AZOBR_p1170091	Ancestral (medium confidence)
AZOLI_p40110	Unassigned	AZOBR_p1170092	Ancestral (high confidence)
AZOLI_p40111	Unassigned	AZOBR_p1170093	Ancestral (high confidence)
AZOLI_p40112	Horizontally Transferred (high confidence)	AZOBR_p1170094	Ancestral (low confidence)
AZOLI_p40113	Horizontally Transferred (high confidence)	AZOBR_p1170095	Unassigned
AZOLI_p40114	Unassigned	AZOBR_p1180001	Ancestral (low confidence)
AZOLI_p40115	Unassigned	AZOBR_p1180002	Horizontally Transferred (high confidence)
AZOLI_p40116	Horizontally Transferred (high confidence)	AZOBR_p1180003	Unassigned
AZOLI_p40117	Horizontally Transferred (high confidence)	AZOBR_p1180004	Unassigned
AZOLI_p40118	Ancestral (low confidence)	AZOBR_p1180005	Unassigned
AZOLI_p40119	Unassigned	AZOBR_p1180006	Ancestral (low confidence)
AZOLI_p40120	Horizontally Transferred (high confidence)	AZOBR_p1180007	Horizontally Transferred (high confidence)

AZOLI_p40121	Horizontally Transferred (high confidence)	AZOBR_p1180008	Ancestral (low confidence)
AZOLI_p40122	Unassigned	AZOBR_p1180009	Unassigned
AZOLI_p40123	Horizontally Transferred (high confidence)	AZOBR_p1180010	Ancestral (low confidence)
AZOLI_p40124	Ancestral (high confidence)	AZOBR_p1180011	Ancestral (medium confidence)
AZOLI_p40125	Ancestral (high confidence)	AZOBR_p1180012	Ancestral (high confidence)
AZOLI_p40126	Ancestral (high confidence)	AZOBR_p1180013	Ancestral (high confidence)
AZOLI_p40127	Ancestral (high confidence)	AZOBR_p1180014	Ancestral (medium confidence)
AZOLI_p40128	Horizontally Transferred (high confidence)	AZOBR_p1180015	Ancestral (medium confidence)
AZOLI_p40129	Unassigned	AZOBR_p1180016	Horizontally Transferred (high confidence)
AZOLI_p40130	Horizontally Transferred (low confidence)	AZOBR_p1180017	Horizontally Transferred (high confidence)
AZOLI_p40131	Horizontally Transferred (high confidence)	AZOBR_p1180018	Ancestral (medium confidence)
AZOLI_p40132	Ancestral (low confidence)	AZOBR_p1180019	Ancestral (medium confidence)
AZOLI_p40133	Ancestral (low confidence)	AZOBR_p1180020	Ancestral (medium confidence)
AZOLI_p40134	Ancestral (high confidence)	AZOBR_p1180021	Ancestral (medium confidence)
AZOLI_p40135	Ancestral (medium confidence)	AZOBR_p1180022	Ancestral (low confidence)
AZOLI_p40137	Ancestral (medium confidence)	AZOBR_p1180023	Horizontally Transferred (medium confidence)
AZOLI_p40138	Ancestral (low confidence)	AZOBR_p1180024	Ancestral (medium confidence)
AZOLI_p40139	Ancestral (low confidence)	AZOBR_p1180025	Ancestral (high confidence)
AZOLI_p40140	Horizontally Transferred (high confidence)	AZOBR_p1180026	Ancestral (high confidence)
AZOLI_p40142	Horizontally Transferred (high confidence)	AZOBR_p1180027	Ancestral (high confidence)
AZOLI_p40144	Ancestral (low confidence)	AZOBR_p1180028	Ancestral (high confidence)
AZOLI_p40145	Horizontally Transferred (low confidence)	AZOBR_p1180029	Ancestral (high confidence)
AZOLI_p40146	Horizontally Transferred (high confidence)	AZOBR_p1180030	Ancestral (medium confidence)
AZOLI_p40147	Horizontally Transferred (medium confidence)	AZOBR_p1180031	Ancestral (medium confidence)
AZOLI_p40148	Unassigned	AZOBR_p1180032	Ancestral (high confidence)
AZOLI_p40149	Unassigned	AZOBR_p1180033	Ancestral (medium confidence)
AZOLI_p40150	Ancestral (medium confidence)	AZOBR_p1180034	Unassigned
AZOLI_p40151	Ancestral (high confidence)	AZOBR_p1180035	Ancestral (high confidence)
AZOLI_p40152	Ancestral (high confidence)	AZOBR_p1180036	Ancestral (high confidence)
AZOLI_p40153	Unassigned	AZOBR_p1180037	Ancestral (high confidence)
AZOLI_p40154	Horizontally Transferred (high confidence)	AZOBR_p210001	Unassigned
AZOLI_p40155	Horizontally Transferred (medium confidence)	AZOBR_p210002	Ancestral (high confidence)
AZOLI_p40156	Unassigned	AZOBR_p210003	Ancestral (high confidence)
AZOLI_p40157	Ancestral (low confidence)	AZOBR_p210004	Ancestral (medium confidence)
AZOLI_p40158	Ancestral (low confidence)	AZOBR_p210005	Ancestral (medium confidence)
AZOLI_p40159	Horizontally Transferred (medium confidence)	AZOBR_p210006	Ancestral (medium confidence)
AZOLI_p40160	Horizontally Transferred (high confidence)	AZOBR_p210007	Ancestral (medium confidence)
AZOLI_p40161	Ancestral (medium confidence)	AZOBR_p210008	Ancestral (medium confidence)
AZOLI_p40163	Ancestral (medium confidence)	AZOBR_p210009	Ancestral (medium confidence)
AZOLI_p40164	Ancestral (high confidence)	AZOBR_p210010	Ancestral (medium confidence)
AZOLI_p40165	Ancestral (medium confidence)	AZOBR_p210011	Ancestral (medium confidence)
AZOLI_p40166	Horizontally Transferred (high confidence)	AZOBR_p210012	Ancestral (medium confidence)
AZOLI_p40167	Horizontally Transferred (high confidence)	AZOBR_p210013	Ancestral (medium confidence)
AZOLI_p40168	Unassigned	AZOBR_p210014	Horizontally Transferred (high confidence)
AZOLI_p40170	Horizontally Transferred (medium confidence)	AZOBR_p210015	Unassigned
AZOLI_p40171	Horizontally Transferred (high confidence)	AZOBR_p210016	Ancestral (high confidence)
AZOLI_p40172	Horizontally Transferred (high confidence)	AZOBR_p210017	Horizontally Transferred (medium confidence)
AZOLI_p40174	Horizontally Transferred (high confidence)	AZOBR_p210018	Horizontally Transferred (medium confidence)
AZOLI_p40175	Ancestral (medium confidence)	AZOBR_p210019	Horizontally Transferred (high confidence)
AZOLI_p40176	Horizontally Transferred (medium confidence)	AZOBR_p210020	Ancestral (medium confidence)
AZOLI_p40177	Ancestral (low confidence)	AZOBR_p210021	Horizontally Transferred (medium confidence)
AZOLI_p40178	Ancestral (low confidence)	AZOBR_p210022	Unassigned
AZOLI_p40179	Ancestral (low confidence)	AZOBR_p210023	Horizontally Transferred (high confidence)
AZOLI_p40180	Ancestral (low confidence)	AZOBR_p210024	Ancestral (medium confidence)
AZOLI_p40181	Horizontally Transferred (high confidence)	AZOBR_p210025	Ancestral (medium confidence)
AZOLI_p40182	Horizontally Transferred (high confidence)	AZOBR_p210026	Ancestral (medium confidence)
AZOLI_p40183	Horizontally Transferred (high confidence)	AZOBR_p210027	Ancestral (medium confidence)
AZOLI_p40184	Horizontally Transferred (high confidence)	AZOBR_p210028	Unassigned
AZOLI_p40185	Horizontally Transferred (low confidence)	AZOBR_p210029	Ancestral (high confidence)
AZOLI_p40186	Unassigned	AZOBR_p210030	Ancestral (medium confidence)
AZOLI_p40187	Ancestral (medium confidence)	AZOBR_p210031	Ancestral (medium confidence)
AZOLI_p40188	Ancestral (medium confidence)	AZOBR_p210032	Horizontally Transferred (low confidence)
AZOLI_p40189	Ancestral (high confidence)	AZOBR_p210033	Unassigned
AZOLI_p40190	Ancestral (medium confidence)	AZOBR_p210034	Unassigned
AZOLI_p40191	Ancestral (medium confidence)	AZOBR_p210035	Unassigned
AZOLI_p40192	Ancestral (high confidence)	AZOBR_p210036	Unassigned
AZOLI_p40193	Ancestral (high confidence)	AZOBR_p210037	Unassigned
AZOLI_p40194	Unassigned	AZOBR_p210038	Horizontally Transferred (high confidence)
AZOLI_p40195	Unassigned	AZOBR_p210039	Unassigned
AZOLI_p40196	Horizontally Transferred (medium confidence)	AZOBR_p210040	Horizontally Transferred (high confidence)
AZOLI_p40197	Unassigned	AZOBR_p210041	Horizontally Transferred (high confidence)
AZOLI_p40198	Horizontally Transferred (low confidence)	AZOBR_p210042	Horizontally Transferred (high confidence)
AZOLI_p40199	Horizontally Transferred (high confidence)	AZOBR_p210043	Horizontally Transferred (high confidence)
AZOLI_p40200	Ancestral (low confidence)	AZOBR_p210044	Horizontally Transferred (high confidence)
AZOLI_p40201	Ancestral (low confidence)	AZOBR_p210045	Horizontally Transferred (high confidence)

AZOLI_p40202	Unassigned	AZOBR_p210046	Horizontally Transferred (high confidence)
AZOLI_p40204	Horizontally Transferred (high confidence)	AZOBR_p210047	Unassigned
AZOLI_p40205	Horizontally Transferred (high confidence)	AZOBR_p210048	Horizontally Transferred (high confidence)
AZOLI_p40206	Horizontally Transferred (high confidence)	AZOBR_p210049	Horizontally Transferred (high confidence)
AZOLI_p40207	Horizontally Transferred (high confidence)	AZOBR_p210050	Horizontally Transferred (high confidence)
AZOLI_p40208	Unassigned	AZOBR_p210051	Horizontally Transferred (high confidence)
AZOLI_p40209	Horizontally Transferred (high confidence)	AZOBR_p210052	Unassigned
AZOLI_p40210	Ancestral (medium confidence)	AZOBR_p210053	Unassigned
AZOLI_p40211	Horizontally Transferred (high confidence)	AZOBR_p210054	Horizontally Transferred (high confidence)
AZOLI_p40212	Ancestral (medium confidence)	AZOBR_p210055	Horizontally Transferred (high confidence)
AZOLI_p40213	Ancestral (medium confidence)	AZOBR_p210056	Horizontally Transferred (high confidence)
AZOLI_p40215	Horizontally Transferred (high confidence)	AZOBR_p210057	Unassigned
AZOLI_p40216	Ancestral (high confidence)	AZOBR_p210058	Horizontally Transferred (high confidence)
AZOLI_p40217	Horizontally Transferred (high confidence)	AZOBR_p210059	Horizontally Transferred (high confidence)
AZOLI_p40218	Ancestral (high confidence)	AZOBR_p210060	Unassigned
AZOLI_p40219	Ancestral (high confidence)	AZOBR_p210061	Horizontally Transferred (high confidence)
AZOLI_p40220	Ancestral (medium confidence)	AZOBR_p210062	Horizontally Transferred (high confidence)
AZOLI_p40221	Horizontally Transferred (high confidence)	AZOBR_p210063	Ancestral (low confidence)
AZOLI_p40222	Horizontally Transferred (high confidence)	AZOBR_p210064	Ancestral (low confidence)
AZOLI_p40223	Ancestral (low confidence)	AZOBR_p210065	Ancestral (low confidence)
AZOLI_p40224	Unassigned	AZOBR_p210066	Horizontally Transferred (high confidence)
AZOLI_p40225	Horizontally Transferred (high confidence)	AZOBR_p210067	Horizontally Transferred (low confidence)
AZOLI_p40227	Horizontally Transferred (high confidence)	AZOBR_p210068	Horizontally Transferred (high confidence)
AZOLI_p40229	Horizontally Transferred (high confidence)	AZOBR_p210069	Horizontally Transferred (high confidence)
AZOLI_p40230	Horizontally Transferred (high confidence)	AZOBR_p210070	Ancestral (low confidence)
AZOLI_p40231	Horizontally Transferred (high confidence)	AZOBR_p210071	Ancestral (medium confidence)
AZOLI_p40232	Horizontally Transferred (high confidence)	AZOBR_p210072	Ancestral (low confidence)
AZOLI_p40233	Horizontally Transferred (high confidence)	AZOBR_p210073	Ancestral (low confidence)
AZOLI_p40234	Horizontally Transferred (high confidence)	AZOBR_p210074	Ancestral (low confidence)
AZOLI_p40235	Horizontally Transferred (high confidence)	AZOBR_p210075	Ancestral (low confidence)
AZOLI_p40236	Ancestral (medium confidence)	AZOBR_p210076	Ancestral (low confidence)
AZOLI_p40237	Ancestral (medium confidence)	AZOBR_p210077	Ancestral (low confidence)
AZOLI_p40238	Horizontally Transferred (high confidence)	AZOBR_p210078	Unassigned
AZOLI_p40239	Ancestral (medium confidence)	AZOBR_p210079	Ancestral (low confidence)
AZOLI_p40240	Horizontally Transferred (medium confidence)	AZOBR_p210080	Unassigned
AZOLI_p40241	Horizontally Transferred (high confidence)	AZOBR_p210081	Horizontally Transferred (high confidence)
AZOLI_p40242	Ancestral (low confidence)	AZOBR_p210082	Horizontally Transferred (high confidence)
AZOLI_p40243	Horizontally Transferred (medium confidence)	AZOBR_p210083	Horizontally Transferred (high confidence)
AZOLI_p40244	Ancestral (low confidence)	AZOBR_p210084	Unassigned
AZOLI_p40245	Horizontally Transferred (high confidence)	AZOBR_p210085	Horizontally Transferred (high confidence)
AZOLI_p40246	Horizontally Transferred (medium confidence)	AZOBR_p210086	Horizontally Transferred (high confidence)
AZOLI_p40247	Horizontally Transferred (high confidence)	AZOBR_p210087	Unassigned
AZOLI_p40248	Horizontally Transferred (low confidence)	AZOBR_p210088	Ancestral (low confidence)
AZOLI_p40249	Horizontally Transferred (medium confidence)	AZOBR_p210089	Horizontally Transferred (high confidence)
AZOLI_p40250	Ancestral (low confidence)	AZOBR_p210090	Horizontally Transferred (high confidence)
AZOLI_p40251	Ancestral (low confidence)	AZOBR_p210091	Horizontally Transferred (high confidence)
AZOLI_p40252	Horizontally Transferred (medium confidence)	AZOBR_p210092	Unassigned
AZOLI_p40253	Ancestral (medium confidence)	AZOBR_p210093	Horizontally Transferred (high confidence)
AZOLI_p40254	Horizontally Transferred (high confidence)	AZOBR_p210094	Ancestral (low confidence)
AZOLI_p40255	Ancestral (medium confidence)	AZOBR_p210095	Ancestral (medium confidence)
AZOLI_p40256	Horizontally Transferred (low confidence)	AZOBR_p210096	Unassigned
AZOLI_p40257	Ancestral (medium confidence)	AZOBR_p210097	Unassigned
AZOLI_p40258	Horizontally Transferred (medium confidence)	AZOBR_p210098	Ancestral (low confidence)
AZOLI_p40259	Ancestral (medium confidence)	AZOBR_p210099	Ancestral (low confidence)
AZOLI_p40260	Ancestral (low confidence)	AZOBR_p210100	Unassigned
AZOLI_p40261	Ancestral (low confidence)	AZOBR_p210101	Unassigned
AZOLI_p40262	Horizontally Transferred (high confidence)	AZOBR_p210102	Unassigned
AZOLI_p40263	Horizontally Transferred (high confidence)	AZOBR_p210103	Ancestral (low confidence)
AZOLI_p40264	Horizontally Transferred (high confidence)	AZOBR_p210104	Ancestral (low confidence)
AZOLI_p40265	Horizontally Transferred (high confidence)	AZOBR_p210105	Ancestral (low confidence)
AZOLI_p40266	Horizontally Transferred (high confidence)	AZOBR_p210106	Ancestral (medium confidence)
AZOLI_p40267	Horizontally Transferred (low confidence)	AZOBR_p210107	Ancestral (medium confidence)
AZOLI_p40268	Ancestral (medium confidence)	AZOBR_p210108	Unassigned
AZOLI_p40269	Ancestral (medium confidence)	AZOBR_p210109	Horizontally Transferred (high confidence)
AZOLI_p40270	Ancestral (low confidence)	AZOBR_p210110	Unassigned
AZOLI_p40271	Horizontally Transferred (high confidence)	AZOBR_p210111	Unassigned
AZOLI_p40272	Horizontally Transferred (high confidence)	AZOBR_p210112	Horizontally Transferred (high confidence)
AZOLI_p40274	Horizontally Transferred (high confidence)	AZOBR_p210113	Horizontally Transferred (high confidence)
AZOLI_p40275	Horizontally Transferred (high confidence)	AZOBR_p210114	Horizontally Transferred (high confidence)
AZOLI_p40276	Ancestral (medium confidence)	AZOBR_p210115	Horizontally Transferred (high confidence)
AZOLI_p40278	Unassigned	AZOBR_p210116	Horizontally Transferred (high confidence)
AZOLI_p40279	Ancestral (medium confidence)	AZOBR_p210117	Horizontally Transferred (medium confidence)
AZOLI_p40280	Horizontally Transferred (high confidence)	AZOBR_p210118	Horizontally Transferred (low confidence)
AZOLI_p40281	Ancestral (medium confidence)	AZOBR_p210119	Unassigned
AZOLI_p40282	Unassigned	AZOBR_p210120	Horizontally Transferred (medium confidence)

AZOLI_p40283	Unassigned	AZOBR_p210121	Unassigned
AZOLI_p40284	Unassigned	AZOBR_p210122	Ancestral (low confidence)
AZOLI_p40285	Unassigned	AZOBR_p210123	Unassigned
AZOLI_p40286	Horizontally Transferred (high confidence)	AZOBR_p210124	Horizontally Transferred (high confidence)
AZOLI_p40287	Horizontally Transferred (high confidence)	AZOBR_p210125	Horizontally Transferred (high confidence)
AZOLI_p40288	Horizontally Transferred (high confidence)	AZOBR_p210126	Horizontally Transferred (high confidence)
AZOLI_p40289	Ancestral (medium confidence)	AZOBR_p210127	Horizontally Transferred (high confidence)
AZOLI_p40290	Ancestral (low confidence)	AZOBR_p210128	Horizontally Transferred (high confidence)
AZOLI_p40291	Ancestral (low confidence)	AZOBR_p210129	Horizontally Transferred (high confidence)
AZOLI_p40293	Ancestral (low confidence)	AZOBR_p210130	Horizontally Transferred (high confidence)
AZOLI_p40294	Horizontally Transferred (high confidence)	AZOBR_p210131	Unassigned
AZOLI_p40295	Ancestral (low confidence)	AZOBR_p210132	Unassigned
AZOLI_p40296	Horizontally Transferred (high confidence)	AZOBR_p210133	Unassigned
AZOLI_p40297	Unassigned	AZOBR_p210134	Horizontally Transferred (high confidence)
AZOLI_p40298	Horizontally Transferred (high confidence)	AZOBR_p210135	Horizontally Transferred (high confidence)
AZOLI_p40299	Horizontally Transferred (medium confidence)	AZOBR_p210136	Horizontally Transferred (high confidence)
AZOLI_p40300	Horizontally Transferred (low confidence)	AZOBR_p210137	Unassigned
AZOLI_p40301	Horizontally Transferred (high confidence)	AZOBR_p210138	Horizontally Transferred (high confidence)
AZOLI_p40302	Ancestral (low confidence)	AZOBR_p210139	Horizontally Transferred (high confidence)
AZOLI_p40303	Horizontally Transferred (medium confidence)	AZOBR_p210140	Horizontally Transferred (high confidence)
AZOLI_p40304	Horizontally Transferred (high confidence)	AZOBR_p210141	Ancestral (low confidence)
AZOLI_p40305	Horizontally Transferred (low confidence)	AZOBR_p210142	Unassigned
AZOLI_p40307	Unassigned	AZOBR_p210143	Horizontally Transferred (high confidence)
AZOLI_p40308	Horizontally Transferred (high confidence)	AZOBR_p210144	Horizontally Transferred (high confidence)
AZOLI_p40309	Ancestral (high confidence)	AZOBR_p210145	Horizontally Transferred (high confidence)
AZOLI_p40310	Horizontally Transferred (high confidence)	AZOBR_p210146	Ancestral (medium confidence)
AZOLI_p40311	Unassigned	AZOBR_p210147	Horizontally Transferred (high confidence)
AZOLI_p40312	Horizontally Transferred (high confidence)	AZOBR_p210148	Ancestral (high confidence)
AZOLI_p40313	Unassigned	AZOBR_p210149	Ancestral (low confidence)
AZOLI_p40314	Ancestral (medium confidence)	AZOBR_p210150	Horizontally Transferred (medium confidence)
AZOLI_p40315	Horizontally Transferred (high confidence)	AZOBR_p210151	Unassigned
AZOLI_p40316	Ancestral (low confidence)	AZOBR_p210152	Horizontally Transferred (high confidence)
AZOLI_p40317	Ancestral (low confidence)	AZOBR_p210153	Horizontally Transferred (high confidence)
AZOLI_p40318	Ancestral (low confidence)	AZOBR_p210154	Horizontally Transferred (medium confidence)
AZOLI_p40319	Ancestral (high confidence)	AZOBR_p210155	Unassigned
AZOLI_p40320	Unassigned	AZOBR_p210156	Horizontally Transferred (low confidence)
AZOLI_p40321	Ancestral (medium confidence)	AZOBR_p210157	Ancestral (high confidence)
AZOLI_p40322	Horizontally Transferred (medium confidence)	AZOBR_p210158	Unassigned
AZOLI_p40323	Horizontally Transferred (medium confidence)	AZOBR_p210159	Ancestral (high confidence)
AZOLI_p40324	Ancestral (medium confidence)	AZOBR_p210160	Unassigned
AZOLI_p40325	Ancestral (low confidence)	AZOBR_p210161	Unassigned
AZOLI_p40326	Ancestral (medium confidence)	AZOBR_p210162	Ancestral (low confidence)
AZOLI_p40327	Ancestral (high confidence)	AZOBR_p210163	Horizontally Transferred (high confidence)
AZOLI_p40328	Ancestral (medium confidence)	AZOBR_p210164	Ancestral (low confidence)
AZOLI_p40329	Ancestral (high confidence)	AZOBR_p210165	Horizontally Transferred (high confidence)
AZOLI_p40330	Ancestral (high confidence)	AZOBR_p210166	Unassigned
AZOLI_p40331	Horizontally Transferred (low confidence)	AZOBR_p210167	Ancestral (low confidence)
AZOLI_p40332	Ancestral (medium confidence)	AZOBR_p210168	Ancestral (medium confidence)
AZOLI_p40333	Ancestral (medium confidence)	AZOBR_p210169	Ancestral (medium confidence)
AZOLI_p40334	Horizontally Transferred (high confidence)	AZOBR_p210170	Horizontally Transferred (high confidence)
AZOLI_p40335	Ancestral (low confidence)	AZOBR_p210171	Unassigned
AZOLI_p40336	Horizontally Transferred (high confidence)	AZOBR_p210172	Horizontally Transferred (high confidence)
AZOLI_p40337	Ancestral (medium confidence)	AZOBR_p210173	Unassigned
AZOLI_p40338	Ancestral (low confidence)	AZOBR_p210174	Horizontally Transferred (high confidence)
AZOLI_p40339	Ancestral (medium confidence)	AZOBR_p210175	Horizontally Transferred (high confidence)
AZOLI_p40340	Ancestral (medium confidence)	AZOBR_p210176	Horizontally Transferred (high confidence)
AZOLI_p40341	Unassigned	AZOBR_p210177	Horizontally Transferred (high confidence)
AZOLI_p40342	Ancestral (medium confidence)	AZOBR_p210178	Horizontally Transferred (high confidence)
AZOLI_p40344	Unassigned	AZOBR_p210179	Unassigned
AZOLI_p40345	Unassigned	AZOBR_p210180	Horizontally Transferred (high confidence)
AZOLI_p40346	Unassigned	AZOBR_p210181	Unassigned
AZOLI_p40349	Horizontally Transferred (high confidence)	AZOBR_p210182	Horizontally Transferred (high confidence)
AZOLI_p40350	Unassigned	AZOBR_p210183	Horizontally Transferred (high confidence)
AZOLI_p40351	Ancestral (medium confidence)	AZOBR_p210184	Horizontally Transferred (high confidence)
AZOLI_p40352	Ancestral (high confidence)	AZOBR_p210185	Horizontally Transferred (high confidence)
AZOLI_p40353	Horizontally Transferred (high confidence)	AZOBR_p210186	Horizontally Transferred (high confidence)
AZOLI_p40354	Horizontally Transferred (low confidence)	AZOBR_p210187	Horizontally Transferred (high confidence)
AZOLI_p40355	Ancestral (low confidence)	AZOBR_p210188	Unassigned
AZOLI_p40356	Ancestral (medium confidence)	AZOBR_p210189	Ancestral (low confidence)
AZOLI_p40357	Horizontally Transferred (high confidence)	AZOBR_p210190	Horizontally Transferred (low confidence)
AZOLI_p40358	Horizontally Transferred (high confidence)	AZOBR_p210191	Ancestral (low confidence)
AZOLI_p40359	Ancestral (low confidence)	AZOBR_p210192	Horizontally Transferred (high confidence)
AZOLI_p40360	Horizontally Transferred (high confidence)	AZOBR_p210193	Unassigned
AZOLI_p40361	Horizontally Transferred (high confidence)	AZOBR_p210194	Horizontally Transferred (high confidence)
AZOLI_p40362	Ancestral (high confidence)	AZOBR_p210195	Horizontally Transferred (high confidence)

AZOLI_p40363	Horizontally Transferred (high confidence)	AZOBR_p210196	Horizontally Transferred (high confidence)
AZOLI_p40364	Horizontally Transferred (high confidence)	AZOBR_p210197	Horizontally Transferred (medium confidence)
AZOLI_p40365	Unassigned	AZOBR_p210198	Horizontally Transferred (high confidence)
AZOLI_p40366	Ancestral (medium confidence)	AZOBR_p210199	Unassigned
AZOLI_p40367	Unassigned	AZOBR_p210200	Ancestral (high confidence)
AZOLI_p40368	Ancestral (low confidence)	AZOBR_p210201	Ancestral (medium confidence)
AZOLI_p40369	Horizontally Transferred (high confidence)	AZOBR_p220001	Unassigned
AZOLI_p40370	Unassigned	AZOBR_p220002	Horizontally Transferred (high confidence)
AZOLI_p40371	Ancestral (medium confidence)	AZOBR_p220003	Unassigned
AZOLI_p40372	Unassigned	AZOBR_p220004	Ancestral (low confidence)
AZOLI_p40373	Ancestral (high confidence)	AZOBR_p220005	Ancestral (low confidence)
AZOLI_p40374	Ancestral (medium confidence)	AZOBR_p220006	Ancestral (low confidence)
AZOLI_p40375	Ancestral (medium confidence)	AZOBR_p220007	Ancestral (low confidence)
AZOLI_p40376	Ancestral (medium confidence)	AZOBR_p220008	Ancestral (low confidence)
AZOLI_p40377	Horizontally Transferred (medium confidence)	AZOBR_p220009	Ancestral (low confidence)
AZOLI_p40378	Horizontally Transferred (high confidence)	AZOBR_p220010	Horizontally Transferred (medium confidence)
AZOLI_p40379	Horizontally Transferred (high confidence)	AZOBR_p220011	Unassigned
AZOLI_p40380	Ancestral (medium confidence)	AZOBR_p220012	Horizontally Transferred (low confidence)
AZOLI_p40381	Unassigned	AZOBR_p220013	Horizontally Transferred (low confidence)
AZOLI_p40382	Ancestral (medium confidence)	AZOBR_p220014	Horizontally Transferred (high confidence)
AZOLI_p40383	Horizontally Transferred (high confidence)	AZOBR_p220015	Horizontally Transferred (high confidence)
AZOLI_p40385	Unassigned	AZOBR_p220016	Horizontally Transferred (high confidence)
AZOLI_p40386	Horizontally Transferred (high confidence)	AZOBR_p220017	Horizontally Transferred (high confidence)
AZOLI_p40387	Horizontally Transferred (high confidence)	AZOBR_p220018	Horizontally Transferred (high confidence)
AZOLI_p40388	Unassigned	AZOBR_p220019	Horizontally Transferred (high confidence)
AZOLI_p40389	Horizontally Transferred (high confidence)	AZOBR_p220020	Ancestral (high confidence)
AZOLI_p40390	Horizontally Transferred (high confidence)	AZOBR_p220021	Ancestral (medium confidence)
AZOLI_p40391	Unassigned	AZOBR_p220022	Unassigned
AZOLI_p40392	Horizontally Transferred (high confidence)	AZOBR_p220023	Horizontally Transferred (high confidence)
AZOLI_p40393	Unassigned	AZOBR_p220024	Horizontally Transferred (high confidence)
AZOLI_p40395	Horizontally Transferred (high confidence)	AZOBR_p220025	Unassigned
AZOLI_p40396	Horizontally Transferred (high confidence)	AZOBR_p220026	Unassigned
AZOLI_p40397	Horizontally Transferred (high confidence)	AZOBR_p220027	Unassigned
AZOLI_p40398	Horizontally Transferred (high confidence)	AZOBR_p220028	Horizontally Transferred (high confidence)
AZOLI_p40399	Unassigned	AZOBR_p220029	Unassigned
AZOLI_p40400	Horizontally Transferred (high confidence)	AZOBR_p220030	Unassigned
AZOLI_p40401	Horizontally Transferred (high confidence)	AZOBR_p220031	Ancestral (low confidence)
AZOLI_p40403	Horizontally Transferred (high confidence)	AZOBR_p220032	Horizontally Transferred (high confidence)
AZOLI_p40404	Horizontally Transferred (medium confidence)	AZOBR_p220033	Horizontally Transferred (high confidence)
AZOLI_p40405	Ancestral (low confidence)	AZOBR_p220034	Ancestral (low confidence)
AZOLI_p40406	Horizontally Transferred (medium confidence)	AZOBR_p220035	Unassigned
AZOLI_p40407	Horizontally Transferred (high confidence)	AZOBR_p220036	Unassigned
AZOLI_p40408	Unassigned	AZOBR_p220037	Horizontally Transferred (high confidence)
AZOLI_p40409	Horizontally Transferred (high confidence)	AZOBR_p220038	Ancestral (medium confidence)
AZOLI_p40410	Ancestral (high confidence)	AZOBR_p220039	Unassigned
AZOLI_p40411	Ancestral (high confidence)	AZOBR_p220040	Ancestral (medium confidence)
AZOLI_p40412	Ancestral (medium confidence)	AZOBR_p220041	Ancestral (medium confidence)
AZOLI_p40413	Ancestral (medium confidence)	AZOBR_p220042	Unassigned
AZOLI_p40414	Ancestral (low confidence)	AZOBR_p220043	Ancestral (low confidence)
AZOLI_p40415	Unassigned	AZOBR_p220044	Horizontally Transferred (high confidence)
AZOLI_p40416	Ancestral (medium confidence)	AZOBR_p220045	Horizontally Transferred (high confidence)
AZOLI_p40417	Ancestral (medium confidence)	AZOBR_p220046	Ancestral (low confidence)
AZOLI_p40418	Ancestral (high confidence)	AZOBR_p220047	Ancestral (low confidence)
AZOLI_p40419	Unassigned	AZOBR_p220048	Unassigned
AZOLI_p40420	Ancestral (medium confidence)	AZOBR_p220049	Ancestral (low confidence)
AZOLI_p40421	Ancestral (low confidence)	AZOBR_p220050	Ancestral (low confidence)
AZOLI_p40422	Ancestral (medium confidence)	AZOBR_p220051	Ancestral (low confidence)
AZOLI_p40423	Ancestral (low confidence)	AZOBR_p220052	Unassigned
AZOLI_p40424	Horizontally Transferred (low confidence)	AZOBR_p220053	Horizontally Transferred (medium confidence)
AZOLI_p40425	Ancestral (low confidence)	AZOBR_p220054	Horizontally Transferred (high confidence)
AZOLI_p40426	Ancestral (medium confidence)	AZOBR_p220055	Unassigned
AZOLI_p40427	Ancestral (low confidence)	AZOBR_p220056	Horizontally Transferred (high confidence)
AZOLI_p40428	Horizontally Transferred (high confidence)	AZOBR_p220057	Horizontally Transferred (high confidence)
AZOLI_p40429	Horizontally Transferred (high confidence)	AZOBR_p220058	Horizontally Transferred (high confidence)
AZOLI_p40430	Horizontally Transferred (high confidence)	AZOBR_p220059	Horizontally Transferred (high confidence)
AZOLI_p40431	Ancestral (low confidence)	AZOBR_p220060	Horizontally Transferred (high confidence)
AZOLI_p40432	Horizontally Transferred (high confidence)	AZOBR_p220061	Horizontally Transferred (high confidence)
AZOLI_p40433	Ancestral (high confidence)	AZOBR_p220062	Unassigned
AZOLI_p40434	Ancestral (high confidence)	AZOBR_p220063	Unassigned
AZOLI_p40435	Ancestral (high confidence)	AZOBR_p220064	Horizontally Transferred (high confidence)
AZOLI_p40436	Ancestral (medium confidence)	AZOBR_p220065	Ancestral (low confidence)
AZOLI_p40437	Ancestral (medium confidence)	AZOBR_p220066	Horizontally Transferred (high confidence)
AZOLI_p40438	Unassigned	AZOBR_p220067	Horizontally Transferred (high confidence)
AZOLI_p40439	Ancestral (low confidence)	AZOBR_p220068	Unassigned
AZOLI_p40440	Ancestral (high confidence)	AZOBR_p220069	Horizontally Transferred (high confidence)

AZOLI_p40441	Ancestral (high confidence)	AZOBR_p220070	Ancestral (low confidence)
AZOLI_p40442	Ancestral (high confidence)	AZOBR_p220071	Ancestral (low confidence)
AZOLI_p40443	Ancestral (high confidence)	AZOBR_p220072	Horizontally Transferred (high confidence)
AZOLI_p40444	Ancestral (medium confidence)	AZOBR_p220073	Horizontally Transferred (high confidence)
AZOLI_p40445	Unassigned	AZOBR_p220074	Horizontally Transferred (high confidence)
AZOLI_p40446	Ancestral (low confidence)	AZOBR_p220075	Horizontally Transferred (high confidence)
AZOLI_p40447	Ancestral (medium confidence)	AZOBR_p220076	Horizontally Transferred (low confidence)
AZOLI_p40448	Unassigned	AZOBR_p220077	Ancestral (low confidence)
AZOLI_p40449	Ancestral (medium confidence)	AZOBR_p220078	Unassigned
AZOLI_p40450	Ancestral (low confidence)	AZOBR_p220079	Unassigned
AZOLI_p40451	Ancestral (medium confidence)	AZOBR_p220080	Ancestral (medium confidence)
AZOLI_p40452	Ancestral (low confidence)	AZOBR_p220081	Ancestral (medium confidence)
AZOLI_p40453	Ancestral (medium confidence)	AZOBR_p220082	Horizontally Transferred (high confidence)
AZOLI_p40454	Ancestral (low confidence)	AZOBR_p220083	Horizontally Transferred (high confidence)
AZOLI_p40455	Unassigned	AZOBR_p220084	Horizontally Transferred (high confidence)
AZOLI_p40456	Ancestral (medium confidence)	AZOBR_p220085	Unassigned
AZOLI_p40457	Horizontally Transferred (high confidence)	AZOBR_p220086	Unassigned
AZOLI_p40458	Horizontally Transferred (high confidence)	AZOBR_p220087	Ancestral (low confidence)
AZOLI_p40459	Ancestral (low confidence)	AZOBR_p220088	Horizontally Transferred (high confidence)
AZOLI_p40460	Horizontally Transferred (high confidence)	AZOBR_p220089	Horizontally Transferred (high confidence)
AZOLI_p40461	Ancestral (low confidence)	AZOBR_p220090	Horizontally Transferred (high confidence)
AZOLI_p40462	Horizontally Transferred (high confidence)	AZOBR_p220091	Horizontally Transferred (high confidence)
AZOLI_p40463	Ancestral (high confidence)	AZOBR_p220092	Unassigned
AZOLI_p40464	Ancestral (high confidence)	AZOBR_p220093	Unassigned
AZOLI_p40465	Ancestral (high confidence)	AZOBR_p220094	Horizontally Transferred (high confidence)
AZOLI_p40466	Ancestral (high confidence)	AZOBR_p220095	Horizontally Transferred (high confidence)
AZOLI_p40467	Unassigned	AZOBR_p220096	Horizontally Transferred (high confidence)
AZOLI_p40468	Ancestral (low confidence)	AZOBR_p220097	Unassigned
AZOLI_p40469	Ancestral (medium confidence)	AZOBR_p220098	Horizontally Transferred (high confidence)
AZOLI_p40470	Ancestral (medium confidence)	AZOBR_p220099	Ancestral (low confidence)
AZOLI_p40471	Ancestral (low confidence)	AZOBR_p220100	Ancestral (low confidence)
AZOLI_p40472	Unassigned	AZOBR_p220101	Ancestral (low confidence)
AZOLI_p40473	Ancestral (medium confidence)	AZOBR_p220102	Horizontally Transferred (high confidence)
AZOLI_p40474	Unassigned	AZOBR_p220103	Unassigned
AZOLI_p40475	Ancestral (medium confidence)	AZOBR_p220104	Horizontally Transferred (high confidence)
AZOLI_p40476	Ancestral (low confidence)	AZOBR_p220105	Horizontally Transferred (high confidence)
AZOLI_p40477	Ancestral (medium confidence)	AZOBR_p220106	Ancestral (medium confidence)
AZOLI_p40478	Ancestral (low confidence)	AZOBR_p220107	Ancestral (medium confidence)
AZOLI_p40479	Unassigned	AZOBR_p220108	Unassigned
AZOLI_p40480	Horizontally Transferred (high confidence)	AZOBR_p220109	Horizontally Transferred (high confidence)
AZOLI_p40481	Unassigned	AZOBR_p220110	Horizontally Transferred (high confidence)
AZOLI_p40482	Ancestral (high confidence)	AZOBR_p220111	Horizontally Transferred (low confidence)
AZOLI_p40483	Ancestral (medium confidence)	AZOBR_p220112	Horizontally Transferred (medium confidence)
AZOLI_p40485	Unassigned	AZOBR_p220113	Horizontally Transferred (high confidence)
AZOLI_p40486	Ancestral (low confidence)	AZOBR_p220114	Unassigned
AZOLI_p40487	Unassigned	AZOBR_p220115	Unassigned
AZOLI_p40488	Ancestral (low confidence)	AZOBR_p220116	Unassigned
AZOLI_p40490	Horizontally Transferred (high confidence)	AZOBR_p220117	Horizontally Transferred (high confidence)
AZOLI_p40491	Ancestral (medium confidence)	AZOBR_p220118	Ancestral (medium confidence)
AZOLI_p40492	Horizontally Transferred (high confidence)	AZOBR_p220119	Horizontally Transferred (high confidence)
AZOLI_p40493	Horizontally Transferred (high confidence)	AZOBR_p220120	Horizontally Transferred (high confidence)
AZOLI_p40494	Horizontally Transferred (high confidence)	AZOBR_p220121	Horizontally Transferred (high confidence)
AZOLI_p40495	Ancestral (low confidence)	AZOBR_p220122	Horizontally Transferred (high confidence)
AZOLI_p40496	Unassigned	AZOBR_p230001	Unassigned
AZOLI_p40497	Ancestral (low confidence)	AZOBR_p230002	Ancestral (medium confidence)
AZOLI_p40498	Horizontally Transferred (high confidence)	AZOBR_p230003	Horizontally Transferred (high confidence)
AZOLI_p40499	Horizontally Transferred (high confidence)	AZOBR_p230004	Unassigned
AZOLI_p40500	Horizontally Transferred (high confidence)	AZOBR_p230005	Horizontally Transferred (high confidence)
AZOLI_p40501	Ancestral (low confidence)	AZOBR_p230006	Horizontally Transferred (high confidence)
AZOLI_p40502	Unassigned	AZOBR_p230007	Unassigned
AZOLI_p40503	Horizontally Transferred (high confidence)	AZOBR_p230008	Horizontally Transferred (high confidence)
AZOLI_p40504	Unassigned	AZOBR_p230009	Horizontally Transferred (high confidence)
AZOLI_p40505	Ancestral (low confidence)	AZOBR_p230010	Horizontally Transferred (high confidence)
AZOLI_p40506	Unassigned	AZOBR_p230011	Unassigned
AZOLI_p40507	Ancestral (low confidence)	AZOBR_p230012	Horizontally Transferred (high confidence)
AZOLI_p40508	Horizontally Transferred (high confidence)	AZOBR_p230013	Unassigned
AZOLI_p40509	Horizontally Transferred (high confidence)	AZOBR_p230014	Unassigned
AZOLI_p40510	Horizontally Transferred (medium confidence)	AZOBR_p230015	Ancestral (low confidence)
AZOLI_p40511	Ancestral (medium confidence)	AZOBR_p230016	Horizontally Transferred (high confidence)
AZOLI_p40512	Ancestral (low confidence)	AZOBR_p230017	Horizontally Transferred (high confidence)
AZOLI_p40513	Ancestral (low confidence)	AZOBR_p230018	Ancestral (medium confidence)
AZOLI_p40514	Ancestral (medium confidence)	AZOBR_p230019	Unassigned
AZOLI_p40515	Ancestral (medium confidence)	AZOBR_p230020	Unassigned
AZOLI_p40516	Ancestral (medium confidence)	AZOBR_p230021	Ancestral (low confidence)
AZOLI_p40517	Ancestral (medium confidence)	AZOBR_p230022	Horizontally Transferred (high confidence)

AZOLI_p40518	Ancestral (high confidence)	AZOBR_p230023	Horizontally Transferred (high confidence)
AZOLI_p40519	Ancestral (medium confidence)	AZOBR_p230024	Ancestral (medium confidence)
AZOLI_p40520	Unassigned	AZOBR_p230025	Unassigned
AZOLI_p40521	Ancestral (low confidence)	AZOBR_p230026	Horizontally Transferred (low confidence)
AZOLI_p40522	Horizontally Transferred (high confidence)	AZOBR_p230027	Ancestral (high confidence)
AZOLI_p40523	Unassigned	AZOBR_p230028	Horizontally Transferred (medium confidence)
AZOLI_p40524	Ancestral (low confidence)	AZOBR_p230029	Ancestral (low confidence)
AZOLI_p40525	Horizontally Transferred (high confidence)	AZOBR_p230030	Ancestral (low confidence)
AZOLI_p40527	Ancestral (low confidence)	AZOBR_p230031	Ancestral (low confidence)
AZOLI_p40529	Ancestral (high confidence)	AZOBR_p230032	Ancestral (low confidence)
AZOLI_p40530	Ancestral (high confidence)	AZOBR_p230033	Unassigned
AZOLI_p40531	Ancestral (high confidence)	AZOBR_p230034	Unassigned
AZOLI_p40532	Ancestral (high confidence)	AZOBR_p230035	Ancestral (high confidence)
AZOLI_p40534	Unassigned	AZOBR_p230036	Horizontally Transferred (high confidence)
AZOLI_p40535	Unassigned	AZOBR_p230037	Ancestral (medium confidence)
AZOLI_p40536	Ancestral (medium confidence)	AZOBR_p230038	Ancestral (medium confidence)
AZOLI_p40537	Ancestral (high confidence)	AZOBR_p230039	Ancestral (low confidence)
AZOLI_p40538	Ancestral (high confidence)	AZOBR_p230040	Unassigned
AZOLI_p40539	Ancestral (high confidence)	AZOBR_p230041	Horizontally Transferred (low confidence)
AZOLI_p40540	Ancestral (low confidence)	AZOBR_p230042	Ancestral (medium confidence)
AZOLI_p40541	Ancestral (low confidence)	AZOBR_p230043	Horizontally Transferred (high confidence)
AZOLI_p40544	Horizontally Transferred (high confidence)	AZOBR_p230044	Horizontally Transferred (high confidence)
AZOLI_p40546	Ancestral (low confidence)	AZOBR_p230045	Horizontally Transferred (high confidence)
AZOLI_p40547	Ancestral (low confidence)	AZOBR_p230046	Ancestral (high confidence)
AZOLI_p40548	Horizontally Transferred (low confidence)	AZOBR_p230047	Unassigned
AZOLI_p40549	Ancestral (medium confidence)	AZOBR_p230048	Horizontally Transferred (high confidence)
AZOLI_p40550	Horizontally Transferred (high confidence)	AZOBR_p230049	Unassigned
AZOLI_p40553	Horizontally Transferred (high confidence)	AZOBR_p230050	Horizontally Transferred (high confidence)
AZOLI_p40554	Horizontally Transferred (high confidence)	AZOBR_p230051	Ancestral (low confidence)
AZOLI_p40556	Horizontally Transferred (high confidence)	AZOBR_p230052	Unassigned
AZOLI_p40557	Horizontally Transferred (medium confidence)	AZOBR_p230053	Ancestral (low confidence)
AZOLI_p40558	Horizontally Transferred (high confidence)	AZOBR_p230054	Ancestral (low confidence)
AZOLI_p40560	Horizontally Transferred (high confidence)	AZOBR_p230055	Unassigned
AZOLI_p40561	Horizontally Transferred (high confidence)	AZOBR_p230056	Unassigned
AZOLI_p40562	Horizontally Transferred (high confidence)	AZOBR_p230057	Unassigned
AZOLI_p40563	Unassigned	AZOBR_p230058	Unassigned
AZOLI_p40565	Horizontally Transferred (high confidence)	AZOBR_p230059	Ancestral (low confidence)
AZOLI_p40566	Ancestral (medium confidence)	AZOBR_p230060	Horizontally Transferred (high confidence)
AZOLI_p40568	Unassigned	AZOBR_p230061	Horizontally Transferred (high confidence)
AZOLI_p40569	Horizontally Transferred (high confidence)	AZOBR_p230062	Ancestral (low confidence)
AZOLI_p40570	Unassigned	AZOBR_p230063	Ancestral (low confidence)
AZOLI_p40571	Unassigned	AZOBR_p230064	Horizontally Transferred (high confidence)
AZOLI_p40575	Unassigned	AZOBR_p230065	Horizontally Transferred (low confidence)
AZOLI_p40576	Horizontally Transferred (medium confidence)	AZOBR_p230066	Horizontally Transferred (high confidence)
AZOLI_p40577	Unassigned	AZOBR_p230067	Ancestral (low confidence)
AZOLI_p40578	Horizontally Transferred (high confidence)	AZOBR_p230068	Horizontally Transferred (high confidence)
AZOLI_p40579	Ancestral (low confidence)	AZOBR_p230069	Horizontally Transferred (high confidence)
AZOLI_p40580	Ancestral (low confidence)	AZOBR_p230070	Horizontally Transferred (high confidence)
AZOLI_p40581	Horizontally Transferred (high confidence)	AZOBR_p230071	Horizontally Transferred (high confidence)
AZOLI_p40582	Unassigned	AZOBR_p230072	Unassigned
AZOLI_p40583	Horizontally Transferred (high confidence)	AZOBR_p230073	Horizontally Transferred (medium confidence)
AZOLI_p40584	Horizontally Transferred (high confidence)	AZOBR_p230074	Unassigned
AZOLI_p40585	Horizontally Transferred (high confidence)	AZOBR_p230075	Horizontally Transferred (high confidence)
AZOLI_p40586	Horizontally Transferred (high confidence)	AZOBR_p230076	Unassigned
AZOLI_p40589	Horizontally Transferred (medium confidence)	AZOBR_p230077	Unassigned
AZOLI_p40590	Horizontally Transferred (high confidence)	AZOBR_p230078	Horizontally Transferred (high confidence)
AZOLI_p40591	Horizontally Transferred (high confidence)	AZOBR_p230079	Horizontally Transferred (high confidence)
AZOLI_p40592	Horizontally Transferred (high confidence)	AZOBR_p230080	Horizontally Transferred (high confidence)
AZOLI_p40593	Horizontally Transferred (high confidence)	AZOBR_p230081	Horizontally Transferred (high confidence)
AZOLI_p40594	Ancestral (low confidence)	AZOBR_p230082	Horizontally Transferred (high confidence)
AZOLI_p40595	Horizontally Transferred (high confidence)	AZOBR_p230083	Unassigned
AZOLI_p40596	Unassigned	AZOBR_p230084	Ancestral (low confidence)
AZOLI_p40597	Horizontally Transferred (high confidence)	AZOBR_p230085	Horizontally Transferred (high confidence)
AZOLI_p40598	Horizontally Transferred (high confidence)	AZOBR_p230086	Ancestral (medium confidence)
AZOLI_p40599	Ancestral (medium confidence)	AZOBR_p230087	Ancestral (medium confidence)
AZOLI_p40600	Ancestral (low confidence)	AZOBR_p230088	Ancestral (medium confidence)
AZOLI_p40601	Ancestral (low confidence)	AZOBR_p230089	Horizontally Transferred (high confidence)
AZOLI_p40603	Unassigned	AZOBR_p230090	Unassigned
AZOLI_p40604	Ancestral (low confidence)	AZOBR_p230091	Unassigned
AZOLI_p40605	Horizontally Transferred (high confidence)	AZOBR_p230092	Horizontally Transferred (high confidence)
AZOLI_p40606	Horizontally Transferred (high confidence)	AZOBR_p230093	Horizontally Transferred (high confidence)
AZOLI_p40607	Horizontally Transferred (low confidence)	AZOBR_p230094	Horizontally Transferred (high confidence)
AZOLI_p40608	Horizontally Transferred (high confidence)	AZOBR_p230095	Horizontally Transferred (high confidence)
AZOLI_p40609	Horizontally Transferred (high confidence)	AZOBR_p230096	Horizontally Transferred (low confidence)
AZOLI_p40610	Horizontally Transferred (high confidence)	AZOBR_p230097	Horizontally Transferred (high confidence)

AZOLI_p40611	Ancestral (high confidence)	AZOBR_p230098	Horizontally Transferred (low confidence)
AZOLI_p40612	Ancestral (low confidence)	AZOBR_p240001	Horizontally Transferred (high confidence)
AZOLI_p40614	Ancestral (medium confidence)	AZOBR_p240002	Horizontally Transferred (high confidence)
AZOLI_p40615	Ancestral (low confidence)	AZOBR_p250001	Horizontally Transferred (high confidence)
AZOLI_p40616	Ancestral (medium confidence)	AZOBR_p250002	Unassigned
AZOLI_p40617	Unassigned	AZOBR_p250003	Unassigned
AZOLI_p40619	Horizontally Transferred (high confidence)	AZOBR_p250004	Unassigned
AZOLI_p40620	Ancestral (medium confidence)	AZOBR_p250005	Ancestral (high confidence)
AZOLI_p40621	Ancestral (high confidence)	AZOBR_p250006	Unassigned
AZOLI_p40622	Unassigned	AZOBR_p250007	Ancestral (high confidence)
AZOLI_p40624	Ancestral (medium confidence)	AZOBR_p250008	Horizontally Transferred (medium confidence)
AZOLI_p40625	Unassigned	AZOBR_p250009	Horizontally Transferred (high confidence)
AZOLI_p40626	Ancestral (medium confidence)	AZOBR_p250010	Horizontally Transferred (high confidence)
AZOLI_p40629	Ancestral (medium confidence)	AZOBR_p250011	Horizontally Transferred (high confidence)
AZOLI_p40630	Ancestral (low confidence)	AZOBR_p250012	Ancestral (low confidence)
AZOLI_p40631	Ancestral (low confidence)	AZOBR_p250013	Horizontally Transferred (high confidence)
AZOLI_p40632	Ancestral (high confidence)	AZOBR_p250014	Unassigned
AZOLI_p40633	Ancestral (high confidence)	AZOBR_p260001	Horizontally Transferred (high confidence)
AZOLI_p40634	Ancestral (high confidence)	AZOBR_p260002	Unassigned
AZOLI_p40635	Horizontally Transferred (high confidence)	AZOBR_p260003	Horizontally Transferred (high confidence)
AZOLI_p40637	Ancestral (high confidence)	AZOBR_p260004	Unassigned
AZOLI_p40638	Ancestral (high confidence)	AZOBR_p260005	Unassigned
AZOLI_p40640	Horizontally Transferred (high confidence)	AZOBR_p260006	Unassigned
AZOLI_p40641	Ancestral (low confidence)	AZOBR_p260007	Horizontally Transferred (high confidence)
AZOLI_p40642	Unassigned	AZOBR_p260008	Ancestral (low confidence)
AZOLI_p40643	Ancestral (high confidence)	AZOBR_p260009	Horizontally Transferred (high confidence)
AZOLI_p40644	Horizontally Transferred (high confidence)	AZOBR_p260010	Unassigned
AZOLI_p40645	Horizontally Transferred (medium confidence)	AZOBR_p260011	Horizontally Transferred (high confidence)
AZOLI_p40646	Ancestral (medium confidence)	AZOBR_p260012	Ancestral (low confidence)
AZOLI_p40647	Ancestral (medium confidence)	AZOBR_p260013	Ancestral (low confidence)
AZOLI_p40648	Ancestral (medium confidence)	AZOBR_p260014	Ancestral (low confidence)
AZOLI_p40649	Ancestral (low confidence)	AZOBR_p260015	Unassigned
AZOLI_p40651	Ancestral (medium confidence)	AZOBR_p260016	Unassigned
AZOLI_p40653	Ancestral (medium confidence)	AZOBR_p260017	Unassigned
AZOLI_p40654	Ancestral (high confidence)	AZOBR_p260018	Horizontally Transferred (high confidence)
AZOLI_p40655	Ancestral (high confidence)	AZOBR_p260019	Ancestral (low confidence)
AZOLI_p40656	Ancestral (medium confidence)	AZOBR_p260020	Horizontally Transferred (medium confidence)
AZOLI_p40658	Ancestral (medium confidence)	AZOBR_p260021	Unassigned
AZOLI_p50001	Horizontally Transferred (high confidence)	AZOBR_p260022	Horizontally Transferred (high confidence)
AZOLI_p50002	Unassigned	AZOBR_p260023	Unassigned
AZOLI_p50003	Unassigned	AZOBR_p260024	Unassigned
AZOLI_p50004	Ancestral (low confidence)	AZOBR_p260025	Horizontally Transferred (high confidence)
AZOLI_p50005	Horizontally Transferred (high confidence)	AZOBR_p260026	Unassigned
AZOLI_p50006	Horizontally Transferred (high confidence)	AZOBR_p260027	Unassigned
AZOLI_p50007	Ancestral (high confidence)	AZOBR_p260028	Unassigned
AZOLI_p50008	Ancestral (low confidence)	AZOBR_p260029	Unassigned
AZOLI_p50009	Ancestral (low confidence)	AZOBR_p260030	Unassigned
AZOLI_p50010	Ancestral (low confidence)	AZOBR_p260031	Ancestral (low confidence)
AZOLI_p50011	Ancestral (low confidence)	AZOBR_p270001	Unassigned
AZOLI_p50012	Ancestral (high confidence)	AZOBR_p270002	Unassigned
AZOLI_p50013	Ancestral (medium confidence)	AZOBR_p270003	Ancestral (medium confidence)
AZOLI_p50014	Ancestral (low confidence)	AZOBR_p270004	Ancestral (low confidence)
AZOLI_p50015	Ancestral (medium confidence)	AZOBR_p270005	Ancestral (medium confidence)
AZOLI_p50016	Ancestral (high confidence)	AZOBR_p270006	Ancestral (low confidence)
AZOLI_p50017	Ancestral (high confidence)	AZOBR_p270007	Unassigned
AZOLI_p50018	Ancestral (medium confidence)	AZOBR_p270008	Unassigned
AZOLI_p50019	Horizontally Transferred (high confidence)	AZOBR_p270009	Unassigned
AZOLI_p50020	Ancestral (high confidence)	AZOBR_p270010	Horizontally Transferred (high confidence)
AZOLI_p50021	Ancestral (low confidence)	AZOBR_p270011	Unassigned
AZOLI_p50022	Ancestral (high confidence)	AZOBR_p270012	Horizontally Transferred (high confidence)
AZOLI_p50023	Ancestral (medium confidence)	AZOBR_p270013	Unassigned
AZOLI_p50024	Ancestral (high confidence)	AZOBR_p270014	Ancestral (low confidence)
AZOLI_p50025	Ancestral (medium confidence)	AZOBR_p270015	Unassigned
AZOLI_p50026	Ancestral (medium confidence)	AZOBR_p270016	Ancestral (high confidence)
AZOLI_p50027	Ancestral (high confidence)	AZOBR_p270017	Ancestral (high confidence)
AZOLI_p50028	Ancestral (medium confidence)	AZOBR_p270018	Ancestral (low confidence)
AZOLI_p50029	Ancestral (medium confidence)	AZOBR_p270019	Ancestral (low confidence)
AZOLI_p50030	Horizontally Transferred (high confidence)	AZOBR_p270020	Ancestral (low confidence)
AZOLI_p50031	Ancestral (high confidence)	AZOBR_p270021	Unassigned
AZOLI_p50032	Horizontally Transferred (high confidence)	AZOBR_p270022	Horizontally Transferred (low confidence)
AZOLI_p50033	Horizontally Transferred (high confidence)	AZOBR_p270023	Horizontally Transferred (high confidence)
AZOLI_p50035	Ancestral (medium confidence)	AZOBR_p270024	Ancestral (low confidence)
AZOLI_p50036	Unassigned	AZOBR_p270025	Horizontally Transferred (high confidence)
AZOLI_p50037	Ancestral (medium confidence)	AZOBR_p270026	Ancestral (low confidence)
AZOLI_p50038	Ancestral (low confidence)	AZOBR_p270027	Ancestral (low confidence)

AZOLI_p50039	Ancestral (medium confidence)	AZOBR_p270028	Unassigned
AZOLI_p50040	Unassigned	AZOBR_p270029	Horizontally Transferred (high confidence)
AZOLI_p50041	Ancestral (high confidence)	AZOBR_p270030	Horizontally Transferred (high confidence)
AZOLI_p50042	Ancestral (low confidence)	AZOBR_p270031	Horizontally Transferred (high confidence)
AZOLI_p50043	Ancestral (high confidence)	AZOBR_p270032	Horizontally Transferred (high confidence)
AZOLI_p50044	Ancestral (high confidence)	AZOBR_p270033	Horizontally Transferred (medium confidence)
AZOLI_p50045	Ancestral (high confidence)	AZOBR_p270034	Unassigned
AZOLI_p50046	Ancestral (medium confidence)	AZOBR_p270035	Horizontally Transferred (medium confidence)
AZOLI_p50047	Ancestral (high confidence)	AZOBR_p270036	Horizontally Transferred (high confidence)
AZOLI_p50048	Ancestral (low confidence)	AZOBR_p270037	Horizontally Transferred (medium confidence)
AZOLI_p50049	Ancestral (high confidence)	AZOBR_p270038	Unassigned
AZOLI_p50050	Unassigned	AZOBR_p270039	Ancestral (low confidence)
AZOLI_p50054	Ancestral (low confidence)	AZOBR_p270040	Horizontally Transferred (high confidence)
AZOLI_p50055	Horizontally Transferred (low confidence)	AZOBR_p270041	Unassigned
AZOLI_p50056	Unassigned	AZOBR_p270042	Unassigned
AZOLI_p50057	Ancestral (high confidence)	AZOBR_p270043	Horizontally Transferred (high confidence)
AZOLI_p50058	Ancestral (high confidence)	AZOBR_p270044	Horizontally Transferred (high confidence)
AZOLI_p50059	Ancestral (medium confidence)	AZOBR_p270045	Unassigned
AZOLI_p50060	Ancestral (high confidence)	AZOBR_p270046	Horizontally Transferred (high confidence)
AZOLI_p50061	Ancestral (high confidence)	AZOBR_p270047	Horizontally Transferred (high confidence)
AZOLI_p50062	Ancestral (high confidence)	AZOBR_p270048	Horizontally Transferred (high confidence)
AZOLI_p50063	Horizontally Transferred (high confidence)	AZOBR_p270049	Horizontally Transferred (high confidence)
AZOLI_p50064	Horizontally Transferred (low confidence)	AZOBR_p270050	Horizontally Transferred (high confidence)
AZOLI_p50065	Unassigned	AZOBR_p270051	Horizontally Transferred (high confidence)
AZOLI_p50066	Ancestral (low confidence)	AZOBR_p270052	Horizontally Transferred (high confidence)
AZOLI_p50067	Horizontally Transferred (high confidence)	AZOBR_p270053	Horizontally Transferred (high confidence)
AZOLI_p50068	Unassigned	AZOBR_p270054	Horizontally Transferred (high confidence)
AZOLI_p50070	Horizontally Transferred (high confidence)	AZOBR_p270055	Ancestral (low confidence)
AZOLI_p50071	Horizontally Transferred (high confidence)	AZOBR_p270056	Horizontally Transferred (low confidence)
AZOLI_p50072	Horizontally Transferred (high confidence)	AZOBR_p270057	Ancestral (high confidence)
AZOLI_p50073	Unassigned	AZOBR_p270058	Unassigned
AZOLI_p50074	Unassigned	AZOBR_p270059	Ancestral (medium confidence)
AZOLI_p50075	Ancestral (low confidence)	AZOBR_p270060	Ancestral (high confidence)
AZOLI_p50076	Ancestral (low confidence)	AZOBR_p270061	Ancestral (medium confidence)
AZOLI_p50077	Ancestral (high confidence)	AZOBR_p270062	Ancestral (high confidence)
AZOLI_p50078	Ancestral (high confidence)	AZOBR_p270063	Unassigned
AZOLI_p50079	Ancestral (medium confidence)	AZOBR_p270064	Unassigned
AZOLI_p50080	Horizontally Transferred (high confidence)	AZOBR_p270065	Unassigned
AZOLI_p50081	Horizontally Transferred (high confidence)	AZOBR_p270066	Unassigned
AZOLI_p50082	Horizontally Transferred (high confidence)	AZOBR_p270067	Unassigned
AZOLI_p50083	Horizontally Transferred (high confidence)	AZOBR_p270068	Ancestral (low confidence)
AZOLI_p50084	Horizontally Transferred (low confidence)	AZOBR_p270069	Ancestral (low confidence)
AZOLI_p50085	Ancestral (low confidence)	AZOBR_p270070	Ancestral (low confidence)
AZOLI_p50086	Unassigned	AZOBR_p270071	Ancestral (low confidence)
AZOLI_p50087	Horizontally Transferred (medium confidence)	AZOBR_p270072	Horizontally Transferred (high confidence)
AZOLI_p50088	Horizontally Transferred (high confidence)	AZOBR_p270073	Horizontally Transferred (high confidence)
AZOLI_p50089	Horizontally Transferred (high confidence)	AZOBR_p270074	Unassigned
AZOLI_p50090	Ancestral (medium confidence)	AZOBR_p270075	Ancestral (low confidence)
AZOLI_p50091	Unassigned	AZOBR_p270076	Unassigned
AZOLI_p50092	Horizontally Transferred (high confidence)	AZOBR_p270077	Horizontally Transferred (low confidence)
AZOLI_p50093	Horizontally Transferred (high confidence)	AZOBR_p270078	Horizontally Transferred (high confidence)
AZOLI_p50094	Ancestral (medium confidence)	AZOBR_p270079	Horizontally Transferred (low confidence)
AZOLI_p50095	Unassigned	AZOBR_p270080	Horizontally Transferred (medium confidence)
AZOLI_p50096	Ancestral (low confidence)	AZOBR_p270081	Horizontally Transferred (low confidence)
AZOLI_p50097	Unassigned	AZOBR_p270082	Horizontally Transferred (medium confidence)
AZOLI_p50098	Ancestral (low confidence)	AZOBR_p270083	Horizontally Transferred (low confidence)
AZOLI_p50099	Ancestral (medium confidence)	AZOBR_p270084	Unassigned
AZOLI_p50100	Unassigned	AZOBR_p270085	Unassigned
AZOLI_p50101	Horizontally Transferred (high confidence)	AZOBR_p270086	Ancestral (low confidence)
AZOLI_p50102	Unassigned	AZOBR_p270087	Horizontally Transferred (high confidence)
AZOLI_p50103	Horizontally Transferred (high confidence)	AZOBR_p270088	Horizontally Transferred (medium confidence)
AZOLI_p50104	Ancestral (low confidence)	AZOBR_p270089	Horizontally Transferred (high confidence)
AZOLI_p50105	Horizontally Transferred (high confidence)	AZOBR_p270090	Horizontally Transferred (low confidence)
AZOLI_p50106	Horizontally Transferred (high confidence)	AZOBR_p270091	Horizontally Transferred (low confidence)
AZOLI_p50107	Horizontally Transferred (high confidence)	AZOBR_p270092	Ancestral (low confidence)
AZOLI_p50108	Horizontally Transferred (high confidence)	AZOBR_p270093	Horizontally Transferred (low confidence)
AZOLI_p50109	Horizontally Transferred (high confidence)	AZOBR_p270094	Horizontally Transferred (low confidence)
AZOLI_p50110	Horizontally Transferred (high confidence)	AZOBR_p270095	Unassigned
AZOLI_p50111	Ancestral (medium confidence)	AZOBR_p270096	Unassigned
AZOLI_p50112	Ancestral (low confidence)	AZOBR_p270097	Horizontally Transferred (low confidence)
AZOLI_p50113	Horizontally Transferred (high confidence)	AZOBR_p270098	Horizontally Transferred (medium confidence)
AZOLI_p50114	Ancestral (low confidence)	AZOBR_p270099	Horizontally Transferred (medium confidence)
AZOLI_p50115	Horizontally Transferred (low confidence)	AZOBR_p270100	Horizontally Transferred (medium confidence)
AZOLI_p50116	Ancestral (low confidence)	AZOBR_p270101	Ancestral (high confidence)
AZOLI_p50117	Ancestral (low confidence)	AZOBR_p270102	Horizontally Transferred (high confidence)

AZOLI_p50118	Unassigned	AZOBR_p270103	Ancestral (low confidence)
AZOLI_p50119	Unassigned	AZOBR_p270104	Horizontally Transferred (low confidence)
AZOLI_p50120	Unassigned	AZOBR_p270105	Horizontally Transferred (medium confidence)
AZOLI_p50121	Unassigned	AZOBR_p270106	Horizontally Transferred (low confidence)
AZOLI_p50122	Horizontally Transferred (high confidence)	AZOBR_p270107	Unassigned
AZOLI_p50123	Horizontally Transferred (high confidence)	AZOBR_p270108	Horizontally Transferred (high confidence)
AZOLI_p50127	Unassigned	AZOBR_p270109	Unassigned
AZOLI_p50128	Horizontally Transferred (high confidence)	AZOBR_p270110	Unassigned
AZOLI_p50129	Ancestral (low confidence)	AZOBR_p270111	Unassigned
AZOLI_p50130	Ancestral (low confidence)	AZOBR_p270112	Horizontally Transferred (high confidence)
AZOLI_p50131	Horizontally Transferred (high confidence)	AZOBR_p270113	Horizontally Transferred (medium confidence)
AZOLI_p50132	Horizontally Transferred (low confidence)	AZOBR_p270114	Unassigned
AZOLI_p50133	Horizontally Transferred (low confidence)	AZOBR_p270115	Unassigned
AZOLI_p50134	Horizontally Transferred (high confidence)	AZOBR_p270116	Ancestral (low confidence)
AZOLI_p50135	Horizontally Transferred (low confidence)	AZOBR_p270117	Ancestral (low confidence)
AZOLI_p50136	Horizontally Transferred (high confidence)	AZOBR_p270118	Horizontally Transferred (high confidence)
AZOLI_p50137	Unassigned	AZOBR_p270119	Horizontally Transferred (high confidence)
AZOLI_p50138	Ancestral (low confidence)	AZOBR_p270120	Ancestral (low confidence)
AZOLI_p50140	Horizontally Transferred (high confidence)	AZOBR_p270121	Horizontally Transferred (medium confidence)
AZOLI_p50141	Horizontally Transferred (high confidence)	AZOBR_p270122	Horizontally Transferred (high confidence)
AZOLI_p50142	Ancestral (low confidence)	AZOBR_p270123	Horizontally Transferred (high confidence)
AZOLI_p50143	Ancestral (low confidence)	AZOBR_p270124	Horizontally Transferred (high confidence)
AZOLI_p50144	Ancestral (low confidence)	AZOBR_p270125	Horizontally Transferred (high confidence)
AZOLI_p50146	Horizontally Transferred (high confidence)	AZOBR_p270126	Ancestral (low confidence)
AZOLI_p50147	Horizontally Transferred (high confidence)	AZOBR_p270127	Horizontally Transferred (high confidence)
AZOLI_p50148	Horizontally Transferred (high confidence)	AZOBR_p270128	Horizontally Transferred (high confidence)
AZOLI_p50149	Unassigned	AZOBR_p270129	Horizontally Transferred (low confidence)
AZOLI_p50150	Unassigned	AZOBR_p270130	Horizontally Transferred (low confidence)
AZOLI_p50151	Unassigned	AZOBR_p270131	Ancestral (low confidence)
AZOLI_p50152	Unassigned	AZOBR_p270132	Horizontally Transferred (medium confidence)
AZOLI_p50153	Ancestral (low confidence)	AZOBR_p270133	Horizontally Transferred (high confidence)
AZOLI_p50154	Horizontally Transferred (high confidence)	AZOBR_p270134	Unassigned
AZOLI_p50155	Ancestral (high confidence)	AZOBR_p270135	Horizontally Transferred (high confidence)
AZOLI_p50156	Horizontally Transferred (high confidence)	AZOBR_p270136	Horizontally Transferred (high confidence)
AZOLI_p50157	Ancestral (high confidence)	AZOBR_p270137	Horizontally Transferred (high confidence)
AZOLI_p50158	Ancestral (medium confidence)	AZOBR_p270138	Horizontally Transferred (high confidence)
AZOLI_p50159	Ancestral (high confidence)	AZOBR_p270139	Horizontally Transferred (high confidence)
AZOLI_p50160	Ancestral (medium confidence)	AZOBR_p270140	Horizontally Transferred (high confidence)
AZOLI_p50161	Ancestral (medium confidence)	AZOBR_p270141	Horizontally Transferred (high confidence)
AZOLI_p50162	Ancestral (high confidence)	AZOBR_p270142	Horizontally Transferred (high confidence)
AZOLI_p50163	Ancestral (high confidence)	AZOBR_p270143	Ancestral (low confidence)
AZOLI_p50164	Ancestral (medium confidence)	AZOBR_p270144	Horizontally Transferred (high confidence)
AZOLI_p50165	Ancestral (medium confidence)	AZOBR_p270145	Ancestral (low confidence)
AZOLI_p50166	Ancestral (high confidence)	AZOBR_p270146	Horizontally Transferred (high confidence)
AZOLI_p50167	Ancestral (high confidence)	AZOBR_p270147	Horizontally Transferred (medium confidence)
AZOLI_p50168	Ancestral (medium confidence)	AZOBR_p270148	Ancestral (low confidence)
AZOLI_p50169	Ancestral (low confidence)	AZOBR_p270149	Ancestral (low confidence)
AZOLI_p50170	Horizontally Transferred (high confidence)	AZOBR_p270150	Ancestral (medium confidence)
AZOLI_p50171	Ancestral (low confidence)	AZOBR_p270151	Ancestral (medium confidence)
AZOLI_p50172	Unassigned	AZOBR_p270152	Ancestral (medium confidence)
AZOLI_p50173	Unassigned	AZOBR_p270153	Ancestral (medium confidence)
AZOLI_p50174	Ancestral (high confidence)	AZOBR_p270154	Horizontally Transferred (low confidence)
AZOLI_p50175	Horizontally Transferred (high confidence)	AZOBR_p270155	Unassigned
AZOLI_p50176	Ancestral (medium confidence)	AZOBR_p270156	Horizontally Transferred (high confidence)
AZOLI_p50177	Horizontally Transferred (high confidence)	AZOBR_p270157	Ancestral (low confidence)
AZOLI_p50178	Ancestral (medium confidence)	AZOBR_p270158	Unassigned
AZOLI_p50179	Unassigned	AZOBR_p270159	Ancestral (low confidence)
AZOLI_p50180	Horizontally Transferred (high confidence)	AZOBR_p270160	Ancestral (low confidence)
AZOLI_p50181	Unassigned	AZOBR_p270161	Ancestral (medium confidence)
AZOLI_p50182	Horizontally Transferred (high confidence)	AZOBR_p270162	Ancestral (low confidence)
AZOLI_p50183	Horizontally Transferred (high confidence)	AZOBR_p270163	Unassigned
AZOLI_p50184	Horizontally Transferred (low confidence)	AZOBR_p270164	Horizontally Transferred (medium confidence)
AZOLI_p50186	Ancestral (high confidence)	AZOBR_p270165	Unassigned
AZOLI_p50187	Horizontally Transferred (medium confidence)	AZOBR_p270166	Horizontally Transferred (high confidence)
AZOLI_p50188	Horizontally Transferred (high confidence)	AZOBR_p270167	Unassigned
AZOLI_p50190	Horizontally Transferred (low confidence)	AZOBR_p270168	Unassigned
AZOLI_p50191	Horizontally Transferred (high confidence)	AZOBR_p270169	Unassigned
AZOLI_p50192	Horizontally Transferred (high confidence)	AZOBR_p270170	Unassigned
AZOLI_p50193	Horizontally Transferred (high confidence)	AZOBR_p270171	Horizontally Transferred (high confidence)
AZOLI_p50194	Horizontally Transferred (high confidence)	AZOBR_p270172	Unassigned
AZOLI_p50195	Horizontally Transferred (high confidence)	AZOBR_p270173	Horizontally Transferred (high confidence)
AZOLI_p50196	Horizontally Transferred (high confidence)	AZOBR_p270174	Horizontally Transferred (high confidence)
AZOLI_p50197	Horizontally Transferred (low confidence)	AZOBR_p270175	Unassigned
AZOLI_p50198	Unassigned	AZOBR_p270176	Ancestral (medium confidence)
AZOLI_p50199	Unassigned	AZOBR_p270177	Ancestral (medium confidence)

AZOLI_p50200	Horizontally Transferred (medium confidence)	AZOBR_p270178	Unassigned
AZOLI_p50201	Horizontally Transferred (high confidence)	AZOBR_p270179	Unassigned
AZOLI_p50202	Ancestral (high confidence)	AZOBR_p270180	Unassigned
AZOLI_p50203	Ancestral (medium confidence)	AZOBR_p270181	Ancestral (low confidence)
AZOLI_p50204	Horizontally Transferred (medium confidence)	AZOBR_p270182	Unassigned
AZOLI_p50205	Horizontally Transferred (high confidence)	AZOBR_p270183	Unassigned
AZOLI_p50206	Unassigned	AZOBR_p270184	Unassigned
AZOLI_p50207	Ancestral (high confidence)	AZOBR_p270185	Unassigned
AZOLI_p50208	Ancestral (low confidence)	AZOBR_p270186	Unassigned
AZOLI_p50209	Horizontally Transferred (high confidence)	AZOBR_p270187	Horizontally Transferred (high confidence)
AZOLI_p50210	Ancestral (high confidence)	AZOBR_p270188	Unassigned
AZOLI_p50211	Ancestral (medium confidence)	AZOBR_p270189	Unassigned
AZOLI_p50212	Ancestral (medium confidence)	AZOBR_p270190	Horizontally Transferred (high confidence)
AZOLI_p50213	Ancestral (medium confidence)	AZOBR_p270191	Ancestral (low confidence)
AZOLI_p50215	Ancestral (high confidence)	AZOBR_p270192	Unassigned
AZOLI_p50216	Ancestral (medium confidence)	AZOBR_p270193	Ancestral (high confidence)
AZOLI_p50217	Horizontally Transferred (high confidence)	AZOBR_p270194	Ancestral (low confidence)
AZOLI_p50218	Ancestral (medium confidence)	AZOBR_p270195	Unassigned
AZOLI_p50219	Horizontally Transferred (high confidence)	AZOBR_p270196	Ancestral (low confidence)
AZOLI_p50220	Ancestral (medium confidence)	AZOBR_p270197	Unassigned
AZOLI_p50221	Horizontally Transferred (high confidence)	AZOBR_p270198	Unassigned
AZOLI_p50222	Ancestral (low confidence)	AZOBR_p270199	Unassigned
AZOLI_p50223	Ancestral (medium confidence)	AZOBR_p270200	Ancestral (low confidence)
AZOLI_p50224	Horizontally Transferred (high confidence)	AZOBR_p270201	Unassigned
AZOLI_p50225	Horizontally Transferred (high confidence)	AZOBR_p270202	Unassigned
AZOLI_p50226	Horizontally Transferred (high confidence)	AZOBR_p270203	Horizontally Transferred (high confidence)
AZOLI_p50227	Ancestral (low confidence)	AZOBR_p270204	Horizontally Transferred (high confidence)
AZOLI_p50228	Ancestral (medium confidence)	AZOBR_p270205	Horizontally Transferred (high confidence)
AZOLI_p50229	Ancestral (low confidence)	AZOBR_p270206	Unassigned
AZOLI_p50230	Ancestral (low confidence)	AZOBR_p270207	Unassigned
AZOLI_p50231	Horizontally Transferred (high confidence)	AZOBR_p270208	Horizontally Transferred (high confidence)
AZOLI_p50232	Horizontally Transferred (high confidence)	AZOBR_p270209	Unassigned
AZOLI_p50233	Horizontally Transferred (high confidence)	AZOBR_p270210	Horizontally Transferred (high confidence)
AZOLI_p50234	Ancestral (low confidence)	AZOBR_p270211	Ancestral (medium confidence)
AZOLI_p50235	Ancestral (high confidence)	AZOBR_p270212	Horizontally Transferred (high confidence)
AZOLI_p50236	Ancestral (medium confidence)	AZOBR_p270213	Unassigned
AZOLI_p50237	Ancestral (medium confidence)	AZOBR_p270214	Unassigned
AZOLI_p50238	Ancestral (low confidence)	AZOBR_p270215	Unassigned
AZOLI_p50239	Ancestral (medium confidence)	AZOBR_p270216	Unassigned
AZOLI_p50240	Ancestral (high confidence)	AZOBR_p270217	Unassigned
AZOLI_p50241	Ancestral (medium confidence)	AZOBR_p270218	Unassigned
AZOLI_p50242	Ancestral (high confidence)	AZOBR_p270219	Unassigned
AZOLI_p50243	Ancestral (medium confidence)	AZOBR_p270220	Ancestral (medium confidence)
AZOLI_p50244	Horizontally Transferred (high confidence)	AZOBR_p270221	Unassigned
AZOLI_p50245	Horizontally Transferred (high confidence)	AZOBR_p270222	Ancestral (medium confidence)
AZOLI_p50246	Ancestral (medium confidence)	AZOBR_p270223	Ancestral (high confidence)
AZOLI_p50247	Ancestral (high confidence)	AZOBR_p270224	Ancestral (medium confidence)
AZOLI_p50248	Ancestral (high confidence)	AZOBR_p270225	Ancestral (high confidence)
AZOLI_p50249	Ancestral (high confidence)	AZOBR_p270226	Ancestral (low confidence)
AZOLI_p50250	Ancestral (medium confidence)	AZOBR_p270227	Ancestral (medium confidence)
AZOLI_p50251	Ancestral (medium confidence)	AZOBR_p270228	Horizontally Transferred (high confidence)
AZOLI_p50252	Ancestral (high confidence)	AZOBR_p270229	Horizontally Transferred (medium confidence)
AZOLI_p50253	Ancestral (medium confidence)	AZOBR_p270230	Horizontally Transferred (medium confidence)
AZOLI_p50254	Horizontally Transferred (high confidence)	AZOBR_p270231	Horizontally Transferred (medium confidence)
AZOLI_p50255	Horizontally Transferred (medium confidence)	AZOBR_p270232	Horizontally Transferred (high confidence)
AZOLI_p50256	Ancestral (low confidence)	AZOBR_p270233	Horizontally Transferred (high confidence)
AZOLI_p50257	Ancestral (medium confidence)	AZOBR_p270234	Horizontally Transferred (high confidence)
AZOLI_p50258	Ancestral (medium confidence)	AZOBR_p270235	Unassigned
AZOLI_p50259	Ancestral (high confidence)	AZOBR_p270236	Ancestral (low confidence)
AZOLI_p50260	Ancestral (medium confidence)	AZOBR_p270237	Ancestral (low confidence)
AZOLI_p50261	Ancestral (medium confidence)	AZOBR_p270238	Ancestral (low confidence)
AZOLI_p50262	Ancestral (medium confidence)	AZOBR_p270239	Ancestral (low confidence)
AZOLI_p50263	Ancestral (low confidence)	AZOBR_p270240	Horizontally Transferred (medium confidence)
AZOLI_p50264	Ancestral (low confidence)	AZOBR_p270241	Horizontally Transferred (low confidence)
AZOLI_p50265	Ancestral (high confidence)	AZOBR_p270242	Horizontally Transferred (high confidence)
AZOLI_p50266	Horizontally Transferred (high confidence)	AZOBR_p270243	Horizontally Transferred (high confidence)
AZOLI_p50267	Horizontally Transferred (high confidence)	AZOBR_p270244	Horizontally Transferred (high confidence)
AZOLI_p50268	Horizontally Transferred (high confidence)	AZOBR_p270245	Horizontally Transferred (high confidence)
AZOLI_p50269	Horizontally Transferred (high confidence)	AZOBR_p270246	Horizontally Transferred (medium confidence)
AZOLI_p50270	Horizontally Transferred (high confidence)	AZOBR_p270247	Horizontally Transferred (high confidence)
AZOLI_p50272	Horizontally Transferred (high confidence)	AZOBR_p270248	Horizontally Transferred (high confidence)
AZOLI_p50273	Ancestral (medium confidence)	AZOBR_p270249	Horizontally Transferred (high confidence)
AZOLI_p50274	Ancestral (medium confidence)	AZOBR_p270250	Horizontally Transferred (high confidence)
AZOLI_p50275	Unassigned	AZOBR_p270251	Ancestral (low confidence)
AZOLI_p50276	Horizontally Transferred (high confidence)	AZOBR_p270252	Horizontally Transferred (high confidence)

AZOLI_p50277	Horizontally Transferred (low confidence)	AZOBR_p270253	Ancestral (low confidence)
AZOLI_p50278	Horizontally Transferred (high confidence)	AZOBR_p270254	Horizontally Transferred (high confidence)
AZOLI_p50279	Horizontally Transferred (high confidence)	AZOBR_p270255	Horizontally Transferred (high confidence)
AZOLI_p50280	Ancestral (high confidence)	AZOBR_p270256	Unassigned
AZOLI_p50281	Horizontally Transferred (high confidence)	AZOBR_p270257	Horizontally Transferred (high confidence)
AZOLI_p50282	Ancestral (medium confidence)	AZOBR_p270258	Horizontally Transferred (high confidence)
AZOLI_p50283	Ancestral (high confidence)	AZOBR_p270259	Unassigned
AZOLI_p50284	Ancestral (high confidence)	AZOBR_p270260	Horizontally Transferred (medium confidence)
AZOLI_p50285	Ancestral (high confidence)	AZOBR_p270261	Horizontally Transferred (medium confidence)
AZOLI_p50286	Ancestral (high confidence)	AZOBR_p270262	Horizontally Transferred (high confidence)
AZOLI_p50287	Ancestral (high confidence)	AZOBR_p270263	Horizontally Transferred (high confidence)
AZOLI_p50288	Ancestral (high confidence)	AZOBR_p270264	Horizontally Transferred (high confidence)
AZOLI_p50290	Horizontally Transferred (high confidence)	AZOBR_p270265	Ancestral (low confidence)
AZOLI_p50291	Ancestral (medium confidence)	AZOBR_p270266	Horizontally Transferred (high confidence)
AZOLI_p50292	Unassigned	AZOBR_p270267	Horizontally Transferred (medium confidence)
AZOLI_p50293	Unassigned	AZOBR_p270268	Ancestral (low confidence)
AZOLI_p50294	Unassigned	AZOBR_p270269	Ancestral (low confidence)
AZOLI_p50295	Ancestral (medium confidence)	AZOBR_p270270	Horizontally Transferred (high confidence)
AZOLI_p50296	Ancestral (medium confidence)	AZOBR_p270271	Horizontally Transferred (high confidence)
AZOLI_p50297	Ancestral (medium confidence)	AZOBR_p270272	Horizontally Transferred (high confidence)
AZOLI_p50299	Ancestral (high confidence)	AZOBR_p270273	Horizontally Transferred (high confidence)
AZOLI_p50300	Ancestral (high confidence)	AZOBR_p270274	Horizontally Transferred (high confidence)
AZOLI_p50301	Ancestral (high confidence)	AZOBR_p270275	Ancestral (low confidence)
AZOLI_p50302	Horizontally Transferred (high confidence)	AZOBR_p270276	Ancestral (low confidence)
AZOLI_p50303	Ancestral (low confidence)	AZOBR_p270277	Horizontally Transferred (low confidence)
AZOLI_p50304	Horizontally Transferred (high confidence)	AZOBR_p270278	Unassigned
AZOLI_p50305	Horizontally Transferred (high confidence)	AZOBR_p270279	Horizontally Transferred (medium confidence)
AZOLI_p50306	Horizontally Transferred (low confidence)	AZOBR_p270280	Ancestral (high confidence)
AZOLI_p50307	Ancestral (low confidence)	AZOBR_p270281	Unassigned
AZOLI_p50308	Horizontally Transferred (high confidence)	AZOBR_p270282	Horizontally Transferred (medium confidence)
AZOLI_p50309	Horizontally Transferred (high confidence)	AZOBR_p270283	Ancestral (low confidence)
AZOLI_p50310	Horizontally Transferred (high confidence)	AZOBR_p270284	Ancestral (low confidence)
AZOLI_p50311	Horizontally Transferred (high confidence)	AZOBR_p270285	Unassigned
AZOLI_p50312	Horizontally Transferred (high confidence)	AZOBR_p270286	Unassigned
AZOLI_p50313	Horizontally Transferred (high confidence)	AZOBR_p270287	Ancestral (low confidence)
AZOLI_p50314	Ancestral (medium confidence)	AZOBR_p270288	Ancestral (low confidence)
AZOLI_p50315	Ancestral (high confidence)	AZOBR_p270289	Ancestral (low confidence)
AZOLI_p50316	Ancestral (medium confidence)	AZOBR_p270290	Ancestral (low confidence)
AZOLI_p50317	Ancestral (medium confidence)	AZOBR_p270291	Ancestral (low confidence)
AZOLI_p50318	Ancestral (medium confidence)	AZOBR_p270292	Horizontally Transferred (high confidence)
AZOLI_p50319	Horizontally Transferred (high confidence)	AZOBR_p270293	Ancestral (low confidence)
AZOLI_p50320	Horizontally Transferred (high confidence)	AZOBR_p270294	Horizontally Transferred (high confidence)
AZOLI_p50321	Ancestral (low confidence)	AZOBR_p270295	Ancestral (low confidence)
AZOLI_p50322	Horizontally Transferred (high confidence)	AZOBR_p270296	Horizontally Transferred (low confidence)
AZOLI_p50323	Ancestral (medium confidence)	AZOBR_p270297	Unassigned
AZOLI_p50325	Horizontally Transferred (high confidence)	AZOBR_p270298	Horizontally Transferred (high confidence)
AZOLI_p50326	Unassigned	AZOBR_p270299	Ancestral (low confidence)
AZOLI_p50328	Horizontally Transferred (low confidence)	AZOBR_p270300	Ancestral (low confidence)
AZOLI_p50329	Horizontally Transferred (high confidence)	AZOBR_p270301	Ancestral (low confidence)
AZOLI_p50330	Horizontally Transferred (high confidence)	AZOBR_p270302	Unassigned
AZOLI_p50331	Ancestral (low confidence)	AZOBR_p270303	Ancestral (low confidence)
AZOLI_p50332	Horizontally Transferred (high confidence)	AZOBR_p270304	Horizontally Transferred (high confidence)
AZOLI_p50333	Horizontally Transferred (high confidence)	AZOBR_p270305	Horizontally Transferred (high confidence)
AZOLI_p50334	Horizontally Transferred (high confidence)	AZOBR_p270306	Horizontally Transferred (medium confidence)
AZOLI_p50335	Horizontally Transferred (high confidence)	AZOBR_p270307	Horizontally Transferred (high confidence)
AZOLI_p50336	Horizontally Transferred (high confidence)	AZOBR_p270308	Horizontally Transferred (high confidence)
AZOLI_p50337	Horizontally Transferred (high confidence)	AZOBR_p270309	Horizontally Transferred (medium confidence)
AZOLI_p50338	Horizontally Transferred (high confidence)	AZOBR_p270310	Horizontally Transferred (high confidence)
AZOLI_p50339	Horizontally Transferred (high confidence)	AZOBR_p280001	Horizontally Transferred (high confidence)
AZOLI_p50340	Horizontally Transferred (high confidence)	AZOBR_p280002	Horizontally Transferred (high confidence)
AZOLI_p50341	Horizontally Transferred (low confidence)	AZOBR_p280003	Horizontally Transferred (high confidence)
AZOLI_p50342	Horizontally Transferred (medium confidence)	AZOBR_p280004	Horizontally Transferred (high confidence)
AZOLI_p50343	Horizontally Transferred (high confidence)	AZOBR_p280005	Horizontally Transferred (high confidence)
AZOLI_p50344	Horizontally Transferred (high confidence)	AZOBR_p280006	Ancestral (low confidence)
AZOLI_p50345	Ancestral (low confidence)	AZOBR_p280007	Horizontally Transferred (high confidence)
AZOLI_p50346	Horizontally Transferred (medium confidence)	AZOBR_p280008	Unassigned
AZOLI_p50347	Ancestral (low confidence)	AZOBR_p280009	Unassigned
AZOLI_p50348	Horizontally Transferred (medium confidence)	AZOBR_p280010	Horizontally Transferred (high confidence)
AZOLI_p50349	Unassigned	AZOBR_p280011	Unassigned
AZOLI_p50350	Unassigned	AZOBR_p280012	Unassigned
AZOLI_p50351	Unassigned	AZOBR_p280013	Horizontally Transferred (high confidence)
AZOLI_p50352	Ancestral (low confidence)	AZOBR_p280014	Unassigned
AZOLI_p50353	Unassigned	AZOBR_p280015	Horizontally Transferred (low confidence)
AZOLI_p50354	Horizontally Transferred (high confidence)	AZOBR_p280016	Horizontally Transferred (low confidence)
AZOLI_p50355	Horizontally Transferred (high confidence)	AZOBR_p280017	Horizontally Transferred (low confidence)

AZOLI_p50356	Ancestral (medium confidence)	AZOBR_p280018	Ancestral (low confidence)
AZOLI_p50357	Ancestral (medium confidence)	AZOBR_p280019	Ancestral (low confidence)
AZOLI_p50358	Ancestral (low confidence)	AZOBR_p280020	Ancestral (low confidence)
AZOLI_p50359	Horizontally Transferred (high confidence)	AZOBR_p280021	Ancestral (low confidence)
AZOLI_p50360	Unassigned	AZOBR_p280022	Horizontally Transferred (high confidence)
AZOLI_p50362	Ancestral (low confidence)	AZOBR_p280023	Horizontally Transferred (high confidence)
AZOLI_p50363	Unassigned	AZOBR_p280024	Horizontally Transferred (low confidence)
AZOLI_p50364	Ancestral (low confidence)	AZOBR_p280025	Unassigned
AZOLI_p50365	Ancestral (low confidence)	AZOBR_p280026	Ancestral (high confidence)
AZOLI_p50366	Horizontally Transferred (high confidence)	AZOBR_p280027	Ancestral (medium confidence)
AZOLI_p50367	Ancestral (low confidence)	AZOBR_p280028	Horizontally Transferred (high confidence)
AZOLI_p50368	Horizontally Transferred (high confidence)	AZOBR_p280029	Horizontally Transferred (high confidence)
AZOLI_p50369	Ancestral (low confidence)	AZOBR_p280030	Unassigned
AZOLI_p50370	Horizontally Transferred (high confidence)	AZOBR_p280031	Horizontally Transferred (high confidence)
AZOLI_p50371	Horizontally Transferred (high confidence)	AZOBR_p280032	Horizontally Transferred (high confidence)
AZOLI_p50372	Horizontally Transferred (high confidence)	AZOBR_p280033	Ancestral (low confidence)
AZOLI_p50373	Horizontally Transferred (high confidence)	AZOBR_p280034	Horizontally Transferred (high confidence)
AZOLI_p50374	Horizontally Transferred (high confidence)	AZOBR_p280035	Ancestral (low confidence)
AZOLI_p50375	Ancestral (high confidence)	AZOBR_p280036	Ancestral (low confidence)
AZOLI_p50376	Horizontally Transferred (high confidence)	AZOBR_p280037	Ancestral (medium confidence)
AZOLI_p50377	Horizontally Transferred (high confidence)	AZOBR_p280038	Ancestral (medium confidence)
AZOLI_p50378	Ancestral (low confidence)	AZOBR_p280039	Horizontally Transferred (low confidence)
AZOLI_p50379	Horizontally Transferred (high confidence)	AZOBR_p280040	Ancestral (low confidence)
AZOLI_p50380	Horizontally Transferred (high confidence)	AZOBR_p280041	Unassigned
AZOLI_p50381	Horizontally Transferred (high confidence)	AZOBR_p280042	Unassigned
AZOLI_p50382	Horizontally Transferred (high confidence)	AZOBR_p280043	Horizontally Transferred (high confidence)
AZOLI_p50383	Horizontally Transferred (high confidence)	AZOBR_p280044	Unassigned
AZOLI_p50384	Horizontally Transferred (high confidence)	AZOBR_p280045	Horizontally Transferred (high confidence)
AZOLI_p50385	Horizontally Transferred (high confidence)	AZOBR_p280046	Unassigned
AZOLI_p50387	Unassigned	AZOBR_p280047	Unassigned
AZOLI_p50388	Unassigned	AZOBR_p280048	Ancestral (medium confidence)
AZOLI_p50389	Ancestral (high confidence)	AZOBR_p280049	Ancestral (high confidence)
AZOLI_p50390	Unassigned	AZOBR_p280050	Unassigned
AZOLI_p50392	Ancestral (low confidence)	AZOBR_p280051	Ancestral (low confidence)
AZOLI_p50393	Ancestral (medium confidence)	AZOBR_p280052	Horizontally Transferred (high confidence)
AZOLI_p50394	Ancestral (medium confidence)	AZOBR_p280053	Ancestral (low confidence)
AZOLI_p50395	Ancestral (medium confidence)	AZOBR_p280054	Horizontally Transferred (high confidence)
AZOLI_p50396	Ancestral (medium confidence)	AZOBR_p280055	Horizontally Transferred (high confidence)
AZOLI_p50397	Ancestral (medium confidence)	AZOBR_p280056	Horizontally Transferred (medium confidence)
AZOLI_p50398	Horizontally Transferred (high confidence)	AZOBR_p280057	Ancestral (low confidence)
AZOLI_p50399	Unassigned	AZOBR_p280058	Ancestral (medium confidence)
AZOLI_p50400	Horizontally Transferred (high confidence)	AZOBR_p280059	Horizontally Transferred (high confidence)
AZOLI_p50401	Horizontally Transferred (high confidence)	AZOBR_p280060	Horizontally Transferred (high confidence)
AZOLI_p50402	Horizontally Transferred (high confidence)	AZOBR_p280061	Horizontally Transferred (medium confidence)
AZOLI_p50403	Horizontally Transferred (high confidence)	AZOBR_p280062	Ancestral (medium confidence)
AZOLI_p50404	Horizontally Transferred (medium confidence)	AZOBR_p280063	Unassigned
AZOLI_p50405	Horizontally Transferred (high confidence)	AZOBR_p280064	Ancestral (low confidence)
AZOLI_p50406	Ancestral (low confidence)	AZOBR_p280065	Unassigned
AZOLI_p50407	Ancestral (low confidence)	AZOBR_p280066	Horizontally Transferred (low confidence)
AZOLI_p50408	Horizontally Transferred (high confidence)	AZOBR_p280067	Ancestral (high confidence)
AZOLI_p50409	Ancestral (low confidence)	AZOBR_p280068	Ancestral (high confidence)
AZOLI_p50411	Unassigned	AZOBR_p280069	Ancestral (low confidence)
AZOLI_p50413	Horizontally Transferred (low confidence)	AZOBR_p280070	Unassigned
AZOLI_p50414	Horizontally Transferred (low confidence)	AZOBR_p280071	Ancestral (low confidence)
AZOLI_p50415	Horizontally Transferred (high confidence)	AZOBR_p280072	Unassigned
AZOLI_p50416	Horizontally Transferred (high confidence)	AZOBR_p280073	Ancestral (low confidence)
AZOLI_p50417	Unassigned	AZOBR_p280074	Ancestral (low confidence)
AZOLI_p50419	Ancestral (medium confidence)	AZOBR_p280075	Unassigned
AZOLI_p50420	Horizontally Transferred (high confidence)	AZOBR_p280076	Ancestral (low confidence)
AZOLI_p50421	Ancestral (low confidence)	AZOBR_p280077	Horizontally Transferred (high confidence)
AZOLI_p50422	Ancestral (low confidence)	AZOBR_p280078	Horizontally Transferred (high confidence)
AZOLI_p50423	Unassigned	AZOBR_p280079	Horizontally Transferred (high confidence)
AZOLI_p50424	Horizontally Transferred (medium confidence)	AZOBR_p280080	Horizontally Transferred (high confidence)
AZOLI_p50425	Horizontally Transferred (high confidence)	AZOBR_p280081	Horizontally Transferred (medium confidence)
AZOLI_p50426	Horizontally Transferred (high confidence)	AZOBR_p280082	Horizontally Transferred (high confidence)
AZOLI_p50427	Horizontally Transferred (high confidence)	AZOBR_p280083	Horizontally Transferred (high confidence)
AZOLI_p50428	Unassigned	AZOBR_p280084	Unassigned
AZOLI_p50429	Horizontally Transferred (high confidence)	AZOBR_p280085	Horizontally Transferred (medium confidence)
AZOLI_p50430	Horizontally Transferred (low confidence)	AZOBR_p280086	Unassigned
AZOLI_p50431	Unassigned	AZOBR_p280087	Horizontally Transferred (low confidence)
AZOLI_p50432	Ancestral (low confidence)	AZOBR_p280088	Horizontally Transferred (high confidence)
AZOLI_p50433	Ancestral (high confidence)	AZOBR_p280089	Horizontally Transferred (high confidence)
AZOLI_p50434	Horizontally Transferred (medium confidence)	AZOBR_p280090	Horizontally Transferred (high confidence)
AZOLI_p50435	Horizontally Transferred (high confidence)	AZOBR_p280091	Horizontally Transferred (high confidence)
AZOLI_p50436	Horizontally Transferred (high confidence)	AZOBR_p280092	Horizontally Transferred (high confidence)

AZOLI_p50437	Ancestral (low confidence)	AZOBR_p280093	Horizontally Transferred (high confidence)
AZOLI_p50438	Horizontally Transferred (high confidence)	AZOBR_p280094	Unassigned
AZOLI_p50442	Horizontally Transferred (high confidence)	AZOBR_p280095	Horizontally Transferred (low confidence)
AZOLI_p60001	Horizontally Transferred (high confidence)	AZOBR_p280096	Horizontally Transferred (medium confidence)
AZOLI_p60002	Horizontally Transferred (high confidence)	AZOBR_p280097	Horizontally Transferred (high confidence)
AZOLI_p60003	Horizontally Transferred (high confidence)	AZOBR_p280098	Ancestral (low confidence)
AZOLI_p60004	Horizontally Transferred (high confidence)	AZOBR_p280099	Ancestral (high confidence)
AZOLI_p60005	Ancestral (low confidence)	AZOBR_p280100	Horizontally Transferred (low confidence)
AZOLI_p60006	Unassigned	AZOBR_p280101	Horizontally Transferred (high confidence)
AZOLI_p60007	Ancestral (medium confidence)	AZOBR_p280102	Unassigned
AZOLI_p60008	Ancestral (low confidence)	AZOBR_p280103	Unassigned
AZOLI_p60009	Horizontally Transferred (high confidence)	AZOBR_p280104	Ancestral (medium confidence)
AZOLI_p60010	Unassigned	AZOBR_p280105	Ancestral (high confidence)
AZOLI_p60011	Ancestral (low confidence)	AZOBR_p280106	Horizontally Transferred (high confidence)
AZOLI_p60012	Ancestral (low confidence)	AZOBR_p280107	Ancestral (medium confidence)
AZOLI_p60013	Unassigned	AZOBR_p280108	Ancestral (medium confidence)
AZOLI_p60014	Ancestral (low confidence)	AZOBR_p280109	Ancestral (medium confidence)
AZOLI_p60015	Ancestral (low confidence)	AZOBR_p280110	Ancestral (medium confidence)
AZOLI_p60016	Ancestral (low confidence)	AZOBR_p280111	Ancestral (medium confidence)
AZOLI_p60017	Unassigned	AZOBR_p280112	Ancestral (low confidence)
AZOLI_p60019	Unassigned	AZOBR_p280113	Ancestral (medium confidence)
AZOLI_p60020	Unassigned	AZOBR_p280114	Horizontally Transferred (medium confidence)
AZOLI_p60022	Horizontally Transferred (high confidence)	AZOBR_p280115	Horizontally Transferred (high confidence)
AZOLI_p60023	Horizontally Transferred (high confidence)	AZOBR_p280116	Unassigned
AZOLI_p60024	Horizontally Transferred (high confidence)	AZOBR_p280117	Horizontally Transferred (high confidence)
AZOLI_p60025	Horizontally Transferred (high confidence)	AZOBR_p280118	Ancestral (low confidence)
AZOLI_p60026	Horizontally Transferred (high confidence)	AZOBR_p280119	Unassigned
AZOLI_p60027	Horizontally Transferred (high confidence)	AZOBR_p280120	Unassigned
AZOLI_p60028	Unassigned	AZOBR_p280121	Unassigned
AZOLI_p60029	Horizontally Transferred (high confidence)	AZOBR_p280122	Unassigned
AZOLI_p60030	Horizontally Transferred (high confidence)	AZOBR_p280123	Ancestral (medium confidence)
AZOLI_p60031	Unassigned	AZOBR_p280124	Ancestral (medium confidence)
AZOLI_p60032	Horizontally Transferred (high confidence)	AZOBR_p280125	Unassigned
AZOLI_p60033	Unassigned	AZOBR_p280126	Unassigned
AZOLI_p60034	Horizontally Transferred (high confidence)	AZOBR_p280127	Unassigned
AZOLI_p60035	Unassigned	AZOBR_p280128	Horizontally Transferred (medium confidence)
AZOLI_p60036	Ancestral (medium confidence)	AZOBR_p280129	Horizontally Transferred (high confidence)
AZOLI_p60037	Ancestral (low confidence)	AZOBR_p280130	Horizontally Transferred (high confidence)
AZOLI_p60038	Unassigned	AZOBR_p280131	Ancestral (medium confidence)
AZOLI_p60039	Horizontally Transferred (high confidence)	AZOBR_p280132	Ancestral (low confidence)
AZOLI_p60040	Horizontally Transferred (high confidence)	AZOBR_p280133	Ancestral (low confidence)
AZOLI_p60041	Unassigned	AZOBR_p280134	Ancestral (medium confidence)
AZOLI_p60042	Ancestral (low confidence)	AZOBR_p280135	Unassigned
AZOLI_p60043	Unassigned	AZOBR_p280136	Ancestral (medium confidence)
AZOLI_p60044	Horizontally Transferred (high confidence)	AZOBR_p280137	Ancestral (medium confidence)
AZOLI_p60045	Ancestral (low confidence)	AZOBR_p280138	Horizontally Transferred (high confidence)
AZOLI_p60046	Horizontally Transferred (medium confidence)	AZOBR_p280139	Unassigned
AZOLI_p60047	Ancestral (low confidence)	AZOBR_p280140	Ancestral (low confidence)
AZOLI_p60048	Ancestral (low confidence)	AZOBR_p280141	Horizontally Transferred (high confidence)
AZOLI_p60049	Ancestral (low confidence)	AZOBR_p280142	Horizontally Transferred (low confidence)
AZOLI_p60050	Ancestral (low confidence)	AZOBR_p280143	Horizontally Transferred (high confidence)
AZOLI_p60051	Horizontally Transferred (high confidence)	AZOBR_p280144	Ancestral (medium confidence)
AZOLI_p60052	Unassigned	AZOBR_p310001	Unassigned
AZOLI_p60053	Unassigned	AZOBR_p310002	Unassigned
AZOLI_p60054	Horizontally Transferred (high confidence)	AZOBR_p310003	Ancestral (medium confidence)
AZOLI_p60055	Horizontally Transferred (medium confidence)	AZOBR_p310004	Ancestral (medium confidence)
AZOLI_p60056	Unassigned	AZOBR_p310005	Horizontally Transferred (high confidence)
AZOLI_p60057	Horizontally Transferred (high confidence)	AZOBR_p310006	Ancestral (medium confidence)
AZOLI_p60058	Ancestral (low confidence)	AZOBR_p310007	Ancestral (low confidence)
AZOLI_p60059	Ancestral (low confidence)	AZOBR_p310008	Horizontally Transferred (high confidence)
AZOLI_p60060	Ancestral (low confidence)	AZOBR_p310009	Unassigned
AZOLI_p60061	Ancestral (low confidence)	AZOBR_p310010	Unassigned
AZOLI_p60062	Ancestral (medium confidence)	AZOBR_p310011	Horizontally Transferred (high confidence)
AZOLI_p60063	Ancestral (medium confidence)	AZOBR_p310012	Unassigned
AZOLI_p60064	Ancestral (medium confidence)	AZOBR_p310013	Horizontally Transferred (high confidence)
AZOLI_p60065	Horizontally Transferred (high confidence)	AZOBR_p310014	Horizontally Transferred (high confidence)
AZOLI_p60069	Ancestral (low confidence)	AZOBR_p310015	Horizontally Transferred (high confidence)
AZOLI_p60070	Horizontally Transferred (high confidence)	AZOBR_p310016	Unassigned
AZOLI_p60071	Horizontally Transferred (high confidence)	AZOBR_p310017	Horizontally Transferred (high confidence)
AZOLI_p60072	Ancestral (low confidence)	AZOBR_p310018	Horizontally Transferred (high confidence)
AZOLI_p60073	Ancestral (medium confidence)	AZOBR_p310019	Horizontally Transferred (high confidence)
AZOLI_p60074	Ancestral (medium confidence)	AZOBR_p310020	Unassigned
AZOLI_p60075	Ancestral (medium confidence)	AZOBR_p310021	Horizontally Transferred (high confidence)
AZOLI_p60076	Unassigned	AZOBR_p310022	Horizontally Transferred (high confidence)
AZOLI_p60077	Horizontally Transferred (high confidence)	AZOBR_p310023	Unassigned

AZOLI_p60078	Horizontally Transferred (high confidence)	AZOBR_p310024	Unassigned
AZOLI_p60079	Horizontally Transferred (medium confidence)	AZOBR_p310025	Ancestral (low confidence)
AZOLI_p60080	Horizontally Transferred (high confidence)	AZOBR_p310026	Unassigned
AZOLI_p60081	Horizontally Transferred (high confidence)	AZOBR_p310027	Unassigned
AZOLI_p60083	Unassigned	AZOBR_p310028	Unassigned
AZOLI_p60084	Unassigned	AZOBR_p310029	Horizontally Transferred (high confidence)
AZOLI_p60085	Ancestral (low confidence)	AZOBR_p310030	Ancestral (low confidence)
AZOLI_p60086	Unassigned	AZOBR_p310031	Unassigned
AZOLI_p60087	Horizontally Transferred (high confidence)	AZOBR_p310032	Ancestral (low confidence)
AZOLI_p60088	Ancestral (low confidence)	AZOBR_p310033	Unassigned
AZOLI_p60089	Horizontally Transferred (high confidence)	AZOBR_p310034	Ancestral (low confidence)
AZOLI_p60091	Unassigned	AZOBR_p310035	Ancestral (low confidence)
AZOLI_p60092	Ancestral (low confidence)	AZOBR_p310036	Horizontally Transferred (high confidence)
AZOLI_p60093	Horizontally Transferred (high confidence)	AZOBR_p310037	Horizontally Transferred (high confidence)
AZOLI_p60094	Ancestral (low confidence)	AZOBR_p310038	Horizontally Transferred (high confidence)
AZOLI_p60095	Horizontally Transferred (high confidence)	AZOBR_p310039	Horizontally Transferred (high confidence)
AZOLI_p60096	Ancestral (low confidence)	AZOBR_p310040	Horizontally Transferred (high confidence)
AZOLI_p60097	Ancestral (low confidence)	AZOBR_p310041	Horizontally Transferred (high confidence)
AZOLI_p60098	Unassigned	AZOBR_p310042	Horizontally Transferred (high confidence)
AZOLI_p60099	Unassigned	AZOBR_p310043	Unassigned
AZOLI_p60100	Ancestral (low confidence)	AZOBR_p310044	Horizontally Transferred (medium confidence)
AZOLI_p60101	Horizontally Transferred (high confidence)	AZOBR_p310045	Horizontally Transferred (high confidence)
AZOLI_p60102	Horizontally Transferred (low confidence)	AZOBR_p310046	Horizontally Transferred (high confidence)
AZOLI_p60103	Horizontally Transferred (high confidence)	AZOBR_p310047	Horizontally Transferred (high confidence)
AZOLI_p60104	Horizontally Transferred (high confidence)	AZOBR_p310048	Unassigned
AZOLI_p60105	Horizontally Transferred (high confidence)	AZOBR_p310049	Horizontally Transferred (high confidence)
AZOLI_p60106	Horizontally Transferred (high confidence)	AZOBR_p310050	Unassigned
AZOLI_p60107	Ancestral (low confidence)	AZOBR_p310051	Horizontally Transferred (high confidence)
AZOLI_p60108	Horizontally Transferred (medium confidence)	AZOBR_p310052	Unassigned
AZOLI_p60109	Horizontally Transferred (high confidence)	AZOBR_p310053	Horizontally Transferred (high confidence)
AZOLI_p60110	Horizontally Transferred (high confidence)	AZOBR_p310054	Horizontally Transferred (high confidence)
AZOLI_p60111	Horizontally Transferred (high confidence)	AZOBR_p310055	Horizontally Transferred (high confidence)
AZOLI_p60112	Ancestral (low confidence)	AZOBR_p310056	Horizontally Transferred (high confidence)
AZOLI_p60113	Horizontally Transferred (high confidence)	AZOBR_p310057	Horizontally Transferred (high confidence)
AZOLI_p60115	Horizontally Transferred (high confidence)	AZOBR_p310058	Horizontally Transferred (high confidence)
AZOLI_p60116	Horizontally Transferred (high confidence)	AZOBR_p310059	Horizontally Transferred (high confidence)
AZOLI_p60117	Horizontally Transferred (high confidence)	AZOBR_p310060	Unassigned
AZOLI_p60118	Horizontally Transferred (high confidence)	AZOBR_p310061	Ancestral (medium confidence)
AZOLI_p60119	Horizontally Transferred (high confidence)	AZOBR_p310062	Unassigned
AZOLI_p60120	Horizontally Transferred (medium confidence)	AZOBR_p310063	Horizontally Transferred (medium confidence)
AZOLI_p60121	Unassigned	AZOBR_p310064	Horizontally Transferred (high confidence)
AZOLI_p60122	Unassigned	AZOBR_p310065	Horizontally Transferred (high confidence)
AZOLI_p60123	Ancestral (low confidence)	AZOBR_p310066	Horizontally Transferred (high confidence)
AZOLI_p60124	Ancestral (low confidence)	AZOBR_p310067	Ancestral (low confidence)
AZOLI_p60126	Ancestral (low confidence)	AZOBR_p310068	Horizontally Transferred (high confidence)
AZOLI_p60128	Ancestral (low confidence)	AZOBR_p310069	Horizontally Transferred (low confidence)
AZOLI_p60129	Horizontally Transferred (high confidence)	AZOBR_p310070	Horizontally Transferred (high confidence)
AZOLI_p60130	Horizontally Transferred (high confidence)	AZOBR_p310071	Unassigned
AZOLI_p60131	Ancestral (low confidence)	AZOBR_p310072	Horizontally Transferred (high confidence)
AZOLI_p60132	Ancestral (medium confidence)	AZOBR_p310073	Horizontally Transferred (high confidence)
AZOLI_p60133	Unassigned	AZOBR_p310074	Ancestral (low confidence)
AZOLI_p60134	Ancestral (low confidence)	AZOBR_p310075	Horizontally Transferred (high confidence)
AZOLI_p60135	Ancestral (low confidence)	AZOBR_p310076	Horizontally Transferred (high confidence)
AZOLI_p60136	Horizontally Transferred (low confidence)	AZOBR_p310077	Horizontally Transferred (high confidence)
AZOLI_p60137	Ancestral (low confidence)	AZOBR_p310078	Horizontally Transferred (high confidence)
AZOLI_p60138	Horizontally Transferred (high confidence)	AZOBR_p310079	Horizontally Transferred (high confidence)
AZOLI_p60139	Horizontally Transferred (high confidence)	AZOBR_p310080	Horizontally Transferred (high confidence)
AZOLI_p60140	Ancestral (low confidence)	AZOBR_p310081	Unassigned
AZOLI_p60141	Horizontally Transferred (medium confidence)	AZOBR_p310082	Horizontally Transferred (high confidence)
AZOLI_p60142	Ancestral (low confidence)	AZOBR_p310083	Horizontally Transferred (high confidence)
AZOLI_p60143	Ancestral (low confidence)	AZOBR_p310084	Horizontally Transferred (high confidence)
AZOLI_p60144	Ancestral (low confidence)	AZOBR_p310085	Horizontally Transferred (high confidence)
AZOLI_p60146	Unassigned	AZOBR_p310086	Horizontally Transferred (medium confidence)
AZOLI_p60149	Unassigned	AZOBR_p310087	Unassigned
AZOLI_p60150	Horizontally Transferred (high confidence)	AZOBR_p310088	Ancestral (low confidence)
AZOLI_p60151	Unassigned	AZOBR_p310089	Horizontally Transferred (high confidence)
AZOLI_p60152	Horizontally Transferred (medium confidence)	AZOBR_p310090	Ancestral (low confidence)
AZOLI_p60153	Horizontally Transferred (low confidence)	AZOBR_p310091	Horizontally Transferred (high confidence)
AZOLI_p60154	Horizontally Transferred (high confidence)	AZOBR_p310092	Horizontally Transferred (medium confidence)
AZOLI_p60155	Horizontally Transferred (medium confidence)	AZOBR_p310093	Horizontally Transferred (high confidence)
AZOLI_p60158	Horizontally Transferred (high confidence)	AZOBR_p310094	Unassigned
AZOLI_p60159	Ancestral (medium confidence)	AZOBR_p310095	Horizontally Transferred (high confidence)
AZOLI_p60160	Unassigned	AZOBR_p310096	Horizontally Transferred (high confidence)
AZOLI_p60161	Unassigned	AZOBR_p310097	Unassigned
AZOLI_p60162	Horizontally Transferred (medium confidence)	AZOBR_p310098	Unassigned

AZOLI_p60164	Ancestral (medium confidence)	AZOBR_p310099	Horizontally Transferred (high confidence)
AZOLI_p60165	Horizontally Transferred (high confidence)	AZOBR_p310100	Horizontally Transferred (high confidence)
AZOLI_p60166	Horizontally Transferred (medium confidence)	AZOBR_p310101	Ancestral (low confidence)
AZOLI_p60167	Horizontally Transferred (high confidence)	AZOBR_p310102	Horizontally Transferred (high confidence)
AZOLI_p60168	Ancestral (medium confidence)	AZOBR_p310103	Horizontally Transferred (high confidence)
AZOLI_p60169	Unassigned	AZOBR_p310104	Horizontally Transferred (high confidence)
AZOLI_p60171	Horizontally Transferred (low confidence)	AZOBR_p310105	Horizontally Transferred (medium confidence)
AZOLI_p60172	Unassigned	AZOBR_p310106	Horizontally Transferred (high confidence)
AZOLI_p60173	Unassigned	AZOBR_p310107	Horizontally Transferred (high confidence)
AZOLI_p60174	Ancestral (medium confidence)	AZOBR_p310108	Horizontally Transferred (high confidence)
AZOLI_p60175	Ancestral (low confidence)	AZOBR_p310109	Horizontally Transferred (high confidence)
AZOLI_p60176	Horizontally Transferred (high confidence)	AZOBR_p310110	Horizontally Transferred (high confidence)
AZOLI_p60177	Ancestral (low confidence)	AZOBR_p310111	Horizontally Transferred (high confidence)
AZOLI_p60178	Ancestral (medium confidence)	AZOBR_p310112	Horizontally Transferred (high confidence)
AZOLI_p60180	Horizontally Transferred (high confidence)	AZOBR_p310113	Horizontally Transferred (high confidence)
AZOLI_p60182	Ancestral (medium confidence)	AZOBR_p310114	Ancestral (low confidence)
AZOLI_p60183	Ancestral (low confidence)	AZOBR_p310115	Horizontally Transferred (high confidence)
AZOLI_p60184	Ancestral (low confidence)	AZOBR_p310116	Horizontally Transferred (high confidence)
AZOLI_p60185	Ancestral (medium confidence)	AZOBR_p310117	Horizontally Transferred (high confidence)
AZOLI_p60186	Ancestral (low confidence)	AZOBR_p310118	Horizontally Transferred (high confidence)
AZOLI_p60187	Ancestral (low confidence)	AZOBR_p310119	Horizontally Transferred (high confidence)
AZOLI_p60188	Horizontally Transferred (high confidence)	AZOBR_p310120	Horizontally Transferred (high confidence)
AZOLI_p60189	Ancestral (low confidence)	AZOBR_p310121	Horizontally Transferred (medium confidence)
AZOLI_p60190	Ancestral (low confidence)	AZOBR_p310122	Unassigned
AZOLI_p60192	Unassigned	AZOBR_p310123	Horizontally Transferred (high confidence)
AZOLI_p60193	Ancestral (high confidence)	AZOBR_p310124	Horizontally Transferred (high confidence)
AZOLI_p60194	Horizontally Transferred (high confidence)	AZOBR_p310125	Horizontally Transferred (high confidence)
AZOLI_p60195	Horizontally Transferred (high confidence)	AZOBR_p310126	Ancestral (low confidence)
AZOLI_p60196	Ancestral (low confidence)	AZOBR_p310127	Horizontally Transferred (high confidence)
AZOLI_p60197	Horizontally Transferred (high confidence)	AZOBR_p310128	Unassigned
AZOLI_p60198	Ancestral (medium confidence)	AZOBR_p310129	Horizontally Transferred (medium confidence)
AZOLI_p60199	Ancestral (low confidence)	AZOBR_p310130	Horizontally Transferred (low confidence)
AZOLI_p60200	Ancestral (low confidence)	AZOBR_p310131	Horizontally Transferred (high confidence)
AZOLI_p60201	Horizontally Transferred (high confidence)	AZOBR_p310132	Unassigned
AZOLI_p60202	Unassigned	AZOBR_p310133	Ancestral (medium confidence)
AZOLI_p60203	Horizontally Transferred (high confidence)	AZOBR_p310134	Ancestral (medium confidence)
AZOLI_p60204	Unassigned	AZOBR_p310135	Unassigned
AZOLI_p60205	Horizontally Transferred (high confidence)	AZOBR_p310136	Ancestral (low confidence)
AZOLI_p60206	Horizontally Transferred (high confidence)	AZOBR_p310137	Horizontally Transferred (high confidence)
AZOLI_p60207	Ancestral (medium confidence)	AZOBR_p310138	Horizontally Transferred (low confidence)
AZOLI_p60210	Ancestral (medium confidence)	AZOBR_p310139	Horizontally Transferred (high confidence)
AZOLI_p60211	Horizontally Transferred (high confidence)	AZOBR_p310140	Horizontally Transferred (high confidence)
AZOLI_p60212	Ancestral (low confidence)	AZOBR_p310141	Ancestral (low confidence)
AZOLI_p60213	Unassigned	AZOBR_p310142	Unassigned
AZOLI_p60214	Ancestral (medium confidence)	AZOBR_p310143	Unassigned
AZOLI_p60215	Ancestral (low confidence)	AZOBR_p310144	Horizontally Transferred (low confidence)
AZOLI_p60216	Ancestral (low confidence)	AZOBR_p310145	Ancestral (low confidence)
AZOLI_p60217	Unassigned	AZOBR_p310146	Unassigned
AZOLI_p60218	Ancestral (medium confidence)	AZOBR_p310147	Horizontally Transferred (high confidence)
AZOLI_p60219	Horizontally Transferred (high confidence)	AZOBR_p310148	Ancestral (low confidence)
AZOLI_p60220	Ancestral (medium confidence)	AZOBR_p310149	Horizontally Transferred (medium confidence)
AZOLI_p60221	Horizontally Transferred (high confidence)	AZOBR_p310150	Horizontally Transferred (high confidence)
AZOLI_p60222	Ancestral (low confidence)	AZOBR_p310151	Unassigned
AZOLI_p60223	Horizontally Transferred (high confidence)	AZOBR_p310152	Unassigned
AZOLI_p60224	Horizontally Transferred (high confidence)	AZOBR_p310153	Horizontally Transferred (high confidence)
AZOLI_p60225	Horizontally Transferred (high confidence)	AZOBR_p310154	Horizontally Transferred (high confidence)
AZOLI_p60226	Unassigned	AZOBR_p310155	Unassigned
AZOLI_p60227	Horizontally Transferred (medium confidence)	AZOBR_p310156	Horizontally Transferred (high confidence)
AZOLI_p60228	Unassigned	AZOBR_p310157	Ancestral (low confidence)
AZOLI_p60229	Ancestral (low confidence)	AZOBR_p310158	Horizontally Transferred (medium confidence)
AZOLI_p60230	Ancestral (medium confidence)	AZOBR_p310159	Horizontally Transferred (high confidence)
AZOLI_p60231	Unassigned	AZOBR_p310160	Unassigned
AZOLI_p60232	Horizontally Transferred (high confidence)	AZOBR_p310161	Ancestral (low confidence)
AZOLI_p60233	Unassigned	AZOBR_p310162	Horizontally Transferred (high confidence)
AZOLI_p60234	Unassigned	AZOBR_p310163	Unassigned
AZOLI_p60235	Ancestral (high confidence)	AZOBR_p310164	Unassigned
AZOLI_p60236	Horizontally Transferred (high confidence)	AZOBR_p310165	Ancestral (high confidence)
AZOLI_p60237	Horizontally Transferred (high confidence)	AZOBR_p310166	Unassigned
AZOLI_p60238	Horizontally Transferred (high confidence)	AZOBR_p310167	Horizontally Transferred (high confidence)
AZOLI_p60239	Horizontally Transferred (medium confidence)	AZOBR_p310168	Ancestral (low confidence)
AZOLI_p60240	Horizontally Transferred (high confidence)	AZOBR_p310169	Ancestral (medium confidence)
AZOLI_p60241	Horizontally Transferred (high confidence)	AZOBR_p310170	Unassigned
AZOLI_p60242	Ancestral (low confidence)	AZOBR_p310171	Horizontally Transferred (high confidence)
AZOLI_p60243	Unassigned	AZOBR_p310172	Ancestral (low confidence)
AZOLI_p60244	Ancestral (low confidence)	AZOBR_p310173	Unassigned

AZOLI_p60245	Horizontally Transferred (low confidence)	AZOBR_p310174	Ancestral (medium confidence)
AZOLI_p60246	Ancestral (low confidence)	AZOBR_p310175	Ancestral (medium confidence)
AZOLI_p60251	Ancestral (low confidence)	AZOBR_p310176	Horizontally Transferred (medium confidence)
AZOLI_p60252	Ancestral (low confidence)	AZOBR_p310177	Ancestral (low confidence)
AZOLI_p60253	Ancestral (low confidence)	AZOBR_p310178	Unassigned
AZOLI_p60254	Unassigned	AZOBR_p310179	Horizontally Transferred (medium confidence)
AZOLI_p60256	Ancestral (low confidence)	AZOBR_p310180	Horizontally Transferred (medium confidence)
AZOLI_p60259	Horizontally Transferred (high confidence)	AZOBR_p310181	Unassigned
AZOLI_p60260	Ancestral (low confidence)	AZOBR_p310182	Horizontally Transferred (high confidence)
AZOLI_p60261	Horizontally Transferred (high confidence)	AZOBR_p310183	Unassigned
AZOLI_p60262	Unassigned	AZOBR_p310184	Horizontally Transferred (medium confidence)
AZOLI_p60263	Horizontally Transferred (high confidence)	AZOBR_p310185	Ancestral (low confidence)
AZOLI_p60264	Horizontally Transferred (high confidence)	AZOBR_p310186	Ancestral (low confidence)
AZOLI_p60265	Horizontally Transferred (high confidence)	AZOBR_p310187	Ancestral (low confidence)
AZOLI_p60266	Ancestral (low confidence)	AZOBR_p310188	Ancestral (low confidence)
		AZOBR_p310189	Unassigned
		AZOBR_p310190	Ancestral (low confidence)
		AZOBR_p310191	Horizontally Transferred (medium confidence)
		AZOBR_p310192	Horizontally Transferred (medium confidence)
		AZOBR_p310193	Horizontally Transferred (high confidence)
		AZOBR_p310194	Ancestral (low confidence)
		AZOBR_p310195	Ancestral (low confidence)
		AZOBR_p310196	Unassigned
		AZOBR_p310197	Horizontally Transferred (high confidence)
		AZOBR_p310198	Horizontally Transferred (high confidence)
		AZOBR_p310199	Horizontally Transferred (high confidence)
		AZOBR_p310200	Horizontally Transferred (high confidence)
		AZOBR_p310201	Horizontally Transferred (high confidence)
		AZOBR_p310202	Horizontally Transferred (high confidence)
		AZOBR_p310203	Ancestral (high confidence)
		AZOBR_p310204	Ancestral (low confidence)
		AZOBR_p310205	Ancestral (medium confidence)
		AZOBR_p310206	Ancestral (medium confidence)
		AZOBR_p310207	Ancestral (low confidence)
		AZOBR_p310208	Ancestral (low confidence)
		AZOBR_p310209	Horizontally Transferred (high confidence)
		AZOBR_p310210	Horizontally Transferred (high confidence)
		AZOBR_p310211	Horizontally Transferred (high confidence)
		AZOBR_p310212	Unassigned
		AZOBR_p310213	Ancestral (low confidence)
		AZOBR_p310214	Ancestral (medium confidence)
		AZOBR_p310215	Horizontally Transferred (high confidence)
		AZOBR_p310216	Unassigned
		AZOBR_p310217	Horizontally Transferred (high confidence)
		AZOBR_p310218	Horizontally Transferred (high confidence)
		AZOBR_p310219	Ancestral (low confidence)
		AZOBR_p310220	Horizontally Transferred (high confidence)
		AZOBR_p310221	Ancestral (low confidence)
		AZOBR_p310222	Horizontally Transferred (high confidence)
		AZOBR_p310223	Horizontally Transferred (high confidence)
		AZOBR_p310224	Ancestral (low confidence)
		AZOBR_p310225	Unassigned
		AZOBR_p310226	Horizontally Transferred (high confidence)
		AZOBR_p310227	Horizontally Transferred (medium confidence)
		AZOBR_p310228	Horizontally Transferred (high confidence)
		AZOBR_p310229	Horizontally Transferred (high confidence)
		AZOBR_p310230	Horizontally Transferred (high confidence)
		AZOBR_p310231	Horizontally Transferred (high confidence)
		AZOBR_p310232	Horizontally Transferred (high confidence)
		AZOBR_p310233	Horizontally Transferred (high confidence)
		AZOBR_p310234	Horizontally Transferred (high confidence)
		AZOBR_p310235	Horizontally Transferred (high confidence)
		AZOBR_p310236	Horizontally Transferred (high confidence)
		AZOBR_p310237	Horizontally Transferred (high confidence)
		AZOBR_p310238	Horizontally Transferred (medium confidence)
		AZOBR_p310239	Horizontally Transferred (high confidence)
		AZOBR_p310240	Horizontally Transferred (medium confidence)
		AZOBR_p310241	Unassigned
		AZOBR_p310242	Ancestral (low confidence)
		AZOBR_p310243	Unassigned
		AZOBR_p310244	Ancestral (low confidence)
		AZOBR_p310245	Ancestral (low confidence)
		AZOBR_p310246	Unassigned
		AZOBR_p310247	Horizontally Transferred (high confidence)
		AZOBR_p310248	Horizontally Transferred (high confidence)

		AZOBR_p310249	Horizontally Transferred (high confidence)
		AZOBR_p310250	Horizontally Transferred (high confidence)
		AZOBR_p310251	Horizontally Transferred (high confidence)
		AZOBR_p310252	Horizontally Transferred (high confidence)
		AZOBR_p310253	Horizontally Transferred (high confidence)
		AZOBR_p310254	Horizontally Transferred (high confidence)
		AZOBR_p310255	Ancestral (low confidence)
		AZOBR_p310256	Ancestral (low confidence)
		AZOBR_p310257	Horizontally Transferred (high confidence)
		AZOBR_p310258	Ancestral (high confidence)
		AZOBR_p310259	Ancestral (low confidence)
		AZOBR_p310260	Ancestral (low confidence)
		AZOBR_p310261	Unassigned
		AZOBR_p310262	Unassigned
		AZOBR_p310263	Unassigned
		AZOBR_p310264	Unassigned
		AZOBR_p310265	Horizontally Transferred (medium confidence)
		AZOBR_p310266	Horizontally Transferred (medium confidence)
		AZOBR_p310267	Horizontally Transferred (medium confidence)
		AZOBR_p310268	Ancestral (low confidence)
		AZOBR_p310269	Horizontally Transferred (high confidence)
		AZOBR_p310270	Horizontally Transferred (high confidence)
		AZOBR_p310271	Unassigned
		AZOBR_p310272	Horizontally Transferred (high confidence)
		AZOBR_p310273	Horizontally Transferred (high confidence)
		AZOBR_p310274	Ancestral (low confidence)
		AZOBR_p310275	Ancestral (low confidence)
		AZOBR_p310276	Ancestral (low confidence)
		AZOBR_p310277	Ancestral (low confidence)
		AZOBR_p310278	Ancestral (low confidence)
		AZOBR_p310279	Ancestral (low confidence)
		AZOBR_p310280	Ancestral (low confidence)
		AZOBR_p310281	Ancestral (low confidence)
		AZOBR_p310282	Horizontally Transferred (high confidence)
		AZOBR_p310283	Unassigned
		AZOBR_p310284	Ancestral (low confidence)
		AZOBR_p310285	Ancestral (low confidence)
		AZOBR_p310286	Horizontally Transferred (high confidence)
		AZOBR_p310287	Horizontally Transferred (high confidence)
		AZOBR_p310288	Unassigned
		AZOBR_p310289	Ancestral (low confidence)
		AZOBR_p310290	Horizontally Transferred (high confidence)
		AZOBR_p310291	Horizontally Transferred (high confidence)
		AZOBR_p310292	Horizontally Transferred (high confidence)
		AZOBR_p310293	Horizontally Transferred (high confidence)
		AZOBR_p310294	Unassigned
		AZOBR_p310295	Horizontally Transferred (medium confidence)
		AZOBR_p310296	Horizontally Transferred (high confidence)
		AZOBR_p310297	Horizontally Transferred (high confidence)
		AZOBR_p310298	Horizontally Transferred (high confidence)
		AZOBR_p310299	Horizontally Transferred (high confidence)
		AZOBR_p310300	Unassigned
		AZOBR_p310301	Unassigned
		AZOBR_p310302	Unassigned
		AZOBR_p310303	Horizontally Transferred (high confidence)
		AZOBR_p310304	Unassigned
		AZOBR_p310305	Unassigned
		AZOBR_p310306	Horizontally Transferred (high confidence)
		AZOBR_p310307	Horizontally Transferred (high confidence)
		AZOBR_p310308	Horizontally Transferred (high confidence)
		AZOBR_p310309	Horizontally Transferred (high confidence)
		AZOBR_p310310	Unassigned
		AZOBR_p310311	Unassigned
		AZOBR_p310312	Ancestral (low confidence)
		AZOBR_p310313	Horizontally Transferred (low confidence)
		AZOBR_p310314	Ancestral (low confidence)
		AZOBR_p310315	Horizontally Transferred (low confidence)
		AZOBR_p310316	Horizontally Transferred (low confidence)
		AZOBR_p310317	Horizontally Transferred (medium confidence)
		AZOBR_p310318	Horizontally Transferred (high confidence)
		AZOBR_p310319	Unassigned
		AZOBR_p310320	Horizontally Transferred (high confidence)
		AZOBR_p310321	Ancestral (low confidence)
		AZOBR_p310322	Horizontally Transferred (high confidence)
		AZOBR_p310323	Horizontally Transferred (low confidence)

		AZOBR_p310324	Horizontally Transferred (medium confidence)
		AZOBR_p310325	Horizontally Transferred (medium confidence)
		AZOBR_p310326	Unassigned
		AZOBR_p310327	Horizontally Transferred (high confidence)
		AZOBR_p310328	Horizontally Transferred (high confidence)
		AZOBR_p310329	Horizontally Transferred (high confidence)
		AZOBR_p310330	Unassigned
		AZOBR_p310331	Unassigned
		AZOBR_p310332	Unassigned
		AZOBR_p310333	Horizontally Transferred (high confidence)
		AZOBR_p310334	Horizontally Transferred (high confidence)
		AZOBR_p310335	Horizontally Transferred (high confidence)
		AZOBR_p310336	Unassigned
		AZOBR_p310337	Unassigned
		AZOBR_p310338	Horizontally Transferred (high confidence)
		AZOBR_p320001	Horizontally Transferred (high confidence)
		AZOBR_p320002	Unassigned
		AZOBR_p320003	Unassigned
		AZOBR_p330001	Unassigned
		AZOBR_p330002	Horizontally Transferred (high confidence)
		AZOBR_p330003	Ancestral (low confidence)
		AZOBR_p330004	Horizontally Transferred (medium confidence)
		AZOBR_p330005	Horizontally Transferred (high confidence)
		AZOBR_p330006	Unassigned
		AZOBR_p330007	Ancestral (low confidence)
		AZOBR_p330008	Unassigned
		AZOBR_p330009	Unassigned
		AZOBR_p330010	Horizontally Transferred (high confidence)
		AZOBR_p330011	Horizontally Transferred (high confidence)
		AZOBR_p330012	Ancestral (low confidence)
		AZOBR_p330013	Horizontally Transferred (low confidence)
		AZOBR_p330014	Horizontally Transferred (medium confidence)
		AZOBR_p330015	Horizontally Transferred (high confidence)
		AZOBR_p330016	Horizontally Transferred (high confidence)
		AZOBR_p330017	Horizontally Transferred (high confidence)
		AZOBR_p330018	Horizontally Transferred (high confidence)
		AZOBR_p330019	Horizontally Transferred (high confidence)
		AZOBR_p330020	Horizontally Transferred (low confidence)
		AZOBR_p330021	Horizontally Transferred (high confidence)
		AZOBR_p330022	Horizontally Transferred (high confidence)
		AZOBR_p330023	Ancestral (low confidence)
		AZOBR_p330024	Ancestral (low confidence)
		AZOBR_p330025	Ancestral (low confidence)
		AZOBR_p330026	Horizontally Transferred (high confidence)
		AZOBR_p330027	Horizontally Transferred (high confidence)
		AZOBR_p330028	Horizontally Transferred (high confidence)
		AZOBR_p330029	Ancestral (low confidence)
		AZOBR_p330030	Ancestral (low confidence)
		AZOBR_p330031	Ancestral (medium confidence)
		AZOBR_p330032	Ancestral (medium confidence)
		AZOBR_p330033	Unassigned
		AZOBR_p330034	Unassigned
		AZOBR_p330035	Unassigned
		AZOBR_p330036	Horizontally Transferred (high confidence)
		AZOBR_p330037	Unassigned
		AZOBR_p330038	Unassigned
		AZOBR_p330039	Horizontally Transferred (high confidence)
		AZOBR_p330040	Horizontally Transferred (high confidence)
		AZOBR_p330041	Unassigned
		AZOBR_p330042	Ancestral (medium confidence)
		AZOBR_p330043	Ancestral (high confidence)
		AZOBR_p330044	Horizontally Transferred (high confidence)
		AZOBR_p330045	Horizontally Transferred (high confidence)
		AZOBR_p330046	Horizontally Transferred (high confidence)
		AZOBR_p330047	Ancestral (medium confidence)
		AZOBR_p330048	Horizontally Transferred (high confidence)
		AZOBR_p330049	Horizontally Transferred (high confidence)
		AZOBR_p330050	Horizontally Transferred (high confidence)
		AZOBR_p330051	Horizontally Transferred (high confidence)
		AZOBR_p330052	Horizontally Transferred (high confidence)
		AZOBR_p330053	Horizontally Transferred (high confidence)
		AZOBR_p330054	Horizontally Transferred (high confidence)
		AZOBR_p330055	Horizontally Transferred (high confidence)
		AZOBR_p330056	Ancestral (low confidence)
		AZOBR_p330057	Horizontally Transferred (high confidence)

		AZOBR_p330058	Horizontally Transferred (high confidence)
		AZOBR_p330059	Horizontally Transferred (medium confidence)
		AZOBR_p330060	Horizontally Transferred (high confidence)
		AZOBR_p330061	Ancestral (low confidence)
		AZOBR_p330062	Ancestral (medium confidence)
		AZOBR_p330063	Unassigned
		AZOBR_p330064	Horizontally Transferred (high confidence)
		AZOBR_p330065	Horizontally Transferred (high confidence)
		AZOBR_p330066	Horizontally Transferred (high confidence)
		AZOBR_p330067	Horizontally Transferred (high confidence)
		AZOBR_p330068	Horizontally Transferred (high confidence)
		AZOBR_p330069	Horizontally Transferred (high confidence)
		AZOBR_p330070	Horizontally Transferred (medium confidence)
		AZOBR_p330071	Horizontally Transferred (low confidence)
		AZOBR_p330072	Horizontally Transferred (low confidence)
		AZOBR_p330073	Unassigned
		AZOBR_p330074	Horizontally Transferred (high confidence)
		AZOBR_p330075	Horizontally Transferred (high confidence)
		AZOBR_p330076	Horizontally Transferred (high confidence)
		AZOBR_p330077	Horizontally Transferred (high confidence)
		AZOBR_p330078	Horizontally Transferred (high confidence)
		AZOBR_p330079	Unassigned
		AZOBR_p330080	Horizontally Transferred (high confidence)
		AZOBR_p330081	Horizontally Transferred (high confidence)
		AZOBR_p330082	Horizontally Transferred (high confidence)
		AZOBR_p330083	Horizontally Transferred (high confidence)
		AZOBR_p330084	Horizontally Transferred (high confidence)
		AZOBR_p330085	Horizontally Transferred (high confidence)
		AZOBR_p330086	Horizontally Transferred (high confidence)
		AZOBR_p330087	Horizontally Transferred (high confidence)
		AZOBR_p330088	Horizontally Transferred (high confidence)
		AZOBR_p330089	Horizontally Transferred (high confidence)
		AZOBR_p330090	Horizontally Transferred (high confidence)
		AZOBR_p330091	Horizontally Transferred (high confidence)
		AZOBR_p330092	Horizontally Transferred (high confidence)
		AZOBR_p330093	Horizontally Transferred (low confidence)
		AZOBR_p330094	Ancestral (low confidence)
		AZOBR_p330095	Horizontally Transferred (high confidence)
		AZOBR_p330096	Ancestral (low confidence)
		AZOBR_p330097	Ancestral (low confidence)
		AZOBR_p330098	Ancestral (medium confidence)
		AZOBR_p330099	Horizontally Transferred (high confidence)
		AZOBR_p330100	Horizontally Transferred (high confidence)
		AZOBR_p330101	Horizontally Transferred (high confidence)
		AZOBR_p330102	Horizontally Transferred (high confidence)
		AZOBR_p330103	Horizontally Transferred (high confidence)
		AZOBR_p330104	Unassigned
		AZOBR_p330105	Unassigned
		AZOBR_p330106	Unassigned
		AZOBR_p330107	Ancestral (medium confidence)
		AZOBR_p330108	Horizontally Transferred (medium confidence)
		AZOBR_p330109	Horizontally Transferred (high confidence)
		AZOBR_p330110	Horizontally Transferred (high confidence)
		AZOBR_p330111	Horizontally Transferred (medium confidence)
		AZOBR_p330112	Horizontally Transferred (high confidence)
		AZOBR_p330113	Unassigned
		AZOBR_p330114	Ancestral (low confidence)
		AZOBR_p330115	Ancestral (low confidence)
		AZOBR_p330116	Ancestral (low confidence)
		AZOBR_p330117	Ancestral (low confidence)
		AZOBR_p330118	Ancestral (low confidence)
		AZOBR_p330119	Ancestral (low confidence)
		AZOBR_p330120	Ancestral (low confidence)
		AZOBR_p330121	Ancestral (low confidence)
		AZOBR_p330122	Horizontally Transferred (high confidence)
		AZOBR_p330123	Horizontally Transferred (high confidence)
		AZOBR_p330124	Ancestral (medium confidence)
		AZOBR_p330125	Horizontally Transferred (medium confidence)
		AZOBR_p330126	Horizontally Transferred (high confidence)
		AZOBR_p330127	Horizontally Transferred (high confidence)
		AZOBR_p330128	Horizontally Transferred (high confidence)
		AZOBR_p330129	Horizontally Transferred (high confidence)
		AZOBR_p330130	Horizontally Transferred (high confidence)
		AZOBR_p330131	Horizontally Transferred (medium confidence)
		AZOBR_p330132	Horizontally Transferred (high confidence)

		AZOBR_p330133	Horizontally Transferred (medium confidence)
		AZOBR_p330134	Horizontally Transferred (high confidence)
		AZOBR_p330135	Horizontally Transferred (high confidence)
		AZOBR_p330136	Horizontally Transferred (high confidence)
		AZOBR_p330137	Unassigned
		AZOBR_p330138	Horizontally Transferred (high confidence)
		AZOBR_p330139	Horizontally Transferred (high confidence)
		AZOBR_p330140	Horizontally Transferred (high confidence)
		AZOBR_p330141	Ancestral (medium confidence)
		AZOBR_p330142	Unassigned
		AZOBR_p330143	Horizontally Transferred (high confidence)
		AZOBR_p330144	Horizontally Transferred (high confidence)
		AZOBR_p330145	Horizontally Transferred (medium confidence)
		AZOBR_p330146	Horizontally Transferred (high confidence)
		AZOBR_p330147	Horizontally Transferred (high confidence)
		AZOBR_p330148	Horizontally Transferred (high confidence)
		AZOBR_p330149	Horizontally Transferred (high confidence)
		AZOBR_p330150	Horizontally Transferred (high confidence)
		AZOBR_p330151	Horizontally Transferred (high confidence)
		AZOBR_p330152	Ancestral (medium confidence)
		AZOBR_p330153	Horizontally Transferred (high confidence)
		AZOBR_p330154	Ancestral (low confidence)
		AZOBR_p330155	Unassigned
		AZOBR_p330156	Ancestral (high confidence)
		AZOBR_p330157	Ancestral (high confidence)
		AZOBR_p330158	Ancestral (medium confidence)
		AZOBR_p330159	Ancestral (low confidence)
		AZOBR_p330160	Horizontally Transferred (high confidence)
		AZOBR_p330161	Unassigned
		AZOBR_p330162	Unassigned
		AZOBR_p330163	Ancestral (low confidence)
		AZOBR_p340001	Ancestral (low confidence)
		AZOBR_p340002	Ancestral (low confidence)
		AZOBR_p340003	Ancestral (low confidence)
		AZOBR_p340004	Ancestral (low confidence)
		AZOBR_p340005	Ancestral (low confidence)
		AZOBR_p340006	Unassigned
		AZOBR_p340007	Unassigned
		AZOBR_p340008	Horizontally Transferred (high confidence)
		AZOBR_p340009	Unassigned
		AZOBR_p340010	Horizontally Transferred (high confidence)
		AZOBR_p340011	Horizontally Transferred (high confidence)
		AZOBR_p340012	Unassigned
		AZOBR_p340013	Ancestral (low confidence)
		AZOBR_p340014	Ancestral (low confidence)
		AZOBR_p340015	Horizontally Transferred (high confidence)
		AZOBR_p340016	Horizontally Transferred (high confidence)
		AZOBR_p340017	Unassigned
		AZOBR_p340018	Ancestral (low confidence)
		AZOBR_p340019	Unassigned
		AZOBR_p340020	Unassigned
		AZOBR_p340021	Unassigned
		AZOBR_p340022	Unassigned
		AZOBR_p340023	Ancestral (low confidence)
		AZOBR_p340024	Horizontally Transferred (medium confidence)
		AZOBR_p340025	Ancestral (low confidence)
		AZOBR_p340026	Horizontally Transferred (high confidence)
		AZOBR_p340027	Horizontally Transferred (high confidence)
		AZOBR_p340028	Horizontally Transferred (high confidence)
		AZOBR_p340029	Unassigned
		AZOBR_p340030	Ancestral (medium confidence)
		AZOBR_p340031	Ancestral (low confidence)
		AZOBR_p340032	Ancestral (low confidence)
		AZOBR_p340033	Unassigned
		AZOBR_p340034	Unassigned
		AZOBR_p340035	Unassigned
		AZOBR_p340036	Horizontally Transferred (high confidence)
		AZOBR_p340037	Unassigned
		AZOBR_p340038	Horizontally Transferred (high confidence)
		AZOBR_p340039	Horizontally Transferred (high confidence)
		AZOBR_p340040	Horizontally Transferred (high confidence)
		AZOBR_p340041	Horizontally Transferred (high confidence)
		AZOBR_p340042	Unassigned
		AZOBR_p340043	Horizontally Transferred (high confidence)
		AZOBR_p340044	Horizontally Transferred (high confidence)

		AZOBR_p340045	Horizontally Transferred (high confidence)
		AZOBR_p340046	Horizontally Transferred (high confidence)
		AZOBR_p340047	Ancestral (low confidence)
		AZOBR_p340048	Unassigned
		AZOBR_p340049	Horizontally Transferred (high confidence)
		AZOBR_p340050	Unassigned
		AZOBR_p340051	Horizontally Transferred (high confidence)
		AZOBR_p340052	Horizontally Transferred (high confidence)
		AZOBR_p340053	Horizontally Transferred (high confidence)
		AZOBR_p340054	Unassigned
		AZOBR_p340055	Unassigned
		AZOBR_p340056	Ancestral (low confidence)
		AZOBR_p340057	Horizontally Transferred (high confidence)
		AZOBR_p340058	Ancestral (medium confidence)
		AZOBR_p340059	Ancestral (low confidence)
		AZOBR_p340060	Ancestral (low confidence)
		AZOBR_p340061	Ancestral (low confidence)
		AZOBR_p340062	Ancestral (medium confidence)
		AZOBR_p340063	Ancestral (medium confidence)
		AZOBR_p340064	Horizontally Transferred (high confidence)
		AZOBR_p340065	Ancestral (medium confidence)
		AZOBR_p340066	Ancestral (medium confidence)
		AZOBR_p340067	Ancestral (low confidence)
		AZOBR_p340068	Unassigned
		AZOBR_p340069	Ancestral (low confidence)
		AZOBR_p340070	Unassigned
		AZOBR_p340071	Ancestral (low confidence)
		AZOBR_p340072	Horizontally Transferred (high confidence)
		AZOBR_p340073	Horizontally Transferred (high confidence)
		AZOBR_p340074	Horizontally Transferred (high confidence)
		AZOBR_p340075	Horizontally Transferred (high confidence)
		AZOBR_p340076	Horizontally Transferred (high confidence)
		AZOBR_p340077	Unassigned
		AZOBR_p340078	Ancestral (low confidence)
		AZOBR_p340079	Unassigned
		AZOBR_p340080	Ancestral (medium confidence)
		AZOBR_p340081	Ancestral (high confidence)
		AZOBR_p340082	Ancestral (low confidence)
		AZOBR_p340083	Horizontally Transferred (medium confidence)
		AZOBR_p340084	Horizontally Transferred (high confidence)
		AZOBR_p340085	Horizontally Transferred (low confidence)
		AZOBR_p340086	Horizontally Transferred (high confidence)
		AZOBR_p340087	Unassigned
		AZOBR_p340088	Unassigned
		AZOBR_p340089	Horizontally Transferred (high confidence)
		AZOBR_p340090	Unassigned
		AZOBR_p340091	Unassigned
		AZOBR_p340092	Ancestral (low confidence)
		AZOBR_p340093	Horizontally Transferred (high confidence)
		AZOBR_p340094	Unassigned
		AZOBR_p340095	Unassigned
		AZOBR_p340096	Unassigned
		AZOBR_p340097	Horizontally Transferred (high confidence)
		AZOBR_p340098	Unassigned
		AZOBR_p340099	Ancestral (medium confidence)
		AZOBR_p340100	Ancestral (high confidence)
		AZOBR_p340101	Horizontally Transferred (high confidence)
		AZOBR_p340102	Ancestral (low confidence)
		AZOBR_p340103	Ancestral (low confidence)
		AZOBR_p340104	Ancestral (low confidence)
		AZOBR_p340105	Horizontally Transferred (high confidence)
		AZOBR_p340106	Unassigned
		AZOBR_p340107	Unassigned
		AZOBR_p340108	Ancestral (low confidence)
		AZOBR_p340109	Ancestral (low confidence)
		AZOBR_p340110	Horizontally Transferred (high confidence)
		AZOBR_p340111	Unassigned
		AZOBR_p340112	Unassigned
		AZOBR_p340113	Horizontally Transferred (high confidence)
		AZOBR_p340114	Ancestral (low confidence)
		AZOBR_p340115	Ancestral (low confidence)
		AZOBR_p340116	Horizontally Transferred (high confidence)
		AZOBR_p340117	Horizontally Transferred (high confidence)
		AZOBR_p340118	Ancestral (low confidence)
		AZOBR_p340119	Ancestral (low confidence)

		AZOBR_p340120	Horizontally Transferred (low confidence)
		AZOBR_p340121	Horizontally Transferred (low confidence)
		AZOBR_p340122	Horizontally Transferred (low confidence)
		AZOBR_p340123	Ancestral (low confidence)
		AZOBR_p340124	Unassigned
		AZOBR_p340125	Horizontally Transferred (high confidence)
		AZOBR_p340126	Unassigned
		AZOBR_p340127	Ancestral (low confidence)
		AZOBR_p340128	Horizontally Transferred (low confidence)
		AZOBR_p340129	Horizontally Transferred (high confidence)
		AZOBR_p340130	Horizontally Transferred (high confidence)
		AZOBR_p340131	Unassigned
		AZOBR_p340132	Unassigned
		AZOBR_p340133	Unassigned
		AZOBR_p340134	Unassigned
		AZOBR_p340135	Horizontally Transferred (high confidence)
		AZOBR_p340136	Unassigned
		AZOBR_p340137	Unassigned
		AZOBR_p340138	Horizontally Transferred (low confidence)
		AZOBR_p340139	Unassigned
		AZOBR_p340140	Horizontally Transferred (high confidence)
		AZOBR_p340141	Unassigned
		AZOBR_p340142	Horizontally Transferred (high confidence)
		AZOBR_p340143	Horizontally Transferred (high confidence)
		AZOBR_p340144	Horizontally Transferred (high confidence)
		AZOBR_p340145	Unassigned
		AZOBR_p340146	Unassigned
		AZOBR_p340147	Horizontally Transferred (high confidence)
		AZOBR_p340148	Unassigned
		AZOBR_p340149	Horizontally Transferred (high confidence)
		AZOBR_p340150	Unassigned
		AZOBR_p340151	Unassigned
		AZOBR_p340152	Unassigned
		AZOBR_p340153	Horizontally Transferred (high confidence)
		AZOBR_p340154	Ancestral (medium confidence)
		AZOBR_p340155	Ancestral (low confidence)
		AZOBR_p340156	Unassigned
		AZOBR_p340157	Ancestral (low confidence)
		AZOBR_p340158	Horizontally Transferred (high confidence)
		AZOBR_p340159	Unassigned
		AZOBR_p340160	Unassigned
		AZOBR_p340161	Unassigned
		AZOBR_p340162	Horizontally Transferred (high confidence)
		AZOBR_p340163	Unassigned
		AZOBR_p340164	Unassigned
		AZOBR_p340165	Horizontally Transferred (high confidence)
		AZOBR_p340166	Horizontally Transferred (high confidence)
		AZOBR_p340167	Unassigned
		AZOBR_p340168	Ancestral (medium confidence)
		AZOBR_p340169	Unassigned
		AZOBR_p340170	Unassigned
		AZOBR_p340171	Unassigned
		AZOBR_p340172	Unassigned
		AZOBR_p340173	Unassigned
		AZOBR_p340174	Unassigned
		AZOBR_p340175	Unassigned
		AZOBR_p340176	Unassigned
		AZOBR_p340177	Unassigned
		AZOBR_p340178	Unassigned
		AZOBR_p340179	Unassigned
		AZOBR_p340180	Horizontally Transferred (low confidence)
		AZOBR_p340181	Horizontally Transferred (high confidence)
		AZOBR_p340182	Horizontally Transferred (high confidence)
		AZOBR_p340183	Horizontally Transferred (high confidence)
		AZOBR_p340184	Unassigned
		AZOBR_p340185	Unassigned
		AZOBR_p340186	Unassigned
		AZOBR_p340187	Unassigned
		AZOBR_p340188	Unassigned
		AZOBR_p340189	Unassigned
		AZOBR_p340190	Unassigned
		AZOBR_p340191	Ancestral (high confidence)
		AZOBR_p340192	Horizontally Transferred (high confidence)
		AZOBR_p340193	Unassigned
		AZOBR_p340194	Ancestral (low confidence)

		AZOBR_p340195	Unassigned
		AZOBR_p340196	Horizontally Transferred (low confidence)
		AZOBR_p340197	Horizontally Transferred (high confidence)
		AZOBR_p340198	Unassigned
		AZOBR_p340199	Horizontally Transferred (high confidence)
		AZOBR_p340200	Ancestral (low confidence)
		AZOBR_p340201	Ancestral (low confidence)
		AZOBR_p340202	Horizontally Transferred (high confidence)
		AZOBR_p340203	Horizontally Transferred (high confidence)
		AZOBR_p340204	Unassigned
		AZOBR_p340205	Ancestral (low confidence)
		AZOBR_p340206	Ancestral (low confidence)
		AZOBR_p340207	Unassigned
		AZOBR_p340208	Horizontally Transferred (high confidence)
		AZOBR_p340209	Horizontally Transferred (high confidence)
		AZOBR_p340210	Horizontally Transferred (medium confidence)
		AZOBR_p340211	Ancestral (low confidence)
		AZOBR_p340212	Ancestral (low confidence)
		AZOBR_p340213	Ancestral (low confidence)
		AZOBR_p340214	Ancestral (low confidence)
		AZOBR_p340215	Horizontally Transferred (high confidence)
		AZOBR_p340216	Ancestral (low confidence)
		AZOBR_p340217	Horizontally Transferred (high confidence)
		AZOBR_p340218	Horizontally Transferred (high confidence)
		AZOBR_p340219	Horizontally Transferred (high confidence)
		AZOBR_p340220	Horizontally Transferred (high confidence)
		AZOBR_p340221	Ancestral (medium confidence)
		AZOBR_p340222	Unassigned
		AZOBR_p350001	Ancestral (high confidence)
		AZOBR_p350002	Horizontally Transferred (high confidence)
		AZOBR_p350003	Horizontally Transferred (high confidence)
		AZOBR_p350004	Horizontally Transferred (high confidence)
		AZOBR_p350005	Horizontally Transferred (high confidence)
		AZOBR_p350006	Horizontally Transferred (high confidence)
		AZOBR_p350007	Ancestral (low confidence)
		AZOBR_p350008	Horizontally Transferred (high confidence)
		AZOBR_p350009	Unassigned
		AZOBR_p350010	Horizontally Transferred (high confidence)
		AZOBR_p350011	Horizontally Transferred (high confidence)
		AZOBR_p350012	Horizontally Transferred (high confidence)
		AZOBR_p350013	Horizontally Transferred (high confidence)
		AZOBR_p350014	Ancestral (low confidence)
		AZOBR_p350015	Horizontally Transferred (high confidence)
		AZOBR_p350016	Ancestral (low confidence)
		AZOBR_p350017	Unassigned
		AZOBR_p350018	Ancestral (low confidence)
		AZOBR_p350019	Unassigned
		AZOBR_p350020	Unassigned
		AZOBR_p350021	Unassigned
		AZOBR_p350022	Unassigned
		AZOBR_p350023	Horizontally Transferred (high confidence)
		AZOBR_p350024	Ancestral (medium confidence)
		AZOBR_p350025	Horizontally Transferred (high confidence)
		AZOBR_p350026	Horizontally Transferred (high confidence)
		AZOBR_p350027	Ancestral (high confidence)
		AZOBR_p350028	Ancestral (high confidence)
		AZOBR_p350029	Ancestral (medium confidence)
		AZOBR_p350030	Ancestral (low confidence)
		AZOBR_p350031	Unassigned
		AZOBR_p350032	Horizontally Transferred (medium confidence)
		AZOBR_p350033	Unassigned
		AZOBR_p350034	Ancestral (medium confidence)
		AZOBR_p350035	Ancestral (medium confidence)
		AZOBR_p350036	Ancestral (low confidence)
		AZOBR_p350037	Ancestral (low confidence)
		AZOBR_p350038	Horizontally Transferred (high confidence)
		AZOBR_p350039	Horizontally Transferred (high confidence)
		AZOBR_p350040	Ancestral (low confidence)
		AZOBR_p350041	Horizontally Transferred (high confidence)
		AZOBR_p350042	Horizontally Transferred (high confidence)
		AZOBR_p350043	Ancestral (low confidence)
		AZOBR_p350044	Horizontally Transferred (high confidence)
		AZOBR_p350045	Unassigned
		AZOBR_p350046	Horizontally Transferred (high confidence)
		AZOBR_p350047	Horizontally Transferred (high confidence)

		AZOBR_p350048	Horizontally Transferred (high confidence)
		AZOBR_p350049	Horizontally Transferred (medium confidence)
		AZOBR_p350050	Ancestral (medium confidence)
		AZOBR_p350051	Ancestral (low confidence)
		AZOBR_p350052	Horizontally Transferred (medium confidence)
		AZOBR_p350053	Horizontally Transferred (high confidence)
		AZOBR_p350054	Horizontally Transferred (high confidence)
		AZOBR_p350055	Horizontally Transferred (high confidence)
		AZOBR_p350056	Unassigned
		AZOBR_p350057	Horizontally Transferred (high confidence)
		AZOBR_p350058	Horizontally Transferred (high confidence)
		AZOBR_p350059	Horizontally Transferred (low confidence)
		AZOBR_p350060	Unassigned
		AZOBR_p350061	Horizontally Transferred (high confidence)
		AZOBR_p350062	Ancestral (medium confidence)
		AZOBR_p350063	Ancestral (high confidence)
		AZOBR_p350064	Unassigned
		AZOBR_p350065	Horizontally Transferred (high confidence)
		AZOBR_p350066	Ancestral (low confidence)
		AZOBR_p350067	Ancestral (low confidence)
		AZOBR_p350068	Horizontally Transferred (high confidence)
		AZOBR_p350069	Horizontally Transferred (high confidence)
		AZOBR_p350070	Horizontally Transferred (high confidence)
		AZOBR_p350071	Unassigned
		AZOBR_p350072	Unassigned
		AZOBR_p350073	Horizontally Transferred (high confidence)
		AZOBR_p350074	Horizontally Transferred (high confidence)
		AZOBR_p350075	Horizontally Transferred (high confidence)
		AZOBR_p350076	Horizontally Transferred (high confidence)
		AZOBR_p350077	Horizontally Transferred (high confidence)
		AZOBR_p350078	Horizontally Transferred (high confidence)
		AZOBR_p350079	Horizontally Transferred (high confidence)
		AZOBR_p350080	Horizontally Transferred (high confidence)
		AZOBR_p350081	Unassigned
		AZOBR_p350082	Unassigned
		AZOBR_p350083	Horizontally Transferred (high confidence)
		AZOBR_p350084	Horizontally Transferred (high confidence)
		AZOBR_p350085	Ancestral (low confidence)
		AZOBR_p350086	Unassigned
		AZOBR_p350087	Unassigned
		AZOBR_p350088	Unassigned
		AZOBR_p350089	Unassigned
		AZOBR_p350090	Unassigned
		AZOBR_p350091	Horizontally Transferred (high confidence)
		AZOBR_p360001	Ancestral (low confidence)
		AZOBR_p360002	Unassigned
		AZOBR_p360003	Horizontally Transferred (high confidence)
		AZOBR_p360004	Ancestral (low confidence)
		AZOBR_p360005	Horizontally Transferred (high confidence)
		AZOBR_p360006	Horizontally Transferred (high confidence)
		AZOBR_p360007	Ancestral (low confidence)
		AZOBR_p410001	Horizontally Transferred (high confidence)
		AZOBR_p410002	Unassigned
		AZOBR_p410003	Unassigned
		AZOBR_p410004	Ancestral (low confidence)
		AZOBR_p410005	Ancestral (low confidence)
		AZOBR_p410006	Horizontally Transferred (low confidence)
		AZOBR_p410007	Horizontally Transferred (high confidence)
		AZOBR_p410008	Horizontally Transferred (high confidence)
		AZOBR_p410009	Horizontally Transferred (high confidence)
		AZOBR_p410010	Horizontally Transferred (high confidence)
		AZOBR_p410011	Horizontally Transferred (high confidence)
		AZOBR_p410012	Horizontally Transferred (high confidence)
		AZOBR_p410013	Horizontally Transferred (high confidence)
		AZOBR_p410014	Horizontally Transferred (medium confidence)
		AZOBR_p410015	Horizontally Transferred (medium confidence)
		AZOBR_p410016	Horizontally Transferred (medium confidence)
		AZOBR_p410017	Horizontally Transferred (high confidence)
		AZOBR_p410018	Horizontally Transferred (high confidence)
		AZOBR_p410019	Horizontally Transferred (high confidence)
		AZOBR_p410020	Horizontally Transferred (high confidence)
		AZOBR_p410021	Ancestral (medium confidence)
		AZOBR_p410022	Ancestral (high confidence)
		AZOBR_p410023	Ancestral (medium confidence)
		AZOBR_p410024	Ancestral (low confidence)

		AZOBR_p410025	Horizontally Transferred (medium confidence)
		AZOBR_p410026	Horizontally Transferred (high confidence)
		AZOBR_p410027	Horizontally Transferred (high confidence)
		AZOBR_p410028	Unassigned
		AZOBR_p410029	Ancestral (low confidence)
		AZOBR_p410030	Unassigned
		AZOBR_p410031	Ancestral (medium confidence)
		AZOBR_p410032	Ancestral (medium confidence)
		AZOBR_p410033	Ancestral (medium confidence)
		AZOBR_p410034	Horizontally Transferred (high confidence)
		AZOBR_p410035	Horizontally Transferred (high confidence)
		AZOBR_p410036	Ancestral (low confidence)
		AZOBR_p410037	Unassigned
		AZOBR_p410038	Ancestral (medium confidence)
		AZOBR_p410039	Unassigned
		AZOBR_p410040	Ancestral (medium confidence)
		AZOBR_p410041	Horizontally Transferred (high confidence)
		AZOBR_p410042	Ancestral (low confidence)
		AZOBR_p410043	Ancestral (low confidence)
		AZOBR_p410044	Ancestral (low confidence)
		AZOBR_p410045	Horizontally Transferred (high confidence)
		AZOBR_p410046	Ancestral (medium confidence)
		AZOBR_p410047	Ancestral (low confidence)
		AZOBR_p410048	Horizontally Transferred (high confidence)
		AZOBR_p410049	Horizontally Transferred (high confidence)
		AZOBR_p410050	Horizontally Transferred (high confidence)
		AZOBR_p410051	Horizontally Transferred (high confidence)
		AZOBR_p410052	Horizontally Transferred (medium confidence)
		AZOBR_p410053	Horizontally Transferred (high confidence)
		AZOBR_p410054	Unassigned
		AZOBR_p410055	Ancestral (medium confidence)
		AZOBR_p410056	Horizontally Transferred (high confidence)
		AZOBR_p410057	Ancestral (medium confidence)
		AZOBR_p410058	Ancestral (medium confidence)
		AZOBR_p410059	Ancestral (medium confidence)
		AZOBR_p410060	Horizontally Transferred (medium confidence)
		AZOBR_p410061	Ancestral (high confidence)
		AZOBR_p410062	Ancestral (low confidence)
		AZOBR_p410063	Ancestral (low confidence)
		AZOBR_p410064	Ancestral (high confidence)
		AZOBR_p410065	Ancestral (high confidence)
		AZOBR_p410066	Ancestral (medium confidence)
		AZOBR_p410067	Ancestral (medium confidence)
		AZOBR_p410068	Ancestral (medium confidence)
		AZOBR_p410069	Ancestral (low confidence)
		AZOBR_p410070	Ancestral (high confidence)
		AZOBR_p410071	Ancestral (high confidence)
		AZOBR_p410072	Ancestral (medium confidence)
		AZOBR_p410073	Ancestral (medium confidence)
		AZOBR_p410074	Horizontally Transferred (high confidence)
		AZOBR_p410075	Horizontally Transferred (high confidence)
		AZOBR_p410076	Horizontally Transferred (medium confidence)
		AZOBR_p410077	Horizontally Transferred (high confidence)
		AZOBR_p410078	Horizontally Transferred (high confidence)
		AZOBR_p410079	Unassigned
		AZOBR_p410080	Unassigned
		AZOBR_p410081	Ancestral (low confidence)
		AZOBR_p410082	Unassigned
		AZOBR_p410083	Horizontally Transferred (medium confidence)
		AZOBR_p410084	Horizontally Transferred (medium confidence)
		AZOBR_p410085	Ancestral (medium confidence)
		AZOBR_p410086	Horizontally Transferred (high confidence)
		AZOBR_p410087	Ancestral (low confidence)
		AZOBR_p410088	Horizontally Transferred (high confidence)
		AZOBR_p410089	Horizontally Transferred (high confidence)
		AZOBR_p410090	Unassigned
		AZOBR_p410091	Ancestral (medium confidence)
		AZOBR_p410092	Horizontally Transferred (high confidence)
		AZOBR_p410093	Ancestral (high confidence)
		AZOBR_p410094	Ancestral (low confidence)
		AZOBR_p410095	Ancestral (low confidence)
		AZOBR_p410096	Ancestral (low confidence)
		AZOBR_p410097	Ancestral (low confidence)
		AZOBR_p410098	Ancestral (medium confidence)
		AZOBR_p410099	Unassigned

		AZOBR_p410100	Horizontally Transferred (high confidence)
		AZOBR_p410101	Unassigned
		AZOBR_p410102	Horizontally Transferred (medium confidence)
		AZOBR_p410103	Horizontally Transferred (high confidence)
		AZOBR_p410104	Unassigned
		AZOBR_p410105	Horizontally Transferred (high confidence)
		AZOBR_p410106	Horizontally Transferred (medium confidence)
		AZOBR_p420001	Ancestral (medium confidence)
		AZOBR_p420002	Horizontally Transferred (high confidence)
		AZOBR_p420003	Ancestral (low confidence)
		AZOBR_p420004	Horizontally Transferred (high confidence)
		AZOBR_p420005	Horizontally Transferred (high confidence)
		AZOBR_p420006	Horizontally Transferred (high confidence)
		AZOBR_p420007	Unassigned
		AZOBR_p420008	Unassigned
		AZOBR_p420009	Horizontally Transferred (high confidence)
		AZOBR_p420010	Ancestral (high confidence)
		AZOBR_p420011	Horizontally Transferred (high confidence)
		AZOBR_p420012	Horizontally Transferred (high confidence)
		AZOBR_p420013	Ancestral (high confidence)
		AZOBR_p420014	Unassigned
		AZOBR_p420015	Horizontally Transferred (high confidence)
		AZOBR_p420016	Ancestral (medium confidence)
		AZOBR_p420017	Ancestral (medium confidence)
		AZOBR_p420018	Ancestral (low confidence)
		AZOBR_p420019	Horizontally Transferred (high confidence)
		AZOBR_p420020	Ancestral (medium confidence)
		AZOBR_p420021	Ancestral (low confidence)
		AZOBR_p420022	Unassigned
		AZOBR_p420023	Horizontally Transferred (high confidence)
		AZOBR_p420024	Ancestral (high confidence)
		AZOBR_p420025	Ancestral (medium confidence)
		AZOBR_p420026	Ancestral (high confidence)
		AZOBR_p420027	Horizontally Transferred (high confidence)
		AZOBR_p420028	Horizontally Transferred (high confidence)
		AZOBR_p420029	Horizontally Transferred (high confidence)
		AZOBR_p420030	Horizontally Transferred (high confidence)
		AZOBR_p420031	Horizontally Transferred (high confidence)
		AZOBR_p420032	Horizontally Transferred (high confidence)
		AZOBR_p420033	Ancestral (low confidence)
		AZOBR_p430001	Unassigned
		AZOBR_p430002	Unassigned
		AZOBR_p430003	Ancestral (low confidence)
		AZOBR_p430004	Horizontally Transferred (high confidence)
		AZOBR_p430005	Horizontally Transferred (high confidence)
		AZOBR_p430006	Horizontally Transferred (high confidence)
		AZOBR_p430007	Horizontally Transferred (high confidence)
		AZOBR_p430008	Horizontally Transferred (high confidence)
		AZOBR_p430009	Ancestral (low confidence)
		AZOBR_p430010	Ancestral (low confidence)
		AZOBR_p430011	Horizontally Transferred (medium confidence)
		AZOBR_p430012	Horizontally Transferred (high confidence)
		AZOBR_p430013	Horizontally Transferred (high confidence)
		AZOBR_p430014	Unassigned
		AZOBR_p430015	Unassigned
		AZOBR_p430016	Unassigned
		AZOBR_p430017	Unassigned
		AZOBR_p430018	Unassigned
		AZOBR_p430019	Unassigned
		AZOBR_p430020	Unassigned
		AZOBR_p430021	Horizontally Transferred (low confidence)
		AZOBR_p430022	Horizontally Transferred (low confidence)
		AZOBR_p430023	Unassigned
		AZOBR_p430024	Unassigned
		AZOBR_p430025	Ancestral (high confidence)
		AZOBR_p430026	Unassigned
		AZOBR_p430027	Unassigned
		AZOBR_p430028	Ancestral (low confidence)
		AZOBR_p430029	Horizontally Transferred (high confidence)
		AZOBR_p430030	Horizontally Transferred (high confidence)
		AZOBR_p430031	Horizontally Transferred (high confidence)
		AZOBR_p430032	Horizontally Transferred (low confidence)
		AZOBR_p430033	Horizontally Transferred (medium confidence)
		AZOBR_p430034	Unassigned
		AZOBR_p430035	Horizontally Transferred (high confidence)

		AZOBR_p430036	Horizontally Transferred (high confidence)
		AZOBR_p430037	Horizontally Transferred (high confidence)
		AZOBR_p430038	Ancestral (medium confidence)
		AZOBR_p430039	Ancestral (low confidence)
		AZOBR_p430040	Ancestral (low confidence)
		AZOBR_p430041	Horizontally Transferred (high confidence)
		AZOBR_p430042	Ancestral (low confidence)
		AZOBR_p430043	Ancestral (low confidence)
		AZOBR_p430044	Horizontally Transferred (high confidence)
		AZOBR_p430045	Horizontally Transferred (high confidence)
		AZOBR_p430046	Horizontally Transferred (high confidence)
		AZOBR_p430047	Horizontally Transferred (high confidence)
		AZOBR_p430048	Unassigned
		AZOBR_p430049	Horizontally Transferred (medium confidence)
		AZOBR_p430050	Ancestral (high confidence)
		AZOBR_p430051	Ancestral (high confidence)
		AZOBR_p430052	Unassigned
		AZOBR_p430053	Ancestral (high confidence)
		AZOBR_p430054	Ancestral (high confidence)
		AZOBR_p430055	Ancestral (high confidence)
		AZOBR_p430056	Horizontally Transferred (high confidence)
		AZOBR_p430057	Ancestral (high confidence)
		AZOBR_p430058	Ancestral (high confidence)
		AZOBR_p430059	Unassigned
		AZOBR_p430060	Ancestral (high confidence)
		AZOBR_p430061	Unassigned
		AZOBR_p430062	Ancestral (medium confidence)
		AZOBR_p430063	Ancestral (high confidence)
		AZOBR_p430064	Ancestral (medium confidence)
		AZOBR_p430065	Unassigned
		AZOBR_p430066	Ancestral (high confidence)
		AZOBR_p440001	Ancestral (medium confidence)
		AZOBR_p440002	Ancestral (medium confidence)
		AZOBR_p440003	Unassigned
		AZOBR_p440004	Ancestral (medium confidence)
		AZOBR_p440005	Ancestral (low confidence)
		AZOBR_p440006	Horizontally Transferred (high confidence)
		AZOBR_p440007	Ancestral (low confidence)
		AZOBR_p440008	Ancestral (low confidence)
		AZOBR_p440009	Horizontally Transferred (high confidence)
		AZOBR_p440010	Horizontally Transferred (high confidence)
		AZOBR_p440011	Unassigned
		AZOBR_p440012	Horizontally Transferred (high confidence)
		AZOBR_p440013	Horizontally Transferred (high confidence)
		AZOBR_p440014	Ancestral (high confidence)
		AZOBR_p440015	Ancestral (medium confidence)
		AZOBR_p440016	Ancestral (high confidence)
		AZOBR_p440017	Unassigned
		AZOBR_p440018	Ancestral (medium confidence)
		AZOBR_p440019	Ancestral (medium confidence)
		AZOBR_p440020	Unassigned
		AZOBR_p440021	Horizontally Transferred (high confidence)
		AZOBR_p440022	Horizontally Transferred (high confidence)
		AZOBR_p440023	Horizontally Transferred (high confidence)
		AZOBR_p440024	Ancestral (low confidence)
		AZOBR_p440025	Unassigned
		AZOBR_p440026	Ancestral (high confidence)
		AZOBR_p440027	Horizontally Transferred (high confidence)
		AZOBR_p440028	Ancestral (low confidence)
		AZOBR_p440029	Horizontally Transferred (high confidence)
		AZOBR_p440030	Ancestral (low confidence)
		AZOBR_p440031	Horizontally Transferred (high confidence)
		AZOBR_p440032	Horizontally Transferred (high confidence)
		AZOBR_p440033	Ancestral (low confidence)
		AZOBR_p440034	Ancestral (high confidence)
		AZOBR_p440035	Horizontally Transferred (high confidence)
		AZOBR_p440036	Ancestral (low confidence)
		AZOBR_p440037	Unassigned
		AZOBR_p440038	Unassigned
		AZOBR_p440039	Unassigned
		AZOBR_p440040	Unassigned
		AZOBR_p440041	Ancestral (medium confidence)
		AZOBR_p440042	Ancestral (high confidence)
		AZOBR_p440043	Ancestral (high confidence)
		AZOBR_p440044	Ancestral (high confidence)

		AZOBR_p440045	Ancestral (low confidence)
		AZOBR_p440046	Horizontally Transferred (high confidence)
		AZOBR_p440047	Horizontally Transferred (high confidence)
		AZOBR_p440048	Horizontally Transferred (high confidence)
		AZOBR_p440049	Horizontally Transferred (high confidence)
		AZOBR_p440050	Ancestral (low confidence)
		AZOBR_p440051	Ancestral (high confidence)
		AZOBR_p440052	Horizontally Transferred (high confidence)
		AZOBR_p440053	Ancestral (medium confidence)
		AZOBR_p440054	Ancestral (low confidence)
		AZOBR_p440055	Ancestral (low confidence)
		AZOBR_p440056	Ancestral (low confidence)
		AZOBR_p440057	Ancestral (low confidence)
		AZOBR_p440058	Horizontally Transferred (high confidence)
		AZOBR_p440059	Horizontally Transferred (high confidence)
		AZOBR_p440060	Horizontally Transferred (high confidence)
		AZOBR_p440061	Horizontally Transferred (high confidence)
		AZOBR_p440062	Horizontally Transferred (low confidence)
		AZOBR_p440063	Horizontally Transferred (high confidence)
		AZOBR_p440064	Ancestral (low confidence)
		AZOBR_p440065	Ancestral (low confidence)
		AZOBR_p440066	Unassigned
		AZOBR_p440067	Ancestral (medium confidence)
		AZOBR_p440068	Horizontally Transferred (high confidence)
		AZOBR_p440069	Horizontally Transferred (medium confidence)
		AZOBR_p440070	Horizontally Transferred (high confidence)
		AZOBR_p440071	Horizontally Transferred (low confidence)
		AZOBR_p440072	Horizontally Transferred (high confidence)
		AZOBR_p440073	Horizontally Transferred (low confidence)
		AZOBR_p440074	Horizontally Transferred (high confidence)
		AZOBR_p440075	Horizontally Transferred (high confidence)
		AZOBR_p440076	Ancestral (high confidence)
		AZOBR_p440077	Unassigned
		AZOBR_p440078	Unassigned
		AZOBR_p440079	Ancestral (medium confidence)
		AZOBR_p440080	Unassigned
		AZOBR_p440081	Unassigned
		AZOBR_p440082	Horizontally Transferred (high confidence)
		AZOBR_p440083	Horizontally Transferred (low confidence)
		AZOBR_p440084	Unassigned
		AZOBR_p440085	Ancestral (low confidence)
		AZOBR_p440086	Horizontally Transferred (high confidence)
		AZOBR_p440087	Unassigned
		AZOBR_p440088	Unassigned
		AZOBR_p440089	Horizontally Transferred (high confidence)
		AZOBR_p440090	Horizontally Transferred (high confidence)
		AZOBR_p440091	Horizontally Transferred (medium confidence)
		AZOBR_p440092	Ancestral (medium confidence)
		AZOBR_p440093	Ancestral (medium confidence)
		AZOBR_p440094	Ancestral (high confidence)
		AZOBR_p440095	Ancestral (medium confidence)
		AZOBR_p440096	Horizontally Transferred (high confidence)
		AZOBR_p440097	Ancestral (low confidence)
		AZOBR_p440098	Unassigned
		AZOBR_p440099	Ancestral (low confidence)
		AZOBR_p440100	Ancestral (low confidence)
		AZOBR_p440101	Horizontally Transferred (high confidence)
		AZOBR_p440102	Ancestral (medium confidence)
		AZOBR_p440103	Ancestral (medium confidence)
		AZOBR_p440104	Unassigned
		AZOBR_p440105	Ancestral (low confidence)
		AZOBR_p440106	Unassigned
		AZOBR_p440107	Ancestral (high confidence)
		AZOBR_p440108	Ancestral (medium confidence)
		AZOBR_p440109	Unassigned
		AZOBR_p440110	Ancestral (high confidence)
		AZOBR_p440111	Ancestral (medium confidence)
		AZOBR_p440112	Ancestral (low confidence)
		AZOBR_p440113	Horizontally Transferred (medium confidence)
		AZOBR_p440114	Horizontally Transferred (medium confidence)
		AZOBR_p440115	Ancestral (low confidence)
		AZOBR_p440116	Ancestral (low confidence)
		AZOBR_p440117	Ancestral (low confidence)
		AZOBR_p440118	Ancestral (low confidence)
		AZOBR_p440119	Horizontally Transferred (high confidence)

		AZOBR_p440120	Ancestral (low confidence)
		AZOBR_p440121	Unassigned
		AZOBR_p440122	Ancestral (low confidence)
		AZOBR_p440123	Ancestral (medium confidence)
		AZOBR_p440124	Horizontally Transferred (high confidence)
		AZOBR_p440125	Unassigned
		AZOBR_p440126	Horizontally Transferred (high confidence)
		AZOBR_p440127	Ancestral (medium confidence)
		AZOBR_p440128	Ancestral (medium confidence)
		AZOBR_p440129	Ancestral (low confidence)
		AZOBR_p440130	Ancestral (low confidence)
		AZOBR_p440131	Ancestral (high confidence)
		AZOBR_p440132	Horizontally Transferred (high confidence)
		AZOBR_p440133	Ancestral (low confidence)
		AZOBR_p440134	Ancestral (low confidence)
		AZOBR_p440135	Ancestral (low confidence)
		AZOBR_p440136	Horizontally Transferred (medium confidence)
		AZOBR_p440137	Ancestral (low confidence)
		AZOBR_p440138	Ancestral (low confidence)
		AZOBR_p440139	Horizontally Transferred (high confidence)
		AZOBR_p440140	Horizontally Transferred (high confidence)
		AZOBR_p440141	Ancestral (high confidence)
		AZOBR_p440142	Horizontally Transferred (high confidence)
		AZOBR_p440143	Ancestral (low confidence)
		AZOBR_p440144	Ancestral (high confidence)
		AZOBR_p440145	Ancestral (medium confidence)
		AZOBR_p440146	Ancestral (high confidence)
		AZOBR_p440147	Ancestral (high confidence)
		AZOBR_p440148	Ancestral (medium confidence)
		AZOBR_p440149	Ancestral (high confidence)
		AZOBR_p440150	Unassigned
		AZOBR_p440151	Ancestral (medium confidence)
		AZOBR_p440152	Ancestral (high confidence)
		AZOBR_p440153	Ancestral (medium confidence)
		AZOBR_p440154	Ancestral (medium confidence)
		AZOBR_p440155	Horizontally Transferred (high confidence)
		AZOBR_p440156	Horizontally Transferred (high confidence)
		AZOBR_p440157	Horizontally Transferred (high confidence)
		AZOBR_p440158	Horizontally Transferred (high confidence)
		AZOBR_p440159	Horizontally Transferred (high confidence)
		AZOBR_p440160	Horizontally Transferred (high confidence)
		AZOBR_p440161	Ancestral (low confidence)
		AZOBR_p440162	Horizontally Transferred (high confidence)
		AZOBR_p440163	Horizontally Transferred (high confidence)
		AZOBR_p440164	Horizontally Transferred (high confidence)
		AZOBR_p440165	Horizontally Transferred (high confidence)
		AZOBR_p440166	Horizontally Transferred (high confidence)
		AZOBR_p440167	Horizontally Transferred (high confidence)
		AZOBR_p440168	Horizontally Transferred (high confidence)
		AZOBR_p440169	Horizontally Transferred (high confidence)
		AZOBR_p440170	Horizontally Transferred (high confidence)
		AZOBR_p440171	Horizontally Transferred (high confidence)
		AZOBR_p440172	Horizontally Transferred (high confidence)
		AZOBR_p440173	Horizontally Transferred (high confidence)
		AZOBR_p440174	Unassigned
		AZOBR_p440175	Ancestral (low confidence)
		AZOBR_p440176	Unassigned
		AZOBR_p440177	Horizontally Transferred (high confidence)
		AZOBR_p440178	Horizontally Transferred (high confidence)
		AZOBR_p440179	Horizontally Transferred (high confidence)
		AZOBR_p440180	Horizontally Transferred (high confidence)
		AZOBR_p440181	Unassigned
		AZOBR_p440182	Horizontally Transferred (high confidence)
		AZOBR_p440183	Horizontally Transferred (medium confidence)
		AZOBR_p440184	Ancestral (medium confidence)
		AZOBR_p440185	Horizontally Transferred (high confidence)
		AZOBR_p440186	Unassigned
		AZOBR_p440187	Horizontally Transferred (high confidence)
		AZOBR_p440188	Unassigned
		AZOBR_p450001	Unassigned
		AZOBR_p450002	Unassigned
		AZOBR_p450003	Unassigned
		AZOBR_p450004	Horizontally Transferred (high confidence)
		AZOBR_p450005	Ancestral (low confidence)
		AZOBR_p450006	Horizontally Transferred (high confidence)

		AZOBR_p450007	Unassigned
		AZOBR_p450008	Unassigned
		AZOBR_p450009	Horizontally Transferred (high confidence)
		AZOBR_p450010	Horizontally Transferred (high confidence)
		AZOBR_p450011	Horizontally Transferred (high confidence)
		AZOBR_p450012	Horizontally Transferred (high confidence)
		AZOBR_p450013	Horizontally Transferred (high confidence)
		AZOBR_p450014	Horizontally Transferred (high confidence)
		AZOBR_p460001	Horizontally Transferred (high confidence)
		AZOBR_p460002	Horizontally Transferred (high confidence)
		AZOBR_p460003	Ancestral (medium confidence)
		AZOBR_p460004	Unassigned
		AZOBR_p460005	Horizontally Transferred (high confidence)
		AZOBR_p460006	Unassigned
		AZOBR_p460007	Unassigned
		AZOBR_p460008	Ancestral (low confidence)
		AZOBR_p460009	Unassigned
		AZOBR_p460010	Horizontally Transferred (high confidence)
		AZOBR_p460011	Horizontally Transferred (high confidence)
		AZOBR_p460012	Horizontally Transferred (high confidence)
		AZOBR_p460013	Horizontally Transferred (high confidence)
		AZOBR_p460014	Horizontally Transferred (high confidence)
		AZOBR_p460015	Ancestral (low confidence)
		AZOBR_p460016	Ancestral (medium confidence)
		AZOBR_p460017	Horizontally Transferred (high confidence)
		AZOBR_p460018	Unassigned
		AZOBR_p460019	Unassigned
		AZOBR_p460020	Unassigned
		AZOBR_p460021	Unassigned
		AZOBR_p460022	Unassigned
		AZOBR_p460023	Horizontally Transferred (high confidence)
		AZOBR_p460024	Ancestral (low confidence)
		AZOBR_p460025	Horizontally Transferred (high confidence)
		AZOBR_p460026	Horizontally Transferred (high confidence)
		AZOBR_p460027	Horizontally Transferred (high confidence)
		AZOBR_p460028	Horizontally Transferred (high confidence)
		AZOBR_p460029	Horizontally Transferred (high confidence)
		AZOBR_p460030	Horizontally Transferred (high confidence)
		AZOBR_p460031	Horizontally Transferred (medium confidence)
		AZOBR_p460032	Unassigned
		AZOBR_p460033	Horizontally Transferred (high confidence)
		AZOBR_p460034	Horizontally Transferred (high confidence)
		AZOBR_p460035	Horizontally Transferred (high confidence)
		AZOBR_p460036	Horizontally Transferred (high confidence)
		AZOBR_p460037	Ancestral (low confidence)
		AZOBR_p460038	Ancestral (low confidence)
		AZOBR_p460039	Ancestral (low confidence)
		AZOBR_p460040	Horizontally Transferred (high confidence)
		AZOBR_p460041	Horizontally Transferred (high confidence)
		AZOBR_p460042	Horizontally Transferred (medium confidence)
		AZOBR_p460043	Horizontally Transferred (medium confidence)
		AZOBR_p460044	Ancestral (medium confidence)
		AZOBR_p460045	Ancestral (high confidence)
		AZOBR_p460046	Ancestral (medium confidence)
		AZOBR_p460047	Ancestral (low confidence)
		AZOBR_p460048	Ancestral (low confidence)
		AZOBR_p460049	Unassigned
		AZOBR_p460050	Horizontally Transferred (high confidence)
		AZOBR_p460051	Unassigned
		AZOBR_p460052	Horizontally Transferred (low confidence)
		AZOBR_p460053	Horizontally Transferred (high confidence)
		AZOBR_p460054	Horizontally Transferred (high confidence)
		AZOBR_p460055	Horizontally Transferred (high confidence)
		AZOBR_p460056	Unassigned
		AZOBR_p460057	Unassigned
		AZOBR_p460058	Horizontally Transferred (high confidence)
		AZOBR_p460059	Horizontally Transferred (high confidence)
		AZOBR_p460060	Horizontally Transferred (high confidence)
		AZOBR_p460061	Horizontally Transferred (high confidence)
		AZOBR_p460062	Horizontally Transferred (high confidence)
		AZOBR_p460063	Horizontally Transferred (high confidence)
		AZOBR_p460064	Unassigned
		AZOBR_p460065	Ancestral (low confidence)
		AZOBR_p460066	Horizontally Transferred (medium confidence)
		AZOBR_p460067	Ancestral (low confidence)

		AZOBR_p460068	Horizontally Transferred (high confidence)
		AZOBR_p460069	Unassigned
		AZOBR_p460070	Unassigned
		AZOBR_p460071	Ancestral (low confidence)
		AZOBR_p460072	Horizontally Transferred (high confidence)
		AZOBR_p460073	Unassigned
		AZOBR_p460074	Horizontally Transferred (high confidence)
		AZOBR_p460075	Unassigned
		AZOBR_p460076	Unassigned
		AZOBR_p460077	Ancestral (low confidence)
		AZOBR_p460078	Horizontally Transferred (low confidence)
		AZOBR_p460079	Horizontally Transferred (low confidence)
		AZOBR_p460080	Horizontally Transferred (medium confidence)
		AZOBR_p460081	Unassigned
		AZOBR_p460082	Horizontally Transferred (low confidence)
		AZOBR_p460083	Horizontally Transferred (low confidence)
		AZOBR_p460084	Ancestral (low confidence)
		AZOBR_p460085	Horizontally Transferred (low confidence)
		AZOBR_p460086	Horizontally Transferred (low confidence)
		AZOBR_p460087	Horizontally Transferred (low confidence)
		AZOBR_p460088	Horizontally Transferred (medium confidence)
		AZOBR_p460089	Horizontally Transferred (low confidence)
		AZOBR_p460090	Horizontally Transferred (low confidence)
		AZOBR_p460091	Ancestral (low confidence)
		AZOBR_p460092	Ancestral (medium confidence)
		AZOBR_p460093	Horizontally Transferred (high confidence)
		AZOBR_p460094	Ancestral (low confidence)
		AZOBR_p460095	Ancestral (low confidence)
		AZOBR_p470001	Unassigned
		AZOBR_p470002	Unassigned
		AZOBR_p470003	Horizontally Transferred (low confidence)
		AZOBR_p470004	Horizontally Transferred (high confidence)
		AZOBR_p470005	Unassigned
		AZOBR_p470006	Horizontally Transferred (low confidence)
		AZOBR_p470007	Unassigned
		AZOBR_p470008	Horizontally Transferred (high confidence)
		AZOBR_p470009	Unassigned
		AZOBR_p470010	Unassigned
		AZOBR_p470011	Unassigned
		AZOBR_p470012	Horizontally Transferred (high confidence)
		AZOBR_p470013	Horizontally Transferred (high confidence)
		AZOBR_p470014	Horizontally Transferred (low confidence)
		AZOBR_p470015	Unassigned
		AZOBR_p470016	Horizontally Transferred (medium confidence)
		AZOBR_p470017	Ancestral (medium confidence)
		AZOBR_p470018	Ancestral (medium confidence)
		AZOBR_p470019	Ancestral (low confidence)
		AZOBR_p470020	Horizontally Transferred (high confidence)
		AZOBR_p470021	Ancestral (medium confidence)
		AZOBR_p470022	Unassigned
		AZOBR_p470023	Horizontally Transferred (high confidence)
		AZOBR_p470024	Ancestral (low confidence)
		AZOBR_p470025	Unassigned
		AZOBR_p470026	Unassigned
		AZOBR_p470027	Unassigned
		AZOBR_p470028	Horizontally Transferred (medium confidence)
		AZOBR_p470029	Unassigned
		AZOBR_p470030	Horizontally Transferred (high confidence)
		AZOBR_p470031	Ancestral (low confidence)
		AZOBR_p470032	Ancestral (low confidence)
		AZOBR_p470033	Unassigned
		AZOBR_p470034	Horizontally Transferred (high confidence)
		AZOBR_p470035	Horizontally Transferred (high confidence)
		AZOBR_p470036	Horizontally Transferred (low confidence)
		AZOBR_p470037	Horizontally Transferred (low confidence)
		AZOBR_p470038	Horizontally Transferred (high confidence)
		AZOBR_p470039	Ancestral (medium confidence)
		AZOBR_p470040	Ancestral (low confidence)
		AZOBR_p470041	Horizontally Transferred (low confidence)
		AZOBR_p470042	Horizontally Transferred (medium confidence)
		AZOBR_p470043	Ancestral (medium confidence)
		AZOBR_p470044	Unassigned
		AZOBR_p470045	Unassigned
		AZOBR_p470046	Unassigned
		AZOBR_p470047	Unassigned

		AZOBR_p470048	Ancestral (low confidence)
		AZOBR_p470049	Horizontally Transferred (high confidence)
		AZOBR_p470050	Horizontally Transferred (high confidence)
		AZOBR_p470051	Horizontally Transferred (high confidence)
		AZOBR_p470052	Ancestral (medium confidence)
		AZOBR_p470053	Horizontally Transferred (high confidence)
		AZOBR_p470054	Ancestral (medium confidence)
		AZOBR_p470055	Ancestral (high confidence)
		AZOBR_p470056	Ancestral (high confidence)
		AZOBR_p470057	Ancestral (high confidence)
		AZOBR_p470058	Ancestral (low confidence)
		AZOBR_p470059	Ancestral (high confidence)
		AZOBR_p470060	Ancestral (medium confidence)
		AZOBR_p470061	Ancestral (high confidence)
		AZOBR_p470062	Ancestral (high confidence)
		AZOBR_p470063	Horizontally Transferred (medium confidence)
		AZOBR_p470064	Horizontally Transferred (high confidence)
		AZOBR_p470065	Ancestral (low confidence)
		AZOBR_p470066	Unassigned
		AZOBR_p470067	Ancestral (low confidence)
		AZOBR_p470068	Ancestral (low confidence)
		AZOBR_p470069	Horizontally Transferred (high confidence)
		AZOBR_p470070	Horizontally Transferred (high confidence)
		AZOBR_p470071	Ancestral (medium confidence)
		AZOBR_p470072	Ancestral (high confidence)
		AZOBR_p470073	Ancestral (medium confidence)
		AZOBR_p470074	Ancestral (high confidence)
		AZOBR_p470075	Ancestral (high confidence)
		AZOBR_p470076	Ancestral (high confidence)
		AZOBR_p470077	Horizontally Transferred (high confidence)
		AZOBR_p470078	Ancestral (low confidence)
		AZOBR_p470079	Ancestral (medium confidence)
		AZOBR_p470080	Ancestral (medium confidence)
		AZOBR_p470081	Ancestral (high confidence)
		AZOBR_p470082	Horizontally Transferred (low confidence)
		AZOBR_p470083	Horizontally Transferred (medium confidence)
		AZOBR_p470084	Horizontally Transferred (high confidence)
		AZOBR_p470085	Horizontally Transferred (high confidence)
		AZOBR_p470086	Horizontally Transferred (medium confidence)
		AZOBR_p470087	Horizontally Transferred (medium confidence)
		AZOBR_p470088	Horizontally Transferred (medium confidence)
		AZOBR_p470089	Horizontally Transferred (medium confidence)
		AZOBR_p470090	Horizontally Transferred (high confidence)
		AZOBR_p470091	Horizontally Transferred (high confidence)
		AZOBR_p470092	Horizontally Transferred (high confidence)
		AZOBR_p470093	Horizontally Transferred (high confidence)
		AZOBR_p470094	Unassigned
		AZOBR_p470095	Unassigned
		AZOBR_p470096	Horizontally Transferred (high confidence)
		AZOBR_p470097	Horizontally Transferred (high confidence)
		AZOBR_p470098	Horizontally Transferred (high confidence)
		AZOBR_p470099	Unassigned
		AZOBR_p470100	Ancestral (medium confidence)
		AZOBR_p470101	Ancestral (low confidence)
		AZOBR_p480001	Unassigned
		AZOBR_p480002	Horizontally Transferred (high confidence)
		AZOBR_p480003	Unassigned
		AZOBR_p480004	Horizontally Transferred (high confidence)
		AZOBR_p480005	Horizontally Transferred (medium confidence)
		AZOBR_p480006	Horizontally Transferred (medium confidence)
		AZOBR_p480007	Horizontally Transferred (high confidence)
		AZOBR_p480008	Horizontally Transferred (high confidence)
		AZOBR_p480009	Horizontally Transferred (medium confidence)
		AZOBR_p480010	Horizontally Transferred (medium confidence)
		AZOBR_p480011	Horizontally Transferred (medium confidence)
		AZOBR_p480012	Horizontally Transferred (medium confidence)
		AZOBR_p480013	Horizontally Transferred (high confidence)
		AZOBR_p480014	Ancestral (low confidence)
		AZOBR_p480015	Ancestral (low confidence)
		AZOBR_p480016	Ancestral (low confidence)
		AZOBR_p480017	Ancestral (medium confidence)
		AZOBR_p480018	Horizontally Transferred (high confidence)
		AZOBR_p480019	Ancestral (low confidence)
		AZOBR_p480020	Unassigned
		AZOBR_p480021	Ancestral (low confidence)

		AZOBR_p480022	Unassigned
		AZOBR_p480023	Horizontally Transferred (high confidence)
		AZOBR_p480024	Horizontally Transferred (medium confidence)
		AZOBR_p480025	Ancestral (low confidence)
		AZOBR_p480026	Ancestral (medium confidence)
		AZOBR_p480027	Ancestral (medium confidence)
		AZOBR_p480028	Horizontally Transferred (high confidence)
		AZOBR_p480029	Horizontally Transferred (high confidence)
		AZOBR_p480030	Unassigned
		AZOBR_p480031	Horizontally Transferred (high confidence)
		AZOBR_p480032	Ancestral (low confidence)
		AZOBR_p480033	Horizontally Transferred (high confidence)
		AZOBR_p480034	Horizontally Transferred (high confidence)
		AZOBR_p480035	Horizontally Transferred (high confidence)
		AZOBR_p480036	Horizontally Transferred (high confidence)
		AZOBR_p480037	Unassigned
		AZOBR_p480038	Unassigned
		AZOBR_p480039	Horizontally Transferred (high confidence)
		AZOBR_p480040	Horizontally Transferred (high confidence)
		AZOBR_p480041	Ancestral (low confidence)
		AZOBR_p480042	Horizontally Transferred (high confidence)
		AZOBR_p480043	Unassigned
		AZOBR_p480044	Horizontally Transferred (high confidence)
		AZOBR_p480045	Horizontally Transferred (high confidence)
		AZOBR_p480046	Horizontally Transferred (high confidence)
		AZOBR_p480047	Horizontally Transferred (high confidence)
		AZOBR_p480048	Ancestral (low confidence)
		AZOBR_p480049	Ancestral (low confidence)
		AZOBR_p480050	Ancestral (low confidence)
		AZOBR_p480051	Ancestral (low confidence)
		AZOBR_p480052	Unassigned
		AZOBR_p480053	Horizontally Transferred (high confidence)
		AZOBR_p480054	Horizontally Transferred (medium confidence)
		AZOBR_p480055	Horizontally Transferred (low confidence)
		AZOBR_p480056	Horizontally Transferred (high confidence)
		AZOBR_p480057	Ancestral (low confidence)
		AZOBR_p480058	Unassigned
		AZOBR_p480059	Unassigned
		AZOBR_p480060	Horizontally Transferred (high confidence)
		AZOBR_p480061	Unassigned
		AZOBR_p480062	Horizontally Transferred (high confidence)
		AZOBR_p480063	Horizontally Transferred (high confidence)
		AZOBR_p480064	Horizontally Transferred (medium confidence)
		AZOBR_p480065	Ancestral (low confidence)
		AZOBR_p480066	Unassigned
		AZOBR_p480067	Unassigned
		AZOBR_p480068	Horizontally Transferred (high confidence)
		AZOBR_p480069	Unassigned
		AZOBR_p480070	Unassigned
		AZOBR_p480071	Ancestral (medium confidence)
		AZOBR_p480072	Unassigned
		AZOBR_p480073	Horizontally Transferred (high confidence)
		AZOBR_p480074	Horizontally Transferred (high confidence)
		AZOBR_p480075	Unassigned
		AZOBR_p480076	Unassigned
		AZOBR_p480077	Ancestral (medium confidence)
		AZOBR_p480078	Ancestral (low confidence)
		AZOBR_p480079	Unassigned
		AZOBR_p480080	Horizontally Transferred (high confidence)
		AZOBR_p480081	Horizontally Transferred (high confidence)
		AZOBR_p480082	Horizontally Transferred (high confidence)
		AZOBR_p480083	Horizontally Transferred (high confidence)
		AZOBR_p480084	Unassigned
		AZOBR_p480085	Unassigned
		AZOBR_p480086	Unassigned
		AZOBR_p480087	Unassigned
		AZOBR_p480088	Horizontally Transferred (medium confidence)
		AZOBR_p50001	Ancestral (low confidence)
		AZOBR_p50002	Ancestral (low confidence)
		AZOBR_p50003	Unassigned
		AZOBR_p50004	Ancestral (low confidence)
		AZOBR_p50005	Ancestral (low confidence)
		AZOBR_p50006	Horizontally Transferred (high confidence)
		AZOBR_p50007	Horizontally Transferred (high confidence)
		AZOBR_p50008	Ancestral (low confidence)

		AZOBR_p50009	Ancestral (medium confidence)
		AZOBR_p50010	Ancestral (low confidence)
		AZOBR_p50011	Ancestral (medium confidence)
		AZOBR_p50012	Horizontally Transferred (high confidence)
		AZOBR_p50013	Horizontally Transferred (high confidence)
		AZOBR_p50014	Horizontally Transferred (high confidence)
		AZOBR_p50015	Horizontally Transferred (high confidence)
		AZOBR_p50016	Ancestral (low confidence)
		AZOBR_p50017	Horizontally Transferred (high confidence)
		AZOBR_p50018	Ancestral (low confidence)
		AZOBR_p50019	Ancestral (medium confidence)
		AZOBR_p50020	Ancestral (high confidence)
		AZOBR_p50021	Horizontally Transferred (high confidence)
		AZOBR_p50022	Ancestral (low confidence)
		AZOBR_p50023	Horizontally Transferred (high confidence)
		AZOBR_p50024	Unassigned
		AZOBR_p50025	Horizontally Transferred (high confidence)
		AZOBR_p50026	Horizontally Transferred (high confidence)
		AZOBR_p50027	Horizontally Transferred (high confidence)
		AZOBR_p50028	Horizontally Transferred (medium confidence)
		AZOBR_p50029	Horizontally Transferred (high confidence)
		AZOBR_p50030	Horizontally Transferred (high confidence)
		AZOBR_p50031	Horizontally Transferred (high confidence)
		AZOBR_p50032	Ancestral (low confidence)
		AZOBR_p50033	Horizontally Transferred (medium confidence)
		AZOBR_p50034	Horizontally Transferred (medium confidence)
		AZOBR_p50035	Unassigned
		AZOBR_p50036	Horizontally Transferred (medium confidence)
		AZOBR_p50037	Horizontally Transferred (medium confidence)
		AZOBR_p50038	Unassigned
		AZOBR_p50039	Unassigned
		AZOBR_p50040	Unassigned
		AZOBR_p50041	Ancestral (medium confidence)
		AZOBR_p50042	Ancestral (low confidence)
		AZOBR_p50043	Ancestral (low confidence)
		AZOBR_p50044	Unassigned
		AZOBR_p50045	Unassigned
		AZOBR_p50046	Unassigned
		AZOBR_p50047	Unassigned
		AZOBR_p50048	Unassigned
		AZOBR_p50049	Unassigned
		AZOBR_p50050	Unassigned
		AZOBR_p50051	Unassigned
		AZOBR_p50052	Unassigned
		AZOBR_p50053	Unassigned
		AZOBR_p50054	Unassigned
		AZOBR_p50055	Horizontally Transferred (high confidence)
		AZOBR_p50056	Horizontally Transferred (high confidence)
		AZOBR_p50057	Unassigned
		AZOBR_p50058	Unassigned
		AZOBR_p50059	Horizontally Transferred (low confidence)
		AZOBR_p50060	Unassigned
		AZOBR_p50061	Unassigned
		AZOBR_p50062	Ancestral (medium confidence)
		AZOBR_p50063	Horizontally Transferred (low confidence)
		AZOBR_p50064	Ancestral (medium confidence)
		AZOBR_p50065	Horizontally Transferred (high confidence)
		AZOBR_p50066	Horizontally Transferred (high confidence)
		AZOBR_p50067	Horizontally Transferred (low confidence)
		AZOBR_p50068	Horizontally Transferred (low confidence)
		AZOBR_p50069	Horizontally Transferred (high confidence)
		AZOBR_p50070	Horizontally Transferred (high confidence)
		AZOBR_p50071	Horizontally Transferred (high confidence)
		AZOBR_p50072	Horizontally Transferred (high confidence)
		AZOBR_p50073	Horizontally Transferred (high confidence)
		AZOBR_p50074	Horizontally Transferred (high confidence)
		AZOBR_p50075	Horizontally Transferred (high confidence)
		AZOBR_p50076	Horizontally Transferred (high confidence)
		AZOBR_p50077	Horizontally Transferred (high confidence)
		AZOBR_p50078	Horizontally Transferred (high confidence)
		AZOBR_p50079	Horizontally Transferred (high confidence)
		AZOBR_p50080	Horizontally Transferred (medium confidence)
		AZOBR_p50081	Horizontally Transferred (medium confidence)
		AZOBR_p50082	Horizontally Transferred (high confidence)
		AZOBR_p50083	Horizontally Transferred (low confidence)

		AZOBR_p50085	Unassigned
		AZOBR_p50086	Unassigned
		AZOBR_p50089	Ancestral (low confidence)
		AZOBR_p50090	Horizontally Transferred (low confidence)
		AZOBR_p50091	Horizontally Transferred (low confidence)
		AZOBR_p50092	Ancestral (low confidence)
		AZOBR_p50093	Horizontally Transferred (medium confidence)
		AZOBR_p50094	Horizontally Transferred (low confidence)
		AZOBR_p50095	Ancestral (medium confidence)
		AZOBR_p50096	Unassigned
		AZOBR_p50097	Horizontally Transferred (high confidence)
		AZOBR_p50098	Unassigned
		AZOBR_p50099	Ancestral (low confidence)
		AZOBR_p50100	Horizontally Transferred (high confidence)
		AZOBR_p50101	Horizontally Transferred (high confidence)
		AZOBR_p50102	Unassigned
		AZOBR_p50103	Unassigned
		AZOBR_p50104	Unassigned
		AZOBR_p50105	Horizontally Transferred (high confidence)
		AZOBR_p50106	Horizontally Transferred (low confidence)
		AZOBR_p50107	Unassigned
		AZOBR_p50108	Horizontally Transferred (high confidence)
		AZOBR_p50109	Unassigned
		AZOBR_p50110	Horizontally Transferred (high confidence)
		AZOBR_p50111	Unassigned
		AZOBR_p50112	Unassigned
		AZOBR_p50113	Horizontally Transferred (high confidence)
		AZOBR_p50114	Ancestral (low confidence)
		AZOBR_p50115	Unassigned
		AZOBR_p50116	Unassigned
		AZOBR_p50117	Horizontally Transferred (medium confidence)
		AZOBR_p50118	Unassigned
		AZOBR_p50119	Unassigned
		AZOBR_p50120	Unassigned
		AZOBR_p50121	Unassigned
		AZOBR_p50122	Unassigned
		AZOBR_p50123	Horizontally Transferred (high confidence)
		AZOBR_p50124	Unassigned
		AZOBR_p50125	Ancestral (low confidence)
		AZOBR_p50126	Unassigned
		AZOBR_p50127	Ancestral (low confidence)
		AZOBR_p50128	Ancestral (low confidence)
		AZOBR_p50129	Unassigned
		AZOBR_p50130	Unassigned
		AZOBR_p50131	Horizontally Transferred (high confidence)
		AZOBR_p50132	Unassigned
		AZOBR_p50133	Horizontally Transferred (high confidence)
		AZOBR_p50134	Unassigned
		AZOBR_p50135	Unassigned
		AZOBR_p50136	Ancestral (low confidence)
		AZOBR_p50137	Horizontally Transferred (medium confidence)
		AZOBR_p50138	Horizontally Transferred (medium confidence)
		AZOBR_p50139	Unassigned
		AZOBR_p50140	Unassigned
		AZOBR_p50141	Unassigned
		AZOBR_p50142	Unassigned
		AZOBR_p50143	Horizontally Transferred (low confidence)
		AZOBR_p50144	Ancestral (low confidence)
		AZOBR_p50145	Ancestral (low confidence)
		AZOBR_p50146	Ancestral (low confidence)
		AZOBR_p50147	Unassigned
		AZOBR_p50148	Unassigned
		AZOBR_p50149	Unassigned
		AZOBR_p50150	Unassigned
		AZOBR_p50151	Unassigned
		AZOBR_p50152	Unassigned
		AZOBR_p50153	Horizontally Transferred (high confidence)
		AZOBR_p50154	Unassigned
		AZOBR_p50155	Horizontally Transferred (high confidence)
		AZOBR_p50156	Horizontally Transferred (high confidence)
		AZOBR_p50157	Horizontally Transferred (medium confidence)
		AZOBR_p50158	Ancestral (low confidence)
		AZOBR_p50159	Ancestral (low confidence)
		AZOBR_p50160	Horizontally Transferred (low confidence)
		AZOBR_p50161	Horizontally Transferred (high confidence)

		AZOBR_p50162	Horizontally Transferred (high confidence)
		AZOBR_p50163	Horizontally Transferred (high confidence)
		AZOBR_p50164	Horizontally Transferred (high confidence)
		AZOBR_p50165	Ancestral (high confidence)
		AZOBR_p50166	Unassigned
		AZOBR_p60001	Horizontally Transferred (high confidence)
		AZOBR_p60002	Horizontally Transferred (high confidence)
		AZOBR_p60003	Ancestral (low confidence)
		AZOBR_p60004	Horizontally Transferred (high confidence)
		AZOBR_p60005	Ancestral (medium confidence)
		AZOBR_p60006	Ancestral (low confidence)
		AZOBR_p60007	Ancestral (medium confidence)
		AZOBR_p60008	Horizontally Transferred (high confidence)
		AZOBR_p60009	Horizontally Transferred (high confidence)
		AZOBR_p60010	Horizontally Transferred (low confidence)
		AZOBR_p60011	Horizontally Transferred (low confidence)
		AZOBR_p60012	Ancestral (medium confidence)
		AZOBR_p60013	Horizontally Transferred (low confidence)
		AZOBR_p60014	Horizontally Transferred (high confidence)
		AZOBR_p60015	Horizontally Transferred (low confidence)
		AZOBR_p60016	Horizontally Transferred (high confidence)
		AZOBR_p60017	Horizontally Transferred (low confidence)
		AZOBR_p60018	Horizontally Transferred (high confidence)
		AZOBR_p60019	Horizontally Transferred (high confidence)
		AZOBR_p60020	Unassigned
		AZOBR_p60021	Horizontally Transferred (high confidence)
		AZOBR_p60022	Horizontally Transferred (high confidence)
		AZOBR_p60023	Unassigned
		AZOBR_p60024	Horizontally Transferred (medium confidence)
		AZOBR_p60025	Horizontally Transferred (high confidence)
		AZOBR_p60026	Ancestral (low confidence)
		AZOBR_p60027	Ancestral (low confidence)
		AZOBR_p60028	Ancestral (low confidence)
		AZOBR_p60029	Ancestral (low confidence)
		AZOBR_p60030	Ancestral (low confidence)
		AZOBR_p60031	Horizontally Transferred (low confidence)
		AZOBR_p60032	Ancestral (low confidence)
		AZOBR_p60033	Horizontally Transferred (high confidence)
		AZOBR_p60034	Horizontally Transferred (high confidence)
		AZOBR_p60035	Horizontally Transferred (high confidence)
		AZOBR_p60036	Ancestral (low confidence)
		AZOBR_p60037	Horizontally Transferred (high confidence)
		AZOBR_p60038	Ancestral (low confidence)
		AZOBR_p60039	Horizontally Transferred (high confidence)
		AZOBR_p60040	Unassigned
		AZOBR_p60041	Horizontally Transferred (high confidence)
		AZOBR_p60042	Horizontally Transferred (high confidence)
		AZOBR_p60043	Unassigned
		AZOBR_p60044	Ancestral (low confidence)
		AZOBR_p60045	Unassigned
		AZOBR_p60046	Horizontally Transferred (high confidence)
		AZOBR_p60047	Unassigned
		AZOBR_p60048	Ancestral (high confidence)
		AZOBR_p60049	Ancestral (low confidence)
		AZOBR_p60050	Ancestral (low confidence)
		AZOBR_p60051	Ancestral (low confidence)
		AZOBR_p60052	Ancestral (low confidence)
		AZOBR_p60053	Horizontally Transferred (low confidence)
		AZOBR_p60054	Horizontally Transferred (high confidence)
		AZOBR_p60055	Horizontally Transferred (high confidence)
		AZOBR_p60056	Horizontally Transferred (high confidence)
		AZOBR_p60057	Unassigned
		AZOBR_p60058	Ancestral (low confidence)
		AZOBR_p60059	Horizontally Transferred (high confidence)
		AZOBR_p60060	Horizontally Transferred (high confidence)
		AZOBR_p60061	Horizontally Transferred (high confidence)
		AZOBR_p60062	Horizontally Transferred (high confidence)
		AZOBR_p60063	Horizontally Transferred (high confidence)
		AZOBR_p60064	Unassigned
		AZOBR_p60065	Ancestral (medium confidence)
		AZOBR_p60066	Ancestral (medium confidence)
		AZOBR_p60067	Ancestral (medium confidence)
		AZOBR_p60068	Ancestral (high confidence)
		AZOBR_p60069	Ancestral (medium confidence)
		AZOBR_p60070	Horizontally Transferred (high confidence)

		AZOBR_p60071	Horizontally Transferred (low confidence)
		AZOBR_p60072	Ancestral (low confidence)
		AZOBR_p60073	Unassigned
		AZOBR_p60074	Unassigned
		AZOBR_p60075	Horizontally Transferred (low confidence)
		AZOBR_p60076	Horizontally Transferred (high confidence)
		AZOBR_p60077	Ancestral (low confidence)
		AZOBR_p60078	Ancestral (low confidence)
		AZOBR_p60079	Horizontally Transferred (low confidence)
		AZOBR_p60080	Ancestral (low confidence)
		AZOBR_p60081	Horizontally Transferred (high confidence)
		AZOBR_p60082	Ancestral (low confidence)
		AZOBR_p60083	Horizontally Transferred (medium confidence)
		AZOBR_p60084	Ancestral (low confidence)
		AZOBR_p60085	Horizontally Transferred (low confidence)
		AZOBR_p60086	Horizontally Transferred (low confidence)
		AZOBR_p60087	Horizontally Transferred (low confidence)
		AZOBR_p60088	Horizontally Transferred (low confidence)
		AZOBR_p60089	Horizontally Transferred (low confidence)
		AZOBR_p60090	Horizontally Transferred (low confidence)
		AZOBR_p60091	Ancestral (low confidence)
		AZOBR_p60092	Horizontally Transferred (high confidence)
		AZOBR_p60093	Unassigned
		AZOBR_p60094	Horizontally Transferred (medium confidence)
		AZOBR_p60095	Unassigned
		AZOBR_p60096	Horizontally Transferred (high confidence)
		AZOBR_p60097	Horizontally Transferred (high confidence)
		AZOBR_p60098	Unassigned
		AZOBR_p60099	Unassigned
		AZOBR_p60100	Unassigned
		AZOBR_p60101	Unassigned
		AZOBR_p60102	Unassigned
		AZOBR_p60103	Unassigned
		AZOBR_p60104	Unassigned
		AZOBR_p60105	Unassigned
		AZOBR_p60106	Unassigned
		AZOBR_p60107	Horizontally Transferred (high confidence)
		AZOBR_p60108	Unassigned
		AZOBR_p60109	Horizontally Transferred (low confidence)
		AZOBR_p60110	Ancestral (high confidence)
		AZOBR_p60111	Ancestral (high confidence)
		AZOBR_p60112	Horizontally Transferred (high confidence)
		AZOBR_p60113	Unassigned
		AZOBR_p60114	Unassigned
		AZOBR_p60115	Unassigned
		AZOBR_p60116	Unassigned
		AZOBR_p60117	Unassigned
		AZOBR_p60118	Unassigned
		AZOBR_p60119	Horizontally Transferred (high confidence)
		AZOBR_p60120	Ancestral (low confidence)
		AZOBR_p60121	Unassigned
		AZOBR_p60122	Horizontally Transferred (high confidence)
		AZOBR_p60123	Horizontally Transferred (high confidence)
		AZOBR_p60124	Horizontally Transferred (low confidence)
		AZOBR_p60125	Horizontally Transferred (low confidence)

Ancestry assignments for all the predicted proteins in *A. lipoferum* 4B and *A. brasilense* Sp245, identified by their locus tags, were produced using the scheme described in Figure S3 and Materials and Methods.
