# Supplementary material for: Azospirillum Genomes Reveal Transition of Bacteria from Aquatic to Terrestrial Environments
Source: PLoS Genet. 2011 Dec 22;7(12):e1002430. doi: 10.1371/journal.pgen.1002430 (PMC3245306; doi:10.1371/journal.pgen.1002430)
Supplement: Table S7 — Genes that are potentially involved in adaptation of Azospirillum to the rhizosphere and its interaction with host plants. (PDF) [file pgen.1002430.s010.pdf]

Supplementary Table 7. Genes that are potentially involved in adaptation of *Azospirillum* to the rhizosphere and its interaction with host plants.

Role in	Gene name	Product	4B	B510	Sp245	Ancestry*
**Alkaline pH adaptation** [1]						
	phaAB	pH adaptation potassium efflux system transmembrane protein	AZOLI_p20321	AZL_c01200	_	hor H
	phaC	pH adaptation potassium efflux system transmembrane protein	AZOLI_p20322	AZL_c01210	AZOBR_p1100017	hor H
	phaD	pH adaptation potassium efflux system transmembrane protein	AZOLI_p20323	AZL_c01220	AZOBR_p1100016	hor H
	phaE	pH adaptation potassium efflux system transmembrane protein	AZOLI_p20324	AZL_c01230	AZOBR_p1100015	hor H
	phaF	pH adaptation potassium efflux system protein	AZOLI_p20325	AZL_c01240	AZOBR_p1100014	hor H
	phaG	pH adaptation potassium efflux system protein	AZOLI_p20326	AZL_c01250	AZOBR_p1100013	hor H
**Aromatic compound transport and metabolism** [2]						
	catA	catechol 1,2 monooxygenase (EC 1.13.11.1)	AZOLI_p11003	AZL_a06810	_	hor H
	catC	muconolactone delta-isomerase (EC 5.3.3.4)	AZOLI_p11002	AZL_a06820	_	hor H
	catB	muconate cycloisomerase (EC 5.5.1.1)	AZOLI_p11001	AZL_a06830	_	hor H
	catI	3-oxoadipate:succinyl-CoA transferase, A subunit (EC 2.8.3.6)	AZOLI_p40024	AZL_d00530	_	hor H
	catJ	3-oxoadipate:succinyl-CoA transferase, B subunit (EC 2.8.3.6)	AZOLI_p40025	AZL_d00520	_	hor H
	pcaF	beta-ketoadipyl CoA thiolase (EC 2.3.1.16)	AZOLI_p40026	AZL_d00510	_	hor H
	catD	3-oxoadipate enol-lactonase II (EC 3.1.1.24)	AZOLI_p40027	AZL_d00500	_	anc L
		3-oxoadipate enol-lactonase II (EC 3.1.1.24)	_	_	AZOBR_70204	hor L
	pcaC	4-carboxymuconolactone decarboxylase (EC 4.1.1.44)	AZOLI_p40028	AZL_d00490	_	unassigned,unassigned
	pcaB	3-carboxy-cis,cis-muconate cycloisomerase (EC 5.5.1.2)	AZOLI_p40029	AZL_d00480	_	hor H
	pcaH	protocatechuate 3,4-dioxygenase beta chain (EC 1.13.11.3)	AZOLI_p40030	AZL_d00470	_	anc L
	pcaG	protocatechuate 3,4-dioxygenase alpha chain (EC 1.13.11.3)	AZOLI_p40031	AZL_d00460	_	anc L
	_	hydroxyquinol 1,2-dioxygenase (EC 1.13.11.37)	AZOLI_p10976	AZL_a08930	_	anc L
	_	maleylacetate reductase (EC 1.3.1.32)	AZOLI_p10977	AZL_a08920	_	hor H
	vanB	vanillate O-demethylase oxidoreductase (EC 1.14.13.-)	AZOLI_p10989	AZL_a08830	_	hor H
	pobA/ phbH	4-hydroxybenzoate-3-monooxygenase (EC 1.14.13.2)	AZOLI_p40609	AZL_d01000	_	hor H
	ligB	protocatechuate 4,5-dioxygenase, beta chain (EC 1.13.11.8)	_	_	AZOBR_p310188	anc L
	ligA	protocatechuate 4,5-dioxygenase, alpha chain (EC 1.13.11.8)	_	_	AZOBR_p310189	unassigned
	ligI	2-pyrone-4,6-dicarboxylic acid hydrolase (EC 3.1.1.57)	_	_	AZOBR_p310190	anc L
	ligK	4-carboxy-4-hydroxy-2-oxoadipate aldolase (EC 4.1.3.17)	AZOLI_p20168	_	AZOBR_p310192	hor M
	ligC	4-carboxy-2-hydroxymuconate-6-semialdehyde dehydrogenase (EC	_	_	AZOBR_p310194	anc L

	1.2.1.45)				
_	muconate cycloisomerase (EC 5.5.1.1)	AZOLI_p40287	AZL_d02920	AZOBR_p170014	hor H
gtdA	gentisate 1,2-dioxygenase (EC 1.13.11.4)	AZOLI_p20646	AZL_a09180	_	hor H
nagL	maleylpyruvate isomerase (EC 5.2.1.4)	AZOLI_p20652	AZL_a09240	_	anc M,anc L
nagK	fumarylpyruvate hydrolase (EC 3.7.1.5)	AZOLI_p20653	AZL_a09250	_	hor H
_	putative fumarylpyruvate hydrolase (EC 3.7.1.5)	AZOLI_0049	AZL_026390	AZOBR_10442	hor M,hor M,hor H
hmgA	homogentisate 1,2-dioxygenase (EC 1.13.11.5)	AZOLI_p40081	AZL_d04870	_	hor H
maiA	maleylacetoacetate isomerase (EC 5.2.1.2)	AZOLI_2553	AZL_024560	_	anc L
_	putative fumarylacetoacetate hydrolase (EC 3.7.1.2)	AZOLI_2565	AZL_024660	_	anc L,hor H
paaK	phenylacetyl-CoA ligase (EC 6.2.1.30)	AZOLI_p40257	AZL_d01530	_	anc M
paaJ	beta-ketoadipyl CoA thiolase (EC 2.3.1.-)	AZOLI_p40258	_	_	hor M
paaI	phenylacetate degradation protein with thioesterase domain	AZOLI_p40259	AZL_d01540	_	anc M
paaH	3-hydroxybutyryl-CoA dehydrogenase (EC 1.1.1.-)	AZOLI_p40260	AZL_d01550	_	anc L
paaG	enoyl-CoA hydratase, phenylacetate catabolism (EC 4.2.1.17)	AZOLI_p40261	AZL_d01560	_	anc L
paaN	aldehyde dehydrogenase, phenylacetate catabolism (EC 1.2.1.-)	AZOLI_p40262	AZL_d01570	_	hor H
paaE	phenylacetate-CoA oxygenase, PaaE subunit	AZOLI_p40263	AZL_d01580	_	hor H
paaD	phenylacetate-CoA oxygenase, PaaD subunit	AZOLI_p40264	AZL_d01590	_	hor H
paaC	phenylacetate-CoA oxygenase, PaaC subunit	AZOLI_p40265	AZL_d01600	_	hor H
paaB	phenylacetate-CoA oxygenase, PaaB subunit	AZOLI_p40266	AZL_d01610	_	hor H
paaA	phenylacetate-CoA oxygenase, PaaA subunit	AZOLI_p40267	AZL_d01620	_	hor L
_	aromatic-ring-hydroxylating dioxygenase, alpha subunit	AZOLI_p10986	AZL_a08860	_	hor L
_	aromatic-ring-hydroxylating dioxygenase, beta subunit	AZOLI_p10987	AZL_a08850	_	hor L
_	aromatic-ring-hydroxylating dioxygenase, ferredoxin subunit	AZOLI_p20647	AZL_a09190	_	hor H
_	aromatic-ring-hydroxylating dioxygenase, beta subunit	AZOLI_p20648	AZL_a09200	_	hor H
_	aromatic-ring-hydroxylating dioxygenase, alpha subunit	AZOLI_p20649	AZL_a09210	_	hor H
_	aromatic-ring-hydroxylated dioxygenase, FAD reductase subunit	AZOLI_p20650	AZL_a09220	_	hor H
_	hydroxybenzoate transporter	_	AZL_b03660	_	anc L
_	protocatechuate transporter	_	_	AZOBR_p310195	anc L
pcaK	hydroxybenzoate transporter	AZOLI_p20645	AZL_a09170	_	hor H

Attachment [3]

flp/fap	component of TAD pili	_	_	AZOBR_p460077	anc L
flp/fap	component of TAD pili	_	AZL_001250	_	anc L
cpaA	component of TAD pili	_	AZL_001260		hor H
cpaA	component of TAD pili	_	_	AZOBR_p460078	hor L
cpaB	component of TAD pili	_	AZL_001270	_	hor M
cpaB	component of TAD pili	_	_	AZOBR_p460079	hor L

cpaC	component of TAD pili	_	AZL_001280	AZOBR_p460080	hor H,hor M
cpaD	component of TAD pili	_	AZL_001290	_	hor H
cpaD	component of TAD pili	_	_	AZOBR_p460081	unassigned
cpaE	component of TAD pili	_	AZL_001300	_	hor M
cpaE	component of TAD pili	_	_	AZOBR_p460082	hor L
cpaF	component of TAD pili	_	AZL_001310	_	anc L
cpaF	component of TAD pili	_	_	AZOBR_p460084	anc L
tadB	component of TAD pili	_	AZL_001320	_	unassigned
tadB	component of TAD pili	_	_	AZOBR_p460085	hor L
tadC	component of TAD pili	_	AZL_001330	_	hor M
tadC	component of TAD pili	_	_	AZOBR_p460087	hor L
tadD	component of TAD pili	_	_	AZOBR_p460088	hor M
tadE	component of TAD pili	_	AZL_001340	AZOBR_p460089	hor L,hor H
tadF	component of TAD pili	_	AZL_001350	AZOBR_p460090	hor L,hor H
tadG	component of TAD pili	_	AZL_001360	_	anc L
tadG	component of TAD pili	_	_	AZOBR_p460091	anc L
celA	cellulose synthase	AZOLI_p30427	AZL_c05160	_	unassigned
celB	cellulose synthase	AZOLI_p30428	AZL_c05170	_	hor H
_	poly(beta-D-mannuronate) O-acetylase (alginate biosynthesis)	AZOLI_0390	AZL_005370	AZOBR_170002	hor H
kdsA	3-deoxy-D-manno-octulosonic acid 8-phosphate synthetase	AZOLI_1226	AZL_013740	AZOBR_100318	anc M,anc L,anc M
_	UDP-glucose/glucuronate epimerase	AZOLI_2265	AZL_021990	AZOBR_140024	anc H
_	UDP-galactopyranose mutase	AZOLI_p30013	_	AZOBR_p1100104	hor H
galU	UTP-glucose-1-phosphate uridylyltransferase	AZOLI_p40163	AZL_d00310	AZOBR_p440128	anc M
glmM	phosphoglucosamine mutase	AZOLI_p50061	AZL_e01250	AZOBR_100216	anc H
rfbE	CDP-tyvelose-2-epimerase	AZOLI_p50348	_	AZOBR_p210161	hor M
cpsB	mannose-6-phosphate isomerase/mannose-1-phosphate guanyltransferase	AZOLI_p60182	AZL_f01870	_	anc L,anc M
rfbB	dTDP-D-glucose 4,6-dehydratase	AZOLI_p60197	AZL_f01790	_	hor H
_	glucose-1-phosphate cytidylyltransferase	_	AZL_f01360	_	hor H
_	UDP-glucose/GDP-mannose dehydrogenase	_	_	AZOBR_p230003	hor H
_	dTDP-4,deoxyrhamnose 3,5 epimerase (fragment)	_	_	AZOBR_p60004	hor H
rfbB	dTDP-D-glucose 4,6-dehydratase	_	_	AZOBR_p60005	anc M
_	UDP-glucose/GDP-mannose dehydrogenase	_	_	AZOBR_p60035	hor H
manC	mannose-1-phosphate guanyltransferase	_	_	AZOBR_p60091	anc L
**Carbohydrate and alcohol transport and metabolism** [4]					
araB	ribulo-/ribitol kinase (EC 2.7.1.16)	AZOLI_p20644	AZL_b05880	_	hor L
_	L-arabinose 1-dehydrogenase (EC 1.1.1.46)	AZOLI_p50383	AZL_e03690	AZOBR_p440166	hor H

_	L-arabinonate dehydratase (EC 4.2.1.25)	AZOLI_p50384	AZL_e03700	AZOBR_p440167	hor H
_	putative L-arbinonolactonase (EC 3.1.1.15)	AZOLI_p50378	AZL_e03640	AZOBR_p440161	anc L
araD	2-dehydro-3-deoxy-L-arabinonate dehydratase (EC 4.2.1.43)	AZOLI_p50377	AZL_e03630	AZOBR_p440160	hor H
_	2-ketoglutarate semialdehyde dehydrogenase (EC 1.2.1.26)	AZOLI_p50376	AZL_e02770	AZOBR_p180015	hor H
adhE2	aldehyde-alcohol dehydrogenase (EC 1.2.1.10 / 1.1.1.1)	AZOLI_p10253	AZL_a08020	AZOBR_p470098	hor H
adhE1	aldehyde-alcohol dehydrogenase (EC 1.2.1.10 / 1.1.1.1)	AZOLI_p20688	_	_	hor H
_	alcohol dehydrogenase, zinc-binding (EC 1.1.1.1)	AZOLI_p30374	AZL_c00300	_	hor H
_	alcohol dehydrogenase, zinc-binding (EC 1.1.1.1)	AZOLI_p10391	AZL_a07970	_	hor H
_	alcohol dehydrogenase, zinc-binding (EC 1.1.1.1)	_	_	AZOBR_190004	hor H
_	alcohol dehydrogenase, iron-type (EC 1.1.1.1)	_	AZL_b03380	AZOBR_p330019	hor H
fruK	fructose-1-phosphate kinase (EC 2.7.1.56)	AZOLI_p30558	AZL_c02620	_	anc L
galM1	aldose 1-epimerase (EC 5.1.3.3)	AZOLI_p10528	AZL_a09030	AZOBR_p440169	hor H
galM2	aldose 1-epimerase (EC 5.1.3.3)	AZOLI_p50370	AZL_e03560	_	hor H
_	putative galactokinase (EC 2.7.1.6)	AZOLI_p60015	_	_	anc L
_	putative galactokinase (EC 2.7.1.6)	_	AZL_f00470	_	anc L
galE1	UDP-glucose 4-epimerase (EC 5.1.3.2)	AZOLI_p60194	AZL_f01830	AZOBR_p60033	hor H
galE2	UDP-glucose 4-epimerase (EC 5.1.3.2)	AZOLI_p20384	AZL_a07190	AZOBR_p210032	hor L
_	putative β-phosphoglucomutase (EC 5.4.2.6)	AZOLI_p30301	_	AZOBR_p470037	hor L
dgoK	2-keto-3-deoxy-galactonokinase (EC 2.7.1.58)	AZOLI_p30272	AZL_c04390	AZOBR_p210119	unassigned
dgoA	2-dehydro-3-deoxy-6-phosphogalactonate aldolase (EC 4.1.2.21)	AZOLI_p30273	AZL_c04400	AZOBR_p210117	hor M
uxaC	glucuronate isomerase (EC 5.3.1.12)	AZOLI_p40500	AZL_d01370	AZOBR_p460005	hor H
uxaB	altronate oxidoreductase (EC 1.1.1.58)	AZOLI_p30296	AZL_c04510	AZOBR_p440170	hor H
uxaA	altronate hydrolase (EC 4.2.1.7)	AZOLI_p40501	AZL_d01360	AZOBR_p460006	anc L,anc L,unassigned
kdgK	ketodeoxygluconokinase (EC 2.7.1.45)	AZOLI_p40503	AZL_d01340	AZOBR_p410051	hor H
kduI1	5-keto 4-deoxyuronate isomerase (EC 5.1.3.17)	AZOLI_p20544	_	_	hor H
kduI2	5-keto 4-deoxyuronate isomerase (EC 5.1.3.17)	AZOLI_p40492	AZL_d01450	AZOBR_p420029	hor H
kduD1	2-deoxy-D-gluconate 3-dehydrogenase (EC 1.1.1.125)	AZOLI_p20543	_	AZOBR_p420028	hor H
kduD2	2-deoxy-D-gluconate 3-dehydrogenase (EC 1.1.1.125)	AZOLI_p40493	AZL_d01440	AZOBR_p450010	hor H
uxuA1	mannonate hydrolyase (EC 4.2.1.8)	AZOLI_p20264	AZL_a08380	_	hor H
uxuA2	mannonate hydrolyase (EC 4.2.1.8)	AZOLI_p50109	AZL_e02160	AZOBR_p450004	hor H
uxuB1	mannonate oxidoreductase (EC 1.1.1.57)	AZOLI_p20265	AZL_a08370	_	unassigned
uxuB2	mannonate oxidoreductase (EC 1.1.1.57)	AZOLI_p50104	AZL_e02210	AZOBR_p450005	anc L
gntK	gluconate kinase 2 (EC 2.7.1.12)	AZOLI_p40040	AZL_d00380	_	hor H
_	putative gluconate kinase 2 (EC 2.7.1.12)	_	_	AZOBR_p130030	hor H
_	putative 6-phosphogluconate dehydrogenase (EC 1.1.1.44)	AZOLI_p10235	AZL_a04610	AZOBR_p310081	unassigned
eda	2-dehydro-3-deoxy-6-phosphogalactonate aldolase (EC 4.1.2.14)	AZOLI_p50154	AZL_e04020	_	hor H

edd	6-phosphogluconate dehydratase (EC 4.2.1.12)	AZOLI_p30595	AZL_a09920	_	hor M,hor H
pgi	glucose-6-phosphate isomerase (EC 5.3.1.9)	AZOLI_0602	AZL_007010	AZOBR_70008	anc L
pfkA	6-phosphofructokinase (EC 2.7.1.11)	AZOLI_1428	AZL_017430	AZOBR_100017	anc M
glpX	fructose-1,6-bisphosphatase (EC 3.1.3.11)	AZOLI_1545	AZL_010940	AZOBR_100390	anc M
fbaB	fructose-bisphosphate aldolase, class I (EC 4.1.2.13)	AZOLI_0810	AZL_009650	AZOBR_150016	anc M
tpiA	triosephosphate isomerase (EC 5.3.1.1)	AZOLI_1222	AZL_013680	AZOBR_100314	anc M,anc H
gapB1	glyceraldehyde 3-phosphate dehydrogenase (EC 1.2.1.12)	AZOLI_0815	AZL_009710	AZOBR_150011	anc L
gapB2	glyceraldehyde 3-phosphate dehydrogenase (EC 1.2.1.12)	AZOLI_p20464	_	_	anc L
pgk	phosphoglycerate kinase (EC 2.7.2.3)	AZOLI_2630	AZL_025100	AZOBR_40277	anc H
_	phosphoglycerate kinase fragment (EC 2.7.2.3)	AZOLI_p20463	AZL_b03050	_	anc H
gpmA	phosphoglycerate mutase (EC 5.4.2.1)	AZOLI_p10567	AZL_a06650	AZOBR_p1120003	anc L
gpmI	phosphoglycerate mutase (EC 5.4.2.1)	AZOLI_2417	AZL_023340	AZOBR_40314	anc M,anc M,anc H
eno1	enolase (EC 4.2.1.11)	AZOLI_p10268	AZL_a02700	AZOBR_100325	anc H
eno2	enolase (EC 4.2.1.11)	AZOLI_p20459	AZL_b03090	AZOBR_p420024	anc H
pykA1	pyruvate kinase (EC 2.7.1.40)	AZOLI_p10291	AZL_a03300	AZOBR_110012	anc H
pykA2	pyruvate kinase (EC 2.7.1.40)	AZOLI_p10527	AZL_a09040	AZOBR_p420013	anc H
pykA3	pyruvate kinase (EC 2.7.1.40)	AZOLI_p40106	AZL_d05100	AZOBR_p1160003	unassigned,hor M, unassigned
glk	glucokinase (2.7.1.2)	AZOLI_0164	AZL_002850	AZOBR_70092	anc H
gcd	quinoprotein glucose dehydrogenase (EC 1.1.5.2)	AZOLI_p50302	AZL_e1560	_	hor H
_	Gluconolactonase (EC 3.1.1.17)	_	AZL_a09340	_	hor H
_	Gluconate 2-dehydrogenase subunit (EC 1.1.99.3), cytochrome C	AZOLI_p20466	_	_	hor H
_	Gluconate 2-dehydrogenase subunit (EC 1.1.99.3), FAD binding	AZOLI_p20467	_	_	hor M
_	Gluconate 2-dehydrogenase subunit (EC 1.1.99.3)	AZOLI_p20468	_	_	hor M
_	Gluconate 2-dehydrogenase flavoprotein (EC 1.1.99.3)	AZOLI_p10738	AZL_c02930	AZOBR_p460054	hor H
glpK	Glycerol kinase (EC 2.7.1.30)	AZOLI_p50190	AZL_e03010	AZOBR_p280095	hor L
glpD	sn-glycerol-3-phosphate dehydrogenase (EC 1.1.5.3)	AZOLI_p50197	AZL_e03080	_	hor L
glpD	sn-glycerol-3-phosphate dehydrogenase (EC 1.1.5.3)	_	_	AZOBR_p280087	hor L
gldA	Glycerol dehydrogenase (EC 1.1.1.6)	_	_	AZOBR_100391	hor H
iolB	5-deoxy-glucuronate isomerase (EC 5.3.1.-)	_	AZL_b00950	_	hor H
iolG	myo-inositol 2-dehydrogenase (EC 1.1.1.18)	_	AZL_b00980	_	anc L
iolA	methylmalonate-semialdehyde dehydrogenase (EC 1.2.1.27)	_	AZL_b01030	_	hor L
_	methylmalonate-semialdehyde dehydrogenase (EC 1.2.1.27)	AZOLI_p10852	AZL_a07240	AZOBRv2_p210063	anc L
iolE	Inosose dehydratase (EC 4.2.1.44)	_	AZL_b01040	_	unassigned
iolB	Epi-inositol hydrolase (EC 3.7.1.-)	_	AZL_b01050	_	anc L
iolC	5-dehydro-2-deoxygluconokinase (EC 2.7.1.92)	_	AZL_b01060	_	hor L

rsbK	ribokinase (EC 2.7.1.15)	AZOLI_p20179	_	_	hor L
_	ribokinase (EC 2.7.1.15)	_	AZL_b03490	_	hor L
deoK	deoxyribokinase/ribokinase (EC 2.7.1.15)	AZOLI_p20643	AZL_b05870	_	anc L
deoM	deoxyribose mutarotase	AZOLI_p20642	AZL_b05860	_	anc L
glgP	glycogen phosphorylase (EC 2.4.1.1)	AZOLI_1134	AZL_014210	AZOBR_p110080	anc H
_	amylo-α-1,6-glucosidase (EC 3.2.1.33)	AZOLI_0954	_	AZOBR_180051	unassigned,unassigned
xylA	D-xylose isomerase (EC 5.3.1.5)	AZOLI_p20701	AZL_b00070	AZOBR_p330086	hor H
xylB	xylulose kinase (EC 2.7.1.17)	AZOLI_p20702	AZL_b00080	_	hor H
_	xylulose kinase (EC 2.7.1.17)	_	_	AZOBR_p330085	hor H
fruB1	PEP-dependent fructose phosphotransferase system, EI/HPr/EIIA components	AZOLI_p30557	AZL_c02630	AZOBR_p480015	anc L
_	sugar ABC transporter, ATP-binding component	AZOLI_0562	AZL_006710	AZOBR_10003	unassigned,anc L,anc L
_	sugar ABC transporter, permease component	AZOLI_0560	AZL_006690	AZOBR_10005	anc L
_	sugar ABC transporter, permease component	AZOLI_0561	AZL_006700	AZOBR_10004	hor H
_	sugar ABC transporter, substrate-binding component	AZOLI_0558	AZL_006680	AZOBR_10006	hor H
kdgT	2-keto-3-deoxy-D-gluconate transporter	AZOLI_p20545	_	AZOBR_p420031	hor H
xylF	D-xylose ABC transporter, periplasmic solute-binding component	AZOLI_p20700	AZL_b00060	_	hor H
xylH	D-xylose ABC transporter, permease component	AZOLI_p20699	AZL_b00050	_	hor H
fruA	fructose phosphotransferase system, IIB/IIC components	AZOLI_p30559	AZL_c02610	AZOBR_p480016	anc L
ptsG	fused glucose-specific PTS enzymes: IIB and IIC components	AZOLI_p20577	AZL_b05000	_	hor H
exuT	hexuronate transporter	AZOLI_p30300	AZL_c04530	AZOBR_p230048	hor H
fruB2	PEP-dependent fructose phosphotransferase system, EI/HPr/EIIA components	AZOLI_p20578	AZL_b04990	_	hor H
_	polysaccharide ABC transporter, ATP-binding component	AZOLI_p60200	_	_	anc L
_	polysaccharide ABC type transporter, permease component	AZOLI_p60201	_	_	hor H
nagE	PTS system, N-acetylglucosamine-specific IIBC component	AZOLI_p20570	AZL_b05060	_	hor H
_	putative glycerol ABC transporter, ATP-binding component	_	_	AZOBR_p280089	hor H
_	putative glycerol ABC transporter, ATP-binding component	_	_	AZOBR_p280088	hor H
_	putative H+/gluconate symporter	AZOLI_p40131	AZL_d00120	_	hor H
_	putative periplasmic component of ABC superfamily	AZOLI_p50230	AZL_e03720	_	anc L
_	putative sugar ABC transporter, ATP-binding component	AZOLI_p50195	AZL_e03060	_	hor H
_	putative sugar ABC transporter, ATP-binding component	AZOLI_p50196	AZL_e03070	_	hor H
_	putative sugar ABC transporter, ATP-binding component	_	_	AZOBR_p330099	hor H
_	putative sugar ABC transporter, ATP-binding component	_	_	AZOBR_p180019	hor H
_	putative sugar ABC transporter, ATP-binding component	_	_	AZOBR_p280021	anc L
_	putative sugar ABC transporter, periplasmic component	AZOLI_p20638	AZL_b05820	_	anc L
_	putative sugar ABC transporter, periplasmic component	AZOLI_p20715	AZL_a02410	_	hor H

_	putative sugar ABC transporter, periplasmic component	AZOLI_p50191	AZL_e03020	AZOBR_p280093	hor H
_	putative sugar ABC transporter, periplasmic component	_	_	AZOBR_p330102	hor H
_	putative sugar ABC transporter, periplasmic component	_	_	AZOBR_p180016	hor H
_	putative sugar ABC transporter, periplasmic component	_	_	AZOBR_p280018	anc L
_	putative sugar ABC transporter, permease component	AZOLI_p50193	AZL_e03040	AZOBR_p280091	hor H
_	putative sugar ABC transporter, permease component	AZOLI_p50194	AZL_e03050	AZOBR_p280090	hor H
_	putative sugar ABC transporter, permease component	_	_	AZOBR_p330100	hor H
_	putative sugar ABC transporter, permease component	_	_	AZOBR_p330101	hor H
_	putative sugar ABC transporter, permease component	_	_	AZOBR_p180017	hor H
_	putative sugar ABC transporter, permease component	_	_	AZOBR_p180018	hor H
_	putative sugar ABC transporter, permease component	_	_	AZOBR_p280019	anc L
_	putative sugar ABC transporter, permease component	_	_	AZOBR_p280020	anc L
_	putative sugar transporter of the major facilitator superfamily	AZOLI_p50398	AZL_e00070	_	hor H
_	putative sugar transporter, MFS family	AZOLI_p50231	AZL_e03730	_	hor H
_	putative TRAP-type mannitol/chloroaromatic compound transporter, periplasmic component	AZOLI_p30383	AZL_c00200	AZOBR_p1110126	anc M
rbsA1	ribose ABC transporter, ATP-binding component	AZOLI_p20585	_	_	hor H
rbsA2	ribose ABC transporter, ATP-binding component	AZOLI_p20714	AZL_a02400	_	hor H
rbsB	ribose ABC transporter, periplasmic component	AZOLI_p20587	_	_	hor H
rbsB	ribose ABC transporter, periplasmic component	_	_	AZOBR_p330089	hor H
rbsC1	ribose ABC transporter, permease component	AZOLI_p20586	_	_	hor H
rbsC	ribose ABC transporter, permease component	_	_	AZOBR_p330090	hor H
rbsC2	ribose ABC transporter, permease component	AZOLI_p20713	AZL_a02390	_	hor H
gguA	sugar ABC transporter, ATP-binding component	AZOLI_p50381	AZL_e03670	AZOBR_p440164	hor H
_	sugar ABC transporter, ATP-binding component (RbsA-like)	AZOLI_p50373	AZL_e03590	AZOBR_p440157	hor H
xylG	sugar ABC transporter, ATP-binding protein	AZOLI_p20698	AZL_b00040	_	hor H
_	sugar ABC transporter, fused ATP-binding components (RbsA-like)	AZOLI_p20639	AZL_b05830	_	anc L
_	sugar ABC transporter, periplasmic component	AZOLI_p50374	AZL_e03600	AZOBR_p440158	hor H
sbpA	sugar ABC transporter, periplasmic component	AZOLI_p50380	AZL_e03660	AZOBR_p440163	hor H
gguB	sugar ABC transporter, permease component	AZOLI_p50382	AZL_e03680	AZOBR_p440165	hor H
_	sugar ABC transporter, permease component (RbsC-like)	AZOLI_p20640	AZL_b05840	_	anc L
_	sugar ABC transporter, permease component (RbsC-like)	AZOLI_p20641	AZL_b05850	_	anc L
_	sugar ABC transporter, permease component (RbsC-like)	AZOLI_p50371	AZL_e03570	AZOBR_p440155	hor H
_	sugar ABC transporter, permease component (RbsC-like)	AZOLI_p50372	AZL_e03580	AZOBR_p440156	hor H
_	sugar permease of the major facilitator superfamily	AZOLI_p50335	AZL_e01340	AZOBR_p220019	hor H
**Exopolysaccharide and lipopolysaccharide production** [5]					
_	glycosyltransferase	AZOLI_0037	AZL_026460	AZOBR_200027	anc H

_	glycosyltransferase	AZOLI_0056	AZL_026330	AZOBR_10419	anc M
_	putative glycosyltransferase	AZOLI_0247	AZL_003980	AZOBR_40145	hor H
kdsB	3-deoxy-manno-octulosonate cytidylyltransferase	AZOLI_0295	AZL_004340	AZOBR_40271	anc M,anc H,anc M
_	putative polysaccharide export protein	AZOLI_0422	AZL_005620	AZOBR_160033	anc M
_	glycosyltransferase	AZOLI_0524	AZL_006390	AZOBR_180018	anc H
kdtA	3-deoxy-D-manno-octulosonic-acid transferase	AZOLI_0529	AZL_006410	AZOBR_180020	anc H,anc M,anc H
lpxK	tetraacyldisaccharide 4'-kinase	AZOLI_0530	AZL_006420	AZOBR_180021	anc H
_	lipid A biosynthesis acyltransferase	AZOLI_0531	AZL_006430	AZOBR_180022	anc H
lspA	Lipoprotein signal peptidase	AZOLI_0608	AZL_007050	AZOBR_70013	anc H
_	putative glycosyltransferase	AZOLI_0610	AZL_007090	AZOBR_p1120031	hor H
_	putative galactosyltransferase	AZOLI_0613	AZL_007120	AZOBR_p1120034	hor H
_	glycosyltransferase family 2	AZOLI_0696	AZL_008870	AZOBR_200183	anc L
exbD	biopolymer transport protein	AZOLI_0830	AZL_009820	AZOBR_140015	anc M
_	putative glycosyltransferase, group 1	AZOLI_0943	AZL_f00040	AZOBR_p60058	anc L,anc M,anc L
_	putative Polysaccharide deacetylase	AZOLI_0959	AZL_010660	AZOBR_180046	anc M
lpxA	UDP-N-acetylglucosamine acyltransferase	AZOLI_1081	AZL_016150	AZOBR_100286	anc H
lpxB	lipid-A-disaccharide synthase	AZOLI_1083	AZL_016130	AZOBR_100288	anc H
_	protein of unknown function; putative Glycosyltransferase domain	AZOLI_1127	_	_	anc L
_	glycosyltransferase	AZOLI_1204	AZL_013210	AZOBR_110049	anc L
_	putative Lipopolysaccharide core biosynthesis glycosyltransferase	AZOLI_1206	AZL_013230	AZOBR_110051	anc L
_	Putative glycosyltransferase, group 2	AZOLI_1208	AZL_013250	AZOBR_110053	anc L
_	glycosyltransferase	AZOLI_1257	AZL_015840	AZOBR_150118	hor H
_	putative glycosyltransferase	AZOLI_1443	AZL_017540	_	unassigned
_	glycosyltransferase family 2	AZOLI_1475	AZL_012160	AZOBR_140212	anc M
_	putative glycosyltransferase	AZOLI_1517	AZL_011160	AZOBR_p1140099	hor H
_	putative glycosyltransferase	AZOLI_1877	AZL_019300	AZOBR_100092	unassigned,hor M,anc M
lpxC	UDP-3-O-acyl N-acetylglucosamine deacetylase	AZOLI_2140	AZL_020890	AZOBR_180110	anc H
pssA	phosphatidylserine synthase	AZOLI_2161	AZL_021060	AZOBR_150144	anc H
_	Putative phospho-N-acetylmuramoyl-pentapeptide-transferase	AZOLI_2266	AZL_022170	_	anc H
_	glycosyltransferase	AZOLI_2267	AZL_022180	_	hor H
_	glycosyltransferase	AZOLI_2268	AZL_022190	_	anc L
_	Putative sugar nucleotide epimerase/dehydratase	AZOLI_2269	AZL_022200	AZOBR_140021	unassigned
_	Putative sugar nucleotide epimerase/dehydratase	AZOLI_2270	AZL_022210	AZOBR_140020	anc H
rfaD	ADP-L-glycero-D-mannoheptose-6-epimerase	AZOLI_2285	AZL_022310	AZOBR_p440146	anc H
_	putative aminotransferase, StrS family	AZOLI_2320	AZL_022560	AZOBR_70138	hor H
_	glycosyltransferase	AZOLI_2370	AZL_023000	AZOBR_40343	anc M

lpxD	UDP-3-O-(3-hydroxymyristoyl) glucosamine N-acyltransferase	AZOLI_2520	AZL_024330	AZOBR_40170	anc L
rfaE	fused heptose 7-phosphate kinase; heptose 1-phosphate adenyltransferase	AZOLI_2623	AZL_025050	AZOBR_40288	anc H
kpsF	arabinose 5-phosphate isomerase	AZOLI_2876	AZL_001050	AZOBR_p1110041	anc M
_	glycosyl transferase	AZOLI_3150	AZL_a11300	AZOBR_p460041	hor H
_	glycosyltransferase	AZOLI_p10100	AZL_a00380	_	anc M
_	putative glycosyltransferase, group 1	AZOLI_p10211	_	_	hor H
glmS	Glucosamine-fructose-6-phosphate aminotransferase	AZOLI_p10413	AZL_a07270	AZOBR_140158	anc H
_	putative glycosyltransferase, group 1	AZOLI_p10554	AZL_a06960	AZOBR_p1110156	hor H
_	putative glycosyltransferase, group 1	AZOLI_p10555	AZL_a06950	AZOBR_p1110157	hor H
_	putative glycosyltransferase, family 2	AZOLI_p10556	AZL_a06940	AZOBR_p1110158	hor H
_	putative O-antigen polymerase	AZOLI_p10557	AZL_a06930	AZOBR_p1110159	hor H
_	Undecaprenyl-phosphate galactose phosphotransferase exoY-like	AZOLI_p10558	AZL_a06920	AZOBR_p1110160	hor H
_	putative glycosyltransferase, WecB/TagA/CpsF family	AZOLI_p10565	AZL_a06670	AZOBR_p1110168	hor H
_	putative 3-deoxy-manno-octulosonate cytidylyltransferase	AZOLI_p10577	AZL_a06590	AZOBR_p1160038	anc M
_	putative glycosyltransferase	AZOLI_p10604	AZL_a05520	AZOBR_p140053	unassigned
_	putative glycosyltransferase, group 2	AZOLI_p10670	_	AZOBR_150003	anc L
_	glycosyl transferase	AZOLI_p10818	AZL_a04300	AZOBR_p1180007	hor M,unassigned,hor H
_	putative glycosyltransferase, group 1 (fragment)	AZOLI_p20132	_	_	unassigned
_	Putative glycosyltransferase, group 2	AZOLI_p20391	AZL_a07140	AZOBR_p210041	hor H
wgeD	glycosyltransferase, group 1	AZOLI_p20393	AZL_a07120	AZOBR_p210044	hor H
_	putative glycosyltransferase, group 1	AZOLI_p20395	AZL_a07100	AZOBR_p210046	hor H
_	putative glycosyltransferase, group 1	AZOLI_p30014	_	_	hor H
_	glycosyltransferase	AZOLI_p30192	AZL_c00920	AZOBR_p1120074	hor H
_	glycosyltransferase, family 9	AZOLI_p30434	AZL_c05270	_	anc H
_	putative Glycosyl transferase, group 2	AZOLI_p30536	_	_	hor H
_	glycosyltransferase, group 1	AZOLI_p30542	AZL_c02730	_	hor H,hor M
_	glycosyltransferase, group 1	AZOLI_p30544	AZL_c02710	_	unassigned
_	putative glycosyl transferase, group 1	AZOLI_p30545	AZL_c02700	_	hor H
_	glycosyltransferase	AZOLI_p30547	AZL_c02680	_	unassigned,hor H
_	glycosyltransferase, group 1	AZOLI_p30548	AZL_c02670	_	unassigned
_	putative UDP-Glycosyltransferase/glycogen phosphorylase	AZOLI_p40090	AZL_d04940	_	anc L,unassigned
_	glycosyltransferase	AZOLI_p40091	AZL_d04950	_	unassigned
_	glycosyltransferase, group 1	AZOLI_p40092	AZL_d04960	_	unassigned
_	putative glycosyltransferase	AZOLI_p40094	AZL_d04980	_	unassigned
rfaC	heptosyltransferase I	AZOLI_p40151	AZL_d00260	AZOBR_100043	anc H
_	putative Lipid A core-O-antigen ligase	AZOLI_p40152	AZL_d00270	AZOBR_100042	anc H

rkpK1	UDP-glucose 6-dehydrogenase	AZOLI_p40164	AZL_d00320	AZOBR_p60068	anc H
exoC	phosphomannomutase	AZOLI_p40165	AZL_d00330	AZOBR_p60069	anc M
exoY	undecaprenyl-phosphate galactose phosphotransferase	AZOLI_p40338	AZL_d02010	AZOBR_p1130130	anc L
_	putative oligosaccharide repeat unit transporter	AZOLI_p40383	AZL_d02540	AZOBR_40159	hor H
_	UDP-glucose 6-dehydrogenase	AZOLI_p40385	AZL_d02530	AZOBR_40158	unassigned
_	nucleoside-diphosphate-sugar epimerase	AZOLI_p40386	AZL_d02520	AZOBR_40157	hor H
wcaG	GDP-L-fucose synthetase	AZOLI_p40387	AZL_d02510	AZOBR_40156	hor H
_	glycosyl transferase	AZOLI_p40388	AZL_d02500	AZOBR_40155	unassigned,hor M,hor M
_	glycosyltransferase	AZOLI_p40389	AZL_d02490	AZOBR_40154	hor H
_	glycosyltransferase	AZOLI_p40390	AZL_d02480	AZOBR_40152	hor H
_	glycosyltransferase	AZOLI_p50171	AZL_e02810	AZOBR_110068	anc L
_	putative glycosyltransferase, family 2	AZOLI_p50173	AZL_e02830	AZOBR_110066	unassigned,unassigned,anc L
_	putative glycosyltransferase, group 1	AZOLI_p50346	_	AZOBR_p340024	hor M
_	putative glycosyltransferase	AZOLI_p60005	AZL_f01540	AZOBR_p170003	anc L
fcl1	GDP-L-fucose synthetase	AZOLI_p60007	AZL_f01640	AZOBR_p60012	anc M
fcl2	GDP-L-fucose synthetase	AZOLI_p60017	_	_	unassigned
gmhA	phosphoheptose isomerase	AZOLI_p60053	AZL_f01620	AZOBR_p60112	hor M,unassigned,hor H
_	putative glycosyltransferase	AZOLI_p60056	_	_	unassigned
_	putative glycosyltransferase	AZOLI_p60057	AZL_f01670	_	hor H
_	UDP-N-acetylglucosamine 2-epimerase	AZOLI_p60088	_	_	anc L
rtbF	glucose-1-phosphate cytidylyltransferase	AZOLI_p60139	_	_	hor H
_	glycosyltransferase	AZOLI_p60158	AZL_f00160	AZOBR_p60034	hor H
_	glycosyltransferase	AZOLI_p60174	_	_	anc M
rkpK2	UDP-glucose 6-dehydrogenase	AZOLI_p60193	_	_	anc M
rfbA	glucose-1-phosphate thymidylyltransferase	AZOLI_p60195	AZL_f01810	_	hor H
rfbD	dTDP-4-dehydrorhamnose reductase	AZOLI_p60196	AZL_f01800	AZOBR_p60006	anc L,anc L,unassigned
rfbC	dTDP-4,deoxyrhamnose 3,5 epimerase	AZOLI_p60198	AZL_f01780	_	anc L,anc M
_	putative glycosyltransferase, family 2	AZOLI_p60199	_	_	anc L
_	NDP-N-acetyl-D-galactosaminuronic acid dehydrogenase	AZOLI_p60211	AZL_f01240	_	hor H
_	glycosyltransferase	AZOLI_p60238	AZL_f01100	_	hor H
_	glycosyltransferase, family 2	AZOLI_p60242	_	_	anc L
gmd1	GDP-mannose 4,6-dehydratase	AZOLI_p60265	AZL_f01050	_	hor M,hor H
gmd2	GDP-mannose 4,6-dehydratase (fragment)	AZOLI_p60266	_	_	anc L
_	glycosyltransferase	_	AZL_d02410	_	anc L
_	glycosyltransferase	_	AZL_e00440	_	anc L
_	glycosyltransferase, group 1	_	AZL_e03440	_	hor H

_	glycosyltransferase, group 1	_	AZL_e03450	_	anc L
fcl	GDP-L-fucose synthase	_	AZL_f00440	_	hor H
gmd	GDP-mannose 4,6-dehydratase	_	AZL_f01060	_	anc L
_	putative glycosyltransferase	_	_	AZOBR_100002	anc L
_	glycosyltransferase, group 1	_	_	AZOBR_100004	anc L
_	putative glycosyltransferase	_	_	AZOBR_10433	anc L
_	putative glycosyltransferase	_	_	AZOBR_10436	anc L
_	glycosyltransferase, group 1	_	_	AZOBR_10437	anc L
_	glycosyltransferase, group 1	_	_	AZOBR_10439	anc L
_	putative glycosyltransferase, group 4	_	_	AZOBR_140022	anc H
_	putative glycosyltransferase, group 1	_	_	AZOBR_140023	anc L
_	putative glycosyltransferase, group 2	_	_	AZOBR_140059	hor H
_	putative glycosyltransferase, group 2	_	_	AZOBR_40077	hor H
_	putative glycosyltransferase, group 2	_	_	AZOBR_40146	hor H
_	glycosyltransferase, group 2 (fragment)	_	_	AZOBR_70072	unassigned
_	glycosyltransferase protein	_	_	AZOBR_70089	anc L
_	putative glycosyltransferase, WecB/TagA/CpsF family (fragment)	_	_	AZOBR_p1120001	hor H
_	putative glycosyltransferase, group 1	_	_	AZOBR_p170001	anc M
_	putative glycosyltransferase	_	_	AZOBR_p170061	hor M
_	glycosyltransferase, group 1	_	_	AZOBR_p210162	anc L
_	glycosyltransferase, group 1 (fragment)	_	_	AZOBR_p210174	hor H
_	putative glycosyltransferase	_	_	AZOBR_p210176	hor H
exoP	Succinoglycan biosynthesis transport protein ExoP	_	_	AZOBR_p230002	anc M
_	glycosyltransferase	_	_	AZOBR_p230009	hor H
_	glycosyltransferase, group 2	_	_	AZOBR_p310019	hor H
_	glycosyltransferase, group 2	_	_	AZOBR_p310022	hor H
_	glycosyltransferase	_	_	AZOBR_p310280	anc L
_	glycosyltransferase, family 51	_	_	AZOBR_p440063	hor H
gmd	GDP-mannose 4,6-dehydratase	_	_	AZOBR_p60001	hor H
_	putative glycosyltransferase	_	_	AZOBR_p60002	hor H
_	putative glycosyltransferase	_	_	AZOBR_p60003	anc L
rfbA	glucose-1-phosphate thymidylyltransferase	_	_	AZOBR_p60007	anc M
_	putative glycosyltranferase	_	_	AZOBR_p60013	hor L
wbpY	glycosyltransferase, group 1	_	_	AZOBR_p60014	hor H
gmd	GDP-D-mannose dehydratase, NAD(P)-binding, colanic acid synthesis	_	_	AZOBR_p60015	hor L
_	putative glycosyltransferase, group 2	_	_	AZOBR_p60021	hor H

_	putative glycosyltransferase, group 1	_	_	AZOBR_p60029	anc L
_	putative glycosyltranferase	_	_	AZOBR_p60031	hor L
_	putative glycosyl transferase, group 2 (fragment)	_	_	AZOBR_p60039	hor H
_	putative glycosyltranferase	_	_	AZOBR_p60049	anc L
_	putative glycosyltranferase	_	_	AZOBR_p60053	hor L
_	putative glycosyltranferase	_	_	AZOBR_p60082	anc L
_	putative glycosyltranferase	_	_	AZOBR_p60084	anc L
_	putative glycosyltransferase, group 1	_	_	AZOBR_p60089	hor L
_	glycosyltransferase, group 1	_	_	AZOBR_p60090	hor L
_	putative glycosyltransferase, group 1	_	_	AZOBR_p60096	hor H
_	putative glycosyltransferase, group 1	_	_	AZOBR_p60097	hor H
_	putative glycosyltransferase, group 1	_	_	AZOBR_p60109	hor L
rfaD	ADP-L-glycero-D-mannoheptose-6-epimerase	_	_	AZOBR_p60110	anc H
rfaE	bifunctional protein RfaE (Involved in ADP-L-glycero-D-manno-heptose synthesis)	_	_	AZOBR_p60111	anc H
_	glycosyltransferase, group 1	_	_	AZOBR_p60119	hor H
_	glycosyltransferase, group 2	_	_	AZOBR_p60120	anc L
_	putative glycosyltransferase, group 1	_	_	AZOBR_p60125	hor L

Drug resistance [6]

ermE	multidrug transporter, SMR superfamily	AZOLI_0213	AZL_025680	AZOBR_200157	anc L
pecM	permease of the drug/metabolite transporter (DMT) superfamily	AZOLI_0927	AZL_010530	_	hor H
_	putative multidrug-efflux transporter, MFS family	AZOLI_0988	AZL_010850	AZOBR_150101	hor H
_	multidrug efflux system, subunit A (AcrB family) (fragment)	_	_	AZOBR_p110066	unassigned
mdtA2	multidrug efflux system, subunit A (AcrB family)	AZOLI_1033	AZL_017630	_	anc L
mdtB2	multidrug efflux system, subunit B (AcrB family)	AZOLI_1034	AZL_017620	AZOBR_p110065	anc L
sugE	multidrug transporter, SMR superfamily	AZOLI_1465	AZL_012220	AZOBR_140035	hor M,hor M,hor H
_	multidrug efflux transporter (NolG-like)	AZOLI_1522	AZL_011100	AZOBR_p110062	hor H
_	putative cation/multidrug efflux transporter, RND family	AZOLI_2099	_	AZOBR_p50027	hor H
_	putative efflux transporter, RND family	AZOLI_2100	_	AZOBR_p50026	hor H
_	putative multidrug efflux transporter	AZOLI_2102	_	AZOBR_p50024	unassigned
_	multidrug efflux transporter, MSF family	AZOLI_2749	AZL_002340	AZOBR_40226	hor H
_	multidrug resistance efflux pump (ErmK-like)	AZOLI_p10652	AZL_a05380	AZOBR_p1130047	anc M
_	multidrug resistance efflux pump (EmrB-like)	AZOLI_p10653	AZL_a05390	AZOBR_p1130049	anc M,anc M,anc H
_	multidrug efflux transporter	AZOLI_p10814	AZL_a04330	AZOBR_p1180011	anc L,anc L,anc M
_	putative RND efflux transporter, MFP subunit	AZOLI_p10815	AZL_a04320	AZOBR_p1180010	anc L
_	RND efflux transporter, AcrB-like component	AZOLI_p10826	_	_	anc L
_	RND efflux transporter, MFP subunit	AZOLI_p10827	_	AZOBR_p310207	anc L

acrA2	multidrug efflux transporter, AcrA component	AZOLI_p20171	_	_	hor H
acrB2	multidrug efflux transporter, AcrB component	AZOLI_p20172	_	_	hor H
oprM	multidrug efflux transporter, OprM component	AZOLI_p20173	_	_	anc L
_	putative multidrug resistance membrane protein (MFP family)	AZOLI_p30050	AZL_a10641	AZOBR_p1120069	hor H
_	putative efflux transporter permease; fusaric acid resistance pump	AZOLI_p30051	AZL_a10650	AZOBR_p1120070	hor H
_	putative multidrug efflux transporter, RND family, MFP subunit	AZOLI_p30309	AZL_b06260	AZOBR_p330150	hor H
_	putative multidrug efflux transporter, acrB/acrD/acrF family	AZOLI_p30310	AZL_b06270	AZOBR_p330151	hor H
_	putative multiple antibiotic resistance protein (MarC-like)	AZOLI_p30514	AZL_c04720	AZOBR_p280101	hor H
mdtC	multidrug efflux system, subunit C (AcrB family)	AZOLI_p40176	AZL_d04480	_	hor H,hor M
mdtB1	multidrug efflux system, subunit B (AcrB family)	AZOLI_p40177	AZL_d04470	_	anc L
mdtA1	multidrug efflux system, subunit A (AcrB family)	AZOLI_p40178	AZL_d04460	_	anc L
_	multiple antibiotic resistance protein (MarC-like)	AZOLI_p40256	AZL_d01520	AZOBR_p470041	hor L
_	putative tetracycline-efflux transporter	AZOLI_p50089	AZL_e02360	_	hor M,hor H
_	drug resistance transporter, Bcr/CflA subfamily	AZOLI_p50221	AZL_e02530	_	hor H
acrB1	multidrug efflux transporter, AcrB component	AZOLI_p50393	AZL_e00110	AZOBR_p420021	anc L,anc M,anc L
acrA1	multidrug efflux transporter, AcrA component	AZOLI_p50394	AZL_e00100	AZOBR_p420020	anc M
_	putative RND efflux transporter, MFP subunit	AZOLI_p50426	AZL_d02690	AZOBR_p440178	hor H
mexB	multidrug efflux transporter (RND family)	AZOLI_p50427	AZL_d02700	AZOBR_p440179	hor H

Glycoside hydrolase activity [7]

_	4-alpha-glucanotransferase; (glyco_hydro_77)	AZOLI_p10625	AZL_a05620	AZOBR_p140015	anc L,anc M,anc L
malQ	4-α-glucanotransferase (EC 2.4.1.25)	AZOLI_p10107	AZL_a00430	AZOBR_140112	anc M
_	alpha-amylase	AZOLI_1942	_	AZOBR_40417	hor H
_	alpha-amylase	AZOLI_p10102	AZL_a00400	AZOBR_140107	anc L,anc M,anc M
_	alpha-amylase	AZOLI_p10624	AZL_a05610	AZOBR_p140016	unassigned
_	alpha-amylase	AZOLI_p30209	AZL_c02820	AZOBR_100146	anc L
_	alpha-amylase	_	_	AZOBR_190016	hor H
_	alpha-amylase	_	_	AZOBR_p280055	hor H
_	alpha-amylase	_	_	AZOBR_p310298	hor H
melA	alpha-galactosidase (melibiase) (EC 3.2.1.22)	AZOLI_p50318	AZL_e01400	AZOBR_p280123	anc M
_	alpha-glucosidase (glyco_hydro_31)	_	AZL_e03400	_	hor H
_	alpha-glucosidase (glyco_hydro_31)	_	_	AZOBR_p310247	hor H
_	cellulase, endogluconase (glyco_hydro_5)	AZOLI_p10561	AZL_a06890	AZOBR_p1110164	hor H
_	cellulase, endogluconase (glyco_hydro_5)	_	_	AZOBR_p470008	hor H
_	cellulase, endogluconase (glyco_hydro_5)	AZOLI_p40099	AZL_d05040	_	hor H
_	cellulase, endogluconase (glyco_hydro_5)	_	_	AZOBR_150049	hor H
_	cellulase, endogluconase (glyco_hydro_8)	AZOLI_p30425	AZL_c05150	_	anc L,unassigned

_	endoxylanase (glyco_hydro_10)	AZOLI_p10563	AZL_a06690	AZOBR_p1110166	hor H
_	endoxylanase (glyco_hydro_10)	_	_	AZOBR_p310014	hor H
_	glucoamylase (glyco_hydro_15)	_	_	AZOBR_p210150	hor M
glgX1	glycogen debranching enzyme (EC 3.2.1-)	AZOLI_p10105	AZL_a00410	AZOBR_140110	anc L
glgX2	glycogen debranching enzyme (EC 3.2.1-)	AZOLI_p30385	AZL_c00180	AZOBR_p1120007	unassigned,anc L,anc L
_	hypothetical glycoside hydrolase family 63	_	_	AZOBR_p310299	hor H
_	Licheninase (glyco_hydro_16)	AZOLI_p10648	AZL_a05340	_	hor H
_	Lysozyme (glyco_hydro_25)	AZOLI_p40527	AZL_d03580	_	anc L
treZ	malto-oligosyltrehalose trehalohydrolase (EC 3.2.1.141)	AZOLI_0953	_	AZOBR_180052	anc L
treS	trehalose synthase	AZOLI_p10101	AZL_a00390	AZOBR_140106	anc M
_	unknown endogluconase (glyco_hydro_12)	_	_	AZOBR_p440082	hor H
_	α-glucosidase (EC 3.2.1.20)	AZOLI_p10598	AZL_a06410	AZOBR_p110052	anc L
_	β- N-acetylhexosaminidase (glyco_hydro_3)	AZOLI_p10769	AZL_a04490	_	anc M,anc L
_	β- N-acetylhexosaminidase (glyco_hydro_3)	_	_	AZOBR_p120114	anc L
_	β-(1-6) glucans synthase (glyco_hydro_17)	AZOLI_p40646	AZL_d00810	AZOBR_10535	anc M
_	β-glucosidase /cellobiase	AZOLI_p50295	AZL_e01630	AZOBR_p140078	anc M
_	β-glucosidase /cellobiase	AZOLI_p50317	AZL_e01410	AZOBR_p280124	anc M
_	β-glucosidase /cellobiase (glyco_hydro_1)	AZOLI_p30407	AZL_c00120	AZOBR_p280134	anc M
_	β-glucosidase /cellobiase (glyco_hydro_1)	AZOLI_p40351	AZL_d02120	AZOBR_p280136	anc M
_	β-glucosidase-related glycosidase (glyco_hydro_3)	_	AZL_c03570	_	anc L
_	β-glucuronidase	_	_	AZOBR_p310253	hor H
_	β-glucuronidase (glyco_hydro_2)	_	_	AZOBR_p310252	hor H
_	β-mannosidase (glyco_hydro_2)	AZOLI_p50309	AZL_e01490	AZOBR_p270141	hor H
**Hormone synthesis** [8]					
ipdC	indole pyruvate decarboxylase	_	_	AZOBR_40354	anc L
nthA	nitrile hydratase (conversion of indole-3-acetonitrile to indole-3-acetamide)	_	_	AZOBR_p310181	unassigned
nthB	nitrile hydratase	_	_	AZOBR_p310180	hor M
_	indole acetic acid transacetylase	AZOLI_1094	AZL_016030	AZOBR_100302	anc L, unassigned,unassigned
acdS	ACC deaminase	AZOLI_p20559	AZL_b04170	_	hor H
acdR	Lrp regulator of ACC deaminase	AZOLI_p20560	AZL_b04180	_	hor H
nirK	copper-containing nitrite reductase	AZOLI_p30082	AZL_c02030	AZOBR_p310167	hor H
nahG	salicylate 1-monooxygenase (EC 1.14.13.1)	AZOLI_p20435	_	_	hor M
_	putative salicylate 1-monooxygenase (EC 1.14.13.1)	_	_	AZOBR_p480008	hor H
**Methylamine degradation** [9]					
mgsA	N-methyl-glutamate (NMG) dehydrogenase	_	AZL_a09510	_	hor M

mgsB	N-methyl-glutamate (NMG) dehydrogenase	_	AZL_a09520	_	anc L
mgsC	N-methyl-glutamate (NMG) dehydrogenase	_	AZL_a09530	_	hor H
gmas	γ-glutamylmethylamide (GMA) synthetase	_	AZL_a09540	_	anc L
mgdA	N-methyl-glutamate (NMG) synthase	_	AZL_a09550	_	unassigned
mgdB	N-methyl-glutamate (NMG) synthase	_	AZL_a09560	_	anc L
mgdC	N-methyl-glutamate (NMG) synthase	_	AZL_a09570	_	hor H
mgdD	N-methyl-glutamate (NMG) synthase	_	AZL_a09580	_	anc L
**Nitrogen fixation and H2 recycling** [10, 11]					
fixA	electron transfer flavoprotein beta subunit	AZOLI_0535	AZL_006470	AZOBR_70108	anc M
fixB	electron transfer flavoprotein alpha chain	AZOLI_0536	AZL_006480	AZOBR_70107	anc M
fixC	flavoprotein-ubiquinone oxidoreductase	AZOLI_0537	AZL_006490	AZOBR_70106	unassigned,anc M,anc M
fixD	4Fe-4S ferredoxin	AZOLI_0538	AZL_006500	AZOBR_70105	unassigned
nifW	nitrogenase-stabilizing/protective protein	AZOLI_0541	AZL_006520	AZOBR_70109	hor M
nifP1	serine acetyltransferase	AZOLI_0542	AZL_006530	AZOBR_70110	hor H
nifV	homocitrate synthase	AZOLI_0543	AZL_006540	AZOBR_70111	anc H
nifS2	cysteine desulfurase	AZOLI_0544	AZL_006550	AZOBR_70112	hor L
nifU	Fe-S cluster assembly protein	AZOLI_0545	AZL_006560	AZOBR_70113	hor M
nifQ2	nitrogenase iron-molybdenum cofactor biosynthesis protein	_	_	AZOBR_70116	anc L
fdxB	ferredoxin III (*nif* specific)	AZOLI_0669	AZL_007610	AZOBR_70117	hor H,hor H,anc L
nifX	nitrogenase molybdenum-iron protein	AZOLI_0672	AZL_007640	AZOBR_70120	hor H
nifN	nitrogenase reductase-associated ferredoxin	AZOLI_0673	AZL_007650	AZOBR_70121	hor H
nifE	nitrogenase molybdenum-cofactor biosynthesis protein	AZOLI_0674	AZL_007660	AZOBR_70122	hor H
nifK	nitrogenase molybdenum-iron protein beta chain	AZOLI_0679	AZL_007690	AZOBR_70126	anc L,anc L,unassigned
nifD	nitrogenase molybdenum-iron protein alpha chain	AZOLI_0680	AZL_007700	AZOBR_70127	hor L,hor L,hor M
nifH	nitrogenase iron protein, dinitrogenase reductase	AZOLI_0681	AZL_007710	AZOBR_70128	unassigned
draT	ADP-ribosyl-[dinitrogenase reductase]transferase	AZOLI_0684	AZL_007730	AZOBR_70132	anc H
draG	ADP-ribosyl-[dinitrogenase reductase]hydrolase	AZOLI_0685	AZL_007740	AZOBR_70133	anc H
ntrB	sensor histidine kinase involved in nitrogen fixation	AZOLI_1342	AZL_015080	AZOBR_p110009	anc H
ntrC	sensor histidine kinase involved in nitrogen fixation	AZOLI_1343	AZL_015070	AZOBR_p110010	anc H
ntrY	transcriptional regulator involved in nitrogen fixation	AZOLI_1344	AZL_015060	AZOBR_p110011	anc H
glnB	nitrogen regulatory protein P-II 2	AZOLI_1865	AZL_019210	AZOBR_100230	anc H
glnA	glutamine synthase	AZOLI_1867	AZL_019220	AZOBR_100228	anc H
glnD	[Protein-P-II]urydyltransferase	AZOLI_1713	AZL_017160	AZOBR_10466	anc H
nifA	Nif-specific transcriptional regulator	AZOLI_2308	AZL_022450	AZOBR_70150	anc M
nifB	nitrogenase iron-molybdenum cofactor biosynthesis protein	AZOLI_2311	AZL_022470	AZOBR_70147	hor H
fdxN	ferredoxin	AZOLI_2312	AZL_022480	AZOBR_70146	hor M,hor M,anc L

nifZ1	nitrogenase P-cluster assembly	AZOLI_2315	AZL_022510	AZOBR_70143	anc M
nifZ2	nitrogenase P-cluster assembly	AZOLI_2316	AZL_022520	AZOBR_70142	hor H
nifS1	cysteine desulfurase	AZOLI_2317	AZL_022530	_	hor H
_	fragment of cysteine desulfurylase	_	_	AZOBR_70141	hor H
nifT	NifT/FixU nitrogen fixation protein	AZOLI_2318	AZL_022540	_	anc M,anc L
nifQ1	nitrogenase iron-molybdenum cofactor synthesis protein	AZOLI_0980	AZL_010780	AZOBR_09008	anc H
nifP2	serine acetyltransferase	AZOLI_1974	AZL_020070	AZOBR_70185	anc H
fixK	nitrogen fixation regulation protein, Crp/FNR family	AZOLI_2692	AZL_003420	AZOBR_10251	anc H
fixS	cytochrome ccb3 oxidase maturation protein	AZOLI_2693	AZL_003410	AZOBR_10252	anc M
fixI	cation-exporting ATPase	AZOLI_2694	AZL_003400	AZOBR_10253	anc H
fixH	integral membrane protein linked to a cation pump	AZOLI_2695	AZL_003390	AZOBR_10254	anc H
fixG	4Fe-4S ferredoxin, iron-sulfur binding	AZOLI_1705	AZL_016980	AZOBR_10255	anc H
cytP	cytochrome C oxidase, CytP subunit	AZOLI_2697	AZL_003380	AZOBR_10256	anc M
cytQ	cytochrome c oxidase CytQ subunit	AZOLI_2698	AZL_003370	AZOBR_10257	hor H
cytO	cytochrome C oxidase, CytO subunit	AZOLI_2699	AZL_003360	AZOBR_10258	anc M,anc M,anc L
cytN	cytochrome C oxidase, CytN subunit	AZOLI_2700	AZL_003350	AZOBR_10259	anc H
rpoN (ntrA)	RNA polymerase sigma-54 factor	AZOLI_2872	AZL_001090	AZOBR_p1110045	anc H
gltD	glutamate synthase, beta subunit	AZOLI_2889	AZL_001000	AZOBR_p1110035	anc M
gltB	glutamate synthase, alpha subunit	AZOLI_2890	AZL_000990	AZOBR_p1110033	anc H
glnZ	nitrogen regulatory protein P-II 1	AZOLI_p40634	AZL_d00720	AZOBR_10503	anc H
vnfE	nitrogenase cofactor synthesis protein	_	_	AZOBR_p350011	hor H
vnfN	nitrogenase cofactor synthesis protein	_	_	AZOBR_p350012	hor H
vnfX	vanadium nitrogenase protein	_	_	AZOBR_p350013	hor H
vnfA	vanadium nitrogenase transcriptional regulator	_	_	AZOBR_p350016	anc L
vnfD	nitrogenase vanadium-iron protein alpha chain	_	_	AZOBR_p350020	unassigned
vnfG	vanadium dinitrogenase delta subunit	_	_	AZOBR_p350021	unassigned
vnfK	nitrogenase vanadium-iron protein, VnfK subunit	_	_	AZOBR_p350022	unassigned
vnfY	nitrogen fixation-related protein	_	_	AZOBR_p350024	anc M
hupT	two-component sensor histidine kinase	AZOLI_p30053	AZL_a10670	_	unassigned
hupU	uptake hydrogenase accessory protein	AZOLI_p30054	AZL_a10680	_	hor H
hupV	uptake hydrogenase accessory protein	AZOLI_p30055	AZL_a10690	_	unassigned
hupD	hydrogenase expression/formation protein	AZOLI_p30060	AZL_c01910	_	hor H
hupF	hydrogenase expression/formation protein	AZOLI_p30061	AZL_c01920	_	unassigned
hupG	hydrogenase expression/formation protein	AZOLI_p30062	AZL_c01930	_	hor H
hupH	hydrogenase expression/formation protein	AZOLI_p30063	AZL_c01940	_	hor H
hupJ	rubredoxin	AZOLI_p30064	AZL_c01950	_	hor H

hypA1	hydrogenase nickel incorporation protein	AZOLI_p30065	AZL_c01960	_	hor H
hupR	hydrogenase transcriptional regulator	AZOLI_p30066	AZL_c01970	_	hor M
hupE	[NiFe]-hydrogenase/urease accessory protein	AZOLI_p30067	AZL_c01980	_	unassigned,anc L
hupS	uptake hydrogenase, small subunit	AZOLI_p30068	AZL_c01990	_	hor H
hupL	uptake hydrogenase, large subunit	AZOLI_p30069	AZL_c02000	_	hor H
hupC	b-type cytochrome subunit, Ni/Fe-hydrogenase	AZOLI_p30070	AZL_c02010	_	hor H
hypF	[NiFe]-hydrogenase maturation protein	AZOLI_p30072	AZL_c02002	AZOBR_p1130134	hor H
hupJ	rubredoxin	_	_	AZOBR_p1130138	hor H
hupF	hydrogenase expression/formation protein	_	_	AZOBR_p1130139	hor M
hupD	hydrogenase expression/formation protein	_	_	AZOBR_p1130144	unassigned
hupL	uptake hydrogenase, large subunit	_	_	AZOBR_p1130145	anc L
hupS	uptake hydrogenase, small subunit	_	_	AZOBR_p1130148	anc L
**Opine degradation** [12]					
occR	regulator of opine catabolism/uptake operon	AZOLI_p20663	_	AZOBR_p310231	hor H
ooxB	opine oxidase subunit B	AZOLI_p20665	_	AZOBR_p310237	hor H,hor M
_	putative sarcosine oxidase, alpha subunit	AZOLI_p20666	_	AZOBR_p310238	hor M
ooxA	opine oxidase subunit A	AZOLI_p20667	_	AZOBR_p310239	hor M,hor H
_	putative sarcosine oxidase, beta subunit	AZOLI_p20668	_	AZOBR_p310241	unassigned
occT	opine ABC transporter, substrate-binding protein	AZOLI_p20670	_	AZOBR_p310243	unassigned,anc L
**Organic acid transport & metabolism** [4]					
acsA	acetyl-CoA synthetase (EC 6.2.1.1)	AZOLI_2526	AZL_024380	AZOBR_10049	anc H
citE	citryl-CoA lyase (EC 4.1.3.34; component of EC 4.1.3.6)	AZOLI_0704	AZL_008940	AZOBR_200176	anc H
_	citrate CoA-transferase (EC 2.8.3.10; component of EC 4.1.3.6)	AZOLI_p40309	AZL_d02810	AZOBR_p250005	anc H
aceA	ATP citrate lyase (EC 4.1.3.1) (glyoxylate bypass)	AZOLI_p40649	AZL_d00840	AZOBR_10531	anc L,anc L,anc M
fdhA	tungsten-containing formate dehydrogenase, alpha subunit	AZOLI_p10713	_	AZOBR_p470032	unassigned,anc L
fdhB	tungsten-containing formate dehydrogenase, beta subunit	AZOLI_p10714	_	AZOBR_p470033	unassigned,hor M
fdsG	formate dehydrogenase, gamma subunit	AZOLI_p10068	AZL_a00150	AZOBR_p110137	hor L,hor L,anc L
fdsB	formate dehydrogenase, beta subunit	AZOLI_p10069	AZL_a00160	AZOBR_p110138	anc L
fdsA	formate dehydrogenase, alpha subunit	AZOLI_p10070	AZL_a00170	AZOBR_p110139	unassigned,unassigned, anc L
fdsC	formate dehydrogenase, accessory protein	AZOLI_p10071	AZL_a00180	AZOBR_p110140	anc L
fdsD	formate dehydrogenase, delta subunit	AZOLI_p10072	AZL_a00190	AZOBR_p110141	hor M,hor M,hor L
fumA	fumarate hydratase, class I (EC 4.2.1.2)	AZOLI_p10015	AZL_a07630	AZOBR_140310	hor H
fumC	fumarate hydratase, class II (EC 4.2.1.2)	AZOLI_1439	AZL_017510	AZOBR_100009	anc L
glcD	glycolate oxidase, subunit GlcD (EC 1.1.99.14)	AZOLI_0349	AZL_004970	AZOBR_70163	anc M,anc M,anc L
glcE	glycolate oxidase, subunit GlcE (EC 1.1.99.14)	AZOLI_0350	AZL_004980	AZOBR_70164	anc M
glcF	Glycolate oxidase, iron-sulfur subunit GlcF (EC 1.1.99.14)	AZOLI_0351	AZL_004990	AZOBR_70165	anc M

gcl	glyoxylate carboligase (EC 4.1.1.47)	AZOLI_p10310	AZL_a03460	AZOBR_110008	hor H
glxR1	tartronate semialdehyde reductase (EC 1.1.1.60)	AZOLI_p40225	AZL_d01180	AZOBR_110006	hor H
glxR2	tartronate semialdehyde reductase (EC 1.1.1.60)	AZOLI_2503	AZL_024200	AZOBR_200141	anc L
glxK	glycerate kinase (EC 2.7.1.31)	_	AZL_a08400	_	hor H
aceB	malate synthase G (EC 2.3.3.9) (glyoxylate bypass)	AZOLI_0237	AZL_003910	AZOBR_40127	anc L
glcB	malate synthase A (EC 2.3.3.9) (glyoxylate bypass)	AZOLI_p30247	AZL_c04210	_	anc L
oxc	oxalyl-CoA decarboxylase (EC 4.1.1.8)	AZOLI_p10318	AZL_a03520	AZOBR_p470034	hor H
frc2	formyl-CoA transferase (EC 2.8.3.16)	AZOLI_p10319	AZL_a03530	_	hor H
frc1	formyl-CoA transferase (EC 2.8.3.16)	AZOLI_0201	AZL_025840	AZOBR_p470024	unassigned,unassigned, anc L
ttuC	tartrate decarboxylase (EC 4.1.1.73)	AZOLI_p10927	AZL_c03210	_	hor H
_	acetate transporter	AZOLI_0083	_	_	anc M
oxlT2	oxalate/formate antiporter	AZOLI_p10287	AZL_a03270	AZOBR_110004	hor H
oxlT1	oxalate/formate antiporter	AZOLI_p10299	AZL_a03370	AZOBR_100402	hor H
dctA1	sodium:dicarboxylate symporter	AZOLI_p10630	AZL_a05650	AZOBR_p1140111	hor H
focA	formate transporter	AZOLI_p20457	AZL_b03100	AZOBR_p420011	hor H
dctA2	sodium:dicarboxylate symporter	AZOLI_p30043	AZL_b05170	_	hor H
_	putative oxalate/formate antiporter	AZOLI_1944	AZL_019790	AZOBR_p220002	hor H
_	citrate transporter	AZOLI_1954	AZL_b02760	_	anc L
dctP	TRAP dicarboxylate transporter, periplasmic component	AZOLI_0785	AZL_009450	AZOBR_140140	anc M
dctQ	TRAP dicarboxylate transporter, permease component	AZOLI_0786	AZL_009460	_	hor H,hor M
dctQ	TRAP dicarboxylate transporter, permease component	_	_	AZOBR_140139	anc M
dctM	TRAP dicarboxylate transporter, large permease component	AZOLI_0787	AZL_009470	AZOBR_140138	anc L,anc M,anc M
_	putative TRAP dicarboxylate transporter, large permease component	AZOLI_1591	AZL_012850	AZOBR_p1130058	anc M
_	putative TRAP dicarboxylate transporter, small permease component	AZOLI_1592	AZL_012840	AZOBR_p1130059	anc M
_	putative TRAP dicarboxylate transporter, periplasmic component	AZOLI_1727	AZL_018210	AZOBR_p1130057	anc M
_	TRAP dicarboxylate transporter, periplasmic component	AZOLI_2523	AZL_024360	AZOBR_30029	anc L
_	putative TRAP dicarboxylate transporter, permease component	_	_	AZOBR_30031	hor H
_	TRAP dicarboxylate transport system, periplasmic component	AZOLI_p10232	_	_	anc L
_	TRAP dicarboxylate transport system, small permease component	AZOLI_p10233	_	_	hor H
_	TRAP dicarboxylate transport system, large permease component	AZOLI_p10234	_	_	hor H
_	putative TRAP dicarboxylate transporter, periplasmic component	_	_	AZOBR_p110070	anc L
_	putative TRAP dicarboxylate transporter, small permease component	_	_	AZOBR_p110071	anc L
_	putative TRAP dicarboxylate transporter, large permease component	_	_	AZOBR_p110072	anc L
_	putative TRAP dicarboxylate transporter, periplasmic component	_	_	AZOBR_p130128	unassigned
_	putative TRAP dicarboxylate transporter, large permease component	_	_	AZOBR_p130130	hor H
_	putative TRAP dicarboxylate transporter, fused permease components	_	_	AZOBR_p130210	hor H

_	putative TRAP dicarboxylate transporter, periplasmic component	_	_	AZOBR_p130211	unassigned
_	TRAP dicarboxylate transporter, large permease component	AZOLI_p20176	AZL_a08570	_	hor H
_	putative TRAP dicarboxylate transporter, small permease component	AZOLI_p20177	AZL_a08580	_	hor H
_	TRAP dicarboxylate transporter, periplasmic component	AZOLI_p20180	AZL_a08600	_	hor H
_	putative dicarboxylate carrier MatC-like	AZOLI_p20198	AZL_b03470	_	hor H
_	TRAP dicarboxylate transport system, periplasmic component	AZOLI_p20448	_	_	hor H
_	TRAP dicarboxylate transport system, small permease component	AZOLI_p20449	_	_	anc L
_	TRAP dicarboxylate transport system, large permease component	AZOLI_p20450	_	_	hor H
_	TRAP dicarboxylate transport system, periplasmic component		_	AZOBR_p270290	anc L
_	TRAP dicarboxylate transport system, small permease component		_	AZOBR_p270291	anc L
_	TRAP dicarboxylate transport system, large permease component		_	AZOBR_p270292	hor H
_	TRAP dicarboxylate transporter, periplasmic component	AZOLI_p30078	_	AZOBR_p230051	anc L
_	TRAP dicarboxylate transporter, periplasmic component	AZOLI_p30079	_	AZOBR_p230052	unassigned
_	TRAP dicarboxylate transporter, permease component	AZOLI_p30080	_	AZOBR_p230053	anc L
_	putative TRAP dicarboxylate transporter, fused permease components	AZOLI_p30105	AZL_c02230	AZOBR_120023	anc M,anc L,anc M
_	putative TRAP dicarboxylate transporter, periplasmic component	AZOLI_p30106	AZL_c02240	AZOBR_120022	anc M
_	putative TRAP dicarboxylate transporter, fused permease components	_	AZL_a04620	AZOBR_p310082	hor H
_	putative TRAP dicarboxylate transporter, periplasmic component	_	AZL_a04630	AZOBR_p310083	hor H
_	TRAP dicarboxylate transport system, large permease component	_	_	AZOBR_p310124	hor H
_	TRAP dicarboxylate transport system, small permease component	_		AZOBR_p310125	hor H
_	TRAP dicarboxylate transport system, large permease component	_	_	AZOBR_p310249	hor H
_	TRAP dicarboxylate transport system, small permease component	_	_	AZOBR_p310250	hor H
_	TRAP dicarboxylate transport system, periplasmic component	_	_	AZOBR_p310251	hor H
_	TRAP dicarboxylate transport system, periplasmic component	AZOLI_p40037	AZL_d00410	AZOBR_p130073	hor H
_	TRAP dicarboxylate transport system, large permease component	AZOLI_p40038	AZL_d00400	AZOBR_p130074	hor H
_	TRAP dicarboxylate transport system, small permease component	AZOLI_p40039	AZL_d00390	AZOBR_p130075	hor H
_	TRAP dicarboxylate transport system, periplasmic component	AZOLI_p40233	_	AZOBR_p450012	hor H
_	TRAP dicarboxylate transport system, small permease component	AZOLI_p40234	_	AZOBR_p450013	hor H
_	TRAP dicarboxylate transport system, large permease component	AZOLI_p40235	_	AZOBR_p420004	hor H
_	TRAP dicarboxylate transport system, small permease component	_	_	AZOBR_p420005	hor H

_	TRAP dicarboxylate transport system, periplasmic component	_	_	AZOBR_p420006	hor H
_	TRAP dicarboxylate transport system, large permease component	_	_	AZOBR_p410027	hor H
_	TRAP dicarboxylate transport system, small permease component	_	_	AZOBR_p410028	unassigned
_	TRAP dicarboxylate transport system, periplasmic component	_	_	AZOBR_p410029	anc L
_	TRAP dicarboxylate transporter, periplasmic component	AZOLI_p50106	AZL_e02190	_	hor H
_	TRAP dicarboxylate transporter, small permease component	AZOLI_p50107	AZL_e02180	_	hor H
_	TRAP dicarboxylate transporter, large permease component	AZOLI_p50108	AZL_e02170	_	hor H
_	TRAP dicarboxylate transporter, small permease component	AZOLI_p50112	AZL_e03940	AZOBR_30030	anc L
_	putative TRAP dicarboxylate transporter, permease component	AZOLI_p50113	AZL_e03950	_	hor H

Osmoprotection [13]

otsA	trehalose 6-phosphate synthase	AZOLI_0165	AZL_002860	AZOBR_70093	anc M
otsB	trehalose 6-phosphate phosphatase	AZOLI_0395	AZL_005410	AZOBR_160045	anc L
potA	spermidine/putrescine ABC transporter, ATP-binding component	AZOLI_0010	AZL_026670	AZOBR_10275	anc L
_	spermidine/putrescine ABC transporter, periplasmic component	AZOLI_0011	AZL_026680	AZOBR_10276	anc L
_	polyamine ABC transporter, permease component	AZOLI_2708	AZL_003310	AZOBR_10269	anc L
_	polyamine ABC transporter, permease component	AZOLI_2709	AZL_003300	AZOBR_10270	anc L
_	putative glycine betaine ABC transporter, ATPase component	AZOLI_p10866	_	_	unassigned
_	glycine betaine ABC transporter, substrate-binding component	AZOLI_p10867	AZL_c01750	AZOBR_p220082	hor H
proV1	glycine betaine ABC transporter, ATP-binding component	AZOLI_p10877	AZL_c01830	_	hor H
_	glycine betaine ABC transporter, ATP-binding component			AZOBR_p220084	hor H
_	glycine betaine ABC transporter, permease component	AZOLI_p10878	AZL_c01840	_	hor H
_	glycine betaine ABC transporter, permease component			AZOBR_p220083	hor H
_	polyamine ABC transporter, periplasmic component	AZOLI_p20199	AZL_a10530	_	unassigned
_	polyamine ABC transporter, ATP-binding component	AZOLI_p20200	AZL_a10520	_	hor H
_	polyamine ABC transporter, permease component	AZOLI_p20201	AZL_a10510	_	anc L
betT	Betaine/Carnitine/Choline transporter	AZOLI_p20508	_	AZOBR_p220028	hor H
_	putative spermidine/putrescine ABC transporter, permease component	AZOLI_p20565	AZL_b05110	AZOBR_p130048	hor H
_	putative spermidine/putrescine ABC transporter, permease component	AZOLI_p20566	AZL_b05100	AZOBR_p130047	hor H
_	putative spermidine/putrescine ABC transporter substrate-binding protein	AZOLI_p20567	AZL_b05090	AZOBR_p130045	hor H
_	putative spermidine/putrescine ABC transporter subunit, ATP-binding component	AZOLI_p20568	AZL_b05080	AZOBR_p130044	hor H
proX	proline/glycine betaine ABC transporter, periplasmic component	AZOLI_p30129	AZL_c02440	_	unassigned,anc L
proW	proline/glycine betaine ABC transporter, membrane component	AZOLI_p30131	AZL_c02460	_	hor H
proV2	proline/glycine betaine ABC transporter, ATP-binding component	AZOLI_p30132	AZL_c02470	_	hor H

potI	putrescine ABC transporter, permease component	AZOLI_p30328	AZL_c00720	AZOBR_130014	anc H
potH	putrescine ABC transporter, permease component	AZOLI_p30329	AZL_c00710	AZOBR_130015	anc M
potG	putrescine ABC transporter, ATP-binding component	AZOLI_p30330	AZL_c00700	AZOBR_130016	anc L,anc L,anc M
potF	putrescine ABC transporter, periplasmic component	AZOLI_p30331	AZL_c00690	AZOBR_130017	anc M
_	putative spermidine/putrescine ABC transporter, permease component	AZOLI_p30380	AZL_c00230	AZOBR_p1120051	anc L
_	putative spermidine/putrescine ABC transporter, periplasmic component	AZOLI_p30381	AZL_c00220	AZOBR_p1120052	anc L
_	putative spermidine/putrescine ABC transporter, ATP-binding component	AZOLI_p30382	AZL_c00210	_	anc L
_	glycine betaine/choline ABC transporter, permease component	AZOLI_p30579	AZL_a10070	AZOBR_p130038	hor H
_	glycine betaine/choline ABC transporter, ATP-binding component	AZOLI_p30580	AZL_a10060	AZOBR_p130039	hor H
_	glycine betaine/choline ABC transporter, permease component	AZOLI_p30581	AZL_a10050	AZOBR_p130040	hor H
**Oxidative stress resistance** [14]					
sodB1	superoxide dismutase Fe/Mn cofactor (EC 1.15.1.1)	AZOLI_1390	AZL_014560	AZOBR_p120093	anc M
sodB2	superoxide dismutase Fe/Mn cofactor (EC 1.15.1.1)	AZOLI_p20656	_	_	anc L
sodB	superoxide dismutase Fe/Mn cofactor (EC 1.15.1.1)	_	_	AZOBR_p440007	anc L
sodC	superoxide dismutase (Cu-Zn) (EC 1.15.1.1)	AZOLI_2596	AZL_024870	_	hor H
katN	catalase (EC 1.11.1.6)	AZOLI_p10484	AZL_a04090	AZOBR_140171	hor H
katG	catalase (EC 1.11.1.6)	AZOLI_p30178	_	_	hor H
katA	catalase (EC 1.11.1.6)	AZOLI_p30387	AZL_c00160	AZOBR_p440183	hor M
**Polyhydroxybutyrate synthesis** [15]					
phbC	PHB polymerase (EC 2.3.1.-)	AZOLI_p10260	AZL_a02630	AZOBR_100383	anc H
_	PHB depolymerase (EC 3.1.1.75)	AZOLI_p10266	AZL_a02680	_	hor M,unassigned
_	PHB depolymerase (EC 3.1.1.75)	AZOLI_p10267	AZL_a02690	_	hor M,unassigned
_	putative polyhydroxyalkanoate synthase (EC 2.3.1.-)	AZOLI_2389	AZL_023140	AZOBR_p440016	anc H
**Polyketide synthase** [16]					
_	putative beta-ketoacyl synthase	AZOLI_p10840	_	_	hor H
_	putative chalcone synthase (EC 2.3.1.74)	AZOLI_p30075	_	AZOBR_140202	anc L
**Quinone oxidation** [17]					
_	Putative Mn2+ multicopper oxidase (laccase-like)	AZOLI_p30139	AZL_c02540	_	anc L
_	Putative Mn2+ multicopper oxidase (laccase-like)	_	_	AZOBR_10264	anc L
_	Putative multicopper oxidase (laccase-like)	AZOLI_1528	AZL_011040	AZOBR_p440175	hor L,hor L,anc L
_	Putative multicopper oxidase (laccase-like)	AZOLI_1691	_	_	hor H
**RTX toxin synthesis** [18]					
_	RTX toxins and related Ca2+-binding domain (1950 AA)	AZOLI_0777	_	AZOBRv2_40002	anc L,hor H
_	putative hemolysin-type Calcium-binding RTX toxin	AZOLI_p20390	AZL_a07150	AZOBR_p210039	unassigned

_	putative Ca binding RTX toxin (5193 AA)	AZOLI_p30303	AZL_c04560	_	anc L
_	RTX toxins and related Ca2+-binding domain (6548 AA)	AZOLI_p40606	AZL_a02370	AZOBR_p1140098	hor H,hor H,hor M
_	RTX toxins and related Ca2+-binding domain (5184 AA)	AZOLI_p60065	_	_	hor H
_	RTX toxins and related Ca2+-binding domain	AZOLI_p60167	AZL_f00090	_	anc L,hor H
_	RTX toxins and related Ca2+-binding domain (2840 AA)	AZOLI_p60263	AZL_f01550	AZOBR_p270071	unassigned,hor H,anc L
_	RTX toxins and related Ca2+-binding domain	_	AZL_a06050	_	hor H
**Siderophore biosynthesis and transport** [19]					
entC	isochorismate synthase I (EC 5.4.4.2)	_	_	AZOBR_p350073	hor H
entE	2,3-dihydroxybenzoate-AMP ligase (EC 2.7.7.58)	_	_	AZOBR_p350074	hor H
entB	isochorismatase (EC 3.3.2.1)	_	_	AZOBR_p350075	hor H
entA	2,3-dihydroxybenzoate dehydrogenase (EC 1.3.1.28)	_	_	AZOBR_p350076	hor H
entF	enterobactin synthase (EC 2.7.7.-)	_	_	AZOBR_p350079	hor H
pchD	2,3-dihydroxybenzoate-AMP ligase	AZOLI_p20159	_	_	hor H
pchE	pyochelin synthase E	AZOLI_p20160	_	_	hor H
pchF	pyochelin synthetase F	AZOLI_p20161	_	_	hor H
pchC	pyochelin biosynthetic thioesterase	AZOLI_p20163	_	_	hor H
pchA	isochorismate synthase	AZOLI_p20165	_	_	hor H
_	putative isochorismatase hydrolase	AZOLI_1628	AZL_016380	AZOBR_p1180028	anc L,anc M,anc H
_	putative siderophore biosynthesis protein RhbC-like	AZOLI_p20604	_	_	hor H
_	putative siderophore biosynthesis protein, RhsF-like	AZOLI_p20605	_	_	hor H
entS	entereobactin exporter	_	_	AZOBR_p350069	hor H
tonB	Ferric siderophore transporter	AZOLI_p40405	AZL_d01940	AZOBR_p210088	anc L
hmuV	hemin ABC transporter, ATP-binding component	AZOLI_p20677	_	AZOBR_p350048	hor H
hmuT	hemin ABC transporter, periplasmic component	AZOLI_p20679	_	AZOBR_p350046	hor H,hor M
hmuU	hemin ABC transporter, permease component	AZOLI_p20678	_	AZOBR_p350047	hor H
fbpC	iron ABC transporter, ATP-binding component	AZOLI_p10397	AZL_a07840	_	hor H
fhuC	iron-hydroxamate ABC transporter, ATP-binding component	AZOLI_p20504	_	AZOBR_p170129	hor H
fhuD	iron-hydroxamate ABC transporter, periplasmic component	AZOLI_p20505	_	AZOBR_p170128	hor H
fhuB	iron-hydroxamate ABC transporter, permease component	AZOLI_p20506	_	AZOBR_p170127	hor H
_	iron-siderophore ABC transporter, ATP-binding component	_	_	AZOBR_p1110105	anc L
_	iron-siderophore ABC transporter, ATP-binding component	AZOLI_p10041	_	_	unassigned
fecE	iron-siderophore ABC transporter, ATP-binding component	AZOLI_p20475	_	AZOBR_p1110094	hor H
_	iron-siderophore ABC transporter, ATP-binding component	AZOLI_p20619	AZL_004840	_	hor H
_	iron-siderophore ABC transporter, ATP-binding component	AZOLI_p20621	AZL_004820	_	hor H
_	iron-siderophore ABC transporter, periplasmic component	_	_	AZOBR_p1110107	anc L
_	iron-siderophore ABC transporter, periplasmic component	AZOLI_p10043	_	_	hor M

_	iron-siderophore ABC transporter, periplasmic component	AZOLI_p20478	_	AZOBR_p1110097	hor H
_	iron-siderophore ABC transporter, permease component	_	_	AZOBR_p1110106	anc L
_	iron-siderophore ABC transporter, permease component	AZOLI_p10042	_	_	hor M
_	iron-siderophore ABC transporter, permease component	AZOLI_p20476	_	AZOBR_p1110095	hor H
_	iron-siderophore ABC transporter, permease component	AZOLI_p20477	_	AZOBR_p1110096	hor H
exbD	membrane spanning protein in TonB-ExbB-ExbD complex	AZOLI_p40406	AZL_d01930	AZOBR_p210087	unassigned,hor M, unassigned
exbB	membrane spanning protein in TonB-ExbB-ExbD complex	AZOLI_p40407	AZL_d01920	AZOBR_p210086	hor H
_	putative ferrous iron transporter, FeoA-like subunit	AZOLI_p20553	AZL_b04590	_	anc L
_	putative ferrous iron transporter, FeoB-like subunit	AZOLI_p20554	AZL_b04580	AZOBR_p170036	anc L
_	putative iron ABC transporter, periplasmic component	AZOLI_p10394	AZL_a07860	AZOBR_p140003	anc L,unassigned, unassigned
_	putative iron ABC transporter, periplasmic component	AZOLI_p10400	AZL_a07810	_	hor H
_	putative iron ABC transporter, periplasmic component	AZOLI_p20620	AZL_004830	_	hor H
_	putative iron ABC transporter, permease component	AZOLI_p10393	AZL_a07870	AZOBR_p140002	hor H
_	putative iron-siderophore ABC transporter, periplasmic component	_	_	AZOBR_p220058	hor H
_	putative iron-siderophore ABC transporter, permease component	_	_	AZOBR_p220059	hor H
_	putative siderophore ABC transporter, ATP-binding component	AZOLI_p20154	_	_	hor M
_	putative siderophore ABC transporter, ATP-binding component	_	_	AZOBR_p220061	hor H
_	putative siderophore ABC transporter, periplasmic component	AZOLI_p20156	_	_	anc L
_	putative siderophore ABC transporter, periplasmic component	_	AZL_026430	_	anc L
_	putative siderophore ABC transporter, periplasmic component	AZOLI_p50135	_	_	hor L
_	putative siderophore ABC transporter, permease component	AZOLI_p20155	_	_	hor L
_	putative siderophore ABC transporter, permease component	_	AZL_026420	_	hor L
_	putative siderophore-interacting protein	_	_	AZOBR_p220060	hor H
_	putative siderophore-interacting protein, MxcB-like	AZOLI_p20501	_	_	hor M
_	putative TonB-dependent receptor	_	AZL_028160	_	hor H
_	putative TonB-dependent receptor	_	AZL_007800	_	anc L
_	putative TonB-dependent receptor	_	AZL_a08620	AZOBR_p440028	anc L
_	putative TonB-dependent receptor	_	_	AZOBR_p1110108	anc L
_	putative TonB-dependent receptor	AZOLI_p20602	_	AZOBR_p440066	unassigned
_	putative TonB-dependent siderophore receptor	AZOLI_p20157	_	AZOBR_p220057	hor H
_	putative TonB-dependent siderophore receptor	AZOLI_p20623	AZL_004810	_	hor H
_	putative TonB-like ferric siderophore receptor	AZOLI_p20482	_	AZOBR_p1110090	hor L
_	siderophore ABC transporter, ATP-binding component	AZOLI_p50132	AZL_026410	_	hor L
_	siderophore ABC transporter, permease component	AZOLI_p50133	_	_	hor L

_	siderophore-interacting protein	AZOLI_p50134	_	_	hor L
_	TonB-dependent outer membrane hemin receptor	AZOLI_p20672	_	AZOBR_p350053	hor H
_	TonB-dependent outer membrane receptor	AZOLI_p10047	_	_	anc L
_	TonB-dependent siderophore receptor	AZOLI_1178	_	AZOBR_p310289	anc L
fhuA	TonB-dependent siderophore receptor	AZOLI_p20503	_	AZOBR_p170130	hor H
_	TonB-dependent siderophore receptor, FhuA-like	AZOLI_p10405	AZL_d04450	_	hor H,hor M
_	TonB-dependent siderophore receptor, FhuA-like	_	_	AZOBR_p310120	hor H
_	TonB-dependent siderophore receptor, FhuA-like	AZOLI_p50136	_	_	hor H
**Type VI secretion system** [20]					
_	putative component of type VI secretion system	AZOLI_0998	AZL_017990	_	hor L
_	putative component of type VI secretion system	AZOLI_0999	AZL_017980	_	hor M,hor H
_	putative component of type VI secretion system	AZOLI_1000	AZL_017970	_	hor L,hor M
_	putative component of type VI secretion system	AZOLI_1001	AZL_017960	_	anc L
_	putative component of type VI secretion system	AZOLI_1004	AZL_017930	_	hor M
_	putative component of type VI secretion system	AZOLI_1005	AZL_017920	_	hor L
_	putative component of type VI secretion system	AZOLI_1006	AZL_017910	_	hor L
_	putative component of type VI secretion system	AZOLI_1007	AZL_017900	_	hor L
_	putative component of type VI secretion system	AZOLI_1008	AZL_017890	_	hor M
_	putative component of type VI secretion system	AZOLI_1009	AZL_017880	_	hor L
_	putative component of type VI secretion system	AZOLI_1010	AZL_017870	_	hor L
_	putative component of type VI secretion system	AZOLI_1011	AZL_017860	_	hor M
_	putative component of type VI secretion system	AZOLI_1015	AZL_017820	_	hor L
_	putative component of type VI secretion system	AZOLI_1016	AZL_017810	_	hor H,hor M
_	putative component of type VI secretion system	AZOLI_1020	AZL_017780	_	anc L,hor L
_	putative component of type VI secretion system	AZOLI_1021	AZL_017770	_	hor L
_	putative effector of type VI secretion system (IcmH-like)	AZOLI_p30482	_	_	hor L
_	putative effector of type VI secretion system (IcmF-like)	AZOLI_p30483	_	_	hor M
_	putative effector of type VI secretion system (Vgr-like)	AZOLI_p30485	_	_	hor H
_	putative effector of type VI secretion system (FHA domain)	AZOLI_p30488	_	_	hor L
_	putative serine-threonine kinase, type VI secretion system associated	AZOLI_p30489	_	_	hor H
_	putative effector of type VI secretion system	_	AZL_a01570	AZOBR_p1150012	hor H
_	putative effector of type VI secretion system	_	AZL_a01580	_	hor H
_	putative effector of type VI secretion system	_	AZL_a01590	AZOBR_p1150011	hor H
_	putative effector of type VI secretion system	_	AZL_a01600	AZOBR_p1150009	hor H
_	putative effector of type VI secretion system	_	AZL_a01610	AZOBR_p1150008	hor H
_	putative effector of type VI secretion system	_	AZL_a01700	AZOBR_p1140133	hor H

_	putative effector of type VI secretion system	_	AZL_a01710	AZOBR_p1140132	hor H
_	putative effector of type VI secretion system	_	AZL_a01720	AZOBR_p1140131	hor H
_	putative effector of type VI secretion system	_	AZL_a01730	AZOBR_p1140130	hor H
_	putative effector of type VI secretion system	_	AZL_a01740	AZOBR_p1140129	hor H
_	putative effector of type VI secretion system	_	AZL_a01750	AZOBR_p1140128	hor H
_	putative effector of type VI secretion system	_	AZL_a01770	AZOBR_p1140126	hor H
_	putative effector of type VI secretion system	_	AZL_a01780	AZOBR_p1140125	hor M,anc L
**Vitamin and cofactor biosynthesis** [21]					
ldc	lysine/ornithine decarboxylase	AZOLI_0502	AZL_006200	AZOBR_10013	anc L
thiG	thiamine biosynthesis (Vitamin B1)	AZOLI_0318	AZL_004540	AZOBR_p440103	anc L,anc M,anc M
thiS	thiamine biosynthesis	AZOLI_0319	AZL_004530	AZOBR_p440104	anc L,anc L,unassigned
thiO	thiamine biosynthesis	AZOLI_0320	AZL_004520	AZOBR_p440105	anc L
thiC	thiamine biosynthesis	AZOLI_0384	AZL_005290	AZOBR_110003	anc M
thiE	thiamine biosynthesis	AZOLI_0809	AZL_009640	AZOBR_150017	anc H
thiL	thiamine biosynthesis	AZOLI_0867	AZL_010130	AZOBR_140246	anc M
thiF	thiamine biosynthesis	AZOLI_2660	AZL_003700	AZOBR_10238	anc H,anc H,anc M
thiD	thiamine biosynthesis	AZOLI_p50062	AZL_e01260	AZOBR_100217	anc H
_	riboflavin biosynthesis (Vitamin B2)	AZOLI_0057	AZL_026320	AZOBR_10418	anc H
ribF	riboflavin biosynthesis	AZOLI_0507	AZL_006230	AZOBR_70017	anc H
ribH	riboflavin biosynthesis	AZOLI_0869	AZL_010150	AZOBR_140244	anc M
ribBA	riboflavin biosynthesis	AZOLI_0870	AZL_010160	AZOBR_140243	anc H
ribC	riboflavin biosynthesis	AZOLI_0871	AZL_010170	AZOBR_140242	anc H
ribD	riboflavin biosynthesis	AZOLI_0872	AZL_010180	AZOBR_140241	anc H
_	nicotinate/nicotinamide biosynthesis (Vitamin B3/PP)	AZOLI_0290	AZL_004310	AZOBR_40269	anc M
_	nicotinate/nicotinamide biosynthesis	AZOLI_2420	AZL_023370	AZOBR_40318	anc M,anc H,anc H
xapA	nicotinate/nicotinamide biosynthesis	AZOLI_p10492	AZL_a04160	AZOBR_100129	hor H
_	nicotinate/nicotinamide biosynthesis	AZOLI_p10549	AZL_a07010	AZOBR_p1110150	hor H
_	nicotinate/nicotinamide biosynthesis	AZOLI_p10607	AZL_a05550	AZOBR_p140049	anc M,anc M,unassigned
nadE	nicotinate/nicotinamide biosynthesis	AZOLI_p30432	AZL_c05250	AZOBR_p170147	hor H
nadC	nicotinate/nicotinamide biosynthesis	AZOLI_p40290	AZL_d02900	AZOBR_p1100106	anc M
nadB	nicotinate/nicotinamide biosynthesis	AZOLI_p40291	AZL_d02890	AZOBR_p1100108	anc L,anc L,anc M
nadA	nicotinate/nicotinamide biosynthesis	AZOLI_p40293	AZL_d02880	AZOBR_p1110003	anc L
pntB1	nicotinate/nicotinamide biosynthesis	AZOLI_p10305	AZL_013370	AZOBR_100082	anc M
pntB2	nicotinate/nicotinamide biosynthesis	AZOLI_p40620	AZL_a03420	AZOBR_100410	anc M
pntAB1	nicotinate/nicotinamide biosynthesis	AZOLI_p10304	AZL_a03410	AZOBR_100409	anc H
pntAB2	nicotinate/nicotinamide biosynthesis	AZOLI_p40621	AZL_013360	AZOBR_100081	anc H

pntAA1	nicotinate/nicotinamide biosynthesis	AZOLI_p10303	AZL_013350	AZOBR_100080	anc L
pntAA2	nicotinate/nicotinamide biosynthesis	AZOLI_p40622	_	_	unassigned
panB	pantothenate biosynthesis (Vitamin B5)	AZOLI_1886	AZL_019370	AZOBR_100084	anc H,anc M,anc M
panE	pantothenate biosynthesis	AZOLI_p10301	AZL_a03380	AZOBR_100406	hor H
panC	pantothenate biosynthesis	AZOLI_p10480	AZL_a03250	AZOBR_140166	anc H
pdxY	pyridoxine biosynthesis (Vitamin B6)	AZOLI_0705	AZL_008950	AZOBR_200175	anc L
pdxH	pyridoxine biosynthesis	AZOLI_0845	AZL_009950	AZOBR_140052	anc M,anc L,anc M
pdxJ	pyridoxine biosynthesis	AZOLI_1507	AZL_011790	AZOBR_p1170088	anc H
pdxA	pyridoxine biosynthesis	AZOLI_1511	AZL_011750	AZOBR_p130179	anc H
_	pyridoxine biosynthesis	AZOLI_p20631	AZL_a10620	AZOBR_p220105	hor H
dxs	pyridoxine biosynthesis	AZOLI_p30184	AZL_c00990	AZOBR_p1140022	anc M
serC	pyridoxine biosynthesis	AZOLI_p30197	AZL_c00880	AZOBR_150084	anc M,anc M,anc H
bioF	biotin biosynthesis (Vitamin B8)	_	_	AZOBR_p440134	anc L
bioCD	biotin biosynthesis	_	_	AZOBR_p440137	anc L
bioA	biotin biosynthesis	_	_	AZOBR_p440138	anc L
bioB	biotin biosynthesis	_	_	AZOBR_p440139	hor H
folD	folate biosynthesis (Vitamin B9)	AZOLI_2920	AZL_026930	AZOBR_10134	hor H
folC	folate biosynthesis	AZOLI_2925	AZL_027050	AZOBR_10102	anc M
folA	folate biosynthesis	AZOLI_3018	AZL_000160	AZOBR_10482	hor H
folE	folate biosynthesis	AZOLI_3091	AZL_028440	AZOBR_10090	anc H
folK	folate biosynthesis	AZOLI_p10810	AZL_a04360	AZOBR_p110121	anc M
folB-like	folate biosynthesis	AZOLI_p10900	AZL_c01880	_	anc H
folP	folate biosynthesis	AZOLI_p50060	AZL_e01240	AZOBR_100215	anc H
pabC	folate biosynthesis	AZOLI_p30457	AZL_c04960	AZOBR_p230039	anc M,anc M,anc L
pabB	folate biosynthesis	AZOLI_p30458	AZL_c04970		anc M
_	folate biosynthesis	_	_	AZOBR_p230038	anc M
cobT	cobalamin biosynthesis (Vitamin B12)	AZOLI_0359	AZL_005060	AZOBR_40175	anc H,anc H,anc M
cobU	cobalamin biosynthesis	AZOLI_1242	AZL_015930	AZOBR_140190/92	hor M
cobO	cobalamin biosynthesis	AZOLI_1243	AZL_015920	AZOBR_140191	hor H,hor M,hor M
cobQ	cobalamin biosynthesis	AZOLI_1244	AZL_015910	AZOBR_140192	anc L,anc M,anc M
cobS	cobalamin biosynthesis	AZOLI_2951	AZL_000760	AZOBR_40113/109	anc H,anc H,anc M
cobT	cobalamin biosynthesis	AZOLI_2953	AZL_000750	AZOBR_40109	anc H
bluB	cobalamin biosynthesis	AZOLI_p40236	AZL_d03400	AZOBR_p140044	anc M,anc M,unassigned
cobB	cobalamin biosynthesis	AZOLI_p40237	AZL_d03390	AZOBR_p140043	unassigned,anc M,hor H
cobA	cobalamin biosynthesis	AZOLI_p40238	AZL_d03380	AZOBR_p140042	hor M
cbiD	cobalamin biosynthesis	AZOLI_p40239	AZL_d03370	AZOBR_p140041	anc M

cobM	cobalamin biosynthesis	AZOLI_p40240	AZL_d03360	AZOBR_p140040	hor M
cobE	cobalamin biosynthesis	AZOLI_p40241	AZL_d03350	AZOBR_p140039	unassigned,hor M,hor H
cobL	cobalamin biosynthesis	AZOLI_p40242	AZL_d03340	AZOBR_p140038	anc M,anc L,anc M
cobK	cobalamin biosynthesis	AZOLI_p40243	AZL_d03330	AZOBR_p140037	hor M
cobJ	cobalamin biosynthesis	AZOLI_p40244	AZL_d03320	AZOBR_p140036	anc L,anc L,hor M
cobI	cobalamin biosynthesis	AZOLI_p40245	AZL_d03310	AZOBR_p140035	hor H
cobH	cobalamin biosynthesis	AZOLI_p40246	AZL_d03300	AZOBR_p140033	hor M,hor M,hor H
cobG	cobalamin biosynthesis	AZOLI_p40247	AZL_d03290	AZOBR_p140032	hor H
cobN	cobalamin biosynthesis	AZOLI_p40248	AZL_d03280	AZOBR_p140031	hor L
cobW	cobalamin biosynthesis	AZOLI_p40249	AZL_d03270	AZOBR_p140030	unassigned,hor M,anc L
cbtA	cobalamin biosynthesis	AZOLI_p40250	AZL_d03260	_	anc L
cbtB	cobalamin biosynthesis	AZOLI_p40251	AZL_d03250	_	anc L
pqqA2	coenzyme PQQ biosynthesis (pyrroloquinoline quinone)	AZOLI_p10697	_		unassigned
pqqA	coenzyme PQQ biosynthesis	_	_	AZOBR_p270073	hor H
pqqA1	coenzyme PQQ biosynthesis	AZOLI_p30590	_	AZOBR_100324	hor H
pqqB	coenzyme PQQ biosynthesis	AZOLI_p30589	AZL_a09970	AZOBR_100323	anc L
pqqC	coenzyme PQQ biosynthesis	AZOLI_p30588	AZL_a09980	AZOBR_100322	anc L
pqqD	coenzyme PQQ biosynthesis	AZOLI_p30587	AZL_a09990	AZOBR_100321	hor M,hor M,hor L
pqqE	coenzyme PQQ biosynthesis	AZOLI_p30586	AZL_a10000	AZOBR_100320	hor L

*Unless otherwise indicated, the orthologues have the same assignment.

References:

1. Putnoky P, Kereszt A, Nakamura T, Endre G, Grosskopf E, et al. (1998) The pha gene cluster of *Rhizobium meliloti* involved in pH adaptation and symbiosis encodes a novel type of K+ efflux system. Molecular Microbiology 28: 1091-1101.
2. Peters NK, Verma DPS (1990) Phenolic-compounds as regulators of gene-expression in plant-microbe interactions. Mol Plant Microbe In 3: 4-8.
3. Dorr J, Hurek T, Reinhold-Hurek B (1998) Type IV pili are involved in plant-microbe and fungus-microbe interactions. Molecular Microbiology 30: 7-17.
4. Lugtenberg B, Kamilova F (2009) Plant-growth-promoting rhizobacteria. Annual Review of Microbiology 63: 541-556.
5. Meneses CH, Rouws LF, Simoes-Araujo JL, Vidal MS, Baldani JI (2011) Exopolysaccharide production is required for biofilm formation and plant colonization by the nitrogen-fixing endophyte *Gluconacetobacter diazotrophicus*. Mol Plant Microbe Interact.
6. Tegos G, Stermitz FR, Lomovskaya O, Lewis K (2002) Multidrug pump inhibitors uncover remarkable activity of plant antimicrobials. Antimicrob Agents Ch 46: 3133-3141.

7. Jimenez-Zurdo JI, Mateos PF, Dazzo FB, MartinezMolina E (1996) Cell-bound cellulase and polygalacturonase production by *Rhizobium* and *Bradyrhizobium* species. Soil Biol Biochem 28: 917-921.
8. Dobbelaere S, Croonenborghs A, Thys A, Vande Broek A, Vanderleyden J (1999) Phytostimulatory effect of *Azospirillum brasilense* wild type and mutant strains altered in IAA production on wheat. Plant Soil 212: 155-164.
9. Sy A, Timmers ACJ, Knief C, Vorholt JA (2005) Methylotrophic metabolism is advantageous for *Methylobacterium extorquens* during colonization of *Medicago truncatula* under competitive conditions. Appl Environ Microb 71: 7245-7252.
10. Zhang F, Dashti N, Hynes RK, Smith DL (1996) Plant growth promoting rhizobacteria and soybean [*Glycine max* (L) Merr] nodulation and nitrogen fixation at suboptimal root zone temperatures. Ann Bot-London 77: 453-459.
11. Zargar MY, Kahlon RS (1995) Comparison of symbiotic effectiveness of *Rhizobium* sp with positive hydrogen-uptake activity compared with negative mutants in relation to nitrogen-fixation in Mungbean (*Vigna radiata* L). Biol Fert Soils 20: 270-274.
12. Savka MA, Farrand SK (1997) Modification of rhizobacterial populations by engineering bacterium utilization of a novel plant-produced resource. Nat Biotechnol 15: 363-368.
13. Cassan F, Maiale S, Masciarelli O, Vidal A, Luna V, et al. (2009) Cadaverine production by *Azospirillum brasilense* and its possible role in plant growth promotion and osmotic stress mitigation. Eur J Soil Biol 45: 12-19.
14. Matilla MA, Espinosa-Urgel M, Rodriguez-Herva JJ, Ramos JL, Ramos-Gonzalez MI (2007) Genomic analysis reveals the major driving forces of bacterial life in the rhizosphere. Genome Biology 8.
15. Fallik E, Okon Y (1996) Inoculants of *Azospirillum brasilense*: Biomass production, survival and growth promotion of *Setaria italica* and *Zea mays*. Soil Biol Biochem 28: 123-126.
16. Bender CL, Rangaswamy V, Loper J (1999) Polyketide production by plant-associated pseudomonads. Annu Rev Phytopathol 37: 175-196.
17. Berg G (2009) Plant-microbe interactions promoting plant growth and health: perspectives for controlled use of microorganisms in agriculture. Appl Microbiol Biot 84: 11-18.
18. Oresnik IJ, Twelker S, Hynes MF (1999) Cloning and characterization of a *Rhizobium leguminosarum* gene encoding a bacteriocin with similarities to RTX toxins. Appl Environ Microb 65: 2833-2840.
19. Molina MA, Godoy P, Ramos-Gonzalez MI, Munoz N, Ramos JL, et al. (2005) Role of iron and the TonB system in colonization of corn seeds and roots by *Pseudomonas putida* KT2440. Environmental Microbiology 7: 443-449.
20. Filloux A, Hachani A, Bleves S (2008) The bacterial type VI secretion machine: yet another player for protein transport across membranes. Microbiol-Sgm 154: 1570-1583.
21. Marek-Kozaczuk M, Skorupska A (2001) Production of B-group vitamins by plant growth-promoting *Pseudomonas fluorescens* strain 267 and the importance of vitamins in the colonization and nodulation of red clover. Biol Fert Soils 33: 146-151.
