# Supplementary material for: Azospirillum Genomes Reveal Transition of Bacteria from Aquatic to Terrestrial Environments
Source: PLoS Genet. 2011 Dec 22;7(12):e1002430. doi: 10.1371/journal.pgen.1002430 (PMC3245306; doi:10.1371/journal.pgen.1002430)
Supplement: Table S8 — Divergence in the 16S rRNA gene between Azospirillum lipoferum 4B and other members of Rhodospirillaceae. (PDF) [file pgen.1002430.s011.pdf]

Table S8. Divergence in the 16S rRNA gene between *Azospirillum lipoferum* 4B and other members of *Rhodospirillaceae*

	***Azospirillum lipoferum* 4B**
Azospirillum sp. B510	2.16 %
Azospirillum brasilense Sp245	2.98 %
Rhodospirillum centenum SW	8.03 %
Nisaea sp. BAL199	9.18 %
Magnetospirillum magnetotacticum MS-1	9.34 %
Magnetospirillum magneticum AMB-1	9.46 %
Magnetospirillum gryphiswaldense MSR-1	10.12 %
Rhodospirillum rubrum ATCC 11170	10.93 %
