# Supplementary material for: Azospirillum Genomes Reveal Transition of Bacteria from Aquatic to Terrestrial Environments
Source: PLoS Genet. 2011 Dec 22;7(12):e1002430. doi: 10.1371/journal.pgen.1002430 (PMC3245306; doi:10.1371/journal.pgen.1002430)
Supplement: Table S9 — Proteomic analysis of Azospirillum. (PDF) [file pgen.1002430.s012.pdf]

Table S9. Proteomic analysis of *Azospirillum*

Locus Tag (4B)	Control Condition	Nitrogen Fixation Condition	Locus Tag (Sp245)	Control Condition	Nitrogen Fixation Condition
AZOLI_0001	1: 0.00434, 2: 0.00903, 3: 0.00685, 4: 0.00601,	1: 0.01254, 2: 0.01002, 3: 0.00861, 4: 0.00796,	AZOBR_10006	1: 0.00010, 2: 0.00009, 3: 0.00011, 4: 0.00011, 5: 0.00011,	1: 0.00038, 2: 0.00024, 3: 0.00051,
AZOLI_0002	1: 0.00009, 2: 0.00007, 4: 0.00009,	1: 0.00004, 3: 0.00005,	AZOBR_10008	4: 0.00005,	
AZOLI_0005	2: 0.00009, 4: 0.00012,		AZOBR_10014	2: 0.00053, 4: 0.00028, 5: 0.00042,	2: 0.00088, 3: 0.00038,
AZOLI_0006	1: 0.00021, 2: 0.00016, 4: 0.00046,	1: 0.00070, 2: 0.00046, 3: 0.00074, 4: 0.00041,	AZOBR_10015	4: 0.00013,	
AZOLI_0008	2: 0.00024,	4: 0.00013,	AZOBR_10018	1: 0.00076, 2: 0.00048, 3: 0.00148, 4: 0.00037, 5: 0.00082,	2: 0.00032, 3: 0.00020,
AZOLI_0009	1: 0.00010, 4: 0.00022,		AZOBR_10019	1: 0.00205, 2: 0.00164, 3: 0.00158, 4: 0.00154, 5: 0.00128,	1: 0.00119, 2: 0.00108, 3: 0.00104,
AZOLI_0011	1: 0.00023, 2: 0.00026, 4: 0.00012,	1: 0.00012, 2: 0.00017, 3: 0.00009, 4: 0.00010,	AZOBR_10022	1: 0.00068, 2: 0.00088, 3: 0.00062, 4: 0.00094, 5: 0.00103,	1: 0.00049, 2: 0.00043, 3: 0.00056,
AZOLI_0024	1: 0.00008, 2: 0.00008,	1: 0.00019, 2: 0.00012, 3: 0.00020, 4: 0.00014,	AZOBR_10023	2: 0.00014, 4: 0.00011,	2: 0.00022, 3: 0.00014,
AZOLI_0026	2: 0.00044,	2: 0.00028, 4: 0.00025,	AZOBR_10024		1: 0.00128, 2: 0.00117, 3: 0.00212,
AZOLI_0028	1: 0.00021, 2: 0.00071, 3: 0.00111, 4: 0.00041,	1: 0.00048, 2: 0.00049, 3: 0.00056, 4: 0.00050,	AZOBR_10026	2: 0.00014, 3: 0.00020, 4: 0.00018, 5: 0.00016,	1: 0.01080, 2: 0.00806, 3: 0.00862,
AZOLI_0032		3: 0.00028, 4: 0.00022,	AZOBR_10027		1: 0.00048, 2: 0.00034, 3: 0.00069,
AZOLI_0033	1: 0.00007, 4: 0.00012,		AZOBR_10028	2: 0.00008, 3: 0.00008, 4: 0.00025, 5: 0.00019,	1: 0.00651, 2: 0.00458, 3: 0.00508,
AZOLI_0034	1: 0.00015, 4: 0.00030,	2: 0.00011, 4: 0.00010,	AZOBR_10031		3: 0.00008,
AZOLI_0036	1: 0.00019, 2: 0.00020, 3: 0.00022, 4: 0.00030,	3: 0.00017, 4: 0.00010,	AZOBR_10032	1: 0.00005, 3: 0.00005, 4: 0.00006, 5: 0.00004,	2: 0.00004, 3: 0.00002,
AZOLI_0049	2: 0.00020, 3: 0.00021, 4: 0.00009,	1: 0.00035, 2: 0.00010, 3: 0.00027,	AZOBR_10035	1: 0.00011, 2: 0.00016, 3: 0.00014, 4: 0.00007,	2: 0.00004,
AZOLI_0051	1: 0.00012, 2: 0.00018, 4: 0.00015,	1: 0.00018,	AZOBR_10036	2: 0.00112, 3: 0.00139, 4: 0.00112, 5: 0.00050,	
AZOLI_0054		2: 0.00008,	AZOBR_10037		1: 0.00011, 2: 0.00009, 3: 0.00011,
AZOLI_0056	2: 0.00019, 4: 0.00022,	3: 0.00021, 4: 0.00033,	AZOBR_10039	2: 0.00046, 3: 0.00045,	
AZOLI_0057	1: 0.00009, 2: 0.00011, 4: 0.00023,	2: 0.00007, 3: 0.00007,	AZOBR_10040	1: 0.00063, 2: 0.00110, 3: 0.00065, 4: 0.00095, 5: 0.00088,	1: 0.00098, 2: 0.00065, 3: 0.00068,
AZOLI_0060	1: 0.00014, 2: 0.00014, 4: 0.00032,	2: 0.00018, 3: 0.00020, 4: 0.00008,	AZOBR_10041		
AZOLI_0061	1: 0.00012, 2: 0.00012, 4: 0.00075,	3: 0.00012, 4: 0.00015,	AZOBR_10043	1: 0.00027, 2: 0.00012, 3: 0.00025, 4: 0.00027, 5: 0.00014,	1: 0.00008, 2: 0.00007, 3: 0.00006,
AZOLI_0064	1: 0.00006, 4: 0.00013,	2: 0.00006, 3: 0.00010, 4: 0.00013,	AZOBR_10045	3: 0.00012, 4: 0.00029, 5: 0.00013,	
AZOLI_0066	4: 0.00016,		AZOBR_10046		
AZOLI_0067	1: 0.00022, 2: 0.00031, 3: 0.00009, 4: 0.00019,	1: 0.00023, 2: 0.00026, 3: 0.00033, 4: 0.00030,	AZOBR_10047	4: 0.00070, 5: 0.00042,	3: 0.00051,
AZOLI_0069	1: 0.00007, 4: 0.00006,	4: 0.00009,	AZOBR_10049	1: 0.00027, 2: 0.00024, 3: 0.00036, 4: 0.00036, 5: 0.00022,	1: 0.00091, 2: 0.00129, 3: 0.00098,
AZOLI_0077	1: 0.00035, 2: 0.00045, 4: 0.00045,	1: 0.00068, 2: 0.00085, 3: 0.00096, 4: 0.00053,	AZOBR_10051		2: 0.00015, 3: 0.00026,
AZOLI_0082	1: 0.00031, 2: 0.00063, 4: 0.00120,	1: 0.00220, 2: 0.00106, 3: 0.00150, 4: 0.00080,	AZOBR_10052	1: 0.00077, 3: 0.00039, 4: 0.00074, 5: 0.00075,	1: 0.00020, 2: 0.00067, 3: 0.00086,
AZOLI_0086		1: 0.00024, 2: 0.00009, 3: 0.00010, 4: 0.00016,	AZOBR_10053	1: 0.00012, 2: 0.00019, 3: 0.00021, 4: 0.00019, 5: 0.00013,	1: 0.00034, 2: 0.00025, 3: 0.00050,
AZOLI_0087		1: 0.00052, 2: 0.00050, 3: 0.00042, 4: 0.00044,	AZOBR_10054	1: 0.00009, 2: 0.00012, 3: 0.00010, 4: 0.00014, 5: 0.00016,	1: 0.00054, 2: 0.00025, 3: 0.00044,
AZOLI_0088	1: 0.00195, 2: 0.00188, 3: 0.00282, 4: 0.00149,	1: 0.00203, 2: 0.00191, 3: 0.00229, 4: 0.00385,	AZOBR_10056	1: 0.00075, 2: 0.00050, 3: 0.00032, 4: 0.00010, 5: 0.00029,	1: 0.00018, 2: 0.00027, 3: 0.00041,
AZOLI_0089	1: 0.00011, 2: 0.00019, 3: 0.00072,	1: 0.00164, 2: 0.00151, 3: 0.00138, 4: 0.00162,	AZOBR_10058	1: 0.00008, 3: 0.00010,	
AZOLI_0090	3: 0.00011,	1: 0.00047, 2: 0.00058, 3: 0.00054, 4: 0.00052,	AZOBR_10060		
AZOLI_0096	2: 0.00008, 4: 0.00014,	1: 0.00008, 2: 0.00012, 4: 0.00025,	AZOBR_10061	3: 0.00006,	
AZOLI_0106	1: 0.00103, 2: 0.00225, 3: 0.00217, 4: 0.00281,	1: 0.00052, 2: 0.00234, 3: 0.00129, 4: 0.00148,	AZOBR_10062		2: 0.00007, 3: 0.00019,
AZOLI_0107	1: 0.00432, 2: 0.00343, 3: 0.00298, 4: 0.00381,	1: 0.00326, 2: 0.00305, 3: 0.00326, 4: 0.00246,	AZOBR_10063	3: 0.00007,	2: 0.00012, 3: 0.00005,
AZOLI_0108	1: 0.00035, 2: 0.00023, 3: 0.00040, 4: 0.00058,	1: 0.00048, 2: 0.00019, 3: 0.00058, 4: 0.00039,	AZOBR_10070		
AZOLI_0109	1: 0.00037, 2: 0.00063, 3: 0.00069, 4: 0.00055,	1: 0.00019, 2: 0.00026, 3: 0.00045, 4: 0.00050,	AZOBR_10071		3: 0.00008,
AZOLI_0111	2: 0.00029,	2: 0.00019, 3: 0.00031,	AZOBR_10074		1: 0.00002,
AZOLI_0113	4: 0.00009,		AZOBR_10081	1: 0.00110, 2: 0.00101, 3: 0.00082, 4: 0.00127, 5: 0.00076,	1: 0.00200, 2: 0.00161, 3: 0.00140,
AZOLI_0114	4: 0.00009,		AZOBR_10083	5: 0.00011,	
AZOLI_0118	1: 0.00040, 2: 0.00019, 3: 0.00008, 4: 0.00016,	1: 0.00029, 2: 0.00020, 3: 0.00018, 4: 0.00024,	AZOBR_10089		1: 0.00021, 2: 0.00024, 3: 0.00056,
AZOLI_0120		3: 0.00004,	AZOBR_10090	1: 0.00033, 3: 0.00011, 4: 0.00041, 5: 0.00050,	1: 0.00013,
AZOLI_0126	1: 0.00044, 4: 0.00038,		AZOBR_10093		1: 0.00017,
AZOLI_0131	1: 0.00015, 4: 0.00010,	3: 0.00008, 4: 0.00013,	AZOBR_10095	2: 0.00022, 4: 0.00022, 5: 0.00013,	
AZOLI_0134		2: 0.00011, 3: 0.00023, 4: 0.00014,	AZOBR_10096		

AZOLI_0135	3: 0.00051,	1: 0.00047, 2: 0.00046, 3: 0.00039, 4: 0.00022,	AZOBR_10097	3: 0.00015, 5: 0.00018,	1: 0.00033, 3: 0.00053,
AZOLI_0137	1: 0.00018, 2: 0.00018, 4: 0.00067,	1: 0.00031, 2: 0.00035, 3: 0.00039, 4: 0.00016,	AZOBR_10099	3: 0.00023, 4: 0.00009,	
AZOLI_0139		1: 0.00022, 3: 0.00016, 4: 0.00006,	AZOBR_10100	2: 0.00011,	2: 0.00009, 3: 0.00011,
AZOLI_0140	2: 0.00015, 3: 0.00262, 4: 0.00032,	1: 0.00121, 2: 0.00182, 3: 0.00176, 4: 0.00212,	AZOBR_10101		
AZOLI_0141	1: 0.00015, 2: 0.00013, 3: 0.00031, 4: 0.00016,	1: 0.00070, 2: 0.00059, 3: 0.00068, 4: 0.00066,	AZOBR_10102	1: 0.00007, 3: 0.00011, 4: 0.00007,	
AZOLI_0141	3: 0.00013,	1: 0.00045, 2: 0.00065, 3: 0.00043, 4: 0.00042,	AZOBR_10103	2: 0.00012, 4: 0.00024, 5: 0.00015,	
AZOLI_0142	1: 0.00025,	2: 0.00016, 3: 0.00009, 4: 0.00011,	AZOBR_10104	1: 0.00056, 2: 0.00104, 3: 0.00091, 4: 0.00053, 5: 0.00056,	1: 0.00079, 2: 0.00074, 3: 0.00067,
AZOLI_0145		1: 0.00018, 2: 0.00017, 3: 0.00013,	AZOBR_10105	1: 0.00011, 2: 0.00013, 3: 0.00027, 4: 0.00025, 5: 0.00022,	1: 0.00018, 2: 0.00010, 3: 0.00007,
AZOLI_0146	3: 0.00010,	1: 0.00011, 2: 0.00008, 3: 0.00004, 4: 0.00005,	AZOBR_10109		
AZOLI_0155	1: 0.00004,	2: 0.00010, 3: 0.00006,	AZOBR_10112		
AZOLI_0156	1: 0.00019, 2: 0.00013, 3: 0.00027, 4: 0.00055,	1: 0.00039, 2: 0.00044, 3: 0.00069, 4: 0.00022,	AZOBR_10113	4: 0.00016, 5: 0.00011,	1: 0.00015, 2: 0.00011, 3: 0.00025,
AZOLI_0157	1: 0.00027, 2: 0.00016, 4: 0.00069,		AZOBR_10114	1: 0.00013, 3: 0.00018, 5: 0.00012,	
AZOLI_0164	1: 0.00021, 2: 0.00007, 4: 0.00009,	4: 0.00006,	AZOBR_10116	3: 0.00022,	1: 0.00017, 2: 0.00013, 3: 0.00011,
AZOLI_0170	2: 0.00044, 3: 0.00037, 4: 0.00023,	1: 0.00053, 2: 0.00025, 3: 0.00037, 4: 0.00030,	AZOBR_10118	3: 0.00024, 4: 0.00023, 5: 0.00023,	1: 0.00011, 3: 0.00024,
AZOLI_0172	1: 0.00054, 2: 0.00045, 3: 0.00056, 4: 0.00045,	1: 0.00040, 2: 0.00063, 3: 0.00069, 4: 0.00036,	AZOBR_10124	1: 0.00149, 2: 0.00359, 3: 0.00127, 4: 0.00224, 5: 0.00234,	1: 0.00252, 2: 0.00277, 3: 0.00287,
AZOLI_0173	1: 0.00059, 2: 0.00103, 3: 0.00041, 4: 0.00071,	1: 0.00158, 2: 0.00143, 3: 0.00089, 4: 0.00059,	AZOBR_10128	1: 0.00011,	
AZOLI_0174	1: 0.00410, 2: 0.00460, 3: 0.00488, 4: 0.00378,	1: 0.00259, 2: 0.00235, 3: 0.00233, 4: 0.00291,	AZOBR_10131	1: 0.00116, 2: 0.00038, 3: 0.00124, 4: 0.00030, 5: 0.00020,	1: 0.00037, 2: 0.00085, 3: 0.00054,
AZOLI_0178	1: 0.00002, 4: 0.00002,		AZOBR_10132		3: 0.00007,
AZOLI_0179	2: 0.00005,		AZOBR_10134	1: 0.00067, 2: 0.00068, 3: 0.00082, 4: 0.00093, 5: 0.00056,	1: 0.00040, 2: 0.00056, 3: 0.00030,
AZOLI_0181	1: 0.00111, 2: 0.00095, 3: 0.00218, 4: 0.00116,	1: 0.00102, 2: 0.00139, 3: 0.00131, 4: 0.00157,	AZOBR_10135	4: 0.00034, 5: 0.00025,	
AZOLI_0192		2: 0.00003,	AZOBR_10141	1: 0.00063, 2: 0.00129, 3: 0.00049, 4: 0.00101, 5: 0.00119,	1: 0.00432, 2: 0.00316, 3: 0.00331,
AZOLI_0193	3: 0.00122,	1: 0.00387, 2: 0.00373, 3: 0.00611, 4: 0.00461,	AZOBR_10142		2: 0.00008, 3: 0.00007,
AZOLI_0194	4: 0.00028,	1: 0.00951, 2: 0.01136, 3: 0.01457, 4: 0.00838,	AZOBR_10146		
AZOLI_0199	1: 0.00033, 2: 0.00025, 3: 0.00183, 4: 0.00077,	1: 0.00173, 2: 0.00167, 3: 0.00166, 4: 0.00155,	AZOBR_10154		
AZOLI_0201	4: 0.00005,		AZOBR_10157		1: 0.00004, 2: 0.00004,
AZOLI_0204	1: 0.00007, 2: 0.00016, 3: 0.00063, 4: 0.00017,	1: 0.00044, 2: 0.00027, 3: 0.00038, 4: 0.00035,	AZOBR_10160	1: 0.00032, 2: 0.00056, 3: 0.00054, 4: 0.00044, 5: 0.00029,	1: 0.00020, 2: 0.00062, 3: 0.00119,
AZOLI_0205	1: 0.00163, 2: 0.00263, 3: 0.00772, 4: 0.00208,	1: 0.00237, 2: 0.00289, 3: 0.00238, 4: 0.00255,	AZOBR_10161	4: 0.00028, 5: 0.00021,	1: 0.00026, 2: 0.00059, 3: 0.00025,
AZOLI_0206	1: 0.00173, 2: 0.00152, 3: 0.00384, 4: 0.00243,	1: 0.00297, 2: 0.00244, 3: 0.00284, 4: 0.00297,	AZOBR_10162	4: 0.00005, 5: 0.00003,	1: 0.00015, 3: 0.00005,
AZOLI_0207	1: 0.00320, 2: 0.00287, 3: 0.01168, 4: 0.00321,	1: 0.00684, 2: 0.00618, 3: 0.00792, 4: 0.00701,	AZOBR_10163	1: 0.00032, 2: 0.00053, 3: 0.00063, 4: 0.00056, 5: 0.00066,	1: 0.00054, 2: 0.00051, 3: 0.00053,
AZOLI_0208	1: 0.00014, 2: 0.00035, 4: 0.00036,	1: 0.00063, 2: 0.00027, 3: 0.00067, 4: 0.00048,	AZOBR_10166	4: 0.00005,	
AZOLI_0212	1: 0.00013, 3: 0.00006, 4: 0.00004,	1: 0.00009, 3: 0.00005, 4: 0.00013,	AZOBR_10167	1: 0.00088, 2: 0.00120, 3: 0.00143, 4: 0.00055, 5: 0.00061,	1: 0.00077, 2: 0.00070, 3: 0.00074,
AZOLI_0215	2: 0.00014,		AZOBR_10172	4: 0.00010,	
AZOLI_0218	1: 0.00021, 2: 0.00020, 3: 0.00035, 4: 0.00018,	1: 0.00039, 2: 0.00049, 3: 0.00048, 4: 0.00041,	AZOBR_10173	2: 0.00007, 4: 0.00014, 5: 0.00025,	1: 0.00008, 2: 0.00006, 3: 0.00012,
AZOLI_0219	1: 0.00140, 2: 0.00167, 3: 0.00203, 4: 0.00251,	1: 0.00228, 2: 0.00196, 3: 0.00211, 4: 0.00300,	AZOBR_10174		3: 0.00013,
AZOLI_0220	1: 0.00019, 2: 0.00025, 3: 0.00077, 4: 0.00028,	1: 0.00022, 2: 0.00021, 3: 0.00027, 4: 0.00026,	AZOBR_10175	1: 0.00014, 2: 0.00009, 3: 0.00009, 4: 0.00012, 5: 0.00010,	1: 0.00005, 2: 0.00008,
AZOLI_0227	1: 0.00008, 2: 0.00030, 4: 0.00018,	1: 0.00009,	AZOBR_10176		
AZOLI_0229		4: 0.00012,	AZOBR_10178	2: 0.00006, 4: 0.00009,	
AZOLI_0230	3: 0.00024, 4: 0.00022,	1: 0.00047, 2: 0.00071, 3: 0.00046, 4: 0.00077,	AZOBR_10179		
AZOLI_0233	1: 0.00018, 4: 0.00038,		AZOBR_10180	1: 0.00012, 3: 0.00012, 4: 0.00010,	2: 0.00007, 3: 0.00006,
AZOLI_0236	2: 0.00034, 3: 0.00016, 4: 0.00025,	1: 0.00023, 2: 0.00022, 3: 0.00024, 4: 0.00020,	AZOBR_10184		3: 0.00011,
AZOLI_0237	3: 0.00009,	1: 0.00064, 2: 0.00054, 3: 0.00064, 4: 0.00057,	AZOBR_10185		1: 0.00004,
AZOLI_0241	1: 0.00259, 2: 0.00174, 3: 0.00086, 4: 0.00306,	1: 0.00077, 2: 0.00059, 3: 0.00114, 4: 0.00070,	AZOBR_10186		2: 0.00011, 3: 0.00019,
AZOLI_0252	1: 0.00018, 2: 0.00031, 3: 0.00025, 4: 0.00028,	1: 0.00010, 2: 0.00007, 3: 0.00008, 4: 0.00015,	AZOBR_10190	1: 0.00026, 3: 0.00022, 4: 0.00033, 5: 0.00021,	1: 0.00030, 2: 0.00022, 3: 0.00032,
AZOLI_0253	1: 0.00019, 2: 0.00029, 3: 0.00101, 4: 0.00045,	1: 0.00059, 2: 0.00064, 3: 0.00071, 4: 0.00084,	AZOBR_10191	1: 0.00029, 2: 0.00036, 3: 0.00035, 4: 0.00031, 5: 0.00017,	1: 0.00005, 2: 0.00009, 3: 0.00013,
AZOLI_0254	4: 0.00008,	4: 0.00008,	AZOBR_10192	1: 0.00319, 2: 0.00594, 3: 0.00312, 4: 0.00333, 5: 0.00497,	1: 0.00423, 2: 0.00258, 3: 0.00242,
AZOLI_0255	1: 0.00094, 2: 0.00048, 3: 0.00082, 4: 0.00053,	1: 0.00044, 2: 0.00060, 3: 0.00062, 4: 0.00069,	AZOBR_10193	1: 0.00704, 2: 0.00272, 3: 0.00880, 4: 0.00424, 5: 0.00470,	1: 0.00268, 2: 0.00224, 3: 0.00273,
AZOLI_0256	3: 0.00009,		AZOBR_10194		
AZOLI_0257	1: 0.00013,	1: 0.00020, 4: 0.00009,	AZOBR_10197		
AZOLI_0262	2: 0.00003, 4: 0.00002,		AZOBR_10202		3: 0.00022,
AZOLI_0264		4: 0.00021,	AZOBR_10204	1: 0.00040, 2: 0.00117, 3: 0.00079,	1: 0.00085, 2: 0.00086,

				4: 0.00091, 5: 0.00073,	3: 0.00131,
AZOLI_0272	1: 0.00015, 2: 0.00007, 4: 0.00038,	1: 0.00007, 3: 0.00012, 4: 0.00006,	AZOBR_10208		
AZOLI_0274	4: 0.00015,	2: 0.00014, 3: 0.00008, 4: 0.00006,	AZOBR_10209	2: 0.00045, 3: 0.00044, 4: 0.00021, 5: 0.00014,	1: 0.00295, 2: 0.00454, 3: 0.00489,
AZOLI_0275	1: 0.00056, 2: 0.00030, 3: 0.00017, 4: 0.00020,	1: 0.00033, 2: 0.00011, 3: 0.00060, 4: 0.00048,	AZOBR_10213	5: 0.00009,	1: 0.00032, 2: 0.00009, 3: 0.00012,
AZOLI_0277		4: 0.00010,	AZOBR_10214	1: 0.00022, 2: 0.00024, 3: 0.00015, 4: 0.00025, 5: 0.00015,	1: 0.00029, 2: 0.00042, 3: 0.00030,
AZOLI_0279	1: 0.00743, 2: 0.00646, 3: 0.01432, 4: 0.00778,	1: 0.01020, 2: 0.01082, 3: 0.01018, 4: 0.01185,	AZOBR_10215	1: 0.00025, 2: 0.00018, 3: 0.00029, 4: 0.00033, 5: 0.00024,	1: 0.00015, 2: 0.00008, 3: 0.00033,
AZOLI_0280	1: 0.00040, 2: 0.00056, 3: 0.00070, 4: 0.00039,	1: 0.00010, 2: 0.00034, 3: 0.00011, 4: 0.00086,	AZOBR_10216		1: 0.00005,
AZOLI_0281		1: 0.00010, 2: 0.00007, 3: 0.00007, 4: 0.00006,	AZOBR_10217		
AZOLI_0285	1: 0.00004, 4: 0.00005,	3: 0.00004, 4: 0.00006,	AZOBR_10218	1: 0.00020, 2: 0.00045, 3: 0.00034, 4: 0.00069, 5: 0.00042,	
AZOLI_0288	2: 0.00018, 3: 0.00037, 4: 0.00015,	1: 0.00026, 2: 0.00030, 3: 0.00019, 4: 0.00015,	AZOBR_10223		
AZOLI_0290	2: 0.00015,		AZOBR_10228	5: 0.00002,	
AZOLI_0294	1: 0.00027, 2: 0.00032, 4: 0.00020,	1: 0.00008, 2: 0.00019, 4: 0.00017,	AZOBR_10229		3: 0.00065,
AZOLI_0295	1: 0.00022, 2: 0.00013, 3: 0.00025, 4: 0.00023,	2: 0.00047, 3: 0.00024, 4: 0.00011,	AZOBR_10231		
AZOLI_0296	4: 0.00016,	2: 0.00012, 4: 0.00016,	AZOBR_10233	3: 0.00009, 4: 0.00005,	1: 0.00007,
AZOLI_0297	1: 0.00019, 2: 0.00012, 4: 0.00017,	1: 0.00016, 4: 0.00010,	AZOBR_10234	2: 0.00094, 3: 0.00083, 4: 0.00067, 5: 0.00079,	1: 0.00057, 2: 0.00058, 3: 0.00022,
AZOLI_0298	1: 0.00015, 4: 0.00026,		AZOBR_10235	1: 0.00048, 2: 0.00085, 3: 0.00074, 4: 0.00093, 5: 0.00053,	1: 0.00049, 2: 0.00042, 3: 0.00078,
AZOLI_0299	1: 0.00005, 2: 0.00005, 3: 0.00005, 4: 0.00017,	1: 0.00087, 2: 0.00087, 3: 0.00103, 4: 0.00106,	AZOBR_10236	1: 0.00187, 2: 0.00289, 3: 0.00198, 4: 0.00213, 5: 0.00210,	1: 0.00426, 2: 0.00335, 3: 0.00280,
AZOLI_0301		1: 0.00008, 3: 0.00011, 4: 0.00009,	AZOBR_10237	4: 0.00015,	3: 0.00017,
AZOLI_0305		1: 0.00012, 2: 0.00008, 4: 0.00007,	AZOBR_10238		
AZOLI_0308	2: 0.00008,	1: 0.00006, 3: 0.00004,	AZOBR_10243		3: 0.00007,
AZOLI_0309	2: 0.00036,	4: 0.00031,	AZOBR_10246	1: 0.00017, 2: 0.00015, 3: 0.00025, 4: 0.00029, 5: 0.00017,	1: 0.00040, 2: 0.00021, 3: 0.00057,
AZOLI_0311		4: 0.00007,	AZOBR_10251		
AZOLI_0313	2: 0.00010, 4: 0.00016,	1: 0.00019, 2: 0.00018, 4: 0.00020,	AZOBR_10254		
AZOLI_0316	4: 0.00042,		AZOBR_10256		1: 0.00050, 2: 0.00072, 3: 0.00089,
AZOLI_0318	1: 0.00139, 2: 0.00089, 3: 0.00054, 4: 0.00066,	1: 0.00034, 2: 0.00016, 3: 0.00045, 4: 0.00051,	AZOBR_10258	3: 0.00020, 4: 0.00012,	1: 0.00057, 2: 0.00107, 3: 0.00073,
AZOLI_0319	1: 0.00066, 2: 0.00066, 4: 0.00085,	4: 0.00028,	AZOBR_10259		
AZOLI_0320	1: 0.00014, 2: 0.00015,		AZOBR_10262	1: 0.00047, 2: 0.00057, 3: 0.00061, 4: 0.00035, 5: 0.00025,	1: 0.00009, 2: 0.00018, 3: 0.00009,
AZOLI_0321		3: 0.00005,	AZOBR_10263	4: 0.00007, 5: 0.00004,	
AZOLI_0322	1: 0.00006, 2: 0.00008, 3: 0.00033, 4: 0.00007,	1: 0.00027, 2: 0.00034, 3: 0.00027, 4: 0.00024,	AZOBR_10267	4: 0.00025,	1: 0.00070,
AZOLI_0327	1: 0.00061, 2: 0.00028, 3: 0.00055, 4: 0.00038,	1: 0.00034, 2: 0.00060, 3: 0.00042, 4: 0.00029,	AZOBR_10273		1: 0.00011, 3: 0.00010,
AZOLI_0340	1: 0.00024, 2: 0.00033, 3: 0.00080, 4: 0.00030,	1: 0.00091, 2: 0.00055, 3: 0.00128, 4: 0.00138,	AZOBR_10275		
AZOLI_0346	2: 0.00009, 4: 0.00005,		AZOBR_10276	1: 0.00011, 2: 0.00023, 3: 0.00028, 4: 0.00013, 5: 0.00008,	1: 0.00021, 2: 0.00013, 3: 0.00030,
AZOLI_0347	1: 0.00014, 2: 0.00028, 4: 0.00051,	1: 0.00107, 2: 0.00113, 3: 0.00132, 4: 0.00097,	AZOBR_10277	3: 0.00005,	2: 0.00006,
AZOLI_0348	1: 0.00303, 2: 0.00251, 3: 0.00389, 4: 0.00595,	1: 0.00252, 2: 0.00296, 3: 0.00178, 4: 0.00119,	AZOBR_10279	1: 0.01300, 2: 0.00984, 3: 0.01150, 4: 0.01860, 5: 0.01409,	1: 0.00314, 2: 0.00295, 3: 0.00629,
AZOLI_0350	1: 0.00005, 2: 0.00005,		AZOBR_10280	1: 0.00039, 2: 0.00020, 3: 0.00048, 4: 0.00027, 5: 0.00023,	1: 0.00011, 2: 0.00033, 3: 0.00049,
AZOLI_0357		1: 0.00010, 2: 0.00013, 3: 0.00012, 4: 0.00021,	AZOBR_10281	4: 0.00012,	1: 0.00017, 2: 0.00013, 3: 0.00032,
AZOLI_0359	1: 0.00010, 3: 0.00019, 4: 0.00011,	1: 0.00010, 2: 0.00013, 3: 0.00025, 4: 0.00026,	AZOBR_10285	4: 0.00007,	2: 0.00008, 3: 0.00007,
AZOLI_0361	1: 0.00009, 2: 0.00006, 4: 0.00008,		AZOBR_10286	1: 0.00006,	1: 0.00004,
AZOLI_0362	1: 0.00024, 2: 0.00033, 4: 0.00049,	1: 0.00086, 2: 0.00036, 3: 0.00074, 4: 0.00042,	AZOBR_10287		1: 0.00034, 2: 0.00013, 3: 0.00022,
AZOLI_0363	1: 0.00043, 2: 0.00048, 4: 0.00037,	3: 0.00030, 4: 0.00012,	AZOBR_10289	1: 0.00424, 2: 0.00450, 3: 0.00414, 4: 0.00265, 5: 0.00357,	1: 0.00206, 2: 0.00297, 3: 0.00156,
AZOLI_0365	3: 0.00006,		AZOBR_10290	2: 0.00008,	1: 0.00006, 3: 0.00009,
AZOLI_0366	1: 0.00013, 2: 0.00013, 4: 0.00009,	1: 0.00018, 2: 0.00027, 3: 0.00014, 4: 0.00022,	AZOBR_10291	1: 0.00003, 3: 0.00004, 4: 0.00002,	2: 0.00008, 3: 0.00005,
AZOLI_0367	2: 0.00022, 4: 0.00060,	1: 0.00011, 2: 0.00011, 4: 0.00023,	AZOBR_10293	1: 0.00031, 2: 0.00027, 3: 0.00019, 4: 0.00052, 5: 0.00027,	2: 0.00022, 3: 0.00019,
AZOLI_0369	1: 0.00005,	1: 0.00005, 2: 0.00005, 4: 0.00009,	AZOBR_10294	1: 0.00043, 2: 0.00054, 3: 0.00044, 4: 0.00042,	1: 0.00017, 2: 0.00013, 3: 0.00016,
AZOLI_0371	1: 0.01332, 2: 0.00658, 3: 0.00500, 4: 0.00860,	1: 0.00613, 2: 0.00590, 3: 0.00772, 4: 0.00538,	AZOBR_10296	1: 0.00021, 3: 0.00018, 4: 0.00014,	1: 0.00020, 2: 0.00015,
AZOLI_0372		4: 0.00015,	AZOBR_10298	1: 0.00034, 2: 0.00050, 3: 0.00058, 4: 0.00060, 5: 0.00052,	1: 0.00020, 2: 0.00014, 3: 0.00031,
AZOLI_0373	1: 0.00025, 2: 0.00008, 4: 0.00007,	1: 0.00013, 3: 0.00014, 4: 0.00018,	AZOBR_10300		
AZOLI_0374	1: 0.00259, 2: 0.00270, 3: 0.00170, 4: 0.00521,	1: 0.00210, 2: 0.00371, 3: 0.00222, 4: 0.00284,	AZOBR_10304		2: 0.00019,
AZOLI_0376	4: 0.00015,		AZOBR_10306	1: 0.00134, 2: 0.00094, 3: 0.00075,	1: 0.00054, 2: 0.00054,

				4: 0.00109, 5: 0.00094,	3: 0.00088,
AZOLI_0378	1: 0.00008, 2: 0.00012, 4: 0.00010,		AZOBR_10308	1: 0.00571, 2: 0.00418, 3: 0.00583, 4: 0.00595, 5: 0.00652,	1: 0.00272, 2: 0.00404, 3: 0.00405,
AZOLI_0379	1: 0.00015, 2: 0.00015, 3: 0.00042, 4: 0.00021,		AZOBR_10309	3: 0.00028, 4: 0.00023, 5: 0.00018,	
AZOLI_0381	1: 0.00026, 2: 0.00034, 3: 0.00043, 4: 0.00042,	1: 0.00092, 2: 0.00144, 3: 0.00089, 4: 0.00075,	AZOBR_10310	2: 0.00013, 3: 0.00032, 4: 0.00021, 5: 0.00010,	2: 0.00011, 3: 0.00014,
AZOLI_0384	1: 0.00006, 2: 0.00010, 4: 0.00014,		AZOBR_10311	1: 0.00107, 2: 0.00124, 3: 0.00104, 4: 0.00136, 5: 0.00114,	1: 0.00088, 2: 0.00079, 3: 0.00108,
AZOLI_0386	1: 0.00054, 2: 0.00052, 3: 0.00043, 4: 0.00046,	1: 0.00026, 2: 0.00041, 3: 0.00042, 4: 0.00035,	AZOBR_10312	2: 0.00016, 3: 0.00016, 4: 0.00033, 5: 0.00029,	1: 0.00008, 2: 0.00031, 3: 0.00023,
AZOLI_0388	4: 0.00007,	3: 0.00004, 4: 0.00002,	AZOBR_10313	1: 0.00145, 2: 0.00093, 3: 0.00109, 4: 0.00151, 5: 0.00131,	1: 0.00109, 2: 0.00095, 3: 0.00108,
AZOLI_0392	1: 0.00070, 2: 0.00089, 3: 0.00030, 4: 0.00194,	1: 0.00143, 2: 0.00086, 3: 0.00158, 4: 0.00127,	AZOBR_10314	2: 0.00024, 4: 0.00019, 5: 0.00025,	1: 0.00017, 2: 0.00020, 3: 0.00050,
AZOLI_0393	1: 0.00031,	1: 0.00053,	AZOBR_10316		2: 0.00015, 3: 0.00037,
AZOLI_0395	1: 0.00017, 4: 0.00007,	3: 0.00014, 4: 0.00022,	AZOBR_10319	2: 0.00010, 3: 0.00010, 4: 0.00027,	1: 0.00015, 2: 0.00012, 3: 0.00014,
AZOLI_0397		4: 0.00018,	AZOBR_10320	1: 0.00366, 2: 0.00221, 3: 0.00229, 4: 0.00162, 5: 0.00185,	1: 0.00105, 2: 0.00117, 3: 0.00118,
AZOLI_0400	4: 0.00007,		AZOBR_10322	1: 0.00296, 2: 0.00301, 3: 0.00305, 4: 0.00327, 5: 0.00371,	3: 0.00011,
AZOLI_0410	1: 0.00009, 4: 0.00015,	1: 0.00022, 3: 0.00033,	AZOBR_10324		3: 0.00006,
AZOLI_0412	1: 0.00052, 2: 0.00040, 3: 0.00083, 4: 0.00042,	1: 0.00110, 2: 0.00131, 3: 0.00102, 4: 0.00105,	AZOBR_10326	2: 0.00010, 3: 0.00010, 4: 0.00011, 5: 0.00014,	1: 0.00018, 2: 0.00009, 3: 0.00024,
AZOLI_0416	1: 0.00032, 2: 0.00049, 4: 0.00040,	1: 0.00055, 2: 0.00055, 3: 0.00055, 4: 0.00028,	AZOBR_10327	1: 0.00009, 2: 0.00006, 3: 0.00011, 4: 0.00022, 5: 0.00013,	1: 0.00014, 2: 0.00011, 3: 0.00006,
AZOLI_0417		1: 0.00024, 2: 0.00030, 3: 0.00058, 4: 0.00027,	AZOBR_10330		
AZOLI_0421	2: 0.00005,		AZOBR_10331	1: 0.00074, 2: 0.00084, 3: 0.00090, 4: 0.00051, 5: 0.00116,	1: 0.00088, 2: 0.00092, 3: 0.00085,
AZOLI_0436	1: 0.00008, 2: 0.00008, 4: 0.00020,	1: 0.00012, 2: 0.00007, 3: 0.00028, 4: 0.00010,	AZOBR_10333		
AZOLI_0439	1: 0.00928, 2: 0.01041, 3: 0.00545, 4: 0.00653,	1: 0.00464, 2: 0.00597, 3: 0.00404, 4: 0.00449,	AZOBR_10335		
AZOLI_0439	1: 0.00991, 2: 0.01113, 3: 0.00632, 4: 0.00707,	1: 0.00490, 2: 0.00630, 3: 0.00426, 4: 0.00474,	AZOBR_10337	3: 0.00007, 4: 0.00006,	1: 0.00005, 3: 0.00010,
AZOLI_0443	1: 0.00521, 2: 0.00649, 3: 0.00425, 4: 0.00244,	1: 0.00261, 2: 0.00326, 3: 0.00310, 4: 0.00217,	AZOBR_10338	2: 0.00028, 3: 0.00027, 4: 0.00027, 5: 0.00027,	1: 0.00035, 3: 0.00020,
AZOLI_0445	1: 0.00456, 2: 0.01078, 3: 0.00664, 4: 0.00623,	1: 0.00494, 2: 0.00437, 3: 0.00548, 4: 0.00604,	AZOBR_10341		1: 0.00026, 2: 0.00041, 3: 0.00050,
AZOLI_0446	1: 0.01083, 2: 0.01041, 3: 0.00861, 4: 0.00761,	1: 0.00644, 2: 0.00447, 3: 0.00476, 4: 0.00524,	AZOBR_10344	1: 0.00214, 2: 0.00093, 3: 0.00101, 4: 0.00169, 5: 0.00081,	1: 0.00073, 2: 0.00099, 3: 0.00035,
AZOLI_0448	1: 0.01138, 2: 0.00952, 3: 0.00934, 4: 0.00874,	1: 0.00732, 2: 0.00698, 3: 0.00607, 4: 0.00775,	AZOBR_10346	1: 0.00016, 2: 0.00038, 3: 0.00041, 4: 0.00026, 5: 0.00033,	2: 0.00027, 3: 0.00013,
AZOLI_0449	1: 0.01918, 2: 0.01361, 3: 0.02409, 4: 0.01717,	1: 0.01634, 2: 0.01246, 3: 0.01836, 4: 0.01525,	AZOBR_10347		
AZOLI_0450	1: 0.00067, 2: 0.00056, 3: 0.00055, 4: 0.00085,	1: 0.00117, 2: 0.00095, 3: 0.00127, 4: 0.00095,	AZOBR_10350	1: 0.00011, 4: 0.00023,	1: 0.00025, 3: 0.00031,
AZOLI_0451	1: 0.00037, 2: 0.00072, 3: 0.00050, 4: 0.00068,	1: 0.00063, 2: 0.00073, 3: 0.00070, 4: 0.00066,	AZOBR_10351	2: 0.00016, 3: 0.00019, 5: 0.00018,	1: 0.00045, 2: 0.00071, 3: 0.00058,
AZOLI_0455	1: 0.00736, 2: 0.00919, 3: 0.00596, 4: 0.00428,	1: 0.00382, 2: 0.00251, 3: 0.00306, 4: 0.00334,	AZOBR_10356	1: 0.00040, 2: 0.00041, 3: 0.00034, 4: 0.00082, 5: 0.00089,	1: 0.00038, 2: 0.00029, 3: 0.00039,
AZOLI_0456	1: 0.00660, 2: 0.00586, 3: 0.00562, 4: 0.00556,	1: 0.00353, 2: 0.00389, 3: 0.00234, 4: 0.00432,	AZOBR_10357	2: 0.00010, 3: 0.00019, 4: 0.00019,	1: 0.00014, 3: 0.00010,
AZOLI_0457	1: 0.00005, 4: 0.00011,	1: 0.00012, 2: 0.00010, 3: 0.00012, 4: 0.00014,	AZOBR_10358	1: 0.00006, 3: 0.00005, 4: 0.00010,	
AZOLI_0460	1: 0.00266, 2: 0.00235, 3: 0.00484, 4: 0.00287,	1: 0.00202, 2: 0.00097, 3: 0.00214, 4: 0.00249,	AZOBR_10359		3: 0.00008,
AZOLI_0461	1: 0.01297, 2: 0.01147, 3: 0.00659, 4: 0.00622,	1: 0.00579, 2: 0.00738, 3: 0.00612, 4: 0.00661,	AZOBR_10363	4: 0.00004,	1: 0.00004, 3: 0.00005,
AZOLI_0462	1: 0.01234, 2: 0.00847, 3: 0.01014, 4: 0.00924,	1: 0.00626, 2: 0.00592, 3: 0.00622, 4: 0.00589,	AZOBR_10366	1: 0.00059, 2: 0.00060, 3: 0.00072, 4: 0.00052, 5: 0.00063,	1: 0.00017, 2: 0.00021, 3: 0.00020,
AZOLI_0463	1: 0.01239, 2: 0.01196, 3: 0.00319, 4: 0.00390,	1: 0.00350, 2: 0.00452, 3: 0.00197, 4: 0.00447,	AZOBR_10367	1: 0.00016, 3: 0.00027, 4: 0.00038, 5: 0.00022,	
AZOLI_0464	1: 0.00539, 2: 0.00502, 3: 0.00660, 4: 0.00450,	1: 0.00254, 2: 0.00241, 3: 0.00225, 4: 0.00259,	AZOBR_10368		
AZOLI_0465	1: 0.01266, 2: 0.01092, 3: 0.00762, 4: 0.00647,	1: 0.01023, 2: 0.01165, 3: 0.00990, 4: 0.00882,	AZOBR_10369	4: 0.00024, 5: 0.00024,	1: 0.00018, 2: 0.00010, 3: 0.00009,
AZOLI_0466	1: 0.00915, 2: 0.00680, 3: 0.00584, 4: 0.00789,	1: 0.00701, 2: 0.00658, 3: 0.00602, 4: 0.00552,	AZOBR_10370	1: 0.00061, 2: 0.00078, 3: 0.00116, 4: 0.00042, 5: 0.00033,	1: 0.00035, 2: 0.00025, 3: 0.00017,
AZOLI_0467	1: 0.00273, 2: 0.00449, 3: 0.00397, 4: 0.00266,	1: 0.00230, 2: 0.00204, 3: 0.00219, 4: 0.00283,	AZOBR_10371	1: 0.00024, 2: 0.00050, 3: 0.00041, 4: 0.00011,	3: 0.00035,
AZOLI_0468	1: 0.00253, 2: 0.00319, 3: 0.00223, 4: 0.00314,	1: 0.00217, 2: 0.00286, 3: 0.00170, 4: 0.00342,	AZOBR_10372	1: 0.00012, 2: 0.00031, 4: 0.00040, 5: 0.00024,	1: 0.00030, 2: 0.00017, 3: 0.00033,
AZOLI_0469	1: 0.00383, 2: 0.00285, 3: 0.00445, 4: 0.00717,	1: 0.00489, 2: 0.00406, 3: 0.00321, 4: 0.00604,	AZOBR_10373	1: 0.00055, 2: 0.00082, 3: 0.00065, 4: 0.00041, 5: 0.00034,	1: 0.00025, 2: 0.00060, 3: 0.00048,
AZOLI_0470	1: 0.00835, 2: 0.00992, 3: 0.00398, 4: 0.00673,	1: 0.00423, 2: 0.00644, 3: 0.00260, 4: 0.00221,	AZOBR_10374	1: 0.00103, 3: 0.00142, 4: 0.00044, 5: 0.00053,	1: 0.00033, 2: 0.00103,
AZOLI_0471	1: 0.00454, 2: 0.00365, 3: 0.00366, 4: 0.00392,	1: 0.00179, 2: 0.00154, 3: 0.00100, 4: 0.00144,	AZOBR_10375	1: 0.00214, 2: 0.00242, 3: 0.00203, 4: 0.00147, 5: 0.00205,	1: 0.00030, 2: 0.00130, 3: 0.00044,
AZOLI_0472	1: 0.00840, 2: 0.01142, 3: 0.01123, 4: 0.00938,	1: 0.00710, 2: 0.00684, 3: 0.00694, 4: 0.00596,	AZOBR_10377		
AZOLI_0473	1: 0.01872, 2: 0.01465, 3: 0.00558, 4: 0.01414,	1: 0.00784, 2: 0.00516, 3: 0.00735, 4: 0.00538,	AZOBR_10378		
AZOLI_0474	1: 0.00358, 2: 0.00396, 3: 0.00268, 4: 0.00512,	1: 0.00273, 2: 0.00219, 3: 0.00289, 4: 0.00310,	AZOBR_10380	1: 0.00014,	
AZOLI_0475	1: 0.00322, 2: 0.00430, 3: 0.00527, 4: 0.00668,	1: 0.00229, 2: 0.00297, 3: 0.00262, 4: 0.00383,	AZOBR_10381	1: 0.00026, 2: 0.00047, 3: 0.00030, 4: 0.00018, 5: 0.00103,	1: 0.00028, 2: 0.00038, 3: 0.00066,
AZOLI_0476	1: 0.00780, 2: 0.00652,	1: 0.00382, 2: 0.00356,	AZOBR_10382	1: 0.00083, 2: 0.00074, 3: 0.00059,	1: 0.00024, 2: 0.00030,

	3: 0.00846, 4: 0.00777,	3: 0.00370, 4: 0.00353,		4: 0.00079, 5: 0.00050,	3: 0.00063,
AZOLI_0477	1: 0.00537, 2: 0.00866, 3: 0.00571, 4: 0.00927,	1: 0.00650, 2: 0.00718, 3: 0.00880, 4: 0.00725,	AZOBR_10384		3: 0.00002,
AZOLI_0478	1: 0.00766, 2: 0.00326, 3: 0.00607, 4: 0.00495,	1: 0.00297, 2: 0.00456, 3: 0.00263, 4: 0.00424,	AZOBR_10388	1: 0.00029, 3: 0.00021, 4: 0.00009, 5: 0.00011,	3: 0.00008,
AZOLI_0479	1: 0.00503, 2: 0.00707, 3: 0.00733, 4: 0.00635,	1: 0.00437, 2: 0.00526, 3: 0.00424, 4: 0.00558,	AZOBR_10390	1: 0.00015, 3: 0.00013, 4: 0.00007,	2: 0.00015,
AZOLI_0480	1: 0.00414, 2: 0.00426, 3: 0.00366, 4: 0.00509,	1: 0.00420, 2: 0.00295, 3: 0.00505, 4: 0.00401,	AZOBR_10391	4: 0.00015, 5: 0.00010,	
AZOLI_0482	1: 0.00200, 2: 0.00175, 3: 0.00400, 4: 0.00200,	1: 0.00267, 2: 0.00313, 3: 0.00373, 4: 0.00418,	AZOBR_10392	1: 0.00047, 2: 0.00023, 3: 0.00031, 4: 0.00018, 5: 0.00018,	1: 0.00010, 2: 0.00030, 3: 0.00013,
AZOLI_0483	1: 0.00009, 2: 0.00031, 4: 0.00030,	1: 0.00022, 2: 0.00021, 3: 0.00019, 4: 0.00030,	AZOBR_10393	2: 0.00012,	
AZOLI_0486	1: 0.00008,		AZOBR_10395	2: 0.00020, 3: 0.00023, 4: 0.00009, 5: 0.00015,	1: 0.00014, 2: 0.00016, 3: 0.00014,
AZOLI_0488		1: 0.00008, 2: 0.00005, 4: 0.00009,	AZOBR_10396		2: 0.00037, 3: 0.00016,
AZOLI_0489	1: 0.00008,	1: 0.00021,	AZOBR_10397		
AZOLI_0490	4: 0.00005,		AZOBR_10398		1: 0.00067, 2: 0.00076, 3: 0.00087,
AZOLI_0507		2: 0.00007, 4: 0.00012,	AZOBR_10399	4: 0.00017,	1: 0.00016, 3: 0.00038,
AZOLI_0508	1: 0.00030, 2: 0.00038, 4: 0.00020,	2: 0.00030,	AZOBR_10400		
AZOLI_0516	3: 0.00301, 4: 0.00018,	1: 0.00487, 2: 0.00417, 3: 0.00515, 4: 0.00516,	AZOBR_10401		2: 0.00003,
AZOLI_0517		4: 0.00011,	AZOBR_10402	1: 0.00021, 3: 0.00018, 4: 0.00015,	1: 0.00018, 2: 0.00021,
AZOLI_0519	1: 0.00018, 2: 0.00022, 4: 0.00023,	1: 0.00036, 2: 0.00039, 3: 0.00033, 4: 0.00023,	AZOBR_10403	1: 0.00022, 3: 0.00014,	
AZOLI_0520	1: 0.00170, 2: 0.00157, 3: 0.00498, 4: 0.00244,	1: 0.00272, 2: 0.00387, 3: 0.00258, 4: 0.00183,	AZOBR_10404	1: 0.00019, 2: 0.00042, 3: 0.00033, 4: 0.00056, 5: 0.00043,	2: 0.00031, 3: 0.00027,
AZOLI_0534	1: 0.00009, 2: 0.00012, 4: 0.00004,	1: 0.00015, 2: 0.00009, 3: 0.00008, 4: 0.00009,	AZOBR_10406		
AZOLI_0535		1: 0.00280, 2: 0.00219, 3: 0.00373, 4: 0.00338,	AZOBR_10408	1: 0.00010, 3: 0.00010, 4: 0.00005, 5: 0.00005,	1: 0.00012, 2: 0.00006, 3: 0.00013,
AZOLI_0536		1: 0.00125, 2: 0.00188, 3: 0.00169, 4: 0.00131,	AZOBR_10409	4: 0.00011,	2: 0.00012,
AZOLI_0537		1: 0.00225, 2: 0.00207, 3: 0.00179, 4: 0.00190,	AZOBR_10410	3: 0.00006, 4: 0.00010, 5: 0.00005,	
AZOLI_0538		1: 0.00237, 2: 0.00171, 3: 0.00188, 4: 0.00131,	AZOBR_10413		
AZOLI_0540	1: 0.00023, 3: 0.00032, 4: 0.00039,	1: 0.00103, 2: 0.00088, 3: 0.00134, 4: 0.00088,	AZOBR_10414	1: 0.00039, 2: 0.00028, 3: 0.00033, 4: 0.00086, 5: 0.00075,	1: 0.00060, 2: 0.00051, 3: 0.00087,
AZOLI_0541		1: 0.00062, 2: 0.00060, 3: 0.00055, 4: 0.00079,	AZOBR_10415	3: 0.00026, 4: 0.00017, 5: 0.00009,	1: 0.00044, 2: 0.00022, 3: 0.00038,
AZOLI_0542		2: 0.00020,	AZOBR_10418	4: 0.00010, 5: 0.00010,	
AZOLI_0544		1: 0.00106, 2: 0.00138, 3: 0.00194, 4: 0.00178,	AZOBR_10423	3: 0.00015,	
AZOLI_0545		1: 0.00168, 2: 0.00245, 3: 0.00309, 4: 0.00242,	AZOBR_10425		
AZOLI_0546		2: 0.00025,	AZOBR_10430		
AZOLI_0548		1: 0.00020, 2: 0.00019, 3: 0.00043,	AZOBR_10432		1: 0.00010,
AZOLI_0549	1: 0.00015, 4: 0.00013,	3: 0.00006, 4: 0.00016,	AZOBR_10434		
AZOLI_0552	1: 0.00112, 2: 0.00103, 3: 0.00144, 4: 0.00123,	1: 0.00196, 2: 0.00179, 3: 0.00186, 4: 0.00203,	AZOBR_10436		
AZOLI_0553	1: 0.00129, 2: 0.00159, 3: 0.00061, 4: 0.00284,	1: 0.00174, 2: 0.00336, 3: 0.00246, 4: 0.00272,	AZOBR_10439		
AZOLI_0554	1: 0.00008, 2: 0.00016, 3: 0.00052, 4: 0.00023,	1: 0.00005, 2: 0.00018, 3: 0.00006, 4: 0.00016,	AZOBR_10440		
AZOLI_0557	1: 0.00004,	2: 0.00004, 4: 0.00004,	AZOBR_10441	3: 0.00009,	
AZOLI_0558	1: 0.00013, 2: 0.00008, 4: 0.00011,	3: 0.00005, 4: 0.00004,	AZOBR_10442	1: 0.00032, 2: 0.00040, 3: 0.00049, 4: 0.00053, 5: 0.00026,	1: 0.00016, 2: 0.00033, 3: 0.00020,
AZOLI_0565	2: 0.00007, 4: 0.00006,	1: 0.00033, 2: 0.00007, 3: 0.00016, 4: 0.00013,	AZOBR_10444	1: 0.00062, 2: 0.00141, 3: 0.00106, 4: 0.00134, 5: 0.00085,	1: 0.00130, 2: 0.00090, 3: 0.00083,
AZOLI_0566		3: 0.00010,	AZOBR_10445	2: 0.00010,	1: 0.00015, 3: 0.00007,
AZOLI_0568	1: 0.00048, 2: 0.00049, 4: 0.00073,	1: 0.00067, 2: 0.00035, 3: 0.00065, 4: 0.00042,	AZOBR_10448		
AZOLI_0572		4: 0.00004,	AZOBR_10453		
AZOLI_0574	3: 0.00079, 4: 0.00039,	2: 0.00066, 4: 0.00048,	AZOBR_10454		2: 0.00025,
AZOLI_0577		2: 0.00008,	AZOBR_10455	4: 0.00044, 5: 0.00044,	3: 0.00017,
AZOLI_0578	1: 0.00019, 2: 0.00038, 3: 0.00016, 4: 0.00035,	1: 0.00053, 2: 0.00042, 3: 0.00050, 4: 0.00029,	AZOBR_10458	1: 0.00022, 2: 0.00008, 3: 0.00011, 4: 0.00033, 5: 0.00021,	1: 0.00017, 2: 0.00016, 3: 0.00022,
AZOLI_0586	1: 0.00022, 2: 0.00022, 4: 0.00031,	1: 0.00095, 2: 0.00099, 3: 0.00070, 4: 0.00149,	AZOBR_10461	1: 0.00186, 2: 0.00159, 3: 0.00178, 4: 0.00180, 5: 0.00184,	1: 0.00149, 2: 0.00207, 3: 0.00198,
AZOLI_0588	1: 0.00293, 2: 0.00202, 3: 0.00853, 4: 0.00384,	1: 0.00371, 2: 0.00448, 3: 0.00557, 4: 0.00995,	AZOBR_10466	3: 0.00003,	3: 0.00002,
AZOLI_0589	1: 0.00027, 2: 0.00009, 4: 0.00012,		AZOBR_10469		
AZOLI_0592		4: 0.00012,	AZOBR_10471	5: 0.00018,	2: 0.00012, 3: 0.00011,
AZOLI_0595		3: 0.00005, 4: 0.00004,	AZOBR_10473	1: 0.00011, 3: 0.00022, 4: 0.00015, 5: 0.00022,	2: 0.00013, 3: 0.00009,
AZOLI_0598	1: 0.00009, 2: 0.00033, 3: 0.00049, 4: 0.00024,	2: 0.00032, 3: 0.00020, 4: 0.00026,	AZOBR_10474	4: 0.00012, 5: 0.00018,	3: 0.00016,
AZOLI_0599	4: 0.00012,	4: 0.00005,	AZOBR_10475		1: 0.00005, 2: 0.00008, 3: 0.00009,
AZOLI_0602	1: 0.00010, 2: 0.00016, 4: 0.00014,	1: 0.00006, 2: 0.00006, 3: 0.00015, 4: 0.00016,	AZOBR_10476	1: 0.00016,	1: 0.00017, 3: 0.00010,
AZOLI_0605	1: 0.00010, 4: 0.00019,	4: 0.00004,	AZOBR_10479	1: 0.00014, 3: 0.00016, 4: 0.00025, 5: 0.00009,	1: 0.00009, 2: 0.00027, 3: 0.00020,
AZOLI_0606	2: 0.00005, 4: 0.00013,		AZOBR_10483		1: 0.00015, 3: 0.00003,

AZOLI_0607	2: 0.00017, 4: 0.00010,		AZOBR_10485	4: 0.00025, 5: 0.00015,	2: 0.00011, 3: 0.00009,
AZOLI_0609	2: 0.00002, 4: 0.00004,	1: 0.00010, 2: 0.00007, 3: 0.00008, 4: 0.00003,	AZOBR_10486	1: 0.00067, 2: 0.00107, 3: 0.00047, 4: 0.00054, 5: 0.00042,	1: 0.00051, 2: 0.00031, 3: 0.00034,
AZOLI_0615	1: 0.00025, 2: 0.00015, 4: 0.00009,	2: 0.00010,	AZOBR_10487		
AZOLI_0616		1: 0.00021, 2: 0.00020, 3: 0.00015, 4: 0.00053,	AZOBR_10488	1: 0.00091, 2: 0.00192, 3: 0.00137, 4: 0.00135, 5: 0.00242,	1: 0.00148, 2: 0.00102, 3: 0.00187,
AZOLI_0617	1: 0.00006, 4: 0.00007,	2: 0.00004, 3: 0.00006, 4: 0.00005,	AZOBR_10489	1: 0.00048, 2: 0.00032, 3: 0.00041, 4: 0.00050, 5: 0.00074,	2: 0.00035, 3: 0.00037,
AZOLI_0618		1: 0.00004,	AZOBR_10491	1: 0.00017, 4: 0.00041, 5: 0.00035,	3: 0.00016,
AZOLI_0619	1: 0.00018, 2: 0.00037, 3: 0.00077, 4: 0.00035,	1: 0.00076, 2: 0.00062, 3: 0.00124, 4: 0.00106,	AZOBR_10493	1: 0.00166, 2: 0.00197, 3: 0.00128, 4: 0.00206, 5: 0.00185,	1: 0.00105, 2: 0.00081, 3: 0.00119,
AZOLI_0620	1: 0.00241, 2: 0.00228, 3: 0.00298, 4: 0.00189,	1: 0.00215, 2: 0.00235, 3: 0.00188, 4: 0.00145,	AZOBR_10494	1: 0.00042, 2: 0.00024, 3: 0.00055, 4: 0.00066, 5: 0.00029,	2: 0.00008, 3: 0.00021,
AZOLI_0622	4: 0.00013,	1: 0.00071, 2: 0.00054, 3: 0.00066, 4: 0.00050,	AZOBR_10496		
AZOLI_0624	2: 0.00005, 4: 0.00003,	1: 0.00012, 3: 0.00011, 4: 0.00006,	AZOBR_10497	1: 0.00018, 3: 0.00025, 4: 0.00012, 5: 0.00012,	1: 0.00008, 3: 0.00018,
AZOLI_0626	1: 0.00067, 2: 0.00056, 3: 0.00067, 4: 0.00048,	1: 0.00133, 2: 0.00124, 3: 0.00094, 4: 0.00137,	AZOBR_10498		1: 0.00022, 3: 0.00033,
AZOLI_0629	1: 0.00067, 4: 0.00053,	1: 0.00026, 2: 0.00050, 3: 0.00055, 4: 0.00018,	AZOBR_10499		
AZOLI_0634	1: 0.00013, 2: 0.00009, 4: 0.00005,	1: 0.00023, 2: 0.00025, 3: 0.00034, 4: 0.00018,	AZOBR_10500	1: 0.00021, 2: 0.00070, 3: 0.00036, 4: 0.00043, 5: 0.00061,	1: 0.00021, 2: 0.00015, 3: 0.00023,
AZOLI_0635	2: 0.00020, 4: 0.00017,	1: 0.00031, 2: 0.00035, 3: 0.00027, 4: 0.00026,	AZOBR_10501		
AZOLI_0638		1: 0.00015, 3: 0.00016, 4: 0.00026,	AZOBR_10502	4: 0.00005,	
AZOLI_0641	1: 0.00085, 2: 0.00136, 3: 0.00070, 4: 0.00110,	1: 0.00130, 2: 0.00056, 3: 0.00084, 4: 0.00068,	AZOBR_10503	1: 0.00065, 2: 0.00092, 3: 0.00099, 4: 0.00116, 5: 0.00063,	1: 0.00864, 2: 0.01076, 3: 0.00765,
AZOLI_0642	1: 0.00141, 2: 0.00106, 3: 0.00538, 4: 0.00185,	1: 0.00202, 2: 0.00143, 3: 0.00230, 4: 0.00199,	AZOBR_10504		
AZOLI_0647		2: 0.00016,	AZOBR_10505	1: 0.00003,	2: 0.00003, 3: 0.00003,
AZOLI_0653	1: 0.00033, 2: 0.00034, 3: 0.00035, 4: 0.00032,	1: 0.00025, 2: 0.00037, 3: 0.00018, 4: 0.00018,	AZOBR_10506		3: 0.00008,
AZOLI_0654	1: 0.00085, 2: 0.00038, 3: 0.00053, 4: 0.00163,	1: 0.00134, 2: 0.00147, 3: 0.00121, 4: 0.00130,	AZOBR_10509	1: 0.00018, 4: 0.00020,	
AZOLI_0655		2: 0.00005,	AZOBR_10510	1: 0.00023, 2: 0.00005, 3: 0.00022, 4: 0.00014, 5: 0.00014,	1: 0.00030, 2: 0.00032, 3: 0.00036,
AZOLI_0657		4: 0.00007,	AZOBR_10514	1: 0.00026, 2: 0.00070, 3: 0.00097, 4: 0.00042, 5: 0.00036,	1: 0.00034, 2: 0.00025, 3: 0.00011,
AZOLI_0658		4: 0.00009,	AZOBR_10520		
AZOLI_0659		4: 0.00006,	AZOBR_10523	1: 0.00025, 2: 0.00036, 3: 0.00035, 4: 0.00040, 5: 0.00028,	1: 0.00189, 2: 0.00275, 3: 0.00133,
AZOLI_0660	1: 0.00033, 2: 0.00076, 3: 0.00035, 4: 0.00050,	1: 0.00042, 2: 0.00032, 3: 0.00027, 4: 0.00014,	AZOBR_10524		1: 0.00027, 2: 0.00008, 3: 0.00023,
AZOLI_0661	4: 0.00019,	4: 0.00010,	AZOBR_10525	5: 0.00005,	1: 0.00018, 2: 0.00020, 3: 0.00019,
AZOLI_0665		2: 0.00043, 3: 0.00038, 4: 0.00015,	AZOBR_10527		
AZOLI_0666	1: 0.00009, 4: 0.00015,		AZOBR_10528	1: 0.00691, 2: 0.00302, 3: 0.00529, 4: 0.00299, 5: 0.00252,	1: 0.00070, 2: 0.00102, 3: 0.00111,
AZOLI_0667	4: 0.00006,		AZOBR_10531	1: 0.00010, 2: 0.00006, 3: 0.00006, 4: 0.00012, 5: 0.00009,	1: 0.00009, 2: 0.00007, 3: 0.00006,
AZOLI_0669		2: 0.00034, 3: 0.00050, 4: 0.00060,	AZOBR_10534	4: 0.00019, 5: 0.00023,	
AZOLI_0670		1: 0.00139, 3: 0.00092, 4: 0.00133,	AZOBR_10535		
AZOLI_0671		1: 0.00053, 2: 0.00117, 3: 0.00137, 4: 0.00246,	AZOBR_10536		2: 0.00013, 3: 0.00008,
AZOLI_0672		1: 0.00191, 2: 0.00293, 3: 0.00120, 4: 0.00067,	AZOBR_10537		
AZOLI_0673		1: 0.00007, 2: 0.00007, 4: 0.00006,	AZOBR_10538		
AZOLI_0677	4: 0.00003,		AZOBR_20005		
AZOLI_0679		1: 0.00243, 2: 0.00213, 3: 0.00243, 4: 0.00228,	AZOBR_20009		
AZOLI_0680		1: 0.00125, 2: 0.00134, 3: 0.00127, 4: 0.00122,	AZOBR_20011		
AZOLI_0681		1: 0.00720, 2: 0.00715, 3: 0.00619, 4: 0.00587,	AZOBR_20012		1: 0.00103, 2: 0.00045, 3: 0.00055,
AZOLI_0685		4: 0.00009,	AZOBR_20015	1: 0.00968, 2: 0.00935, 3: 0.00644, 4: 0.01333, 5: 0.01272,	1: 0.01133, 2: 0.00683, 3: 0.00771,
AZOLI_0686		1: 0.00024, 2: 0.00031, 3: 0.00051,	AZOBR_20016	1: 0.01441, 2: 0.01416, 3: 0.01184, 4: 0.01314, 5: 0.01523,	1: 0.00955, 2: 0.00970, 3: 0.00910,
AZOLI_0690	1: 0.00014, 2: 0.00022, 3: 0.00031, 4: 0.00021,	1: 0.00022, 2: 0.00005, 3: 0.00015, 4: 0.00017,	AZOBR_20017		
AZOLI_0694	2: 0.00012, 4: 0.00031,	2: 0.00008, 3: 0.00013, 4: 0.00014,	AZOBR_20019	1: 0.00091, 2: 0.00073, 3: 0.00056, 4: 0.00090, 5: 0.00066,	1: 0.00484, 2: 0.00535, 3: 0.00370,
AZOLI_0698	1: 0.00228, 2: 0.00247, 3: 0.00204, 4: 0.00216,	1: 0.00040, 2: 0.00093, 3: 0.00084, 4: 0.00067,	AZOBR_20020		
AZOLI_0699	1: 0.00058, 2: 0.00068, 3: 0.00039, 4: 0.00066,	1: 0.00080, 2: 0.00048, 3: 0.00062, 4: 0.00068,	AZOBR_20022		
AZOLI_0704	4: 0.00017,	1: 0.00016, 2: 0.00023, 3: 0.00050, 4: 0.00017,	AZOBR_20027	2: 0.00029, 3: 0.00028, 4: 0.00040, 5: 0.00058,	3: 0.00036,
AZOLI_0707	1: 0.00015, 2: 0.00018,	1: 0.00015, 2: 0.00020, 3: 0.00006,	AZOBR_20032	3: 0.00016, 4: 0.00019, 5: 0.00019,	2: 0.00027, 3: 0.00029,
AZOLI_0715		3: 0.00007,	AZOBR_30003		1: 0.00014, 3: 0.00027,
AZOLI_0730	4: 0.00003,	4: 0.00005,	AZOBR_30005	4: 0.00027, 5: 0.00027,	3: 0.00087,
AZOLI_0732		2: 0.00006, 3: 0.00006,	AZOBR_30007	1: 0.00041, 2: 0.00036, 3: 0.00039,	1: 0.00035, 2: 0.00051,

		4: 0.00010,		4: 0.00049, 5: 0.00041,	3: 0.00059,
AZOLI_0733		1: 0.00013, 2: 0.00034, 3: 0.00023, 4: 0.00034,	AZOBR_30012		1: 0.00010,
AZOLI_0734	3: 0.00009,		AZOBR_30018	1: 0.00017, 2: 0.00030, 3: 0.00048, 4: 0.00035, 5: 0.00035,	1: 0.00022, 2: 0.00012, 3: 0.00024,
AZOLI_0735		4: 0.00006,	AZOBR_30019	1: 0.00019, 3: 0.00014, 4: 0.00015, 5: 0.00004,	1: 0.00005, 3: 0.00005,
AZOLI_0739	1: 0.00005, 4: 0.00004,	2: 0.00005, 4: 0.00010,	AZOBR_30020	1: 0.00025, 2: 0.00017, 3: 0.00043, 4: 0.00018, 5: 0.00013,	1: 0.00016, 2: 0.00037, 3: 0.00032,
AZOLI_0746	1: 0.00094, 2: 0.00097, 3: 0.00076, 4: 0.00119,	1: 0.00119, 2: 0.00099, 3: 0.00101, 4: 0.00081,	AZOBR_30021	1: 0.00027, 3: 0.00060, 4: 0.00024, 5: 0.00012,	1: 0.00011, 2: 0.00019, 3: 0.00017,
AZOLI_0747		3: 0.00015, 4: 0.00024,	AZOBR_30029	1: 0.00063, 2: 0.00067, 3: 0.00064, 4: 0.00078, 5: 0.00055,	1: 0.00097, 2: 0.00074, 3: 0.00081,
AZOLI_0748		3: 0.00005,	AZOBR_30034	1: 0.00095, 2: 0.00084, 3: 0.00099, 4: 0.00124, 5: 0.00117,	3: 0.00059,
AZOLI_0753	2: 0.00013, 4: 0.00007,		AZOBR_40002		1: 0.00002, 2: 0.00006, 3: 0.00011,
AZOLI_0755		2: 0.00007,	AZOBR_40038	1: 0.00039, 2: 0.00068, 3: 0.00051, 4: 0.00063, 5: 0.00034,	1: 0.00014, 2: 0.00015, 3: 0.00031,
AZOLI_0758	2: 0.00003, 3: 0.00009, 4: 0.00008,	1: 0.00020, 2: 0.00012, 3: 0.00023, 4: 0.00016,	AZOBR_40039	1: 0.00161, 2: 0.00329, 3: 0.00149, 4: 0.00275, 5: 0.00298,	1: 0.00268, 2: 0.00164, 3: 0.00260,
AZOLI_0763	2: 0.00013, 4: 0.00006,	1: 0.00017, 3: 0.00014, 4: 0.00011,	AZOBR_40040	1: 0.00014, 2: 0.00013, 3: 0.00015, 4: 0.00015, 5: 0.00014,	1: 0.00014, 2: 0.00013, 3: 0.00017,
AZOLI_0764		4: 0.00005,	AZOBR_40042	1: 0.00040, 2: 0.00045, 3: 0.00053, 4: 0.00045, 5: 0.00045,	
AZOLI_0768	4: 0.00014,	1: 0.00024, 3: 0.00043, 4: 0.00028,	AZOBR_40045	2: 0.00041, 3: 0.00020, 4: 0.00043, 5: 0.00048,	1: 0.00050, 2: 0.00045, 3: 0.00068,
AZOLI_0769	1: 0.00005, 2: 0.00013, 3: 0.00087, 4: 0.00013,	1: 0.00020, 2: 0.00020, 3: 0.00020, 4: 0.00028,	AZOBR_40049	4: 0.00007, 5: 0.00013,	
AZOLI_0770	1: 0.00026, 2: 0.00017, 3: 0.00132, 4: 0.00015,	1: 0.00013, 3: 0.00023, 4: 0.00007,	AZOBR_40052	2: 0.00059, 3: 0.00028, 4: 0.00034, 5: 0.00023,	2: 0.00024, 3: 0.00021,
AZOLI_0771	1: 0.00014, 2: 0.00015, 4: 0.00008,	2: 0.00009, 4: 0.00008,	AZOBR_40053		
AZOLI_0773	1: 0.00017, 2: 0.00023, 4: 0.00054,	1: 0.00047, 3: 0.00018, 4: 0.00020,	AZOBR_40054	3: 0.00024,	
AZOLI_0775	1: 0.00004, 2: 0.00004, 3: 0.00025, 4: 0.00005,	1: 0.00004, 2: 0.00006, 4: 0.00003,	AZOBR_40057		
AZOLI_0776	1: 0.00129, 2: 0.00103, 3: 0.00096, 4: 0.00100,	1: 0.00076, 2: 0.00060, 3: 0.00066, 4: 0.00029,	AZOBR_40058		1: 0.00005, 3: 0.00003,
AZOLI_0783		2: 0.00011,	AZOBR_40059		
AZOLI_0785	1: 0.01447, 2: 0.01128, 3: 0.01017, 4: 0.01102,	1: 0.01026, 2: 0.00803, 3: 0.00875, 4: 0.00788,	AZOBR_40062		
AZOLI_0788	1: 0.00005, 2: 0.00008, 3: 0.00005, 4: 0.00003,	1: 0.00007, 2: 0.00005, 3: 0.00009, 4: 0.00007,	AZOBR_40064		
AZOLI_0789	1: 0.00011,	4: 0.00003,	AZOBR_40067		2: 0.00002, 3: 0.00005,
AZOLI_0794	4: 0.00007,	3: 0.00007, 4: 0.00007,	AZOBR_40075	2: 0.00056, 3: 0.00062, 4: 0.00031, 5: 0.00031,	2: 0.00053, 3: 0.00040,
AZOLI_0796	1: 0.00014, 2: 0.00010, 3: 0.00007, 4: 0.00010,	1: 0.00015, 2: 0.00009, 3: 0.00010, 4: 0.00017,	AZOBR_40081		
AZOLI_0797	4: 0.00012,	1: 0.00005, 3: 0.00006, 4: 0.00012,	AZOBR_40082		2: 0.00004,
AZOLI_0802	1: 0.00060, 2: 0.00060, 3: 0.00072, 4: 0.00048,	1: 0.00048, 2: 0.00058, 3: 0.00023, 4: 0.00030,	AZOBR_40083		1: 0.00006, 3: 0.00006,
AZOLI_0803	1: 0.00289, 2: 0.00261, 3: 0.00161, 4: 0.00109,	1: 0.00159, 2: 0.00129, 3: 0.00123, 4: 0.00104,	AZOBR_40084		
AZOLI_0809	2: 0.00010, 4: 0.00009,		AZOBR_40085		
AZOLI_0810	1: 0.00227, 2: 0.00296, 3: 0.00366, 4: 0.00278,	1: 0.00312, 2: 0.00268, 3: 0.00275, 4: 0.00247,	AZOBR_40086		1: 0.00015, 2: 0.00011, 3: 0.00015,
AZOLI_0814	4: 0.00004,		AZOBR_40087	3: 0.00040, 4: 0.00043,	3: 0.00029,
AZOLI_0815	1: 0.00496, 2: 0.00470, 3: 0.00823, 4: 0.00454,	1: 0.00603, 2: 0.00643, 3: 0.00652, 4: 0.00610,	AZOBR_40088	3: 0.00037, 4: 0.00083, 5: 0.00068,	1: 0.00056, 2: 0.00056, 3: 0.00027,
AZOLI_0816	1: 0.00034, 2: 0.00036, 3: 0.00019, 4: 0.00023,	1: 0.00041, 2: 0.00055, 3: 0.00031, 4: 0.00035,	AZOBR_40089		1: 0.00003, 2: 0.00016, 3: 0.00009,
AZOLI_0820	2: 0.00046, 4: 0.00053,	1: 0.00047, 2: 0.00119, 3: 0.00098, 4: 0.00053,	AZOBR_40090	1: 0.00055, 2: 0.00029, 3: 0.00056, 4: 0.00036, 5: 0.00020,	1: 0.00015, 2: 0.00021, 3: 0.00020,
AZOLI_0821	1: 0.00033, 4: 0.00048,	2: 0.00032,	AZOBR_40091	1: 0.00013, 3: 0.00022, 4: 0.00009, 5: 0.00032,	2: 0.00029, 3: 0.00016,
AZOLI_0822	2: 0.00012,	1: 0.00024, 4: 0.00020,	AZOBR_40092	1: 0.00429, 2: 0.00518, 3: 0.00480, 4: 0.00408, 5: 0.00491,	1: 0.00389, 2: 0.00437, 3: 0.00392,
AZOLI_0823	2: 0.00013,		AZOBR_40094	4: 0.00053, 5: 0.00021,	1: 0.00129, 2: 0.00124, 3: 0.00134,
AZOLI_0824	1: 0.00032, 2: 0.00014, 3: 0.00038, 4: 0.00051,	1: 0.00041, 2: 0.00062, 3: 0.00058, 4: 0.00055,	AZOBR_40095		1: 0.00043, 3: 0.00042,
AZOLI_0826	1: 0.00027, 2: 0.00011, 4: 0.00019,	1: 0.00017, 2: 0.00011, 3: 0.00023, 4: 0.00009,	AZOBR_40096		
AZOLI_0827	1: 0.00016, 2: 0.00010, 4: 0.00008,		AZOBR_40097		
AZOLI_0832	1: 0.00068, 2: 0.00089, 3: 0.00053, 4: 0.00061,	1: 0.00069, 2: 0.00071, 3: 0.00059, 4: 0.00046,	AZOBR_40099		
AZOLI_0833	2: 0.00052, 3: 0.00083, 4: 0.00051,	1: 0.00022, 4: 0.00013,	AZOBR_40100	1: 0.00110, 2: 0.00081, 3: 0.00073, 4: 0.00101, 5: 0.00068,	1: 0.00055, 2: 0.00049, 3: 0.00046,
AZOLI_0835	1: 0.00051, 2: 0.00072, 4: 0.00065,	1: 0.00072, 2: 0.00054, 3: 0.00063, 4: 0.00048,	AZOBR_40101	1: 0.00047, 2: 0.00042, 3: 0.00035, 4: 0.00028, 5: 0.00023,	1: 0.00017, 2: 0.00010, 3: 0.00017,
AZOLI_0837	1: 0.00014, 2: 0.00012, 3: 0.00035, 4: 0.00014,	1: 0.00004, 2: 0.00009, 4: 0.00009,	AZOBR_40103	1: 0.00060, 2: 0.00137, 3: 0.00063, 4: 0.00079, 5: 0.00056,	1: 0.00082, 2: 0.00078, 3: 0.00104,
AZOLI_0839	1: 0.00008,	1: 0.00016, 2: 0.00027, 3: 0.00009,	AZOBR_40106		
AZOLI_0842	1: 0.00010, 2: 0.00028, 3: 0.00039, 4: 0.00018,	1: 0.00028, 2: 0.00017, 3: 0.00019, 4: 0.00033,	AZOBR_40107	1: 0.00204, 2: 0.00326, 3: 0.00257, 4: 0.00304, 5: 0.00281,	1: 0.00362, 2: 0.00325, 3: 0.00382,
AZOLI_0847	2: 0.00005, 3: 0.00007,		AZOBR_40108	3: 0.00022, 4: 0.00024, 5: 0.00012,	1: 0.00049, 2: 0.00050,

	4: 0.00021,				3: 0.00069,
AZOLI_0848		4: 0.00007,	AZOBR_40110		
AZOLI_0851		2: 0.00009,	AZOBR_40113	5: 0.00006,	2: 0.00006,
AZOLI_0852	3: 0.00041, 4: 0.00014,	1: 0.00055, 2: 0.00040, 3: 0.00049, 4: 0.00050,	AZOBR_40116	1: 0.00032, 2: 0.00028, 3: 0.00027, 4: 0.00056, 5: 0.00044,	1: 0.00083, 2: 0.00059, 3: 0.00060,
AZOLI_0854	4: 0.00014,		AZOBR_40117	1: 0.00018,	1: 0.00034, 3: 0.00022,
AZOLI_0859	1: 0.00285, 2: 0.00287, 3: 0.00206, 4: 0.00305,	1: 0.00297, 2: 0.00203, 3: 0.00223, 4: 0.00226,	AZOBR_40118	1: 0.00014, 3: 0.00024, 4: 0.00015,	3: 0.00009,
AZOLI_0861	2: 0.00031, 3: 0.00066, 4: 0.00054,	1: 0.00040, 2: 0.00038, 3: 0.00042, 4: 0.00027,	AZOBR_40120	1: 0.00012, 2: 0.00014, 3: 0.00016, 4: 0.00016, 5: 0.00008,	1: 0.00012, 2: 0.00006, 3: 0.00012,
AZOLI_0862	1: 0.00073, 2: 0.00076, 3: 0.00144, 4: 0.00088,	1: 0.00077, 2: 0.00071, 3: 0.00070, 4: 0.00104,	AZOBR_40127	4: 0.00004,	1: 0.00003, 2: 0.00006, 3: 0.00012,
AZOLI_0867	2: 0.00014,		AZOBR_40128		
AZOLI_0868	1: 0.00081, 2: 0.00099, 3: 0.00033, 4: 0.00100,	1: 0.00129, 2: 0.00147, 3: 0.00124, 4: 0.00085,	AZOBR_40130	1: 0.00032, 2: 0.00060, 3: 0.00062, 4: 0.00063, 5: 0.00072,	1: 0.00050, 2: 0.00057, 3: 0.00062,
AZOLI_0870	1: 0.00033, 2: 0.00027, 4: 0.00026,	1: 0.00058, 2: 0.00035, 3: 0.00067, 4: 0.00041,	AZOBR_40135	4: 0.00019, 5: 0.00028,	
AZOLI_0871	1: 0.00064, 2: 0.00088, 3: 0.00057, 4: 0.00080,	1: 0.00094, 2: 0.00102, 3: 0.00093, 4: 0.00055,	AZOBR_40136	1: 0.00141, 2: 0.00066, 3: 0.00068, 4: 0.00097, 5: 0.00052,	1: 0.00042, 2: 0.00051, 3: 0.00064,
AZOLI_0873	4: 0.00012,		AZOBR_40139	1: 0.00013, 2: 0.00041, 3: 0.00011, 4: 0.00041, 5: 0.00036,	1: 0.00013, 2: 0.00019, 3: 0.00041,
AZOLI_0875	4: 0.00015,	3: 0.00025,	AZOBR_40140	1: 0.00015, 3: 0.00006, 4: 0.00009, 5: 0.00005,	1: 0.00005, 2: 0.00009, 3: 0.00005,
AZOLI_0876	3: 0.00180,	1: 0.00167, 2: 0.00143,	AZOBR_40142	1: 0.00073, 2: 0.00024, 3: 0.00023, 4: 0.00025, 5: 0.00031,	1: 0.00023, 2: 0.00040,
AZOLI_0877	4: 0.00006,	4: 0.00006,	AZOBR_40143		
AZOLI_0878	2: 0.00014, 4: 0.00009,	2: 0.00007,	AZOBR_40155		
AZOLI_0880	1: 0.00103, 2: 0.00145, 3: 0.00116, 4: 0.00089,	1: 0.00042, 2: 0.00050, 3: 0.00033, 4: 0.00027,	AZOBR_40156		
AZOLI_0918	1: 0.00024, 2: 0.00041, 4: 0.00042,		AZOBR_40157		
AZOLI_0922	1: 0.00007, 2: 0.00015, 4: 0.00006,	1: 0.00026, 2: 0.00025, 3: 0.00012, 4: 0.00009,	AZOBR_40166	2: 0.00020, 3: 0.00025, 4: 0.00021, 5: 0.00010,	1: 0.00067, 2: 0.00054, 3: 0.00047,
AZOLI_0923	2: 0.00051, 4: 0.00060,	1: 0.00051, 2: 0.00020, 3: 0.00043, 4: 0.00052,	AZOBR_40167		
AZOLI_0924	1: 0.00030, 2: 0.00083, 3: 0.00038, 4: 0.00099,	1: 0.00030, 2: 0.00037, 3: 0.00035, 4: 0.00028,	AZOBR_40170	1: 0.00023, 2: 0.00007, 3: 0.00010, 4: 0.00040, 5: 0.00035,	1: 0.00015, 2: 0.00011, 3: 0.00041,
AZOLI_0928	1: 0.00099, 2: 0.00117, 3: 0.00123, 4: 0.00073,	1: 0.00112, 2: 0.00099, 3: 0.00146, 4: 0.00113,	AZOBR_40175	1: 0.00013, 2: 0.00015, 3: 0.00011, 4: 0.00018, 5: 0.00018,	1: 0.00016, 2: 0.00009, 3: 0.00032,
AZOLI_0929	1: 0.00051, 2: 0.00059, 3: 0.00166, 4: 0.00158,	1: 0.00060, 2: 0.00065, 3: 0.00055, 4: 0.00089,	AZOBR_40178	1: 0.00015, 2: 0.00006, 3: 0.00016, 4: 0.00020, 5: 0.00005,	1: 0.00009, 2: 0.00011, 3: 0.00011,
AZOLI_0931	1: 0.00016, 2: 0.00013,	1: 0.00004,	AZOBR_40179	1: 0.00026, 2: 0.00009, 3: 0.00013, 4: 0.00021, 5: 0.00014,	1: 0.00007, 2: 0.00015, 3: 0.00019,
AZOLI_0932	1: 0.00034, 2: 0.00032, 3: 0.00041, 4: 0.00055,	1: 0.00045, 2: 0.00033, 3: 0.00056, 4: 0.00053,	AZOBR_40180	1: 0.00033, 2: 0.00011, 3: 0.00028, 4: 0.00022, 5: 0.00027,	2: 0.00014,
AZOLI_0938	3: 0.00016,		AZOBR_40181		1: 0.00003, 2: 0.00004,
AZOLI_0952	4: 0.00035,		AZOBR_40182		
AZOLI_0958		1: 0.00025,	AZOBR_40183	1: 0.00014, 2: 0.00012, 3: 0.00012, 4: 0.00028, 5: 0.00009,	1: 0.00031, 2: 0.00025, 3: 0.00013,
AZOLI_0961	1: 0.00013, 2: 0.00021, 4: 0.00013,		AZOBR_40185	2: 0.00009, 3: 0.00011, 4: 0.00007, 5: 0.00007,	1: 0.00006, 2: 0.00012, 3: 0.00010,
AZOLI_0964	1: 0.00202, 2: 0.00209, 3: 0.00067, 4: 0.00215,	1: 0.00092, 2: 0.00078, 3: 0.00057, 4: 0.00060,	AZOBR_40187	1: 0.00038, 2: 0.00034, 3: 0.00033, 4: 0.00079, 5: 0.00055,	1: 0.00022, 2: 0.00014, 3: 0.00022,
AZOLI_0966	1: 0.00005, 4: 0.00006,		AZOBR_40188	1: 0.01042, 2: 0.00690, 3: 0.00919, 4: 0.00444, 5: 0.00428,	1: 0.01121, 2: 0.01003, 3: 0.00447,
AZOLI_0967		4: 0.00007,	AZOBR_40189		2: 0.00011, 3: 0.00009,
AZOLI_0969	1: 0.00023, 2: 0.00046, 3: 0.00127, 4: 0.00052,	1: 0.00038, 2: 0.00029, 3: 0.00032, 4: 0.00039,	AZOBR_40190	1: 0.00011, 2: 0.00010, 3: 0.00009, 4: 0.00019, 5: 0.00015,	1: 0.00017, 2: 0.00012, 3: 0.00017,
AZOLI_0972		2: 0.00004, 3: 0.00004, 4: 0.00005,	AZOBR_40191	1: 0.00085, 2: 0.00084, 3: 0.00144, 4: 0.00226, 5: 0.00241,	1: 0.00278, 2: 0.00062, 3: 0.00290,
AZOLI_0975		1: 0.00016, 3: 0.00025, 4: 0.00034,	AZOBR_40194	1: 0.00137, 2: 0.00154, 3: 0.00116, 4: 0.00113, 5: 0.00109,	1: 0.00139, 2: 0.00113, 3: 0.00106,
AZOLI_0980		4: 0.00014,	AZOBR_40199	5: 0.00012,	
AZOLI_0983	1: 0.00026, 2: 0.00020, 3: 0.00006, 4: 0.00034,	1: 0.00007, 2: 0.00011, 3: 0.00014, 4: 0.00011,	AZOBR_40200	1: 0.00041, 2: 0.00012, 3: 0.00019, 4: 0.00034, 5: 0.00016,	1: 0.00006, 2: 0.00013, 3: 0.00006,
AZOLI_0984	1: 0.00020, 2: 0.00010, 4: 0.00026,	2: 0.00010, 3: 0.00021, 4: 0.00026,	AZOBR_40201	1: 0.00052, 2: 0.00076, 3: 0.00037, 4: 0.00065, 5: 0.00042,	1: 0.00033, 3: 0.00032,
AZOLI_0990	1: 0.00574, 2: 0.00546, 3: 0.00751, 4: 0.00418,	1: 0.00377, 2: 0.00418, 3: 0.00286, 4: 0.00377,	AZOBR_40202	1: 0.00363, 2: 0.00371, 3: 0.00455, 4: 0.00355, 5: 0.00329,	1: 0.00171, 2: 0.00195, 3: 0.00226,
AZOLI_0991	1: 0.00020, 4: 0.00035,	1: 0.00020, 3: 0.00029, 4: 0.00035,	AZOBR_40203	1: 0.00631, 2: 0.00632, 3: 0.00734, 4: 0.00739, 5: 0.01117,	1: 0.00485, 2: 0.00444, 3: 0.00418,
AZOLI_0997	1: 0.00015,	4: 0.00013,	AZOBR_40205		
AZOLI_1001		2: 0.00029,	AZOBR_40207		1: 0.00021, 2: 0.00014, 3: 0.00035,
AZOLI_1007		1: 0.00013, 4: 0.00007,	AZOBR_40208	1: 0.00012, 2: 0.00016, 3: 0.00020, 4: 0.00020, 5: 0.00012,	1: 0.00008,
AZOLI_1009	1: 0.00009, 2: 0.00005, 4: 0.00006,	1: 0.00016, 2: 0.00013, 3: 0.00020, 4: 0.00010,	AZOBR_40209	3: 0.00014, 4: 0.00008, 5: 0.00008,	1: 0.00018, 2: 0.00016, 3: 0.00031,
AZOLI_1010	1: 0.00034, 2: 0.00054, 3: 0.00256, 4: 0.00134,	1: 0.00137, 2: 0.00092, 3: 0.00101, 4: 0.00157,	AZOBR_40210	4: 0.00011, 5: 0.00017,	
AZOLI_1011	1: 0.00024, 2: 0.00021, 3: 0.00013, 4: 0.00028,	1: 0.00015, 2: 0.00017, 3: 0.00019, 4: 0.00021,	AZOBR_40211	1: 0.00029, 2: 0.00013, 3: 0.00015, 4: 0.00030, 5: 0.00030,	1: 0.00005, 2: 0.00010, 3: 0.00004,
AZOLI_1015	1: 0.00006, 3: 0.00007, 4: 0.00004,	1: 0.00013, 2: 0.00006, 3: 0.00018, 4: 0.00010,	AZOBR_40214	3: 0.00008, 4: 0.00008, 5: 0.00006,	
AZOLI_1016	4: 0.00025,	1: 0.00022, 2: 0.00021, 4: 0.00043,	AZOBR_40216	1: 0.00005,	2: 0.00004, 3: 0.00005,
AZOLI_1018	4: 0.00004,	1: 0.00003,	AZOBR_40218	1: 0.00047, 2: 0.00041, 3: 0.00062, 4: 0.00079, 5: 0.00079,	1: 0.00157, 2: 0.00174, 3: 0.00152,
AZOLI_1020	4: 0.00005,	1: 0.00005, 2: 0.00011, 4: 0.00005,	AZOBR_40223		2: 0.00018, 3: 0.00009,

AZOLI_1024	2: 0.00010, 4: 0.00011,	2: 0.00006,	AZOBR_40227		
AZOLI_1029		1: 0.00020, 2: 0.00020,	AZOBR_40228		2: 0.00008, 3: 0.00003,
AZOLI_1030	1: 0.00059, 2: 0.00020, 4: 0.00034,	2: 0.00029, 3: 0.00063,	AZOBR_40232		
AZOLI_1033	4: 0.00007,		AZOBR_40233	1: 0.00021, 3: 0.00042, 4: 0.00024, 5: 0.00010,	1: 0.00027, 2: 0.00015, 3: 0.00026,
AZOLI_1036	2: 0.00005, 3: 0.00006, 4: 0.00019,	1: 0.00007, 2: 0.00007, 3: 0.00012, 4: 0.00008,	AZOBR_40234		
AZOLI_1037	1: 0.00011, 2: 0.00027, 4: 0.00033,	1: 0.00004, 2: 0.00005, 3: 0.00012, 4: 0.00008,	AZOBR_40238	1: 0.00028, 2: 0.00025, 3: 0.00035, 4: 0.00020, 5: 0.00025,	1: 0.00008, 3: 0.00015,
AZOLI_1047	1: 0.00083, 2: 0.00118, 4: 0.00126,	1: 0.00061, 2: 0.00071, 3: 0.00054, 4: 0.00055,	AZOBR_40241	1: 0.00036, 2: 0.00043, 3: 0.00046, 4: 0.00037, 5: 0.00037,	1: 0.00027, 2: 0.00018, 3: 0.00015,
AZOLI_1055	1: 0.00033, 2: 0.00039, 3: 0.00029, 4: 0.00039,	1: 0.00043, 2: 0.00031, 3: 0.00034, 4: 0.00037,	AZOBR_40244		1: 0.00002,
AZOLI_1061	4: 0.00005,	4: 0.00005,	AZOBR_40250		
AZOLI_1062	3: 0.00026, 4: 0.00008,	1: 0.00038, 2: 0.00023, 3: 0.00015, 4: 0.00024,	AZOBR_40252		3: 0.00004,
AZOLI_1063	1: 0.00074, 2: 0.00112, 3: 0.00067, 4: 0.00059,	1: 0.00059, 2: 0.00051, 3: 0.00062, 4: 0.00045,	AZOBR_40253	1: 0.00009, 4: 0.00006,	1: 0.00008, 2: 0.00012, 3: 0.00019,
AZOLI_1064	1: 0.00016, 2: 0.00008, 3: 0.00017, 4: 0.00010,	1: 0.00024, 2: 0.00008, 3: 0.00038, 4: 0.00010,	AZOBR_40257		1: 0.00003,
AZOLI_1068	1: 0.00023, 2: 0.00032, 3: 0.00028, 4: 0.00025,	1: 0.00017, 2: 0.00039, 3: 0.00025, 4: 0.00027,	AZOBR_40259	1: 0.00036,	
AZOLI_1069	1: 0.00033, 2: 0.00034, 3: 0.00016, 4: 0.00053,	1: 0.00040, 2: 0.00042, 3: 0.00054, 4: 0.00040,	AZOBR_40262		1: 0.00003, 2: 0.00005,
AZOLI_1070	1: 0.00283, 2: 0.00220, 3: 0.00386, 4: 0.00285,	1: 0.00137, 2: 0.00174, 3: 0.00177, 4: 0.00188,	AZOBR_40263		
AZOLI_1071	1: 0.00795, 2: 0.00715, 3: 0.00802, 4: 0.00670,	1: 0.00474, 2: 0.00567, 3: 0.00662, 4: 0.00583,	AZOBR_40268	2: 0.00015, 4: 0.00012,	3: 0.00017,
AZOLI_1072	3: 0.00070, 4: 0.00016,	1: 0.00046, 2: 0.00048, 3: 0.00087, 4: 0.00055,	AZOBR_40269		
AZOLI_1073	1: 0.00131, 2: 0.00119, 3: 0.00221, 4: 0.00214,	1: 0.00119, 2: 0.00109, 3: 0.00168, 4: 0.00249,	AZOBR_40270		3: 0.00021,
AZOLI_1078	3: 0.00020,		AZOBR_40271	1: 0.00017,	3: 0.00007,
AZOLI_1079	1: 0.00148, 2: 0.00229, 3: 0.00089, 4: 0.00260,	1: 0.00209, 2: 0.00176, 3: 0.00165, 4: 0.00169,	AZOBR_40272		1: 0.00021,
AZOLI_1080		1: 0.00014, 4: 0.00018,	AZOBR_40273		
AZOLI_1081	1: 0.00017, 2: 0.00048, 3: 0.00066, 4: 0.00044,	1: 0.00048, 2: 0.00071, 3: 0.00041, 4: 0.00030,	AZOBR_40274	1: 0.00475, 2: 0.00395, 3: 0.00381, 4: 0.00308, 5: 0.00402,	1: 0.00243, 2: 0.00326, 3: 0.00118,
AZOLI_1088	1: 0.00075, 2: 0.00112, 3: 0.00015, 4: 0.00062,	1: 0.00074, 2: 0.00091, 3: 0.00081, 4: 0.00060,	AZOBR_40275	1: 0.00015, 3: 0.00013, 5: 0.00010,	
AZOLI_1089	1: 0.00034, 2: 0.00034, 3: 0.00071, 4: 0.00054,	1: 0.00098, 2: 0.00064, 3: 0.00070, 4: 0.00077,	AZOBR_40277	1: 0.00226, 2: 0.00261, 3: 0.00199, 4: 0.00141, 5: 0.00219,	1: 0.00168, 2: 0.00218, 3: 0.00154,
AZOLI_1091	1: 0.00028, 2: 0.00048, 4: 0.00033,	1: 0.00014,	AZOBR_40279		
AZOLI_1092		4: 0.00009,	AZOBR_40280	1: 0.00014, 2: 0.00012, 4: 0.00014,	1: 0.00009, 2: 0.00015, 3: 0.00026,
AZOLI_1093	1: 0.00014,		AZOBR_40283		
AZOLI_1094	1: 0.00022, 2: 0.00044, 4: 0.00012,	1: 0.00022, 3: 0.00031,	AZOBR_40286		
AZOLI_1095	1: 0.00025, 2: 0.00030, 3: 0.00018, 4: 0.00026,	1: 0.00030, 2: 0.00008, 3: 0.00032, 4: 0.00018,	AZOBR_40288		
AZOLI_1096	1: 0.00016, 3: 0.00014, 4: 0.00006,	1: 0.00013, 2: 0.00019, 3: 0.00007, 4: 0.00009,	AZOBR_40290		
AZOLI_1097	1: 0.00101, 2: 0.00057, 3: 0.00032, 4: 0.00072,	1: 0.00011, 2: 0.00011, 3: 0.00018, 4: 0.00010,	AZOBR_40292	1: 0.00040, 2: 0.00061, 3: 0.00088, 4: 0.00075, 5: 0.00077,	1: 0.00030, 2: 0.00032, 3: 0.00052,
AZOLI_1102		4: 0.00003,	AZOBR_40299	1: 0.00186, 2: 0.00141, 3: 0.00136, 4: 0.00083, 5: 0.00115,	1: 0.00060, 2: 0.00097, 3: 0.00066,
AZOLI_1103	1: 0.00268, 2: 0.00235, 3: 0.00214, 4: 0.00164,	1: 0.00173, 2: 0.00178, 3: 0.00159, 4: 0.00138,	AZOBR_40300		
AZOLI_1104	1: 0.00014, 4: 0.00043,		AZOBR_40301	4: 0.00012,	
AZOLI_1105	1: 0.00013,	1: 0.00010, 2: 0.00016, 3: 0.00011,	AZOBR_40303	1: 0.01121, 2: 0.01038, 3: 0.00863, 4: 0.00624, 5: 0.00646,	1: 0.00689, 2: 0.00755, 3: 0.00482,
AZOLI_1107	1: 0.00016, 2: 0.00027, 4: 0.00032,	1: 0.00016, 2: 0.00010, 3: 0.00017, 4: 0.00009,	AZOBR_40304	1: 0.00237, 2: 0.00244, 3: 0.00221, 4: 0.00212, 5: 0.00279,	1: 0.00214, 2: 0.00218, 3: 0.00193,
AZOLI_1109	1: 0.00012,		AZOBR_40305	1: 0.00044, 2: 0.00087, 3: 0.00075, 4: 0.00105, 5: 0.00105,	1: 0.00113, 2: 0.00075, 3: 0.00146,
AZOLI_1110	1: 0.00010, 2: 0.00010, 4: 0.00017,	1: 0.00014,	AZOBR_40306	1: 0.00573, 2: 0.00401, 3: 0.00423, 4: 0.00330, 5: 0.00314,	1: 0.00340, 2: 0.00424, 3: 0.00307,
AZOLI_1115		1: 0.00013, 2: 0.00016, 4: 0.00019,	AZOBR_40307	1: 0.00191, 2: 0.00084, 3: 0.00090, 4: 0.00117, 5: 0.00095,	1: 0.00231, 2: 0.00293, 3: 0.00165,
AZOLI_1116	1: 0.00058, 2: 0.00044, 4: 0.00099,	1: 0.00051, 2: 0.00028, 3: 0.00023, 4: 0.00075,	AZOBR_40309	2: 0.00016, 3: 0.00022, 4: 0.00018, 5: 0.00012,	2: 0.00019, 3: 0.00022,
AZOLI_1119	2: 0.00008,	1: 0.00011, 2: 0.00014, 3: 0.00006, 4: 0.00007,	AZOBR_40310		
AZOLI_1123	2: 0.00025,	1: 0.00008,	AZOBR_40311	1: 0.00101, 2: 0.00135, 3: 0.00091, 4: 0.00076, 5: 0.00091,	1: 0.00157, 2: 0.00136, 3: 0.00156,
AZOLI_1126	1: 0.00010,		AZOBR_40312		3: 0.00008,
AZOLI_1134	1: 0.00008, 2: 0.00003, 4: 0.00007,		AZOBR_40313	4: 0.00008, 5: 0.00008,	
AZOLI_1135	1: 0.00127, 2: 0.00116, 3: 0.00134, 4: 0.00118,	1: 0.00053, 2: 0.00077, 3: 0.00098, 4: 0.00059,	AZOBR_40314	2: 0.00010, 3: 0.00014, 4: 0.00008, 5: 0.00012,	1: 0.00005, 2: 0.00004, 3: 0.00010,
AZOLI_1137	1: 0.00015, 4: 0.00016,	1: 0.00006, 2: 0.00019, 3: 0.00009, 4: 0.00005,	AZOBR_40317	1: 0.00030, 3: 0.00017, 4: 0.00034, 5: 0.00021,	1: 0.00032, 2: 0.00029, 3: 0.00043,
AZOLI_1143	1: 0.00003, 4: 0.00003,	3: 0.00005, 4: 0.00003,	AZOBR_40319	1: 0.00048, 2: 0.00033, 3: 0.00032, 4: 0.00059, 5: 0.00038,	1: 0.00022, 2: 0.00030, 3: 0.00047,
AZOLI_1145	2: 0.00020, 4: 0.00033,	1: 0.00012, 2: 0.00019, 3: 0.00021, 4: 0.00013,	AZOBR_40320		
AZOLI_1150	4: 0.00002,	4: 0.00002,	AZOBR_40323	5: 0.00008,	2: 0.00005, 3: 0.00005,
AZOLI_1151	1: 0.00144, 2: 0.00242,	1: 0.00097, 2: 0.00094,	AZOBR_40324	1: 0.00007, 3: 0.00009, 4: 0.00020,	1: 0.00007, 2: 0.00013,

	3: 0.00143, 4: 0.00253,	3: 0.00051, 4: 0.00181,		5: 0.00007,	3: 0.00022,
AZOLI_1154	1: 0.00046, 2: 0.00075, 4: 0.00080,	1: 0.00094, 2: 0.00118, 3: 0.00080, 4: 0.00056,	AZOBR_40325	1: 0.00063, 2: 0.00307, 3: 0.00228, 4: 0.00663, 5: 0.00403,	1: 0.00283, 2: 0.00299, 3: 0.00216,
AZOLI_1158	1: 0.00005, 4: 0.00008,	1: 0.00005, 2: 0.00009, 4: 0.00008,	AZOBR_40326	1: 0.00180, 3: 0.00118, 4: 0.00343, 5: 0.00248,	1: 0.00044, 2: 0.00040, 3: 0.00112,
AZOLI_1161		1: 0.00031, 2: 0.00012, 3: 0.00019, 4: 0.00026,	AZOBR_40327	1: 0.00016, 4: 0.00007,	
AZOLI_1164	4: 0.00009,		AZOBR_40335		
AZOLI_1165	1: 0.00007, 2: 0.00020, 4: 0.00004,	1: 0.00015, 4: 0.00006,	AZOBR_40339	1: 0.00071, 2: 0.00089, 3: 0.00108, 4: 0.00098, 5: 0.00114,	1: 0.00209, 2: 0.00150, 3: 0.00158,
AZOLI_1166	1: 0.00005, 2: 0.00007, 4: 0.00011,	2: 0.00007,	AZOBR_40340	1: 0.00013, 4: 0.00014,	3: 0.00008,
AZOLI_1182	1: 0.00012, 2: 0.00012, 4: 0.00015,	2: 0.00017, 3: 0.00012, 4: 0.00025,	AZOBR_40341	4: 0.00004,	1: 0.00013, 3: 0.00003,
AZOLI_1183	2: 0.00008, 4: 0.00011,	2: 0.00012, 4: 0.00007,	AZOBR_40343		
AZOLI_1187	1: 0.00005, 2: 0.00017, 4: 0.00014,	1: 0.00029, 2: 0.00009, 3: 0.00015, 4: 0.00010,	AZOBR_40346		3: 0.00013,
AZOLI_1188	1: 0.00014, 2: 0.00039, 4: 0.00033,	1: 0.00059, 2: 0.00042, 3: 0.00062, 4: 0.00067,	AZOBR_40347		1: 0.00063, 2: 0.00052, 3: 0.00105,
AZOLI_1190	1: 0.00010, 3: 0.00029,	1: 0.00011, 2: 0.00010, 3: 0.00022, 4: 0.00009,	AZOBR_40349		
AZOLI_1191	1: 0.00019, 4: 0.00008,	1: 0.00029, 2: 0.00034, 3: 0.00028, 4: 0.00025,	AZOBR_40353	2: 0.00012, 4: 0.00023, 5: 0.00018,	1: 0.00013, 3: 0.00012,
AZOLI_1192	1: 0.00012, 2: 0.00022, 3: 0.00017, 4: 0.00042,	1: 0.00010, 2: 0.00033, 3: 0.00013, 4: 0.00031,	AZOBR_40354		
AZOLI_1196	1: 0.00017, 2: 0.00022, 4: 0.00015,	1: 0.00009, 4: 0.00011,	AZOBR_40356	1: 0.00010, 3: 0.00011, 4: 0.00007, 5: 0.00007,	1: 0.00006, 2: 0.00007, 3: 0.00008,
AZOLI_1207	1: 0.00058, 2: 0.00184, 3: 0.00041, 4: 0.00138,	1: 0.00118, 2: 0.00135, 3: 0.00086, 4: 0.00082,	AZOBR_40357	5: 0.00004,	3: 0.00006,
AZOLI_1210	1: 0.00033, 2: 0.00020,		AZOBR_40362	4: 0.00013, 5: 0.00006,	1: 0.00003, 3: 0.00012,
AZOLI_1212	1: 0.00019, 2: 0.00008, 4: 0.00013,		AZOBR_40363	1: 0.00051, 3: 0.00010,	1: 0.00007, 2: 0.00025,
AZOLI_1214		1: 0.00020, 2: 0.00032, 3: 0.00014, 4: 0.00034,	AZOBR_40364		
AZOLI_1215	2: 0.00010,	4: 0.00004,	AZOBR_40366	3: 0.00088,	
AZOLI_1216	1: 0.00052, 2: 0.00024, 3: 0.00020, 4: 0.00045,	1: 0.00043, 2: 0.00032, 3: 0.00015, 4: 0.00029,	AZOBR_40367		1: 0.00028, 3: 0.00083,
AZOLI_1219	2: 0.00015, 4: 0.00038,	2: 0.00024,	AZOBR_40368	1: 0.01295, 2: 0.01276, 3: 0.01375, 4: 0.01068, 5: 0.00947,	1: 0.00643, 2: 0.00871, 3: 0.00482,
AZOLI_1220	4: 0.00032,		AZOBR_40371	4: 0.00014,	2: 0.00012,
AZOLI_1222	1: 0.00050, 2: 0.00064, 3: 0.00095, 4: 0.00062,	1: 0.00087, 2: 0.00066, 3: 0.00145, 4: 0.00113,	AZOBR_40375	1: 0.00218, 2: 0.00374, 3: 0.00230, 4: 0.00106, 5: 0.00284,	1: 0.00362, 2: 0.00346, 3: 0.00319,
AZOLI_1224	1: 0.00004, 2: 0.00006, 3: 0.00018, 4: 0.00022,	1: 0.00023, 2: 0.00037, 3: 0.00025, 4: 0.00038,	AZOBR_40376	1: 0.00110, 2: 0.00114, 3: 0.00282, 4: 0.00114, 5: 0.00089,	1: 0.00141, 2: 0.00094, 3: 0.00148,
AZOLI_1226	1: 0.00053, 2: 0.00037, 4: 0.00085,	2: 0.00012, 4: 0.00011,	AZOBR_40378	1: 0.00014, 2: 0.00012, 3: 0.00012, 4: 0.00014, 5: 0.00005,	1: 0.00013, 2: 0.00010, 3: 0.00023,
AZOLI_1227		2: 0.00026, 4: 0.00011,	AZOBR_40379	1: 0.00161, 2: 0.00131, 3: 0.00148, 4: 0.00141, 5: 0.00111,	1: 0.00072, 2: 0.00068, 3: 0.00104,
AZOLI_1232	1: 0.00069, 2: 0.00093, 3: 0.00049, 4: 0.00179,	1: 0.00105, 2: 0.00124, 3: 0.00099, 4: 0.00060,	AZOBR_40382	1: 0.00044, 3: 0.00027, 4: 0.00022,	2: 0.00018, 3: 0.00012,
AZOLI_1234	1: 0.00042, 4: 0.00026,	1: 0.00025, 2: 0.00012, 3: 0.00032, 4: 0.00026,	AZOBR_40385	1: 0.00200, 2: 0.00417, 3: 0.00294, 4: 0.00312, 5: 0.00250,	1: 0.00186, 2: 0.00225, 3: 0.00158,
AZOLI_1235	1: 0.00011, 2: 0.00013, 3: 0.00008, 4: 0.00009,	1: 0.00023, 2: 0.00019, 3: 0.00040, 4: 0.00021,	AZOBR_40386	1: 0.00057, 2: 0.00025, 3: 0.00044, 4: 0.00039, 5: 0.00024,	1: 0.00007, 2: 0.00012, 3: 0.00011,
AZOLI_1243	2: 0.00011, 4: 0.00009,		AZOBR_40388	3: 0.00012,	2: 0.00010, 3: 0.00009,
AZOLI_1244	1: 0.00014, 2: 0.00021, 4: 0.00008,		AZOBR_40389	4: 0.00019, 5: 0.00019,	1: 0.00079, 2: 0.00090, 3: 0.00068,
AZOLI_1245	1: 0.00009,	1: 0.00009, 2: 0.00021, 3: 0.00041, 4: 0.00007,	AZOBR_40390	1: 0.00013, 2: 0.00033, 3: 0.00037, 4: 0.00042, 5: 0.00035,	1: 0.00006, 2: 0.00005, 3: 0.00015,
AZOLI_1245	2: 0.00020, 3: 0.00018,		AZOBR_40394	1: 0.00058, 2: 0.00042, 3: 0.00067, 4: 0.00029, 5: 0.00022,	1: 0.00017, 3: 0.00036,
AZOLI_1246	2: 0.00025, 4: 0.00022,	4: 0.00011,	AZOBR_40396	4: 0.00019, 5: 0.00008,	1: 0.00011, 2: 0.00020, 3: 0.00021,
AZOLI_1252	1: 0.00009, 2: 0.00028, 3: 0.00060, 4: 0.00016,	1: 0.00053, 2: 0.00033, 3: 0.00062, 4: 0.00021,	AZOBR_40401	1: 0.00138, 2: 0.00162, 3: 0.00141, 4: 0.00146, 5: 0.00196,	1: 0.00259, 2: 0.00288, 3: 0.00251,
AZOLI_1254	1: 0.00013, 2: 0.00008, 3: 0.00025, 4: 0.00006,	1: 0.00008, 2: 0.00015, 4: 0.00013,	AZOBR_40406	1: 0.00013, 2: 0.00014, 3: 0.00008, 4: 0.00016, 5: 0.00018,	1: 0.00008, 2: 0.00012, 3: 0.00018,
AZOLI_1255	1: 0.00011, 2: 0.00015, 4: 0.00021,	1: 0.00004, 3: 0.00012, 4: 0.00006,	AZOBR_40407	2: 0.00018,	1: 0.00013, 2: 0.00006, 3: 0.00010,
AZOLI_1259		2: 0.00017,	AZOBR_40408	4: 0.00007,	
AZOLI_1261	4: 0.00003,		AZOBR_40409	1: 0.00027, 2: 0.00030, 3: 0.00061, 4: 0.00042, 5: 0.00042,	1: 0.00026, 2: 0.00027, 3: 0.00023,
AZOLI_1263	4: 0.00010,	4: 0.00007,	AZOBR_40410	1: 0.00043, 2: 0.00019, 4: 0.00030,	1: 0.00014, 2: 0.00063,
AZOLI_1270	3: 0.00014, 4: 0.00004,	1: 0.00010, 2: 0.00025, 3: 0.00024, 4: 0.00039,	AZOBR_40411		3: 0.00026,
AZOLI_1274	1: 0.00183, 2: 0.00218, 3: 0.00082, 4: 0.00351,	1: 0.00388, 2: 0.00268, 3: 0.00339, 4: 0.00287,	AZOBR_40414	1: 0.00054, 2: 0.00044, 3: 0.00043, 4: 0.00045, 5: 0.00040,	1: 0.00009, 2: 0.00010, 3: 0.00020,
AZOLI_1275	1: 0.00882, 2: 0.00414, 3: 0.00525, 4: 0.00675,	1: 0.00580, 2: 0.00740, 3: 0.00881, 4: 0.00827,	AZOBR_40415		
AZOLI_1278	3: 0.00015, 4: 0.00005,	1: 0.00004, 2: 0.00008, 3: 0.00004, 4: 0.00008,	AZOBR_40420	4: 0.00012,	
AZOLI_1280	3: 0.00026, 4: 0.00011,	1: 0.00013, 4: 0.00013,	AZOBR_40424		
AZOLI_1281	1: 0.00007, 4: 0.00015,	2: 0.00010, 3: 0.00011, 4: 0.00027,	AZOBR_40426		
AZOLI_1291	1: 0.00114, 2: 0.00144, 3: 0.00064, 4: 0.00153,	1: 0.00194, 2: 0.00180, 3: 0.00190, 4: 0.00180,	AZOBR_40427		
AZOLI_1297	1: 0.00013,	1: 0.00022, 3: 0.00028, 4: 0.00034,	AZOBR_40428		
AZOLI_1300	1: 0.00041, 2: 0.00021, 3: 0.00038, 4: 0.00029,	1: 0.00021, 2: 0.00013,	AZOBR_40431		

AZOLI_1302		4: 0.00010,	AZOBR_40434		
AZOLI_1303	4: 0.00024,	1: 0.00069, 2: 0.00027, 3: 0.00059,	AZOBR_40435		
AZOLI_1304	1: 0.00045, 2: 0.00023, 4: 0.00020,		AZOBR_40436		
AZOLI_1307	1: 0.00037, 2: 0.00038, 4: 0.00056,	1: 0.00066, 2: 0.00037, 4: 0.00016,	AZOBR_50003		
AZOLI_1308	1: 0.00537, 2: 0.00535, 3: 0.00139, 4: 0.00384,	1: 0.00129, 2: 0.00117, 3: 0.00106, 4: 0.00110,	AZOBR_50007		3: 0.00014,
AZOLI_1310	1: 0.00050, 2: 0.00029, 4: 0.00012,	2: 0.00021, 4: 0.00012,	AZOBR_50008		
AZOLI_1312	1: 0.00008, 4: 0.00007,	4: 0.00011,	AZOBR_50012		
AZOLI_1313	1: 0.00013, 2: 0.00009, 4: 0.00053,	1: 0.00040, 2: 0.00077, 3: 0.00066, 4: 0.00064,	AZOBR_50013		1: 0.00012, 2: 0.00018, 3: 0.00007,
AZOLI_1314	1: 0.00025, 4: 0.00027,	3: 0.00013, 4: 0.00011,	AZOBR_50017	2: 0.00019, 3: 0.00018, 4: 0.00010,	1: 0.00027, 2: 0.00031, 3: 0.00057,
AZOLI_1316	1: 0.00006, 2: 0.00019, 4: 0.00013,	1: 0.00006, 2: 0.00004, 3: 0.00005, 4: 0.00005,	AZOBR_50021	4: 0.00024,	2: 0.00039,
AZOLI_1322	1: 0.00073, 2: 0.00047, 3: 0.00088, 4: 0.00054,	1: 0.00032, 2: 0.00020, 3: 0.00028, 4: 0.00077,	AZOBR_50022	1: 0.00068, 3: 0.00043, 4: 0.00046, 5: 0.00035,	
AZOLI_1323	1: 0.00021, 2: 0.00013,		AZOBR_50027	4: 0.00059, 5: 0.00069,	1: 0.00055, 3: 0.00063,
AZOLI_1329	1: 0.00040, 2: 0.00081, 3: 0.00258, 4: 0.00127,	1: 0.00179, 2: 0.00108, 3: 0.00190, 4: 0.00167,	AZOBR_50037	1: 0.00017, 5: 0.00008,	2: 0.00008,
AZOLI_1330	1: 0.00166, 2: 0.00199, 3: 0.00514, 4: 0.00155,	1: 0.00343, 2: 0.00305, 3: 0.00333, 4: 0.00305,	AZOBR_50054	4: 0.00008, 5: 0.00004,	3: 0.00003,
AZOLI_1331	1: 0.00078, 2: 0.00094, 3: 0.00256, 4: 0.00100,	1: 0.00131, 2: 0.00173, 3: 0.00154, 4: 0.00181,	AZOBR_60010		2: 0.00008,
AZOLI_1332	2: 0.00023,	2: 0.00022, 4: 0.00020,	AZOBR_70002		
AZOLI_1333	1: 0.00270, 2: 0.00277, 3: 0.00620, 4: 0.00252,	1: 0.00400, 2: 0.00371, 3: 0.00426, 4: 0.00345,	AZOBR_70006		1: 0.00031, 2: 0.00028, 3: 0.00041,
AZOLI_1335	1: 0.00043, 2: 0.00039, 3: 0.00075, 4: 0.00037,	1: 0.00061, 2: 0.00045, 3: 0.00061, 4: 0.00046,	AZOBR_70008		3: 0.00003,
AZOLI_1337	1: 0.00036, 4: 0.00142,		AZOBR_70010	1: 0.00054, 2: 0.00023, 3: 0.00038, 4: 0.00022, 5: 0.00031,	1: 0.00047, 2: 0.00054, 3: 0.00064,
AZOLI_1338	1: 0.00047, 2: 0.00062, 4: 0.00029,	1: 0.00062, 2: 0.00093, 3: 0.00051, 4: 0.00047,	AZOBR_70011	1: 0.00016, 3: 0.00008, 4: 0.00015, 5: 0.00006,	1: 0.00014, 2: 0.00014, 3: 0.00015,
AZOLI_1339	2: 0.00009, 3: 0.00008,	1: 0.00006, 2: 0.00011,	AZOBR_70012	2: 0.00051, 4: 0.00025,	3: 0.00009,
AZOLI_1342	1: 0.00026, 2: 0.00006, 3: 0.00012, 4: 0.00012,	1: 0.00009, 2: 0.00019, 4: 0.00015,	AZOBR_70014	3: 0.00006, 4: 0.00007, 5: 0.00003,	1: 0.00002, 3: 0.00009,
AZOLI_1343		1: 0.00007, 2: 0.00014, 3: 0.00005, 4: 0.00006,	AZOBR_70015	1: 0.00025, 2: 0.00045, 3: 0.00032, 4: 0.00017, 5: 0.00013,	1: 0.00012, 2: 0.00028, 3: 0.00012,
AZOLI_1344		3: 0.00006, 4: 0.00004,	AZOBR_70017	4: 0.00019,	1: 0.00009, 3: 0.00006,
AZOLI_1345	2: 0.00007, 3: 0.00021, 4: 0.00015,	1: 0.00022, 2: 0.00028, 3: 0.00044, 4: 0.00036,	AZOBR_70019	1: 0.00029, 2: 0.00017, 3: 0.00025,	2: 0.00014, 3: 0.00024,
AZOLI_1346		1: 0.00026, 4: 0.00022,	AZOBR_70024		
AZOLI_1348		4: 0.00008,	AZOBR_70025		1: 0.00054, 2: 0.00151, 3: 0.00053,
AZOLI_1349	1: 0.00234, 2: 0.00209, 3: 0.00058, 4: 0.00178,	1: 0.00238, 2: 0.00216, 3: 0.00178, 4: 0.00072,	AZOBR_70028	1: 0.00008, 2: 0.00009, 3: 0.00009, 4: 0.00011, 5: 0.00010,	
AZOLI_1350	3: 0.00012,	1: 0.00008, 2: 0.00022, 3: 0.00006, 4: 0.00007,	AZOBR_70031		
AZOLI_1351		3: 0.00024,	AZOBR_70032	1: 0.00075, 2: 0.00078, 3: 0.00068, 4: 0.00084, 5: 0.00095,	1: 0.00181, 2: 0.00148, 3: 0.00125,
AZOLI_1352	4: 0.00010,	3: 0.00030,	AZOBR_70033	1: 0.00037, 2: 0.00025, 3: 0.00032, 5: 0.00026,	1: 0.00048, 3: 0.00058,
AZOLI_1353	1: 0.00017, 2: 0.00008, 3: 0.00024,	1: 0.00025, 2: 0.00020, 3: 0.00013, 4: 0.00014,	AZOBR_70034		
AZOLI_1355		2: 0.00028, 3: 0.00013, 4: 0.00011,	AZOBR_70037		
AZOLI_1356	1: 0.00123, 2: 0.00088, 3: 0.00081, 4: 0.00090,	1: 0.00084, 2: 0.00074, 3: 0.00097, 4: 0.00066,	AZOBR_70039	1: 0.00021, 2: 0.00018, 3: 0.00019, 4: 0.00021, 5: 0.00012,	1: 0.00010, 2: 0.00027, 3: 0.00020,
AZOLI_1358		4: 0.00008,	AZOBR_70040		
AZOLI_1362	1: 0.00018, 2: 0.00025, 3: 0.00014, 4: 0.00018,	1: 0.00017, 2: 0.00016, 3: 0.00020, 4: 0.00011,	AZOBR_70041	1: 0.00234, 2: 0.00406, 3: 0.00177, 4: 0.00248, 5: 0.00236,	1: 0.00225, 2: 0.00098, 3: 0.00162,
AZOLI_1365	1: 0.00074, 2: 0.00082, 3: 0.00047, 4: 0.00081,	1: 0.00191, 2: 0.00112, 3: 0.00130, 4: 0.00087,	AZOBR_70042	1: 0.00530, 2: 0.00641, 3: 0.00354, 4: 0.00466, 5: 0.00489,	1: 0.01003, 2: 0.00766, 3: 0.00933,
AZOLI_1371	1: 0.00002,		AZOBR_70043	5: 0.00009,	
AZOLI_1372	4: 0.00006,		AZOBR_70045	4: 0.00006, 5: 0.00011,	
AZOLI_1375	1: 0.00018, 2: 0.00018, 4: 0.00016,	1: 0.00024, 2: 0.00035, 3: 0.00045, 4: 0.00026,	AZOBR_70046	1: 0.00178, 2: 0.00184, 3: 0.00182, 4: 0.00201, 5: 0.00122,	1: 0.00201, 2: 0.00198, 3: 0.00182,
AZOLI_1378		4: 0.00008,	AZOBR_70049	3: 0.00012, 5: 0.00010,	3: 0.00017,
AZOLI_1380	1: 0.00003, 4: 0.00005,	1: 0.00016, 2: 0.00019, 3: 0.00010, 4: 0.00019,	AZOBR_70050	1: 0.00018,	3: 0.00011,
AZOLI_1381	1: 0.00026,		AZOBR_70054	1: 0.00003, 2: 0.00008, 3: 0.00013, 4: 0.00005, 5: 0.00003,	1: 0.00054, 2: 0.00060, 3: 0.00062,
AZOLI_1383	1: 0.00004, 4: 0.00004,	1: 0.00005,	AZOBR_70055	4: 0.00056, 5: 0.00046,	1: 0.00183, 2: 0.00166, 3: 0.00191,
AZOLI_1385	2: 0.00010,	2: 0.00005, 3: 0.00005,	AZOBR_70056		
AZOLI_1386	1: 0.00016, 4: 0.00010,		AZOBR_70057		3: 0.00008,
AZOLI_1390	1: 0.01102, 2: 0.00690, 3: 0.00081, 4: 0.00381,	1: 0.00047, 2: 0.00062, 3: 0.00068, 4: 0.00045,	AZOBR_70058		1: 0.00072, 2: 0.00025, 3: 0.00056,
AZOLI_1394	1: 0.00007,	4: 0.00015,	AZOBR_70063	3: 0.00004,	2: 0.00003,
AZOLI_1395	2: 0.00006, 4: 0.00007,		AZOBR_70064	2: 0.00014, 3: 0.00023, 5: 0.00015,	2: 0.00016,
AZOLI_1396	1: 0.00040, 2: 0.00048, 4: 0.00061,	1: 0.00048, 2: 0.00031,	AZOBR_70065	1: 0.00055, 2: 0.00048, 3: 0.00078, 4: 0.00119, 5: 0.00057,	1: 0.00140, 2: 0.00073, 3: 0.00096,
AZOLI_1397	1: 0.00019, 2: 0.00011,		AZOBR_70066	1: 0.00060, 2: 0.00059, 3: 0.00023, 4: 0.00074, 5: 0.00065,	1: 0.00258, 2: 0.00303, 3: 0.00155,
AZOLI_1398	4: 0.00007,		AZOBR_70073		2: 0.00004, 3: 0.00004,
AZOLI_1400	1: 0.00044, 2: 0.00035, 4: 0.00060,	2: 0.00013, 3: 0.00019, 4: 0.00011,	AZOBR_70077	1: 0.00046, 2: 0.00020, 3: 0.00020, 4: 0.00036, 5: 0.00028,	1: 0.00011, 2: 0.00017, 3: 0.00029,

AZOLI_1401	1: 0.00045, 2: 0.00081, 4: 0.00078,	1: 0.00046, 2: 0.00047, 3: 0.00034, 4: 0.00044,	AZOBR_70078	1: 0.00048, 2: 0.00061, 3: 0.00041, 4: 0.00052, 5: 0.00041,	1: 0.00027, 2: 0.00016, 3: 0.00020,
AZOLI_1403	4: 0.00009,		AZOBR_70079	1: 0.00078, 2: 0.00137, 3: 0.00038, 4: 0.00160, 5: 0.00206,	1: 0.00121, 2: 0.00073, 3: 0.00172,
AZOLI_1408	1: 0.00041, 2: 0.00062,	3: 0.00033,	AZOBR_70080		
AZOLI_1409	1: 0.00825, 2: 0.00745, 3: 0.01560, 4: 0.00725,	1: 0.00685, 2: 0.00698, 3: 0.00590, 4: 0.00753,	AZOBR_70081	3: 0.00013,	1: 0.00010,
AZOLI_1411	1: 0.00010,	4: 0.00009,	AZOBR_70082		
AZOLI_1412	1: 0.00091, 2: 0.00057, 3: 0.00030, 4: 0.00067,	1: 0.00121, 2: 0.00048, 3: 0.00106, 4: 0.00079,	AZOBR_70083		1: 0.00009,
AZOLI_1413	1: 0.00041, 2: 0.00029, 4: 0.00036,	1: 0.00013, 2: 0.00048, 3: 0.00018, 4: 0.00029,	AZOBR_70085		
AZOLI_1414	1: 0.00020,		AZOBR_70086		1: 0.00008, 3: 0.00019,
AZOLI_1415	1: 0.00036, 2: 0.00036, 3: 0.00076, 4: 0.00139,	1: 0.00091, 2: 0.00140, 3: 0.00106, 4: 0.00116,	AZOBR_70088		
AZOLI_1416	1: 0.00005,		AZOBR_70089		2: 0.00005,
AZOLI_1421	1: 0.00055, 2: 0.00039, 4: 0.00029,	1: 0.00034, 2: 0.00049, 3: 0.00042, 4: 0.00043,	AZOBR_70090		1: 0.00035, 3: 0.00020,
AZOLI_1423		2: 0.00010, 3: 0.00011, 4: 0.00026,	AZOBR_70092		2: 0.00006,
AZOLI_1428	1: 0.00056, 2: 0.00041, 4: 0.00029,	1: 0.00047, 2: 0.00058, 3: 0.00033, 4: 0.00021,	AZOBR_70095		1: 0.00006,
AZOLI_1430	1: 0.00057, 2: 0.00057, 4: 0.00049,	2: 0.00092, 3: 0.00162, 4: 0.00049,	AZOBR_70096	1: 0.00016, 2: 0.00016, 3: 0.00014, 4: 0.00022, 5: 0.00013,	1: 0.00003, 2: 0.00012, 3: 0.00012,
AZOLI_1432	1: 0.00111, 2: 0.00131, 3: 0.00052, 4: 0.00053,	1: 0.00075, 2: 0.00060, 3: 0.00066, 4: 0.00032,	AZOBR_70098	1: 0.00064, 2: 0.00032, 3: 0.00043, 4: 0.00069, 5: 0.00044,	1: 0.00037, 2: 0.00099, 3: 0.00071,
AZOLI_1433	1: 0.00009, 2: 0.00015, 4: 0.00018,	1: 0.00009, 2: 0.00018, 3: 0.00013, 4: 0.00021,	AZOBR_70099	4: 0.00012, 5: 0.00008,	1: 0.00012,
AZOLI_1438	4: 0.00027,		AZOBR_70100		
AZOLI_1439	1: 0.00027, 2: 0.00022, 4: 0.00021,	1: 0.00010, 2: 0.00007, 3: 0.00013, 4: 0.00015,	AZOBR_70102		1: 0.00013, 2: 0.00003, 3: 0.00008,
AZOLI_1442	2: 0.00021, 4: 0.00036,	1: 0.00021, 2: 0.00013, 3: 0.00015, 4: 0.00018,	AZOBR_70104		1: 0.00038, 2: 0.00043, 3: 0.00018,
AZOLI_1443	1: 0.00010, 2: 0.00006, 4: 0.00011,	1: 0.00010, 4: 0.00008,	AZOBR_70105		3: 0.00084,
AZOLI_1444	4: 0.00007,	2: 0.00005,	AZOBR_70106		1: 0.00037, 2: 0.00051, 3: 0.00052,
AZOLI_1447		1: 0.00002, 2: 0.00002, 4: 0.00006,	AZOBR_70107		1: 0.00010, 2: 0.00018, 3: 0.00015,
AZOLI_1461	1: 0.00024, 2: 0.00034, 4: 0.00029,	1: 0.00010, 2: 0.00019, 3: 0.00028, 4: 0.00025,	AZOBR_70108		1: 0.00128, 2: 0.00086, 3: 0.00134,
AZOLI_1463	1: 0.00024, 4: 0.00062,	3: 0.00038, 4: 0.00021,	AZOBR_70109		
AZOLI_1464	1: 0.00051, 2: 0.00032, 4: 0.00038,	1: 0.00071, 2: 0.00074, 3: 0.00095, 4: 0.00060,	AZOBR_70112		1: 0.00039, 2: 0.00011, 3: 0.00034,
AZOLI_1475	4: 0.00004,		AZOBR_70113		1: 0.00165, 2: 0.00180, 3: 0.00157,
AZOLI_1478	1: 0.00038, 2: 0.00045, 3: 0.00044, 4: 0.00068,	1: 0.00042, 2: 0.00052, 3: 0.00037, 4: 0.00027,	AZOBR_70117		1: 0.00086, 2: 0.00033, 3: 0.00047,
AZOLI_1484	2: 0.00025,		AZOBR_70118		1: 0.00287, 2: 0.00342, 3: 0.00186,
AZOLI_1487	1: 0.00017, 2: 0.00026,		AZOBR_70119		1: 0.00036, 3: 0.00023,
AZOLI_1491	1: 0.00037, 2: 0.00088, 4: 0.00065,	1: 0.00013,	AZOBR_70120		1: 0.00048, 2: 0.00068, 3: 0.00069,
AZOLI_1492	1: 0.00002, 2: 0.00002, 4: 0.00002,	2: 0.00002, 3: 0.00002, 4: 0.00005,	AZOBR_70121		
AZOLI_1495	1: 0.00029, 2: 0.00039, 4: 0.00062,	1: 0.00049, 2: 0.00033, 3: 0.00052, 4: 0.00046,	AZOBR_70122		3: 0.00006,
AZOLI_1497		2: 0.00007,	AZOBR_70123	1: 0.00006, 4: 0.00012, 5: 0.00010,	
AZOLI_1504	1: 0.00074, 2: 0.00026, 4: 0.00015,		AZOBR_70126		1: 0.00116, 2: 0.00112, 3: 0.00118,
AZOLI_1505	1: 0.00008, 2: 0.00009,		AZOBR_70127		1: 0.00031, 2: 0.00071, 3: 0.00058,
AZOLI_1507	1: 0.00018, 2: 0.00028, 4: 0.00047,	1: 0.00014, 2: 0.00040, 3: 0.00024, 4: 0.00020,	AZOBR_70128		1: 0.00959, 2: 0.01108, 3: 0.00456,
AZOLI_1508		3: 0.00010, 4: 0.00005,	AZOBR_70134		1: 0.00046, 3: 0.00044,
AZOLI_1509	1: 0.00259, 2: 0.00317, 3: 0.00277, 4: 0.00142,	1: 0.00343, 2: 0.00215, 3: 0.00236, 4: 0.00251,	AZOBR_70136	4: 0.00019,	
AZOLI_1510	2: 0.00019, 4: 0.00011,	1: 0.00052, 2: 0.00044, 3: 0.00014, 4: 0.00033,	AZOBR_70137		1: 0.00038, 2: 0.00036, 3: 0.00022,
AZOLI_1511	4: 0.00009,	2: 0.00013, 3: 0.00018, 4: 0.00011,	AZOBR_70138		
AZOLI_1512	1: 0.00040, 2: 0.00052, 3: 0.00016, 4: 0.00092,	1: 0.00052, 2: 0.00057, 3: 0.00057, 4: 0.00040,	AZOBR_70140	3: 0.00054, 4: 0.00029,	1: 0.00216, 2: 0.00214, 3: 0.00209,
AZOLI_1516	1: 0.00004, 2: 0.00004, 4: 0.00006,	1: 0.00009, 2: 0.00017, 3: 0.00007, 4: 0.00013,	AZOBR_70142		1: 0.00090, 2: 0.00185, 3: 0.00079,
AZOLI_1521	4: 0.00005,		AZOBR_70144		2: 0.00008,
AZOLI_1527	2: 0.00061, 4: 0.00052,	3: 0.00049,	AZOBR_70146		1: 0.00030, 3: 0.00158,
AZOLI_1534	1: 0.00001, 4: 0.00002,	4: 0.00002,	AZOBR_70147		
AZOLI_1538	4: 0.00007,	2: 0.00010,	AZOBR_70149		1: 0.00056, 2: 0.00030, 3: 0.00075,
AZOLI_1540	1: 0.00032, 2: 0.00022,	2: 0.00021, 3: 0.00023,	AZOBR_70150		
AZOLI_1545	1: 0.00221, 2: 0.00158, 3: 0.00273, 4: 0.00167,	1: 0.00141, 2: 0.00149, 3: 0.00124, 4: 0.00141,	AZOBR_70155		
AZOLI_1546	1: 0.00023, 2: 0.00029, 3: 0.00026, 4: 0.00045,	1: 0.00050, 2: 0.00041, 3: 0.00050, 4: 0.00036,	AZOBR_70159	4: 0.00006,	2: 0.00013, 3: 0.00011,
AZOLI_1547	1: 0.00027, 2: 0.00024, 3: 0.00045, 4: 0.00021,	1: 0.00049, 2: 0.00026, 3: 0.00029, 4: 0.00023,	AZOBR_70161	1: 0.00389, 2: 0.00156, 3: 0.00422, 4: 0.00317, 5: 0.00244,	1: 0.00249, 2: 0.00180, 3: 0.00242,
AZOLI_1548	2: 0.00003, 3: 0.00014, 4: 0.00007,		AZOBR_70169		1: 0.00012, 2: 0.00018, 3: 0.00015,
AZOLI_1549		4: 0.00021,	AZOBR_70172	5: 0.00004,	3: 0.00006,

AZOLI_1552	4: 0.00031,	1: 0.00042, 2: 0.00023, 3: 0.00045, 4: 0.00036,	AZOBR_70173	1: 0.00022, 2: 0.00016, 3: 0.00027, 4: 0.00015, 5: 0.00015,	1: 0.00026, 2: 0.00043, 3: 0.00017,
AZOLI_1554	1: 0.00004, 4: 0.00005,	3: 0.00004, 4: 0.00003,	AZOBR_70174	4: 0.00009, 5: 0.00009,	1: 0.00025, 3: 0.00037,
AZOLI_1557		1: 0.00034, 2: 0.00039, 3: 0.00065, 4: 0.00058,	AZOBR_70176	1: 0.00052, 2: 0.00023, 3: 0.00059, 4: 0.00096, 5: 0.00054,	1: 0.00033, 2: 0.00038, 3: 0.00032,
AZOLI_1558	1: 0.00003,		AZOBR_70177		
AZOLI_1559	1: 0.00005, 2: 0.00012, 4: 0.00015,	1: 0.00007, 2: 0.00004, 3: 0.00004, 4: 0.00006,	AZOBR_70179		
AZOLI_1560	1: 0.00015, 2: 0.00015, 3: 0.00108, 4: 0.00018,	1: 0.00047, 2: 0.00025, 3: 0.00016, 4: 0.00026,	AZOBR_70181	1: 0.00028, 2: 0.00012, 3: 0.00036, 4: 0.00031, 5: 0.00010,	1: 0.00004, 2: 0.00015, 3: 0.00009,
AZOLI_1561	1: 0.00034, 2: 0.00012, 3: 0.00075, 4: 0.00025,	1: 0.00022, 2: 0.00033, 3: 0.00018, 4: 0.00013,	AZOBR_70182		
AZOLI_1562	4: 0.00027,		AZOBR_70185	1: 0.00018, 2: 0.00016,	
AZOLI_1563	1: 0.00140, 2: 0.00152, 3: 0.00190, 4: 0.00067,	1: 0.00147, 2: 0.00132, 3: 0.00117, 4: 0.00157,	AZOBR_70186		
AZOLI_1571	4: 0.00010,		AZOBR_70187	5: 0.00009,	3: 0.00012,
AZOLI_1572	1: 0.00037, 2: 0.00038, 3: 0.00043, 4: 0.00026,	1: 0.00034, 2: 0.00027, 3: 0.00029, 4: 0.00021,	AZOBR_70188	4: 0.00014, 5: 0.00014,	
AZOLI_1573	2: 0.00015, 4: 0.00025,		AZOBR_70189	4: 0.00009,	
AZOLI_1574	1: 0.00045, 2: 0.00057, 4: 0.00062,	1: 0.00015, 2: 0.00007, 4: 0.00026,	AZOBR_70190	1: 0.00034, 2: 0.00020, 3: 0.00039, 5: 0.00016,	
AZOLI_1576	2: 0.00017,		AZOBR_70193	4: 0.00032,	1: 0.00059, 2: 0.00135, 3: 0.00029,
AZOLI_1579	1: 0.00009,		AZOBR_80014		1: 0.00007, 2: 0.00008, 3: 0.00007,
AZOLI_1580	1: 0.00013, 4: 0.00007,	1: 0.00009, 2: 0.00013, 3: 0.00014, 4: 0.00037,	AZOBR_90006		1: 0.00014,
AZOLI_1583	1: 0.00370, 2: 0.00236, 3: 0.00075, 4: 0.00285,	1: 0.00413, 2: 0.00414, 3: 0.00383, 4: 0.00424,	AZOBR_90008		2: 0.00015,
AZOLI_1584	1: 0.00009, 4: 0.00007,		AZOBR_90012		1: 0.00006, 3: 0.00010,
AZOLI_1585	1: 0.00024, 2: 0.00048, 4: 0.00061,	1: 0.00016,	AZOBR_90018		2: 0.00017, 3: 0.00008,
AZOLI_1596	1: 0.00007, 2: 0.00009, 3: 0.00016, 4: 0.00004,	2: 0.00007, 3: 0.00020, 4: 0.00016,	AZOBR_90020		1: 0.00095, 2: 0.00104, 3: 0.00096,
AZOLI_1597	4: 0.00005,		AZOBR_100001		1: 0.00062, 2: 0.00099, 3: 0.00036,
AZOLI_1599	1: 0.00024, 2: 0.00036,		AZOBR_100004		
AZOLI_1603	1: 0.00070, 2: 0.00035, 3: 0.00061, 4: 0.00023,	2: 0.00017, 4: 0.00023,	AZOBR_100006	3: 0.00038,	
AZOLI_1605	1: 0.00177, 2: 0.00096, 3: 0.00218, 4: 0.00145,	1: 0.00175, 2: 0.00138, 3: 0.00161, 4: 0.00236,	AZOBR_100009		2: 0.00007,
AZOLI_1606	1: 0.00110, 2: 0.00078, 3: 0.00166, 4: 0.00063,	1: 0.00175, 2: 0.00126, 3: 0.00138, 4: 0.00143,	AZOBR_100010		3: 0.00027,
AZOLI_1608	3: 0.00011, 4: 0.00017,		AZOBR_100012		1: 0.00032, 3: 0.00062,
AZOLI_1613	1: 0.00146, 2: 0.00162, 3: 0.00012, 4: 0.00078,	1: 0.00051, 2: 0.00036, 3: 0.00021, 4: 0.00056,	AZOBR_100014	2: 0.00011, 4: 0.00014, 5: 0.00006,	1: 0.00010,
AZOLI_1614	4: 0.00011,		AZOBR_100015	1: 0.00076, 2: 0.00059, 3: 0.00064, 4: 0.00040, 5: 0.00046,	1: 0.00054, 2: 0.00079, 3: 0.00037,
AZOLI_1616		4: 0.00014,	AZOBR_100016	3: 0.00075, 4: 0.00106, 5: 0.00046,	3: 0.00041,
AZOLI_1623	1: 0.00031, 2: 0.00021,		AZOBR_100017	1: 0.00008, 2: 0.00007, 3: 0.00007, 4: 0.00011,	1: 0.00008, 2: 0.00009, 3: 0.00012,
AZOLI_1625	1: 0.00012, 2: 0.00037, 4: 0.00007,	4: 0.00010,	AZOBR_100018		
AZOLI_1626	1: 0.00085, 2: 0.00171, 4: 0.00110,	1: 0.00057, 2: 0.00041,	AZOBR_100021	4: 0.00003,	
AZOLI_1627	1: 0.00041, 2: 0.00062, 3: 0.00078, 4: 0.00098,	1: 0.00069, 2: 0.00056, 3: 0.00064, 4: 0.00061,	AZOBR_100023	4: 0.00022,	3: 0.00008,
AZOLI_1628		4: 0.00016,	AZOBR_100025	2: 0.00031, 3: 0.00024, 4: 0.00024, 5: 0.00044,	1: 0.00104, 2: 0.00113, 3: 0.00106,
AZOLI_1629	2: 0.00010, 4: 0.00024,		AZOBR_100027	1: 0.00065, 2: 0.00042, 3: 0.00037, 4: 0.00051, 5: 0.00060,	2: 0.00050, 3: 0.00011,
AZOLI_1631	1: 0.00010, 2: 0.00016, 4: 0.00013,	1: 0.00009, 2: 0.00005, 3: 0.00009, 4: 0.00013,	AZOBR_100028		1: 0.00030, 2: 0.00034, 3: 0.00073,
AZOLI_1632	1: 0.00069, 2: 0.00061,		AZOBR_100032		3: 0.00003,
AZOLI_1634	1: 0.00081, 2: 0.00056, 3: 0.00018, 4: 0.00067,	1: 0.00145, 2: 0.00124, 3: 0.00161, 4: 0.00090,	AZOBR_100035	1: 0.00065, 2: 0.00104, 3: 0.00067, 4: 0.00072, 5: 0.00117,	1: 0.00101, 2: 0.00076, 3: 0.00065,
AZOLI_1636	4: 0.00007,	2: 0.00008,	AZOBR_100037	1: 0.00042, 4: 0.00022, 5: 0.00029,	1: 0.00020, 2: 0.00038, 3: 0.00030,
AZOLI_1639		2: 0.00012, 3: 0.00009, 4: 0.00015,	AZOBR_100038	4: 0.00012, 5: 0.00019,	
AZOLI_1640	4: 0.00007,		AZOBR_100039		1: 0.00008, 2: 0.00010, 3: 0.00016,
AZOLI_1641	2: 0.00009,	1: 0.00023, 2: 0.00039, 4: 0.00042,	AZOBR_100040		3: 0.00004,
AZOLI_1643		4: 0.00015,	AZOBR_100041		1: 0.00013, 2: 0.00026, 3: 0.00016,
AZOLI_1645		2: 0.00004,	AZOBR_100047	1: 0.00087,	1: 0.00074, 3: 0.00054,
AZOLI_1660	1: 0.00002,		AZOBR_100048	1: 0.00015, 2: 0.00016, 3: 0.00012, 4: 0.00012, 5: 0.00010,	1: 0.00007, 3: 0.00007,
AZOLI_1668	1: 0.00090, 2: 0.00057, 4: 0.00098,	3: 0.00049, 4: 0.00029,	AZOBR_100049	4: 0.00009,	
AZOLI_1669	2: 0.00007,	3: 0.00007,	AZOBR_100050		
AZOLI_1670	1: 0.00148, 2: 0.00130, 3: 0.00102, 4: 0.00144,	1: 0.00082, 2: 0.00076, 3: 0.00098, 4: 0.00088,	AZOBR_100054	1: 0.00087, 2: 0.00029, 3: 0.00046, 4: 0.00045, 5: 0.00053,	1: 0.00021, 2: 0.00016, 3: 0.00020,
AZOLI_1671	1: 0.00029, 2: 0.00043, 4: 0.00049,	1: 0.00029, 3: 0.00061, 4: 0.00025,	AZOBR_100055	2: 0.00004, 4: 0.00010,	3: 0.00003,
AZOLI_1673	1: 0.00083, 2: 0.00051, 3: 0.00124, 4: 0.00107,	1: 0.00052, 2: 0.00072, 3: 0.00094, 4: 0.00096,	AZOBR_100059	1: 0.00025, 2: 0.00004, 3: 0.00029, 4: 0.00009, 5: 0.00006,	1: 0.00015, 2: 0.00041, 3: 0.00024,
AZOLI_1674	1: 0.00014, 4: 0.00019,	2: 0.00011, 3: 0.00012, 4: 0.00012,	AZOBR_100064	4: 0.00012,	

AZOLI_1676	1: 0.00457, 2: 0.00274, 3: 0.00557, 4: 0.00314,	1: 0.00244, 2: 0.00288, 3: 0.00225, 4: 0.00301,	AZOBR_100070		3: 0.00006,
AZOLI_1677	1: 0.00546, 2: 0.00577, 4: 0.00405,	1: 0.00206, 2: 0.00310, 3: 0.00218, 4: 0.00209,	AZOBR_100071		
AZOLI_1679	1: 0.00327, 2: 0.00311, 3: 0.00247, 4: 0.00313,	1: 0.00294, 2: 0.00419, 3: 0.00305, 4: 0.00235,	AZOBR_100075		
AZOLI_1681	1: 0.00061, 2: 0.00053, 3: 0.00082, 4: 0.00072,	1: 0.00042, 2: 0.00043, 3: 0.00072, 4: 0.00091,	AZOBR_100079	1: 0.00089, 2: 0.00141, 3: 0.00121, 4: 0.00146, 5: 0.00147,	1: 0.00034, 3: 0.00044,
AZOLI_1682	3: 0.00101,	1: 0.00127, 2: 0.00158, 3: 0.00183, 4: 0.00194,	AZOBR_100080	1: 0.00185, 2: 0.00176, 3: 0.00193, 4: 0.00213, 5: 0.00114,	1: 0.00167, 2: 0.00189, 3: 0.00122,
AZOLI_1683		2: 0.00007,	AZOBR_100082		1: 0.00016, 2: 0.00018, 3: 0.00017,
AZOLI_1690		4: 0.00006,	AZOBR_100083		1: 0.00078, 2: 0.00078, 3: 0.00152,
AZOLI_1704	1: 0.00047, 2: 0.00071, 3: 0.00066, 4: 0.00041,	1: 0.00066, 2: 0.00017, 3: 0.00044, 4: 0.00036,	AZOBR_100084	1: 0.00021, 2: 0.00037, 3: 0.00041, 4: 0.00015, 5: 0.00015,	1: 0.00010, 2: 0.00027, 3: 0.00013,
AZOLI_1713	4: 0.00004,	1: 0.00010, 2: 0.00005, 3: 0.00005, 4: 0.00005,	AZOBR_100085	1: 0.00065, 2: 0.00172, 3: 0.00111, 4: 0.00283, 5: 0.00120,	1: 0.00056, 3: 0.00094,
AZOLI_1721	1: 0.00011, 4: 0.00007,	1: 0.00009, 2: 0.00020, 3: 0.00006, 4: 0.00015,	AZOBR_100087	5: 0.00011,	3: 0.00025,
AZOLI_1722	1: 0.00010, 2: 0.00010, 4: 0.00021,	4: 0.00009,	AZOBR_100088		3: 0.00012,
AZOLI_1723	3: 0.00038, 4: 0.00008,	1: 0.00006, 2: 0.00015, 4: 0.00010,	AZOBR_100090	1: 0.00015, 3: 0.00013, 4: 0.00063, 5: 0.00058,	
AZOLI_1724	1: 0.00009, 2: 0.00009, 4: 0.00009,	4: 0.00008,	AZOBR_100091	1: 0.00014, 2: 0.00025, 3: 0.00008, 4: 0.00019, 5: 0.00019,	1: 0.00009, 3: 0.00012,
AZOLI_1725	4: 0.00011,		AZOBR_100092		
AZOLI_1727		1: 0.00025, 2: 0.00024, 3: 0.00027, 4: 0.00043,	AZOBR_100095	1: 0.00041, 2: 0.00111, 3: 0.00065, 4: 0.00078, 5: 0.00056,	1: 0.00277, 2: 0.00158, 3: 0.00188,
AZOLI_1728	1: 0.00018, 4: 0.00015,	1: 0.00018,	AZOBR_100097		
AZOLI_1732	1: 0.00008, 2: 0.00022, 4: 0.00011,	1: 0.00011, 2: 0.00013, 3: 0.00013, 4: 0.00007,	AZOBR_100099	1: 0.00033, 2: 0.00030, 3: 0.00025, 4: 0.00031, 5: 0.00015,	1: 0.00043, 2: 0.00027, 3: 0.00037,
AZOLI_1733	1: 0.00007, 4: 0.00014,		AZOBR_100102	2: 0.00042, 4: 0.00054, 5: 0.00060,	1: 0.00040, 2: 0.00080, 3: 0.00078,
AZOLI_1734	3: 0.00014, 4: 0.00009,		AZOBR_100103	1: 0.00018, 2: 0.00016, 3: 0.00024, 4: 0.00013, 5: 0.00035,	1: 0.00018, 2: 0.00023, 3: 0.00026,
AZOLI_1748	1: 0.00030, 4: 0.00051,		AZOBR_100105	1: 0.00054, 2: 0.00068, 3: 0.00046, 4: 0.00075, 5: 0.00055,	1: 0.00132, 2: 0.00125, 3: 0.00097,
AZOLI_1755		4: 0.00008,	AZOBR_100106	1: 0.00024, 2: 0.00068, 3: 0.00061, 4: 0.00036, 5: 0.00053,	1: 0.00012, 2: 0.00017, 3: 0.00006,
AZOLI_1792	2: 0.00067, 4: 0.00029,	2: 0.00054, 4: 0.00029,	AZOBR_100108		3: 0.00008,
AZOLI_1798	1: 0.00041, 2: 0.00066, 3: 0.00046, 4: 0.00049,	1: 0.00066, 2: 0.00024, 3: 0.00044, 4: 0.00014,	AZOBR_100109		1: 0.00005, 2: 0.00005, 3: 0.00007,
AZOLI_1799	1: 0.00016, 3: 0.00045, 4: 0.00048,	1: 0.00016, 2: 0.00031, 3: 0.00078, 4: 0.00062,	AZOBR_100110	1: 0.00010, 2: 0.00017, 3: 0.00019, 4: 0.00017, 5: 0.00010,	1: 0.00005, 2: 0.00007, 3: 0.00012,
AZOLI_1807	1: 0.00169, 2: 0.00140, 4: 0.00093,	1: 0.00023, 2: 0.00023, 3: 0.00025, 4: 0.00033,	AZOBR_100111	1: 0.00078, 2: 0.00084, 3: 0.00066, 4: 0.00060, 5: 0.00042,	1: 0.00033, 2: 0.00044, 3: 0.00059,
AZOLI_1808	1: 0.00006, 2: 0.00006,	1: 0.00016, 2: 0.00006, 3: 0.00017, 4: 0.00016,	AZOBR_100112	1: 0.00450, 2: 0.00329, 3: 0.00394, 4: 0.00313, 5: 0.00301,	1: 0.00206, 2: 0.00200, 3: 0.00170,
AZOLI_1811	3: 0.00048,	1: 0.00100, 2: 0.00063, 3: 0.00124, 4: 0.00107,	AZOBR_100113	2: 0.00011, 3: 0.00016, 4: 0.00034, 5: 0.00030,	1: 0.00020, 2: 0.00027, 3: 0.00023,
AZOLI_1812	4: 0.00004,	2: 0.00004, 3: 0.00005, 4: 0.00010,	AZOBR_100116	1: 0.00017, 3: 0.00018, 4: 0.00046, 5: 0.00014,	1: 0.00040, 2: 0.00046, 3: 0.00060,
AZOLI_1815	1: 0.00018, 2: 0.00017, 3: 0.00009, 4: 0.00027,	1: 0.00021, 2: 0.00012, 3: 0.00026, 4: 0.00017,	AZOBR_100120		1: 0.00025, 2: 0.00011, 3: 0.00021,
AZOLI_1817	1: 0.00021, 2: 0.00013, 3: 0.00088, 4: 0.00022,	1: 0.00013, 2: 0.00020, 3: 0.00054, 4: 0.00018,	AZOBR_100124	1: 0.00017, 3: 0.00022, 4: 0.00036, 5: 0.00027,	1: 0.00073, 2: 0.00108, 3: 0.00079,
AZOLI_1818	2: 0.00026, 4: 0.00017,	1: 0.00027, 3: 0.00014, 4: 0.00017,	AZOBR_100128		
AZOLI_1819	1: 0.00042, 2: 0.00056, 3: 0.00048, 4: 0.00034,	1: 0.00083, 2: 0.00079, 3: 0.00065, 4: 0.00057,	AZOBR_100129		
AZOLI_1823		1: 0.00050, 3: 0.00026, 4: 0.00021,	AZOBR_100130	1: 0.00010,	1: 0.00016, 2: 0.00007, 3: 0.00024,
AZOLI_1824		3: 0.00008,	AZOBR_100131	4: 0.00018,	3: 0.00008,
AZOLI_1833	4: 0.00004,	1: 0.00005, 4: 0.00004,	AZOBR_100132	4: 0.00008,	3: 0.00011,
AZOLI_1834	2: 0.00034, 4: 0.00022,	1: 0.00034, 2: 0.00041, 3: 0.00036,	AZOBR_100136	1: 0.00030, 2: 0.00027, 3: 0.00042, 4: 0.00044, 5: 0.00076,	1: 0.00041, 2: 0.00036, 3: 0.00075,
AZOLI_1836	1: 0.00037, 2: 0.00065, 3: 0.00010, 4: 0.00023,	1: 0.00024, 2: 0.00020, 3: 0.00007, 4: 0.00009,	AZOBR_100141	1: 0.00047, 2: 0.00035, 3: 0.00049, 4: 0.00045, 5: 0.00050,	1: 0.00046, 2: 0.00040, 3: 0.00058,
AZOLI_1837	2: 0.00008, 4: 0.00014,		AZOBR_100142	5: 0.00004,	
AZOLI_1838	1: 0.00060, 4: 0.00169,	2: 0.00177, 4: 0.00065,	AZOBR_100144	4: 0.00006,	1: 0.00035, 2: 0.00040, 3: 0.00059,
AZOLI_1843	1: 0.01099, 2: 0.00868, 3: 0.00718, 4: 0.00646,	1: 0.00590, 2: 0.00591, 3: 0.00581, 4: 0.00578,	AZOBR_100145		
AZOLI_1844	1: 0.00191, 2: 0.00260, 3: 0.00236, 4: 0.00176,	1: 0.00102, 2: 0.00108, 3: 0.00151, 4: 0.00171,	AZOBR_100149		1: 0.00004, 3: 0.00002,
AZOLI_1845	1: 0.00137, 2: 0.00199, 3: 0.00507, 4: 0.00362,	1: 0.00400, 2: 0.00259, 3: 0.00570, 4: 0.00348,	AZOBR_100150		1: 0.00020,
AZOLI_1846	1: 0.00162, 2: 0.00143, 3: 0.00256, 4: 0.00257,	1: 0.00169, 2: 0.00173, 3: 0.00190, 4: 0.00131,	AZOBR_100154	2: 0.00035, 4: 0.00023, 5: 0.00032,	1: 0.00017,
AZOLI_1846	1: 0.00248, 2: 0.00223, 3: 0.01865, 4: 0.00357,	1: 0.00756, 2: 0.00620, 4: 0.00072,	AZOBR_100157	3: 0.00007, 5: 0.00009,	1: 0.00006, 3: 0.00005,
AZOLI_1851	1: 0.00173, 2: 0.00231, 3: 0.00270, 4: 0.00248,	1: 0.00161, 2: 0.00170, 3: 0.00192, 4: 0.00204,	AZOBR_100162		
AZOLI_1852	1: 0.00197, 2: 0.00050, 4: 0.00119,	1: 0.00050, 2: 0.00086, 3: 0.00106, 4: 0.00076,	AZOBR_100163	1: 0.00019,	2: 0.00023,
AZOLI_1853	4: 0.00012,		AZOBR_100164		3: 0.00005,
AZOLI_1856	1: 0.00591, 2: 0.00317, 3: 0.00177, 4: 0.00336,	1: 0.00255, 2: 0.00319, 3: 0.00216, 4: 0.00478,	AZOBR_100169		
AZOLI_1857	1: 0.00052, 2: 0.00055,	1: 0.00050, 2: 0.00043,	AZOBR_100170		3: 0.00010,

	3: 0.00050, 4: 0.00091,	3: 0.00068, 4: 0.00050,			
AZOLI_1858	1: 0.00089, 2: 0.00073, 3: 0.00095, 4: 0.00058,	1: 0.00087, 2: 0.00092, 3: 0.00069, 4: 0.00067,	AZOBR_100174		
AZOLI_1860	1: 0.00104, 2: 0.00171, 3: 0.00062, 4: 0.00170,	1: 0.00094, 2: 0.00117, 3: 0.00164, 4: 0.00090,	AZOBR_100175	4: 0.00006,	1: 0.00006, 2: 0.00004, 3: 0.00009,
AZOLI_1861	1: 0.00412, 2: 0.00278, 3: 0.00474, 4: 0.00386,	1: 0.00213, 2: 0.00203, 3: 0.00244, 4: 0.00293,	AZOBR_100176	1: 0.00333, 2: 0.00609, 3: 0.00618, 4: 0.00434, 5: 0.00230,	1: 0.00343, 2: 0.00243, 3: 0.00296,
AZOLI_1863		2: 0.00006, 4: 0.00006,	AZOBR_100177	1: 0.00220, 2: 0.00175, 3: 0.00187, 4: 0.00356, 5: 0.00364,	1: 0.00373, 2: 0.00136, 3: 0.00308,
AZOLI_1864	4: 0.00004,		AZOBR_100178	1: 0.00411, 2: 0.00363, 3: 0.00328, 4: 0.00289, 5: 0.00304,	1: 0.00214, 2: 0.00302, 3: 0.00216,
AZOLI_1865	1: 0.00131, 2: 0.00173, 3: 0.00327, 4: 0.00253,	1: 0.00215, 2: 0.00207, 3: 0.00173, 4: 0.00297,	AZOBR_100179	1: 0.00840, 2: 0.00916, 3: 0.00856, 4: 0.00750, 5: 0.00715,	1: 0.00531, 2: 0.00619, 3: 0.00445,
AZOLI_1867	1: 0.00070, 2: 0.00102, 3: 0.00048, 4: 0.00075,	1: 0.00054, 2: 0.00082, 3: 0.00072, 4: 0.00065,	AZOBR_100187	1: 0.00039, 2: 0.00072, 3: 0.00091, 4: 0.00050, 5: 0.00044,	1: 0.00055, 2: 0.00037, 3: 0.00029,
AZOLI_1872	1: 0.00028, 2: 0.00060, 3: 0.00065, 4: 0.00079,	1: 0.00098, 2: 0.00092, 3: 0.00113, 4: 0.00083,	AZOBR_100190	3: 0.00011, 4: 0.00013,	2: 0.00009,
AZOLI_1873	1: 0.00131, 2: 0.00264, 3: 0.00117, 4: 0.00051,	1: 0.00048, 2: 0.00093, 3: 0.00064, 4: 0.00072,	AZOBR_100191	1: 0.00031, 2: 0.00022, 3: 0.00021, 4: 0.00017,	1: 0.00008,
AZOLI_1876	1: 0.00085, 2: 0.00049, 4: 0.00156,	1: 0.00037, 2: 0.00030, 3: 0.00039, 4: 0.00068,	AZOBR_100192		1: 0.00021,
AZOLI_1878	1: 0.00025, 2: 0.00011, 3: 0.00010, 4: 0.00028,	1: 0.00011, 2: 0.00011, 3: 0.00019, 4: 0.00012,	AZOBR_100193		
AZOLI_1881	2: 0.00021, 4: 0.00024,	1: 0.00021, 2: 0.00020, 3: 0.00052, 4: 0.00036,	AZOBR_100195	2: 0.00013, 3: 0.00008, 4: 0.00010, 5: 0.00007,	2: 0.00007, 3: 0.00015,
AZOLI_1883	1: 0.00020, 4: 0.00008,	3: 0.00011, 4: 0.00008,	AZOBR_100197	3: 0.00037, 4: 0.00030,	
AZOLI_1884	1: 0.00012, 4: 0.00027,	3: 0.00013,	AZOBR_100198	4: 0.00004, 5: 0.00004,	
AZOLI_1885	1: 0.00051, 2: 0.00051, 4: 0.00233,	1: 0.00103, 3: 0.00181, 4: 0.00088,	AZOBR_100201	1: 0.00156, 2: 0.00233, 3: 0.00245, 4: 0.00207, 5: 0.00204,	1: 0.00220, 2: 0.00297, 3: 0.00201,
AZOLI_1886	2: 0.00014,	2: 0.00014, 3: 0.00015, 4: 0.00008,	AZOBR_100202		3: 0.00013,
AZOLI_1890		1: 0.00136, 2: 0.00287, 3: 0.00220, 4: 0.00277,	AZOBR_100206		
AZOLI_1894	1: 0.00012, 2: 0.00015, 3: 0.00102, 4: 0.00027,	1: 0.00029, 2: 0.00031, 3: 0.00025, 4: 0.00020,	AZOBR_100207		
AZOLI_1895	1: 0.00005, 2: 0.00010, 4: 0.00010,	1: 0.00026, 2: 0.00015, 3: 0.00011, 4: 0.00010,	AZOBR_100208		3: 0.00003,
AZOLI_1901	2: 0.00031, 4: 0.00039,	2: 0.00015, 3: 0.00024, 4: 0.00026,	AZOBR_100209		
AZOLI_1902	1: 0.00072, 2: 0.00080, 3: 0.00077, 4: 0.00066,	1: 0.00088, 2: 0.00082, 3: 0.00106, 4: 0.00064,	AZOBR_100210		
AZOLI_1902	1: 0.00031, 4: 0.00026,	4: 0.00018,	AZOBR_100214		
AZOLI_1904	1: 0.00022, 2: 0.00029, 3: 0.00021, 4: 0.00023,	1: 0.00015, 2: 0.00012, 3: 0.00013, 4: 0.00013,	AZOBR_100215		2: 0.00008, 3: 0.00007,
AZOLI_1909	3: 0.00006,	1: 0.00006, 2: 0.00006,	AZOBR_100216	1: 0.00010, 4: 0.00004,	
AZOLI_1912	1: 0.00051, 2: 0.00047, 4: 0.00032,	1: 0.00052, 2: 0.00032, 3: 0.00030, 4: 0.00024,	AZOBR_100217	5: 0.00007,	1: 0.00010, 3: 0.00020,
AZOLI_1913	4: 0.00008,	4: 0.00005,	AZOBR_100219		
AZOLI_1914	1: 0.00383, 2: 0.00368, 3: 0.00263, 4: 0.00274,	1: 0.00540, 2: 0.00520, 3: 0.00472, 4: 0.00477,	AZOBR_100221		
AZOLI_1915	4: 0.00010,	2: 0.00008, 4: 0.00004,	AZOBR_100224		
AZOLI_1917	1: 0.00015, 3: 0.00022, 4: 0.00013,	1: 0.00012, 3: 0.00012, 4: 0.00007,	AZOBR_100225		
AZOLI_1931	4: 0.00004,	3: 0.00008,	AZOBR_100228	1: 0.00108, 2: 0.00104, 3: 0.00103, 4: 0.00085, 5: 0.00100,	1: 0.00077, 2: 0.00068, 3: 0.00071,
AZOLI_1941	2: 0.00024,		AZOBR_100230	1: 0.00117, 2: 0.00183, 3: 0.00077, 4: 0.00116, 5: 0.00054,	1: 0.00257, 2: 0.00160, 3: 0.00346,
AZOLI_1946	2: 0.00012,		AZOBR_100233		
AZOLI_1947	1: 0.00049, 2: 0.00042, 4: 0.00051,	1: 0.00042, 2: 0.00026, 3: 0.00018, 4: 0.00028,	AZOBR_100234	3: 0.00005, 4: 0.00004,	3: 0.00005,
AZOLI_1948	2: 0.00032,		AZOBR_100235	1: 0.00216, 2: 0.00372, 3: 0.00284, 4: 0.00299, 5: 0.00433,	1: 0.00325, 2: 0.00236, 3: 0.00305,
AZOLI_1949	1: 0.00011, 2: 0.00007,		AZOBR_100236	1: 0.00131, 2: 0.00237, 3: 0.00170, 4: 0.00156, 5: 0.00180,	1: 0.00084, 2: 0.00095, 3: 0.00103,
AZOLI_1951	1: 0.00005, 2: 0.00008, 4: 0.00014,	2: 0.00015, 3: 0.00006, 4: 0.00021,	AZOBR_100237	1: 0.00234, 2: 0.00128, 3: 0.00231, 4: 0.00142, 5: 0.00211,	1: 0.00057, 2: 0.00083, 3: 0.00077,
AZOLI_1955	3: 0.00009, 4: 0.00008,		AZOBR_100239	1: 0.00020, 2: 0.00029, 3: 0.00029, 4: 0.00046, 5: 0.00035,	1: 0.00038, 2: 0.00024, 3: 0.00052,
AZOLI_1956	1: 0.00086, 2: 0.00209, 3: 0.00170, 4: 0.00233,	1: 0.00082, 2: 0.00067, 3: 0.00111, 4: 0.00074,	AZOBR_100241	4: 0.00022,	2: 0.00023, 3: 0.00060,
AZOLI_1957	1: 0.00015, 2: 0.00033, 4: 0.00030,	1: 0.00013, 2: 0.00042, 3: 0.00022, 4: 0.00026,	AZOBR_100244		
AZOLI_1960	1: 0.00194, 2: 0.00254, 3: 0.00507, 4: 0.00227,	1: 0.00110, 2: 0.00162, 3: 0.00221, 4: 0.00242,	AZOBR_100245	1: 0.00021, 2: 0.00025, 3: 0.00018, 5: 0.00029,	1: 0.00022, 2: 0.00010, 3: 0.00026,
AZOLI_1961	1: 0.00012, 2: 0.00042,	1: 0.00012, 2: 0.00012, 3: 0.00013, 4: 0.00021,	AZOBR_100246	3: 0.00006, 4: 0.00015, 5: 0.00005,	1: 0.00005, 2: 0.00008,
AZOLI_1962	1: 0.00133, 2: 0.00165, 3: 0.00065, 4: 0.00123,	1: 0.00100, 2: 0.00094, 3: 0.00086, 4: 0.00075,	AZOBR_100247	1: 0.00106, 2: 0.00056, 3: 0.00060, 4: 0.00205, 5: 0.00161,	1: 0.00073, 2: 0.00083, 3: 0.00097,
AZOLI_1969		1: 0.00026, 3: 0.00037, 4: 0.00030,	AZOBR_100248	1: 0.00024, 2: 0.00042, 3: 0.00032, 4: 0.00014, 5: 0.00014,	1: 0.00004, 2: 0.00022, 3: 0.00008,
AZOLI_1973	1: 0.00010, 4: 0.00009,	4: 0.00018,	AZOBR_100249	1: 0.00038, 2: 0.00028, 3: 0.00041, 4: 0.00033, 5: 0.00033,	1: 0.00028, 2: 0.00029, 3: 0.00035,
AZOLI_1975	2: 0.00035, 4: 0.00018,	1: 0.00014, 2: 0.00027,	AZOBR_100251	1: 0.00161, 2: 0.00135, 3: 0.00137, 4: 0.00099, 5: 0.00074,	1: 0.00144, 2: 0.00170, 3: 0.00061,
AZOLI_1976	2: 0.00012, 4: 0.00021,		AZOBR_100259	1: 0.00005, 4: 0.00009, 5: 0.00009,	3: 0.00003,
AZOLI_1978	1: 0.00010, 4: 0.00017,	2: 0.00019, 4: 0.00013,	AZOBR_100260	1: 0.00054, 2: 0.00077, 3: 0.00092, 4: 0.00052, 5: 0.00067,	1: 0.00062, 2: 0.00079, 3: 0.00121,
AZOLI_1979	1: 0.00253, 2: 0.00085,		AZOBR_100263		1: 0.00013, 3: 0.00009,
AZOLI_1980	2: 0.00012, 4: 0.00016,	1: 0.00012, 3: 0.00016, 4: 0.00008,	AZOBR_100264		1: 0.00035, 3: 0.00027,

AZOLI_1996	2: 0.00027,		AZOBR_100265	2: 0.00012, 3: 0.00008, 4: 0.00016, 5: 0.00016,	1: 0.00013, 2: 0.00010, 3: 0.00019,
AZOLI_2071		4: 0.00005,	AZOBR_100267	1: 0.00019, 3: 0.00011,	2: 0.00009,
AZOLI_2101	4: 0.00011,		AZOBR_100268	2: 0.00009,	1: 0.00020, 2: 0.00022, 3: 0.00041,
AZOLI_2110	1: 0.00002, 4: 0.00002,	1: 0.00009, 2: 0.00009, 3: 0.00007, 4: 0.00006,	AZOBR_100274	1: 0.00300, 2: 0.00378, 3: 0.00237, 4: 0.00299, 5: 0.00326,	1: 0.00356, 2: 0.00328, 3: 0.00304,
AZOLI_2113	1: 0.00013, 2: 0.00018, 3: 0.00031,	2: 0.00017, 4: 0.00030,	AZOBR_100275	1: 0.00510, 2: 0.00368, 3: 0.00434, 4: 0.00351, 5: 0.00405,	1: 0.00237, 2: 0.00394, 3: 0.00365,
AZOLI_2115	1: 0.00013, 2: 0.00013, 4: 0.00026,	1: 0.00011, 2: 0.00009, 3: 0.00016, 4: 0.00006,	AZOBR_100276	1: 0.00906, 2: 0.00808, 3: 0.00679, 4: 0.00676, 5: 0.00550,	1: 0.00677, 2: 0.00654, 3: 0.00407,
AZOLI_2121	2: 0.00023, 4: 0.00026,	2: 0.00015, 4: 0.00020,	AZOBR_100277	1: 0.00058, 2: 0.00031, 3: 0.00064, 4: 0.00024, 5: 0.00024,	2: 0.00034, 3: 0.00022,
AZOLI_2122	1: 0.00007, 2: 0.00007, 4: 0.00021,	2: 0.00010, 4: 0.00021,	AZOBR_100278	1: 0.00212, 2: 0.00243, 3: 0.00267, 4: 0.00357, 5: 0.00325,	1: 0.00151, 2: 0.00160, 3: 0.00200,
AZOLI_2125		2: 0.00019, 3: 0.00021, 4: 0.00017,	AZOBR_100281		
AZOLI_2125	1: 0.00009, 2: 0.00013,	1: 0.00016, 2: 0.00021, 3: 0.00017, 4: 0.00027,	AZOBR_100283	2: 0.00003, 4: 0.00009, 5: 0.00008,	3: 0.00006,
AZOLI_2126	2: 0.00009,	1: 0.00021, 2: 0.00018, 3: 0.00019, 4: 0.00016,	AZOBR_100284	1: 0.00265, 2: 0.00234, 3: 0.00251, 4: 0.00266, 5: 0.00214,	1: 0.00247, 2: 0.00176, 3: 0.00249,
AZOLI_2130	1: 0.00016, 4: 0.00014,	2: 0.00011, 3: 0.00006, 4: 0.00012,	AZOBR_100285		
AZOLI_2132	1: 0.00007, 2: 0.00007, 4: 0.00004,	1: 0.00007, 2: 0.00009, 3: 0.00008, 4: 0.00012,	AZOBR_100286	1: 0.00050, 2: 0.00078, 3: 0.00075, 4: 0.00080, 5: 0.00050,	1: 0.00025, 2: 0.00028, 3: 0.00045,
AZOLI_2133	4: 0.00006,		AZOBR_100287	3: 0.00013, 4: 0.00007,	1: 0.00010,
AZOLI_2134	2: 0.00007, 4: 0.00012,	1: 0.00032, 2: 0.00021, 3: 0.00030, 4: 0.00018,	AZOBR_100292		
AZOLI_2136	4: 0.00005,		AZOBR_100294	1: 0.00092, 2: 0.00064, 3: 0.00050, 4: 0.00053, 5: 0.00047,	1: 0.00019, 2: 0.00064, 3: 0.00027,
AZOLI_2137	1: 0.00028, 2: 0.00037, 3: 0.00022, 4: 0.00023,	1: 0.00023, 2: 0.00039, 3: 0.00024, 4: 0.00030,	AZOBR_100296	1: 0.00065, 2: 0.00044, 3: 0.00066, 4: 0.00064, 5: 0.00053,	1: 0.00055, 2: 0.00038, 3: 0.00048,
AZOLI_2142	3: 0.00046,	2: 0.00040, 3: 0.00052, 4: 0.00042,	AZOBR_100299	2: 0.00022, 3: 0.00021, 4: 0.00009, 5: 0.00022,	1: 0.00012, 2: 0.00009, 3: 0.00023,
AZOLI_2143	2: 0.00004, 3: 0.00008, 4: 0.00007,	1: 0.00006, 2: 0.00010, 3: 0.00012, 4: 0.00015,	AZOBR_100300		
AZOLI_2145	1: 0.00386, 2: 0.00241, 3: 0.00204, 4: 0.00299,	1: 0.00135, 2: 0.00164, 3: 0.00131, 4: 0.00141,	AZOBR_100301	2: 0.00031, 3: 0.00038,	1: 0.00023, 2: 0.00026, 3: 0.00017,
AZOLI_2146	1: 0.00024, 2: 0.00029, 4: 0.00016,	1: 0.00029, 2: 0.00023, 3: 0.00026,	AZOBR_100302	2: 0.00025, 3: 0.00032, 4: 0.00013,	2: 0.00014, 3: 0.00018,
AZOLI_2150		4: 0.00006,	AZOBR_100303	3: 0.00024, 4: 0.00008, 5: 0.00011,	1: 0.00014, 2: 0.00012, 3: 0.00014,
AZOLI_2151	1: 0.00018, 2: 0.00013, 4: 0.00023,	1: 0.00019, 2: 0.00010, 3: 0.00017, 4: 0.00007,	AZOBR_100304	1: 0.00016, 3: 0.00010, 4: 0.00020, 5: 0.00014,	2: 0.00018, 3: 0.00018,
AZOLI_2152	1: 0.00066, 2: 0.00030, 4: 0.00076,	1: 0.00022, 4: 0.00025,	AZOBR_100305	4: 0.00010,	3: 0.00018,
AZOLI_2154		2: 0.00031, 4: 0.00036,	AZOBR_100311	2: 0.00015, 3: 0.00007, 4: 0.00010, 5: 0.00006,	1: 0.00011, 2: 0.00004,
AZOLI_2156		1: 0.00013, 2: 0.00012, 3: 0.00009, 4: 0.00010,	AZOBR_100312	3: 0.00004, 4: 0.00013, 5: 0.00011,	1: 0.00016, 2: 0.00019, 3: 0.00030,
AZOLI_2160		4: 0.00018,	AZOBR_100314	1: 0.00122, 2: 0.00100, 3: 0.00089, 4: 0.00102, 5: 0.00145,	1: 0.00067, 2: 0.00076, 3: 0.00092,
AZOLI_2165	1: 0.00155, 2: 0.00239, 3: 0.00076, 4: 0.00185,	1: 0.00263, 2: 0.00324, 3: 0.00244, 4: 0.00227,	AZOBR_100316	1: 0.00008, 2: 0.00012, 3: 0.00011, 4: 0.00009, 5: 0.00007,	1: 0.00015, 2: 0.00010, 3: 0.00018,
AZOLI_2169	1: 0.00014, 2: 0.00026, 3: 0.00041, 4: 0.00044,	1: 0.00022, 2: 0.00025, 3: 0.00019, 4: 0.00016,	AZOBR_100317	1: 0.00069, 2: 0.00044, 3: 0.00043, 4: 0.00026, 5: 0.00035,	2: 0.00014, 3: 0.00008,
AZOLI_2172	1: 0.00030, 2: 0.00025, 4: 0.00009,	1: 0.00015, 2: 0.00015, 4: 0.00013,	AZOBR_100318	1: 0.00021, 3: 0.00018, 4: 0.00011, 5: 0.00007,	1: 0.00017, 2: 0.00015, 3: 0.00016,
AZOLI_2173	1: 0.00032, 2: 0.00055, 3: 0.00018, 4: 0.00041,	1: 0.00019, 2: 0.00025, 3: 0.00027, 4: 0.00022,	AZOBR_100319		
AZOLI_2175	1: 0.00018,		AZOBR_100320		
AZOLI_2176	1: 0.00091, 2: 0.00188, 3: 0.00022, 4: 0.00137,	1: 0.00044, 2: 0.00027, 3: 0.00043, 4: 0.00017,	AZOBR_100322		
AZOLI_2177	1: 0.00060, 2: 0.00042, 3: 0.00065, 4: 0.00012,	1: 0.00047, 2: 0.00049, 3: 0.00035, 4: 0.00028,	AZOBR_100323		
AZOLI_2180	1: 0.00026, 2: 0.00031, 3: 0.00015, 4: 0.00040,	1: 0.00021, 2: 0.00038, 3: 0.00019, 4: 0.00040,	AZOBR_100325	1: 0.00304, 2: 0.00407, 3: 0.00317, 4: 0.00216, 5: 0.00351,	1: 0.00414, 2: 0.00353, 3: 0.00321,
AZOLI_2183	1: 0.00044,	3: 0.00047,	AZOBR_100326	1: 0.00092, 2: 0.00133, 3: 0.00128, 4: 0.00100, 5: 0.00087,	1: 0.00078, 2: 0.00113, 3: 0.00097,
AZOLI_2188	1: 0.00059, 2: 0.00077, 3: 0.00059, 4: 0.00015,	1: 0.00103, 2: 0.00066, 3: 0.00118, 4: 0.00044,	AZOBR_100329	1: 0.00020, 2: 0.00010, 3: 0.00013, 4: 0.00020, 5: 0.00019,	1: 0.00025, 2: 0.00018, 3: 0.00024,
AZOLI_2190	1: 0.00006, 2: 0.00004, 4: 0.00005,		AZOBR_100333	4: 0.00017,	1: 0.00037, 2: 0.00042, 3: 0.00092,
AZOLI_2191		3: 0.00014,	AZOBR_100334		
AZOLI_2198	1: 0.00049, 2: 0.00028, 3: 0.00039, 4: 0.00078,	1: 0.00064, 2: 0.00102, 3: 0.00067, 4: 0.00060,	AZOBR_100337		1: 0.00017, 3: 0.00017,
AZOLI_2200	1: 0.00010, 2: 0.00007, 3: 0.00026, 4: 0.00014,	1: 0.00030, 2: 0.00029, 3: 0.00037, 4: 0.00043,	AZOBR_100340	1: 0.00025, 2: 0.00022, 3: 0.00052, 4: 0.00040, 5: 0.00019,	1: 0.00014, 2: 0.00029, 3: 0.00013,
AZOLI_2201	1: 0.00024, 2: 0.00016, 3: 0.00015, 4: 0.00009,	1: 0.00005, 2: 0.00005, 3: 0.00006, 4: 0.00009,	AZOBR_100341	1: 0.00017, 4: 0.00012, 5: 0.00015,	2: 0.00008, 3: 0.00014,
AZOLI_2202	1: 0.00063, 2: 0.00101, 3: 0.00030, 4: 0.00100,	1: 0.00080, 2: 0.00036, 3: 0.00056, 4: 0.00041,	AZOBR_100344	4: 0.00021, 5: 0.00014,	1: 0.00050, 2: 0.00057, 3: 0.00068,
AZOLI_2203	1: 0.00008, 3: 0.00015,	1: 0.00008, 3: 0.00009, 4: 0.00005,	AZOBR_100347		
AZOLI_2204	2: 0.00038, 3: 0.00099, 4: 0.00019,	1: 0.00071, 2: 0.00063, 3: 0.00035, 4: 0.00098,	AZOBR_100348		
AZOLI_2208	1: 0.00045, 2: 0.00030, 4: 0.00052,	1: 0.00076, 2: 0.00059, 3: 0.00081, 4: 0.00091,	AZOBR_100353	1: 0.00026, 2: 0.00015, 3: 0.00033, 4: 0.00029, 5: 0.00027,	1: 0.00077, 2: 0.00081, 3: 0.00085,
AZOLI_2212	2: 0.00015, 4: 0.00032,	1: 0.00060, 2: 0.00051, 3: 0.00128, 4: 0.00077,	AZOBR_100354	4: 0.00028, 5: 0.00028,	1: 0.00052, 2: 0.00089, 3: 0.00044,

AZOLI_2213	4: 0.00009,		AZOBR_100355		
AZOLI_2214	2: 0.00005, 3: 0.00032, 4: 0.00003,	1: 0.00043, 2: 0.00026, 3: 0.00034, 4: 0.00032,	AZOBR_100358		1: 0.00002,
AZOLI_2215	1: 0.00030,	3: 0.00016,	AZOBR_100361		
AZOLI_2219	2: 0.00004, 4: 0.00010,	4: 0.00003,	AZOBR_100363		
AZOLI_2228	1: 0.00046, 2: 0.00017, 3: 0.00046, 4: 0.00045,	1: 0.00023, 2: 0.00086, 3: 0.00091, 4: 0.00054,	AZOBR_100364	1: 0.00017,	
AZOLI_2232	1: 0.00350, 2: 0.00397, 3: 0.00334, 4: 0.00296,	1: 0.00098, 2: 0.00101, 3: 0.00082, 4: 0.00081,	AZOBR_100368		
AZOLI_2235	1: 0.00026,	2: 0.00025, 3: 0.00041, 4: 0.00033,	AZOBR_100373	1: 0.00081, 2: 0.00057, 3: 0.00052, 4: 0.00047, 5: 0.00082,	1: 0.00054, 2: 0.00054, 3: 0.00061,
AZOLI_2236	1: 0.00081, 2: 0.00082, 3: 0.00152, 4: 0.00075,	1: 0.00061, 2: 0.00138, 3: 0.00051, 4: 0.00099,	AZOBR_100374		3: 0.00010,
AZOLI_2237	1: 0.00021, 2: 0.00043, 4: 0.00045,	1: 0.00025, 2: 0.00011, 3: 0.00017, 4: 0.00020,	AZOBR_100375		3: 0.00010,
AZOLI_2238	2: 0.00011, 4: 0.00018,		AZOBR_100378	1: 0.00763, 2: 0.01249, 3: 0.00931, 4: 0.00850, 5: 0.00884,	1: 0.00652, 2: 0.00625, 3: 0.00451,
AZOLI_2239	2: 0.00010, 4: 0.00006,		AZOBR_100379	1: 0.00508, 2: 0.00585, 3: 0.00471, 4: 0.00362, 5: 0.00432,	1: 0.00337, 2: 0.00436, 3: 0.00231,
AZOLI_2240	2: 0.00035, 3: 0.00048, 4: 0.00033,	1: 0.00042, 2: 0.00048, 3: 0.00033, 4: 0.00036,	AZOBR_100380	1: 0.00025, 2: 0.00037, 3: 0.00039, 4: 0.00020, 5: 0.00011,	1: 0.00013, 2: 0.00006, 3: 0.00015,
AZOLI_2241		1: 0.00067, 2: 0.00032, 3: 0.00071, 4: 0.00065,	AZOBR_100382	1: 0.00432, 2: 0.00375, 3: 0.00330, 4: 0.00631, 5: 0.00632,	1: 0.00692, 2: 0.00602, 3: 0.00592,
AZOLI_2242	1: 0.00100, 2: 0.00066, 3: 0.00165, 4: 0.00105,	1: 0.00114, 2: 0.00178, 3: 0.00126, 4: 0.00124,	AZOBR_100383	1: 0.00007, 2: 0.00019, 3: 0.00010, 4: 0.00013, 5: 0.00008,	1: 0.00033, 2: 0.00026, 3: 0.00033,
AZOLI_2246	1: 0.00048, 2: 0.00048, 3: 0.00037, 4: 0.00018,		AZOBR_100388	1: 0.00038, 2: 0.00030, 3: 0.00044, 4: 0.00019, 5: 0.00017,	1: 0.00013, 2: 0.00027, 3: 0.00019,
AZOLI_2248	1: 0.00014, 4: 0.00024,		AZOBR_100389	1: 0.00117, 2: 0.00071, 3: 0.00085, 4: 0.00094, 5: 0.00087,	1: 0.00045, 2: 0.00063, 3: 0.00066,
AZOLI_2250	4: 0.00005,	1: 0.00006, 2: 0.00010, 3: 0.00009, 4: 0.00012,	AZOBR_100390	1: 0.00172, 2: 0.00159, 3: 0.00236, 4: 0.00294, 5: 0.00243,	1: 0.00132, 2: 0.00237, 3: 0.00126,
AZOLI_2251	2: 0.00034, 4: 0.00039,	1: 0.00034, 2: 0.00055, 3: 0.00048, 4: 0.00039,	AZOBR_100391		1: 0.00049, 2: 0.00032, 3: 0.00082,
AZOLI_2252	1: 0.00124, 2: 0.00080, 4: 0.00078,	1: 0.00103, 2: 0.00144, 3: 0.00146, 4: 0.00147,	AZOBR_100398	4: 0.00008,	
AZOLI_2257	1: 0.00018, 2: 0.00018,		AZOBR_100400		2: 0.00015,
AZOLI_2259	1: 0.00028, 2: 0.00086, 4: 0.00025,	2: 0.00021, 4: 0.00018,	AZOBR_100408	1: 0.00095, 2: 0.00094, 3: 0.00100, 4: 0.00139, 5: 0.00073,	1: 0.00132, 2: 0.00123, 3: 0.00082,
AZOLI_2260		2: 0.00005,	AZOBR_100410		1: 0.00016, 2: 0.00018, 3: 0.00017,
AZOLI_2261	4: 0.00021,	3: 0.00013, 4: 0.00021,	AZOBR_100411		3: 0.00015,
AZOLI_2264	1: 0.00313, 2: 0.00204, 3: 0.00185, 4: 0.00226,	1: 0.00123, 2: 0.00168, 3: 0.00173, 4: 0.00157,	AZOBR_110003	1: 0.00026, 2: 0.00031, 3: 0.00024, 4: 0.00036, 5: 0.00028,	3: 0.00004,
AZOLI_2269		3: 0.00008,	AZOBR_110005	1: 0.00028, 2: 0.00015, 3: 0.00038, 4: 0.00021, 5: 0.00014,	1: 0.00009, 2: 0.00018, 3: 0.00013,
AZOLI_2271	1: 0.00069, 2: 0.00086, 3: 0.00009, 4: 0.00073,	1: 0.00010, 2: 0.00012, 3: 0.00007, 4: 0.00005,	AZOBR_110006		3: 0.00006,
AZOLI_2272	2: 0.00018,		AZOBR_110010	3: 0.00012, 4: 0.00010,	2: 0.00015,
AZOLI_2275	1: 0.00411, 2: 0.00664, 3: 0.00898, 4: 0.00674,	1: 0.00897, 2: 0.00763, 3: 0.00684, 4: 0.00746,	AZOBR_110011	1: 0.00025, 2: 0.00045, 3: 0.00032, 4: 0.00061, 5: 0.00044,	1: 0.00012, 2: 0.00014, 3: 0.00024,
AZOLI_2284	1: 0.00018, 2: 0.00018, 3: 0.00020, 4: 0.00036,	1: 0.00024, 2: 0.00027, 3: 0.00026, 4: 0.00021,	AZOBR_110015		
AZOLI_2285	1: 0.00007, 2: 0.00010, 4: 0.00006,	1: 0.00010, 4: 0.00006,	AZOBR_110016		
AZOLI_2288	1: 0.00049, 2: 0.00018, 4: 0.00042,	1: 0.00009, 2: 0.00009, 4: 0.00008,	AZOBR_110019	1: 0.00064, 2: 0.00028, 3: 0.00095, 4: 0.00066, 5: 0.00039,	1: 0.00046, 2: 0.00017, 3: 0.00055,
AZOLI_2290	1: 0.00069, 2: 0.00084, 3: 0.00123, 4: 0.00044,	1: 0.00114, 2: 0.00096, 3: 0.00113, 4: 0.00113,	AZOBR_110020		3: 0.00071,
AZOLI_2296	1: 0.00009,		AZOBR_110023		
AZOLI_2300	1: 0.00199, 2: 0.00278, 3: 0.00323, 4: 0.00185,	1: 0.00248, 2: 0.00254, 3: 0.00197, 4: 0.00225,	AZOBR_110028		
AZOLI_2308		2: 0.00003, 4: 0.00003,	AZOBR_110029		
AZOLI_2309		4: 0.00007,	AZOBR_110031	1: 0.00116, 2: 0.00154, 3: 0.00111, 4: 0.00180, 5: 0.00120,	1: 0.00102, 2: 0.00127, 3: 0.00108,
AZOLI_2311		3: 0.00009,	AZOBR_110032	4: 0.00040,	
AZOLI_2313		1: 0.00013,	AZOBR_110033	4: 0.00004,	2: 0.00004, 3: 0.00003,
AZOLI_2314		1: 0.00017, 2: 0.00012, 3: 0.00018, 4: 0.00022,	AZOBR_110035	4: 0.00021, 5: 0.00010,	1: 0.00010, 2: 0.00011,
AZOLI_2316		2: 0.00033, 3: 0.00048, 4: 0.00048,	AZOBR_110037	1: 0.00041, 2: 0.00030, 3: 0.00052, 4: 0.00061, 5: 0.00033,	1: 0.00022, 2: 0.00020, 3: 0.00026,
AZOLI_2317		1: 0.00018, 2: 0.00017, 3: 0.00028, 4: 0.00010,	AZOBR_110041	1: 0.00028, 2: 0.00083, 3: 0.00085, 4: 0.00061, 5: 0.00042,	1: 0.00039, 2: 0.00085, 3: 0.00066,
AZOLI_2318		1: 0.00333, 2: 0.00224, 3: 0.00405, 4: 0.00368,	AZOBR_110043		3: 0.00027,
AZOLI_2319		2: 0.00047,	AZOBR_110044		
AZOLI_2320		1: 0.00008, 2: 0.00008, 3: 0.00021, 4: 0.00010,	AZOBR_110045	3: 0.00043, 4: 0.00026, 5: 0.00017,	3: 0.00016,
AZOLI_2321		1: 0.00035, 2: 0.00030, 3: 0.00033, 4: 0.00030,	AZOBR_110052	5: 0.00026,	1: 0.00024, 2: 0.00034, 3: 0.00041,
AZOLI_2326		1: 0.00035, 2: 0.00042, 3: 0.00028, 4: 0.00074,	AZOBR_110057	4: 0.00010,	
AZOLI_2333	2: 0.00017, 4: 0.00067,	1: 0.00026, 2: 0.00059, 3: 0.00065, 4: 0.00060,	AZOBR_110059		1: 0.00016, 3: 0.00010,
AZOLI_2341	2: 0.00015, 4: 0.00046,	1: 0.00038, 2: 0.00082, 4: 0.00066,	AZOBR_110060	1: 0.00017, 3: 0.00009, 4: 0.00007, 5: 0.00005,	1: 0.00004, 2: 0.00007, 3: 0.00004,
AZOLI_2344	1: 0.00013, 2: 0.00018, 3: 0.00025, 4: 0.00028,	1: 0.00020, 2: 0.00017, 3: 0.00021, 4: 0.00031,	AZOBR_110063	2: 0.00062, 3: 0.00045, 4: 0.00060, 5: 0.00060,	1: 0.00022, 2: 0.00038,
AZOLI_2345	1: 0.00084, 2: 0.00093, 3: 0.00024, 4: 0.00064,	1: 0.00050, 2: 0.00063, 3: 0.00061, 4: 0.00043,	AZOBR_110065		
AZOLI_2348	1: 0.00133, 2: 0.00135,	1: 0.00135, 2: 0.00116,	AZOBR_110066		2: 0.00013, 3: 0.00011,

	3: 0.00132, 4: 0.00128,	3: 0.00143, 4: 0.00107,			
AZOLI_2349	1: 0.00012, 2: 0.00017, 3: 0.00009, 4: 0.00022,	1: 0.00023, 2: 0.00019, 3: 0.00028, 4: 0.00025,	AZOBR_110073	1: 0.00066, 2: 0.00078, 3: 0.00047, 4: 0.00098, 5: 0.00034,	1: 0.00018, 2: 0.00024, 3: 0.00024,
AZOLI_2353	1: 0.00019,	1: 0.00048, 2: 0.00019, 3: 0.00031, 4: 0.00025,	AZOBR_110076		
AZOLI_2357	1: 0.00012, 2: 0.00017, 3: 0.00010, 4: 0.00017,	1: 0.00020, 3: 0.00008, 4: 0.00006,	AZOBR_110077		1: 0.00024, 3: 0.00023,
AZOLI_2358	1: 0.00025, 2: 0.00018, 4: 0.00031,	1: 0.00018, 2: 0.00022, 3: 0.00022, 4: 0.00022,	AZOBR_110079	1: 0.00416, 2: 0.00396, 3: 0.00449, 4: 0.00415, 5: 0.00326,	1: 0.01626, 2: 0.02285, 3: 0.01188,
AZOLI_2360	1: 0.01263, 2: 0.01093, 3: 0.01025, 4: 0.00729,	1: 0.01288, 2: 0.01438, 3: 0.01158, 4: 0.01341,	AZOBR_110082	2: 0.00054, 3: 0.00087, 4: 0.00042, 5: 0.00169,	1: 0.00052, 2: 0.00045, 3: 0.00025,
AZOLI_2361	1: 0.00127, 2: 0.00067, 3: 0.00022, 4: 0.00092,	1: 0.00057, 2: 0.00060, 3: 0.00071, 4: 0.00075,	AZOBR_110083	4: 0.00006,	3: 0.00023,
AZOLI_2364		1: 0.00004,	AZOBR_110084		
AZOLI_2366	1: 0.00007, 4: 0.00015,	1: 0.00017, 2: 0.00010, 4: 0.00006,	AZOBR_110088	1: 0.00019, 2: 0.00011, 3: 0.00027, 4: 0.00022, 5: 0.00013,	
AZOLI_2367	3: 0.00023,		AZOBR_110089	1: 0.00043, 2: 0.00033, 3: 0.00042, 4: 0.00025,	1: 0.00016, 3: 0.00008,
AZOLI_2371	4: 0.00003,	3: 0.00004,	AZOBR_110090	2: 0.00016, 3: 0.00021, 4: 0.00010,	3: 0.00006,
AZOLI_2372	2: 0.00010, 4: 0.00013,	1: 0.00141, 2: 0.00095, 3: 0.00099, 4: 0.00071,	AZOBR_110092		
AZOLI_2380	1: 0.00056, 2: 0.00107, 3: 0.00070, 4: 0.00054,	1: 0.00095, 2: 0.00085, 3: 0.00060, 4: 0.00043,	AZOBR_110093	1: 0.00032, 2: 0.00028, 3: 0.00045, 4: 0.00025, 5: 0.00033,	1: 0.00007, 2: 0.00012, 3: 0.00013,
AZOLI_2381	1: 0.00025, 2: 0.00018, 3: 0.00015, 4: 0.00040,	1: 0.00014, 2: 0.00017, 3: 0.00023, 4: 0.00040,	AZOBR_110094	3: 0.00013, 4: 0.00026, 5: 0.00010,	
AZOLI_2383	1: 0.00193, 2: 0.00255, 4: 0.00090,	1: 0.00151, 2: 0.00087, 3: 0.00096, 4: 0.00103,	AZOBR_110095	1: 0.00040, 2: 0.00053, 3: 0.00052, 4: 0.00042, 5: 0.00056,	1: 0.00116, 2: 0.00103, 3: 0.00125,
AZOLI_2384	1: 0.00071, 2: 0.00090, 4: 0.00092,		AZOBR_110096	1: 0.00016, 2: 0.00019, 3: 0.00019, 4: 0.00007,	1: 0.00010, 2: 0.00012, 3: 0.00007,
AZOLI_2387	1: 0.00330, 2: 0.00304, 3: 0.00611, 4: 0.00224,	1: 0.00736, 2: 0.00911, 3: 0.00896, 4: 0.00986,	AZOBR_110098		
AZOLI_2388	1: 0.00480, 2: 0.00438, 3: 0.01002, 4: 0.00499,	1: 0.00910, 2: 0.00833, 3: 0.01065, 4: 0.00920,	AZOBR_110099	1: 0.00017, 3: 0.00015, 4: 0.00018, 5: 0.00006,	
AZOLI_2391	1: 0.00102, 2: 0.00189, 3: 0.00370, 4: 0.00217,	1: 0.00201, 2: 0.00241, 3: 0.00236, 4: 0.00250,	AZOBR_110102	1: 0.00255, 2: 0.00327, 3: 0.00217, 4: 0.00234, 5: 0.00234,	1: 0.00396, 2: 0.00287, 3: 0.00317,
AZOLI_2392	1: 0.00094, 2: 0.00206, 4: 0.00054,	1: 0.00143, 2: 0.00215, 3: 0.00152, 4: 0.00068,	AZOBR_110104	1: 0.00500, 2: 0.00598, 3: 0.00591, 4: 0.00489, 5: 0.00857,	1: 0.00341, 2: 0.00282, 3: 0.00371,
AZOLI_2393	1: 0.00011,	1: 0.00009,	AZOBR_110105	1: 0.00380, 2: 0.00495, 3: 0.00281, 4: 0.00248, 5: 0.00449,	1: 0.00282, 2: 0.00299, 3: 0.00268,
AZOLI_2395	4: 0.00006,	1: 0.00013, 2: 0.00035, 3: 0.00024, 4: 0.00014,	AZOBR_110106	1: 0.00009, 2: 0.00040, 3: 0.00043, 4: 0.00050, 5: 0.00031,	1: 0.00023, 2: 0.00037, 3: 0.00034,
AZOLI_2402	1: 0.01223, 2: 0.01157, 3: 0.00969, 4: 0.00606,	1: 0.00372, 2: 0.00358, 3: 0.00377, 4: 0.00423,	AZOBR_110107	1: 0.00047, 2: 0.00058, 3: 0.00030, 4: 0.00041, 5: 0.00074,	1: 0.00091, 2: 0.00078, 3: 0.00133,
AZOLI_2404		2: 0.00014,	AZOBR_110108	3: 0.00094, 4: 0.00038, 5: 0.00063,	1: 0.00306, 2: 0.00214, 3: 0.00217,
AZOLI_2408	1: 0.00166, 2: 0.00246, 3: 0.00125, 4: 0.00239,	1: 0.00157, 2: 0.00184, 3: 0.00083, 4: 0.00086,	AZOBR_110109	1: 0.00093, 2: 0.00156, 3: 0.00088, 4: 0.00109, 5: 0.00100,	1: 0.00080, 2: 0.00096, 3: 0.00073,
AZOLI_2409	1: 0.00321, 2: 0.00300, 3: 0.00621, 4: 0.00216,	1: 0.00362, 2: 0.00349, 3: 0.00435, 4: 0.00206,	AZOBR_110110		2: 0.00006,
AZOLI_2411	1: 0.00050, 2: 0.00035, 4: 0.00041,	1: 0.00021, 2: 0.00018, 3: 0.00011, 4: 0.00018,	AZOBR_110113	4: 0.00008, 5: 0.00008,	1: 0.00011, 2: 0.00010, 3: 0.00015,
AZOLI_2412	1: 0.00009, 2: 0.00018, 4: 0.00026,	4: 0.00008,	AZOBR_110114	1: 0.00130, 2: 0.00185, 3: 0.00127, 4: 0.00089, 5: 0.00134,	1: 0.00105, 2: 0.00109, 3: 0.00115,
AZOLI_2415	1: 0.00036, 2: 0.00056, 3: 0.00027, 4: 0.00075,	1: 0.00054, 2: 0.00078, 3: 0.00057, 4: 0.00050,	AZOBR_110115		
AZOLI_2416		3: 0.00005,	AZOBR_110117	1: 0.00018, 3: 0.00047, 4: 0.00057, 5: 0.00025,	2: 0.00013,
AZOLI_2417	1: 0.00033, 2: 0.00046, 3: 0.00049, 4: 0.00075,	1: 0.00033, 2: 0.00038, 3: 0.00054, 4: 0.00058,	AZOBR_110119		
AZOLI_2419	4: 0.00020,	2: 0.00030, 3: 0.00025,	AZOBR_110120	1: 0.00009, 2: 0.00011, 3: 0.00022, 4: 0.00024, 5: 0.00024,	1: 0.00006, 2: 0.00013,
AZOLI_2421	1: 0.00051, 2: 0.00032, 3: 0.00011, 4: 0.00090,	2: 0.00010, 3: 0.00009, 4: 0.00021,	AZOBR_110123	1: 0.00008, 4: 0.00008,	1: 0.00008, 2: 0.00012,
AZOLI_2423	1: 0.00056, 2: 0.00042, 3: 0.00029, 4: 0.00030,	1: 0.00028, 2: 0.00041, 3: 0.00022, 4: 0.00018,	AZOBR_110125	2: 0.00049, 3: 0.00016, 4: 0.00070, 5: 0.00083,	1: 0.00012, 3: 0.00115,
AZOLI_2425	1: 0.00037, 2: 0.00057, 4: 0.00016,	1: 0.00019, 2: 0.00055, 4: 0.00048,	AZOBR_110127		
AZOLI_2426	1: 0.00004, 2: 0.00007, 3: 0.00006, 4: 0.00003,	1: 0.00009, 3: 0.00017, 4: 0.00014,	AZOBR_110128	1: 0.00007, 2: 0.00006, 3: 0.00009,	1: 0.00014, 2: 0.00008, 3: 0.00011,
AZOLI_2427	4: 0.00016,	1: 0.00025, 2: 0.00037, 3: 0.00020, 4: 0.00043,	AZOBR_120001	1: 0.00035, 2: 0.00031, 3: 0.00045,	
AZOLI_2431		1: 0.00016, 2: 0.00019, 3: 0.00010, 4: 0.00017,	AZOBR_120007		1: 0.00007,
AZOLI_2433	2: 0.00034, 4: 0.00039,	1: 0.00045, 2: 0.00022, 3: 0.00048, 4: 0.00087,	AZOBR_120009		
AZOLI_2434		2: 0.00009,	AZOBR_120010		1: 0.00017, 2: 0.00014, 3: 0.00033,
AZOLI_2437	1: 0.00787, 2: 0.00884, 3: 0.01273, 4: 0.00617,	1: 0.00664, 2: 0.00601, 3: 0.00375, 4: 0.00484,	AZOBR_120014		
AZOLI_2439	1: 0.00021, 2: 0.00043, 3: 0.00040, 4: 0.00062,	1: 0.00044, 2: 0.00021, 3: 0.00054, 4: 0.00056,	AZOBR_120019		1: 0.00042, 2: 0.00024, 3: 0.00024,
AZOLI_2441		1: 0.00022, 2: 0.00019, 3: 0.00024, 4: 0.00005,	AZOBR_120020		1: 0.00004, 2: 0.00004, 3: 0.00003,
AZOLI_2443	3: 0.00007, 4: 0.00003,		AZOBR_120021		3: 0.00016,
AZOLI_2447	1: 0.00006,	1: 0.00009, 2: 0.00011, 3: 0.00012, 4: 0.00010,	AZOBR_120022	4: 0.00010,	1: 0.00042, 2: 0.00064, 3: 0.00067,
AZOLI_2449	2: 0.00009, 4: 0.00027,		AZOBR_120034	1: 0.00040, 4: 0.00048, 5: 0.00021,	1: 0.00102, 2: 0.00072, 3: 0.00111,
AZOLI_2450	2: 0.00009, 4: 0.00005,	1: 0.00009, 2: 0.00014, 3: 0.00016, 4: 0.00008,	AZOBR_120036	2: 0.00065, 3: 0.00038, 4: 0.00041, 5: 0.00082,	1: 0.00057, 2: 0.00032, 3: 0.00064,

AZOLI_2451	4: 0.00005,	1: 0.00005, 4: 0.00007,	AZOBR_120038	1: 0.00030, 2: 0.00036, 3: 0.00017, 4: 0.00042, 5: 0.00028,	
AZOLI_2454	1: 0.00075, 2: 0.00072, 3: 0.00019, 4: 0.00080,	1: 0.00103, 2: 0.00100, 3: 0.00052, 4: 0.00065,	AZOBR_120039	2: 0.00007, 3: 0.00016, 4: 0.00013, 5: 0.00005,	2: 0.00011, 3: 0.00009,
AZOLI_2457		4: 0.00006,	AZOBR_120040	1: 0.00145, 2: 0.00260, 3: 0.00113, 4: 0.00150, 5: 0.00236,	1: 0.01075, 2: 0.01175, 3: 0.00708,
AZOLI_2459	1: 0.01198, 2: 0.01630, 3: 0.00937, 4: 0.00704,	1: 0.00485, 2: 0.00431, 3: 0.00481, 4: 0.00455,	AZOBR_120043	4: 0.00031,	2: 0.00012,
AZOLI_2460	1: 0.00037, 2: 0.00040, 3: 0.00043, 4: 0.00032,	1: 0.00053, 2: 0.00039, 3: 0.00043, 4: 0.00032,	AZOBR_120044		1: 0.00008,
AZOLI_2465	2: 0.00009, 4: 0.00005,	4: 0.00005,	AZOBR_120048	1: 0.00015, 2: 0.00013, 3: 0.00016, 4: 0.00013, 5: 0.00020,	1: 0.00012, 2: 0.00025, 3: 0.00027,
AZOLI_2468	1: 0.00051, 2: 0.00059, 4: 0.00057,	3: 0.00047, 4: 0.00025,	AZOBR_120049	2: 0.00016,	3: 0.00012,
AZOLI_2469	1: 0.00002,		AZOBR_120050	1: 0.00065, 2: 0.00054, 3: 0.00067, 4: 0.00090, 5: 0.00081,	1: 0.00019, 2: 0.00051, 3: 0.00049,
AZOLI_2470	2: 0.00002,	2: 0.00002,	AZOBR_120052		
AZOLI_2471	1: 0.00019, 2: 0.00032, 3: 0.00038, 4: 0.00044,	1: 0.00030, 2: 0.00031, 3: 0.00029, 4: 0.00021,	AZOBR_120057	1: 0.00080, 2: 0.00082, 3: 0.00040, 4: 0.00128, 5: 0.00106,	1: 0.00060, 2: 0.00029, 3: 0.00046,
AZOLI_2473	1: 0.00045, 2: 0.00091, 3: 0.00117, 4: 0.00059,	1: 0.00130, 2: 0.00133, 3: 0.00227, 4: 0.00150,	AZOBR_120058	4: 0.00019, 5: 0.00037,	1: 0.00017, 3: 0.00067,
AZOLI_2474	3: 0.00060, 4: 0.00049,	1: 0.00115, 2: 0.00167, 3: 0.00153, 4: 0.00148,	AZOBR_120059	1: 0.00174, 2: 0.00089, 3: 0.00171, 4: 0.00180, 5: 0.00125,	1: 0.00056, 2: 0.00097, 3: 0.00033,
AZOLI_2475	4: 0.00013,	1: 0.00015, 2: 0.00015, 3: 0.00016, 4: 0.00013,	AZOBR_120060	1: 0.00141, 2: 0.00120, 3: 0.00094, 4: 0.00152, 5: 0.00166,	1: 0.00161, 2: 0.00091, 3: 0.00103,
AZOLI_2476	1: 0.00027, 2: 0.00017, 4: 0.00035,	1: 0.00028, 2: 0.00020, 3: 0.00022, 4: 0.00024,	AZOBR_120061	1: 0.00042, 3: 0.00036, 4: 0.00046, 5: 0.00047,	1: 0.00027, 2: 0.00025, 3: 0.00037,
AZOLI_2478	1: 0.00014, 4: 0.00019,	1: 0.00012, 2: 0.00014, 3: 0.00018, 4: 0.00012,	AZOBR_120063	1: 0.00010, 2: 0.00004, 3: 0.00004, 4: 0.00002, 5: 0.00002,	1: 0.00007, 2: 0.00006, 3: 0.00003,
AZOLI_2479	1: 0.00228, 2: 0.00196, 3: 0.00198, 4: 0.00184,	1: 0.00049, 2: 0.00062, 3: 0.00026, 4: 0.00048,	AZOBR_120064		1: 0.00013, 3: 0.00003,
AZOLI_2484	1: 0.00107, 2: 0.00040, 3: 0.00042, 4: 0.00075,	1: 0.00027, 2: 0.00020, 3: 0.00021, 4: 0.00014,	AZOBR_120065		
AZOLI_2485	1: 0.00019, 2: 0.00026, 4: 0.00029,	1: 0.00056, 2: 0.00035, 3: 0.00050, 4: 0.00042,	AZOBR_130001		2: 0.00033,
AZOLI_2490	1: 0.00014,		AZOBR_130002	1: 0.00019, 2: 0.00026, 3: 0.00029, 4: 0.00021, 5: 0.00015,	1: 0.00014, 2: 0.00008, 3: 0.00012,
AZOLI_2491	1: 0.00018, 2: 0.00025, 4: 0.00018,	1: 0.00032, 2: 0.00014, 3: 0.00023, 4: 0.00009,	AZOBR_130003	2: 0.00012, 4: 0.00019, 5: 0.00019,	
AZOLI_2492	4: 0.00023,		AZOBR_130005	1: 0.00023, 2: 0.00005, 3: 0.00005, 4: 0.00010,	1: 0.00018, 2: 0.00020, 3: 0.00010,
AZOLI_2496	1: 0.00010, 2: 0.00015, 4: 0.00036,		AZOBR_130006		
AZOLI_2501	2: 0.00015, 4: 0.00007,		AZOBR_130008	1: 0.00011, 3: 0.00006, 4: 0.00008,	1: 0.00017, 2: 0.00016, 3: 0.00041,
AZOLI_2503	2: 0.00031, 3: 0.00011, 4: 0.00013,	1: 0.00023, 2: 0.00034, 3: 0.00016, 4: 0.00007,	AZOBR_130010		
AZOLI_2511	4: 0.00015,	1: 0.00024, 3: 0.00013, 4: 0.00015,	AZOBR_130012	3: 0.00007, 4: 0.00009,	1: 0.00008, 2: 0.00009, 3: 0.00011,
AZOLI_2512		1: 0.00009, 2: 0.00012, 3: 0.00013, 4: 0.00004,	AZOBR_130016		
AZOLI_2517		4: 0.00020,	AZOBR_130017	1: 0.00131, 2: 0.00116, 3: 0.00081, 4: 0.00076, 5: 0.00112,	1: 0.00257, 2: 0.00280, 3: 0.00177,
AZOLI_2519	2: 0.00009, 4: 0.00011,	1: 0.00018, 2: 0.00008, 3: 0.00019,	AZOBR_130018	5: 0.00007,	1: 0.00004,
AZOLI_2520	1: 0.00050, 2: 0.00050, 3: 0.00025, 4: 0.00058,	1: 0.00021, 2: 0.00023, 3: 0.00035, 4: 0.00038,	AZOBR_130026	1: 0.00032, 2: 0.00042, 4: 0.00066,	1: 0.00153, 2: 0.00070, 3: 0.00099,
AZOLI_2523	1: 0.00075, 2: 0.00043, 3: 0.00032, 4: 0.00088,	1: 0.00007, 2: 0.00016, 4: 0.00011,	AZOBR_130027	1: 0.00021, 2: 0.00094, 3: 0.00027, 4: 0.00074, 5: 0.00103,	1: 0.00130, 2: 0.00109, 3: 0.00159,
AZOLI_2525		4: 0.00018,	AZOBR_130028		1: 0.00011, 3: 0.00021,
AZOLI_2526	1: 0.00028, 2: 0.00042, 3: 0.00072, 4: 0.00032,	1: 0.00112, 2: 0.00104, 3: 0.00107, 4: 0.00129,	AZOBR_130030	1: 0.00010, 4: 0.00007,	1: 0.00021, 2: 0.00005, 3: 0.00008,
AZOLI_2528	1: 0.00016, 2: 0.00016, 4: 0.00014,	1: 0.00033, 2: 0.00016, 3: 0.00026, 4: 0.00021,	AZOBR_130031		
AZOLI_2531		1: 0.00029, 2: 0.00028, 3: 0.00018, 4: 0.00034,	AZOBR_130037	1: 0.00019, 3: 0.00024, 4: 0.00039,	2: 0.00035,
AZOLI_2547	1: 0.00037, 2: 0.00083, 3: 0.00039, 4: 0.00055,	1: 0.00033, 2: 0.00045, 3: 0.00034, 4: 0.00020,	AZOBR_130039	2: 0.00006, 3: 0.00009, 4: 0.00005,	
AZOLI_2558	4: 0.00005,	1: 0.00024, 2: 0.00046, 3: 0.00016, 4: 0.00025,	AZOBR_130040	1: 0.00017, 3: 0.00014, 4: 0.00034,	3: 0.00015,
AZOLI_2562		1: 0.00013, 3: 0.00025, 4: 0.00017,	AZOBR_130044		2: 0.00008,
AZOLI_2563		1: 0.00007, 2: 0.00006, 3: 0.00014,	AZOBR_140002	1: 0.00041, 2: 0.00019, 3: 0.00028, 4: 0.00033, 5: 0.00022,	1: 0.00025, 2: 0.00014, 3: 0.00028,
AZOLI_2564	1: 0.00007, 2: 0.00007, 3: 0.00007, 4: 0.00006,	3: 0.00004, 4: 0.00004,	AZOBR_140004	4: 0.00095, 5: 0.00095,	1: 0.00050, 3: 0.00024,
AZOLI_2565	2: 0.00007, 4: 0.00006,		AZOBR_140005	1: 0.00154, 2: 0.00099, 3: 0.00071,	1: 0.00089, 2: 0.00071, 3: 0.00043,
AZOLI_2572		2: 0.00068, 4: 0.00105,	AZOBR_140006		1: 0.00009,
AZOLI_2573	2: 0.00005,		AZOBR_140007	1: 0.00016, 3: 0.00009, 4: 0.00011,	
AZOLI_2574	2: 0.00040, 3: 0.00033, 4: 0.00034,	1: 0.00127, 2: 0.00123, 3: 0.00093, 4: 0.00081,	AZOBR_140009	2: 0.00072, 3: 0.00040, 4: 0.00048, 5: 0.00028,	1: 0.00056, 2: 0.00055, 3: 0.00079,
AZOLI_2575	1: 0.00009, 4: 0.00007,	1: 0.00009, 2: 0.00010, 3: 0.00007, 4: 0.00015,	AZOBR_140011	4: 0.00014, 5: 0.00014,	3: 0.00022,
AZOLI_2579	1: 0.00010, 2: 0.00010, 3: 0.00009, 4: 0.00005,	1: 0.00029, 2: 0.00040, 3: 0.00034, 4: 0.00019,	AZOBR_140012	1: 0.00008, 3: 0.00007, 4: 0.00011, 5: 0.00008,	2: 0.00009,
AZOLI_2580	1: 0.00036, 2: 0.00037, 3: 0.00026, 4: 0.00016,	1: 0.00148, 2: 0.00160, 3: 0.00108, 4: 0.00063,	AZOBR_140014		
AZOLI_2583		3: 0.00034,	AZOBR_140016	4: 0.00005,	
AZOLI_2591	3: 0.00074,	3: 0.00042,	AZOBR_140017	1: 0.00139, 2: 0.00131, 3: 0.00110,	1: 0.00085, 2: 0.00092,

				4: 0.00096, 5: 0.00109,	3: 0.00118,
AZOLI_2595	2: 0.00070, 3: 0.00146, 4: 0.00074,	1: 0.00070, 2: 0.00042, 3: 0.00056, 4: 0.00022,	AZOBR_140018	1: 0.00101, 2: 0.00081, 3: 0.00117, 4: 0.00089, 5: 0.00089,	1: 0.00071, 2: 0.00047, 3: 0.00154,
AZOLI_2596	1: 0.00068, 2: 0.00081, 3: 0.00069, 4: 0.00122,	1: 0.00025, 2: 0.00048, 3: 0.00033, 4: 0.00042,	AZOBR_140023	1: 0.00008, 3: 0.00010,	
AZOLI_2598	1: 0.00036, 2: 0.00023, 3: 0.00019, 4: 0.00094,	1: 0.00007, 2: 0.00015, 3: 0.00012, 4: 0.00017,	AZOBR_140024		
AZOLI_2599	1: 0.00067, 2: 0.00095, 3: 0.00039, 4: 0.00062,	1: 0.00051, 2: 0.00060, 3: 0.00036, 4: 0.00034,	AZOBR_140025	1: 0.00052, 2: 0.00126, 3: 0.00099, 4: 0.00098, 5: 0.00098,	1: 0.00100, 2: 0.00104, 3: 0.00097,
AZOLI_2601	1: 0.00115, 2: 0.00058, 3: 0.00358, 4: 0.00177,	1: 0.00108, 2: 0.00272, 3: 0.00211, 4: 0.00205,	AZOBR_140030		
AZOLI_2602	1: 0.00560, 2: 0.00384, 3: 0.00970, 4: 0.00386,	1: 0.00667, 2: 0.00525, 3: 0.00526, 4: 0.00603,	AZOBR_140031		
AZOLI_2603	1: 0.00054, 2: 0.00050, 3: 0.00227, 4: 0.00155,	1: 0.00121, 2: 0.00169, 3: 0.00123, 4: 0.00166,	AZOBR_140032		
AZOLI_2604	1: 0.00220, 2: 0.00209, 3: 0.00476, 4: 0.00314,	1: 0.00221, 2: 0.00217, 3: 0.00219, 4: 0.00238,	AZOBR_140042		
AZOLI_2605	1: 0.00527, 2: 0.00580, 3: 0.00776, 4: 0.00310,	1: 0.00449, 2: 0.00281, 3: 0.00315, 4: 0.00383,	AZOBR_140045		2: 0.00013, 3: 0.00007,
AZOLI_2619	1: 0.00090, 2: 0.00120, 3: 0.00284, 4: 0.00084,	1: 0.00138, 2: 0.00169, 3: 0.00157, 4: 0.00177,	AZOBR_140047		2: 0.00008, 3: 0.00010,
AZOLI_2623		1: 0.00021, 2: 0.00018, 3: 0.00023, 4: 0.00030,	AZOBR_140051	4: 0.00026,	3: 0.00012,
AZOLI_2628	1: 0.00044, 2: 0.00088, 4: 0.00052,	1: 0.00022, 2: 0.00059, 3: 0.00041, 4: 0.00028,	AZOBR_140052	1: 0.00036, 3: 0.00030, 5: 0.00010,	1: 0.00009,
AZOLI_2629	4: 0.00005,	4: 0.00004,	AZOBR_140054		1: 0.00006, 2: 0.00009, 3: 0.00016,
AZOLI_2630	1: 0.00134, 2: 0.00155, 3: 0.00297, 4: 0.00206,	1: 0.00228, 2: 0.00234, 3: 0.00226, 4: 0.00239,	AZOBR_140061	3: 0.00006, 4: 0.00010, 5: 0.00005,	1: 0.00012, 2: 0.00011, 3: 0.00024,
AZOLI_2632		4: 0.00003,	AZOBR_140062	4: 0.00018,	1: 0.00017, 2: 0.00025, 3: 0.00011,
AZOLI_2633	1: 0.00190, 2: 0.00203, 3: 0.00536, 4: 0.00146,	1: 0.00493, 2: 0.00320, 3: 0.00340, 4: 0.00320,	AZOBR_140065	3: 0.00005,	
AZOLI_2635	1: 0.00017, 2: 0.00012, 3: 0.00016,	2: 0.00017, 4: 0.00015,	AZOBR_140066		
AZOLI_2641	1: 0.00053, 2: 0.00054, 3: 0.00090, 4: 0.00080,	1: 0.00065, 2: 0.00076, 3: 0.00076, 4: 0.00049,	AZOBR_140072		3: 0.00006,
AZOLI_2646	4: 0.00009,	4: 0.00007,	AZOBR_140074	5: 0.00018,	
AZOLI_2649	1: 0.00048, 2: 0.00063, 4: 0.00057,	1: 0.00034, 2: 0.00033, 3: 0.00063, 4: 0.00036,	AZOBR_140091		
AZOLI_2650	1: 0.00014, 2: 0.00011, 3: 0.00028, 4: 0.00016,	1: 0.00024, 2: 0.00023, 3: 0.00027, 4: 0.00028,	AZOBR_140103	4: 0.00008, 5: 0.00008,	1: 0.00062, 2: 0.00021, 3: 0.00074,
AZOLI_2651	4: 0.00005,		AZOBR_140104		3: 0.00016,
AZOLI_2652	2: 0.00027, 4: 0.00023,	4: 0.00023,	AZOBR_140105		
AZOLI_2657		4: 0.00013,	AZOBR_140106		
AZOLI_2659		1: 0.00014,	AZOBR_140107	2: 0.00003, 3: 0.00005,	3: 0.00004,
AZOLI_2660	2: 0.00009, 4: 0.00015,	3: 0.00018, 4: 0.00007,	AZOBR_140110		
AZOLI_2661		1: 0.00013, 2: 0.00013, 3: 0.00018, 4: 0.00011,	AZOBR_140112	1: 0.00006,	3: 0.00002,
AZOLI_2663	1: 0.00280, 2: 0.00165, 3: 0.00514, 4: 0.00217,	1: 0.00219, 2: 0.00277, 3: 0.00263, 4: 0.00322,	AZOBR_140113	1: 0.00135, 2: 0.00080, 3: 0.00115, 4: 0.00106, 5: 0.00050,	1: 0.00052, 2: 0.00059, 3: 0.00067,
AZOLI_2664	1: 0.00015, 2: 0.00015, 4: 0.00019,	1: 0.00038, 2: 0.00015, 4: 0.00019,	AZOBR_140118		3: 0.00009,
AZOLI_2665	2: 0.00024,	4: 0.00020,	AZOBR_140120		3: 0.00012,
AZOLI_2666	4: 0.00009,	4: 0.00009,	AZOBR_140121	1: 0.00096, 2: 0.00122, 3: 0.00113, 4: 0.00127, 5: 0.00131,	1: 0.00135, 2: 0.00089, 3: 0.00107,
AZOLI_2667	1: 0.00005,		AZOBR_140122		2: 0.00009, 3: 0.00004,
AZOLI_2668	1: 0.00020, 2: 0.00014, 3: 0.00029, 4: 0.00009,	1: 0.00024, 2: 0.00030, 3: 0.00018, 4: 0.00023,	AZOBR_140124		
AZOLI_2679		1: 0.00010, 2: 0.00009, 3: 0.00051, 4: 0.00014,	AZOBR_140126	1: 0.00020, 3: 0.00025, 4: 0.00010, 5: 0.00014,	1: 0.00010, 2: 0.00014, 3: 0.00018,
AZOLI_2683	2: 0.00111, 3: 0.00028, 4: 0.00052,	4: 0.00017,	AZOBR_140129	2: 0.00004, 3: 0.00005, 4: 0.00004, 5: 0.00004,	2: 0.00008, 3: 0.00004,
AZOLI_2685	1: 0.00914, 2: 0.00718, 3: 0.01451, 4: 0.00792,	1: 0.00757, 2: 0.00681, 3: 0.00875, 4: 0.00840,	AZOBR_140131	1: 0.00026,	1: 0.00011, 2: 0.00019, 3: 0.00037,
AZOLI_2686	1: 0.00766, 2: 0.00654, 3: 0.00830, 4: 0.00711,	1: 0.00526, 2: 0.00725, 3: 0.00493, 4: 0.00601,	AZOBR_140132		
AZOLI_2697		1: 0.00023, 2: 0.00011, 3: 0.00029, 4: 0.00023,	AZOBR_140133		
AZOLI_2699		1: 0.00079, 2: 0.00054, 3: 0.00074, 4: 0.00060,	AZOBR_140134	2: 0.00004,	
AZOLI_2700		1: 0.00014, 4: 0.00006,	AZOBR_140135		1: 0.00185, 2: 0.00291, 3: 0.00111,
AZOLI_2703	1: 0.00014, 2: 0.00020, 4: 0.00031,	1: 0.00017, 2: 0.00011, 3: 0.00015, 4: 0.00022,	AZOBR_140136		1: 0.00021, 2: 0.00004, 3: 0.00010,
AZOLI_2705	1: 0.00005,		AZOBR_140137	2: 0.00033, 3: 0.00031, 4: 0.00119, 5: 0.00051,	1: 0.00032, 2: 0.00027, 3: 0.00076,
AZOLI_2710	2: 0.00018, 3: 0.00043,	1: 0.00053, 2: 0.00056, 3: 0.00052, 4: 0.00057,	AZOBR_140140	1: 0.00691, 2: 0.00885, 3: 0.00714, 4: 0.00663, 5: 0.00861,	1: 0.00930, 2: 0.00853, 3: 0.00744,
AZOLI_2712	1: 0.00036, 2: 0.00045, 3: 0.00012, 4: 0.00034,	1: 0.00009, 3: 0.00009, 4: 0.00015,	AZOBR_140149		1: 0.00006, 3: 0.00006,
AZOLI_2715	1: 0.00043, 2: 0.00014, 3: 0.00094, 4: 0.00078,	1: 0.00067, 2: 0.00056, 3: 0.00087, 4: 0.00037,	AZOBR_140158	1: 0.00005, 2: 0.00011,	
AZOLI_2717	1: 0.00069, 2: 0.00048, 3: 0.00015, 4: 0.00027,	1: 0.00059, 2: 0.00046, 3: 0.00034, 4: 0.00046,	AZOBR_140159	1: 0.00023, 2: 0.00014, 3: 0.00038, 4: 0.00031, 5: 0.00022,	1: 0.00021, 2: 0.00021, 3: 0.00018,
AZOLI_2718	1: 0.00023, 2: 0.00038, 3: 0.00033, 4: 0.00049,	1: 0.00019, 2: 0.00037, 3: 0.00045, 4: 0.00057,	AZOBR_140160	1: 0.00051, 2: 0.00062, 3: 0.00055, 4: 0.00040, 5: 0.00031,	1: 0.00012, 2: 0.00014, 3: 0.00020,
AZOLI_2721	1: 0.00012, 2: 0.00030, 3: 0.00017, 4: 0.00008,	1: 0.00018, 2: 0.00016, 3: 0.00016, 4: 0.00007,	AZOBR_140166	3: 0.00017, 4: 0.00010, 5: 0.00010,	2: 0.00007,
AZOLI_2724	1: 0.00012, 2: 0.00036,	1: 0.00042, 2: 0.00017,	AZOBR_140167	2: 0.00010, 3: 0.00010, 4: 0.00014,	1: 0.00007, 3: 0.00007,

	4: 0.00036,	3: 0.00025,		5: 0.00008,	
AZOLI_2726	1: 0.00042, 2: 0.00074, 4: 0.00050,	1: 0.00027, 2: 0.00026, 3: 0.00034, 4: 0.00014,	AZOBR_140169	1: 0.00047, 2: 0.00047, 3: 0.00067, 4: 0.00061, 5: 0.00024,	1: 0.00026, 2: 0.00046, 3: 0.00022,
AZOLI_2732		2: 0.00005,	AZOBR_140171		
AZOLI_2737	3: 0.00159,	1: 0.00079, 2: 0.00138, 3: 0.00129, 4: 0.00202,	AZOBR_140172		
AZOLI_2740	1: 0.00073, 2: 0.00094, 3: 0.00102, 4: 0.00098,	1: 0.00137, 2: 0.00071, 3: 0.00111, 4: 0.00081,	AZOBR_140174	2: 0.00013, 4: 0.00013,	3: 0.00005,
AZOLI_2742	4: 0.00029,		AZOBR_140175		3: 0.00016,
AZOLI_2745	1: 0.00064, 2: 0.00060, 3: 0.00110, 4: 0.00102,	1: 0.00084, 2: 0.00085, 3: 0.00100, 4: 0.00106,	AZOBR_140177		1: 0.00009,
AZOLI_2747	1: 0.00018, 2: 0.00019, 3: 0.00011, 4: 0.00018,	1: 0.00021, 2: 0.00015, 3: 0.00020, 4: 0.00020,	AZOBR_140180	1: 0.00004,	
AZOLI_2748	3: 0.00012,	1: 0.00022, 2: 0.00034, 3: 0.00014, 4: 0.00007,	AZOBR_140184	4: 0.00029, 5: 0.00029,	
AZOLI_2751	2: 0.00008, 4: 0.00010,	2: 0.00015, 3: 0.00008, 4: 0.00030,	AZOBR_140186	4: 0.00020, 5: 0.00026,	1: 0.00037, 2: 0.00021, 3: 0.00047,
AZOLI_2755	1: 0.00007, 3: 0.00006, 4: 0.00006,	1: 0.00004, 2: 0.00006, 4: 0.00004,	AZOBR_140189	1: 0.00035, 3: 0.00019, 4: 0.00024,	
AZOLI_2757	1: 0.00034, 2: 0.00021, 3: 0.00048, 4: 0.00021,	1: 0.00042, 2: 0.00050, 3: 0.00048, 4: 0.00027,	AZOBR_140191	1: 0.00050, 2: 0.00044, 3: 0.00018, 4: 0.00039, 5: 0.00044,	1: 0.00014,
AZOLI_2758	1: 0.00011, 2: 0.00022, 3: 0.00024, 4: 0.00019,	3: 0.00042, 4: 0.00019,	AZOBR_140192	1: 0.00009, 3: 0.00005, 4: 0.00008, 5: 0.00004,	3: 0.00004,
AZOLI_2761	1: 0.00039, 2: 0.00072, 3: 0.00093, 4: 0.00078,	1: 0.00056, 2: 0.00073, 3: 0.00095, 4: 0.00071,	AZOBR_140194		
AZOLI_2763	1: 0.00018,		AZOBR_140200	2: 0.00008, 4: 0.00018, 5: 0.00018,	1: 0.00079, 2: 0.00045, 3: 0.00082,
AZOLI_2765	1: 0.00141, 2: 0.00037, 4: 0.00080,	1: 0.00031, 2: 0.00048, 3: 0.00073, 4: 0.00037,	AZOBR_140206	2: 0.00028,	
AZOLI_2766	1: 0.00006,	4: 0.00005,	AZOBR_140214	4: 0.00034,	3: 0.00031,
AZOLI_2768	3: 0.00019,	1: 0.00172, 2: 0.00140, 3: 0.00178, 4: 0.00155,	AZOBR_140216		3: 0.00017,
AZOLI_2774	1: 0.00085, 3: 0.00103, 4: 0.00063,	1: 0.00074, 2: 0.00095, 3: 0.00131,	AZOBR_140217	1: 0.00026, 2: 0.00027, 3: 0.00037, 4: 0.00043, 5: 0.00036,	1: 0.00014, 2: 0.00017, 3: 0.00016,
AZOLI_2776	1: 0.00007, 2: 0.00007,	1: 0.00025, 2: 0.00014, 3: 0.00034, 4: 0.00012,	AZOBR_140219		
AZOLI_2777	1: 0.00015, 2: 0.00009, 4: 0.00008,	2: 0.00006, 4: 0.00008,	AZOBR_140220	4: 0.00003,	1: 0.00001, 2: 0.00002, 3: 0.00003,
AZOLI_2778		1: 0.00009,	AZOBR_140222		
AZOLI_2785	1: 0.00102, 2: 0.00066, 3: 0.00144, 4: 0.00044,	1: 0.00074, 2: 0.00100, 3: 0.00071, 4: 0.00101,	AZOBR_140224	4: 0.00010,	1: 0.00009, 2: 0.00014,
AZOLI_2786	4: 0.00003,	3: 0.00004, 4: 0.00005,	AZOBR_140230	4: 0.00036, 5: 0.00022,	3: 0.00013,
AZOLI_2787		1: 0.00029, 2: 0.00048, 3: 0.00046, 4: 0.00024,	AZOBR_140234		
AZOLI_2788		1: 0.00140, 2: 0.00148, 3: 0.00116, 4: 0.00128,	AZOBR_140235	1: 0.00128, 2: 0.00253, 3: 0.00118, 4: 0.00252, 5: 0.00218,	1: 0.00247, 2: 0.00252, 3: 0.00227,
AZOLI_2793	1: 0.00187, 2: 0.00177, 3: 0.00080, 4: 0.00139,	1: 0.00095, 2: 0.00091, 3: 0.00094, 4: 0.00093,	AZOBR_140238	1: 0.00071, 2: 0.00063, 3: 0.00104, 4: 0.00119, 5: 0.00105,	1: 0.00039, 2: 0.00044, 3: 0.00019,
AZOLI_2794	1: 0.00048, 2: 0.00137, 3: 0.00146, 4: 0.00206,	1: 0.00339, 2: 0.00233, 3: 0.00325, 4: 0.00379,	AZOBR_140239	1: 0.00023, 2: 0.00079, 3: 0.00034, 4: 0.00053, 5: 0.00044,	1: 0.00034, 2: 0.00024, 3: 0.00039,
AZOLI_2795	1: 0.00009, 2: 0.00006, 3: 0.00187, 4: 0.00005,	1: 0.00663, 2: 0.00608, 3: 0.00675, 4: 0.00644,	AZOBR_140240	4: 0.00013,	3: 0.00012,
AZOLI_2801	3: 0.00024,		AZOBR_140242	2: 0.00026, 3: 0.00019, 5: 0.00010,	1: 0.00029, 2: 0.00016, 3: 0.00042,
AZOLI_2807	1: 0.00011, 2: 0.00011, 4: 0.00046,	1: 0.00049, 2: 0.00037, 3: 0.00029, 4: 0.00019,	AZOBR_140243		1: 0.00015, 2: 0.00006, 3: 0.00012,
AZOLI_2811	1: 0.00061, 2: 0.00031, 3: 0.00025, 4: 0.00064,	1: 0.00040, 2: 0.00047, 3: 0.00024, 4: 0.00034,	AZOBR_140244	1: 0.00019, 4: 0.00019,	1: 0.00018, 3: 0.00035,
AZOLI_2812	1: 0.00048, 2: 0.00060, 3: 0.00086, 4: 0.00049,	1: 0.00056, 2: 0.00028, 3: 0.00033, 4: 0.00039,	AZOBR_140245	1: 0.00053, 2: 0.00020, 3: 0.00046, 4: 0.00026, 5: 0.00021,	1: 0.00024, 2: 0.00039, 3: 0.00028,
AZOLI_2817	1: 0.00078, 2: 0.00026,	1: 0.00066,	AZOBR_140248		
AZOLI_2819	4: 0.00005,	3: 0.00009, 4: 0.00013,	AZOBR_140249		
AZOLI_2820	1: 0.00192, 2: 0.00156, 3: 0.00435, 4: 0.00075,	1: 0.00082, 2: 0.00097, 3: 0.00066, 4: 0.00080,	AZOBR_140251	1: 0.00022, 4: 0.00020, 5: 0.00020,	3: 0.00022,
AZOLI_2821		1: 0.00065, 2: 0.00036, 3: 0.00029, 4: 0.00032,	AZOBR_140253		2: 0.00052, 3: 0.00044,
AZOLI_2825	2: 0.00006, 4: 0.00010,	1: 0.00023, 2: 0.00020, 3: 0.00019, 4: 0.00022,	AZOBR_140254	5: 0.00006,	3: 0.00008,
AZOLI_2829	1: 0.00020, 2: 0.00016, 3: 0.00086, 4: 0.00010,	1: 0.00164, 2: 0.00186, 3: 0.00170, 4: 0.00182,	AZOBR_140255	1: 0.00013, 3: 0.00008, 4: 0.00015,	3: 0.00011,
AZOLI_2836	2: 0.00026, 4: 0.00067,	1: 0.00079, 3: 0.00070, 4: 0.00067,	AZOBR_140256	1: 0.00065, 2: 0.00023, 3: 0.00022, 4: 0.00045, 5: 0.00027,	1: 0.00108, 2: 0.00076, 3: 0.00072,
AZOLI_2844		4: 0.00011,	AZOBR_140257	4: 0.00012,	3: 0.00027,
AZOLI_2845	4: 0.00010,		AZOBR_140258		
AZOLI_2846	4: 0.00005,		AZOBR_140261	1: 0.00158, 2: 0.00194, 3: 0.00127, 4: 0.00109, 5: 0.00176,	1: 0.00163, 2: 0.00147, 3: 0.00208,
AZOLI_2848	2: 0.00006, 4: 0.00005,	1: 0.00006, 3: 0.00006, 4: 0.00010,	AZOBR_140262	1: 0.00008, 2: 0.00005, 3: 0.00015, 4: 0.00020, 5: 0.00007,	2: 0.00004, 3: 0.00003,
AZOLI_2849	2: 0.00010, 4: 0.00006,	3: 0.00005, 4: 0.00006,	AZOBR_140263	1: 0.00327, 2: 0.00346, 3: 0.00200, 4: 0.00171, 5: 0.00198,	1: 0.00067, 2: 0.00105, 3: 0.00106,
AZOLI_2850	1: 0.00004, 2: 0.00009, 3: 0.00007, 4: 0.00018,	1: 0.00025, 2: 0.00034, 3: 0.00063, 4: 0.00039,	AZOBR_140264		
AZOLI_2853	1: 0.00031, 2: 0.00045, 4: 0.00066,	2: 0.00017,	AZOBR_140265	1: 0.00016, 2: 0.00028, 3: 0.00033, 4: 0.00011, 5: 0.00022,	1: 0.00010, 3: 0.00034,
AZOLI_2854	1: 0.00015, 2: 0.00062, 3: 0.00140, 4: 0.00105,	1: 0.00178, 2: 0.00097, 3: 0.00197, 4: 0.00145,	AZOBR_140266	1: 0.00024, 2: 0.00016, 3: 0.00030, 4: 0.00025, 5: 0.00057,	
AZOLI_2857	1: 0.00006, 2: 0.00024, 4: 0.00018,	1: 0.00012, 2: 0.00015, 4: 0.00013,	AZOBR_140267	1: 0.00066, 2: 0.00097, 3: 0.00084, 4: 0.00060, 5: 0.00064,	
AZOLI_2859		2: 0.00006, 3: 0.00012,	AZOBR_140268	1: 0.00023, 2: 0.00027, 3: 0.00024, 4: 0.00032, 5: 0.00026,	

AZOLI_2860	1: 0.00008, 2: 0.00006, 4: 0.00009,		AZOBR_140269	1: 0.00669, 2: 0.00628, 3: 0.00483, 4: 0.00391, 5: 0.00517,	1: 0.00420, 2: 0.00487, 3: 0.00337,
AZOLI_2863	1: 0.00050, 2: 0.00064, 3: 0.00120, 4: 0.00047,	1: 0.00128, 2: 0.00218, 3: 0.00139, 4: 0.00143,	AZOBR_140271	3: 0.00010, 4: 0.00020, 5: 0.00012,	3: 0.00025,
AZOLI_2869	4: 0.00011,		AZOBR_140273		3: 0.00013,
AZOLI_2870	1: 0.00089, 2: 0.00143, 3: 0.00094, 4: 0.00128,	1: 0.00128, 2: 0.00094, 3: 0.00088, 4: 0.00109,	AZOBR_140274		
AZOLI_2871	1: 0.00312, 2: 0.00402, 3: 0.00439, 4: 0.00294,	1: 0.00152, 2: 0.00321, 3: 0.00229, 4: 0.00234,	AZOBR_140277	3: 0.00048, 5: 0.00023,	3: 0.00021,
AZOLI_2872	1: 0.00008, 2: 0.00008, 3: 0.00018, 4: 0.00009,	1: 0.00013, 2: 0.00012, 3: 0.00018, 4: 0.00018,	AZOBR_140279	1: 0.00010, 2: 0.00013, 3: 0.00021, 4: 0.00014, 5: 0.00021,	3: 0.00009,
AZOLI_2873	1: 0.00013, 2: 0.00017, 3: 0.00018, 4: 0.00026,	1: 0.00017, 2: 0.00013, 3: 0.00014, 4: 0.00011,	AZOBR_140281	3: 0.00020, 4: 0.00011,	1: 0.00030, 2: 0.00068, 3: 0.00024,
AZOLI_2874	1: 0.00039, 2: 0.00091, 4: 0.00071,	1: 0.00075, 2: 0.00050, 3: 0.00046, 4: 0.00037,	AZOBR_140285	1: 0.00025, 2: 0.00011, 3: 0.00010, 4: 0.00023, 5: 0.00030,	2: 0.00018, 3: 0.00006,
AZOLI_2876	1: 0.00006, 3: 0.00014, 4: 0.00008,		AZOBR_140288		
AZOLI_2877	1: 0.00038, 2: 0.00038, 4: 0.00019,	1: 0.00022, 2: 0.00011, 3: 0.00018, 4: 0.00014,	AZOBR_140290		
AZOLI_2881	1: 0.00063, 2: 0.00025, 3: 0.00039, 4: 0.00051,	1: 0.00053, 2: 0.00103, 3: 0.00060, 4: 0.00064,	AZOBR_140291		
AZOLI_2889	1: 0.00021, 3: 0.00015, 4: 0.00012,	1: 0.00018, 2: 0.00022, 3: 0.00011, 4: 0.00014,	AZOBR_140292		
AZOLI_2889	1: 0.00038, 2: 0.00052, 3: 0.00036, 4: 0.00050,	1: 0.00013, 2: 0.00044, 3: 0.00021, 4: 0.00055,	AZOBR_140297		
AZOLI_2890	1: 0.00016, 2: 0.00017, 3: 0.00010, 4: 0.00021,	1: 0.00023, 2: 0.00025, 3: 0.00018, 4: 0.00021,	AZOBR_140302		
AZOLI_2894	3: 0.00014,	2: 0.00007, 3: 0.00013, 4: 0.00006,	AZOBR_140303		
AZOLI_2896		3: 0.00014,	AZOBR_140305		
AZOLI_2898		2: 0.00013,	AZOBR_140306		3: 0.00015,
AZOLI_2899	1: 0.00014, 2: 0.00014, 3: 0.00032, 4: 0.00008,	2: 0.00018, 3: 0.00020,	AZOBR_140307	1: 0.00023, 2: 0.00041, 4: 0.00040, 5: 0.00024,	1: 0.00037, 2: 0.00067, 3: 0.00029,
AZOLI_2901	2: 0.00030, 3: 0.00247, 4: 0.00034,		AZOBR_140308		3: 0.00005,
AZOLI_2902	1: 0.00260, 2: 0.00206, 3: 0.00255, 4: 0.00107,	1: 0.00402, 2: 0.00365, 3: 0.00304, 4: 0.00371,	AZOBR_140309	1: 0.00068, 2: 0.00049, 3: 0.00061, 4: 0.00039, 5: 0.00036,	1: 0.00004, 2: 0.00025, 3: 0.00017,
AZOLI_2903	4: 0.00004,		AZOBR_140310	1: 0.00088, 2: 0.00082, 3: 0.00098, 4: 0.00108, 5: 0.00090,	1: 0.00063, 2: 0.00076, 3: 0.00086,
AZOLI_2905	1: 0.00177, 2: 0.00272, 3: 0.00353, 4: 0.00258,	1: 0.00149, 2: 0.00203, 3: 0.00138, 4: 0.00100,	AZOBR_140311	1: 0.00004,	2: 0.00008,
AZOLI_2908		1: 0.00007, 4: 0.00009,	AZOBR_150005		
AZOLI_2909	1: 0.00011, 2: 0.00009, 3: 0.00014, 4: 0.00007,	1: 0.00022, 2: 0.00025, 3: 0.00024, 4: 0.00028,	AZOBR_150011	1: 0.00239, 2: 0.00334, 3: 0.00373, 4: 0.00267, 5: 0.00281,	1: 0.00038, 2: 0.00065, 3: 0.00074,
AZOLI_2918	1: 0.00020, 2: 0.00017,		AZOBR_150011	1: 0.00308, 2: 0.00352, 3: 0.00343, 4: 0.00313, 5: 0.00341,	1: 0.00105, 2: 0.00142, 3: 0.00156,
AZOLI_2920	1: 0.00171, 2: 0.00097, 3: 0.00016, 4: 0.00045,	1: 0.00056, 2: 0.00029, 3: 0.00048, 4: 0.00035,	AZOBR_150011	1: 0.00301, 2: 0.00331, 3: 0.00288, 4: 0.00314, 5: 0.00397,	1: 0.00152, 2: 0.00146, 3: 0.00233,
AZOLI_2921	1: 0.00030,		AZOBR_150016	1: 0.00039, 2: 0.00017, 3: 0.00063, 4: 0.00054, 5: 0.00048,	1: 0.00030, 2: 0.00026, 3: 0.00049,
AZOLI_2925	1: 0.00008, 4: 0.00009,	3: 0.00011, 4: 0.00009,	AZOBR_150017	3: 0.00012, 4: 0.00014, 5: 0.00024,	2: 0.00010, 3: 0.00009,
AZOLI_2926	3: 0.00029, 4: 0.00021,	1: 0.00014, 2: 0.00010, 3: 0.00026, 4: 0.00015,	AZOBR_150020	3: 0.00011, 4: 0.00012, 5: 0.00006,	1: 0.00028, 2: 0.00026, 3: 0.00033,
AZOLI_2929	1: 0.00032, 2: 0.00041, 3: 0.00017, 4: 0.00049,	1: 0.00045, 2: 0.00051, 3: 0.00052, 4: 0.00021,	AZOBR_150021		
AZOLI_2930	1: 0.00020, 2: 0.00031, 4: 0.00010,	1: 0.00011, 2: 0.00016, 3: 0.00012, 4: 0.00010,	AZOBR_150022	1: 0.00116, 2: 0.00116, 3: 0.00131, 4: 0.00106, 5: 0.00096,	1: 0.00054, 2: 0.00079, 3: 0.00091,
AZOLI_2932	1: 0.00082, 2: 0.00099, 4: 0.00226,	1: 0.00033, 4: 0.00028,	AZOBR_150023	1: 0.00153, 2: 0.00144, 3: 0.00139, 4: 0.00141, 5: 0.00130,	1: 0.00075, 2: 0.00108, 3: 0.00067,
AZOLI_2934	1: 0.00006, 2: 0.00014, 3: 0.00008, 4: 0.00010,	1: 0.00056, 2: 0.00022, 3: 0.00042, 4: 0.00034,	AZOBR_150025		
AZOLI_2935	1: 0.00037, 2: 0.00068, 3: 0.00053, 4: 0.00032,	1: 0.00053, 2: 0.00022, 4: 0.00039,	AZOBR_150026		
AZOLI_2940	3: 0.00009,		AZOBR_150028	2: 0.00007, 3: 0.00019, 4: 0.00013, 5: 0.00006,	1: 0.00004, 2: 0.00006,
AZOLI_2944	1: 0.00027, 2: 0.00024, 3: 0.00029, 4: 0.00015,	1: 0.00030, 2: 0.00038, 3: 0.00016, 4: 0.00026,	AZOBR_150029		
AZOLI_2947	1: 0.00011,		AZOBR_150030	2: 0.00031, 3: 0.00040, 4: 0.00024, 5: 0.00040,	2: 0.00017, 3: 0.00036,
AZOLI_2949	1: 0.00037, 2: 0.00088, 4: 0.00129,	1: 0.00189, 2: 0.00182, 3: 0.00200, 4: 0.00172,	AZOBR_150031	1: 0.00012, 2: 0.00038, 3: 0.00020, 4: 0.00041, 5: 0.00022,	1: 0.00048, 2: 0.00046, 3: 0.00027,
AZOLI_2950	4: 0.00010,		AZOBR_150033		2: 0.00026, 3: 0.00045,
AZOLI_2953	1: 0.00009, 2: 0.00009, 3: 0.00010, 4: 0.00006,	1: 0.00011, 2: 0.00009, 3: 0.00008, 4: 0.00008,	AZOBR_150034		
AZOLI_2957		4: 0.00007,	AZOBR_150035		3: 0.00009,
AZOLI_2958	4: 0.00005,	4: 0.00003,	AZOBR_150037	1: 0.00013, 2: 0.00008, 4: 0.00009,	
AZOLI_2959	1: 0.00023, 4: 0.00039,	4: 0.00029,	AZOBR_150040	4: 0.00024,	
AZOLI_2960	1: 0.00146, 2: 0.00078, 3: 0.00084, 4: 0.00118,	1: 0.00130, 2: 0.00151, 3: 0.00156, 4: 0.00126,	AZOBR_150042	1: 0.00290, 2: 0.00328, 3: 0.00278, 4: 0.00212, 5: 0.00275,	1: 0.00046, 2: 0.00040, 3: 0.00090,
AZOLI_2961	2: 0.00010,		AZOBR_150047		
AZOLI_2962	1: 0.00014,		AZOBR_150048	1: 0.00015, 2: 0.00014, 3: 0.00011,	1: 0.00012, 2: 0.00013, 3: 0.00012,
AZOLI_2966	1: 0.00034, 2: 0.00040, 3: 0.00048, 4: 0.00013,	1: 0.00025, 2: 0.00014, 3: 0.00016, 4: 0.00013,	AZOBR_150060		
AZOLI_2969	1: 0.00004, 2: 0.00006, 3: 0.00016, 4: 0.00013,	1: 0.00023, 2: 0.00027, 3: 0.00042, 4: 0.00039,	AZOBR_150061		
AZOLI_2971	1: 0.00008, 2: 0.00004, 4: 0.00007,	1: 0.00018, 2: 0.00015, 3: 0.00021, 4: 0.00019,	AZOBR_150062	1: 0.00012, 2: 0.00011, 3: 0.00021, 4: 0.00019, 5: 0.00019,	1: 0.00031, 2: 0.00018, 3: 0.00038,
AZOLI_2973	1: 0.00493, 2: 0.00820, 3: 0.00041, 4: 0.00425,		AZOBR_150063	5: 0.00017,	1: 0.00011, 2: 0.00024,

AZOLI_2980		2: 0.00007, 4: 0.00013,	AZOBR_150065		
AZOLI_2982	2: 0.00075, 3: 0.00047, 4: 0.00078,	1: 0.00100, 2: 0.00105, 3: 0.00142, 4: 0.00078,	AZOBR_150072	1: 0.00020, 3: 0.00013,	
AZOLI_2983	1: 0.00067, 2: 0.00027, 4: 0.00046,	1: 0.00055, 2: 0.00053, 3: 0.00072, 4: 0.00047,	AZOBR_150074	5: 0.00014,	3: 0.00005,
AZOLI_2985	1: 0.00009, 2: 0.00023, 4: 0.00016,		AZOBR_150075	3: 0.00013, 4: 0.00010, 5: 0.00007,	
AZOLI_2987		2: 0.00006, 4: 0.00006,	AZOBR_150077		
AZOLI_2988	4: 0.00022,	4: 0.00011,	AZOBR_150079	1: 0.00037, 2: 0.00030, 3: 0.00026, 4: 0.00037, 5: 0.00030,	1: 0.00019, 2: 0.00015, 3: 0.00019,
AZOLI_2990	1: 0.00131, 2: 0.00141, 3: 0.00275, 4: 0.00206,	1: 0.00149, 2: 0.00164, 3: 0.00217, 4: 0.00176,	AZOBR_150080	1: 0.00011, 2: 0.00006, 3: 0.00006, 4: 0.00010, 5: 0.00007,	3: 0.00007,
AZOLI_2991	1: 0.00027, 2: 0.00051, 3: 0.00021, 4: 0.00061,	1: 0.00024, 2: 0.00032, 3: 0.00006, 4: 0.00020,	AZOBR_150084	3: 0.00016, 4: 0.00006,	
AZOLI_2994	1: 0.00008,		AZOBR_150085		
AZOLI_2995	4: 0.00022,		AZOBR_150088	1: 0.00026, 2: 0.00009, 3: 0.00022, 4: 0.00007,	3: 0.00006,
AZOLI_2997	1: 0.00006, 2: 0.00016, 4: 0.00008,		AZOBR_150090	1: 0.00032, 2: 0.00057, 3: 0.00035, 4: 0.00014, 5: 0.00022,	1: 0.00039, 2: 0.00047, 3: 0.00040,
AZOLI_2999	4: 0.00014,	1: 0.00011, 3: 0.00024, 4: 0.00019,	AZOBR_150091	4: 0.00011,	1: 0.00010, 3: 0.00014,
AZOLI_3000	4: 0.00023,		AZOBR_150093	5: 0.00011,	1: 0.00015, 3: 0.00028,
AZOLI_3004	4: 0.00017,		AZOBR_150094		
AZOLI_3008		2: 0.00009, 4: 0.00006,	AZOBR_150096		
AZOLI_3014	1: 0.00054, 2: 0.00030, 3: 0.00021, 4: 0.00039,	1: 0.00005, 2: 0.00007, 3: 0.00005, 4: 0.00008,	AZOBR_150100		3: 0.00009,
AZOLI_3015	1: 0.00080, 2: 0.00053, 4: 0.00053,	1: 0.00024, 2: 0.00037, 3: 0.00015, 4: 0.00020,	AZOBR_150105		2: 0.00009, 3: 0.00020,
AZOLI_3016	1: 0.00040, 2: 0.00023, 3: 0.00032, 4: 0.00049,	1: 0.00029, 2: 0.00011, 3: 0.00018, 4: 0.00029,	AZOBR_150107	1: 0.00048, 2: 0.00063, 3: 0.00034, 4: 0.00049, 5: 0.00022,	1: 0.00036, 2: 0.00023, 3: 0.00015,
AZOLI_3019	1: 0.00022, 2: 0.00023, 3: 0.00047, 4: 0.00052,	1: 0.00015, 2: 0.00029, 3: 0.00008, 4: 0.00026,	AZOBR_150110	1: 0.00124, 2: 0.00254, 3: 0.00198, 4: 0.00203, 5: 0.00211,	1: 0.00175, 2: 0.00138, 3: 0.00161,
AZOLI_3024		1: 0.00057, 4: 0.00049,	AZOBR_150112	1: 0.00063, 2: 0.00056, 3: 0.00043, 4: 0.00104, 5: 0.00052,	1: 0.00194, 2: 0.00230, 3: 0.00133,
AZOLI_3025	1: 0.00095, 2: 0.00083,	1: 0.00097, 2: 0.00120, 3: 0.00132, 4: 0.00082,	AZOBR_150114		
AZOLI_3026	4: 0.00003,	4: 0.00003,	AZOBR_150126	1: 0.00034, 3: 0.00015, 4: 0.00047, 5: 0.00018,	1: 0.00011, 2: 0.00012, 3: 0.00032,
AZOLI_3027	1: 0.00027,		AZOBR_150128	1: 0.00024, 2: 0.00026, 3: 0.00026, 4: 0.00034, 5: 0.00026,	2: 0.00008, 3: 0.00014,
AZOLI_3028	1: 0.00007, 2: 0.00014, 4: 0.00012,	1: 0.00025, 2: 0.00007, 3: 0.00015, 4: 0.00009,	AZOBR_150129	3: 0.00006,	
AZOLI_3031	1: 0.00008, 3: 0.00016,		AZOBR_150130	1: 0.00180, 2: 0.00184, 3: 0.00194, 4: 0.00216, 5: 0.00222,	1: 0.00176, 2: 0.00124, 3: 0.00165,
AZOLI_3032		1: 0.00011,	AZOBR_150132	1: 0.00171, 2: 0.00105, 3: 0.00145, 4: 0.00112, 5: 0.00128,	1: 0.00026, 2: 0.00054, 3: 0.00042,
AZOLI_3033	1: 0.00032,	1: 0.00012, 2: 0.00028, 3: 0.00013, 4: 0.00014,	AZOBR_150133		
AZOLI_3035	1: 0.00035, 2: 0.00016, 4: 0.00022,	1: 0.00010, 2: 0.00028, 4: 0.00014,	AZOBR_150137	3: 0.00006, 4: 0.00022, 5: 0.00007,	1: 0.00011, 2: 0.00005, 3: 0.00020,
AZOLI_3036		4: 0.00005,	AZOBR_150139	1: 0.00026, 2: 0.00023, 3: 0.00033, 4: 0.00035, 5: 0.00044,	1: 0.00041, 2: 0.00019, 3: 0.00056,
AZOLI_3038	1: 0.00092, 2: 0.00052, 3: 0.00038, 4: 0.00072,	1: 0.00055, 2: 0.00048, 3: 0.00035, 4: 0.00068,	AZOBR_150140	1: 0.00014, 2: 0.00033, 3: 0.00032, 4: 0.00035, 5: 0.00029,	3: 0.00023,
AZOLI_3039	1: 0.00056, 2: 0.00042, 4: 0.00054,	1: 0.00042, 2: 0.00061, 3: 0.00030, 4: 0.00036,	AZOBR_150146	1: 0.00057, 4: 0.00026, 5: 0.00033,	2: 0.00041, 3: 0.00012,
AZOLI_3042	4: 0.00022,	3: 0.00016, 4: 0.00009,	AZOBR_150147	4: 0.00006, 5: 0.00006,	3: 0.00008,
AZOLI_3044	1: 0.00018, 2: 0.00018, 3: 0.00014, 4: 0.00035,	2: 0.00010, 3: 0.00014, 4: 0.00007,	AZOBR_150149		2: 0.00013,
AZOLI_3045	4: 0.00026,		AZOBR_150150	1: 0.00103, 2: 0.00123, 3: 0.00064, 4: 0.00054, 5: 0.00066,	1: 0.00030, 2: 0.00055, 3: 0.00060,
AZOLI_3048	1: 0.00026, 2: 0.00021, 4: 0.00018,	1: 0.00016, 2: 0.00040, 3: 0.00028,	AZOBR_150151		1: 0.00037, 3: 0.00029,
AZOLI_3049	1: 0.00058, 2: 0.00032, 4: 0.00059,	1: 0.00011, 4: 0.00014,	AZOBR_150153	5: 0.00016,	
AZOLI_3050		4: 0.00011,	AZOBR_150156		
AZOLI_3051	1: 0.00018, 3: 0.00039, 4: 0.00016,	1: 0.00042, 2: 0.00022, 3: 0.00020, 4: 0.00047,	AZOBR_150158	2: 0.00048, 3: 0.00047, 4: 0.00028,	1: 0.00018, 2: 0.00150, 3: 0.00026,
AZOLI_3052	1: 0.00013, 2: 0.00026, 3: 0.00060, 4: 0.00026,	1: 0.00022, 2: 0.00025, 3: 0.00023, 4: 0.00030,	AZOBR_150160		1: 0.00017, 2: 0.00004, 3: 0.00005,
AZOLI_3053	4: 0.00033,		AZOBR_150164	1: 0.00048, 2: 0.00014, 3: 0.00027,	1: 0.00015, 2: 0.00012, 3: 0.00010,
AZOLI_3054	1: 0.00074, 2: 0.00122, 3: 0.00222, 4: 0.00112,	1: 0.00179, 2: 0.00154, 3: 0.00139, 4: 0.00152,	AZOBR_150169	2: 0.00018, 3: 0.00013, 4: 0.00018, 5: 0.00007,	
AZOLI_3067	1: 0.00009, 2: 0.00028, 4: 0.00016,	1: 0.00024, 3: 0.00015, 4: 0.00016,	AZOBR_150171	1: 0.00005, 2: 0.00022, 3: 0.00015, 4: 0.00038, 5: 0.00028,	1: 0.00017, 2: 0.00011, 3: 0.00033,
AZOLI_3068	1: 0.00130, 2: 0.00140, 3: 0.00127, 4: 0.00168,	1: 0.00132, 2: 0.00135, 3: 0.00126, 4: 0.00140,	AZOBR_150173	1: 0.00065, 2: 0.00061, 3: 0.00062, 4: 0.00067, 5: 0.00067,	1: 0.00018, 2: 0.00032, 3: 0.00048,
AZOLI_3070	1: 0.00470, 2: 0.00693, 3: 0.00770, 4: 0.00373,	1: 0.00413, 2: 0.00335, 3: 0.00314, 4: 0.00406,	AZOBR_150176		
AZOLI_3071	1: 0.00018, 2: 0.00018, 4: 0.00044,	3: 0.00012, 4: 0.00012,	AZOBR_150180	1: 0.00073, 4: 0.00030, 5: 0.00020,	1: 0.00019, 2: 0.00032,
AZOLI_3072	1: 0.00046, 2: 0.00029, 4: 0.00064,	1: 0.00012, 2: 0.00028, 3: 0.00012, 4: 0.00010,	AZOBR_150191	3: 0.00003,	1: 0.00002, 2: 0.00002, 3: 0.00003,
AZOLI_3075	1: 0.00041, 2: 0.00048, 3: 0.00067, 4: 0.00128,	1: 0.00123, 2: 0.00204, 3: 0.00152, 4: 0.00117,	AZOBR_150194	5: 0.00008,	1: 0.00008, 2: 0.00009, 3: 0.00011,
AZOLI_3075	1: 0.00082, 2: 0.00107, 3: 0.00133, 4: 0.00086,	1: 0.00063, 2: 0.00083, 3: 0.00065, 4: 0.00073,	AZOBR_150195		
AZOLI_3076	1: 0.00043, 2: 0.00026, 4: 0.00055,	1: 0.00035, 2: 0.00033, 3: 0.00041, 4: 0.00033,	AZOBR_150196	1: 0.00009, 4: 0.00013, 5: 0.00010,	1: 0.00009, 3: 0.00014,

AZOLI_3077	1: 0.00133, 2: 0.00119, 3: 0.00089, 4: 0.00146,	1: 0.00093, 2: 0.00070, 3: 0.00091, 4: 0.00126,	AZOBR_150197		1: 0.00012,
AZOLI_3078	1: 0.00020, 2: 0.00034, 4: 0.00065,	1: 0.00028, 2: 0.00053, 4: 0.00035,	AZOBR_150203	4: 0.00008,	
AZOLI_3081	1: 0.00302, 2: 0.00314, 3: 0.00467, 4: 0.00328,	1: 0.00147, 2: 0.00159, 3: 0.00159, 4: 0.00168,	AZOBR_150206		
AZOLI_3082	1: 0.00462, 2: 0.00501, 3: 0.00253, 4: 0.00440,	1: 0.00322, 2: 0.00162, 3: 0.00237, 4: 0.00179,	AZOBR_150207	2: 0.00028, 4: 0.00014, 5: 0.00036,	1: 0.00020, 2: 0.00046, 3: 0.00045,
AZOLI_3084	1: 0.00043, 2: 0.00018, 3: 0.00025, 4: 0.00012,	1: 0.00047, 2: 0.00042, 3: 0.00027, 4: 0.00025,	AZOBR_150208	1: 0.00021, 2: 0.00019, 3: 0.00018, 4: 0.00022, 5: 0.00022,	1: 0.00027, 3: 0.00026,
AZOLI_3085	4: 0.00012,		AZOBR_150213	4: 0.00008, 5: 0.00008,	
AZOLI_3086	1: 0.00006, 2: 0.00006, 3: 0.00030, 4: 0.00019,	2: 0.00012, 3: 0.00016, 4: 0.00019,	AZOBR_150215		
AZOLI_3087	1: 0.00005, 4: 0.00004,	1: 0.00012, 2: 0.00012, 4: 0.00008,	AZOBR_150224	1: 0.01177, 2: 0.01213, 3: 0.01169, 4: 0.01094, 5: 0.00920,	1: 0.00666, 2: 0.00500, 3: 0.00488,
AZOLI_3089	1: 0.00017, 2: 0.00018, 4: 0.00015,	1: 0.00018, 2: 0.00042, 3: 0.00028, 4: 0.00015,	AZOBR_150226		
AZOLI_3091	1: 0.00056, 2: 0.00071, 4: 0.00026,	1: 0.00020, 2: 0.00020, 4: 0.00017,	AZOBR_150228		
AZOLI_3092		4: 0.00012,	AZOBR_150229	1: 0.00017, 2: 0.00012, 3: 0.00014, 4: 0.00011, 5: 0.00007,	1: 0.00006, 2: 0.00012, 3: 0.00006,
AZOLI_3096		4: 0.00025,	AZOBR_150233	1: 0.00016, 2: 0.00009, 3: 0.00027, 4: 0.00030, 5: 0.00011,	2: 0.00008, 3: 0.00027,
AZOLI_3099	2: 0.00012, 3: 0.00100, 4: 0.00038,	1: 0.00028, 2: 0.00019, 4: 0.00021,	AZOBR_150234	1: 0.00074, 2: 0.00197, 3: 0.00092, 4: 0.00098, 5: 0.00084,	1: 0.00229, 2: 0.00236, 3: 0.00176,
AZOLI_3101	1: 0.00171, 2: 0.00128, 3: 0.00063, 4: 0.00112,	1: 0.00094, 2: 0.00083, 3: 0.00084, 4: 0.00071,	AZOBR_150236	1: 0.00775, 2: 0.00589, 3: 0.00553, 4: 0.00503, 5: 0.00553,	1: 0.00416, 2: 0.00466, 3: 0.00347,
AZOLI_3106	2: 0.00026, 3: 0.00081, 4: 0.00022,	1: 0.00085, 2: 0.00082, 3: 0.00055, 4: 0.00050,	AZOBR_150237	1: 0.00576, 2: 0.00326, 3: 0.00412, 4: 0.00714, 5: 0.00413,	1: 0.00369, 2: 0.00403, 3: 0.00458,
AZOLI_3107	2: 0.00048, 3: 0.00050, 4: 0.00067,	1: 0.00018, 3: 0.00032, 4: 0.00026,	AZOBR_150238	1: 0.00445, 2: 0.00434, 3: 0.00478, 4: 0.00443, 5: 0.00678,	1: 0.00660, 2: 0.00546, 3: 0.00553,
AZOLI_3109	1: 0.00065, 2: 0.00104, 4: 0.00121,	1: 0.00038, 2: 0.00018, 4: 0.00065,	AZOBR_150239	1: 0.00774, 2: 0.00855, 3: 0.01102, 4: 0.00900, 5: 0.00701,	1: 0.00426, 2: 0.00308, 3: 0.00511,
AZOLI_3110	1: 0.00109, 2: 0.00154, 3: 0.00431, 4: 0.00231,	1: 0.00172, 2: 0.00160, 3: 0.00182, 4: 0.00170,	AZOBR_150240	1: 0.00631, 2: 0.00717, 3: 0.00670, 4: 0.00768, 5: 0.00622,	1: 0.00615, 2: 0.00496, 3: 0.00540,
AZOLI_3111		1: 0.00010, 3: 0.00014, 4: 0.00006,	AZOBR_150242	1: 0.00188, 2: 0.00464, 3: 0.00256, 4: 0.00577, 5: 0.00466,	1: 0.00297, 2: 0.00173, 3: 0.00334,
AZOLI_3112	4: 0.00008,	2: 0.00018, 3: 0.00010, 4: 0.00012,	AZOBR_150243	1: 0.00316, 2: 0.00292, 3: 0.00269, 4: 0.00178, 5: 0.00357,	1: 0.00239, 2: 0.00178, 3: 0.00188,
AZOLI_3118	2: 0.00092,		AZOBR_150244	1: 0.00314, 2: 0.00612, 3: 0.00481, 4: 0.00583, 5: 0.00590,	1: 0.00470, 2: 0.00417, 3: 0.00501,
AZOLI_3120	1: 0.00014, 2: 0.00038, 3: 0.00043, 4: 0.00044,	1: 0.00066, 2: 0.00047, 3: 0.00077, 4: 0.00041,	AZOBR_150245	1: 0.00511, 2: 0.00464, 3: 0.00541, 4: 0.00866, 5: 0.00543,	1: 0.00345, 2: 0.00342, 3: 0.00498,
AZOLI_3121	1: 0.00029, 2: 0.00014, 3: 0.00010, 4: 0.00022,	1: 0.00022, 2: 0.00028, 3: 0.00008, 4: 0.00012,	AZOBR_150246	1: 0.00571, 2: 0.00494, 3: 0.00740, 4: 0.00361, 5: 0.00287,	1: 0.00335, 2: 0.00217, 3: 0.00288,
AZOLI_3122	1: 0.00020, 2: 0.00020, 3: 0.00023, 4: 0.00021,	1: 0.00037, 2: 0.00008, 3: 0.00022, 4: 0.00010,	AZOBR_150247	1: 0.01279, 2: 0.01282, 3: 0.01825, 4: 0.01012, 5: 0.01085,	1: 0.00609, 2: 0.00604, 3: 0.00709,
AZOLI_3124	2: 0.00012, 4: 0.00007,	1: 0.00023, 4: 0.00017,	AZOBR_150248	1: 0.00320, 2: 0.00754, 3: 0.00309, 4: 0.00500, 5: 0.00559,	1: 0.00492, 2: 0.00249, 3: 0.00318,
AZOLI_3128	1: 0.00174, 2: 0.00155, 3: 0.00063, 4: 0.00136,	1: 0.00340, 2: 0.00288, 3: 0.00321, 4: 0.00346,	AZOBR_150249	1: 0.00457, 2: 0.00475, 3: 0.00441, 4: 0.00224, 5: 0.00448,	1: 0.00286, 2: 0.00303, 3: 0.00227,
AZOLI_3129		4: 0.00015,	AZOBR_150250	1: 0.00343, 2: 0.00393, 3: 0.00369, 4: 0.00294, 5: 0.00392,	1: 0.00227, 2: 0.00283, 3: 0.00199,
AZOLI_3133	1: 0.00020,		AZOBR_150251	1: 0.00942, 2: 0.01165, 3: 0.00944, 4: 0.00916, 5: 0.00955,	1: 0.00689, 2: 0.00847, 3: 0.00468,
AZOLI_3135	4: 0.00010,		AZOBR_150252	1: 0.01341, 2: 0.00989, 3: 0.01317, 4: 0.00652, 5: 0.00990,	1: 0.00799, 2: 0.00862, 3: 0.00686,
AZOLI_3153	1: 0.00019, 2: 0.00025, 3: 0.00027, 4: 0.00035,	1: 0.00039, 2: 0.00044, 3: 0.00040, 4: 0.00031,	AZOBR_150253	1: 0.00565, 2: 0.00536, 3: 0.00414, 4: 0.00458, 5: 0.00459,	1: 0.00308, 2: 0.00246, 3: 0.00226,
AZOLI_3154		3: 0.00009, 4: 0.00005,	AZOBR_150254	1: 0.01659, 2: 0.01624, 3: 0.01342, 4: 0.00771, 5: 0.00992,	1: 0.00319, 2: 0.00665, 3: 0.00412,
AZOLI_3155	1: 0.00075, 2: 0.00061, 3: 0.00034, 4: 0.00067,	1: 0.00067, 2: 0.00047, 3: 0.00078, 4: 0.00083,	AZOBR_150255	1: 0.01092, 2: 0.00834, 3: 0.00864, 4: 0.00975, 5: 0.01050,	1: 0.00912, 2: 0.00780, 3: 0.00661,
AZOLI_3158	1: 0.00020, 2: 0.00021, 4: 0.00056,	1: 0.00050, 2: 0.00040, 3: 0.00039, 4: 0.00018,	AZOBR_150256	1: 0.01205, 2: 0.00782, 3: 0.00995, 4: 0.00801, 5: 0.00908,	1: 0.00418, 2: 0.00421, 3: 0.00344,
AZOLI_3159	1: 0.00359, 2: 0.00406, 3: 0.00194, 4: 0.00428,	1: 0.00172, 2: 0.00145, 3: 0.00113, 4: 0.00146,	AZOBR_150257	1: 0.00171, 2: 0.00390, 3: 0.00424, 4: 0.00480, 5: 0.00697,	1: 0.00228, 2: 0.00404, 3: 0.00212,
AZOLI_3164	1: 0.00023, 3: 0.00026, 4: 0.00012,	1: 0.00019, 2: 0.00049, 3: 0.00010, 4: 0.00020,	AZOBR_160002	4: 0.00003, 5: 0.00004,	1: 0.00005, 3: 0.00009,
AZOLI_3166	1: 0.00028, 2: 0.00014, 4: 0.00012,	1: 0.00022, 2: 0.00021, 3: 0.00015, 4: 0.00012,	AZOBR_160003	1: 0.00428, 2: 0.00698, 3: 0.00618, 4: 0.00455, 5: 0.00353,	1: 0.00310, 2: 0.00339, 3: 0.00272,
AZOLI_3167		1: 0.00049, 4: 0.00021,	AZOBR_160004	1: 0.01274, 2: 0.00980, 3: 0.01276, 4: 0.00853, 5: 0.01001,	1: 0.00204, 2: 0.00370, 3: 0.00249,
AZOLI_3168	1: 0.00007, 2: 0.00017,	1: 0.00013, 3: 0.00011, 4: 0.00011,	AZOBR_160005		
AZOLI_3169	1: 0.00022, 2: 0.00029, 3: 0.00022, 4: 0.00044,	1: 0.00016, 2: 0.00026, 3: 0.00026, 4: 0.00019,	AZOBR_160006	1: 0.00034, 2: 0.00048, 3: 0.00041, 4: 0.00046, 5: 0.00045,	1: 0.00024, 2: 0.00022, 3: 0.00036,
AZOLI_3174	1: 0.00062, 2: 0.00042, 3: 0.00039, 4: 0.00024,	1: 0.00063, 2: 0.00040, 3: 0.00067, 4: 0.00036,	AZOBR_160007	1: 0.00044, 2: 0.00023, 3: 0.00035, 4: 0.00044, 5: 0.00037,	1: 0.00033, 2: 0.00038, 3: 0.00050,
AZOLI_3178	1: 0.00088, 2: 0.00119, 3: 0.00401, 4: 0.00072,	1: 0.00169, 2: 0.00197, 3: 0.00245, 4: 0.00273,	AZOBR_160009	1: 0.02435, 2: 0.01975, 3: 0.01695, 4: 0.01580, 5: 0.01914,	1: 0.02175, 2: 0.02055, 3: 0.02270,
AZOLI_p10011	1: 0.00020, 2: 0.00027, 4: 0.00024,		AZOBR_160010	1: 0.01595, 2: 0.01304, 3: 0.01099, 4: 0.00680, 5: 0.00827,	1: 0.01049, 2: 0.01174, 3: 0.00472,
AZOLI_p10015	1: 0.00048, 2: 0.00072, 3: 0.00127, 4: 0.00061,	1: 0.00115, 2: 0.00141, 3: 0.00119, 4: 0.00114,	AZOBR_160011	1: 0.00573, 2: 0.00605, 3: 0.00584, 4: 0.00622, 5: 0.00872,	1: 0.00467, 2: 0.00304, 3: 0.00403,
AZOLI_p10018	1: 0.00020, 2: 0.00020,		AZOBR_160012	1: 0.00907, 2: 0.00828, 3: 0.00532, 4: 0.00896, 5: 0.01098,	1: 0.00413, 2: 0.00565, 3: 0.00495,
AZOLI_p10024		4: 0.00007,	AZOBR_160013	1: 0.00487, 2: 0.00284, 3: 0.00421, 4: 0.00222, 5: 0.00233,	1: 0.00042, 2: 0.00282, 3: 0.00103,
AZOLI_p10025		2: 0.00003, 4: 0.00003,	AZOBR_160015	1: 0.01081, 2: 0.01340, 3: 0.01233,	1: 0.00787, 2: 0.00790,

				4: 0.01002, 5: 0.01135,	3: 0.00649,
AZOLI_p10026		1: 0.00018, 3: 0.00009, 4: 0.00019,	AZOBR_160015	1: 0.01081, 2: 0.01340, 3: 0.01233, 4: 0.01002, 5: 0.01135,	1: 0.00787, 2: 0.00790, 3: 0.00649,
AZOLI_p10027		1: 0.00006, 3: 0.00005, 4: 0.00005,	AZOBR_160017	2: 0.00014, 4: 0.00007,	1: 0.00029, 2: 0.00007, 3: 0.00016,
AZOLI_p10028	4: 0.00005,		AZOBR_160024	2: 0.00011, 4: 0.00008, 5: 0.00013,	
AZOLI_p10029	1: 0.00006, 3: 0.00036,	1: 0.00044, 2: 0.00037, 3: 0.00053, 4: 0.00042,	AZOBR_160033		
AZOLI_p10030		2: 0.00008, 3: 0.00009, 4: 0.00005,	AZOBR_160036		
AZOLI_p10035	1: 0.00034, 3: 0.00048,		AZOBR_160038		1: 0.00115, 2: 0.00087, 3: 0.00093,
AZOLI_p10036	4: 0.00002,		AZOBR_160039	1: 0.00042, 2: 0.00056, 3: 0.00042, 4: 0.00072, 5: 0.00046,	1: 0.00103, 2: 0.00077, 3: 0.00076,
AZOLI_p10043	1: 0.00020, 2: 0.00013, 4: 0.00040,	3: 0.00007, 4: 0.00006,	AZOBR_160051		
AZOLI_p10045	1: 0.00004, 3: 0.00002, 4: 0.00002,	3: 0.00004, 4: 0.00002,	AZOBR_160052		
AZOLI_p10053	1: 0.00023, 2: 0.00010,	2: 0.00029, 3: 0.00032, 4: 0.00014,	AZOBR_160053		
AZOLI_p10054	1: 0.00012, 2: 0.00040, 4: 0.00031,	2: 0.00008, 4: 0.00014,	AZOBR_160054		2: 0.00009,
AZOLI_p10066	1: 0.00041, 2: 0.00064, 4: 0.00026,	1: 0.00034, 2: 0.00011, 3: 0.00024, 4: 0.00010,	AZOBR_160056		
AZOLI_p10074	1: 0.00007,		AZOBR_160057		1: 0.00150, 2: 0.00161, 3: 0.00150,
AZOLI_p10075	1: 0.00008, 2: 0.00005, 4: 0.00013,	1: 0.00092, 2: 0.00081, 3: 0.00100, 4: 0.00103,	AZOBR_160063		2: 0.00006,
AZOLI_p10078		4: 0.00012,	AZOBR_160066		
AZOLI_p10079		3: 0.00016, 4: 0.00021,	AZOBR_160068		
AZOLI_p10082	1: 0.00008, 2: 0.00008, 4: 0.00020,		AZOBR_160070	1: 0.00051, 4: 0.00058, 5: 0.00070,	1: 0.00043, 2: 0.00037, 3: 0.00063,
AZOLI_p10086	1: 0.00066, 2: 0.00071, 4: 0.00091,	1: 0.00049, 2: 0.00064, 3: 0.00099, 4: 0.00057,	AZOBR_160073		2: 0.00023, 3: 0.00019,
AZOLI_p10093	1: 0.00727, 2: 0.01083, 3: 0.00766, 4: 0.00665,	1: 0.00745, 2: 0.00631, 3: 0.00654, 4: 0.00539,	AZOBR_160075		
AZOLI_p10094	1: 0.00426, 2: 0.00296, 3: 0.00521, 4: 0.00421,	1: 0.00404, 2: 0.00361, 3: 0.00292, 4: 0.00434,	AZOBR_160076		
AZOLI_p10095	1: 0.00070, 2: 0.00054, 3: 0.00009, 4: 0.00022,	1: 0.00067, 2: 0.00065, 3: 0.00051, 4: 0.00025,	AZOBR_160078	1: 0.00102, 2: 0.00079, 3: 0.00084, 4: 0.00082, 5: 0.00080,	1: 0.00175, 2: 0.00137, 3: 0.00101,
AZOLI_p10097	1: 0.00496, 2: 0.00523, 3: 0.00188, 4: 0.00274,	1: 0.00232, 2: 0.00169, 3: 0.00209, 4: 0.00101,	AZOBR_160079		1: 0.00036,
AZOLI_p10102	4: 0.00004,		AZOBR_170001	1: 0.00068, 2: 0.00036, 3: 0.00105, 4: 0.00113, 5: 0.00097,	1: 0.00058, 2: 0.00053, 3: 0.00074,
AZOLI_p10154	4: 0.00003,	4: 0.00003,	AZOBR_170004		
AZOLI_p10155	4: 0.00002,	1: 0.00002, 3: 0.00002, 4: 0.00003,	AZOBR_170005	4: 0.00007, 5: 0.00005,	
AZOLI_p10156	1: 0.00018, 2: 0.00017, 3: 0.00014, 4: 0.00021,	1: 0.00020, 2: 0.00032, 3: 0.00036, 4: 0.00024,	AZOBR_170006	1: 0.00060, 2: 0.00053, 3: 0.00048, 4: 0.00064, 5: 0.00065,	1: 0.00065, 2: 0.00050, 3: 0.00072,
AZOLI_p10189	4: 0.00003,		AZOBR_170007		
AZOLI_p10197	3: 0.00022,	1: 0.00013, 2: 0.00021, 3: 0.00031, 4: 0.00018,	AZOBR_170009	1: 0.00202, 2: 0.00174, 3: 0.00217, 4: 0.00147, 5: 0.00183,	1: 0.00130, 2: 0.00173, 3: 0.00183,
AZOLI_p10199		1: 0.00017, 2: 0.00008, 3: 0.00006, 4: 0.00010,	AZOBR_170010		
AZOLI_p10200	1: 0.00027, 2: 0.00094, 3: 0.00062, 4: 0.00052,	2: 0.00027, 3: 0.00023, 4: 0.00033,	AZOBR_170011	1: 0.00008, 4: 0.00010, 5: 0.00005,	1: 0.00010, 2: 0.00006,
AZOLI_p10214	4: 0.00008,		AZOBR_180002	3: 0.00006, 4: 0.00005,	3: 0.00007,
AZOLI_p10227	2: 0.00019,	1: 0.00121, 2: 0.00108, 3: 0.00099, 4: 0.00087,	AZOBR_180003	4: 0.00004, 5: 0.00007,	3: 0.00004,
AZOLI_p10232	4: 0.00012,	1: 0.00018, 2: 0.00007, 4: 0.00027,	AZOBR_180004		2: 0.00020, 3: 0.00017,
AZOLI_p10247	1: 0.00005,	1: 0.00011, 2: 0.00005, 3: 0.00012, 4: 0.00007,	AZOBR_180007		
AZOLI_p10251	1: 0.00004,		AZOBR_180009	1: 0.00009, 4: 0.00006, 5: 0.00008,	1: 0.00004, 2: 0.00006,
AZOLI_p10253		1: 0.00023, 2: 0.00014, 3: 0.00029, 4: 0.00016,	AZOBR_180012	1: 0.00058, 2: 0.00034, 3: 0.00033, 4: 0.00038, 5: 0.00040,	1: 0.00043, 2: 0.00047, 3: 0.00048,
AZOLI_p10253		1: 0.00024, 2: 0.00023, 4: 0.00016,	AZOBR_180013		
AZOLI_p10260	1: 0.00006, 2: 0.00006, 3: 0.00023, 4: 0.00011,	1: 0.00036, 2: 0.00038, 3: 0.00028, 4: 0.00027,	AZOBR_180015		1: 0.00010, 2: 0.00016, 3: 0.00047,
AZOLI_p10265	1: 0.00103, 2: 0.00046, 3: 0.00036, 4: 0.00039,	1: 0.00079, 2: 0.00158, 3: 0.00090, 4: 0.00084,	AZOBR_180019		1: 0.00008,
AZOLI_p10267		2: 0.00030, 3: 0.00013, 4: 0.00024,	AZOBR_180020		
AZOLI_p10268	1: 0.00178, 2: 0.00196, 3: 0.00405, 4: 0.00220,	1: 0.00189, 2: 0.00169, 3: 0.00214, 4: 0.00251,	AZOBR_180022	3: 0.00016,	
AZOLI_p10269	1: 0.00039, 2: 0.00048, 3: 0.00037, 4: 0.00019,	1: 0.00049, 2: 0.00030, 3: 0.00051, 4: 0.00030,	AZOBR_180025		
AZOLI_p10271	4: 0.00045,	1: 0.00008, 2: 0.00016, 3: 0.00022, 4: 0.00014,	AZOBR_180027		1: 0.00012, 3: 0.00012,
AZOLI_p10276	1: 0.00021, 2: 0.00019, 3: 0.00122, 4: 0.00038,	1: 0.00062, 2: 0.00078, 3: 0.00096, 4: 0.00083,	AZOBR_180029		1: 0.00030, 3: 0.00025,
AZOLI_p10279	3: 0.00047,		AZOBR_180030	1: 0.00029, 2: 0.00017, 3: 0.00028, 4: 0.00056, 5: 0.00031,	1: 0.00017, 2: 0.00009, 3: 0.00010,
AZOLI_p10286	4: 0.00030,		AZOBR_180031	1: 0.00058, 2: 0.00129, 3: 0.00133, 4: 0.00094, 5: 0.00094,	1: 0.00062, 2: 0.00021, 3: 0.00036,
AZOLI_p10290	1: 0.00025, 2: 0.00020, 4: 0.00021,	1: 0.00065, 2: 0.00048, 3: 0.00021, 4: 0.00064,	AZOBR_180034		
AZOLI_p10303	1: 0.00171, 2: 0.00078, 3: 0.00078, 4: 0.00131,	1: 0.00061, 2: 0.00073, 3: 0.00071, 4: 0.00059,	AZOBR_180036	4: 0.00083, 5: 0.00033,	

AZOLI_p10305	4: 0.00011,	4: 0.00004,	AZOBR_180037	1: 0.00016, 2: 0.00003, 3: 0.00015, 4: 0.00011, 5: 0.00009,	1: 0.00023, 2: 0.00017, 3: 0.00030,
AZOLI_p10306	3: 0.00028, 4: 0.00023,	2: 0.00019, 4: 0.00029,	AZOBR_180039	1: 0.00158, 2: 0.00177, 3: 0.00155, 4: 0.00151, 5: 0.00159,	1: 0.00016, 3: 0.00023,
AZOLI_p10309	1: 0.00049, 2: 0.00069, 4: 0.00025,	1: 0.00049, 2: 0.00114, 3: 0.00021, 4: 0.00051,	AZOBR_180043		2: 0.00005, 3: 0.00004,
AZOLI_p10311	4: 0.00003,		AZOBR_180063	1: 0.00015, 2: 0.00014, 3: 0.00020, 4: 0.00009, 5: 0.00012,	1: 0.00013, 2: 0.00007, 3: 0.00017,
AZOLI_p10320	1: 0.00011, 3: 0.00080, 4: 0.00049,	1: 0.00023, 2: 0.00036, 3: 0.00021, 4: 0.00054,	AZOBR_180064	1: 0.00010, 3: 0.00011, 4: 0.00024, 5: 0.00009,	1: 0.00004, 2: 0.00009, 3: 0.00012,
AZOLI_p10321	1: 0.00096, 2: 0.00158, 3: 0.00727, 4: 0.00105,	1: 0.00221, 2: 0.00258, 3: 0.00217, 4: 0.00231,	AZOBR_180068		
AZOLI_p10325		1: 0.00005, 4: 0.00004,	AZOBR_180069		
AZOLI_p10331	1: 0.00027, 2: 0.00036, 4: 0.00061,	1: 0.00090, 2: 0.00069, 3: 0.00028, 4: 0.00107,	AZOBR_180071	1: 0.00043, 2: 0.00023, 3: 0.00033, 4: 0.00038, 5: 0.00030,	1: 0.00006, 2: 0.00006, 3: 0.00011,
AZOLI_p10333	1: 0.00021, 2: 0.00017, 4: 0.00007,		AZOBR_180074	4: 0.00015, 5: 0.00023,	1: 0.00043, 3: 0.00028,
AZOLI_p10337		1: 0.00004, 4: 0.00003,	AZOBR_180079	1: 0.00248, 2: 0.00422, 3: 0.00276, 4: 0.00158, 5: 0.00290,	1: 0.00477, 2: 0.00445, 3: 0.00439,
AZOLI_p10340	1: 0.00053, 2: 0.00039, 3: 0.00027, 4: 0.00028,	1: 0.00101, 2: 0.00066, 3: 0.00068, 4: 0.00077,	AZOBR_180080	1: 0.00088, 2: 0.00111, 3: 0.00128, 4: 0.00146, 5: 0.00086,	1: 0.00032, 2: 0.00064, 3: 0.00039,
AZOLI_p10345	2: 0.00014, 4: 0.00009,	2: 0.00007,	AZOBR_180081	2: 0.00008, 3: 0.00005, 4: 0.00021, 5: 0.00017,	
AZOLI_p10347	1: 0.00035, 2: 0.00162, 3: 0.00138, 4: 0.00138,	1: 0.00156, 2: 0.00082, 3: 0.00105, 4: 0.00127,	AZOBR_180083		2: 0.00002,
AZOLI_p10352	2: 0.00014, 3: 0.00009, 4: 0.00009,	1: 0.00010, 3: 0.00018, 4: 0.00023,	AZOBR_180085	2: 0.00010, 3: 0.00010, 4: 0.00008,	
AZOLI_p10355	1: 0.00005, 2: 0.00005, 4: 0.00021,	1: 0.00025, 2: 0.00013, 3: 0.00020, 4: 0.00012,	AZOBR_180086	1: 0.00012, 2: 0.00008, 3: 0.00010, 4: 0.00010, 5: 0.00003,	1: 0.00003, 2: 0.00009, 3: 0.00007,
AZOLI_p10367	4: 0.00005,		AZOBR_180090		1: 0.00011, 3: 0.00011,
AZOLI_p10368	4: 0.00010,	4: 0.00013,	AZOBR_180091	1: 0.00022, 2: 0.00011, 3: 0.00029, 4: 0.00009, 5: 0.00009,	1: 0.00014, 2: 0.00016, 3: 0.00032,
AZOLI_p10369	1: 0.00059, 2: 0.00025, 4: 0.00021,	1: 0.00028, 2: 0.00021, 3: 0.00030, 4: 0.00019,	AZOBR_180094	1: 0.00018, 2: 0.00013, 3: 0.00018, 4: 0.00014, 5: 0.00006,	1: 0.00004,
AZOLI_p10376	1: 0.00067, 2: 0.00082, 3: 0.00014, 4: 0.00093,	1: 0.00260, 2: 0.00187, 3: 0.00242, 4: 0.00175,	AZOBR_180095	1: 0.00015, 3: 0.00012, 4: 0.00004,	1: 0.00004, 2: 0.00013, 3: 0.00009,
AZOLI_p10386	1: 0.00010, 2: 0.00010, 4: 0.00017,	1: 0.00007, 2: 0.00019, 3: 0.00021, 4: 0.00011,	AZOBR_180097	3: 0.00005,	2: 0.00005,
AZOLI_p10388	1: 0.00015, 2: 0.00012, 4: 0.00026,	2: 0.00009, 4: 0.00008,	AZOBR_180099		1: 0.00024, 2: 0.00013, 3: 0.00027,
AZOLI_p10389	1: 0.00084, 2: 0.00063, 3: 0.00141, 4: 0.00105,	1: 0.00083, 2: 0.00104, 3: 0.00076, 4: 0.00152,	AZOBR_180101	3: 0.00044, 5: 0.00035,	
AZOLI_p10390	3: 0.00152, 4: 0.00074,	1: 0.00044, 2: 0.00074, 3: 0.00081, 4: 0.00084,	AZOBR_180103	1: 0.00015, 2: 0.00005, 3: 0.00008, 5: 0.00009,	1: 0.00010, 2: 0.00020, 3: 0.00023,
AZOLI_p10391	3: 0.00022,	1: 0.00129, 2: 0.00161, 3: 0.00122, 4: 0.00159,	AZOBR_180104	1: 0.00057, 2: 0.00008, 3: 0.00020, 4: 0.00019, 5: 0.00033,	1: 0.00009, 2: 0.00010, 3: 0.00021,
AZOLI_p10392		1: 0.00007, 2: 0.00010, 4: 0.00009,	AZOBR_180105	1: 0.00009, 2: 0.00028, 3: 0.00027, 4: 0.00025, 5: 0.00013,	1: 0.00015, 2: 0.00023, 3: 0.00014,
AZOLI_p10413	1: 0.00011, 2: 0.00024, 4: 0.00003,	1: 0.00013, 2: 0.00020, 3: 0.00008, 4: 0.00005,	AZOBR_180107	4: 0.00010,	2: 0.00005, 3: 0.00006,
AZOLI_p10414	1: 0.00013, 2: 0.00023, 4: 0.00033,	1: 0.00010, 2: 0.00007, 3: 0.00008,	AZOBR_180108	1: 0.00076, 2: 0.00050, 3: 0.00077, 4: 0.00050, 5: 0.00084,	1: 0.00027, 2: 0.00034, 3: 0.00047,
AZOLI_p10421	1: 0.00032, 2: 0.00032, 4: 0.00018,	1: 0.00043, 2: 0.00052, 3: 0.00057, 4: 0.00028,	AZOBR_180110		
AZOLI_p10480	1: 0.00015, 2: 0.00016,		AZOBR_180111		1: 0.00030, 3: 0.00059,
AZOLI_p10482	1: 0.00066, 2: 0.00074, 3: 0.00024, 4: 0.00082,	1: 0.00052, 2: 0.00036, 3: 0.00024, 4: 0.00051,	AZOBR_180112	4: 0.00004,	3: 0.00002,
AZOLI_p10487	1: 0.00015, 2: 0.00015, 4: 0.00015,	4: 0.00006,	AZOBR_180116	1: 0.00010, 2: 0.00007, 3: 0.00016, 4: 0.00009, 5: 0.00007,	3: 0.00005,
AZOLI_p10489	1: 0.00018, 4: 0.00010,	2: 0.00012,	AZOBR_180117		
AZOLI_p10490	4: 0.00008,		AZOBR_180118	2: 0.00026, 3: 0.00050, 4: 0.00024, 5: 0.00016,	3: 0.00026,
AZOLI_p10490	2: 0.00028,	1: 0.00022, 2: 0.00011, 4: 0.00009,	AZOBR_180119	1: 0.00019, 4: 0.00023, 5: 0.00010,	1: 0.00005, 2: 0.00005, 3: 0.00005,
AZOLI_p10491	1: 0.00021, 2: 0.00021, 4: 0.00021,	1: 0.00021, 2: 0.00016, 3: 0.00022, 4: 0.00021,	AZOBR_180120	1: 0.00300, 2: 0.00477, 3: 0.00398, 4: 0.00372, 5: 0.00563,	1: 0.00430, 2: 0.00341, 3: 0.00440,
AZOLI_p10498	1: 0.00081, 2: 0.00109, 3: 0.00030, 4: 0.00098,	1: 0.00058, 2: 0.00050, 3: 0.00061, 4: 0.00036,	AZOBR_180121		
AZOLI_p10502	1: 0.00015, 2: 0.00021, 4: 0.00020,	1: 0.00005, 2: 0.00005, 3: 0.00006, 4: 0.00011,	AZOBR_180123	1: 0.00021, 2: 0.00015, 3: 0.00021, 4: 0.00020, 5: 0.00017,	1: 0.00016, 2: 0.00027, 3: 0.00026,
AZOLI_p10512	3: 0.00011,	1: 0.00095, 2: 0.00147, 3: 0.00127, 4: 0.00169,	AZOBR_180127	3: 0.00005, 4: 0.00003,	
AZOLI_p10518		4: 0.00005,	AZOBR_180128		
AZOLI_p10528		4: 0.00005,	AZOBR_180129		3: 0.00012,
AZOLI_p10532	1: 0.00503, 2: 0.00484, 3: 0.00062, 4: 0.00346,	1: 0.00226, 2: 0.00221, 3: 0.00170, 4: 0.00169,	AZOBR_180136	4: 0.00005,	
AZOLI_p10535	2: 0.00007, 4: 0.00020,	1: 0.00013, 2: 0.00016, 3: 0.00014, 4: 0.00025,	AZOBR_180137		1: 0.00186, 2: 0.00211, 3: 0.00193,
AZOLI_p10566	1: 0.00008, 2: 0.00011, 4: 0.00024,	1: 0.00018, 2: 0.00013, 3: 0.00014, 4: 0.00011,	AZOBR_180140		1: 0.00016, 3: 0.00031,
AZOLI_p10572	1: 0.00003,		AZOBR_180141		3: 0.00016,
AZOLI_p10573		1: 0.00008,	AZOBR_180142	1: 0.00027, 2: 0.00013, 3: 0.00020, 4: 0.00054, 5: 0.00044,	1: 0.00048, 2: 0.00039, 3: 0.00075,
AZOLI_p10574		2: 0.00031, 4: 0.00054,	AZOBR_180146		
AZOLI_p10576	4: 0.00006,	3: 0.00007,	AZOBR_180152		
AZOLI_p10581	1: 0.00008,		AZOBR_180154	3: 0.00008, 4: 0.00017, 5: 0.00010,	
AZOLI_p10584	1: 0.00027, 2: 0.00046, 3: 0.00118, 4: 0.00063,	1: 0.00124, 2: 0.00112, 3: 0.00163, 4: 0.00174,	AZOBR_180157	4: 0.00011,	
AZOLI_p10585	1: 0.00046, 2: 0.00039,	1: 0.00039, 2: 0.00060,	AZOBR_180160		2: 0.00006,
AZOLI_p10587		1: 0.00204, 2: 0.00049,	AZOBR_180162		1: 0.00011, 2: 0.00012,

		3: 0.00054, 4: 0.00087,			3: 0.00041,
AZOLI_p10590	1: 0.00003, 2: 0.00004,	1: 0.00010, 2: 0.00003, 3: 0.00002, 4: 0.00006,	AZOBR_180164	4: 0.00006,	
AZOLI_p10591	2: 0.00014, 4: 0.00007,	2: 0.00005, 3: 0.00012, 4: 0.00009,	AZOBR_180166		1: 0.00040, 2: 0.00023, 3: 0.00039,
AZOLI_p10592		4: 0.00006,	AZOBR_180172	4: 0.00029,	1: 0.00359, 2: 0.00347, 3: 0.00735,
AZOLI_p10596	1: 0.00033, 2: 0.00013, 4: 0.00036,	1: 0.00021, 2: 0.00016, 4: 0.00011,	AZOBR_180173		1: 0.00042, 3: 0.00104,
AZOLI_p10599	1: 0.00087, 2: 0.00041, 3: 0.00009, 4: 0.00043,		AZOBR_180179		
AZOLI_p10602	1: 0.00004,	2: 0.00005, 4: 0.00009,	AZOBR_180180		1: 0.00011,
AZOLI_p10605	2: 0.00020,		AZOBR_180181		
AZOLI_p10611	1: 0.00077, 2: 0.00112, 3: 0.00075, 4: 0.00141,	1: 0.00039, 2: 0.00014, 3: 0.00067, 4: 0.00054,	AZOBR_180182		2: 0.00004,
AZOLI_p10630	2: 0.00008,		AZOBR_180183		1: 0.00009, 2: 0.00012, 3: 0.00015,
AZOLI_p10633	1: 0.00041, 2: 0.00043, 3: 0.00087, 4: 0.00086,	1: 0.00067, 2: 0.00078, 3: 0.00071, 4: 0.00072,	AZOBR_180184		1: 0.00030, 2: 0.00029, 3: 0.00037,
AZOLI_p10634	2: 0.00003,	4: 0.00004,	AZOBR_180191	4: 0.00015, 5: 0.00016,	
AZOLI_p10637	4: 0.00008,	2: 0.00006, 4: 0.00005,	AZOBR_180192	4: 0.00011,	
AZOLI_p10638	4: 0.00012,		AZOBR_180193		
AZOLI_p10643	1: 0.00021, 2: 0.00027, 4: 0.00023,	1: 0.00019, 2: 0.00015, 3: 0.00016, 4: 0.00013,	AZOBR_180199	1: 0.00028, 3: 0.00018, 4: 0.00024, 5: 0.00034,	1: 0.00013, 3: 0.00030,
AZOLI_p10647	1: 0.00014,	2: 0.00009, 4: 0.00012,	AZOBR_180203		1: 0.00172, 2: 0.00089, 3: 0.00118,
AZOLI_p10655	1: 0.00022, 3: 0.00008, 4: 0.00007,	1: 0.00020, 2: 0.00033, 3: 0.00030, 4: 0.00031,	AZOBR_180204		
AZOLI_p10656	1: 0.00021, 2: 0.00054,		AZOBR_180206		1: 0.00006, 3: 0.00014,
AZOLI_p10663	1: 0.00029, 2: 0.00030, 4: 0.00022,	1: 0.00019, 2: 0.00013, 3: 0.00008, 4: 0.00012,	AZOBR_180208		
AZOLI_p10668	1: 0.00017, 2: 0.00038, 3: 0.00030, 4: 0.00065,	1: 0.00021, 3: 0.00018, 4: 0.00015,	AZOBR_180211	1: 0.00007, 4: 0.00003,	1: 0.00019, 2: 0.00036, 3: 0.00020,
AZOLI_p10676	1: 0.00006, 2: 0.00006, 4: 0.00010,	1: 0.00029, 2: 0.00020, 3: 0.00040, 4: 0.00032,	AZOBR_180212		3: 0.00006,
AZOLI_p10680	2: 0.00025,	1: 0.00025, 2: 0.00036, 4: 0.00021,	AZOBR_180213		1: 0.00014, 2: 0.00027, 3: 0.00013,
AZOLI_p10684		1: 0.00013, 3: 0.00009, 4: 0.00011,	AZOBR_180214		
AZOLI_p10719	3: 0.00038,		AZOBR_180215		
AZOLI_p10720	3: 0.00008,	1: 0.00009, 2: 0.00012, 3: 0.00010, 4: 0.00008,	AZOBR_180216		3: 0.00075,
AZOLI_p10723	3: 0.00033,	1: 0.00031, 3: 0.00067, 4: 0.00027,	AZOBR_180217		1: 0.00014, 2: 0.00016, 3: 0.00027,
AZOLI_p10752	4: 0.00002,	1: 0.00003, 2: 0.00002, 3: 0.00006, 4: 0.00002,	AZOBR_180218		1: 0.00026, 2: 0.00030, 3: 0.00048,
AZOLI_p10762	1: 0.00023, 2: 0.00040, 3: 0.00014, 4: 0.00057,	1: 0.00047, 2: 0.00049, 3: 0.00048, 4: 0.00040,	AZOBR_180221		
AZOLI_p10765	4: 0.00074,		AZOBR_180225		1: 0.00022, 2: 0.00031, 3: 0.00032,
AZOLI_p10770	2: 0.00015,		AZOBR_180226		1: 0.00030, 2: 0.00019, 3: 0.00027,
AZOLI_p10771	1: 0.00004, 2: 0.00014, 3: 0.00035, 4: 0.00020,	1: 0.00049, 2: 0.00043, 3: 0.00052, 4: 0.00037,	AZOBR_180227	1: 0.00022, 2: 0.00028, 3: 0.00027,	1: 0.00028, 2: 0.00055, 3: 0.00013,
AZOLI_p10773	4: 0.00041,		AZOBR_180228		1: 0.00014, 3: 0.00028,
AZOLI_p10774	1: 0.00018, 4: 0.00011,		AZOBR_180232	1: 0.00013, 4: 0.00013,	
AZOLI_p10801	1: 0.00031, 2: 0.00031,	1: 0.00016, 2: 0.00023, 4: 0.00013,	AZOBR_180235		1: 0.00009, 2: 0.00020, 3: 0.00013,
AZOLI_p10802		1: 0.00011, 3: 0.00024,	AZOBR_180240		1: 0.00026, 2: 0.00017, 3: 0.00015,
AZOLI_p10806	1: 0.00003, 2: 0.00003,	1: 0.00005, 3: 0.00003, 4: 0.00010,	AZOBR_180241		
AZOLI_p10807	1: 0.00243, 2: 0.00208, 3: 0.00974, 4: 0.00226,	1: 0.00626, 2: 0.00612, 3: 0.00823, 4: 0.00743,	AZOBR_180242	1: 0.00005, 3: 0.00013, 4: 0.00005,	2: 0.00004,
AZOLI_p10810	1: 0.00052, 2: 0.00033, 4: 0.00034,	1: 0.00033, 2: 0.00045, 3: 0.00035, 4: 0.00011,	AZOBR_180247	2: 0.00009,	3: 0.00007,
AZOLI_p10811	2: 0.00017,	2: 0.00016,	AZOBR_180250	1: 0.00020, 2: 0.00033, 3: 0.00022, 4: 0.00018, 5: 0.00020,	1: 0.00022, 2: 0.00020, 3: 0.00030,
AZOLI_p10813	1: 0.00017,	2: 0.00025,	AZOBR_180251	1: 0.00067, 2: 0.00025, 3: 0.00057, 4: 0.00118, 5: 0.00105,	1: 0.00110, 2: 0.00104, 3: 0.00184,
AZOLI_p10815	4: 0.00008,		AZOBR_180253	4: 0.00016,	2: 0.00014, 3: 0.00012,
AZOLI_p10820	1: 0.00103, 2: 0.00148, 3: 0.00169, 4: 0.00117,	1: 0.00132, 2: 0.00165, 3: 0.00158, 4: 0.00127,	AZOBR_180254	1: 0.00032, 2: 0.00021, 3: 0.00027, 4: 0.00027, 5: 0.00060,	1: 0.00030, 2: 0.00029, 3: 0.00044,
AZOLI_p10840		4: 0.00001,	AZOBR_180255	1: 0.00013, 2: 0.00008,	
AZOLI_p10850		1: 0.00008, 4: 0.00006,	AZOBR_180256	4: 0.00014, 5: 0.00009,	1: 0.00009, 2: 0.00010, 3: 0.00021,
AZOLI_p10867		1: 0.00019, 2: 0.00007, 3: 0.00029, 4: 0.00020,	AZOBR_180258	1: 0.00012, 2: 0.00011, 4: 0.00019, 5: 0.00011,	1: 0.00008, 2: 0.00017,
AZOLI_p10870		1: 0.00012, 2: 0.00012,	AZOBR_180260	2: 0.00023, 3: 0.00030, 4: 0.00012, 5: 0.00012,	1: 0.00151, 2: 0.00070, 3: 0.00060,
AZOLI_p10873		2: 0.00003,	AZOBR_180261		1: 0.00017, 2: 0.00032,
AZOLI_p10885		4: 0.00009,	AZOBR_180262		
AZOLI_p10886		3: 0.00014, 4: 0.00017,	AZOBR_180263	4: 0.00003,	1: 0.00041, 2: 0.00033, 3: 0.00064,
AZOLI_p10930	1: 0.00031, 2: 0.00057,	1: 0.00010, 2: 0.00010,	AZOBR_180265	1: 0.00297, 2: 0.00179, 3: 0.00240, 4: 0.00177, 5: 0.00217,	1: 0.00174, 2: 0.00216, 3: 0.00160,
AZOLI_p10931	4: 0.00013,		AZOBR_180266	1: 0.00082, 2: 0.00135, 3: 0.00104, 4: 0.00120, 5: 0.00134,	1: 0.00196, 2: 0.00104, 3: 0.00159,
AZOLI_p11028	1: 0.00063, 2: 0.00029, 4: 0.00020,	1: 0.00058, 2: 0.00047, 3: 0.00052, 4: 0.00034,	AZOBR_180269	2: 0.00049, 5: 0.00051,	2: 0.00080, 3: 0.00057,

AZOLI_p20005		1: 0.00015, 2: 0.00014, 3: 0.00016, 4: 0.00017,	AZOBR_180271		3: 0.00005,
AZOLI_p20008		2: 0.00007, 3: 0.00005,	AZOBR_180272		2: 0.00004, 3: 0.00007,
AZOLI_p20040	1: 0.00023, 4: 0.00020,	1: 0.00023, 3: 0.00061, 4: 0.00069,	AZOBR_180273		
AZOLI_p20161	4: 0.00001,		AZOBR_180274		3: 0.00004,
AZOLI_p20180		3: 0.00007,	AZOBR_180275	1: 0.00003,	1: 0.00002, 3: 0.00002,
AZOLI_p20216	1: 0.00013, 4: 0.00011,		AZOBR_180276	1: 0.00003,	1: 0.00005, 2: 0.00002, 3: 0.00002,
AZOLI_p20222	1: 0.00058, 2: 0.00038, 3: 0.00010, 4: 0.00065,	1: 0.00041, 2: 0.00033, 3: 0.00026, 4: 0.00035,	AZOBR_180277	4: 0.00014, 5: 0.00006,	1: 0.00016, 2: 0.00006, 3: 0.00020,
AZOLI_p20223	1: 0.00009, 4: 0.00008,		AZOBR_180278	1: 0.00013, 2: 0.00008, 3: 0.00006, 4: 0.00026, 5: 0.00020,	1: 0.00035, 2: 0.00029, 3: 0.00037,
AZOLI_p20224	1: 0.00035, 2: 0.00013, 4: 0.00038,	1: 0.00018, 3: 0.00028, 4: 0.00011,	AZOBR_180281	4: 0.00008, 5: 0.00008,	1: 0.00005, 2: 0.00014, 3: 0.00010,
AZOLI_p20229	4: 0.00008,	4: 0.00008,	AZOBR_180282	4: 0.00030,	1: 0.00014, 3: 0.00013,
AZOLI_p20232		4: 0.00007,	AZOBR_180283	4: 0.00024, 5: 0.00018,	3: 0.00017,
AZOLI_p20236	3: 0.00015, 4: 0.00006,	1: 0.00032, 2: 0.00041, 3: 0.00037, 4: 0.00018,	AZOBR_180290		2: 0.00006,
AZOLI_p20249	4: 0.00011,		AZOBR_180300		3: 0.00010,
AZOLI_p20272		2: 0.00019,	AZOBR_180320		
AZOLI_p20275		1: 0.00049, 2: 0.00054, 3: 0.00066, 4: 0.00059,	AZOBR_190005		
AZOLI_p20283	2: 0.00007, 4: 0.00006,		AZOBR_190013		
AZOLI_p20288	1: 0.00004, 2: 0.00009, 3: 0.00016,	1: 0.00049, 2: 0.00028, 3: 0.00038, 4: 0.00023,	AZOBR_190023		3: 0.00005,
AZOLI_p20296		4: 0.00011,	AZOBR_200002		2: 0.00004, 3: 0.00003,
AZOLI_p20307	2: 0.00018,		AZOBR_200014		
AZOLI_p20311	2: 0.00004,	1: 0.00004, 4: 0.00004,	AZOBR_200015		
AZOLI_p20319	1: 0.00022,	1: 0.00042, 2: 0.00044, 3: 0.00028, 4: 0.00035,	AZOBR_200026	4: 0.00011,	
AZOLI_p20320	1: 0.00050, 2: 0.00063, 3: 0.00070, 4: 0.00032,	1: 0.00152, 2: 0.00134, 3: 0.00081, 4: 0.00092,	AZOBR_200027		
AZOLI_p20329	1: 0.00009, 4: 0.00011,	2: 0.00021, 3: 0.00018, 4: 0.00011,	AZOBR_200028	3: 0.00017, 4: 0.00017, 5: 0.00007,	1: 0.00016, 2: 0.00015, 3: 0.00006,
AZOLI_p20332	1: 0.00013, 2: 0.00015, 3: 0.00006, 4: 0.00030,	1: 0.00004, 2: 0.00006, 3: 0.00007, 4: 0.00011,	AZOBR_200029		
AZOLI_p20335		2: 0.00013, 4: 0.00011,	AZOBR_200030	3: 0.00007, 4: 0.00005,	3: 0.00012,
AZOLI_p20336		4: 0.00004,	AZOBR_200031		2: 0.00010,
AZOLI_p20337	4: 0.00029,		AZOBR_200034	1: 0.00020, 3: 0.00026, 4: 0.00083, 5: 0.00076,	1: 0.00019, 3: 0.00106,
AZOLI_p20340	1: 0.00031, 4: 0.00014,	2: 0.00023, 3: 0.00017, 4: 0.00027,	AZOBR_200035	2: 0.00006, 3: 0.00004, 4: 0.00009, 5: 0.00005,	1: 0.00003, 3: 0.00004,
AZOLI_p20346		1: 0.00015, 2: 0.00007, 3: 0.00023, 4: 0.00028,	AZOBR_200038		1: 0.00010, 3: 0.00018,
AZOLI_p20349		4: 0.00035,	AZOBR_200039		3: 0.00015,
AZOLI_p20353	1: 0.00014, 3: 0.00019,		AZOBR_200072		
AZOLI_p20361	1: 0.00004, 2: 0.00004,		AZOBR_200075		2: 0.00031,
AZOLI_p20366	1: 0.00006,		AZOBR_200083	1: 0.00089, 2: 0.00109, 3: 0.00090, 4: 0.00122, 5: 0.00110,	
AZOLI_p20367	1: 0.00011, 2: 0.00011,		AZOBR_200128	2: 0.00012, 4: 0.00014, 5: 0.00014,	1: 0.00018, 2: 0.00015, 3: 0.00021,
AZOLI_p20370	1: 0.00053, 2: 0.00046, 4: 0.00020,		AZOBR_200130		1: 0.00010, 3: 0.00023,
AZOLI_p20381		2: 0.00012, 3: 0.00005, 4: 0.00011,	AZOBR_200131		1: 0.00022, 2: 0.00010, 3: 0.00008,
AZOLI_p20404		4: 0.00006,	AZOBR_200133	1: 0.00051, 2: 0.00030, 3: 0.00053, 4: 0.00054, 5: 0.00047,	1: 0.00025, 2: 0.00102, 3: 0.00049,
AZOLI_p20409		4: 0.00007,	AZOBR_200135	1: 0.00141, 2: 0.00174, 3: 0.00202, 4: 0.00108, 5: 0.00066,	1: 0.00077, 2: 0.00092, 3: 0.00047,
AZOLI_p20418	1: 0.00039, 2: 0.00019, 3: 0.00217, 4: 0.00022,	1: 0.00052, 2: 0.00056, 3: 0.00041, 4: 0.00011,	AZOBR_200138	1: 0.00167, 2: 0.00059, 3: 0.00043, 4: 0.00034,	1: 0.00032, 2: 0.00109,
AZOLI_p20424		3: 0.00013, 4: 0.00008,	AZOBR_200139	1: 0.00034, 2: 0.00042, 3: 0.00051, 4: 0.00032, 5: 0.00021,	1: 0.00014, 2: 0.00016, 3: 0.00005,
AZOLI_p20433		4: 0.00006,	AZOBR_200141	1: 0.00054,	2: 0.00026, 3: 0.00017,
AZOLI_p20459	1: 0.00170, 2: 0.00185, 3: 0.00322, 4: 0.00204,	1: 0.00181, 2: 0.00146, 3: 0.00203, 4: 0.00223,	AZOBR_200143		3: 0.00013,
AZOLI_p20460	3: 0.00007,		AZOBR_200145		1: 0.00017, 2: 0.00006, 3: 0.00016,
AZOLI_p20464	1: 0.00220, 2: 0.00238, 3: 0.00483, 4: 0.00327,	1: 0.00486, 2: 0.00471, 3: 0.00496, 4: 0.00457,	AZOBR_200151		1: 0.00015, 3: 0.00003,
AZOLI_p20471		1: 0.00032, 2: 0.00040, 3: 0.00020, 4: 0.00019,	AZOBR_200156		1: 0.00020, 2: 0.00008, 3: 0.00040,
AZOLI_p20478	1: 0.00014, 4: 0.00009,		AZOBR_200158	1: 0.00014, 3: 0.00014,	1: 0.00004, 2: 0.00004,
AZOLI_p20517	4: 0.00002,		AZOBR_200160		
AZOLI_p20523	1: 0.00056, 2: 0.00092, 3: 0.00079, 4: 0.00124,	1: 0.00137, 2: 0.00148, 3: 0.00137, 4: 0.00145,	AZOBR_200161	2: 0.00015, 5: 0.00018,	1: 0.00073, 2: 0.00019, 3: 0.00168,
AZOLI_p20526	3: 0.00010,		AZOBR_200162	1: 0.00437, 2: 0.00223, 3: 0.00353, 4: 0.00304, 5: 0.00292,	1: 0.00560, 2: 0.00683, 3: 0.00364,
AZOLI_p20528		4: 0.00005,	AZOBR_200163	1: 0.00091, 2: 0.00097, 3: 0.00161, 4: 0.00143, 5: 0.00169,	1: 0.00147, 2: 0.00117, 3: 0.00161,
AZOLI_p20529		1: 0.00064, 2: 0.00110, 3: 0.00113, 4: 0.00042,	AZOBR_200164	1: 0.00130, 2: 0.00154, 3: 0.00127, 4: 0.00161, 5: 0.00172,	1: 0.00259, 2: 0.00240, 3: 0.00270,
AZOLI_p20530		1: 0.00013, 2: 0.00025, 4: 0.00011,	AZOBR_200166	1: 0.00003, 2: 0.00016, 3: 0.00016, 4: 0.00016, 5: 0.00013,	1: 0.00008, 2: 0.00015, 3: 0.00016,
AZOLI_p20536	3: 0.00008,	1: 0.00044, 2: 0.00039, 3: 0.00057, 4: 0.00039,	AZOBR_200167	1: 0.00115, 2: 0.00074, 3: 0.00107, 4: 0.00151, 5: 0.00187,	1: 0.00083, 2: 0.00071, 3: 0.00106,
AZOLI_p20537		1: 0.00096, 2: 0.00072, 4: 0.00055,	AZOBR_200168		
AZOLI_p20543		3: 0.00014,	AZOBR_200172	2: 0.00007, 3: 0.00006, 4: 0.00013,	3: 0.00005,

				5: 0.00013,	
AZOLI_p20547	1: 0.00016, 2: 0.00018, 3: 0.00009, 4: 0.00014,		AZOBR_200174	1: 0.00038, 2: 0.00027, 3: 0.00026, 4: 0.00057, 5: 0.00047,	2: 0.00017, 3: 0.00028,
AZOLI_p20548	1: 0.00011, 4: 0.00015,		AZOBR_200175		
AZOLI_p20556	4: 0.00018,		AZOBR_200176	4: 0.00007, 5: 0.00007,	1: 0.00019, 2: 0.00022, 3: 0.00019,
AZOLI_p20567		1: 0.00016, 2: 0.00018, 3: 0.00017, 4: 0.00014,	AZOBR_200178		
AZOLI_p20572	4: 0.00008,	1: 0.00014, 3: 0.00010,	AZOBR_200179	1: 0.00047, 2: 0.00052, 3: 0.00057, 4: 0.00027, 5: 0.00043,	1: 0.00055, 2: 0.00060, 3: 0.00066,
AZOLI_p20573	3: 0.00043, 4: 0.00018,	1: 0.00041, 2: 0.00025, 3: 0.00027, 4: 0.00026,	AZOBR_200181	1: 0.00093, 2: 0.00272, 3: 0.00170, 4: 0.00231, 5: 0.00247,	1: 0.00206, 2: 0.00151, 3: 0.00116,
AZOLI_p20577	3: 0.00016,	1: 0.00027, 2: 0.00033, 3: 0.00020, 4: 0.00026,	AZOBR_200186	1: 0.00010, 2: 0.00014, 3: 0.00009, 4: 0.00014,	1: 0.00030, 3: 0.00032,
AZOLI_p20578	1: 0.00008, 2: 0.00020, 3: 0.00065, 4: 0.00008,	1: 0.00074, 2: 0.00074, 3: 0.00070, 4: 0.00072,	AZOBR_200187	5: 0.00005,	
AZOLI_p20580	1: 0.00016, 4: 0.00007,	2: 0.00011,	AZOBR_200188	1: 0.00022, 2: 0.00032, 3: 0.00046, 4: 0.00027, 5: 0.00025,	1: 0.00005, 2: 0.00008, 3: 0.00009,
AZOLI_p20587	1: 0.00014,	1: 0.00049, 2: 0.00040, 3: 0.00044, 4: 0.00039,	AZOBR_200189		
AZOLI_p20590	2: 0.00010, 4: 0.00012,		AZOBR_200193		
AZOLI_p20595	3: 0.00030,		AZOBR_200194		
AZOLI_p20603	3: 0.00034,		AZOBR_200195		1: 0.00067, 2: 0.00046, 3: 0.00046,
AZOLI_p20620	3: 0.00024,		AZOBR_200196		
AZOLI_p20627		1: 0.00028, 4: 0.00024,	AZOBR_200199	3: 0.00036,	1: 0.00045, 2: 0.00062, 3: 0.00035,
AZOLI_p20638	1: 0.00016, 2: 0.00010, 4: 0.00031,	1: 0.00026, 2: 0.00029, 3: 0.00042, 4: 0.00025,	AZOBR_200200	1: 0.00108, 2: 0.00085, 3: 0.00112, 4: 0.00058, 5: 0.00058,	1: 0.00061, 2: 0.00035, 3: 0.00045,
AZOLI_p20643	2: 0.00011, 4: 0.00010,	2: 0.00014, 3: 0.00016, 4: 0.00016,	AZOBR_200202		1: 0.00009, 3: 0.00008,
AZOLI_p20656	1: 0.00016, 4: 0.00023,		AZOBR_200203		
AZOLI_p20658	1: 0.00013, 4: 0.00007,	4: 0.00007,	AZOBR_200204	4: 0.00004,	1: 0.00003, 2: 0.00006, 3: 0.00013,
AZOLI_p20662		4: 0.00006,	AZOBR_200206	3: 0.00012,	
AZOLI_p20681		2: 0.00004,	AZOBR_200207		3: 0.00015,
AZOLI_p20685	3: 0.00019, 4: 0.00008,		AZOBR_200208	3: 0.00006, 4: 0.00010, 5: 0.00008,	3: 0.00009,
AZOLI_p20688		1: 0.00015, 2: 0.00011, 3: 0.00020, 4: 0.00014,	AZOBR_200209		3: 0.00063,
AZOLI_p20691	1: 0.00009, 2: 0.00019,	4: 0.00008,	AZOBR_200210		
AZOLI_p20692	1: 0.00008, 4: 0.00009,		AZOBR_200212		
AZOLI_p20700	4: 0.00008,	1: 0.00033, 2: 0.00016, 3: 0.00028, 4: 0.00025,	AZOBR_200220		
AZOLI_p20707	1: 0.00146, 2: 0.00082, 3: 0.00057, 4: 0.00136,	2: 0.00028, 4: 0.00010,	AZOBR_200223	4: 0.00038, 5: 0.00013,	3: 0.00017,
AZOLI_p20715		2: 0.00006, 4: 0.00005,	AZOBR_200225	1: 0.00067, 2: 0.00038, 3: 0.00052, 4: 0.00046, 5: 0.00061,	1: 0.00006, 2: 0.00004, 3: 0.00015,
AZOLI_p20731	1: 0.00019, 2: 0.00018, 3: 0.00010, 4: 0.00014,	1: 0.00037, 2: 0.00037, 4: 0.00003,	AZOBR_200226		2: 0.00013,
AZOLI_p20742	1: 0.00011, 2: 0.00008, 4: 0.00013,	1: 0.00023, 2: 0.00015, 3: 0.00008, 4: 0.00010,	AZOBR_p110001	1: 0.00050, 2: 0.00040, 3: 0.00038, 4: 0.00042, 5: 0.00027,	1: 0.00036, 2: 0.00045, 3: 0.00035,
AZOLI_p20743		3: 0.00014,	AZOBR_p110002	1: 0.00268, 2: 0.00240, 3: 0.00258, 4: 0.00172, 5: 0.00224,	1: 0.00166, 2: 0.00196, 3: 0.00155,
AZOLI_p20745	1: 0.00180, 2: 0.00099, 4: 0.00155,		AZOBR_p110003	1: 0.00064, 2: 0.00048, 3: 0.00035, 4: 0.00050, 5: 0.00050,	1: 0.00009, 2: 0.00007, 3: 0.00023,
AZOLI_p20747	1: 0.00027, 2: 0.00011, 4: 0.00037,	3: 0.00017, 4: 0.00014,	AZOBR_p110005	4: 0.00018,	
AZOLI_p30023		2: 0.00018,	AZOBR_p110006	1: 0.00011, 3: 0.00019, 4: 0.00013, 5: 0.00008,	3: 0.00007,
AZOLI_p30032		1: 0.00019, 4: 0.00011,	AZOBR_p110009	3: 0.00006, 4: 0.00017,	2: 0.00008,
AZOLI_p30037		2: 0.00031, 3: 0.00017, 4: 0.00014,	AZOBR_p110010		2: 0.00007,
AZOLI_p30039	3: 0.00098,	1: 0.00071, 2: 0.00118, 3: 0.00102, 4: 0.00099,	AZOBR_p110011		3: 0.00002,
AZOLI_p30044	1: 0.00010, 4: 0.00029,	4: 0.00006,	AZOBR_p110013	4: 0.00006,	1: 0.00004, 2: 0.00007, 3: 0.00025,
AZOLI_p30062		4: 0.00021,	AZOBR_p110016		
AZOLI_p30068		1: 0.00013, 3: 0.00007, 4: 0.00005,	AZOBR_p110017	3: 0.00009, 4: 0.00010,	
AZOLI_p30069		1: 0.00004, 2: 0.00004, 3: 0.00021, 4: 0.00009,	AZOBR_p110018	5: 0.00009,	
AZOLI_p30078	4: 0.00006,		AZOBR_p110019	1: 0.00142, 2: 0.00266, 3: 0.00226, 4: 0.00219, 5: 0.00159,	1: 0.00204, 2: 0.00297, 3: 0.00154,
AZOLI_p30082	2: 0.00006,	1: 0.00010,	AZOBR_p110020	1: 0.00007, 3: 0.00011, 4: 0.00009, 5: 0.00007,	
AZOLI_p30083		4: 0.00011,	AZOBR_p110022		1: 0.00020, 3: 0.00019,
AZOLI_p30092	2: 0.00006,	1: 0.00006, 2: 0.00006, 4: 0.00005,	AZOBR_p110023		2: 0.00017, 3: 0.00019,
AZOLI_p30106	1: 0.00017, 2: 0.00039, 4: 0.00054,	1: 0.00092, 2: 0.00061, 3: 0.00082, 4: 0.00072,	AZOBR_p110024	5: 0.00011,	
AZOLI_p30108	3: 0.00006, 4: 0.00002,	4: 0.00004,	AZOBR_p110027	3: 0.00014, 4: 0.00011,	2: 0.00012, 3: 0.00010,
AZOLI_p30109	1: 0.00034, 2: 0.00039, 3: 0.00018, 4: 0.00052,	1: 0.00083, 2: 0.00084, 3: 0.00111, 4: 0.00089,	AZOBR_p110028		3: 0.00010,
AZOLI_p30114	1: 0.00093, 4: 0.00054,	1: 0.00021, 2: 0.00081, 3: 0.00033, 4: 0.00036,	AZOBR_p110029		
AZOLI_p30115	1: 0.00049, 2: 0.00040, 3: 0.00125, 4: 0.00055,	1: 0.00090, 2: 0.00067, 3: 0.00074, 4: 0.00055,	AZOBR_p110036	1: 0.00013, 2: 0.00010, 3: 0.00017, 4: 0.00039, 5: 0.00032,	1: 0.00005, 2: 0.00011, 3: 0.00011,
AZOLI_p30117		1: 0.00028, 2: 0.00016, 4: 0.00010,	AZOBR_p110045		
AZOLI_p30118		3: 0.00005, 4: 0.00004,	AZOBR_p110048		3: 0.00003,

AZOLI_p30120	1: 0.00080, 2: 0.00070, 4: 0.00057,		AZOBR_p110049	1: 0.00014,	
AZOLI_p30126	1: 0.00004, 2: 0.00014,	1: 0.00018, 2: 0.00020, 3: 0.00010, 4: 0.00008,	AZOBR_p110050	1: 0.00051, 2: 0.00031, 3: 0.00040, 4: 0.00043, 5: 0.00030,	1: 0.00058, 2: 0.00080, 3: 0.00076,
AZOLI_p30127	1: 0.00029, 2: 0.00034, 4: 0.00029,	1: 0.00030, 3: 0.00026, 4: 0.00021,	AZOBR_p110058		3: 0.00003,
AZOLI_p30128	4: 0.00007,		AZOBR_p110066		
AZOLI_p30129	1: 0.00157, 2: 0.00120, 3: 0.00089, 4: 0.00108,	1: 0.00050, 2: 0.00027, 3: 0.00030, 4: 0.00036,	AZOBR_p110069	1: 0.00015, 3: 0.00008,	
AZOLI_p30135		1: 0.00005,	AZOBR_p110070	1: 0.00137, 2: 0.00109, 3: 0.00094, 4: 0.00168, 5: 0.00140,	1: 0.00164, 2: 0.00203, 3: 0.00220,
AZOLI_p30138	1: 0.00016, 2: 0.00035, 4: 0.00039,	1: 0.00035, 2: 0.00023, 3: 0.00023, 4: 0.00023,	AZOBR_p110073		
AZOLI_p30144		1: 0.00015, 2: 0.00033, 3: 0.00026, 4: 0.00018,	AZOBR_p110074	3: 0.00004,	
AZOLI_p30151	1: 0.00158, 2: 0.00160, 3: 0.00105, 4: 0.00161,	1: 0.00329, 2: 0.00305, 3: 0.00312, 4: 0.00320,	AZOBR_p110075		2: 0.00023,
AZOLI_p30154	1: 0.00016, 4: 0.00014,	3: 0.00018,	AZOBR_p110076		
AZOLI_p30165	1: 0.00017, 2: 0.00012, 3: 0.00016, 4: 0.00025,	1: 0.00006, 2: 0.00006, 3: 0.00013, 4: 0.00013,	AZOBR_p110077		2: 0.00006, 3: 0.00003,
AZOLI_p30170	4: 0.00003,		AZOBR_p110079	1: 0.00047, 2: 0.00053, 3: 0.00044, 4: 0.00071, 5: 0.00051,	1: 0.00066, 2: 0.00078, 3: 0.00075,
AZOLI_p30171	1: 0.00004, 4: 0.00003,		AZOBR_p110080	2: 0.00005, 3: 0.00003,	1: 0.00009, 2: 0.00015, 3: 0.00005,
AZOLI_p30175	1: 0.00025, 2: 0.00025, 4: 0.00009,	2: 0.00020, 4: 0.00009,	AZOBR_p110090	1: 0.00077, 4: 0.00015, 5: 0.00030,	3: 0.00021,
AZOLI_p30178		4: 0.00003,	AZOBR_p110101		
AZOLI_p30182	4: 0.00008,		AZOBR_p110103		
AZOLI_p30185	1: 0.00008, 2: 0.00015, 4: 0.00010,	1: 0.00008, 4: 0.00007,	AZOBR_p110104	1: 0.00036, 3: 0.00031,	
AZOLI_p30186	4: 0.00020,		AZOBR_p110105	4: 0.00006, 5: 0.00006,	
AZOLI_p30187	1: 0.00055, 2: 0.00018, 3: 0.00015, 4: 0.00019,	1: 0.00041, 2: 0.00021, 3: 0.00043, 4: 0.00041,	AZOBR_p110106	1: 0.00197, 2: 0.00138, 3: 0.00104, 4: 0.00184, 5: 0.00136,	1: 0.00056, 2: 0.00085, 3: 0.00069,
AZOLI_p30190	1: 0.00027,		AZOBR_p110107	1: 0.00074, 2: 0.00031, 3: 0.00047, 4: 0.00058, 5: 0.00044,	1: 0.00012, 2: 0.00012, 3: 0.00012,
AZOLI_p30194	2: 0.00005, 4: 0.00004,		AZOBR_p110108	1: 0.00021, 2: 0.00009, 3: 0.00021, 4: 0.00019,	
AZOLI_p30197	1: 0.00014, 2: 0.00017, 4: 0.00015,	1: 0.00015, 2: 0.00008, 4: 0.00007,	AZOBR_p110109	1: 0.00028, 2: 0.00025, 3: 0.00016, 4: 0.00045, 5: 0.00039,	1: 0.00012,
AZOLI_p30198	1: 0.00023, 3: 0.00044, 4: 0.00047,	2: 0.00038,	AZOBR_p110110		3: 0.00020,
AZOLI_p30205	1: 0.00058, 2: 0.00135, 4: 0.00066,		AZOBR_p110117	1: 0.00005, 3: 0.00008, 5: 0.00003,	1: 0.00002, 3: 0.00002,
AZOLI_p30206	1: 0.00058, 2: 0.00041, 3: 0.00010, 4: 0.00012,	1: 0.00021, 2: 0.00020, 3: 0.00026, 4: 0.00006,	AZOBR_p110118	1: 0.00173, 2: 0.00366, 3: 0.00177, 4: 0.00190, 5: 0.00326,	1: 0.00694, 2: 0.00915, 3: 0.00515,
AZOLI_p30210		1: 0.00020, 2: 0.00020, 3: 0.00026, 4: 0.00024,	AZOBR_p110121	1: 0.00016, 4: 0.00011,	1: 0.00015,
AZOLI_p30224	1: 0.00028, 2: 0.00028, 4: 0.00007,	1: 0.00008, 2: 0.00016,	AZOBR_p110124	5: 0.00039,	
AZOLI_p30235	1: 0.00034, 2: 0.00029, 4: 0.00050,	1: 0.00020, 2: 0.00056, 3: 0.00036, 4: 0.00025,	AZOBR_p110126		1: 0.00054, 2: 0.00086,
AZOLI_p30238	1: 0.00006, 2: 0.00009, 4: 0.00015,	1: 0.00006, 2: 0.00012, 4: 0.00010,	AZOBR_p110127	3: 0.00009,	1: 0.00055, 2: 0.00081, 3: 0.00038,
AZOLI_p30239	1: 0.00024, 2: 0.00035, 3: 0.00011, 4: 0.00039,	1: 0.00069, 2: 0.00069, 3: 0.00065, 4: 0.00082,	AZOBR_p110128		
AZOLI_p30242	1: 0.00065, 2: 0.00038, 3: 0.00068, 4: 0.00033,	1: 0.00027, 2: 0.00032, 3: 0.00023, 4: 0.00023,	AZOBR_p110131	1: 0.00290, 2: 0.00214, 3: 0.00233, 4: 0.00172, 5: 0.00145,	1: 0.00444, 2: 0.00593, 3: 0.00271,
AZOLI_p30243	1: 0.00012,	2: 0.00008, 4: 0.00011,	AZOBR_p110136		
AZOLI_p30247	1: 0.00016, 2: 0.00013, 3: 0.00035, 4: 0.00028,	1: 0.00037, 2: 0.00026, 3: 0.00029, 4: 0.00049,	AZOBR_p110141		3: 0.00024,
AZOLI_p30253	2: 0.00009, 4: 0.00012,		AZOBR_p110144	1: 0.00010, 2: 0.00011, 3: 0.00014, 4: 0.00013, 5: 0.00022,	1: 0.00006, 2: 0.00012, 3: 0.00020,
AZOLI_p30255	1: 0.00041, 2: 0.00082, 3: 0.00102, 4: 0.00160,	1: 0.00083, 2: 0.00062, 3: 0.00107, 4: 0.00082,	AZOBR_p110145		
AZOLI_p30256	1: 0.00030, 2: 0.00023, 4: 0.00037,	1: 0.00036, 2: 0.00045, 3: 0.00068, 4: 0.00040,	AZOBR_p110146	1: 0.00317, 2: 0.00632, 3: 0.00357, 4: 0.00458, 5: 0.00592,	1: 0.01238, 2: 0.01154, 3: 0.00832,
AZOLI_p30257		4: 0.00005,	AZOBR_p110147		
AZOLI_p30258	1: 0.00015, 2: 0.00022, 4: 0.00019,	3: 0.00024, 4: 0.00013,	AZOBR_p110150		
AZOLI_p30267	1: 0.00011,	4: 0.00010,	AZOBR_p110151	1: 0.00035, 2: 0.00045, 3: 0.00032, 4: 0.00068, 5: 0.00060,	1: 0.00043, 2: 0.00029, 3: 0.00060,
AZOLI_p30268	1: 0.00052, 3: 0.00059, 4: 0.00099,	1: 0.00053, 2: 0.00113, 3: 0.00045, 4: 0.00045,	AZOBR_p120003		1: 0.00026,
AZOLI_p30270	1: 0.00089, 2: 0.00053, 3: 0.00073, 4: 0.00045,	1: 0.00093, 2: 0.00132, 3: 0.00072, 4: 0.00095,	AZOBR_p120003		1: 0.00025,
AZOLI_p30274	1: 0.00014, 2: 0.00010, 3: 0.00010, 4: 0.00030,	1: 0.00056, 2: 0.00054, 3: 0.00063, 4: 0.00051,	AZOBR_p120035		
AZOLI_p30277	2: 0.00009,		AZOBR_p120049	4: 0.00010,	
AZOLI_p30282	1: 0.00019, 2: 0.00030, 3: 0.00184, 4: 0.00007,	1: 0.00146, 2: 0.00114, 3: 0.00180, 4: 0.00175,	AZOBR_p120068	2: 0.00014,	3: 0.00020,
AZOLI_p30282	1: 0.00038, 2: 0.00043, 3: 0.00102, 4: 0.00042,	1: 0.00177, 2: 0.00153, 3: 0.00209, 4: 0.00182,	AZOBR_p120069	1: 0.00005, 2: 0.00011, 3: 0.00013, 4: 0.00016, 5: 0.00007,	1: 0.00002, 2: 0.00008, 3: 0.00004,
AZOLI_p30283		4: 0.00017,	AZOBR_p120080	1: 0.00026, 2: 0.00039, 3: 0.00045, 4: 0.00139, 5: 0.00097,	1: 0.00073, 2: 0.00070, 3: 0.00142,
AZOLI_p30290	3: 0.00033, 4: 0.00003,	1: 0.00027, 2: 0.00023, 3: 0.00040, 4: 0.00019,	AZOBR_p120082	4: 0.00002,	
AZOLI_p30291		4: 0.00007,	AZOBR_p120085		2: 0.00020,
AZOLI_p30297		1: 0.00004, 2: 0.00006, 3: 0.00005, 4: 0.00004,	AZOBR_p120086	1: 0.00006, 2: 0.00011, 3: 0.00011, 4: 0.00013, 5: 0.00011,	3: 0.00008,
AZOLI_p30313	1: 0.00007, 2: 0.00004,	1: 0.00015, 2: 0.00014,	AZOBR_p120088	4: 0.00003,	1: 0.00013, 2: 0.00003,

	3: 0.00010, 4: 0.00008,	3: 0.00018, 4: 0.00017,			3: 0.00008,
AZOLI_p30314		1: 0.00009,	AZOBR_p120089	1: 0.00010, 3: 0.00009, 4: 0.00007, 5: 0.00007,	3: 0.00006,
AZOLI_p30315		1: 0.00051, 2: 0.00067, 3: 0.00049, 4: 0.00051,	AZOBR_p120091		
AZOLI_p30318		1: 0.00070, 2: 0.00085, 3: 0.00114, 4: 0.00070,	AZOBR_p120092		
AZOLI_p30320		4: 0.00007,	AZOBR_p120093	1: 0.00621, 2: 0.00300, 3: 0.00314, 4: 0.00279, 5: 0.00189,	1: 0.00310, 2: 0.00268, 3: 0.00171,
AZOLI_p30323	1: 0.00013,		AZOBR_p120094	1: 0.00023, 2: 0.00021,	3: 0.00022,
AZOLI_p30331	1: 0.00328, 2: 0.00275, 3: 0.00175, 4: 0.00240,	1: 0.00245, 2: 0.00203, 3: 0.00180, 4: 0.00214,	AZOBR_p120095	5: 0.00006,	
AZOLI_p30340	3: 0.00022,	4: 0.00004,	AZOBR_p120096	5: 0.00006,	2: 0.00006, 3: 0.00008,
AZOLI_p30344	1: 0.00242, 2: 0.00234, 3: 0.00068, 4: 0.00153,	1: 0.00104, 2: 0.00100, 3: 0.00052, 4: 0.00061,	AZOBR_p120097	1: 0.00024, 3: 0.00020, 4: 0.00024, 5: 0.00041,	2: 0.00034, 3: 0.00044,
AZOLI_p30350	1: 0.00024, 2: 0.00030, 3: 0.00021, 4: 0.00039,	1: 0.00018, 2: 0.00012, 3: 0.00010, 4: 0.00026,	AZOBR_p120098		3: 0.00007,
AZOLI_p30352		1: 0.00063, 2: 0.00047, 3: 0.00031, 4: 0.00033,	AZOBR_p120099	1: 0.00020, 2: 0.00040, 3: 0.00065, 4: 0.00014, 5: 0.00014,	1: 0.00010, 2: 0.00015, 3: 0.00009,
AZOLI_p30353		1: 0.00016, 2: 0.00023, 3: 0.00025, 4: 0.00075,	AZOBR_p120101		
AZOLI_p30354	1: 0.00016, 2: 0.00011, 4: 0.00063,	1: 0.00021, 2: 0.00066, 3: 0.00084, 4: 0.00045,	AZOBR_p120103	1: 0.00011, 2: 0.00010, 4: 0.00008, 5: 0.00023,	1: 0.00014, 3: 0.00017,
AZOLI_p30355		1: 0.00010, 2: 0.00009, 3: 0.00004, 4: 0.00008,	AZOBR_p120104	1: 0.00021, 2: 0.00009, 3: 0.00044, 4: 0.00050, 5: 0.00048,	1: 0.00018, 2: 0.00018, 3: 0.00036,
AZOLI_p30356		1: 0.00007, 2: 0.00010, 3: 0.00005, 4: 0.00005,	AZOBR_p120109		
AZOLI_p30357	1: 0.00006, 2: 0.00018, 3: 0.00017, 4: 0.00015,	2: 0.00018, 3: 0.00013, 4: 0.00005,	AZOBR_p120110	1: 0.00010, 2: 0.00009, 3: 0.00008, 4: 0.00016, 5: 0.00009,	1: 0.00011, 2: 0.00022, 3: 0.00021,
AZOLI_p30360	1: 0.00005, 2: 0.00005, 4: 0.00005,	1: 0.00009, 2: 0.00010, 3: 0.00008, 4: 0.00010,	AZOBR_p120114		
AZOLI_p30365	4: 0.00003,	4: 0.00005,	AZOBR_p120116	4: 0.00025, 5: 0.00006,	
AZOLI_p30370	4: 0.00004,		AZOBR_p120117		2: 0.00006, 3: 0.00005,
AZOLI_p30371	2: 0.00018,		AZOBR_p120120	1: 0.00024, 2: 0.00095, 3: 0.00051, 4: 0.00049,	1: 0.00030, 2: 0.00026, 3: 0.00030,
AZOLI_p30375	1: 0.00006,	1: 0.00006, 2: 0.00006, 3: 0.00006, 4: 0.00007,	AZOBR_p120121	1: 0.00022, 3: 0.00014, 4: 0.00034, 5: 0.00012,	
AZOLI_p30376	2: 0.00008,	1: 0.00021, 3: 0.00014, 4: 0.00005,	AZOBR_p130008	1: 0.00014, 2: 0.00015, 3: 0.00027, 4: 0.00017, 5: 0.00007,	1: 0.00025, 2: 0.00033, 3: 0.00031,
AZOLI_p30377		1: 0.00005, 3: 0.00005, 4: 0.00004,	AZOBR_p130009	3: 0.00009,	
AZOLI_p30381	1: 0.00101, 2: 0.00095, 3: 0.00018, 4: 0.00137,	1: 0.00201, 2: 0.00251, 3: 0.00220, 4: 0.00197,	AZOBR_p130010	1: 0.00030, 3: 0.00045, 4: 0.00036, 5: 0.00021,	1: 0.00029, 2: 0.00027, 3: 0.00014,
AZOLI_p30382		1: 0.00012, 4: 0.00011,	AZOBR_p130011	4: 0.00010, 5: 0.00007,	
AZOLI_p30398		1: 0.00010, 2: 0.00006, 3: 0.00009,	AZOBR_p130015		
AZOLI_p30406	4: 0.00009,		AZOBR_p130016	4: 0.00007,	
AZOLI_p30420		4: 0.00023,	AZOBR_p130017	1: 0.00108, 2: 0.00047, 3: 0.00046, 4: 0.00093, 5: 0.00121,	1: 0.00034, 2: 0.00020, 3: 0.00058,
AZOLI_p30424	2: 0.00008, 4: 0.00008,	1: 0.00025, 2: 0.00013, 3: 0.00018, 4: 0.00010,	AZOBR_p130019		1: 0.00004,
AZOLI_p30430	2: 0.00022, 4: 0.00019,	1: 0.00022, 2: 0.00056, 3: 0.00061, 4: 0.00056,	AZOBR_p130022	1: 0.00033, 2: 0.00067, 3: 0.00065, 4: 0.00060, 5: 0.00068,	1: 0.00119, 2: 0.00060, 3: 0.00210,
AZOLI_p30433	1: 0.00018, 2: 0.00019, 3: 0.00043, 4: 0.00053,	1: 0.00125, 2: 0.00024, 3: 0.00106, 4: 0.00064,	AZOBR_p130023	5: 0.00019,	2: 0.00008,
AZOLI_p30438		4: 0.00005,	AZOBR_p130024	1: 0.00019, 3: 0.00012, 4: 0.00013, 5: 0.00019,	1: 0.00009, 2: 0.00010, 3: 0.00014,
AZOLI_p30441	2: 0.00009,	2: 0.00009,	AZOBR_p130027	1: 0.00063, 2: 0.00025, 3: 0.00044, 4: 0.00047, 5: 0.00043,	1: 0.00037, 2: 0.00042, 3: 0.00014,
AZOLI_p30442	1: 0.00012, 2: 0.00030, 4: 0.00021,	1: 0.00030, 2: 0.00012, 4: 0.00031,	AZOBR_p130032	1: 0.00018, 3: 0.00008, 4: 0.00006,	
AZOLI_p30443	1: 0.00010, 2: 0.00010, 4: 0.00022,	2: 0.00010, 3: 0.00017, 4: 0.00013,	AZOBR_p130033	1: 0.00016, 2: 0.00010, 4: 0.00019,	1: 0.00021, 2: 0.00012, 3: 0.00020,
AZOLI_p30450		1: 0.00007, 3: 0.00009, 4: 0.00006,	AZOBR_p130034	5: 0.00014,	
AZOLI_p30451		3: 0.00005,	AZOBR_p130041	4: 0.00010, 5: 0.00013,	1: 0.00024, 2: 0.00028, 3: 0.00024,
AZOLI_p30455		2: 0.00004, 4: 0.00007,	AZOBR_p130042	3: 0.00007,	
AZOLI_p30456		2: 0.00015, 4: 0.00013,	AZOBR_p130044		
AZOLI_p30457	3: 0.00018, 4: 0.00011,	1: 0.00026, 2: 0.00008, 3: 0.00014,	AZOBR_p130045		1: 0.00031, 2: 0.00029, 3: 0.00020,
AZOLI_p30459	4: 0.00007,	1: 0.00013, 3: 0.00028, 4: 0.00015,	AZOBR_p130049		
AZOLI_p30461	4: 0.00002,	1: 0.00004, 3: 0.00008, 4: 0.00002,	AZOBR_p130050		
AZOLI_p30466	1: 0.00029, 2: 0.00053,	1: 0.00053, 2: 0.00023, 3: 0.00019, 4: 0.00010,	AZOBR_p130052		
AZOLI_p30469		4: 0.00005,	AZOBR_p130057		
AZOLI_p30470	1: 0.00019, 4: 0.00008,		AZOBR_p130059	3: 0.00010, 5: 0.00008,	
AZOLI_p30476		1: 0.00004, 2: 0.00006,	AZOBR_p130067		
AZOLI_p30479	2: 0.00013, 3: 0.00012, 4: 0.00018,	1: 0.00009, 2: 0.00012, 3: 0.00023, 4: 0.00007,	AZOBR_p130068	1: 0.00024, 2: 0.00015, 3: 0.00018, 4: 0.00018, 5: 0.00013,	1: 0.00009, 2: 0.00010, 3: 0.00012,
AZOLI_p30480	4: 0.00004,	3: 0.00005, 4: 0.00004,	AZOBR_p130071	3: 0.00009, 4: 0.00007, 5: 0.00011,	3: 0.00006,
AZOLI_p30480	1: 0.00003, 2: 0.00010, 4: 0.00009,	1: 0.00026, 2: 0.00015, 3: 0.00027, 4: 0.00016,	AZOBR_p130072	4: 0.00005,	
AZOLI_p30488	1: 0.00008, 4: 0.00004,	2: 0.00010, 4: 0.00009,	AZOBR_p130073	2: 0.00008, 4: 0.00009, 5: 0.00009,	1: 0.00030, 2: 0.00019, 3: 0.00054,
AZOLI_p30489	4: 0.00005,	1: 0.00011, 3: 0.00011, 4: 0.00008,	AZOBR_p130078	5: 0.00006,	3: 0.00008,
AZOLI_p30491	2: 0.00105, 3: 0.00025,	1: 0.00079, 2: 0.00042,	AZOBR_p130082	3: 0.00032,	

	4: 0.00023,	3: 0.00047, 4: 0.00045,			
AZOLI_p30492		1: 0.00014,	AZOBR_p130083		1: 0.00002,
AZOLI_p30493	4: 0.00020,		AZOBR_p130098	1: 0.00013, 2: 0.00011, 3: 0.00011, 4: 0.00042, 5: 0.00012,	2: 0.00013, 3: 0.00005,
AZOLI_p30495	2: 0.00028, 4: 0.00016,	1: 0.00046, 2: 0.00036, 3: 0.00049, 4: 0.00016,	AZOBR_p130103	1: 0.00009, 2: 0.00023, 3: 0.00011, 4: 0.00021, 5: 0.00012,	1: 0.00036, 2: 0.00047, 3: 0.00066,
AZOLI_p30500	1: 0.00003, 3: 0.00013, 4: 0.00007,	1: 0.00019, 2: 0.00025, 3: 0.00030, 4: 0.00027,	AZOBR_p130108		1: 0.00005, 3: 0.00009,
AZOLI_p30501	1: 0.00015, 2: 0.00022, 3: 0.00033, 4: 0.00023,	1: 0.00044, 2: 0.00036, 3: 0.00031, 4: 0.00031,	AZOBR_p130110	2: 0.00015, 3: 0.00011, 4: 0.00015, 5: 0.00015,	3: 0.00029,
AZOLI_p30502	1: 0.00004, 4: 0.00008,	1: 0.00004, 2: 0.00009, 4: 0.00010,	AZOBR_p130112		
AZOLI_p30506	4: 0.00016,	3: 0.00013, 4: 0.00016,	AZOBR_p130113		3: 0.00007,
AZOLI_p30507	4: 0.00008,	1: 0.00014, 2: 0.00017, 3: 0.00038, 4: 0.00008,	AZOBR_p130114	2: 0.00026, 3: 0.00025, 4: 0.00025, 5: 0.00030,	2: 0.00011, 3: 0.00009,
AZOLI_p30509	1: 0.00036, 2: 0.00012, 3: 0.00093, 4: 0.00036,	1: 0.00079, 2: 0.00117, 3: 0.00167, 4: 0.00119,	AZOBR_p130115	3: 0.00014, 4: 0.00027, 5: 0.00055,	1: 0.00018, 2: 0.00021, 3: 0.00032,
AZOLI_p30517	4: 0.00009,	4: 0.00022,	AZOBR_p130121		
AZOLI_p30533	1: 0.00007, 2: 0.00007, 4: 0.00024,	1: 0.00014, 2: 0.00009, 3: 0.00022, 4: 0.00006,	AZOBR_p130122	1: 0.00080, 2: 0.00102, 3: 0.00068, 4: 0.00116, 5: 0.00147,	1: 0.00068, 2: 0.00062, 3: 0.00070,
AZOLI_p30534	1: 0.00022, 2: 0.00016, 4: 0.00019,	1: 0.00023, 2: 0.00018, 3: 0.00022, 4: 0.00012,	AZOBR_p130123	1: 0.00049, 2: 0.00022, 3: 0.00031, 4: 0.00055, 5: 0.00047,	2: 0.00013, 3: 0.00023,
AZOLI_p30537	2: 0.00004,		AZOBR_p130127	1: 0.00026, 2: 0.00011, 3: 0.00029, 4: 0.00018, 5: 0.00015,	
AZOLI_p30557	1: 0.00003,	1: 0.00010, 2: 0.00013, 3: 0.00006, 4: 0.00013,	AZOBR_p130128	1: 0.00027, 2: 0.00047, 3: 0.00031, 4: 0.00028, 5: 0.00034,	1: 0.00043, 2: 0.00049, 3: 0.00058,
AZOLI_p30559		1: 0.00004, 3: 0.00008, 4: 0.00007,	AZOBR_p130133	4: 0.00003,	
AZOLI_p30562		3: 0.00040,	AZOBR_p130134		
AZOLI_p30580	4: 0.00006,		AZOBR_p130135	1: 0.00713, 2: 0.00303, 3: 0.00966, 4: 0.00872, 5: 0.01256,	1: 0.00321, 2: 0.00327, 3: 0.00279,
AZOLI_p30582	1: 0.00055, 2: 0.00048, 4: 0.00044,	1: 0.00048, 2: 0.00029, 3: 0.00043, 4: 0.00032,	AZOBR_p130136	1: 0.00006, 5: 0.00006,	
AZOLI_p30589	1: 0.00011,	2: 0.00018, 3: 0.00012, 4: 0.00016,	AZOBR_p130137	3: 0.00027, 4: 0.00051, 5: 0.00015,	1: 0.00034, 3: 0.00046,
AZOLI_p30595	2: 0.00015, 4: 0.00003,	1: 0.00043, 2: 0.00022, 3: 0.00038, 4: 0.00016,	AZOBR_p130140	1: 0.00051, 2: 0.00036, 3: 0.00039, 4: 0.00021, 5: 0.00025,	1: 0.00016, 2: 0.00018, 3: 0.00016,
AZOLI_p30596	1: 0.00007, 2: 0.00007,	4: 0.00004,	AZOBR_p130141	4: 0.00007,	1: 0.00025, 2: 0.00014, 3: 0.00025,
AZOLI_p40001	1: 0.00037, 2: 0.00028, 4: 0.00016,		AZOBR_p130142	1: 0.00101, 2: 0.00107, 3: 0.00120,	2: 0.00029,
AZOLI_p40005		2: 0.00004,	AZOBR_p130143	1: 0.00024, 2: 0.00008, 3: 0.00016, 4: 0.00018, 5: 0.00025,	1: 0.00015, 2: 0.00023, 3: 0.00025,
AZOLI_p40009	4: 0.00008,		AZOBR_p130144	1: 0.00007, 2: 0.00013, 3: 0.00019, 4: 0.00018, 5: 0.00015,	2: 0.00005,
AZOLI_p40012	3: 0.00023,	1: 0.00151, 2: 0.00130, 3: 0.00139, 4: 0.00182,	AZOBR_p130147		2: 0.00011, 3: 0.00009,
AZOLI_p40023	2: 0.00006, 4: 0.00022,	1: 0.00023, 2: 0.00006, 3: 0.00037, 4: 0.00017,	AZOBR_p130148		1: 0.00003, 2: 0.00003, 3: 0.00010,
AZOLI_p40027	4: 0.00007,		AZOBR_p130149	4: 0.00035,	
AZOLI_p40031		4: 0.00011,	AZOBR_p130150	4: 0.00004,	
AZOLI_p40032	3: 0.00009,	1: 0.00003, 2: 0.00003, 3: 0.00003,	AZOBR_p130154	1: 0.00017, 2: 0.00030, 3: 0.00038, 4: 0.00035, 5: 0.00019,	1: 0.00018, 2: 0.00045, 3: 0.00007,
AZOLI_p40037	4: 0.00006,	1: 0.00020, 2: 0.00016, 3: 0.00039, 4: 0.00043,	AZOBR_p130155	1: 0.00018, 2: 0.00027, 3: 0.00016, 4: 0.00008, 5: 0.00008,	
AZOLI_p40044	3: 0.00007, 4: 0.00006,	3: 0.00004, 4: 0.00006,	AZOBR_p130158	2: 0.00016, 3: 0.00013, 4: 0.00017, 5: 0.00015,	1: 0.00049, 2: 0.00049, 3: 0.00060,
AZOLI_p40045	4: 0.00011,	2: 0.00008,	AZOBR_p130159	4: 0.00009, 5: 0.00009,	1: 0.00008, 2: 0.00009, 3: 0.00008,
AZOLI_p40046	1: 0.00017, 4: 0.00019,	1: 0.00008, 2: 0.00022, 4: 0.00005,	AZOBR_p130162		1: 0.00035,
AZOLI_p40053		3: 0.00014,	AZOBR_p130163	1: 0.01120, 2: 0.00568, 3: 0.00971, 4: 0.00707, 5: 0.00815,	1: 0.00485, 2: 0.00544, 3: 0.00296,
AZOLI_p40055	2: 0.00015, 4: 0.00030,	2: 0.00011, 3: 0.00012, 4: 0.00020,	AZOBR_p130164		3: 0.00006,
AZOLI_p40058	1: 0.00218, 2: 0.00100, 4: 0.00180,		AZOBR_p130169	1: 0.00015, 2: 0.00013, 3: 0.00026, 4: 0.00024, 5: 0.00007,	3: 0.00016,
AZOLI_p40060	1: 0.00030, 2: 0.00013, 4: 0.00017,		AZOBR_p130170	1: 0.00054, 2: 0.00048, 3: 0.00052, 4: 0.00037, 5: 0.00037,	1: 0.00043, 2: 0.00025, 3: 0.00055,
AZOLI_p40061	1: 0.00110, 2: 0.00048, 4: 0.00041,		AZOBR_p130173	5: 0.00007,	2: 0.00019, 3: 0.00010,
AZOLI_p40062	1: 0.00143, 2: 0.00092, 4: 0.00191,		AZOBR_p130179	3: 0.00007, 4: 0.00009, 5: 0.00009,	1: 0.00005, 3: 0.00005,
AZOLI_p40065	1: 0.00016, 2: 0.00010, 4: 0.00030,		AZOBR_p130180	1: 0.00027, 2: 0.00043, 3: 0.00064, 4: 0.00039, 5: 0.00052,	1: 0.00062, 2: 0.00062, 3: 0.00077,
AZOLI_p40066	1: 0.00010, 2: 0.00007, 4: 0.00004,	2: 0.00012,	AZOBR_p130182	1: 0.00087, 2: 0.00090, 3: 0.00087, 4: 0.00076, 5: 0.00086,	1: 0.00050, 2: 0.00057, 3: 0.00058,
AZOLI_p40067	1: 0.00006,		AZOBR_p130183	4: 0.00013,	2: 0.00014, 3: 0.00012,
AZOLI_p40070	4: 0.00020,		AZOBR_p130186	1: 0.00027, 2: 0.00014, 3: 0.00010, 4: 0.00005, 5: 0.00005,	1: 0.00025, 2: 0.00063, 3: 0.00029,
AZOLI_p40073	1: 0.00014, 2: 0.00016, 4: 0.00008,	1: 0.00087, 2: 0.00082, 3: 0.00097, 4: 0.00112,	AZOBR_p130191		
AZOLI_p40074		1: 0.00015, 2: 0.00006, 3: 0.00013, 4: 0.00028,	AZOBR_p130192	2: 0.00022, 3: 0.00021, 4: 0.00024, 5: 0.00014,	2: 0.00015, 3: 0.00012,
AZOLI_p40075		1: 0.00081, 2: 0.00093, 3: 0.00086, 4: 0.00073,	AZOBR_p130195	3: 0.00091, 4: 0.00191, 5: 0.00088,	1: 0.00027, 3: 0.00119,
AZOLI_p40078	3: 0.00033,		AZOBR_p130197		1: 0.00008,
AZOLI_p40079	1: 0.00013, 4: 0.00016,	2: 0.00012,	AZOBR_p130206		
AZOLI_p40100		1: 0.00009,	AZOBR_p130208		
AZOLI_p40104		1: 0.00083, 2: 0.00084,	AZOBR_p130209	1: 0.00009,	1: 0.00025, 2: 0.00019,

		3: 0.00097, 4: 0.00096,			3: 0.00029,
AZOLI_p40106	1: 0.00027, 2: 0.00038, 3: 0.00027, 4: 0.00029,	1: 0.00041, 2: 0.00047, 3: 0.00045, 4: 0.00040,	AZOBR_p130211	1: 0.00111, 2: 0.00066, 3: 0.00041, 4: 0.00064, 5: 0.00073,	1: 0.00167, 2: 0.00164, 3: 0.00171,
AZOLI_p40112	2: 0.00011,		AZOBR_p130213	4: 0.00008,	
AZOLI_p40116		4: 0.00004,	AZOBR_p140003	1: 0.00088, 2: 0.00052, 3: 0.00054, 4: 0.00078, 5: 0.00072,	1: 0.00099, 2: 0.00070, 3: 0.00091,
AZOLI_p40117	4: 0.00023,		AZOBR_p140006	1: 0.00077, 2: 0.00048, 3: 0.00072, 4: 0.00040, 5: 0.00019,	
AZOLI_p40121	1: 0.00053, 2: 0.00085, 4: 0.00122,	1: 0.00072, 2: 0.00069, 3: 0.00062, 4: 0.00050,	AZOBR_p140008	4: 0.00011,	3: 0.00005,
AZOLI_p40123	4: 0.00003,		AZOBR_p140015		
AZOLI_p40124	2: 0.00009,		AZOBR_p140019	3: 0.00015,	
AZOLI_p40128	4: 0.00160,		AZOBR_p140022	1: 0.00075, 2: 0.00061, 3: 0.00074, 4: 0.00064, 5: 0.00034,	2: 0.00023, 3: 0.00035,
AZOLI_p40132	2: 0.00031, 3: 0.00044,	1: 0.00084, 2: 0.00071, 3: 0.00055, 4: 0.00040,	AZOBR_p140023		3: 0.00007,
AZOLI_p40133	3: 0.00052,	1: 0.00075, 2: 0.00026, 3: 0.00056, 4: 0.00018,	AZOBR_p140024	3: 0.00006,	2: 0.00003, 3: 0.00003,
AZOLI_p40135		2: 0.00081, 4: 0.00051,	AZOBR_p140027	1: 0.00094, 2: 0.00083, 3: 0.00099, 4: 0.00052, 5: 0.00048,	1: 0.00265, 2: 0.00243, 3: 0.00221,
AZOLI_p40147	1: 0.00078, 2: 0.00086, 3: 0.00087, 4: 0.00094,	1: 0.00042, 2: 0.00034, 3: 0.00045, 4: 0.00044,	AZOBR_p140029		
AZOLI_p40163	1: 0.00188, 2: 0.00225, 3: 0.00430, 4: 0.00172,	1: 0.00254, 2: 0.00264, 3: 0.00218, 4: 0.00247,	AZOBR_p140030	1: 0.00020, 2: 0.00058, 3: 0.00063, 4: 0.00008, 5: 0.00008,	1: 0.00005, 2: 0.00009, 3: 0.00028,
AZOLI_p40164	1: 0.00010, 2: 0.00023, 3: 0.00018, 4: 0.00022,	1: 0.00047, 2: 0.00030, 3: 0.00047, 4: 0.00024,	AZOBR_p140033	1: 0.00041, 2: 0.00018, 3: 0.00029, 4: 0.00023, 5: 0.00014,	1: 0.00009, 2: 0.00040, 3: 0.00021,
AZOLI_p40165	1: 0.00090, 2: 0.00071, 3: 0.00137, 4: 0.00080,	1: 0.00089, 2: 0.00119, 3: 0.00107, 4: 0.00124,	AZOBR_p140035	1: 0.00025, 2: 0.00022, 4: 0.00017,	
AZOLI_p40178	4: 0.00004,		AZOBR_p140036	4: 0.00015, 5: 0.00008,	
AZOLI_p40179	4: 0.00009,	1: 0.00007, 3: 0.00011,	AZOBR_p140038	1: 0.00011,	
AZOLI_p40187	1: 0.00043, 2: 0.00035, 3: 0.00037, 4: 0.00017,	1: 0.00047, 2: 0.00023, 3: 0.00025, 4: 0.00015,	AZOBR_p140041		3: 0.00005,
AZOLI_p40188		2: 0.00004, 4: 0.00005,	AZOBR_p140042	1: 0.00011, 3: 0.00009, 4: 0.00011,	2: 0.00020, 3: 0.00007,
AZOLI_p40196	1: 0.00027, 2: 0.00009, 3: 0.00043, 4: 0.00016,	1: 0.00138, 2: 0.00154, 3: 0.00140, 4: 0.00126,	AZOBR_p140043		
AZOLI_p40206	2: 0.00042, 4: 0.00059,	1: 0.00070, 2: 0.00054, 3: 0.00089, 4: 0.00036,	AZOBR_p140045		1: 0.00008,
AZOLI_p40213		1: 0.00021, 2: 0.00021, 3: 0.00023, 4: 0.00014,	AZOBR_p140049		1: 0.00009, 2: 0.00012, 3: 0.00014,
AZOLI_p40215	4: 0.00017,		AZOBR_p140055	1: 0.00009, 3: 0.00004, 4: 0.00006, 5: 0.00003,	1: 0.00002, 2: 0.00005, 3: 0.00009,
AZOLI_p40216	2: 0.00005,		AZOBR_p140056		
AZOLI_p40218	1: 0.00007, 2: 0.00009, 3: 0.00013, 4: 0.00024,	1: 0.00010, 2: 0.00018, 3: 0.00023, 4: 0.00016,	AZOBR_p140058	1: 0.00074, 2: 0.00065, 3: 0.00039, 4: 0.00089, 5: 0.00045,	1: 0.00095, 2: 0.00054, 3: 0.00121,
AZOLI_p40221		4: 0.00010,	AZOBR_p140060		3: 0.00003,
AZOLI_p40224	4: 0.00014,		AZOBR_p140066	2: 0.00011, 3: 0.00012, 4: 0.00012, 5: 0.00017,	1: 0.00012, 2: 0.00007, 3: 0.00015,
AZOLI_p40233	1: 0.00010, 4: 0.00026,	1: 0.00048, 2: 0.00040, 3: 0.00047, 4: 0.00038,	AZOBR_p140067	1: 0.00058, 2: 0.00036, 3: 0.00052, 4: 0.00078, 5: 0.00052,	1: 0.00081, 2: 0.00075, 3: 0.00121,
AZOLI_p40238	1: 0.00013, 2: 0.00017, 3: 0.00012,	1: 0.00038, 2: 0.00033, 3: 0.00022, 4: 0.00029,	AZOBR_p140071		1: 0.00474, 2: 0.00483, 3: 0.00376,
AZOLI_p40243	4: 0.00012,	1: 0.00014, 2: 0.00032, 3: 0.00020, 4: 0.00008,	AZOBR_p140074	1: 0.00010, 3: 0.00016, 4: 0.00013, 5: 0.00007,	
AZOLI_p40247		4: 0.00004,	AZOBR_p140080	1: 0.00031, 3: 0.00020, 4: 0.00011,	1: 0.00020, 2: 0.00022, 3: 0.00024,
AZOLI_p40249	1: 0.00026, 2: 0.00013, 3: 0.00226, 4: 0.00022,	1: 0.00016, 2: 0.00025, 3: 0.00041, 4: 0.00014,	AZOBR_p140081	1: 0.00128, 2: 0.00247, 3: 0.00199, 4: 0.00213, 5: 0.00205,	1: 0.00202, 2: 0.00187, 3: 0.00167,
AZOLI_p40257		3: 0.00006, 4: 0.00007,	AZOBR_p140082	1: 0.00395, 2: 0.00308, 3: 0.00309, 4: 0.00224, 5: 0.00196,	1: 0.00212, 2: 0.00278, 3: 0.00200,
AZOLI_p40258		3: 0.00015, 4: 0.00010,	AZOBR_p140083	1: 0.00050, 2: 0.00009, 3: 0.00038, 4: 0.00038, 5: 0.00045,	1: 0.00035, 2: 0.00022, 3: 0.00049,
AZOLI_p40261		2: 0.00013, 3: 0.00009, 4: 0.00007,	AZOBR_p140086	2: 0.00013, 3: 0.00013, 4: 0.00017, 5: 0.00013,	1: 0.00019, 2: 0.00018, 3: 0.00021,
AZOLI_p40262		3: 0.00004, 4: 0.00003,	AZOBR_p140088		
AZOLI_p40272		4: 0.00024,	AZOBR_p140091		
AZOLI_p40276	1: 0.00008, 2: 0.00010, 4: 0.00013,	1: 0.00013, 2: 0.00008, 3: 0.00008, 4: 0.00013,	AZOBR_p140093		
AZOLI_p40279		2: 0.00035, 3: 0.00015, 4: 0.00025,	AZOBR_p140097		1: 0.00026, 2: 0.00037, 3: 0.00025,
AZOLI_p40284	4: 0.00039,		AZOBR_p140100		
AZOLI_p40289	1: 0.00037, 2: 0.00068, 3: 0.00078, 4: 0.00053,	1: 0.00044, 2: 0.00060, 3: 0.00026, 4: 0.00048,	AZOBR_p150007		
AZOLI_p40290	1: 0.00069, 2: 0.00037, 3: 0.00029, 4: 0.00056,	1: 0.00157, 2: 0.00195, 3: 0.00241, 4: 0.00208,	AZOBR_p150011	1: 0.00007, 2: 0.00006, 3: 0.00015, 4: 0.00012,	1: 0.00004,
AZOLI_p40291		3: 0.00009, 4: 0.00004,	AZOBR_p150013	1: 0.00045, 2: 0.00035, 3: 0.00024, 4: 0.00051, 5: 0.00066,	1: 0.00138, 2: 0.00116, 3: 0.00155,
AZOLI_p40293		1: 0.00020, 2: 0.00036, 3: 0.00036, 4: 0.00038,	AZOBR_p150015		
AZOLI_p40302		4: 0.00005,	AZOBR_p150017	1: 0.00066, 2: 0.00055, 3: 0.00062, 4: 0.00056, 5: 0.00035,	1: 0.00043, 2: 0.00054, 3: 0.00062,
AZOLI_p40309		2: 0.00007, 4: 0.00004,	AZOBR_p150027	5: 0.00018,	1: 0.00017, 2: 0.00026, 3: 0.00060,
AZOLI_p40317		2: 0.00009, 4: 0.00015,	AZOBR_p150029	4: 0.00023,	3: 0.00008,
AZOLI_p40319	1: 0.00008, 4: 0.00010,		AZOBR_p150033	4: 0.00020,	3: 0.00027,
AZOLI_p40321	4: 0.00011,		AZOBR_p150039	2: 0.00020, 3: 0.00019, 4: 0.00047,	1: 0.00044, 2: 0.00041, 3: 0.00021,
AZOLI_p40326	1: 0.00168, 2: 0.00266, 3: 0.00166, 4: 0.00256,	1: 0.00148, 2: 0.00082, 3: 0.00048, 4: 0.00092,	AZOBR_p150042	2: 0.00020,	1: 0.00050, 2: 0.00032, 3: 0.00083,
AZOLI_p40327	1: 0.00110, 2: 0.00111, 3: 0.00164, 4: 0.00083,	1: 0.00153, 2: 0.00127, 3: 0.00162, 4: 0.00101,	AZOBR_p150055		3: 0.00024,

AZOLI_p40328	1: 0.00016, 4: 0.00009,		AZOBR_p150061	1: 0.00005, 4: 0.00005,	1: 0.00021, 2: 0.00009, 3: 0.00008,
AZOLI_p40330	1: 0.00036,		AZOBR_p150064		
AZOLI_p40331	1: 0.00019, 2: 0.00027, 3: 0.00016, 4: 0.00013,	1: 0.00031, 2: 0.00018, 3: 0.00036, 4: 0.00007,	AZOBR_p150071		3: 0.00016,
AZOLI_p40335	1: 0.00010, 2: 0.00011, 4: 0.00027,	1: 0.00032, 2: 0.00024, 3: 0.00015, 4: 0.00018,	AZOBR_p150077	2: 0.00009, 4: 0.00013, 5: 0.00009,	1: 0.00008, 3: 0.00026,
AZOLI_p40337		2: 0.00005, 3: 0.00005, 4: 0.00006,	AZOBR_p150080		
AZOLI_p40339		2: 0.00006, 4: 0.00008,	AZOBR_p150083	1: 0.00018, 3: 0.00015, 4: 0.00025, 5: 0.00031,	2: 0.00019, 3: 0.00039,
AZOLI_p40341	1: 0.00016, 2: 0.00007, 4: 0.00022,	1: 0.00056, 2: 0.00067, 3: 0.00094, 4: 0.00087,	AZOBR_p150084	1: 0.00008,	2: 0.00009, 3: 0.00008,
AZOLI_p40342		3: 0.00006,	AZOBR_p150096		
AZOLI_p40349		4: 0.00013,	AZOBR_p170010		1: 0.00004,
AZOLI_p40365	2: 0.00004, 4: 0.00003,	1: 0.00003, 2: 0.00004,	AZOBR_p170022		
AZOLI_p40366		1: 0.00020, 2: 0.00024, 3: 0.00039, 4: 0.00021,	AZOBR_p170033		
AZOLI_p40369		3: 0.00005,	AZOBR_p170037	1: 0.00024, 2: 0.00015, 3: 0.00029, 4: 0.00009, 5: 0.00019,	1: 0.00007, 2: 0.00012, 3: 0.00023,
AZOLI_p40376	2: 0.00017, 3: 0.00032,	1: 0.00023, 2: 0.00056, 4: 0.00054,	AZOBR_p170038	4: 0.00010, 5: 0.00007,	
AZOLI_p40386	2: 0.00010,		AZOBR_p170039	2: 0.00010, 3: 0.00010, 4: 0.00019, 5: 0.00008,	1: 0.00017, 2: 0.00014, 3: 0.00029,
AZOLI_p40392		3: 0.00007,	AZOBR_p170040	2: 0.00006, 3: 0.00006, 4: 0.00005,	
AZOLI_p40401		1: 0.00014, 4: 0.00024,	AZOBR_p170041	1: 0.00011, 2: 0.00012, 3: 0.00006, 4: 0.00021, 5: 0.00028,	1: 0.00016, 2: 0.00014, 3: 0.00021,
AZOLI_p40420	4: 0.00002,		AZOBR_p170055		
AZOLI_p40428	1: 0.00043, 2: 0.00113, 3: 0.00109, 4: 0.00149,	1: 0.00140, 2: 0.00093, 3: 0.00120, 4: 0.00090,	AZOBR_p170093	4: 0.00003,	1: 0.00002, 3: 0.00004,
AZOLI_p40429	1: 0.00007, 2: 0.00008, 3: 0.00013, 4: 0.00018,	1: 0.00025, 2: 0.00027, 3: 0.00030, 4: 0.00032,	AZOBR_p170094	1: 0.00016, 2: 0.00014, 3: 0.00009, 4: 0.00044, 5: 0.00040,	1: 0.00020, 3: 0.00026,
AZOLI_p40433		4: 0.00002,	AZOBR_p170096	1: 0.00045, 2: 0.00023, 3: 0.00034, 4: 0.00040, 5: 0.00066,	1: 0.00051, 2: 0.00025, 3: 0.00038,
AZOLI_p40436	1: 0.00055, 2: 0.00088, 4: 0.00059,	1: 0.00051, 2: 0.00058, 3: 0.00025, 4: 0.00040,	AZOBR_p170117		
AZOLI_p40437	1: 0.01442, 2: 0.01192, 3: 0.00115, 4: 0.01414,	1: 0.00166, 2: 0.00192, 3: 0.00088, 4: 0.00113,	AZOBR_p170119	1: 0.00027, 4: 0.00008, 5: 0.00011,	1: 0.00007, 3: 0.00012,
AZOLI_p40439	1: 0.00168, 2: 0.00262, 4: 0.00356,	1: 0.00031, 2: 0.00075, 3: 0.00033,	AZOBR_p170140	4: 0.00013, 5: 0.00009,	
AZOLI_p40440	4: 0.00010,	2: 0.00008, 3: 0.00013,	AZOBR_p170141		2: 0.00008,
AZOLI_p40441	1: 0.00034, 2: 0.00019, 3: 0.00009, 4: 0.00005,	1: 0.00016, 2: 0.00018, 3: 0.00010, 4: 0.00008,	AZOBR_p170142	1: 0.00022, 2: 0.00046, 3: 0.00045, 4: 0.00048, 5: 0.00027,	1: 0.00034, 2: 0.00048, 3: 0.00024,
AZOLI_p40442	1: 0.00056, 2: 0.00057, 4: 0.00073,		AZOBR_p170145	4: 0.00009,	
AZOLI_p40443	1: 0.00020, 3: 0.00125,	2: 0.00040, 3: 0.00037, 4: 0.00035,	AZOBR_p170146	4: 0.00004,	1: 0.00015, 3: 0.00003,
AZOLI_p40444	1: 0.00039, 2: 0.00059, 3: 0.00070, 4: 0.00028,	1: 0.00059, 2: 0.00053, 3: 0.00059, 4: 0.00059,	AZOBR_p170147		3: 0.00004,
AZOLI_p40445	2: 0.00040, 3: 0.00112, 4: 0.00120,	1: 0.00040, 2: 0.00058, 4: 0.00051,	AZOBR_p170152	2: 0.00010, 3: 0.00010, 4: 0.00008, 5: 0.00012,	
AZOLI_p40446	4: 0.00013,		AZOBR_p170153		
AZOLI_p40453		1: 0.00062, 2: 0.00034, 3: 0.00075, 4: 0.00053,	AZOBR_p170156		1: 0.00138, 2: 0.00117, 3: 0.00103,
AZOLI_p40460	2: 0.00105, 3: 0.00146, 4: 0.00034,	2: 0.00031,	AZOBR_p180001		2: 0.00047, 3: 0.00027,
AZOLI_p40461	1: 0.00015,		AZOBR_p180006	1: 0.00035, 2: 0.00010, 3: 0.00040, 4: 0.00085, 5: 0.00033,	2: 0.00013, 3: 0.00007,
AZOLI_p40463	1: 0.00033, 2: 0.00044, 3: 0.00015, 4: 0.00036,	1: 0.00051, 2: 0.00045, 3: 0.00043, 4: 0.00042,	AZOBR_p180009		
AZOLI_p40464	1: 0.00014, 2: 0.00029, 3: 0.00035, 4: 0.00025,	1: 0.00007, 2: 0.00017, 3: 0.00042, 4: 0.00028,	AZOBR_p180016		3: 0.00006,
AZOLI_p40465	1: 0.00067, 2: 0.00083, 3: 0.00067, 4: 0.00101,	1: 0.00035, 2: 0.00043, 3: 0.00048, 4: 0.00052,	AZOBR_p190002	1: 0.00015,	
AZOLI_p40466	1: 0.00018, 2: 0.00015, 3: 0.00017, 4: 0.00028,	1: 0.00015, 2: 0.00029, 3: 0.00019, 4: 0.00020,	AZOBR_p1100001		3: 0.00002,
AZOLI_p40467		1: 0.00006, 4: 0.00005,	AZOBR_p1100002	1: 0.00526, 2: 0.00720, 3: 0.00578, 4: 0.00362, 5: 0.00498,	
AZOLI_p40471	1: 0.00031, 2: 0.00085, 3: 0.00177, 4: 0.00063,	1: 0.00159, 2: 0.00051, 3: 0.00157, 4: 0.00054,	AZOBR_p1100009	4: 0.00028, 5: 0.00028,	
AZOLI_p40472		1: 0.00011, 4: 0.00014,	AZOBR_p1100012		2: 0.00015, 3: 0.00013,
AZOLI_p40473	2: 0.00009, 4: 0.00008,	1: 0.00032, 2: 0.00044, 3: 0.00019, 4: 0.00016,	AZOBR_p1100018	1: 0.00018, 3: 0.00038, 4: 0.00049, 5: 0.00037,	1: 0.00017, 2: 0.00039, 3: 0.00050,
AZOLI_p40476		1: 0.00023, 3: 0.00016, 4: 0.00013,	AZOBR_p1100019	2: 0.00008,	1: 0.00009, 2: 0.00007,
AZOLI_p40482	1: 0.00005, 2: 0.00006, 4: 0.00005,	1: 0.00003, 2: 0.00002,	AZOBR_p1100020	1: 0.00027, 2: 0.00024, 3: 0.00015, 4: 0.00028, 5: 0.00006,	1: 0.00009, 2: 0.00026, 3: 0.00022,
AZOLI_p40483	2: 0.00006,	1: 0.00014, 3: 0.00012, 4: 0.00015,	AZOBR_p1100022		
AZOLI_p40488	1: 0.00037, 2: 0.00017, 4: 0.00023,	1: 0.00132, 2: 0.00180, 3: 0.00172, 4: 0.00173,	AZOBR_p1100026	4: 0.00012,	
AZOLI_p40490	1: 0.00009, 4: 0.00012,		AZOBR_p1100032		2: 0.00011,
AZOLI_p40491	1: 0.00046, 2: 0.00039, 4: 0.00059,		AZOBR_p1100034		3: 0.00013,
AZOLI_p40492	4: 0.00011,		AZOBR_p1100035	4: 0.00003,	
AZOLI_p40499		3: 0.00031, 4: 0.00018,	AZOBR_p1100045		
AZOLI_p40500	1: 0.00022, 2: 0.00027, 4: 0.00023,		AZOBR_p1100049	4: 0.00032,	1: 0.00030,
AZOLI_p40501	1: 0.00013, 2: 0.00016, 4: 0.00019,	4: 0.00004,	AZOBR_p1100059		

AZOLI_p40505	4: 0.00011,		AZOBR_p1100060	4: 0.00003, 5: 0.00002,	3: 0.00003,
AZOLI_p40506		1: 0.00018, 2: 0.00008, 3: 0.00008, 4: 0.00007,	AZOBR_p1100061		2: 0.00005,
AZOLI_p40508	1: 0.00019, 2: 0.00005, 4: 0.00005,	2: 0.00010, 4: 0.00009,	AZOBR_p1100065	1: 0.00026, 3: 0.00015, 4: 0.00012, 5: 0.00012,	
AZOLI_p40512	4: 0.00003,		AZOBR_p1100066	1: 0.00010, 2: 0.00026, 3: 0.00038, 4: 0.00051, 5: 0.00034,	1: 0.00013, 2: 0.00022, 3: 0.00022,
AZOLI_p40518	1: 0.00005, 3: 0.00005, 4: 0.00007,		AZOBR_p1100074		
AZOLI_p40520	4: 0.00025,		AZOBR_p1100079	3: 0.00009, 4: 0.00011,	2: 0.00012,
AZOLI_p40521		4: 0.00001,	AZOBR_p1100082	3: 0.00007,	
AZOLI_p40522	1: 0.00056, 2: 0.00082, 3: 0.00109, 4: 0.00063,	1: 0.00074, 2: 0.00050, 3: 0.00051, 4: 0.00045,	AZOBR_p1100083	3: 0.00010,	
AZOLI_p40529	1: 0.00009, 2: 0.00018, 3: 0.00025, 4: 0.00019,	1: 0.00018, 2: 0.00017, 3: 0.00019, 4: 0.00019,	AZOBR_p1100090		2: 0.00023,
AZOLI_p40530	1: 0.00057, 2: 0.00016, 3: 0.00029, 4: 0.00056,	1: 0.00019, 2: 0.00053, 3: 0.00034, 4: 0.00029,	AZOBR_p1100091		1: 0.00063, 3: 0.00085,
AZOLI_p40531	3: 0.00040,	2: 0.00014, 3: 0.00030,	AZOBR_p1100092	4: 0.00029,	
AZOLI_p40537	1: 0.00006, 4: 0.00008,	2: 0.00012, 3: 0.00022, 4: 0.00020,	AZOBR_p1100098	1: 0.00085, 3: 0.00072, 5: 0.00025,	
AZOLI_p40538	1: 0.00047, 2: 0.00159, 3: 0.00199, 4: 0.00217,	1: 0.00351, 2: 0.00322, 3: 0.00354, 4: 0.00299,	AZOBR_p1100099	4: 0.00012,	1: 0.00027, 2: 0.00019, 3: 0.00021,
AZOLI_p40561		2: 0.00024,	AZOBR_p1100102	3: 0.00012,	
AZOLI_p40585		1: 0.00022, 2: 0.00015, 3: 0.00029, 4: 0.00022,	AZOBR_p1100108		
AZOLI_p40593		2: 0.00020, 3: 0.00022, 4: 0.00012,	AZOBR_p1110003	1: 0.00030, 2: 0.00038, 3: 0.00022, 4: 0.00033, 5: 0.00030,	1: 0.00011, 2: 0.00009, 3: 0.00019,
AZOLI_p40594		1: 0.00022, 2: 0.00044, 3: 0.00013, 4: 0.00031,	AZOBR_p1110013		
AZOLI_p40599	4: 0.00011,		AZOBR_p1110014		
AZOLI_p40607	1: 0.00016, 2: 0.00008,		AZOBR_p1110016	1: 0.00008, 5: 0.00006,	1: 0.00008, 3: 0.00010,
AZOLI_p40614	3: 0.00051, 4: 0.00019,	3: 0.00010,	AZOBR_p1110017	4: 0.00008,	
AZOLI_p40616	1: 0.00095, 2: 0.00076, 3: 0.00115, 4: 0.00099,	1: 0.00086, 2: 0.00057, 3: 0.00077, 4: 0.00054,	AZOBR_p1110021	1: 0.00138, 2: 0.00091, 3: 0.00103, 4: 0.00120, 5: 0.00151,	1: 0.00137, 2: 0.00120, 3: 0.00147,
AZOLI_p40619	1: 0.00036, 4: 0.00051,		AZOBR_p1110022	4: 0.00019,	
AZOLI_p40620	1: 0.00012, 4: 0.00017,	4: 0.00008,	AZOBR_p1110032	2: 0.00005, 3: 0.00005, 4: 0.00004,	2: 0.00015, 3: 0.00015,
AZOLI_p40622	1: 0.00219, 2: 0.00124, 3: 0.00104, 4: 0.00159,	1: 0.00084, 2: 0.00096, 3: 0.00096, 4: 0.00077,	AZOBR_p1110033	1: 0.00007, 2: 0.00015, 3: 0.00011, 4: 0.00015, 5: 0.00007,	1: 0.00004, 2: 0.00003, 3: 0.00009,
AZOLI_p40624	2: 0.00084, 3: 0.00330, 4: 0.00226,	1: 0.00098, 2: 0.00094, 3: 0.00089, 4: 0.00119,	AZOBR_p1110035	2: 0.00011, 4: 0.00006, 5: 0.00008,	1: 0.00012, 2: 0.00011, 3: 0.00013,
AZOLI_p40625		2: 0.00004,	AZOBR_p1110039	1: 0.00031, 2: 0.00035, 3: 0.00011, 4: 0.00009, 5: 0.00012,	1: 0.00014, 2: 0.00013, 3: 0.00025,
AZOLI_p40626	1: 0.00086, 2: 0.00139, 3: 0.00141, 4: 0.00107,	1: 0.00185, 2: 0.00205, 3: 0.00181, 4: 0.00128,	AZOBR_p1110040	1: 0.00063, 2: 0.00031, 3: 0.00065, 4: 0.00072, 5: 0.00053,	1: 0.00009, 2: 0.00025, 3: 0.00013,
AZOLI_p40633	4: 0.00005,		AZOBR_p1110041	2: 0.00033, 3: 0.00035, 4: 0.00011,	
AZOLI_p40634	1: 0.00242, 2: 0.00173, 3: 0.00341, 4: 0.00165,	1: 0.00543, 2: 0.00690, 3: 0.00542, 4: 0.00480,	AZOBR_p1110043	3: 0.00017, 4: 0.00013, 5: 0.00007,	1: 0.00053, 2: 0.00018, 3: 0.00069,
AZOLI_p40635	4: 0.00016,		AZOBR_p1110044	1: 0.00016, 3: 0.00018, 4: 0.00007, 5: 0.00011,	1: 0.00017, 2: 0.00015, 3: 0.00016,
AZOLI_p40637	1: 0.00005, 4: 0.00005,		AZOBR_p1110045		1: 0.00007, 2: 0.00008,
AZOLI_p40638		2: 0.00005,	AZOBR_p1110046	1: 0.00257, 2: 0.00291, 3: 0.00256, 4: 0.00126, 5: 0.00177,	1: 0.00348, 2: 0.00246, 3: 0.00319,
AZOLI_p40641	1: 0.00009,		AZOBR_p1110047	1: 0.00143, 2: 0.00118, 3: 0.00146, 4: 0.00145, 5: 0.00092,	1: 0.00092, 2: 0.00125, 3: 0.00107,
AZOLI_p40642	1: 0.00024, 2: 0.00021, 3: 0.00206, 4: 0.00024,	1: 0.00025, 2: 0.00031, 4: 0.00018,	AZOBR_p1110048	4: 0.00012,	
AZOLI_p40643	1: 0.00013, 2: 0.00039, 3: 0.00012, 4: 0.00029,	1: 0.00065, 2: 0.00092, 3: 0.00046, 4: 0.00066,	AZOBR_p1110049	1: 0.00018, 2: 0.00016, 3: 0.00021, 4: 0.00024, 5: 0.00012,	1: 0.00011, 2: 0.00031, 3: 0.00011,
AZOLI_p40647	2: 0.00036, 4: 0.00054,	2: 0.00035, 3: 0.00019, 4: 0.00031,	AZOBR_p1110051		
AZOLI_p40647		1: 0.00045, 2: 0.00015, 3: 0.00024, 4: 0.00026,	AZOBR_p1110053		
AZOLI_p40649		1: 0.00011, 3: 0.00006, 4: 0.00005,	AZOBR_p1110054	4: 0.00011, 5: 0.00013,	3: 0.00005,
AZOLI_p40651	1: 0.00139, 2: 0.00116, 3: 0.00239, 4: 0.00104,	1: 0.00025, 2: 0.00071, 3: 0.00026, 4: 0.00016,	AZOBR_p1110056	2: 0.00011, 3: 0.00014, 4: 0.00028, 5: 0.00028,	1: 0.00016, 2: 0.00012, 3: 0.00033,
AZOLI_p40653	4: 0.00003,	4: 0.00005,	AZOBR_p1110059	1: 0.00040, 2: 0.00018, 3: 0.00017, 4: 0.00041, 5: 0.00027,	1: 0.00019, 3: 0.00031,
AZOLI_p40654		3: 0.00008,	AZOBR_p1110060	1: 0.00011, 2: 0.00024, 3: 0.00014, 5: 0.00015,	2: 0.00024,
AZOLI_p40656	1: 0.00770, 2: 0.00697, 3: 0.00172, 4: 0.00643,	1: 0.00229, 2: 0.00316, 3: 0.00315, 4: 0.00343,	AZOBR_p1110063	1: 0.00005, 3: 0.00004, 4: 0.00017, 5: 0.00008,	1: 0.00007, 2: 0.00013, 3: 0.00009,
AZOLI_p40658	3: 0.00033,	1: 0.00043, 2: 0.00062, 3: 0.00053, 4: 0.00053,	AZOBR_p1110065		2: 0.00011,
AZOLI_p50014		4: 0.00002,	AZOBR_p1110069		1: 0.00009, 2: 0.00003, 3: 0.00013,
AZOLI_p50036	1: 0.00006, 2: 0.00008, 3: 0.00035, 4: 0.00024,		AZOBR_p1110075	1: 0.00107, 2: 0.00094, 3: 0.00104, 4: 0.00235, 5: 0.00207,	1: 0.00127, 2: 0.00104, 3: 0.00174,
AZOLI_p50060	1: 0.00006, 2: 0.00009, 4: 0.00013,	1: 0.00024, 2: 0.00011, 3: 0.00016, 4: 0.00020,	AZOBR_p1110076	1: 0.00740, 2: 0.00936, 3: 0.00890, 4: 0.00700, 5: 0.01412,	1: 0.00781, 2: 0.00603, 3: 0.00767,
AZOLI_p50061	1: 0.00012, 2: 0.00007, 3: 0.00014, 4: 0.00019,	1: 0.00010, 2: 0.00015, 3: 0.00027, 4: 0.00017,	AZOBR_p1110080	1: 0.00041, 2: 0.00016, 3: 0.00022, 4: 0.00028, 5: 0.00008,	1: 0.00017, 2: 0.00030, 3: 0.00012,
AZOLI_p50062	1: 0.00008, 3: 0.00035, 4: 0.00021,	1: 0.00021, 2: 0.00012, 3: 0.00022, 4: 0.00014,	AZOBR_p1110081		3: 0.00006,
AZOLI_p50063	1: 0.00016, 4: 0.00017,	1: 0.00012, 2: 0.00015, 4: 0.00031,	AZOBR_p1110082	3: 0.00019, 4: 0.00006, 5: 0.00006,	2: 0.00023, 3: 0.00011,
AZOLI_p50080	1: 0.00019, 2: 0.00016, 4: 0.00022,	3: 0.00007,	AZOBR_p1110083		3: 0.00009,
AZOLI_p50090	1: 0.00005, 2: 0.00003, 4: 0.00006,		AZOBR_p1110084	1: 0.00036, 2: 0.00055, 3: 0.00048, 4: 0.00077, 5: 0.00075,	1: 0.00019, 2: 0.00019, 3: 0.00039,

AZOLI_p50095	4: 0.00008,		AZOBR_p1110087	4: 0.00024, 5: 0.00016,	1: 0.00015, 2: 0.00034, 3: 0.00081,
AZOLI_p50096		4: 0.00008,	AZOBR_p1110097	2: 0.00017, 3: 0.00012, 4: 0.00013,	1: 0.00033, 2: 0.00027, 3: 0.00026,
AZOLI_p50098		3: 0.00019, 4: 0.00020,	AZOBR_p1110099	1: 0.00094, 2: 0.00083, 3: 0.00080, 5: 0.00028,	1: 0.00043, 2: 0.00078, 3: 0.00042,
AZOLI_p50099	4: 0.00004,	4: 0.00003,	AZOBR_p1110100	1: 0.00014, 4: 0.00014, 5: 0.00004,	2: 0.00015, 3: 0.00011,
AZOLI_p50101	1: 0.00011, 2: 0.00009, 3: 0.00005, 4: 0.00015,	1: 0.00002, 2: 0.00005, 3: 0.00004, 4: 0.00005,	AZOBR_p1110104	1: 0.00024, 3: 0.00010, 5: 0.00008,	1: 0.00051, 2: 0.00058, 3: 0.00042,
AZOLI_p50103		4: 0.00008,	AZOBR_p1110105		3: 0.00007,
AZOLI_p50106	1: 0.00013, 2: 0.00020, 4: 0.00008,	1: 0.00020, 2: 0.00022, 3: 0.00035, 4: 0.00025,	AZOBR_p1110108		1: 0.00003, 2: 0.00007, 3: 0.00003,
AZOLI_p50117	4: 0.00002,		AZOBR_p1110117		2: 0.00008,
AZOLI_p50138	4: 0.00009,		AZOBR_p1110126	2: 0.00007,	3: 0.00005,
AZOLI_p50151	1: 0.00030, 2: 0.00030, 4: 0.00038,	1: 0.00008, 2: 0.00008, 3: 0.00007, 4: 0.00009,	AZOBR_p1110127	3: 0.00015, 4: 0.00029, 5: 0.00012,	2: 0.00044, 3: 0.00027,
AZOLI_p50152	1: 0.00024, 4: 0.00024,	1: 0.00008,	AZOBR_p1110128	2: 0.00018, 3: 0.00012, 4: 0.00033, 5: 0.00010,	1: 0.00013, 2: 0.00025, 3: 0.00047,
AZOLI_p50154	1: 0.00073, 2: 0.00079, 4: 0.00085,	1: 0.00032, 2: 0.00041, 3: 0.00045, 4: 0.00045,	AZOBR_p1110129	1: 0.00029, 3: 0.00016, 4: 0.00020,	3: 0.00030,
AZOLI_p50160	1: 0.00083, 2: 0.00067, 4: 0.00043,	3: 0.00036, 4: 0.00029,	AZOBR_p1110133	1: 0.00286, 2: 0.00216, 3: 0.00254, 4: 0.00160, 5: 0.00117,	1: 0.00380, 2: 0.00423, 3: 0.00271,
AZOLI_p50162	1: 0.00606, 2: 0.00484, 3: 0.00100, 4: 0.00469,	1: 0.00661, 2: 0.00721, 3: 0.00647, 4: 0.00660,	AZOBR_p1110136	3: 0.00014,	3: 0.00010,
AZOLI_p50164		1: 0.00015, 2: 0.00019, 4: 0.00008,	AZOBR_p1110140		
AZOLI_p50164		1: 0.00015, 2: 0.00019, 4: 0.00008,	AZOBR_p1110150		
AZOLI_p50165		3: 0.00020, 4: 0.00013,	AZOBR_p1110152		
AZOLI_p50165		3: 0.00020, 4: 0.00013,	AZOBR_p1110165		
AZOLI_p50168	1: 0.00052, 2: 0.00049, 4: 0.00036,		AZOBR_p1110167		
AZOLI_p50173	1: 0.00018,		AZOBR_p1110170	3: 0.00005, 4: 0.00007,	2: 0.00008, 3: 0.00002,
AZOLI_p50175	1: 0.00035, 2: 0.00043, 3: 0.00153, 4: 0.00087,	1: 0.00079, 2: 0.00079, 3: 0.00104, 4: 0.00064,	AZOBR_p1120005	5: 0.00018,	
AZOLI_p50182		1: 0.00030, 2: 0.00023, 3: 0.00019, 4: 0.00013,	AZOBR_p1120007		
AZOLI_p50183	1: 0.00053, 2: 0.00057, 3: 0.00011, 4: 0.00045,	1: 0.00021, 2: 0.00028, 3: 0.00048, 4: 0.00039,	AZOBR_p1120008		2: 0.00006,
AZOLI_p50184	2: 0.00011,		AZOBR_p1120009		
AZOLI_p50186		4: 0.00005,	AZOBR_p1120011	1: 0.00229, 2: 0.00352, 3: 0.00228, 4: 0.00267, 5: 0.00327,	1: 0.00332, 2: 0.00298, 3: 0.00315,
AZOLI_p50188		1: 0.00006, 2: 0.00006, 3: 0.00011, 4: 0.00005,	AZOBR_p1120013	5: 0.00006,	
AZOLI_p50191	2: 0.00006, 4: 0.00005,	1: 0.00039, 2: 0.00019, 3: 0.00033, 4: 0.00037,	AZOBR_p1120014	2: 0.00007, 3: 0.00007, 4: 0.00004, 5: 0.00004,	
AZOLI_p50201	1: 0.00013, 2: 0.00029, 4: 0.00017,		AZOBR_p1120016	1: 0.00247, 2: 0.00198, 3: 0.00220, 4: 0.00208, 5: 0.00213,	1: 0.00237, 2: 0.00204, 3: 0.00195,
AZOLI_p50207	1: 0.00058, 2: 0.00022, 3: 0.00138, 4: 0.00044,	1: 0.00287, 2: 0.00276, 3: 0.00300, 4: 0.00382,	AZOBR_p1120020	1: 0.00024, 2: 0.00026, 3: 0.00041, 4: 0.00049, 5: 0.00025,	1: 0.00008, 2: 0.00017, 3: 0.00007,
AZOLI_p50210	1: 0.00231, 2: 0.00151, 3: 0.00167, 4: 0.00156,	1: 0.00247, 2: 0.00275, 3: 0.00248, 4: 0.00281,	AZOBR_p1120021	1: 0.00015, 3: 0.00010, 4: 0.00015, 5: 0.00015,	1: 0.00011, 2: 0.00014, 3: 0.00018,
AZOLI_p50212	2: 0.00003,	4: 0.00003,	AZOBR_p1120024		
AZOLI_p50216		4: 0.00014,	AZOBR_p1120026	1: 0.00262, 2: 0.00178, 3: 0.00155, 4: 0.00292, 5: 0.00222,	1: 0.00129, 2: 0.00059, 3: 0.00175,
AZOLI_p50217	1: 0.00012,		AZOBR_p1120027	1: 0.00028, 2: 0.00050, 3: 0.00036, 5: 0.00015,	1: 0.00014, 2: 0.00051, 3: 0.00018,
AZOLI_p50218	1: 0.00010, 4: 0.00011,	1: 0.00007,	AZOBR_p1120028	1: 0.00010, 2: 0.00015, 3: 0.00021, 4: 0.00007, 5: 0.00005,	2: 0.00008, 3: 0.00013,
AZOLI_p50219	4: 0.00036,		AZOBR_p1120033		
AZOLI_p50220		1: 0.00013, 2: 0.00022, 3: 0.00017, 4: 0.00033,	AZOBR_p1120035		
AZOLI_p50224	2: 0.00074, 3: 0.00162, 4: 0.00028,	2: 0.00016,	AZOBR_p1120038		
AZOLI_p50225		1: 0.00030, 3: 0.00026, 4: 0.00010,	AZOBR_p1120039	3: 0.00013, 4: 0.00013, 5: 0.00011,	2: 0.00009,
AZOLI_p50233		2: 0.00031, 3: 0.00011, 4: 0.00018,	AZOBR_p1120040		1: 0.00018, 2: 0.00008, 3: 0.00043,
AZOLI_p50235	2: 0.00005, 4: 0.00009,	1: 0.00034, 2: 0.00025, 3: 0.00033, 4: 0.00013,	AZOBR_p1120044	1: 0.00020,	2: 0.00015, 3: 0.00013,
AZOLI_p50239	1: 0.00009,		AZOBR_p1120045	5: 0.00017,	
AZOLI_p50240	1: 0.00002,		AZOBR_p1120047	1: 0.00007, 2: 0.00006,	2: 0.00007, 3: 0.00006,
AZOLI_p50241		1: 0.00009,	AZOBR_p1120048	1: 0.00006,	2: 0.00004,
AZOLI_p50242		3: 0.00008, 4: 0.00006,	AZOBR_p1120049		
AZOLI_p50244	1: 0.00005,	2: 0.00005,	AZOBR_p1120051		
AZOLI_p50247	1: 0.00031, 2: 0.00033, 3: 0.00026, 4: 0.00044,	1: 0.00075, 2: 0.00056, 3: 0.00071, 4: 0.00048,	AZOBR_p1120052	1: 0.00033, 2: 0.00032, 3: 0.00026, 4: 0.00036, 5: 0.00035,	1: 0.00124, 2: 0.00145, 3: 0.00088,
AZOLI_p50248	4: 0.00017,	4: 0.00007,	AZOBR_p1120056	3: 0.00009, 4: 0.00012,	3: 0.00005,
AZOLI_p50251		4: 0.00005,	AZOBR_p1120060		
AZOLI_p50252	4: 0.00009,	3: 0.00004,	AZOBR_p1120061		
AZOLI_p50253		2: 0.00033, 4: 0.00022,	AZOBR_p1120064	2: 0.00019, 4: 0.00015, 5: 0.00015,	1: 0.00010, 2: 0.00012, 3: 0.00016,
AZOLI_p50254		2: 0.00009,	AZOBR_p1130006		3: 0.00005,
AZOLI_p50256		4: 0.00005,	AZOBR_p1130008	3: 0.00011,	
AZOLI_p50258		1: 0.00008,	AZOBR_p1130019		1: 0.00006,
AZOLI_p50259	1: 0.00012, 2: 0.00034, 3: 0.00030, 4: 0.00023,	1: 0.00015, 2: 0.00035, 3: 0.00032, 4: 0.00021,	AZOBR_p1130021		
AZOLI_p50265	1: 0.00033, 2: 0.00013, 4: 0.00040,	1: 0.00101, 2: 0.00026, 3: 0.00036, 4: 0.00034,	AZOBR_p1130022		

AZOLI_p50268	3: 0.00051,	3: 0.00026, 4: 0.00028,	AZOBR_p1130024	1: 0.00016, 2: 0.00011, 3: 0.00020, 4: 0.00017, 5: 0.00014,	1: 0.00008, 2: 0.00009, 3: 0.00010,
AZOLI_p50274		1: 0.00020, 2: 0.00021, 3: 0.00027, 4: 0.00025,	AZOBR_p1130025		1: 0.00025, 3: 0.00060,
AZOLI_p50276		2: 0.00007, 4: 0.00013,	AZOBR_p1130026		1: 0.00006,
AZOLI_p50282	4: 0.00025,	2: 0.00034, 4: 0.00020,	AZOBR_p1130039		
AZOLI_p50283		1: 0.00005, 4: 0.00004,	AZOBR_p1130041		3: 0.00010,
AZOLI_p50284	1: 0.00006,	1: 0.00006, 4: 0.00008,	AZOBR_p1130044		
AZOLI_p50286	4: 0.00008,		AZOBR_p1130051	4: 0.00016,	
AZOLI_p50297	1: 0.00018, 2: 0.00048, 4: 0.00067,	2: 0.00029, 4: 0.00026,	AZOBR_p1130052		
AZOLI_p50299	1: 0.00269, 2: 0.00266, 3: 0.00358, 4: 0.00240,	1: 0.00201, 2: 0.00216, 3: 0.00182, 4: 0.00342,	AZOBR_p1130056	3: 0.00004, 4: 0.00005,	1: 0.00006, 3: 0.00005,
AZOLI_p50300	1: 0.00421, 2: 0.00329, 3: 0.00373, 4: 0.00422,	1: 0.00202, 2: 0.00251, 3: 0.00195, 4: 0.00191,	AZOBR_p1130057	3: 0.00007, 4: 0.00011,	1: 0.00010, 3: 0.00010,
AZOLI_p50301	1: 0.00058, 2: 0.00090, 3: 0.00011, 4: 0.00063,	1: 0.00020, 2: 0.00023, 3: 0.00037, 4: 0.00017,	AZOBR_p1130061	4: 0.00006,	
AZOLI_p50307		1: 0.00017, 2: 0.00011, 4: 0.00007,	AZOBR_p1130062	2: 0.00005, 3: 0.00007, 4: 0.00004, 5: 0.00006,	1: 0.00016, 2: 0.00016, 3: 0.00033,
AZOLI_p50317		3: 0.00011, 4: 0.00009,	AZOBR_p1130065		2: 0.00003,
AZOLI_p50318		1: 0.00035, 2: 0.00010, 3: 0.00020, 4: 0.00018,	AZOBR_p1130067		
AZOLI_p50321	1: 0.00003,		AZOBR_p1130068		
AZOLI_p50336	4: 0.00008,		AZOBR_p1130071	4: 0.00028,	1: 0.00013, 2: 0.00015, 3: 0.00030,
AZOLI_p50355	1: 0.00057, 2: 0.00061, 4: 0.00025,		AZOBR_p1130073	1: 0.00022, 2: 0.00011, 3: 0.00019, 4: 0.00024, 5: 0.00022,	1: 0.00071, 2: 0.00049, 3: 0.00064,
AZOLI_p50360		1: 0.00007, 2: 0.00008, 3: 0.00007, 4: 0.00013,	AZOBR_p1130074	3: 0.00030, 4: 0.00036,	2: 0.00013, 3: 0.00016,
AZOLI_p50362		1: 0.00047, 2: 0.00043, 3: 0.00039, 4: 0.00046,	AZOBR_p1130075	1: 0.00024, 2: 0.00117, 3: 0.00102, 4: 0.00058, 5: 0.00066,	1: 0.00108, 2: 0.00079, 3: 0.00037,
AZOLI_p50365		3: 0.00009, 4: 0.00004,	AZOBR_p1130076	1: 0.00016, 2: 0.00007, 3: 0.00030, 4: 0.00014, 5: 0.00014,	3: 0.00007,
AZOLI_p50366	1: 0.00012, 4: 0.00007,		AZOBR_p1130077	3: 0.00013, 4: 0.00011, 5: 0.00014,	1: 0.00017, 3: 0.00013,
AZOLI_p50367		1: 0.00003,	AZOBR_p1130080	1: 0.00072, 2: 0.00037, 3: 0.00066, 4: 0.00049, 5: 0.00037,	1: 0.00057, 2: 0.00039, 3: 0.00059,
AZOLI_p50380	4: 0.00005,	1: 0.00006,	AZOBR_p1130088	1: 0.00038, 3: 0.00019, 4: 0.00023, 5: 0.00015,	2: 0.00024, 3: 0.00024,
AZOLI_p50389	1: 0.00005, 2: 0.00005, 4: 0.00004,		AZOBR_p1130089		
AZOLI_p50394	1: 0.00008, 2: 0.00020, 4: 0.00007,		AZOBR_p1130090	1: 0.00004,	
AZOLI_p50397		2: 0.00019, 3: 0.00016, 4: 0.00013,	AZOBR_p1130091	1: 0.00009, 2: 0.00013, 3: 0.00014, 4: 0.00043, 5: 0.00018,	1: 0.00011, 2: 0.00019, 3: 0.00014,
AZOLI_p50402	1: 0.00013, 2: 0.00008, 4: 0.00015,		AZOBR_p1130093		
AZOLI_p50404	2: 0.00011,	1: 0.00007, 2: 0.00021, 3: 0.00016, 4: 0.00022,	AZOBR_p1130095		
AZOLI_p50406	1: 0.00185, 2: 0.00140, 3: 0.00228, 4: 0.00076,	1: 0.00080, 2: 0.00122, 3: 0.00069, 4: 0.00080,	AZOBR_p1130096	1: 0.00015, 3: 0.00013,	
AZOLI_p50414	4: 0.00009,		AZOBR_p1130097	4: 0.00008, 5: 0.00008,	
AZOLI_p50415	1: 0.00011, 2: 0.00011, 3: 0.00031, 4: 0.00019,	1: 0.00011, 2: 0.00014, 3: 0.00020, 4: 0.00016,	AZOBR_p1130098		
AZOLI_p50428		1: 0.00035, 2: 0.00054, 3: 0.00030, 4: 0.00027,	AZOBR_p1130099	2: 0.00006, 3: 0.00006, 4: 0.00006, 5: 0.00006,	1: 0.00010, 2: 0.00006, 3: 0.00008,
AZOLI_p60004	2: 0.00004,		AZOBR_p1130101	1: 0.00008, 4: 0.00008, 5: 0.00011,	1: 0.00015, 3: 0.00017,
AZOLI_p60007	3: 0.00011,	4: 0.00006,	AZOBR_p1130105	3: 0.00010, 4: 0.00008,	1: 0.00034, 2: 0.00033, 3: 0.00049,
AZOLI_p60008	1: 0.00024, 2: 0.00032, 4: 0.00035,	1: 0.00028, 2: 0.00020, 3: 0.00022, 4: 0.00014,	AZOBR_p1130106		1: 0.00005, 2: 0.00007, 3: 0.00005,
AZOLI_p60009	1: 0.00003, 2: 0.00005,	2: 0.00003,	AZOBR_p1130109	1: 0.00102, 2: 0.00168, 3: 0.00202, 4: 0.00163, 5: 0.00154,	1: 0.00161, 2: 0.00217, 3: 0.00152,
AZOLI_p60012		2: 0.00005, 3: 0.00005,	AZOBR_p1130110		3: 0.00044,
AZOLI_p60015	1: 0.00010, 2: 0.00007,	1: 0.00007, 3: 0.00014, 4: 0.00006,	AZOBR_p1130112		3: 0.00008,
AZOLI_p60016		2: 0.00004, 3: 0.00007,	AZOBR_p1130116		3: 0.00007,
AZOLI_p60023	4: 0.00013,	3: 0.00022, 4: 0.00009,	AZOBR_p1130117	3: 0.00002,	
AZOLI_p60034	1: 0.00005, 2: 0.00005,		AZOBR_p1130118	1: 0.00276, 2: 0.00267, 3: 0.00224, 4: 0.00186, 5: 0.00190,	1: 0.00177, 2: 0.00262, 3: 0.00224,
AZOLI_p60035	4: 0.00005,	3: 0.00007, 4: 0.00011,	AZOBR_p1130119		1: 0.00012,
AZOLI_p60036	2: 0.00008,	1: 0.00008,	AZOBR_p1130124	4: 0.00021,	
AZOLI_p60040	2: 0.00007, 3: 0.00035, 4: 0.00009,	1: 0.00011, 2: 0.00010, 4: 0.00006,	AZOBR_p1130125	1: 0.00008, 3: 0.00010, 4: 0.00006, 5: 0.00006,	
AZOLI_p60042	1: 0.00025, 2: 0.00025, 4: 0.00022,	1: 0.00007, 2: 0.00014, 3: 0.00012, 4: 0.00019,	AZOBR_p1130128	1: 0.00079, 2: 0.00124, 3: 0.00105, 4: 0.00158, 5: 0.00115,	1: 0.00056, 2: 0.00026, 3: 0.00082,
AZOLI_p60045	1: 0.00004,	4: 0.00008,	AZOBR_p1130129	1: 0.00522, 2: 0.00514, 3: 0.00418, 4: 0.00422, 5: 0.00793,	1: 0.00295, 2: 0.00215, 3: 0.00249,
AZOLI_p60047		3: 0.00010,	AZOBR_p1130130	2: 0.00005, 4: 0.00008, 5: 0.00006,	2: 0.00004,
AZOLI_p60050		2: 0.00009,	AZOBR_p1130132		3: 0.00018,
AZOLI_p60053		1: 0.00017, 3: 0.00024, 4: 0.00015,	AZOBR_p1130136		2: 0.00033,
AZOLI_p60055		4: 0.00004,	AZOBR_p1130137		3: 0.00007,
AZOLI_p60056	4: 0.00007,	1: 0.00009, 3: 0.00011, 4: 0.00011,	AZOBR_p1130142	3: 0.00011,	
AZOLI_p60059		4: 0.00003,	AZOBR_p1130143	1: 0.00026, 2: 0.00023, 3: 0.00026, 4: 0.00021, 5: 0.00036,	1: 0.00022, 2: 0.00051, 3: 0.00035,
AZOLI_p60075	4: 0.00024,		AZOBR_p1130144		1: 0.00018, 2: 0.00020, 3: 0.00028,
AZOLI_p60076	2: 0.00010, 4: 0.00020,		AZOBR_p1130145		1: 0.00016, 2: 0.00021, 3: 0.00022,

AZOLI_p60084	1: 0.00014,		AZOBR_p1130147		1: 0.00010, 2: 0.00012, 3: 0.00015,
AZOLI_p60087	4: 0.00005,		AZOBR_p1130148		2: 0.00005,
AZOLI_p60091	1: 0.00010,	1: 0.00008, 2: 0.00013, 3: 0.00011, 4: 0.00014,	AZOBR_p1130149	4: 0.00004,	1: 0.00021, 2: 0.00015, 3: 0.00022,
AZOLI_p60094	1: 0.00018, 2: 0.00007, 4: 0.00015,	1: 0.00032, 2: 0.00041, 3: 0.00023, 4: 0.00027,	AZOBR_p1130151		2: 0.00009, 3: 0.00006,
AZOLI_p60095	1: 0.00010, 2: 0.00007, 4: 0.00017,	1: 0.00024, 2: 0.00010, 3: 0.00032, 4: 0.00017,	AZOBR_p1130155	4: 0.00011, 5: 0.00006,	3: 0.00005,
AZOLI_p60101		3: 0.00007, 4: 0.00005,	AZOBR_p1130156		3: 0.00011,
AZOLI_p60105		1: 0.00009, 2: 0.00017, 3: 0.00009, 4: 0.00008,	AZOBR_p1130158		3: 0.00010,
AZOLI_p60109		1: 0.00015, 4: 0.00008,	AZOBR_p1130159		
AZOLI_p60124		3: 0.00010,	AZOBR_p1130161	5: 0.00008,	
AZOLI_p60129		3: 0.00004,	AZOBR_p1130162	1: 0.00029, 3: 0.00041, 4: 0.00030, 5: 0.00016,	1: 0.00009, 2: 0.00021, 3: 0.00024,
AZOLI_p60137		4: 0.00008,	AZOBR_p1130163	1: 0.00012, 2: 0.00011, 3: 0.00017, 4: 0.00019, 5: 0.00044,	1: 0.00013, 2: 0.00006, 3: 0.00017,
AZOLI_p60140		1: 0.00009,	AZOBR_p1130164	4: 0.00010, 5: 0.00007,	1: 0.00006,
AZOLI_p60155	4: 0.00005,	2: 0.00008,	AZOBR_p1130166	1: 0.00013, 3: 0.00022, 4: 0.00027, 5: 0.00018,	1: 0.00044, 2: 0.00072, 3: 0.00077,
AZOLI_p60158	4: 0.00006,		AZOBR_p1130167		2: 0.00011,
AZOLI_p60160		4: 0.00008,	AZOBR_p1130169	1: 0.00411, 2: 0.00603, 3: 0.00516, 4: 0.00535, 5: 0.00733,	1: 0.00497, 2: 0.00543, 3: 0.00526,
AZOLI_p60165	1: 0.00007, 2: 0.00011,	1: 0.00007, 2: 0.00010, 3: 0.00011,	AZOBR_p1130170	1: 0.00014, 3: 0.00005, 4: 0.00006, 5: 0.00006,	2: 0.00006, 3: 0.00003,
AZOLI_p60168	1: 0.00280, 2: 0.00454, 3: 0.00120, 4: 0.00199,	1: 0.00160, 2: 0.00261, 3: 0.00104, 4: 0.00158,	AZOBR_p1130183	4: 0.00007, 5: 0.00005,	3: 0.00008,
AZOLI_p60173	4: 0.00005,	1: 0.00010, 4: 0.00008,	AZOBR_p1130184		
AZOLI_p60182	4: 0.00010,	2: 0.00007, 3: 0.00012, 4: 0.00008,	AZOBR_p1130186		3: 0.00015,
AZOLI_p60192	4: 0.00006,	1: 0.00007, 2: 0.00007,	AZOBR_p1130187		
AZOLI_p60193	2: 0.00013,	1: 0.00016, 2: 0.00018, 3: 0.00017, 4: 0.00013,	AZOBR_p1130192		
AZOLI_p60195	1: 0.00008, 4: 0.00017,	1: 0.00020, 3: 0.00012, 4: 0.00017,	AZOBR_p1130194	1: 0.00089, 2: 0.00066, 3: 0.00089, 4: 0.00036, 5: 0.00015,	1: 0.00038, 2: 0.00049, 3: 0.00042,
AZOLI_p60196	2: 0.00008, 4: 0.00013,	1: 0.00015, 3: 0.00008, 4: 0.00010,	AZOBR_p1130196		2: 0.00004, 3: 0.00003,
AZOLI_p60197	1: 0.00009, 4: 0.00005,	2: 0.00006,	AZOBR_p1130197	1: 0.00138, 2: 0.00067, 3: 0.00104, 4: 0.00116, 5: 0.00053,	1: 0.00020,
AZOLI_p60198		1: 0.00013, 4: 0.00016,	AZOBR_p1130198	1: 0.00062, 2: 0.00070, 3: 0.00065, 4: 0.00065, 5: 0.00051,	1: 0.00030, 2: 0.00043, 3: 0.00033,
AZOLI_p60199	4: 0.00002,	1: 0.00002, 4: 0.00004,	AZOBR_p1130199	1: 0.00057, 2: 0.00082, 3: 0.00046, 4: 0.00075, 5: 0.00052,	1: 0.00044, 2: 0.00035, 3: 0.00035,
AZOLI_p60207		2: 0.00004,	AZOBR_p1130200		
AZOLI_p60210	2: 0.00026, 3: 0.00057, 4: 0.00031,	2: 0.00010, 4: 0.00009,	AZOBR_p1130208		
AZOLI_p60215	1: 0.00022,	1: 0.00033,	AZOBR_p1140002		1: 0.00009,
AZOLI_p60217	4: 0.00066,		AZOBR_p1140003	4: 0.00014, 5: 0.00014,	1: 0.00008, 3: 0.00016,
AZOLI_p60218	1: 0.00044, 2: 0.00111, 3: 0.00103, 4: 0.00247,	1: 0.00186, 2: 0.00208, 3: 0.00276, 4: 0.00197,	AZOBR_p1140004	4: 0.00015,	2: 0.00011, 3: 0.00009,
AZOLI_p60219	1: 0.00004,		AZOBR_p1140005	5: 0.00008,	2: 0.00013, 3: 0.00007,
AZOLI_p60224		3: 0.00022,	AZOBR_p1140008		
AZOLI_p60226	1: 0.00016, 4: 0.00005,	1: 0.00005, 4: 0.00007,	AZOBR_p1140009		
AZOLI_p60230	4: 0.00008,		AZOBR_p1140010	1: 0.00100, 2: 0.00095, 3: 0.00059, 4: 0.00122, 5: 0.00095,	1: 0.00069, 2: 0.00101, 3: 0.00072,
AZOLI_p60233		4: 0.00010,	AZOBR_p1140011		1: 0.00009, 2: 0.00010, 3: 0.00004,
AZOLI_p60234		4: 0.00009,	AZOBR_p1140012		
AZOLI_p60251		1: 0.00018, 3: 0.00013,	AZOBR_p1140013	1: 0.00040, 3: 0.00047, 4: 0.00033, 5: 0.00011,	1: 0.00020, 2: 0.00029, 3: 0.00035,
AZOLI_p60253		2: 0.00002, 4: 0.00003,	AZOBR_p1140016	2: 0.00054, 3: 0.00021,	1: 0.00016, 2: 0.00018,
AZOLI_p60256	4: 0.00008,	1: 0.00023, 2: 0.00013, 4: 0.00016,	AZOBR_p1140019		2: 0.00010, 3: 0.00018,
AZOLI_p60260		3: 0.00002, 4: 0.00002,	AZOBR_p1140020	4: 0.00036,	
AZOLI_p60261	4: 0.00008,	1: 0.00005, 2: 0.00003, 3: 0.00003, 4: 0.00003,	AZOBR_p1140021	3: 0.00008, 4: 0.00007,	1: 0.00013, 2: 0.00011, 3: 0.00009,
AZOLI_p60264	4: 0.00020,	1: 0.00032, 3: 0.00017,	AZOBR_p1140022		
AZOLI_p60265	4: 0.00006,		AZOBR_p1140023	1: 0.00034, 3: 0.00020, 4: 0.00017, 5: 0.00013,	1: 0.00015, 2: 0.00025, 3: 0.00024,
AZOLI_p60266	1: 0.00013, 2: 0.00022, 4: 0.00023,	1: 0.00011, 2: 0.00008, 4: 0.00005,	AZOBR_p1140024	5: 0.00008,	
			AZOBR_p1140042		
			AZOBR_p1140059	4: 0.00009, 5: 0.00009,	1: 0.00008, 2: 0.00023, 3: 0.00020,
			AZOBR_p1140074		
			AZOBR_p1140076		3: 0.00004,
			AZOBR_p1140088		1: 0.00013, 3: 0.00010,
			AZOBR_p1140098		3: 0.00001,
			AZOBR_p1140099		
			AZOBR_p1140101		
			AZOBR_p1140102		
			AZOBR_p1140103		1: 0.00009, 2: 0.00008, 3: 0.00004,
			AZOBR_p1140104	5: 0.00003,	1: 0.00011, 2: 0.00006, 3: 0.00016,
			AZOBR_p1140106	1: 0.00004,	1: 0.00005, 3: 0.00004,
			AZOBR_p1140109		
			AZOBR_p1140110		

			AZOBR_p1140114		1: 0.00012, 2: 0.00009, 3: 0.00019,
			AZOBR_p1140117		
			AZOBR_p1140118		
			AZOBR_p1140119	2: 0.00005, 4: 0.00009, 5: 0.00004,	1: 0.00005, 2: 0.00013, 3: 0.00009,
			AZOBR_p1140120	4: 0.00004,	
			AZOBR_p1140132	3: 0.00005,	
			AZOBR_p1140133	2: 0.00029, 4: 0.00011, 5: 0.00011,	
			AZOBR_p1150011		
			AZOBR_p1150014		
			AZOBR_p1150015		
			AZOBR_p1150016		
			AZOBR_p1150017	1: 0.00041, 3: 0.00015, 4: 0.00032, 5: 0.00016,	1: 0.00034, 2: 0.00051, 3: 0.00069,
			AZOBR_p1150021		1: 0.00017, 2: 0.00019,
			AZOBR_p1160003	1: 0.00012, 2: 0.00022, 3: 0.00021, 4: 0.00040, 5: 0.00028,	2: 0.00009, 3: 0.00006,
			AZOBR_p1160013		1: 0.00053, 2: 0.00068, 3: 0.00084,
			AZOBR_p1160014		
			AZOBR_p1160015		
			AZOBR_p1160017		2: 0.00024,
			AZOBR_p1160021		
			AZOBR_p1160029		
			AZOBR_p1160030		3: 0.00012,
			AZOBR_p1160031		
			AZOBR_p1160038		
			AZOBR_p1160040		
			AZOBR_p1160042		
			AZOBR_p1160043		
			AZOBR_p1160045	1: 0.00101, 2: 0.00165, 3: 0.00121, 4: 0.00138, 5: 0.00185,	1: 0.00356, 2: 0.00276, 3: 0.00256,
			AZOBR_p1160046	1: 0.00020,	2: 0.00030, 3: 0.00013,
			AZOBR_p1160048		
			AZOBR_p1160051		
			AZOBR_p1160052		
			AZOBR_p1160053		
			AZOBR_p1160055		1: 0.00007, 3: 0.00010,
			AZOBR_p1160056		
			AZOBR_p1170004	4: 0.00047, 5: 0.00031,	1: 0.00022, 2: 0.00025, 3: 0.00056,
			AZOBR_p1170006		2: 0.00008, 3: 0.00010,
			AZOBR_p1170013		
			AZOBR_p1170014	3: 0.00006, 5: 0.00007,	
			AZOBR_p1170018	3: 0.00004, 4: 0.00007,	2: 0.00009, 3: 0.00003,
			AZOBR_p1170021	4: 0.00016, 5: 0.00016,	1: 0.00033, 2: 0.00017, 3: 0.00082,
			AZOBR_p1170023	1: 0.00041, 2: 0.00082, 3: 0.00065, 4: 0.00088, 5: 0.00047,	1: 0.00038, 2: 0.00054, 3: 0.00042,
			AZOBR_p1170026	4: 0.00006,	
			AZOBR_p1170027		
			AZOBR_p1170029		2: 0.00023, 3: 0.00020,
			AZOBR_p1170034	2: 0.00079, 3: 0.00061, 4: 0.00086, 5: 0.00056,	1: 0.00046, 2: 0.00013, 3: 0.00061,
			AZOBR_p1170036		1: 0.00006, 2: 0.00019, 3: 0.00008,
			AZOBR_p1170047		
			AZOBR_p1170051	1: 0.00009,	
			AZOBR_p1170056		
			AZOBR_p1170057		
			AZOBR_p1170058		
			AZOBR_p1170059		3: 0.00014,
			AZOBR_p1170066		1: 0.00009, 3: 0.00009,
			AZOBR_p1170069		1: 0.00003, 3: 0.00008,
			AZOBR_p1170073	4: 0.00025, 5: 0.00015,	1: 0.00014, 2: 0.00027, 3: 0.00020,
			AZOBR_p1170077		
			AZOBR_p1170078		3: 0.00013,
			AZOBR_p1170079	4: 0.00040, 5: 0.00028,	3: 0.00025,
			AZOBR_p1170080		1: 0.00080, 2: 0.00053, 3: 0.00104,
			AZOBR_p1170081		
			AZOBR_p1170084		
			AZOBR_p1170085	1: 0.00200, 2: 0.00149, 3: 0.00288, 4: 0.00127, 5: 0.00154,	1: 0.00089, 2: 0.00151, 3: 0.00086,
			AZOBR_p1170086		
			AZOBR_p1170087		
			AZOBR_p1170088	4: 0.00008, 5: 0.00008,	1: 0.00015, 3: 0.00040,
			AZOBR_p1170089	1: 0.00019,	1: 0.00012, 3: 0.00012,
			AZOBR_p1170091	3: 0.00014, 4: 0.00008, 5: 0.00008,	
			AZOBR_p1170092	1: 0.00087, 2: 0.00041, 3: 0.00089, 4: 0.00048, 5: 0.00024,	1: 0.00015, 2: 0.00042, 3: 0.00014,
			AZOBR_p1170093		3: 0.00005,
			AZOBR_p1170094		
			AZOBR_p1180006	1: 0.00070, 2: 0.00099, 3: 0.00071, 4: 0.00144, 5: 0.00120,	1: 0.00054, 2: 0.00071, 3: 0.00056,
			AZOBR_p1180007		1: 0.00013,

			AZOBR_p1180010		3: 0.00005,
			AZOBR_p1180012		
			AZOBR_p1180013		
			AZOBR_p1180014		1: 0.00003, 2: 0.00002, 3: 0.00002,
			AZOBR_p1180016	3: 0.00018, 4: 0.00015, 5: 0.00015,	1: 0.00021, 3: 0.00027,
			AZOBR_p1180019	1: 0.00278, 2: 0.00308, 3: 0.00223, 4: 0.00273, 5: 0.00334,	1: 0.00325, 2: 0.00243, 3: 0.00251,
			AZOBR_p1180022	4: 0.00018, 5: 0.00027,	1: 0.00126, 2: 0.00086, 3: 0.00081,
			AZOBR_p1180025		
			AZOBR_p1180027	1: 0.00017, 2: 0.00029, 3: 0.00026, 4: 0.00036, 5: 0.00023,	1: 0.00044, 2: 0.00022, 3: 0.00029,
			AZOBR_p1180028		
			AZOBR_p1180029	4: 0.00009, 5: 0.00009,	3: 0.00008,
			AZOBR_p1180030		
			AZOBR_p1180032	1: 0.00011, 3: 0.00007, 4: 0.00018, 5: 0.00010,	1: 0.00004, 2: 0.00006, 3: 0.00005,
			AZOBR_p1180036	1: 0.00047, 2: 0.00095, 3: 0.00077, 4: 0.00112, 5: 0.00077,	1: 0.00132, 2: 0.00106, 3: 0.00147,
			AZOBR_p1180037	1: 0.00159, 2: 0.00099, 3: 0.00128, 4: 0.00116, 5: 0.00153,	1: 0.00130, 2: 0.00164, 3: 0.00178,
			AZOBR_p1180037	1: 0.00070, 2: 0.00062, 3: 0.00060, 4: 0.00076, 5: 0.00131,	1: 0.00167, 2: 0.00197, 3: 0.00286,
			AZOBR_p210002	2: 0.00005, 4: 0.00009, 5: 0.00007,	1: 0.00008, 3: 0.00026,
			AZOBR_p210004		
			AZOBR_p210005		1: 0.00009,
			AZOBR_p210006		
			AZOBR_p210007		3: 0.00014,
			AZOBR_p210008	4: 0.00004,	1: 0.00007, 3: 0.00010,
			AZOBR_p210009		1: 0.00007, 2: 0.00006, 3: 0.00012,
			AZOBR_p210010	2: 0.00025, 3: 0.00024, 4: 0.00012,	1: 0.00011, 2: 0.00024, 3: 0.00024,
			AZOBR_p210012		1: 0.00010, 3: 0.00023,
			AZOBR_p210013		1: 0.00004, 2: 0.00006, 3: 0.00005,
			AZOBR_p210016		2: 0.00008, 3: 0.00005,
			AZOBR_p210018	1: 0.00017, 2: 0.00012, 3: 0.00020, 4: 0.00014, 5: 0.00005,	1: 0.00061, 2: 0.00077, 3: 0.00061,
			AZOBR_p210022		
			AZOBR_p210024	1: 0.00029, 3: 0.00025, 4: 0.00010,	1: 0.00022, 2: 0.00061, 3: 0.00021,
			AZOBR_p210025		
			AZOBR_p210027	4: 0.00006,	3: 0.00005,
			AZOBR_p210050		3: 0.00023,
			AZOBR_p210070		
			AZOBR_p210091	1: 0.00007, 2: 0.00015, 3: 0.00012, 4: 0.00031, 5: 0.00031,	1: 0.00004, 3: 0.00013,
			AZOBR_p210097	1: 0.00020, 2: 0.00014, 3: 0.00017, 4: 0.00025, 5: 0.00025,	1: 0.00015, 2: 0.00029, 3: 0.00020,
			AZOBR_p210103		3: 0.00011,
			AZOBR_p210104		1: 0.00007,
			AZOBR_p210106		1: 0.00021,
			AZOBR_p210107		
			AZOBR_p210115	1: 0.00055, 2: 0.00059, 3: 0.00038, 4: 0.00069, 5: 0.00031,	1: 0.00128, 3: 0.00096,
			AZOBR_p210116	1: 0.00055, 2: 0.00049, 3: 0.00067, 4: 0.00043, 5: 0.00044,	1: 0.00061, 2: 0.00075, 3: 0.00054,
			AZOBR_p210117		
			AZOBR_p210122		
			AZOBR_p210129		1: 0.00008, 2: 0.00009, 3: 0.00013,
			AZOBR_p210130	5: 0.00021,	3: 0.00019,
			AZOBR_p210134		
			AZOBR_p210135		
			AZOBR_p210136		1: 0.00118, 2: 0.00161, 3: 0.00135,
			AZOBR_p210140		
			AZOBR_p210148	1: 0.00032, 2: 0.00029, 3: 0.00035, 4: 0.00024, 5: 0.00022,	
			AZOBR_p210167		
			AZOBR_p210169		1: 0.00012,
			AZOBR_p210180		3: 0.00011,
			AZOBR_p210181		
			AZOBR_p210182		1: 0.00016, 2: 0.00010, 3: 0.00013,
			AZOBR_p210185		
			AZOBR_p210197	3: 0.00004, 4: 0.00003,	
			AZOBR_p220010	1: 0.00973, 2: 0.01282, 3: 0.00920, 4: 0.01117, 5: 0.01161,	1: 0.01157, 2: 0.00821, 3: 0.01046,
			AZOBR_p220011	1: 0.00161, 2: 0.00175, 3: 0.00137, 4: 0.00188, 5: 0.00197,	1: 0.00072, 2: 0.00063, 3: 0.00116,
			AZOBR_p220033	1: 0.00030, 2: 0.00011, 4: 0.00046, 5: 0.00042,	1: 0.00043, 2: 0.00053, 3: 0.00053,
			AZOBR_p220034		1: 0.00015,
			AZOBR_p220036	5: 0.00041,	
			AZOBR_p220037		
			AZOBR_p220038	4: 0.00001,	3: 0.00001,

			AZOBR_p220040	1: 0.00005, 4: 0.00003, 5: 0.00005,	
			AZOBR_p220043		3: 0.00017,
			AZOBR_p220044		
			AZOBR_p220049	5: 0.00006,	3: 0.00006,
			AZOBR_p220076	1: 0.00282, 2: 0.00231, 3: 0.00258, 4: 0.00295, 5: 0.00173,	1: 0.00033, 2: 0.00038, 3: 0.00058,
			AZOBR_p220077		
			AZOBR_p220082	1: 0.00235, 2: 0.00300, 3: 0.00265, 4: 0.00254, 5: 0.00314,	
			AZOBR_p220084	1: 0.00015, 2: 0.00013, 4: 0.00015, 5: 0.00013,	
			AZOBR_p220087	1: 0.00011, 2: 0.00006, 3: 0.00009, 4: 0.00004,	2: 0.00005, 3: 0.00004,
			AZOBR_p220088	1: 0.00053, 2: 0.00047, 3: 0.00039, 4: 0.00068, 5: 0.00068,	1: 0.00039, 2: 0.00033, 3: 0.00038,
			AZOBR_p220089		3: 0.00009,
			AZOBR_p220094		1: 0.00011, 2: 0.00008, 3: 0.00024,
			AZOBR_p220099		
			AZOBR_p220101		
			AZOBR_p220102	3: 0.00013, 4: 0.00017, 5: 0.00010,	3: 0.00015,
			AZOBR_p220105	1: 0.00009, 3: 0.00011, 4: 0.00009,	1: 0.00057, 2: 0.00064, 3: 0.00063,
			AZOBR_p220106		2: 0.00003,
			AZOBR_p220110	2: 0.00009, 3: 0.00017, 4: 0.00020, 5: 0.00020,	1: 0.00009, 3: 0.00015,
			AZOBR_p220111		
			AZOBR_p220112		
			AZOBR_p220115		2: 0.00013, 3: 0.00012,
			AZOBR_p230002		
			AZOBR_p230016		
			AZOBR_p230018		
			AZOBR_p230036		
			AZOBR_p230046		3: 0.00009,
			AZOBR_p230051	1: 0.00027, 2: 0.00024, 3: 0.00030, 4: 0.00015, 5: 0.00015,	1: 0.00009, 2: 0.00039, 3: 0.00019,
			AZOBR_p230063		
			AZOBR_p230066		
			AZOBR_p230087	1: 0.00159, 2: 0.00174, 3: 0.00174, 4: 0.00141, 5: 0.00068,	1: 0.00063, 2: 0.00077, 3: 0.00052,
			AZOBR_p230091	3: 0.00016,	
			AZOBR_p230092	1: 0.00035, 4: 0.00016,	2: 0.00026,
			AZOBR_p230093		
			AZOBR_p230094		
			AZOBR_p250007		
			AZOBR_p250011		
			AZOBR_p270012	2: 0.00009, 3: 0.00021,	1: 0.00013, 2: 0.00022, 3: 0.00009,
			AZOBR_p270033		2: 0.00007,
			AZOBR_p270039		
			AZOBR_p270057	5: 0.00004,	1: 0.00003, 3: 0.00008,
			AZOBR_p270059	1: 0.00010, 3: 0.00004, 4: 0.00023, 5: 0.00009,	1: 0.00026, 2: 0.00022, 3: 0.00033,
			AZOBR_p270061		
			AZOBR_p270080		
			AZOBR_p270113	3: 0.00007,	
			AZOBR_p270126		
			AZOBR_p270130	3: 0.00005,	
			AZOBR_p270132	1: 0.00076, 2: 0.00052, 3: 0.00072, 4: 0.00018, 5: 0.00035,	1: 0.00044, 2: 0.00043, 3: 0.00058,
			AZOBR_p270133	2: 0.00004, 4: 0.00003, 5: 0.00002,	1: 0.00003, 2: 0.00003, 3: 0.00006,
			AZOBR_p270135		
			AZOBR_p270144		
			AZOBR_p270147		1: 0.00019, 2: 0.00005, 3: 0.00012,
			AZOBR_p270149		1: 0.00007, 2: 0.00002, 3: 0.00005,
			AZOBR_p270150		
			AZOBR_p270151		1: 0.00010, 3: 0.00017,
			AZOBR_p270152		
			AZOBR_p270154		
			AZOBR_p270158	5: 0.00014,	
			AZOBR_p270160		3: 0.00006,
			AZOBR_p270161		
			AZOBR_p270162		
			AZOBR_p270171	1: 0.00020, 4: 0.00009,	1: 0.00017, 2: 0.00015, 3: 0.00033,
			AZOBR_p270174		
			AZOBR_p270177	4: 0.00004,	
			AZOBR_p270187		1: 0.00016, 2: 0.00007, 3: 0.00031,
			AZOBR_p270203		1: 0.00002,
			AZOBR_p270222		1: 0.00008, 3: 0.00016,
			AZOBR_p270223		2: 0.00009,
			AZOBR_p270224		2: 0.00021, 3: 0.00014,
			AZOBR_p270225		
			AZOBR_p270229	1: 0.00011,	

			AZOBR_p270230	1: 0.00011, 4: 0.00016, 5: 0.00012,	1: 0.00015, 3: 0.00014,
			AZOBR_p270232		
			AZOBR_p270244		1: 0.00020, 2: 0.00016, 3: 0.00043,
			AZOBR_p270247	3: 0.00011, 5: 0.00015,	1: 0.00023, 2: 0.00029, 3: 0.00058,
			AZOBR_p270279		3: 0.00019,
			AZOBR_p270280	3: 0.00005, 4: 0.00011, 5: 0.00009,	1: 0.00009, 2: 0.00012, 3: 0.00015,
			AZOBR_p280010	1: 0.00549, 2: 0.00318, 3: 0.00392, 4: 0.00447, 5: 0.00427,	1: 0.00318, 2: 0.00279, 3: 0.00359,
			AZOBR_p280013		1: 0.00029, 2: 0.00059, 3: 0.00039,
			AZOBR_p280015	4: 0.00014, 5: 0.00007,	3: 0.00010,
			AZOBR_p280016	4: 0.00006,	1: 0.00014, 2: 0.00006, 3: 0.00013,
			AZOBR_p280017	2: 0.00049, 3: 0.00059,	
			AZOBR_p280024	1: 0.00044, 2: 0.00020, 3: 0.00025, 4: 0.00026, 5: 0.00036,	1: 0.00009, 2: 0.00049,
			AZOBR_p280026	4: 0.00004,	1: 0.00008, 3: 0.00009,
			AZOBR_p280027	2: 0.00054, 3: 0.00022, 5: 0.00012,	1: 0.00011, 2: 0.00013, 3: 0.00016,
			AZOBR_p280028		
			AZOBR_p280029		3: 0.00007,
			AZOBR_p280033	1: 0.00632, 2: 0.00499, 3: 0.00538, 4: 0.00484, 5: 0.00376,	1: 0.00709, 2: 0.00697, 3: 0.00422,
			AZOBR_p280034	1: 0.00115, 2: 0.00109, 3: 0.00070, 4: 0.00152, 5: 0.00136,	1: 0.00047, 2: 0.00066, 3: 0.00066,
			AZOBR_p280035		
			AZOBR_p280036	1: 0.00033, 2: 0.00046, 3: 0.00073, 4: 0.00021, 5: 0.00026,	2: 0.00005, 3: 0.00016,
			AZOBR_p280039	4: 0.00010, 5: 0.00015,	1: 0.00024, 2: 0.00011, 3: 0.00023,
			AZOBR_p280040		
			AZOBR_p280043	1: 0.00057, 2: 0.00028, 3: 0.00070, 4: 0.00031, 5: 0.00022,	2: 0.00023,
			AZOBR_p280048		
			AZOBR_p280052		
			AZOBR_p280056	4: 0.00020, 5: 0.00051,	1: 0.00028, 3: 0.00037,
			AZOBR_p280064	1: 0.00024, 2: 0.00021, 3: 0.00040, 4: 0.00049, 5: 0.00057,	1: 0.00076, 2: 0.00026, 3: 0.00095,
			AZOBR_p280067		1: 0.00007,
			AZOBR_p280071	4: 0.00009, 5: 0.00028,	1: 0.00009, 2: 0.00010, 3: 0.00013,
			AZOBR_p280077		
			AZOBR_p280093		1: 0.00011, 2: 0.00009, 3: 0.00031,
			AZOBR_p280099	2: 0.00009, 4: 0.00018, 5: 0.00016,	2: 0.00005, 3: 0.00008,
			AZOBR_p280100	4: 0.00014, 5: 0.00007,	
			AZOBR_p280103	4: 0.00022,	1: 0.00028, 3: 0.00033,
			AZOBR_p280105	1: 0.00010, 2: 0.00007, 3: 0.00011, 4: 0.00019, 5: 0.00012,	1: 0.00013, 2: 0.00004, 3: 0.00014,
			AZOBR_p280106		1: 0.00025,
			AZOBR_p280107		3: 0.00003,
			AZOBR_p280112		
			AZOBR_p280114		
			AZOBR_p280115		
			AZOBR_p280116		3: 0.00008,
			AZOBR_p280118	4: 0.00009,	3: 0.00005,
			AZOBR_p280120		
			AZOBR_p280122	1: 0.00044, 2: 0.00023, 3: 0.00030, 4: 0.00091, 5: 0.00061,	1: 0.00079, 2: 0.00058, 3: 0.00098,
			AZOBR_p280124		
			AZOBR_p280126		2: 0.00009, 3: 0.00015,
			AZOBR_p280132		
			AZOBR_p280133		
			AZOBR_p280137	1: 0.00169, 2: 0.00190, 3: 0.00167, 4: 0.00064, 5: 0.00155,	1: 0.00090, 2: 0.00150, 3: 0.00111,
			AZOBR_p280141	2: 0.00008, 4: 0.00007, 5: 0.00007,	1: 0.00015, 2: 0.00010, 3: 0.00012,
			AZOBR_p280144	3: 0.00015, 4: 0.00016, 5: 0.00016,	1: 0.00026, 2: 0.00029, 3: 0.00032,
			AZOBR_p310004		
			AZOBR_p310028		3: 0.00004,
			AZOBR_p310044	3: 0.00007,	
			AZOBR_p310057	4: 0.00005,	
			AZOBR_p310071	5: 0.00008,	1: 0.00012, 3: 0.00007,
			AZOBR_p310073	2: 0.00007, 3: 0.00007, 4: 0.00009,	
			AZOBR_p310075	1: 0.00136, 2: 0.00053, 3: 0.00065, 4: 0.00045, 5: 0.00026,	1: 0.00063, 2: 0.00087, 3: 0.00041,
			AZOBR_p310089		
			AZOBR_p310110		2: 0.00006,
			AZOBR_p310123		
			AZOBR_p310127	4: 0.00006, 5: 0.00006,	2: 0.00006, 3: 0.00005,
			AZOBR_p310134		
			AZOBR_p310137	3: 0.00008,	
			AZOBR_p310141	1: 0.00008,	
			AZOBR_p310162		
			AZOBR_p310167	1: 0.00107, 2: 0.00120, 3: 0.00112,	1: 0.00050, 2: 0.00033,

				4: 0.00090, 5: 0.00076,	3: 0.00084,
			AZOBR_p310169	3: 0.00014,	
			AZOBR_p310174		1: 0.00019, 2: 0.00019, 3: 0.00014,
			AZOBR_p310177	5: 0.00013,	
			AZOBR_p310187		
			AZOBR_p310190		
			AZOBR_p310197		1: 0.00021, 2: 0.00013, 3: 0.00018,
			AZOBR_p310203		
			AZOBR_p310205	4: 0.00090, 5: 0.00030,	
			AZOBR_p310206	1: 0.00091, 2: 0.00080, 3: 0.00086, 4: 0.00229, 5: 0.00195,	1: 0.00058, 2: 0.00066, 3: 0.00157,
			AZOBR_p310211		
			AZOBR_p310213		
			AZOBR_p310214		1: 0.00007, 3: 0.00014,
			AZOBR_p310215		1: 0.00037, 2: 0.00037, 3: 0.00045,
			AZOBR_p310232		
			AZOBR_p310251	1: 0.00009,	1: 0.00014, 2: 0.00010, 3: 0.00022,
			AZOBR_p310255		
			AZOBR_p310257		1: 0.00062, 3: 0.00038,
			AZOBR_p310258		1: 0.00024,
			AZOBR_p310259		1: 0.00008, 3: 0.00008,
			AZOBR_p310260		
			AZOBR_p310267	1: 0.00034, 2: 0.00011, 3: 0.00033, 4: 0.00044, 5: 0.00026,	1: 0.00027, 2: 0.00056, 3: 0.00048,
			AZOBR_p310268	2: 0.00025,	
			AZOBR_p310271		2: 0.00010,
			AZOBR_p310277		
			AZOBR_p310279		
			AZOBR_p310336	2: 0.00010,	
			AZOBR_p330015		2: 0.00006,
			AZOBR_p330020		2: 0.00003,
			AZOBR_p330026		2: 0.00010, 3: 0.00008,
			AZOBR_p330032		
			AZOBR_p330044		
			AZOBR_p330047		
			AZOBR_p330048		
			AZOBR_p330049		
			AZOBR_p330050		1: 0.00007, 2: 0.00006, 3: 0.00008,
			AZOBR_p330053		3: 0.00005,
			AZOBR_p330060		3: 0.00008,
			AZOBR_p330069		1: 0.00024, 3: 0.00010,
			AZOBR_p330074		1: 0.00006, 2: 0.00007, 3: 0.00014,
			AZOBR_p330076	4: 0.00009,	2: 0.00005,
			AZOBR_p330080		1: 0.00018, 3: 0.00009,
			AZOBR_p330082		2: 0.00027, 3: 0.00023,
			AZOBR_p330087		2: 0.00011, 3: 0.00009,
			AZOBR_p330089		1: 0.00062, 2: 0.00030, 3: 0.00071,
			AZOBR_p330094		
			AZOBR_p330126		
			AZOBR_p330143		3: 0.00009,
			AZOBR_p330151		
			AZOBR_p330152	4: 0.00040, 5: 0.00013,	
			AZOBR_p330155	1: 0.00017, 3: 0.00029, 4: 0.00018,	
			AZOBR_p330159	4: 0.00025,	1: 0.00069, 2: 0.00017, 3: 0.00133,
			AZOBR_p340002	2: 0.00009, 3: 0.00017,	
			AZOBR_p340004	1: 0.00020, 2: 0.00028, 3: 0.00046, 4: 0.00051, 5: 0.00042,	1: 0.00057, 2: 0.00067, 3: 0.00063,
			AZOBR_p340005		
			AZOBR_p340006		
			AZOBR_p340008	1: 0.00004,	
			AZOBR_p340061		
			AZOBR_p340062		1: 0.00006,
			AZOBR_p340072		
			AZOBR_p340099		1: 0.00019, 3: 0.00012,
			AZOBR_p340166	1: 0.00025, 4: 0.00028, 5: 0.00011,	3: 0.00021,
			AZOBR_p340169		3: 0.00010,
			AZOBR_p340215		
			AZOBR_p340220		
			AZOBR_p340221		
			AZOBR_p340221		
			AZOBR_p350001	1: 0.00030, 3: 0.00025, 4: 0.00031, 5: 0.00041,	1: 0.00022, 2: 0.00011, 3: 0.00037,
			AZOBR_p350036	4: 0.00007, 5: 0.00019,	
			AZOBR_p350058	4: 0.00014, 5: 0.00011,	2: 0.00011, 3: 0.00010,
			AZOBR_p350059	5: 0.00006,	
			AZOBR_p350061		
			AZOBR_p350063	1: 0.00031, 2: 0.00037, 3: 0.00027,	
			AZOBR_p350084	1: 0.00024, 4: 0.00012, 5: 0.00016,	
			AZOBR_p350085		
			AZOBR_p410001		

			AZOBR_p410006		
			AZOBR_p410007		3: 0.00005,
			AZOBR_p410021	1: 0.00390, 2: 0.00290, 3: 0.00288, 4: 0.00214, 5: 0.00191,	1: 0.00205, 2: 0.00267, 3: 0.00196,
			AZOBR_p410022	1: 0.00105, 2: 0.00221, 3: 0.00159, 4: 0.00157, 5: 0.00177,	1: 0.00198, 2: 0.00183, 3: 0.00156,
			AZOBR_p410029		3: 0.00005,
			AZOBR_p410040	1: 0.00011, 4: 0.00013, 5: 0.00005,	3: 0.00005,
			AZOBR_p410042		
			AZOBR_p410049		2: 0.00015,
			AZOBR_p410063		
			AZOBR_p410074	3: 0.00021, 4: 0.00022,	2: 0.00012, 3: 0.00010,
			AZOBR_p410076		2: 0.00006, 3: 0.00007,
			AZOBR_p410085	4: 0.00009,	1: 0.00017, 2: 0.00015, 3: 0.00008,
			AZOBR_p410086	1: 0.00006, 2: 0.00010, 3: 0.00007, 4: 0.00008, 5: 0.00010,	3: 0.00004,
			AZOBR_p410087	4: 0.00013, 5: 0.00020,	
			AZOBR_p410090		1: 0.00069, 3: 0.00067,
			AZOBR_p410091		
			AZOBR_p410098	1: 0.00029, 2: 0.00051, 3: 0.00073, 4: 0.00010,	1: 0.00032, 3: 0.00022,
			AZOBR_p410099	1: 0.00068, 2: 0.00060, 3: 0.00116, 4: 0.00084, 5: 0.00103,	1: 0.00183, 2: 0.00138, 3: 0.00169,
			AZOBR_p410100	1: 0.00025, 2: 0.00015, 3: 0.00025, 4: 0.00012, 5: 0.00017,	1: 0.00013, 2: 0.00015, 3: 0.00029,
			AZOBR_p410101	3: 0.00037,	1: 0.00056, 2: 0.00079,
			AZOBR_p410103		
			AZOBR_p420001	1: 0.00005, 4: 0.00003,	
			AZOBR_p420006		3: 0.00017,
			AZOBR_p420012		
			AZOBR_p420020	3: 0.00016, 4: 0.00010,	3: 0.00007,
			AZOBR_p420023		3: 0.00005,
			AZOBR_p420024	1: 0.00297, 2: 0.00404, 3: 0.00305, 4: 0.00212, 5: 0.00337,	1: 0.00403, 2: 0.00348, 3: 0.00318,
			AZOBR_p420025	1: 0.00006,	1: 0.00004, 2: 0.00004, 3: 0.00004,
			AZOBR_p430003	1: 0.00003, 3: 0.00003, 4: 0.00002, 5: 0.00002,	1: 0.00003, 2: 0.00004, 3: 0.00007,
			AZOBR_p430004		
			AZOBR_p430005	3: 0.00024, 4: 0.00040,	3: 0.00027,
			AZOBR_p430006	1: 0.00014, 2: 0.00013, 3: 0.00013, 4: 0.00004, 5: 0.00005,	1: 0.00004, 2: 0.00008, 3: 0.00004,
			AZOBR_p430008		1: 0.00004,
			AZOBR_p430009		1: 0.00002,
			AZOBR_p430010		
			AZOBR_p430031	4: 0.00004,	
			AZOBR_p430035	1: 0.00016, 3: 0.00019, 4: 0.00009,	1: 0.00010, 2: 0.00019, 3: 0.00020,
			AZOBR_p430038	2: 0.00017, 4: 0.00013,	1: 0.00008,
			AZOBR_p430039	1: 0.00041, 3: 0.00028, 4: 0.00011,	
			AZOBR_p430041		
			AZOBR_p430046	1: 0.00006,	1: 0.00009, 2: 0.00011, 3: 0.00007,
			AZOBR_p430050		
			AZOBR_p430053	1: 0.00098, 2: 0.00096, 3: 0.00139, 4: 0.00105, 5: 0.00113,	1: 0.00133, 2: 0.00183, 3: 0.00115,
			AZOBR_p430054		
			AZOBR_p430056	4: 0.00027,	
			AZOBR_p430058	1: 0.00031, 2: 0.00022, 3: 0.00032, 4: 0.00013, 5: 0.00017,	1: 0.00016, 2: 0.00018, 3: 0.00023,
			AZOBR_p430060		3: 0.00007,
			AZOBR_p430062	1: 0.00014, 2: 0.00038, 3: 0.00026, 4: 0.00046, 5: 0.00036,	1: 0.00069, 2: 0.00082, 3: 0.00085,
			AZOBR_p430065	4: 0.00053,	1: 0.00033, 2: 0.00037, 3: 0.00032,
			AZOBR_p440007		3: 0.00007,
			AZOBR_p440013		
			AZOBR_p440014	1: 0.00053, 2: 0.00071, 3: 0.00041, 4: 0.00040, 5: 0.00059,	1: 0.00130, 2: 0.00082, 3: 0.00106,
			AZOBR_p440018	1: 0.00431, 2: 0.00528, 3: 0.00455, 4: 0.00330, 5: 0.00435,	1: 0.00925, 2: 0.00814, 3: 0.00699,
			AZOBR_p440019	1: 0.00151, 2: 0.00203, 3: 0.00160, 4: 0.00146, 5: 0.00159,	1: 0.00701, 2: 0.01026, 3: 0.00530,
			AZOBR_p440026	1: 0.00218, 2: 0.00144, 3: 0.00149, 4: 0.00137, 5: 0.00153,	1: 0.00089, 2: 0.00078, 3: 0.00099,
			AZOBR_p440030		2: 0.00003,
			AZOBR_p440033		
			AZOBR_p440034	1: 0.00005, 2: 0.00008, 3: 0.00005, 4: 0.00005, 5: 0.00005,	1: 0.00003, 2: 0.00003, 3: 0.00011,
			AZOBR_p440042	3: 0.00011,	
			AZOBR_p440043		3: 0.00030,
			AZOBR_p440053		3: 0.00015,
			AZOBR_p440054		3: 0.00009,
			AZOBR_p440060		1: 0.00017, 2: 0.00028, 3: 0.00010,
			AZOBR_p440062	3: 0.00008,	
			AZOBR_p440063		
			AZOBR_p440069		

			AZOBR_p440070		1: 0.00009, 2: 0.00010, 3: 0.00006,
			AZOBR_p440071		2: 0.00013,
			AZOBR_p440072		
			AZOBR_p440073		
			AZOBR_p440083	1: 0.00182, 2: 0.00118, 3: 0.00110, 4: 0.00100, 5: 0.00103,	1: 0.00109, 2: 0.00100, 3: 0.00060,
			AZOBR_p440088	2: 0.00006, 4: 0.00007, 5: 0.00012,	1: 0.00011, 2: 0.00005, 3: 0.00019,
			AZOBR_p440089	1: 0.00005,	2: 0.00004, 3: 0.00005,
			AZOBR_p440091		
			AZOBR_p440094	1: 0.00015, 4: 0.00015, 5: 0.00015,	1: 0.00023, 2: 0.00016, 3: 0.00036,
			AZOBR_p440095	1: 0.00024, 2: 0.00032, 3: 0.00010, 4: 0.00059, 5: 0.00042,	1: 0.00067, 2: 0.00054, 3: 0.00099,
			AZOBR_p440099	2: 0.00007, 3: 0.00005, 4: 0.00021, 5: 0.00028,	1: 0.00050, 2: 0.00030, 3: 0.00064,
			AZOBR_p440101	1: 0.00074, 2: 0.00049, 3: 0.00031, 4: 0.00083, 5: 0.00128,	1: 0.00095, 2: 0.00108, 3: 0.00149,
			AZOBR_p440102		3: 0.00012,
			AZOBR_p440103	1: 0.00028, 2: 0.00025, 3: 0.00034, 4: 0.00020, 5: 0.00012,	1: 0.00011, 2: 0.00008, 3: 0.00011,
			AZOBR_p440104	2: 0.00037, 3: 0.00072, 4: 0.00029, 5: 0.00058,	2: 0.00046, 3: 0.00052,
			AZOBR_p440105		
			AZOBR_p440108	3: 0.00049,	
			AZOBR_p440110		
			AZOBR_p440112	1: 0.00027, 2: 0.00019, 3: 0.00015, 4: 0.00027, 5: 0.00015,	1: 0.00004, 2: 0.00009, 3: 0.00009,
			AZOBR_p440113		1: 0.00046, 2: 0.00040, 3: 0.00051,
			AZOBR_p440118	4: 0.00005,	
			AZOBR_p440120		1: 0.00007, 2: 0.00008, 3: 0.00007,
			AZOBR_p440122		
			AZOBR_p440124		
			AZOBR_p440127		2: 0.00010,
			AZOBR_p440128	1: 0.00035, 2: 0.00106, 3: 0.00073, 4: 0.00104, 5: 0.00111,	1: 0.00087, 2: 0.00084, 3: 0.00094,
			AZOBR_p440132	4: 0.00016, 5: 0.00008,	1: 0.00027, 2: 0.00039, 3: 0.00028,
			AZOBR_p440139	1: 0.00057, 2: 0.00031, 3: 0.00037, 4: 0.00039, 5: 0.00015,	1: 0.00017, 2: 0.00019, 3: 0.00011,
			AZOBR_p440141	1: 0.00049, 2: 0.00034, 3: 0.00048, 4: 0.00045, 5: 0.00037,	1: 0.00058, 2: 0.00062, 3: 0.00080,
			AZOBR_p440144		1: 0.00025, 2: 0.00035, 3: 0.00048,
			AZOBR_p440146		
			AZOBR_p440148	2: 0.00007,	
			AZOBR_p440149	3: 0.00019, 4: 0.00024,	
			AZOBR_p440152	1: 0.00084, 2: 0.00078, 3: 0.00072, 4: 0.00051, 5: 0.00087,	1: 0.00015, 2: 0.00024, 3: 0.00041,
			AZOBR_p440153	1: 0.00429, 2: 0.00448, 3: 0.00274, 4: 0.00227, 5: 0.00286,	1: 0.00362, 2: 0.00312, 3: 0.00222,
			AZOBR_p440154	2: 0.00013, 3: 0.00032, 4: 0.00026, 5: 0.00016,	1: 0.00015, 2: 0.00022, 3: 0.00024,
			AZOBR_p440158		1: 0.00049, 2: 0.00059, 3: 0.00070,
			AZOBR_p440163		1: 0.00036, 2: 0.00032, 3: 0.00038,
			AZOBR_p440175		2: 0.00005, 3: 0.00004,
			AZOBR_p440178	4: 0.00005,	3: 0.00005,
			AZOBR_p440179		
			AZOBR_p440180	1: 0.00010, 2: 0.00009, 4: 0.00007,	3: 0.00013,
			AZOBR_p440187	1: 0.00015, 2: 0.00007, 3: 0.00016, 4: 0.00028, 5: 0.00018,	1: 0.00017, 2: 0.00011, 3: 0.00033,
			AZOBR_p450004	1: 0.00011, 3: 0.00012, 4: 0.00015,	
			AZOBR_p450005		
			AZOBR_p450012	1: 0.00009, 3: 0.00015, 4: 0.00009, 5: 0.00006,	1: 0.00020, 2: 0.00019, 3: 0.00036,
			AZOBR_p460001		
			AZOBR_p460002	3: 0.00014, 4: 0.00026, 5: 0.00022,	1: 0.00045, 2: 0.00024, 3: 0.00044,
			AZOBR_p460005	3: 0.00005, 5: 0.00004,	
			AZOBR_p460010		
			AZOBR_p460011		1: 0.00011, 3: 0.00014,
			AZOBR_p460012		1: 0.00050, 3: 0.00073,
			AZOBR_p460018		
			AZOBR_p460023	1: 0.00151, 2: 0.00095, 3: 0.00174, 4: 0.00096, 5: 0.00067,	1: 0.00048, 2: 0.00102, 3: 0.00047,
			AZOBR_p460025		3: 0.00007,
			AZOBR_p460040	1: 0.00019, 2: 0.00017, 3: 0.00021, 4: 0.00030,	3: 0.00008,
			AZOBR_p460042		
			AZOBR_p460044		
			AZOBR_p460045		
			AZOBR_p460046		
			AZOBR_p460061		1: 0.00002,
			AZOBR_p460065	1: 0.00045, 2: 0.00025, 3: 0.00042,	1: 0.00005, 2: 0.00012,

				4: 0.00023,	
			AZOBR_p460066		
			AZOBR_p460067	1: 0.00011, 2: 0.00019, 3: 0.00016, 4: 0.00009, 5: 0.00004,	2: 0.00008, 3: 0.00007,
			AZOBR_p460068	1: 0.00068, 3: 0.00044, 5: 0.00024,	
			AZOBR_p460070		2: 0.00006,
			AZOBR_p460071	3: 0.00023, 4: 0.00014,	1: 0.00025, 2: 0.00020, 3: 0.00032,
			AZOBR_p460073	1: 0.00140, 2: 0.00099, 3: 0.00128, 4: 0.00120, 5: 0.00094,	1: 0.00188, 2: 0.00141, 3: 0.00144,
			AZOBR_p460074	1: 0.00015, 2: 0.00007, 4: 0.00018, 5: 0.00021,	1: 0.00024, 2: 0.00024, 3: 0.00035,
			AZOBR_p460092		2: 0.00026, 3: 0.00038,
			AZOBR_p470004	1: 0.00023, 3: 0.00020, 4: 0.00032,	1: 0.00052, 2: 0.00017, 3: 0.00094,
			AZOBR_p470012		
			AZOBR_p470013		
			AZOBR_p470015		
			AZOBR_p470016	1: 0.00117, 2: 0.00169, 3: 0.00133, 4: 0.00150, 5: 0.00150,	1: 0.00242, 2: 0.00225, 3: 0.00192,
			AZOBR_p470017		
			AZOBR_p470020		
			AZOBR_p470021		1: 0.00004, 2: 0.00006, 3: 0.00014,
			AZOBR_p470024		3: 0.00004,
			AZOBR_p470034		1: 0.00005, 2: 0.00007,
			AZOBR_p470039	1: 0.00029, 2: 0.00015, 3: 0.00024, 4: 0.00016, 5: 0.00020,	2: 0.00038, 3: 0.00014,
			AZOBR_p470042		
			AZOBR_p470043	2: 0.00011, 3: 0.00033, 4: 0.00040, 5: 0.00018,	1: 0.00057, 2: 0.00056, 3: 0.00052,
			AZOBR_p470046	5: 0.00011,	1: 0.00007, 2: 0.00016, 3: 0.00010,
			AZOBR_p470061	1: 0.00007,	
			AZOBR_p470067	4: 0.00015,	
			AZOBR_p470068	2: 0.00056, 3: 0.00046, 4: 0.00056, 5: 0.00019,	3: 0.00017,
			AZOBR_p470069	1: 0.00010, 2: 0.00015, 3: 0.00028, 4: 0.00017, 5: 0.00012,	1: 0.00002, 3: 0.00003,
			AZOBR_p470070	2: 0.00026, 4: 0.00041, 5: 0.00041,	
			AZOBR_p470083	3: 0.00012,	
			AZOBR_p470098		
			AZOBR_p480004		
			AZOBR_p480018	2: 0.00005, 3: 0.00005,	
			AZOBR_p480019		1: 0.00025, 2: 0.00037, 3: 0.00028,
			AZOBR_p480025		
			AZOBR_p480027		3: 0.00025,
			AZOBR_p480032		
			AZOBR_p480035		
			AZOBR_p480054	1: 0.00010, 3: 0.00022, 4: 0.00028, 5: 0.00028,	1: 0.00023, 3: 0.00016,
			AZOBR_p480055	1: 0.00013, 2: 0.00008, 3: 0.00018, 4: 0.00012, 5: 0.00018,	2: 0.00016, 3: 0.00013,
			AZOBR_p480058		1: 0.00100, 2: 0.00049, 3: 0.00083,
			AZOBR_p480060	1: 0.00080, 2: 0.00053, 3: 0.00063, 4: 0.00055, 5: 0.00032,	1: 0.00021, 2: 0.00044, 3: 0.00046,
			AZOBR_p480077	1: 0.00043, 2: 0.00053, 3: 0.00066, 4: 0.00012, 5: 0.00030,	1: 0.00017, 2: 0.00013, 3: 0.00032,
			AZOBR_p480080	3: 0.00007, 4: 0.00004, 5: 0.00004,	1: 0.00007, 2: 0.00004, 3: 0.00007,
			AZOBR_p480081	5: 0.00009,	
			AZOBR_p480082		
			AZOBR_p480084		2: 0.00010, 3: 0.00013,
			AZOBR_p50013		
			AZOBR_p50020		2: 0.00018, 3: 0.00015,
			AZOBR_p50030	5: 0.00007,	3: 0.00015,
			AZOBR_p50063	1: 0.00025, 3: 0.00025, 4: 0.00035, 5: 0.00044,	2: 0.00028, 3: 0.00008,
			AZOBR_p50064	1: 0.00027, 4: 0.00025, 5: 0.00025,	1: 0.00021, 2: 0.00017, 3: 0.00020,
			AZOBR_p50095		2: 0.00003, 3: 0.00004,
			AZOBR_p50100		
			AZOBR_p50101	1: 0.00973, 2: 0.01268, 3: 0.00907, 4: 0.01053, 5: 0.01097,	1: 0.01128, 2: 0.00798, 3: 0.00979,
			AZOBR_p50105		
			AZOBR_p50106		2: 0.00007, 3: 0.00006,
			AZOBR_p50108		
			AZOBR_p50114	3: 0.00006,	
			AZOBR_p50121	4: 0.00003,	1: 0.00003, 3: 0.00003,
			AZOBR_p50122	3: 0.00023, 4: 0.00019, 5: 0.00031,	
			AZOBR_p50123	3: 0.00003,	
			AZOBR_p50127		3: 0.00006,
			AZOBR_p50144	4: 0.00024, 5: 0.00016,	1: 0.00015, 3: 0.00029,
			AZOBR_p50153	1: 0.00016, 4: 0.00008,	
			AZOBR_p50159		1: 0.00006, 2: 0.00007, 3: 0.00007,
			AZOBR_p50160		3: 0.00004,

			AZOBR_p50162		3: 0.00003,
			AZOBR_p50165		
			AZOBR_p60001		1: 0.00006,
			AZOBR_p60003		
			AZOBR_p60004		3: 0.00015,
			AZOBR_p60006	3: 0.00008,	1: 0.00006, 2: 0.00007, 3: 0.00006,
			AZOBR_p60009	1: 0.00005, 3: 0.00017, 4: 0.00011, 5: 0.00005,	
			AZOBR_p60010		
			AZOBR_p60011		3: 0.00002,
			AZOBR_p60012	3: 0.00015,	
			AZOBR_p60015	1: 0.00254, 2: 0.00201, 3: 0.00201, 4: 0.00144, 5: 0.00129,	1: 0.00094, 2: 0.00133, 3: 0.00105,
			AZOBR_p60018	1: 0.00041, 3: 0.00022, 4: 0.00021, 5: 0.00039,	3: 0.00006,
			AZOBR_p60019		
			AZOBR_p60020	3: 0.00013, 4: 0.00031,	3: 0.00009,
			AZOBR_p60023	1: 0.00011, 2: 0.00014, 3: 0.00023, 4: 0.00007, 5: 0.00007,	1: 0.00007, 2: 0.00008, 3: 0.00012,
			AZOBR_p60024	1: 0.00024, 3: 0.00014, 5: 0.00008,	1: 0.00005, 3: 0.00010,
			AZOBR_p60028		2: 0.00006,
			AZOBR_p60029	2: 0.00006,	
			AZOBR_p60030	5: 0.00010,	2: 0.00011, 3: 0.00009,
			AZOBR_p60031		1: 0.00003, 3: 0.00003,
			AZOBR_p60033		
			AZOBR_p60035	4: 0.00016, 5: 0.00005,	
			AZOBR_p60036		
			AZOBR_p60037	2: 0.00025,	
			AZOBR_p60041	5: 0.00007,	
			AZOBR_p60042		
			AZOBR_p60044		
			AZOBR_p60045		
			AZOBR_p60046	5: 0.00007,	3: 0.00006,
			AZOBR_p60050		
			AZOBR_p60051		
			AZOBR_p60052	2: 0.00011, 3: 0.00004, 4: 0.00003, 5: 0.00003,	1: 0.00003, 2: 0.00005, 3: 0.00003,
			AZOBR_p60055	1: 0.00038, 2: 0.00019, 3: 0.00027, 4: 0.00022, 5: 0.00026,	1: 0.00041, 2: 0.00035, 3: 0.00037,
			AZOBR_p60056	2: 0.00009, 3: 0.00009,	1: 0.00016, 2: 0.00011, 3: 0.00035,
			AZOBR_p60058		
			AZOBR_p60063		1: 0.00025, 2: 0.00029, 3: 0.00037,
			AZOBR_p60065	4: 0.00006,	1: 0.00005, 3: 0.00008,
			AZOBR_p60066		
			AZOBR_p60067	1: 0.00311, 2: 0.00379, 3: 0.00353, 4: 0.00245, 5: 0.00276,	1: 0.00209, 2: 0.00183, 3: 0.00331,
			AZOBR_p60068	1: 0.00013, 4: 0.00009, 5: 0.00005,	1: 0.00004, 3: 0.00006,
			AZOBR_p60069	1: 0.00052, 2: 0.00040, 3: 0.00050, 4: 0.00076, 5: 0.00067,	1: 0.00066, 2: 0.00042, 3: 0.00040,
			AZOBR_p60069	1: 0.00074, 2: 0.00085, 3: 0.00061, 4: 0.00105, 5: 0.00086,	1: 0.00087, 2: 0.00050, 3: 0.00087,
			AZOBR_p60070	3: 0.00017, 4: 0.00028, 5: 0.00007,	3: 0.00016,
			AZOBR_p60071	1: 0.00017, 3: 0.00022, 4: 0.00021, 5: 0.00012,	1: 0.00006, 2: 0.00022, 3: 0.00008,
			AZOBR_p60076	1: 0.00016, 3: 0.00021, 4: 0.00022,	
			AZOBR_p60078		
			AZOBR_p60080	5: 0.00007,	
			AZOBR_p60086		
			AZOBR_p60087	2: 0.00004, 3: 0.00006,	1: 0.00005, 3: 0.00003,
			AZOBR_p60091		2: 0.00007, 3: 0.00008,
			AZOBR_p60092		
			AZOBR_p60093	3: 0.00045,	
			AZOBR_p60098	4: 0.00004,	
			AZOBR_p60099		3: 0.00003,
			AZOBR_p60100	2: 0.00011,	
			AZOBR_p60109	3: 0.00001, 4: 0.00001, 5: 0.00001,	
			AZOBR_p60111	3: 0.00008,	
			AZOBR_p60112		1: 0.00015,
			AZOBR_p60120		
			AZOBR_p60124	2: 0.00005, 4: 0.00004, 5: 0.00006,	
			AZOBR_p60125	3: 0.00002,	1: 0.00003, 2: 0.00003, 3: 0.00003,

Protein abundance of *Azospirillum lipoferum* 4B and *Azospirillum brasilense* Sp245 as a fraction of the total detected protein. The abundance is displayed for each control and nitrogen fixation replicate. 4 control and 4 nitrogen fixation replicates were produced for 4B. 5 control and 3 nitrogen fixation replicates were produced for Sp245. The data is displayed as (replicate number): (fraction of detected protein). Absent replicates indicate that the protein was not detected during that run. Abundance was calculated as described in Materials and Methods.
