# Supplementary material for: Azospirillum Genomes Reveal Transition of Bacteria from Aquatic to Terrestrial Environments
Source: PLoS Genet. 2011 Dec 22;7(12):e1002430. doi: 10.1371/journal.pgen.1002430 (PMC3245306; doi:10.1371/journal.pgen.1002430)
Supplement: Table S10 — Orthologous chemotaxis operons in Azospirillum and Rhodospirillum centenum. (PDF) [file pgen.1002430.s013.pdf]

Table S10. Orthologous chemotaxis operons in *Azospirillum* and *Rhodospirillum centenum*

Chemotaxis genes	*R. centenum* SW	*A. brasilense* Sp245	*A. iipoferum* 4B	*Azospirillum* sp. B510
Operon 1 - F5				
CheA	RC1_1758	AZOBR_p1130073	AZOLI_p40444	AZL_d03050
CheW	RC1_1757	AZOBR_p1130074	AZOLI_p40443	AZL_d03040
CheY	RC1_1756	AZOBR_p1130075	AZOLI_p40442	AZL_d03030
CheB	RC1_1755	AZOBR_p1130076	AZOLI_p40441	AZL_d03020
CheR	RC1_1754	AZOBR_p1130077	AZOLI_p40440	AZL_d03010
Operon 2 - F9				
MCP	RC1_0344	AZOBR_p280105	AZOLI_p40518	AZL_d03500
CheW	RC1_0343	AZOBR_p280107	AZOLI_p40517	AZL_d03490
CheB	RC1_0342	AZOBR_p280108	AZOLI_p40516	AZL_d03480
Other (HEAT)	RC1_0341	AZOBR_p280109	AZOLI_p40515	AZL_d03470
CheR	RC1_0340	AZOBR_p280110	AZOLI_p40514	AZL_d03460
CheY	RC1_0339	AZOBR_p280111	AZOLI_p40513	AZL_d03450
CheA	RC1_0338, RC1_0337	AZOBR_p280112	AZOLI_p40512	AZL_d03440
Operon 3 - ACF				
CheY	RC1_2133			
CheW	RC1_2132	AZOBR_p1100030	AZOLI_p20369	AZL_a03160
CheR	RC1_2131, RC1_2130	AZOBR_p1100031	AZOLI_p20368	AZL_a03150
CheW	RC1_2129	AZOBR_p1100032	AZOLI_p20367	AZL_a03140
MCP	RC1_2128	AZOBR_p1100034	AZOLI_p20366	AZL_a03130
CheA	RC1_2127, RC1_2126	AZOBR_p1100035	AZOLI_p20364	AZL_a03120
CheB	RC1_2125	AZOBR_p1100037	AZOLI_p20363	AZL_a03110
RR	RC1_2124	AZOBR_p1100039	AZOLI_p20362	AZL_a03100
Operon 4 - F7				
CheY		AZOBR_200200	AZOLI_2425	AZL_023410
CheA		AZOBR_200201, AZOBR_200202	AZOLI_2426	AZL_023420
CheW		AZOBR_200203	AZOLI_2427	AZL_023430
MCP		AZOBR_200204	AZOLI_2428	
CheR		AZOBR_200205	AZOLI_2429	AZL_023450
CheD		AZOBR_200206	AZOLI_2430	AZL_023460
CheB		AZOBR_200207	AZOLI_2431	AZL_023470
MCP		AZOBR_200208	AZOLI_2432	AZL_023480
Operon 5 - Unc				
MCP			AZOLI_1666	AZL_016690
MCP			AZOLI_1665	AZL_016680
CheR			AZOLI_1664	AZL_016670
CheW			AZOLI_1663	AZL_016660

CheB			AZOLI_1662	AZL_016650
CheY			AZOLI_1661	AZL_016640
CheA			AZOLI_1660	AZL_016630
Operon 6 - F8				
CheY				AZL_a08750
CheA				AZL_a08740
CheW				AZL_a08730
MCP				AZL_a08720
CheW				AZL_a08710
CheR				AZL_a08700
CheB				AZL_a08690
MCP				AZL_a08680
