# Supplementary material for: Azospirillum Genomes Reveal Transition of Bacteria from Aquatic to Terrestrial Environments
Source: PLoS Genet. 2011 Dec 22;7(12):e1002430. doi: 10.1371/journal.pgen.1002430 (PMC3245306; doi:10.1371/journal.pgen.1002430)
Supplement: Table S11 — Classification of chemotaxis systems in rhizosphere. (PDF) [file pgen.1002430.s014.pdf]

Table S11. Classification of chemotaxis systems in rhizosphere

Clone name	Assigned chemotaxis class
cprootA02	F7
cprootA03	F8
cprootA04	F5
cprootA05	F5
cprootA07	F7
cprootA09	F5
cprootA11	F5
cprootA12	F7
cprootB01	F7
cprootB02	F7
cprootB03	F5
cprootB04	F5
cprootB05	F5
cprootB06	F2
cprootB07	Unc
cprootB08	F7
cprootB09	F5
cprootB10	F7
cprootB11	F7
cprootB12	Unc
cprootC01	F7
cprootC02	F7
cprootC03	F5
cprootC04	Unc
cprootC05	F7
cprootC06	F5
cprootC07	Unc
cprootC08	Unc
cprootC09	F8
cprootC10	F5
cprootC11	F5
cprootclone1	F3
cprootclone2	F8
cprootclone3	F5
cprootD01	F5
cprootD02	Unc
cprootD03	F7
cprootD04	ACF
cprootD08	F7
cprootD09	F7
cprootD10	F7
cprootD11	F8
cprootD12	F7
cprootE01	F7
cprootE02	F7
cprootE03	Tfp
cprootE04	F2
cprootE06	F5
cprootE07	F2
cprootE08	F8
cprootE10	F7
cprootE12	F8
cprootF01	F7
cprootF03	F7
cprootF04	F7
cprootF07	F7
cprootF08	F5
cprootF09	ACF
cprootF10	Unc
cprootF12	F7
cprootG01	F8
cprootG03	Unc
cprootG04	F7
cprootG05	F7
cprootG06	F7
cprootG07	F8
cprootG08	F5
cprootG09	F7

cprootG10	F7
cprootG11	F7
cprootG12	F5
cprootH01	F5
cprootH02	Unc
cprootH03	F2
cprootH05	F7
cprootH06	F5
cprootH07	F7
cprootH08	F5
cprootH09	F5
cprootH10	ACF
cprootH11	F7
cprootH12	F7
soilA01	F7
soilA01	F7
soilA02	Unc
soilA02N	Unc
soilA03	F7
soilA03N	F7
soilA04	F5
soilA04N	F8
soilA05	F7
soilA05N	Unc
soilA06	F7
soilA06N	F7
soilA07	F7
soilA07N	Unc
soilA08	Unc
soilA08N	F7
soilA09	F7
soilA09N	Unc
soilA10N	F5
soilA11	Unc
soilA11N	F7
soilA12	F7
soilA12N	Tfp
soilB01	F7
soilB01N	F2
soilB02	F5
soilB03	F5
soilB03N	F7
soilB04	F7
soilB04N	Tfp
soilB05	F2
soilB05N	Tfp
soilB06	F6
soilB07	F7
soilB08N	F7
soilB09	F7
soilB09N	F5
soilB10	Unc
soilB10N	F8
soilB11	F2
soilB11N	F7
soilB12	Unc
soilB12N	F5
soilC01	F7
soilC01N	Unc
soilC02	F8
soilC02N	F2
soilC03	F7
soilC03N	F7
soilC04	F2
soilC04N	F7
soilC05	F7
soilC05N	F7
soilC06	F8
soilC06N	F7
soilC07	F7
soilC08	F5
soilC08N	F7
soilC09	F7
soilC10	F7
soilC11	F2

soilC11N	F7
soilC12	Unc
soilC12N	F7
soilCL1	F7
soilCL2	F7
soilCL2B	F7
soilCL3	F7
soilCL3b	F7
soilCL4b	F5
soilCL5b	F2
soilclone10	F7
soilclone2	F8
soilclone3	F2
soilclone5	F2
soilclone6	F8
soilclone7	F2
soilclone8a	F8
soilclone9	F7
soilD01	F6
soilD01N	F7
soilD02	F2
soilD02N	F7
soilD03	F7
soilD03N	F5
soilD04	F8
soilD04N	F13
soilD05	F7
soilD05N	Unc
soilD06	F7
soilD06N	F5
soilD07N	F7
soilD08	F5
soilD08a	F7
soilD09	F7
soilD09N	F7
soilD10	F5
soilD10N	F7
soilD11	F7
soilD11N	F7
soilD12	F7
soilD12N	F7
soilE02N	Tfp
soilE03N	F7
soilE04N	F5
soilE05N	F2
soilE06N	F7
soilE07N	F7
soilE08N	F5
soilE10N	F7
soilE12N	F5
soilF02N	Tfp
soilF03N	F7
soilF04	F13
soilF05N	F7
soilF06N	Unc
soilF07N	F5
soilF08N	F5
soilF09N	Unc
soilF10N	F13
soilF11N	F7
soilF12N	F5
soilG01N	F7
soilG04N	Unc
soilG05N	Unc
soilG06	F7
soilG07N	F6
soilG08N	F7
soilG09N	F1
soilG10N	F7
soilG11N	F7
soilG12	Unc
soilH01N	F13
soilH02N	F5
soilH03N	F5
soilH05N	F7

soilH06N	F7
soilH07N	Unc
soilH08N	F7
soilH10N	Unc
soilH11N	F5
soilH12N	F7
whtrootA01	F5
whtrootA02	Unc
whtrootA03	F7
whtrootA04	F5
whtrootA06	F8
whtrootA07	F7
whtrootA08	F8
whtrootA10	F7
whtrootA12	F7
whtrootB01	F8
whtrootB05	F7
whtrootB06	F7
whtrootB07	F8
whtrootB08	Unc
whtrootB09	F7
whtrootB10	F7
whtrootB11	F5
whtrootB12	F7
whtrootC01	F2
whtrootC02	F7
whtrootC03	F8
whtrootC04	F7
whtrootC05	F7
whtrootC06	F7
whtrootC07	F8
whtrootC08	F7
whtrootC09	F8
whtrootC10	F13
whtrootC12	F7
whtrootclone2	F7
whtrootclone3	F8
whtrootclone4	F7
whtrootclone6	F8
whtrootD01	F5
whtrootD02	F2
whtrootD05	Unc
whtrootD07	F7
whtrootD09	F6
whtrootD10	F1
whtrootD11	F8
whtrootD12	F8
whtrootE01	F7
whtrootE02	F7
whtrootE03	F7
whtrootE04	F8
whtrootE05	F7
whtrootE06	F8
whtrootE07	F13
whtrootE09	F8
whtrootE10	F7
whtrootE11	F7
whtrootE12	F8
whtrootF01	F13
whtrootF02	F7
whtrootF03	F7
whtrootF04	F7
whtrootF05	F8
whtrootF06	F8
whtrootF08	F7
whtrootF09	F5
whtrootF10x429bpFOR	F7
whtrootF11	F7
whtrootF12	F8
whtrootG01	F7
whtrootG03	Unc
whtrootG04	F7
whtrootG05	F5
whtrootG06	Unc
whtrootG08	F7

whtrootG09	F7
whtrootG10	F2
whtrootG11	F7
whtrootG12	F7
whtrootH02	F2
whtrootH03	F8
whtrootH04	F7
whtrootH06	F5
whtrootH07	F7
whtrootH10	F7
whtrootH12	Unc

Clones were classified as described in Materials and Methods.
