# Supplementary material for: Azospirillum Genomes Reveal Transition of Bacteria from Aquatic to Terrestrial Environments
Source: PLoS Genet. 2011 Dec 22;7(12):e1002430. doi: 10.1371/journal.pgen.1002430 (PMC3245306; doi:10.1371/journal.pgen.1002430)
Supplement: Table S12 — Putative complex carbohydrate-degrading enzymes in three Azospirillum species in comparison with a soil cellulolytic bacterium Thermobifida fusca. (PDF) [file pgen.1002430.s015.pdf]

Table S12. Putative complex carbohydrate-degrading enzymes in three *Azospirillum* species in comparison with a soil cellulolytic bacterium *Thermobifida fusca*

**CAZy family**	**Putative activity**	***A. lipoferum* 4B**	***A. brasilense* Sp245**	***A.* sp. B510**	***T. fusca***
GH 1	β-glucosidase, cellobiase	4	4	4	2
GH 2	β-mannosidase, β-glucruonidase, galactosidase	1	3	1	1
GH 3	Xylosidase, β -N-acetylhexosaminidase	1	1	2	2
GH 4	Glucuronidase, galactosidase, glucosidase	1	1	1	1
GH 5	Mannanase	0	0	0	1
GH 5	Cellulase, endogluconase	2	3	2	2
GH 6	Endogluconase	0	0	0	2
GH 8	Cellulase, endogluconase	1	0	1	0
GH 9	Endogluconase	0	0	0	2
GH 10, GH11	Endoxylanase, xylanase	1	2	1	3
GH 12	Endogluconase	0	1	0	0
GH 13, GH15	Amylase, pullulanase, dextranase	10	14	8	8
GH 16	Lichninase	1	0	1	0
GH 48	1,4-exocellulase	0	0	0	1
GH 17, GH25	Other	4	5	5	12
All	Glycosyl hydrolases	26	34	26	37

Proteins were assigned to CAZy families as described in Materials and Methods.
